# Supplementary material for: miR-200c-3p, miR-222-5p, and miR-512-3p Constitute a Biomarker Signature of Sorafenib Effectiveness in Advanced Hepatocellular Carcinoma
Source: Cells. 2022 Aug 28;11(17):2673. doi: 10.3390/cells11172673 (PMC9454520; doi:10.3390/cells11172673)
Supplement: Supplementary file 1 [file cells-11-02673-s001.zip › cells-1858257-Supplementary-revised.pdf]

SUPPLEMENTARY MATERIAL

miR-200c-3p, miR-222-5p and miR-512-3p constitute a biomarker signature of Sorafenib effectiveness in advanced hepatocellular carcinoma

Patricia de la Cruz-Ojeda[1,2,3], Tobias Schmid[4], Loreto Boix[2,5], Manuela Moreno[6], Víctor Sapena[5], Juan M. Praena-Fernández[7], Francisco J. Castell[8], Juan Manuel Falcón-Pérez[2,9], María Reig[2,5], Bernhard Brüne[4,10,11,12], Miguel A. Gómez-Bravo[6], Alvaro Giráldez[13], Jordi Bruix[2,5], María T. Ferrer[13], Jordi Muntané[1,2,3,*]

[1]Institute of Biomedicine of Seville (IBiS), Hospital University "Virgen del Rocío"/CSIC/University of Seville, Seville, 41013, Spain

[2]Networked Biomedical Research Center Hepatic and Digestive Diseases (CIBEREHD)

[3]Department of Medical Physiology and Biophysics, University of Seville, Seville, 41004, Spain

[4]Institute of Biochemistry I, Faculty of Medicine, Goethe-University Frankfurt, Frankfurt, 60528, Germany

[5]BCLC Group, Liver Unit, Hospital Clinic, University of Barcelona, IDIBAPS, CIBEREHD, Barcelona, 08036, Spain

[6]Department of General Surgery, Hospital University "Virgen del Rocío"/CSIC/University of Seville/IBIS, Seville, 41013, Spain

[7]Department of Statistics and Operations Research, University of Granada, Granada, 18011, Spain

[8]Department of Radiology, Hospital University "Virgen del Rocío"/CSIC/University of Seville/IBIS, Seville, 41013, Spain

[9]Exosomes Lab, CIC bioGUNE, Derio, 48160, Spain

[10]German Cancer Consortium (DKTK), Partner Site Frankfurt, Frankfurt, 60528, Germany

[11]Frankfurt Cancer Institute, Goethe-University Frankfurt, Frankfurt, 60528, Germany

[12]Fraunhofer Institute for Translational Medicine and Pharmacology ITMP, Frankfurt, 60528, Germany

[13]Unit for the Clinical Management of Digestive Diseases, Hospital University "Virgen del Rocío"/CSIC/University of Seville/IBIS, Seville, 41013, Spain

*Correspondencejmuntane-ibis@us.es; Tel. +34-955923122; Fax. +34-955923002

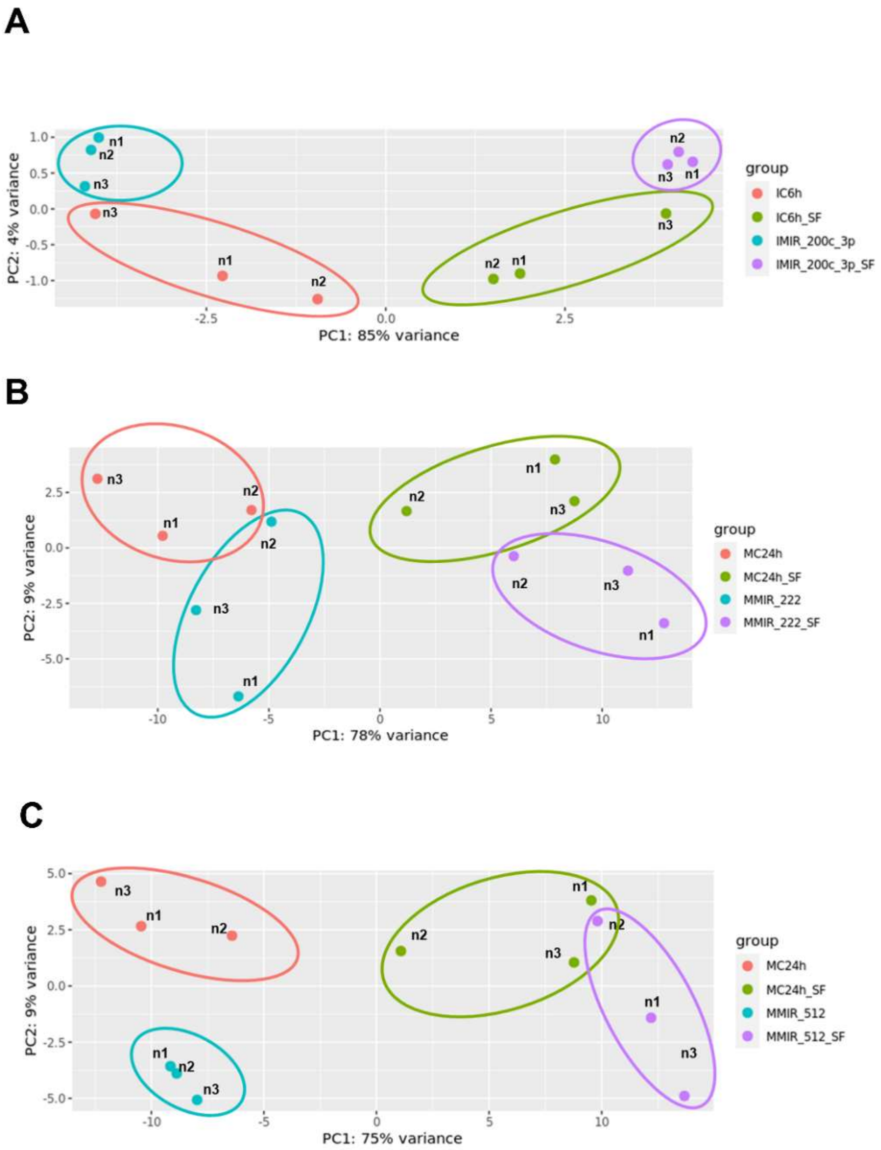

Figure S1. Principal component analysis (PCA) of RNA-Seq data. (A) PCA analysis of differentially expressed genes (p-adj<0.05) in miR-200c-3p inhibitor and control transfected cells, treated with Sorafenib. **(B)** PCA analysis of differentially expressed genes (p-adj<0.05) in miR-222-5p and control transfected cells, treated with Sorafenib. **(C)** PCA analysis of differentially expressed genes (p-adj<0.05) in miR-512-3p mimic and control transfected cells, treated with Sorafenib.

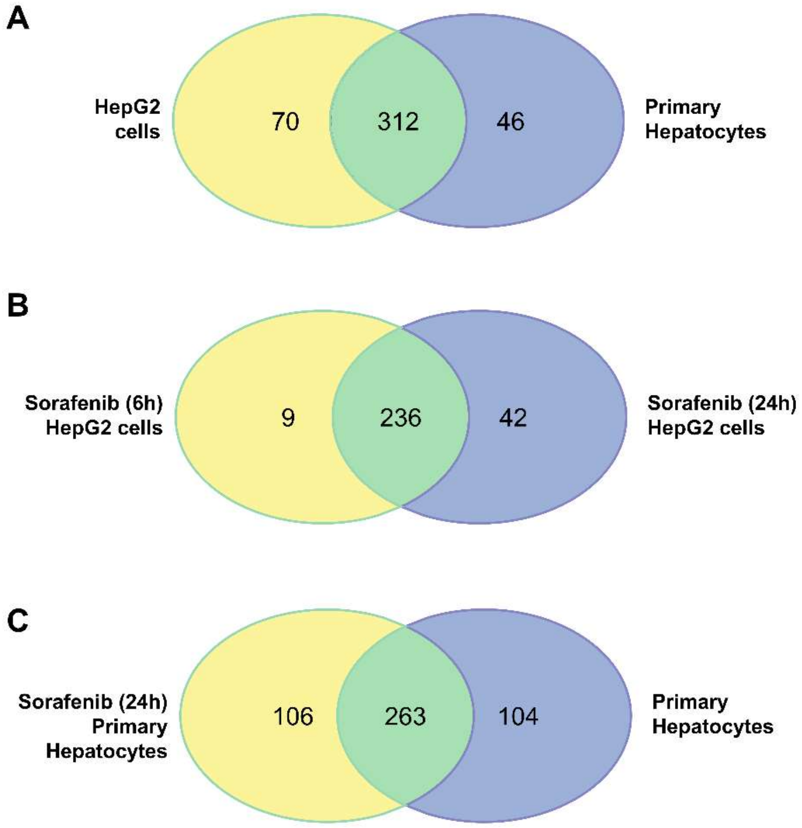

Figure S2. Venn analysis of microarray data. **(A)** Comparison of HepG2 cells and primary human hepatocytes. **(B)** Comparison of miRNAs in HepG2 cells treated with Sorafenib for 6 and 24 h. **(C)** miRNAs induced by Sorafenib (24 h) in primary hepatocytes, compared to non-treated cells.

A

miRNA	Fold-Change	Corrected p-value
hsa-let-7d-5p	0.092	0.019
hsa-let-7g-5p	0.161	0.029
hsa-miR-17-5p	6.754	0.021
hsa-miR-18a-5p	15.702	0.008
hsa-miR-20a-5p	5.167	0.031
hsa-miR-34a-5p	3.582	0.03
hsa-miR-92a-3p	4.49	0.027
hsa-miR-100-5p	0.004	0.008
hsa-miR-106a-5p	6.279	0.015
hsa-miR-106b-5p	7.935	0.014
hsa-miR-130b-3p	17.28	0.019
hsa-miR-149-5p	4.547	0.015
hsa-miR-150-5p	0.136	0.029
hsa-miR-193a-5p	0.02	0.012
hsa-miR-195-5p	0.06	0.011
hsa-miR-204-5p	0.133	0.027
hsa-miR-221-3p	34.979	0.03
hsa-miR-301a-3p	202.186	0.005
hsa-miR-324-3p	4.614	0.031
hsa-miR-324-5p	14.068	0.015
hsa-miR-339-3p	9.326	0.023
hsa-miR-339-5p	2.987	0.007
hsa-miR-361-5p	4.928	0.033
hsa-miR-362-5p	7.719	0.044
hsa-miR-375	0.037	0.007
hsa-miR-425-5p	4.532	0.027
hsa-miR-483-5p	7.97	0.017
hsa-miR-500a-5p	3.783	0.043
hsa-miR-532-3p	6.617	0.007
hsa-miR-532-5p	4.532	0.037
hsa-miR-660-5p	3.1	0.016
hsa-miR-744-5p	6.633	0.014
hsa-miR-885-5p	0.068	0.01

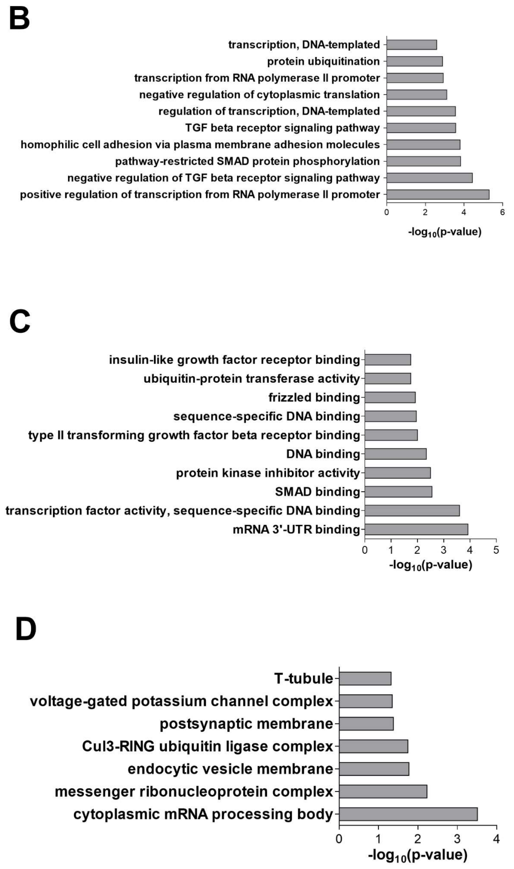

Figure S3. miRNA expression in HepG2 cells, compared to primary hepatocytes. **(A)** Summary Table of significant differentially expressed miRNAs in tumor cells, showing fold-change and corrected p-value. Up-regulated miRNAs are shown in red and down-regulated miRNAs are shown in green. **(B-D)** Top 10 gene ontology (GO) terms biological process, molecular function and cellular component significantly enriched in miRNA targets, respectively.

A

miRNA	Fold-Change	p-value
hsa-miR-29c-3p	3.076	0.009
hsa-miR-376a-3p	0.405	0.042
hsa-miR-671-3p	4.779	0.036

B

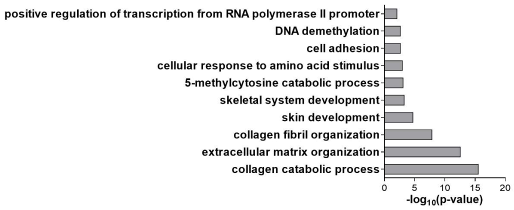

C

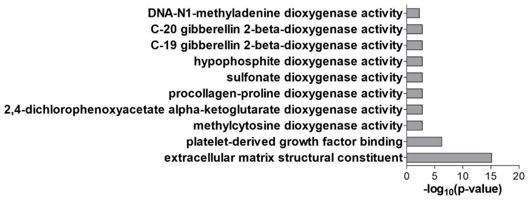

D

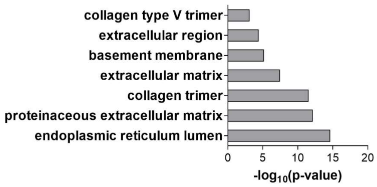

Figure S4. miRNA expression profile in Sorafenib treated primary hepatocytes. **(A)** Summary Table of significant differentially expressed miRNAs after Sorafenib treatment, showing fold-change and p-value. Up-regulated miRNAs are shown in red and down-regulated miRNAs are shown in green. **(B-D)** Top 10 gene ontology (GO) terms biological process, molecular function and cellular component significantly enriched in miRNA targets, respectively.

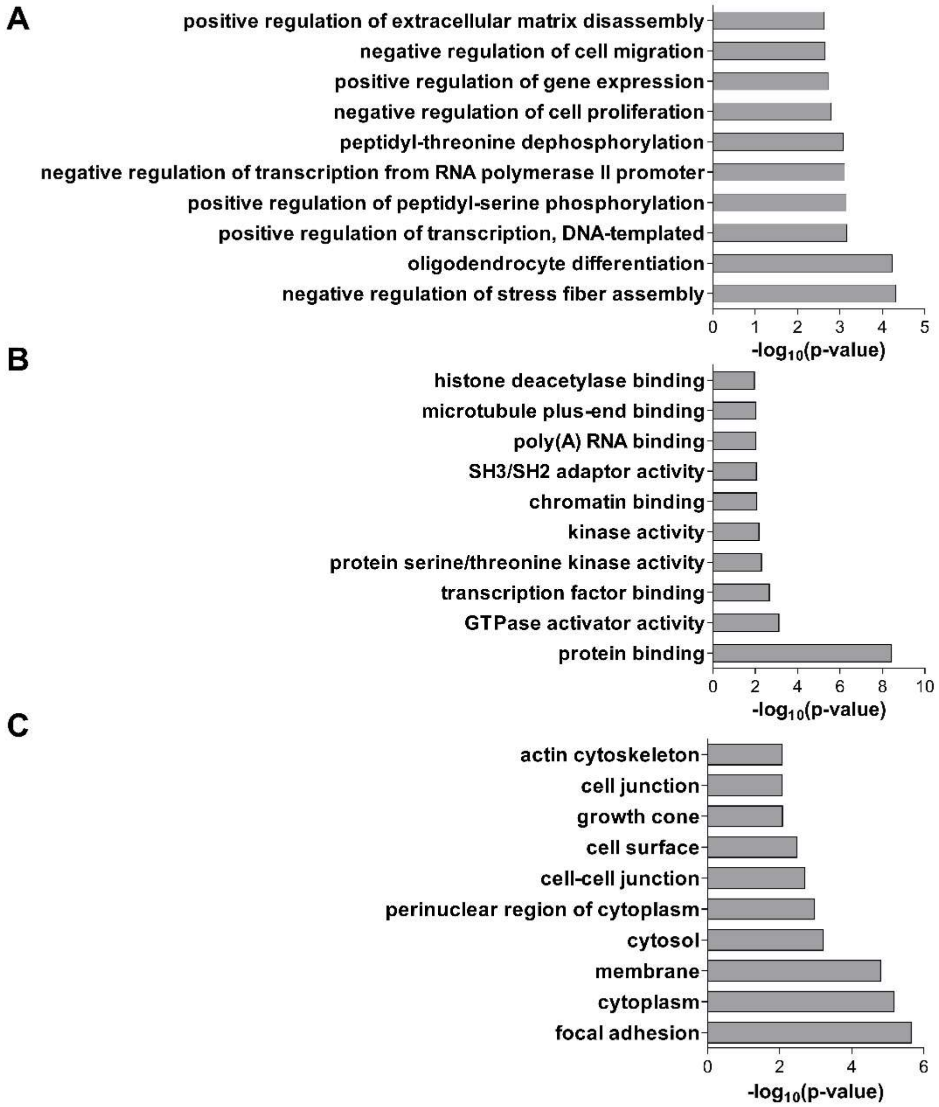

Figure S5. (A-C) Top 10 gene ontology (GO) biological process, molecular function and cellular component terms significantly enriched in targets of miRNAs differentially expressed at 6 h of Sorafenib treatment, respectively.

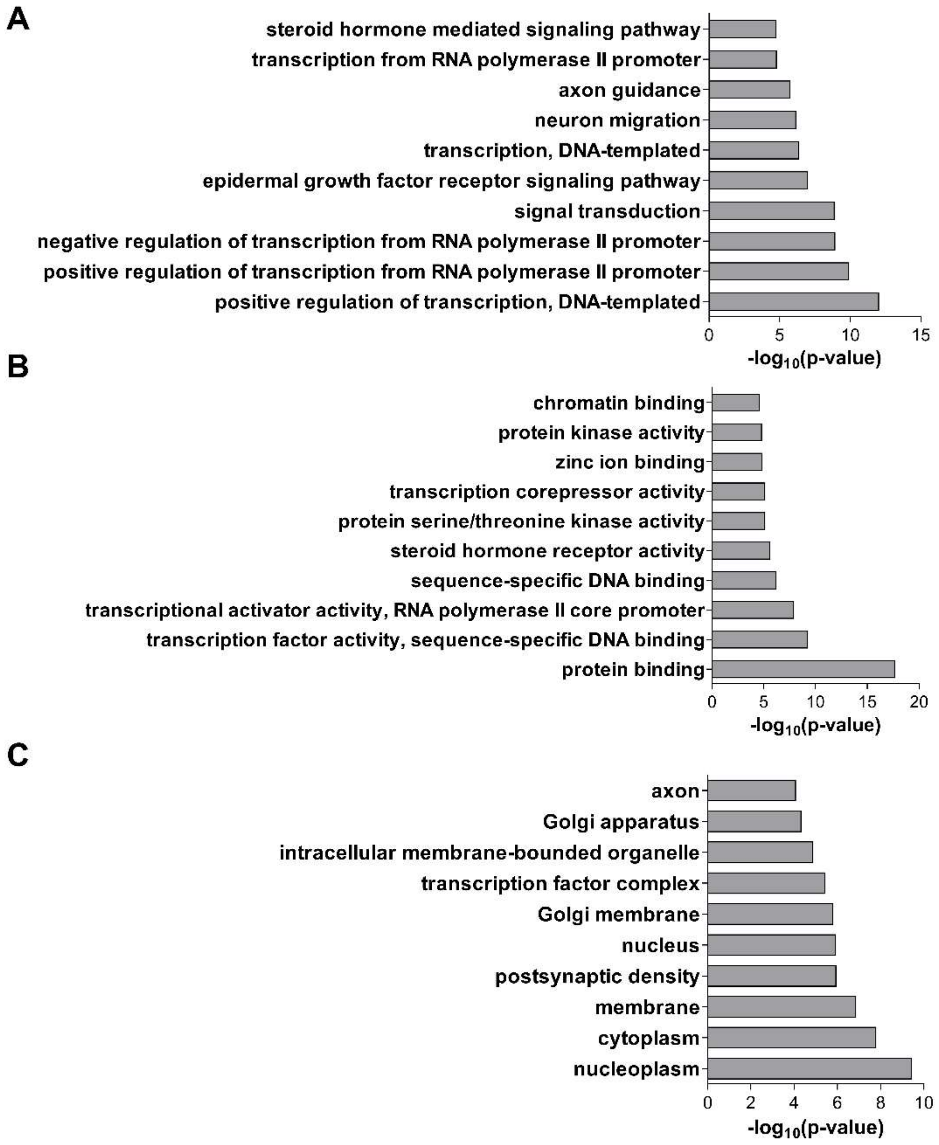

Figure S6. (A-C) Top 10 gene ontology (GO) biological process, molecular function and cellular component terms significantly enriched in targets of miRNAs differentially expressed at 24 h of Sorafenib treatment, respectively.

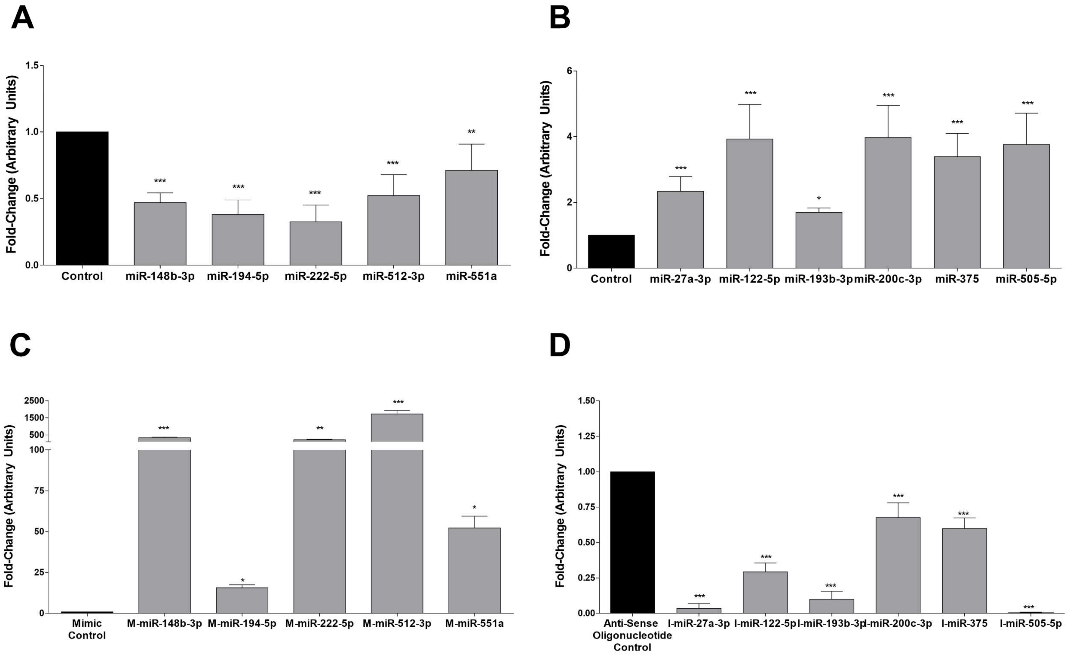

Figure S7. miRNA validation**. (A)** q-PCR validation of down-regulated miRNAs in Sorafenib treated HepG2 cells. **(B)** q-PCR validation of upregulated miRNAs in Sorafenib treated HepG2 cells. **(C)** miRNA expression values after miRNA mimics of down-regulated miRNAs. **(D)** miRNA expression values after miRNA inhibition of up-regulated miRNAs. * $p \leq 0.05$ ** $p \leq 0.01$, and *** $p \leq 0.001$ between control and miRNA tested.

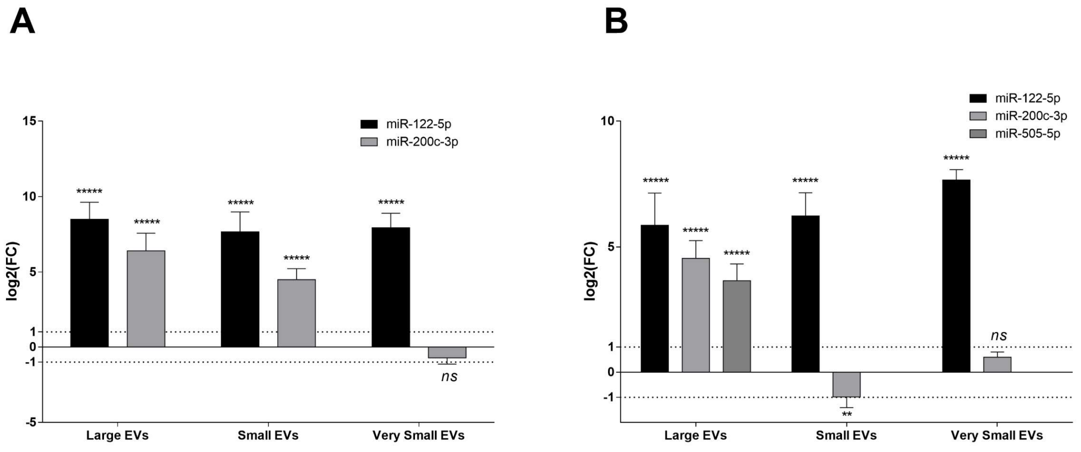

Figure S8. miRNA expression values in extracellular vesicles (EVs) compared to cellular content at 6 h**. (A)** miRNA expression in Large, Small and Very Small EVs related to intracellular content in control cells (6 h). **(B)** miRNA expression in Large, Small and Very Small EVs related to intracellular content in Sorafenib treated cells (6 h). Ns, non-significant; ** $p \leq 0.01$, and *** $p \leq 0.0001$ between the miRNA expression in the EVs fractions compared to cellular levels.

A

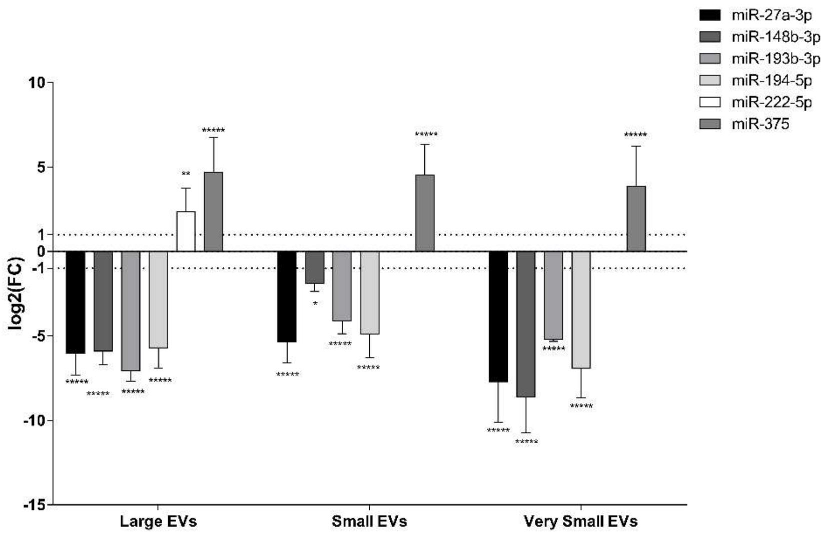

B

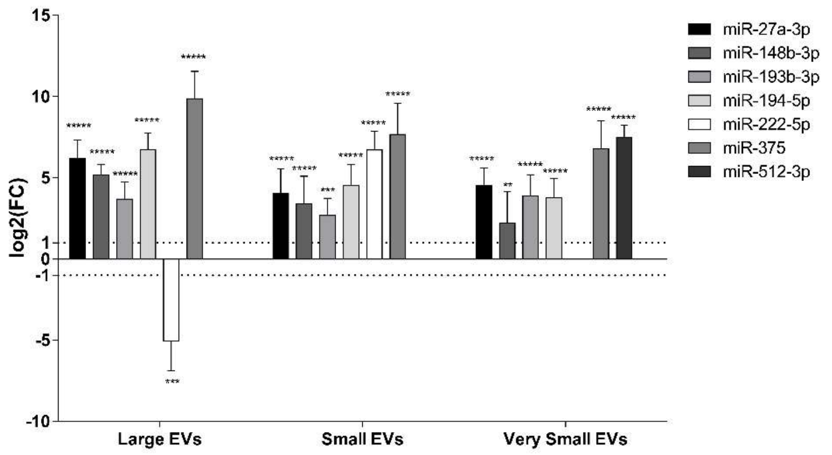

Figure S9. miRNA expression values in extracellular vesicles (EVs) compared to cellular content at 24 h. **(A)** miRNA expression in Large, Small and Very Small EVs related to intracellular content in control cells (24 h). **(B)** miRNA expression in Large, Small and Very Small EVs related to intracellular content in Sorafenib treated cells (24 h). * $p \leq 0.05$, ** $p \leq 0.01$, *** $p \leq 0.001$, and *** $p \leq 0.0001$ between the miRNA expression in the EVs fractions compared to cellular levels.

Table S1. Comparison of demographic and epidemiologic data of the study cohort (n=36), and the validation cohort (n=81).

	**Study Cohort (n=36)**	**Validation Cohort (n=81)**
**Age, median [25th; 75th]**	67.1 [56.8; 73.9]	64.1 [54.4; 70.3]
**Male, % (n)**	83.3 (30)	85.2 (69)
**Exitus, % (n)**	66.7 (24)	95.1 (77)
**Survival (months), median [25th; 75th]**	10.4 [6.1; 20.6]	11.4 [6.9; 20.1]
**BCLC Stage, % (n)**		
B	25.0 (9)	41.9 (34)
C	75.0 (27)	58.0 (47)
**Cirrhosis Stage, % (n)**		
Child-Pugh Class A	63.9 (23)	81.5 (66)
Child-Pugh Class B	16.7 (6)	14.8 (12)
Non-cirrhotic liver	19.4 (7)	3.7 (3)
**Length of Sorafenib treatment, median [25th; 75th]**	5.5 [4.0; 9.0]	6.7 [3.0; 10.3]
**Extrahepatic Metastasis, %(n)**	36.1 (13)	23.5 (19)
**Portal vein invasion, % (n)**	44.4 (16)	38.3 (31)
**Total Bilirubin median (mg/dL), [25th; 75th]**	0.9 [0.5; 1.2]	1.1 [0.7; 1.6]
**Alanine aminotransferase (U/L), median [25th; 75th]**	36.0 [23.0; 65.0]	66.0 [46.0; 109.5]

**Aspartate aminotransferase (U/L), median [25th; 75th]**	51.0 [35.0; 67.0]	71.5 [48.5; 109.0]
**Alkaline phosphatase (U/L), median [25th; 75th]**	141.0 [85.3; 193.5]	320.0 [227.5; 459.0]
**Gamma-glutamyl transferase (U/L), median [25th; 75th]**	153.0 [63.0; 234.0]	161.5 [83.5; 280.5]
**Albumin (g/dL), median [25th; 75th]**	4.0 [3.6; 4.3]	3.8 [3.6; 4.2]
**Alfa-fetoprotein (ng/mL), median [25th; 75th]**	215.5 [19.3; 1462.0]	26.0 [6.0; 233]

Table S2. miRNA target prediction according to TargetScan and miRDB databases. UniProt entry ID, gene name and description are provided.

Database	miRNA	UniProt entry	Gene Name	Description
**TargetScan**	**miR-122-5p Upregulated 6 hours**	O60241	ADGRB2	Adhesion G protein-coupled receptor B2 (Brain-specific angiogenesis inhibitor 2)
		P04075	ALDOA	Fructose-bisphosphate aldolase A (EC 4.1.2.13) (Lung cancer antigen NY-LU-1) (Muscle-type aldolase)
		Q8IX12	CCAR1	Cell division cycle and apoptosis regulator protein 1 (Cell cycle and apoptosis regulatory protein 1) (CARP-1) (Death inducer with SAP domain)
		P51959	CCNG1	Cyclin-G1 (Cyclin-G)
		Q9NPF0	CD320	CD320 antigen (8D6 antigen) (FDC-signaling molecule 8D6) (FDC-SM-8D6) (Transcobalamin receptor) (TCblR) (CD antigen CD320)
		O00299	CLIC1	Chloride intracellular channel protein 1 (Chloride channel ABP) (Nuclear chloride ion channel 27) (NCC27) (Regulatory nuclear chloride ion channel protein) (hRNCC)
		Q9Y696	CLIC4	Chloride intracellular channel protein 4 (Intracellular chloride ion channel protein p64H1)
		P29762	CRABP1	Cellular retinoic acid-binding protein 1 (Cellular retinoic acid-binding protein I) (CRABP-I)
		O95476	CTDNEP1	CTD nuclear envelope phosphatase 1 (EC 3.1.3.16) (Serine/threonine-protein phosphatase dullard)
		Q13115	DUSP4	Dual specificity protein phosphatase 4 (EC 3.1.3.16) (EC 3.1.3.48) (Dual specificity protein phosphatase hVH2) (Mitogen-activated protein kinase phosphatase 2) (MAP kinase phosphatase 2) (MKP-2)
		P01588	EPO	Erythropoietin (Epoetin)

		P11678	EPX	Eosinophil peroxidase (EPO) (EC 1.11.1.7) [Cleaved into: Eosinophil peroxidase light chain; Eosinophil peroxidase heavy chain]
		Q01167	FOXK2	Forkhead box protein K2 (G/T-mismatch specific binding protein) (nGTBP) (Interleukin enhancer-binding factor 1)
		Q9BWH2	FUNDC2	FUN14 domain-containing protein 2 (Cervical cancer proto-oncogene 3 protein) (HCC-3) (Hepatitis C virus core-binding protein 6)
		Q9BUM1	G6PC3	Glucose-6-phosphatase 3 (G-6-Pase 3) (G6Pase 3) (EC 3.1.3.9) (Glucose-6-phosphatase beta) (G6Pase-beta) (Ubiquitous glucose-6-phosphatase catalytic subunit-related protein)
		Q9NSI5	IGSF5	Immunoglobulin superfamily member 5 (IgSF5) (Junctional adhesion molecule 4) (JAM-4)
		P48740	MASP1	Mannan-binding lectin serine protease 1 (EC 3.4.21.-) (Complement factor MASP-3) (Complement-activating component of Ra-reactive factor) (Mannose-binding lectin-associated serine protease 1) (MASP-1) (Mannose-binding protein-associated serine protease) (Ra-reactive factor serine protease p100) (RaRF) (Serine protease 5) [Cleaved into: Mannan-binding lectin serine protease 1 heavy chain; Mannan-binding lectin serine protease 1 light chain]
		Q86XE3	MICU3	Calcium uptake protein 3, mitochondrial (EF-hand domain-containing family member A2)
		Q9BSH3	NICN1	Nicolin-1 (NPCEDRG) (Tubulin polyglutamylase complex subunit 5) (PGs5)
		P55786	NPEPPS	Puromycin-sensitive aminopeptidase (PSA) (EC 3.4.11.14) (Cytosol alanyl aminopeptidase) (AAP-S)
		P13674	P4HA1	Prolyl 4-hydroxylase subunit alpha-1 (4-PH alpha-1) (EC 1.14.11.2) (Procollagen-proline,2-oxoglutarate-4-dioxygenase subunit alpha-1)

		Q16654	PDK4	[Pyruvate dehydrogenase (acetyl-transferring)] kinase isozyme 4, mitochondrial (EC 2.7.11.2) (Pyruvate dehydrogenase kinase isoform 4)
		Q96S52	PIGS	GPI transamidase component PIG-S (Phosphatidylinositol-glycan biosynthesis class S protein)
		Q96CS7	PLEKHB2	Pleckstrin homology domain-containing family B member 2 (PH domain-containing family B member 2) (Evectin-2)
		O75569	PRKRA	Interferon-inducible double-stranded RNA-dependent protein kinase activator A (PKR-associated protein X) (PKR-associating protein X) (Protein activator of the interferon-induced protein kinase) (Protein kinase, interferon-inducible double-stranded RNA-dependent activator)
		P82980	RBP5	Retinol-binding protein 5 (Cellular retinol-binding protein III) (CRBP-III) (HRBPiso)
		O00287	RFXAP	Regulatory factor X-associated protein (RFX-associated protein) (RFX DNA-binding complex 36 kDa subunit)
		Q86UR5	RIMS1	Regulating synaptic membrane exocytosis protein 1 (Rab-3-interacting molecule 1) (RIM 1) (Rab-3-interacting protein 2)
		Q15758	SLC1A5	Neutral amino acid transporter B(0) (ATB(0)) (Baboon M7 virus receptor) (RD114/simian type D retrovirus receptor) (Sodium-dependent neutral amino acid transporter type 2) (Solute carrier family 1 member 5)
		Q6PIV7	SLC25A34	Solute carrier family 25 member 34
		Q9HAB3	SLC52A2	Solute carrier family 52, riboflavin transporter, member 2 (Porcine endogenous retrovirus A receptor 1) (PERV-A receptor 1) (Protein GPR172A) (Riboflavin transporter 3) (hRFT3)

		P30825	SLC7A1	High affinity cationic amino acid transporter 1 (CAT-1) (CAT1) (Ecotropic retroviral leukemia receptor homolog) (Ecotropic retrovirus receptor homolog) (Solute carrier family 7 member 1) (System Y+ basic amino acid transporter)
		Q9H4F1	ST6GALNAC4	Alpha-N-acetyl-neuraminyl-2,3-beta-galactosyl-1,3-N-acetyl-galactosaminide alpha-2,6-sialyltransferase (EC 2.4.99.7) (NeuAc-alpha-2,3-Gal-beta-1,3-GalNAc-alpha-2,6-sialyltransferase) (ST6GalNAc IV) (ST6GalNAcIV) (Sialyltransferase 3C) (SIAT3-C) (Sialyltransferase 7D) (SIAT7-D)
		Q16650	TBR1	T-box brain protein 1 (T-brain-1) (TBR-1) (TES-56)
		Q17R98	ZNF827	Zinc finger protein 827
**miRDB**		Q8NFV4	ABHD11	abhydrolase domain containing 11
		O14672	ADAM10	ADAM metallopeptidase domain 10
		O60241	ADGRB2	adhesion G protein-coupled receptor B2
		P30520	ADSS	adenylosuccinate synthase
		Q8N6S4	ANKRD13C	ankyrin repeat domain 13C
		Q9Y6D6	ARFGEF1	ADP ribosylation factor guanine nucleotide exchange factor 1
		P55201	BRPF1	bromodomain and PHD finger containing 1
		Q5BKX5	C19orf54	chromosome 19 open reading frame 54
		Q9NYP8	C21orf62	chromosome 21 open reading frame 62
		Q9NS85	CA10	carbonic anhydrase 10
		Q7Z3E2	CCDC186	coiled-coil domain containing 186
		P51959	CCNG1	cyclin G1
		Q9NPF0	CD320	CD320 molecule
		P29965	CD40LG	CD40 ligand
		Q9Y696	CLIC4	chloride intracellular channel 4
		Q9NZA1	CLIC5	chloride intracellular channel 5
		Q9BZB8	CPEB1	cytoplasmic polyadenylation element binding protein 1

		O75390	CS	citrate synthase
		O95476	CTDNEP1	CTD nuclear envelope phosphatase 1
		Q9UI47	CTNNA3	catenin alpha 3
		P39880	CUX1	cut like homeobox 1
		Q6UW02	CYP20A1	cytochrome P450 family 20 subfamily A member 1
		Q16832	DDR2	discoidin domain receptor tyrosine kinase 2
		Q9UPY3	DICER1	dicer 1, ribonuclease III
		Q9P225	DNAH2	dynein axonemal heavy chain 2
		Q5T9C2	FAM102A	family with sequence similarity 102 member A
		Q13451	FKBP5	FKBP prolyl isomerase 5
		O75369	FLNB	filamin B
		Q9BWH2	FUNDC2	FUN14 domain containing 2
		P11413	G6PD	glucose-6-phosphate dehydrogenase
		P34903	GABRA3	gamma-aminobutyric acid type A receptor alpha3 subunit
		Q10472	GALNT1	polypeptide N-acetylgalactosaminyltransferase 1
		Q9Y2X7	GIT1	GIT ArfGAP 1
		Q8TDQ7	GNPDA2	glucosamine-6-phosphate deaminase 2
		P30550	GRPR	gastrin releasing peptide receptor
		P13807	GYS1	glycogen synthase 1
		Q5T447	HECTD3	HECT domain E3 ubiquitin protein ligase 3
		Q9P2P5	HECW2	HECT, C2 and WW domain containing E3 ubiquitin protein ligase 2
		Q00839	HNRNPU	heterogeneous nuclear ribonucleoprotein U
		Q9NSI5	IGSF5	immunoglobulin superfamily member 5
		Q9UGI6	KCNN3	potassium calcium-activated channel subfamily N member 3
		P52732	KIF11	kinesin family member 11
		P11047	LAMC1	laminin subunit gamma 1
		Q12852	MAP3K12	mitogen-activated protein kinase kinase kinase 12
		Q03112	MECOM	MDS1 and EVI1 complex locus

		Q86XE3	MICU3	mitochondrial calcium uptake family member 3
		Q8TD10	MIPOL1	mirror-image polydactyly 1
		Q9UJG1	MOSPD1	motile sperm domain containing 1
		B2RTY4	MYO9A	myosin IXA
		Q8IZQ8	MYOCD	myocardin
		Q96MY1	NOL4L	nucleolar protein 4 like
		Q16625	OCLN	occludin
		P13674	P4HA1	prolyl 4-hydroxylase subunit alpha 1
		Q16654	PDK4	pyruvate dehydrogenase kinase 4
		P48426	PIP4K2A	phosphatidylinositol-5-phosphate 4-kinase type 2 alpha
		P14618	PKM	pyruvate kinase M1/2
		Q96HE9	PRR11	proline rich 11
		O95758	PTBP3	polypyrimidine tract binding protein 3
		Q3YEC7	RABL6	RAB, member RAS oncogene family like 6
		Q86VV4	RANBP3L	RAN binding protein 3 like
		Q8IY67	RAVER1	ribonucleoprotein, PTB binding 1
		P28749	RBL1	RB transcriptional corepressor like 1
		Q6ZSC3	RBM43	RNA binding motif protein 43
		A0AV96	RBM47	RNA binding motif protein 47
		Q15434	RBMS2	RNA binding motif single stranded interacting protein 2
		Q9BRL7	SEC22C	SEC22 homolog C, vesicle trafficking protein
		Q92854	SEMA4D	semaphorin 4D
		P58004	SESN2	sestrin 2
		Q149N8	SHPRH	SNF2 histone linker PHD RING helicase
		Q6PIV7	SLC25A34	solute carrier family 25 member 34
		Q2Y0W8	SLC4A8	solute carrier family 4 member 8
		Q9HAB3	SLC52A2	solute carrier family 52 member 2
		P30825	SLC7A1	solute carrier family 7 member 1
		Q8TBB6	SLC7A14	solute carrier family 7 member 14

		Q9H2Y9	SLCO5A1	solute carrier organic anion transporter family member 5A1
		Q96GM5	SMARCD1	SWI/SNF related, matrix associated, actin dependent regulator of chromatin, subfamily d, member 1
		Q8IYR2	SMYD4	SET and MYND domain containing 4
		O43597	SPRY2	sprouty RTK signaling antagonist 2
		Q93045	STMN2	stathmin 2
		Q4KMP7	TBC1D10B	TBC1 domain family member 10B
		Q9NU19	TBC1D22B	TBC1 domain family member 22B
		Q16650	TBR1	T-box, brain 1
		Q8WVM0	TFB1M	transcription factor B1, mitochondrial
		Q7Z4N2	TRPM1	transient receptor potential cation channel subfamily M member 1
		Q9UHF7	TRPS1	transcriptional repressor GATA binding 1
		Q9BQE3	TUBA1C	tubulin alpha 1c
		O94888	UBXN7	UBX domain protein 7
		Q15836	VAMP3	vesicle associated membrane protein 3
		O94967	WDR47	WD repeat domain 47
		O14771	ZNF213	zinc finger protein 213
		Q17R98	ZNF827	zinc finger protein 827
		Q86W11	ZSCAN30	zinc finger and SCAN domain containing 30
**TargetScan**	**miR-148-3p Downregulated 24 hours**	Q9HCK5	AGO4	Protein argonaute-4 (Argonaute4) (hAgo4) (Argonaute RISC catalytic component 4) (Eukaryotic translation initiation factor 2C 4) (eIF-2C 4) (eIF2C 4)
		O75027	ABCB7	ATP-binding cassette sub-family B member 7, mitochondrial (ATP-binding cassette transporter 7) (ABC transporter 7 protein)
		P28288	ABCD3	ATP-binding cassette sub-family D member 3 (70 kDa peroxisomal membrane protein) (PMP70)

		Q04771	ACVR1	Activin receptor type-1 (EC 2.7.11.30) (Activin receptor type I) (ACTR-I) (Activin receptor-like kinase 2) (ALK-2) (Serine/threonine-protein kinase receptor R1) (SKR1) (TGF-B superfamily receptor type I) (TSR-I)
		Q9UNA0	ADAMTS5	A disintegrin and metalloproteinase with thrombospondin motifs 5 (ADAM-TS 5) (ADAM-TS5) (ADAMTS-5) (EC 3.4.24.-) (A disintegrin and metalloproteinase with thrombospondin motifs 11) (ADAM-TS 11) (ADAMTS-11) (ADMP-2) (Aggrecanase-2)
		O60242	ADGRB3	Adhesion G protein-coupled receptor B3 (Brain-specific angiogenesis inhibitor 3)
		P52594	AGFG1	Arf-GAP domain and FG repeat-containing protein 1 (HIV-1 Rev-binding protein) (Nucleoporin-like protein RIP) (Rev-interacting protein) (Rev/Rex activation domain-binding protein)
		Q5TGY3	AHDC1	AT-hook DNA-binding motif-containing protein 1
		P53367	ARFIP1	Arfaptin-1 (ADP-ribosylation factor-interacting protein 1)
		Q9NVJ2	ARL8B	ADP-ribosylation factor-like protein 8B (ADP-ribosylation factor-like protein 10C) (Novel small G protein indispensable for equal chromosome segregation 1)
		Q96B67	ARRDC3	Arrestin domain-containing protein 3 (TBP-2-like inducible membrane protein) (TLIMP)
		P23634	ATP2B4	Plasma membrane calcium-transporting ATPase 4 (PMCA4) (EC 7.2.2.10) (Matrix-remodeling-associated protein 1) (Plasma membrane calcium ATPase isoform 4) (Plasma membrane calcium pump isoform 4)

		O75787	ATP6AP2	Renin receptor (ATPase H(+)-transporting lysosomal accessory protein 2) (ATPase H(+)-transporting lysosomal-interacting protein 2) (ER-localized type I transmembrane adaptor) (Embryonic liver differentiation factor 10) (N14F) (Renin/prorenin receptor) (Vacuolar ATP synthase membrane sector-associated protein M8-9) (ATP6M8-9) (V-ATPase M8.9 subunit) [Cleaved into: Renin receptor N-terminal fragment; Renin receptor C-terminal fragment]
		O43286	B4GALT5	Beta-1,4-galactosyltransferase 5 (Beta-1,4-GalTase 5) (Beta4Gal-T5) (b4Gal-T5) (EC 2.4.1.-) (Beta-1,4-GalT II) (Glucosylceramide beta-1,4-galactosyltransferase) (EC 2.4.1.274) (Lactosylceramide synthase) (LacCer synthase) (UDP-Gal:beta-GlcNAc beta-1,4-galactosyltransferase 5) (UDP-galactose:beta-N-acetylglucosamine beta-1,4-galactosyltransferase 5)
		Q9UBX8	B4GALT6	Beta-1,4-galactosyltransferase 6 (Beta-1,4-GalTase 6) (Beta4Gal-T6) (b4Gal-T6) (EC 2.4.1.-) (Glucosylceramide beta-1,4-galactosyltransferase) (EC 2.4.1.274) (Lactosylceramide synthase) (LacCer synthase) (UDP-Gal:beta-GlcNAc beta-1,4-galactosyltransferase 6) (UDP-Gal:glucosylceramide beta-1,4-galactosyltransferase) (UDP-galactose:beta-N-acetylglucosamine beta-1,4-galactosyltransferase 6)
		Q9NY43	BARHL2	BarH-like 2 homeobox protein
		Q9C0J9	BHLHE41	Protein BEX3 (Brain-expressed X-linked protein 3) (Nerve growth factor receptor-associated protein 1) (Ovarian granulosa cell 13.0 kDa protein HGR74) (p75NTR-associated cell death executor)
		Q9H694	BICC1	Class E basic helix-loop-helix protein 41 (bHLHe41) (Class B basic helix-loop-helix protein 3) (bHLHb3) (Differentially expressed in chondrocytes protein 2) (hDEC2) (Enhancer-of-split and hairy-related protein 1) (SHARP-1)

		O14981	BTAF1	Protein bicaudal C homolog 1 (Bic-C)
		Q9Y2F9	BTBD3	TATA-binding protein-associated factor 172 (EC 3.6.4.-) (ATP-dependent helicase BTAF1) (B-TFIID transcription factor-associated 170 kDa subunit) (TAF(II)170) (TBP-associated factor 172) (TAF-172)
**miRDB**		Q9UL18	AGO1	argonaute RISC catalytic component 1
		Q9HCK5	AGO4	argonaute RISC catalytic component 4
		Q9UNA3	A4GNT	alpha-1,4-N-acetylglucosaminyltransferase
		O95477	ABCA1	ATP binding cassette subfamily A member 1
		O75027	ABCB7	ATP binding cassette subfamily B member 7
		P28288	ABCD3	ATP binding cassette subfamily D member 3
		Q04771	ACVR1	activin A receptor type 1
		O14672	ADAM10	ADAM metallopeptidase domain 10
		P78536	ADAM17	ADAM metallopeptidase domain 17
		Q9P0K1	ADAM22	ADAM metallopeptidase domain 22
		O75077	ADAM23	ADAM metallopeptidase domain 23
		Q8TE58	ADAMTS15	ADAM metallopeptidase with thrombospondin type 1 motif 15
		Q8TE59	ADAMTS19	ADAM metallopeptidase with thrombospondin type 1 motif 19
		Q08462	ADCY2	adenylate cyclase 2
		O60242	ADGRB3	adhesion G protein-coupled receptor B3
		Q8IZF2	ADGRF5	adhesion G protein-coupled receptor F5
		P52594	AGFG1	ArfGAP with FG repeats 1
		Q5TGY3	AHDC1	AT-hook DNA binding motif containing 1
		Q92667	AKAP1	A-kinase anchoring protein 1
		Q13740	ALCAM	activated leukocyte cell adhesion molecule
		Q5VTE6	ANGEL2	angel homolog 2
		P09525	ANXA4	annexin A4
		Q9UPM8	AP4E1	adaptor related protein complex 4 subunit epsilon 1
		P18085	ARF4	ADP ribosylation factor 4

		P53367	ARFIP1	ADP ribosylation factor interacting protein 1
		Q5T5U3	ARHGAP21	Rho GTPase activating protein 21
		Q9NZN5	ARHGEF12	Rho guanine nucleotide exchange factor 12
		Q15041	ARL6IP1	ADP ribosylation factor like GTPase 6 interacting protein 1
		Q9NVJ2	ARL8B	ADP ribosylation factor like GTPase 8B
		Q96B67	ARRDC3	arrestin domain containing 3
		Q6ZNE5	ATG14	autophagy related 14
		P98196	ATP11A	ATPase phospholipid transporting 11A
		P23634	ATP2B4	ATPase plasma membrane Ca2+ transporting 4
		P51164	ATP4B	ATPase H+/K+ transporting subunit beta
		O75787	ATP6AP2	ATPase H+ transporting accessory protein 2
		Q9Y2Q0	ATP8A1	ATPase phospholipid transporting 8A1
		P54253	ATXN1	ataxin 1
		Q9ULK2	ATXN7L1	ataxin 7 like 1
		O43286	B4GALT5	beta-1,4-galactosyltransferase 5
		Q9UBX8	B4GALT6	beta-1,4-galactosyltransferase 6
		Q9BYV9	BACH2	BTB domain and CNC homolog 2
		Q9UIF8	BAZ2B	bromodomain adjacent to zinc finger domain 2B
		O43521	BCL2L11	BCL2 like 11
		Q9H694	BICC1	BicC family RNA binding protein 1
		P12645	BMP3	bone morphogenetic protein 3
		P55201	BRPF1	bromodomain and PHD finger containing 1
		O14981	BTAF1	B-TFIID TATA-box binding protein associated factor 1
		Q9BSF8	BTBD10	BTB domain containing 10
		Q9Y2F9	BTBD3	BTB domain containing 3
		Q96B23	C18orf25	chromosome 18 open reading frame 25
		Q9NS00	C1GALT1	core 1 synthase, glycoprotein-N-acetylgalactosamine 3-beta-galactosyltransferase 1
		Q96GV9	C5orf30	chromosome 5 open reading frame 30

		Q86V35	CABP7	calcium binding protein 7
		Q9BY67	CADM1	cell adhesion molecule 1
		Q08AD1	CAMSAP2	calmodulin regulated spectrin associated protein family member 2
		Q86VP6	CAND1	cullin associated and neddylation dissociated 1
		P27824	CANX	calnexin
		Q86V15	CASZ1	castor zinc finger 1
		Q13191	CBLB	Cbl proto-oncogene B
		Q75N03	CBLL1	Cbl proto-oncogene like 1
		Q6ZP82	CCDC141	coiled-coil domain containing 141
		Q16204	CCDC6	coiled-coil domain containing 6
		P32239	CCKBR	cholecystokinin B receptor
		P40227	CCT6A	chaperonin containing TCP1 subunit 6A
		P21854	CD72	CD72 molecule
		Q9UNH5	CDC14A	cell division cycle 14A
		Q9HBT6	CDH20	cadherin 20
		Q9BWU1	CDK19	cyclin dependent kinase 19
		Q15078	CDK5R1	cyclin dependent kinase 5 regulatory subunit 1
		O76039	CDKL5	cyclin dependent kinase like 5
		P46527	CDKN1B	cyclin dependent kinase inhibitor 1B
		Q92903	CDS1	CDP-diacylglycerol synthase 1
		Q9NYQ6	CELSR1	cadherin EGF LAG seven-pass G-type receptor 1
		Q53EZ4	CEP55	centrosomal protein 55
		Q6ZMG9	CERS6	ceramide synthase 6
		Q9Y281	CFL2	cofilin 2
		Q9P2D1	CHD7	chromodomain helicase DNA binding protein 7
		O15111	CHUK	conserved helix-loop-helix ubiquitous kinase
		P33076	CIITA	class II major histocompatibility complex transactivator
		P51797	CLCN6	chloride voltage-gated channel 6

		P09496	CLTA	clathrin light chain A
		Q8IWV2	CNTN4	contactin 4
		Q96NU0	CNTNAP3B	contactin associated protein like 3B
		P02462	COL4A1	collagen type IV alpha 1 chain
		O75390	CS	citrate synthase
		P09603	CSF1	colony stimulating factor 1
		P10619	CTSA	cathepsin A
		Q93034	CUL5	cullin 5
		Q7L1T6	CYB5R4	cytochrome b5 reductase 4
		O43739	CYTH3	cytohesin 3
		Q8IU60	DCP2	decapping mRNA 2
		P26196	DDX6	DEAD-box helicase 6
		Q5VZ89	DENND4C	DENN domain containing 4C
		Q9UPY3	DICER1	dicer 1, ribonuclease III
		Q15700	DLG2	discs large MAGUK scaffold protein 2
		Q9Y485	DMXL1	Dmx like 1
		P26358	DNMT1	DNA methyltransferase 1
		Q96HP0	DOCK6	dedicator of cytokinesis 6
		P28562	DUSP1	dual specificity phosphatase 1
		Q92630	DYRK2	dual specificity tyrosine phosphorylation regulated kinase 2
		Q96AV8	E2F7	E2F transcription factor 7
		P52799	EFNB2	ephrin B2
		Q12926	ELAVL2	ELAV like RNA binding protein 2
		P26378	ELAVL4	ELAV like RNA binding protein 4
		Q5NDL2	EOGT	EGF domain specific O-linked N-acetylglucosamine transferase
		Q99814	EPAS1	endothelial PAS domain protein 1
		O95278	EPM2A	EPM2A, laforin glucan phosphatase
		O95208	EPN2	epsin 2
		P42566	EPS15	epidermal growth factor receptor pathway substrate 15

		P21860	ERBB3	erb-b2 receptor tyrosine kinase 3
		Q9UJM3	ERRFI1	ERBB receptor feedback inhibitor 1
		P03372	ESR1	estrogen receptor 1
		P62508	ESRRG	estrogen related receptor gamma
		Q99504	EYA3	EYA transcriptional coactivator and phosphatase 3
		P13726	F3	coagulation factor III, tissue factor
		Q969W3	FAM104A	family with sequence similarity 104 member A
		Q3B820	FAM161A	FAM161A, centrosomal protein
		A1KXE4	FAM168B	family with sequence similarity 168 member B
		Q9H0X4	FAM234A	family with sequence similarity 234 member A
		Q8N2R8	FAM43A	family with sequence similarity 43 member A
		P35555	FBN1	fibrillin 1
		Q9UHY8	FEZ2	fasciculation and elongation protein zeta 2
		Q06787	FMR1	fragile X mental retardation 1
		Q12946	FOXF1	forkhead box F1
		P51114	FXR1	FMR1 autosomal homolog 1
		P24522	GADD45A	growth arrest and DNA damage inducible alpha
		P17677	GAP43	growth associated protein 43
		Q86SX6	GLRX5	glutaredoxin 5
		P60983	GMFB	glia maturation factor beta
		Q9UKJ3	GPATCH8	G-patch domain containing 8
		Q9NPB8	GPCPD1	glycerophosphocholine phosphodiesterase 1
		P51674	GPM6A	glycoprotein M6A
		Q8N3F9	GPR137C	G protein-coupled receptor 137C
		Q8NFJ5	GPRC5A	G protein-coupled receptor class C group 5 member A
		O43424	GRID2	glutamate ionotropic receptor delta type subunit 2
		P32780	GTF2H1	general transcription factor IIH subunit 1
		O75367	H2AFY	H2A histone family member Y
		Q9Y450	HBS1L	HBS1 like translational GTPase

		Q9P2P5	HECW2	HECT, C2 and WW domain containing E3 ubiquitin protein ligase 2
		Q86YM7	HOMER1	homer scaffold protein 1
		P31273	HOXC8	homeobox C8
		P24593	IGFBP5	insulin like growth factor binding protein 5
		P40933	IL15	interleukin 15
		Q9H160	ING2	inhibitor of growth family member 2
		P09529	INHBB	inhibin subunit beta B
		Q9ULG1	INO80	INO80 complex subunit
		B1AKI9	ISM1	isthmin 1
		P08648	ITGA5	integrin subunit alpha 5
		Q13797	ITGA9	integrin subunit alpha 9
		P26012	ITGB8	integrin subunit beta 8
		Q13572	ITPK1	inositol-tetrakisphosphate 1-kinase
		Q9NZM3	ITSN2	intersectin 2
		Q92833	JARID2	jumonji and AT-rich interaction domain containing 2
		Q8N9B5	JMY	junction mediating and regulatory protein, p53 cofactor
		O95251	KAT7	lysine acetyltransferase 7
		Q68DU8	KCTD16	potassium channel tetramerization domain containing 16
		Q6ZMT4	KDM7A	lysine demethylase 7A
		Q92628	KIAA0232	KIAA0232
		Q5T5P2	KIAA1217	KIAA1217
		Q15058	KIF14	kinesin family member 14
		O43474	KLF4	Kruppel like factor 4
		Q13887	KLF5	Kruppel like factor 5
		Q99612	KLF6	Kruppel like factor 6
		Q03164	KMT2A	lysine methyltransferase 2A
		Q01546	KRT76	keratin 76
		Q14739	LBR	lamin B receptor

		P01130	LDLR	low density lipoprotein receptor
		O95214	LEPROTL1	leptin receptor overlapping transcript like 1
		P38571	LIPA	lipase A, lysosomal acid type
		Q8IWU2	LMTK2	lemur tyrosine kinase 2
		P98164	LRP2	LDL receptor related protein 2
		Q15345	LRRC41	leucine rich repeat containing 41
		Q14766	LTBP1	latent transforming growth factor beta binding protein 1
		Q8IV50	LYSMD2	LysM domain containing 2
		Q9Y5Q3	MAFB	MAF bZIP transcription factor B
		O15525	MAFG	MAF bZIP transcription factor G
		Q02750	MAP2K1	mitogen-activated protein kinase kinase 1
		Q9Y6R4	MAP3K4	mitogen-activated protein kinase kinase kinase 4
		P80192	MAP3K9	mitogen-activated protein kinase kinase kinase 9
		Q9P1T7	MDFIC	MyoD family inhibitor domain containing
		Q86YW9	MED12L	mediator complex subunit 12 like
		P50222	MEOX2	mesenchyme homeobox 2
		Q8N108	MIER1	MIER1 transcriptional regulator
		O75030	MITF	melanocyte inducing transcription factor
		P55197	MLLT10	MLLT10, histone lysine methyltransferase DOT1L cofactor
		P55198	MLLT6	MLLT6, PHD finger containing
		Q15546	MMD	monocyte to macrophage differentiation associated
		P09238	MMP10	matrix metallopeptidase 10
		P45452	MMP13	matrix metallopeptidase 13
		P51511	MMP15	matrix metallopeptidase 15
		Q99583	MNT	MAX network transcriptional repressor
		Q9UJG1	MOSPD1	motile sperm domain containing 1
		P40238	MPL	MPL proto-oncogene, thrombopoietin receptor
		O14807	MRAS	muscle RAS oncogene homolog
		Q96LB0	MRGPRX3	MAS related GPR family member X3

		Q9Y4B5	MTCL1	microtubule crosslinking factor 1
		Q9NXD2	MTMR10	myotubularin related protein 10
		Q8NCE2	MTMR14	myotubularin related protein 14
		Q96QG7	MTMR9	myotubularin related protein 9
		Q05195	MXD1	MAX dimerization protein 1
		Q8WUY8	NAT14	N-acetyltransferase 14 (putative)
		Q9NZQ3	NCKIPSD	NCK interacting protein with SH3 domain
		Q15788	NCOA1	nuclear receptor coactivator 1
		Q96JN8	NEURL4	neuralized E3 ubiquitin protein ligase 4
		O94916	NFAT5	nuclear factor of activated T cells 5
		Q6T4R5	NHS	NHS actin remodeling regulator
		Q9Y5B8	NME7	NME/NM23 family member 7
		Q13253	NOG	noggin
		O94818	NOL4	nucleolar protein 4
		Q96MY1	NOL4L	nucleolar protein 4 like
		Q8NDH3	NPEPL1	aminopeptidase like 1
		Q9Y639	NPTN	neuroplastin
		Q15818	NPTX1	neuronal pentraxin 1
		Q86WQ0	NR2C2AP	nuclear receptor 2C2 associated protein
		Q7Z6K4	NRARP	NOTCH regulated ankyrin repeat protein
		O14786	NRP1	neuropilin 1
		Q9H0P0	NT5C3A	5'-nucleotidase, cytosolic IIIA
		Q9BXB4	OSBPL11	oxysterol binding protein like 11
		Q01804	OTUD4	OTU deubiquitinase 4
		Q14123	PDE1C	phosphodiesterase 1C
		Q9H792	PEAK1	pseudopodium enriched atypical kinase 1
		O15173	PGRMC2	progesterone receptor membrane component 2
		O75167	PHACTR2	phosphatase and actin regulator 2
		Q9BVI0	PHF20	PHD finger protein 20

		Q92576	PHF3	PHD finger protein 3
		P37287	PIGA	phosphatidylinositol glycan anchor biosynthesis class A
		Q96S52	PIGS	phosphatidylinositol glycan anchor biosynthesis class S
		O00443	PIK3C2A	phosphatidylinositol-4-phosphate 3-kinase catalytic subunit type 2 alpha
		Q9BZ72	PITPNM2	phosphatidylinositol transfer protein membrane associated 2
		Q9Y263	PLAA	phospholipase A2 activating protein
		Q8IY17	PNPLA6	patatin like phospholipase domain containing 6
		P20265	POU3F2	POU class 3 homeobox 2
		Q96QC0	PPP1R10	protein phosphatase 1 regulatory subunit 10
		Q96SB3	PPP1R9B	protein phosphatase 1 regulatory subunit 9B
		Q9UPN7	PPP6R1	protein phosphatase 6 regulatory subunit 1
		Q7Z3G6	PRICKLE2	prickle planar cell polarity protein 2
		Q13131	PRKAA1	protein kinase AMP-activated catalytic subunit alpha 1
		Q9UGJ0	PRKAG2	protein kinase AMP-activated non-catalytic subunit gamma 2
		Q05513	PRKCZ	protein kinase C zeta
		P04156	PRNP	prion protein
		Q15185	PTGES3	prostaglandin E synthase 3
		Q96PU8	QKI	QKI, KH domain containing RNA binding
		P61106	RAB14	RAB14, member RAS oncogene family
		P0DI83	RAB34	RAB34, member RAS oncogene family
		Q15311	RALBP1	ralA binding protein 1
		P46060	RANGAP1	Ran GTPase activating protein 1
		Q8NHQ8	RASSF8	Ras association domain family member 8
		Q9BX46	RBM24	RNA binding motif protein 24
		Q5TC82	RC3H1	ring finger and CCCH-type domains 1
		Q2KHR2	RFX7	regulatory factor X7
		Q96B86	RGMA	repulsive guidance molecule BMP co-receptor a
		Q6R327	RICTOR	RPTOR independent companion of MTOR complex 2

		Q9H871	RMND5A	required for meiotic nuclear division 5 homolog A
		Q5W0B1	RNF219	ring finger protein 219
		Q9H0F5	RNF38	ring finger protein 38
		Q9Y6N7	ROBO1	roundabout guidance receptor 1
		Q9HCK4	ROBO2	roundabout guidance receptor 2
		Q13464	ROCK1	Rho associated coiled-coil containing protein kinase 1
		O75582	RPS6KA5	ribosomal protein S6 kinase A5
		Q9NQC3	RTN4	reticulon 4
		Q13950	RUNX2	runt related transcription factor 2
		P21453	S1PR1	sphingosine-1-phosphate receptor 1
		Q86WG5	SBF2	SET binding factor 2
		Q15858	SCN9A	sodium voltage-gated channel alpha subunit 9
		Q93073	SECISBP2L	SECIS binding protein 2 like
		P58004	SESN2	sestrin 2
		P58005	SESN3	sestrin 3
		Q86VW0	SESTD1	SEC14 and spectrin domain containing 1
		Q16585	SGCB	sarcoglycan beta
		P57059	SIK1	salt inducible kinase 1
		A0A0B4J2F2	SIK1B	salt inducible kinase 1B (putative)
		Q9NRC8	SIRT7	sirtuin 7
		Q9UIU6	SIX4	SIX homeobox 4
		Q1XH10	SKIDA1	SKI/DACH domain containing 1
		O15403	SLC16A6	solute carrier family 16 member 6
		Q9HC58	SLC24A3	solute carrier family 24 member 3
		Q96H78	SLC25A44	solute carrier family 25 member 44
		O43511	SLC26A4	solute carrier family 26 member 4
		P11166	SLC2A1	solute carrier family 2 member 1
		P52788	SMS	spermine synthase
		O60641	SNAP91	synaptosome associated protein 91

		O75324	SNN	stannin
		Q96L92	SNX27	sorting nexin family member 27
		Q07890	SOS2	SOS Ras/Rho guanine nucleotide exchange factor 2
		Q68D10	SPTY2D1	SPT2 chromatin protein domain containing 1
		Q05519	SRSF11	serine and arginine rich splicing factor 11
		P43307	SSR1	signal sequence receptor subunit 1
		Q9Y3M8	STARD13	StAR related lipid transfer domain containing 13
		Q9P2F5	STOX2	storkhead box 2
		O43426	SYNJ1	synaptojanin 1
		Q7Z422	SZRD1	SUZ RNA binding domain containing 1
		Q9C0D5	TANC1	tetratricopeptide repeat, ankyrin repeat and coiled-coil containing 1
		O95759	TBC1D8	TBC1 domain family member 8
		Q9BZK7	TBL1XR1	transducin beta like 1 X-linked receptor 1
		Q02763	TEK	TEK receptor tyrosine kinase
		Q6PIY7	TENT2	terminal nucleotidyltransferase 2
		P02786	TFRC	transferrin receptor
		P01135	TGFA	transforming growth factor alpha
		P61812	TGFB2	transforming growth factor beta 2
		Q9GZN2	TGIF2	TGFB induced factor homeobox 2
		O43493	TGOLN2	trans-golgi network protein 2
		Q9Y3B3	TMED7	transmembrane p24 trafficking protein 7
		Q8WVE7	TMEM170A	transmembrane protein 170A
		Q2M3C6	TMEM266	transmembrane protein 266
		Q969K7	TMEM54	transmembrane protein 54
		Q5T3F8	TMEM63B	transmembrane protein 63B
		Q9NQ34	TMEM9B	TMEM9 domain family member B
		Q8NDV7	TNRC6A	trinucleotide repeat containing 6A
		Q9HCJ0	TNRC6C	trinucleotide repeat containing 6C

		O94826	TOMM70	translocase of outer mitochondrial membrane 70
		O60296	TRAK2	trafficking kinesin protein 2
		Q8IWR1	TRIM59	tripartite motif containing 59
		Q9H3M7	TXNIP	thioredoxin interacting protein
		Q14157	UBAP2L	ubiquitin associated protein 2 like
		P61077	UBE2D3	ubiquitin conjugating enzyme E2 D3
		O95155	UBE4B	ubiquitination factor E4B
		P55916	UCP3	uncoupling protein 3
		Q8TAS1	UHMK1	U2AF homology motif kinase 1
		Q70CQ4	USP31	ubiquitin specific peptidase 31
		Q8NFA0	USP32	ubiquitin specific peptidase 32
		Q8TEY7	USP33	ubiquitin specific peptidase 33
		Q86UV5	USP48	ubiquitin specific peptidase 48
		Q96GC9	VMP1	vacuole membrane protein 1
		Q8NEZ2	VPS37A	VPS37A, ESCRT-I subunit
		Q86XK7	VSIG1	V-set and immunoglobulin domain containing 1
		Q6ZS81	WDFY4	WDFY family member 4
		Q8TBZ3	WDR20	WD repeat domain 20
		O94967	WDR47	WD repeat domain 47
		P04628	WNT1	Wnt family member 1
		O00744	WNT10B	Wnt family member 10B
		Q9C0E2	XPO4	exportin 4
		Q9H6S0	YTHDC2	YTH domain containing 2
		P31946	YWHAB	tyrosine 3-monooxygenase/tryptophan 5-monooxygenase activation protein beta
		Q99592	ZBTB18	zinc finger and BTB domain containing 18
		Q9C0B9	ZCCHC2	zinc finger CCHC-type containing 2
		Q8IUH5	ZDHHC17	zinc finger DHHC-type containing 17
		Q8IYP9	ZDHHC23	zinc finger DHHC-type containing 23

		Q9NXF8	ZDHHC7	zinc finger DHHC-type containing 7
		Q68DK2	ZFYVE26	zinc finger FYVE-type containing 26
		Q7Z570	ZNF804A	zinc finger protein 804A
		O75541	ZNF821	zinc finger protein 821
		Q8ND25	ZNRF1	zinc and ring finger 1
**TargetScan**	**miR-193-3p Upregulated 24 hours**	Q8IZ07	ANKRD13A	Ankyrin repeat domain-containing protein 13A (Protein KE03)
		Q96CW1	AP2M1	AP-2 complex subunit mu (AP-2 mu chain) (Adaptin-mu2) (Adaptor protein complex AP-2 subunit mu) (Adaptor-related protein complex 2 subunit mu) (Clathrin assembly protein complex 2 mu medium chain) (Clathrin coat assembly protein AP50) (Clathrin coat-associated protein AP50) (HA2 50 kDa subunit) (Plasma membrane adaptor AP-2 50 kDa protein)
		Q14CB8	ARHGAP19	Rho GTPase-activating protein 19 (Rho-type GTPase-activating protein 19)
		Q9NZN5	ARHGEF12	Rho guanine nucleotide exchange factor 12 (Leukemia-associated RhoGEF)
		P0DP23	CALM1	Calmodulin-1
		O95931	CBX7	Chromobox protein homolog 7
		P24385	CCND1	G1/S-specific cyclin-D1
		O94985	CLSTN1	Calsyntenin-1
		P62633	CNBP	CCHC-type zinc finger nucleic acid binding protein
		Q9ULM6	CNOT6	CCR4-NOT transcription complex subunit 6
		Q05D32	CTDSPL2	CTD small phosphatase-like protein 2
		Q15303	ERBB4	Receptor tyrosine-protein kinase erbB-4
		Q96KN4	FAM84A	Protein LRATD1
		Q9C0D6	FHDC1	FH2 domain-containing protein 1 (Inverted formin-1)
		Q01543	FLI1	Friend leukemia integration 1 transcription factor (Proto-oncogene Fli-1) (Transcription factor ERGB)

		P42694	HELZ	Probable helicase with zinc finger domain (EC 3.6.4.-) (Down-regulated in human cancers protein)
		P35453	HOXD13	Homeobox protein Hox-D13 (Homeobox protein Hox-4I)
		Q8NFM7	IL17RD	Interleukin-17 receptor D (IL-17 receptor D) (IL-17RD) (IL17Rhom) (Interleukin-17 receptor-like protein) (Sef homolog) (hSef)
		Q8WYH8	ING5	Inhibitor of growth protein 5
		P63252	KCNJ2	Inward rectifier potassium channel 2
		P01116	KRAS	GTPase KRas
		Q07820	MCL1	Induced myeloid leukemia cell differentiation protein Mcl-1 (Bcl-2-like protein 3) (Bcl2-L-3) (Bcl-2-related protein EAT/mcl1) (mcl1/EAT)
		P51513	NOVA1	RNA-binding protein Nova-1
		P46459	NSF	Vesicle-fusing ATPase
		O75914	PAK3	Serine/threonine-protein kinase PAK 3
		O96013	PAK4	Serine/threonine-protein kinase PAK 4 (EC 2.7.11.1) (p21-activated kinase 4) (PAK-4)
		Q6DJT9	PLAG1	Zinc finger protein PLAG1
		O60486	PLXNC1	Plexin-C1
		Q13362	PPP2R5C	Serine/threonine-protein phosphatase 2A 56 kDa regulatory subunit gamma isoform
		Q06455	RUNX1T1	Protein CBFA2T1
		Q3KNW5	SLC10A6	Solute carrier family 10 member 6
		Q9UGH3	SLC23A2	Solute carrier family 23 member 2
		Q01130	SRSF2	Serine/arginine-rich splicing factor 2
		Q9BVH7	ST6GALNAC5	Alpha-N-acetylgalactosaminide alpha-2,6-sialyltransferase
		P15884	TCF4	Transcription factor 7-like 2 (HMG box transcription factor 4) (T-cell-specific transcription factor 4) (T-cell factor 4) (TCF-4) (hTCF-4)

		Q9NZI7	UBP1	Upstream-binding protein 1 (Transcription factor LBP-1)
		Q6UXN9	WDR82	WD repeat-containing protein 82 (Protein TMEM113) (Swd2)
**miRDB**		Q2M2I8	AAK1	AP2 associated kinase 1
		Q9NUT2	ABCB8	ATP binding cassette subfamily B member 8
		Q9NYB9	ABI2	abl interactor 2
		P78563	ADARB1	adenosine deaminase, RNA specific B1
		P48960	ADGRE5	adhesion G protein-coupled receptor E5
		Q13155	AIMP2	aminoacyl tRNA synthetase complex interacting multifunctional protein 2
		Q96IF1	AJUBA	ajuba LIM protein
		Q96CW1	AP2M1	adaptor related protein complex 2 subunit mu 1
		O15033	AREL1	apoptosis resistant E3 ubiquitin protein ligase 1
		Q9NZN5	ARHGEF12	Rho guanine nucleotide exchange factor 12
		Q5U623	ATF7IP2	activating transcription factor 7 interacting protein 2
		Q9BY67	CADM1	cell adhesion molecule 1
		Q8N187	CARF	calcium responsive transcription factor
		O95931	CBX7	chromobox 7
		Q8IWP9	CCDC28A	coiled-coil domain containing 28A
		P24385	CCND1	cyclin D1
		O15516	CLOCK	clock circadian regulator
		Q00610	CLTC	clathrin heavy chain
		Q9ULM6	CNOT6	CCR4-NOT transcription complex subunit 6
		Q8IUR6	CREBRF	CREB3 regulatory factor
		Q9BWK5	CYREN	cell cycle regulator of NHEJ
		P61962	DCAF7	DDB1 and CUL4 associated factor 7
		Q8TDJ6	DMXL2	Dmx like 2
		Q8WXU2	DNAAF4	dynein axonemal assembly factor 4
		Q5JPH6	EARS2	glutamyl-tRNA synthetase 2, mitochondrial
		P54756	EPHA5	EPH receptor A5

		Q15303	ERBB4	erb-b2 receptor tyrosine kinase 4
		P14921	ETS1	ETS proto-oncogene 1, transcription factor
		P50549	ETV1	ETS variant 1
		Q6V0I7	FAT4	FAT atypical cadherin 4
		Q9C0D6	FHDC1	FH2 domain containing 1
		Q01543	FLI1	Fli-1 proto-oncogene, ETS transcription factor
		Q99678	GPR20	G protein-coupled receptor 20
		Q9ULI3	HEG1	heart development protein with EGF like domains 1
		Q1KMD3	HNRNPUL2	heterogeneous nuclear ribonucleoprotein U like 2
		P35453	HOXD13	homeobox D13
		P24593	IGFBP5	insulin like growth factor binding protein 5
		Q8NFM7	IL17RD	interleukin 17 receptor D
		Q53TQ3	INO80D	INO80 complex subunit D
		P63252	KCNJ2	potassium voltage-gated channel subfamily J member 2
		P10721	KIT	KIT proto-oncogene receptor tyrosine kinase
		P01116	KRAS	KRAS proto-oncogene, GTPase
		P11047	LAMC1	laminin subunit gamma 1
		Q13753	LAMC2	laminin subunit gamma 2
		O75096	LRP4	LDL receptor related protein 4
		Q9NU23	LYRM2	LYR motif containing 2
		P53779	MAPK10	mitogen-activated protein kinase 10
		Q9Y4F3	MARF1	meiosis regulator and mRNA stability factor 1
		Q6P1R3	MSANTD2	Myb/SANT DNA binding domain containing 2
		Q8IVL0	NAV3	neuron navigator 3
		P35240	NF2	neurofibromin 2
		P21589	NT5E	5'-nucleotidase ecto
		Q99650	OSMR	oncostatin M receptor
		Q93086	P2RX5	purinergic receptor P2X 5
		P37287	PIGA	phosphatidylinositol glycan anchor biosynthesis class A

		Q6DJT9	PLAG1	PLAG1 zinc finger
		P00749	PLAU	plasminogen activator, urokinase
		Q4KWH8	PLCH1	phospholipase C eta 1
		O60486	PLXNC1	plexin C1
		Q8TEU7	RAPGEF6	Rap guanine nucleotide exchange factor 6
		Q96E39	RBMXL1	RBMX like 1
		Q96B86	RGMA	repulsive guidance molecule BMP co-receptor a
		P62070	RRAS2	RAS related 2
		Q06455	RUNX1T1	RUNX1 translocation partner 1
		Q8IUQ4	SIAH1	siah E3 ubiquitin protein ligase 1
		Q3KNW5	SLC10A6	solute carrier family 10 member 6
		O15403	SLC16A6	solute carrier family 16 member 6
		Q07890	SOS2	SOS Ras/Rho guanine nucleotide exchange factor 2
		Q01130	SRSF2	serine and arginine rich splicing factor 2
		Q9BVH7	ST6GALNAC5	ST6 N-acetylgalactosaminide alpha-2,6-sialyltransferase 5
		Q7L7X3	TAOK1	TAO kinase 1
		Q6NXT6	TAPT1	transmembrane anterior posterior transformation 1
		Q03167	TGFBR3	transforming growth factor beta receptor 3
		Q9Y5J9	TIMM8B	translocase of inner mitochondrial membrane 8 homolog B
		Q92574	TSC1	TSC complex subunit 1
		Q9NZI7	UBP1	upstream binding protein 1
		Q96BW1	UPRT	uracil phosphoribosyltransferase homolog
		Q70EK8	USP53	ubiquitin specific peptidase 53
		Q9GZP7	VN1R1	vomeronasal 1 receptor 1
		Q6UXN9	WDR82	WD repeat domain 82
		P63104	YWHAZ	tyrosine 3-monooxygenase/tryptophan 5-monooxygenase activation protein zeta
		P24278	ZBTB25	zinc finger and BTB domain containing 25
		Q9HA38	ZMAT3	zinc finger matrin-type 3

		Q5T7W0	ZNF618	zinc finger protein 618
**TargetScan**	**miR-194-5p Downregulated 24 hours**	Q9Y281	CFL2	Centromere protein L (CENP-L) (Interphase centromere complex protein 33)
		Q8TD26	CHD6	Chromodomain-helicase-DNA-binding protein 6 (CHD-6) (EC 3.6.4.12) (ATP-dependent helicase CHD6) (Radiation-induced gene B protein)
		Q52LR7	EPC2	Enhancer of polycomb homolog 2
		Q53TX7	FLJ20373	Uncharacterized protein FLJ20373
		Q8NBR6	MINDY2	Ubiquitin carboxyl-terminal hydrolase MINDY-2 (EC 3.4.19.12) (Deubiquitinating enzyme MINDY-2) (Protein FAM63B)
		Q9Y266	NUDC	Nuclear migration protein nudC (Nuclear distribution protein C homolog)
		Q9ULI1	NWD2	NACHT and WD repeat domain-containing protein 2 (Leucine-rich repeat and WD repeat-containing protein KIAA1239)
		Q9BPZ3	PAIP2	Polyadenylate-binding protein-interacting protein 2
		P10644	PRKAR1A	cAMP-dependent protein kinase type II-alpha regulatory subunit
		Q15382	RHEB	GTP-binding protein Rheb
		P61956	SUMO2	SUZ domain-containing protein 1 (Putative MAPK-activating protein PM18/PM20/PM22)
**miRDB**		Q9NRK6	ABCB10	ATP binding cassette subfamily B member 10
		Q9H3P7	ACBD3	acyl-CoA binding domain containing 3
		P22303	ACHE	acetylcholinesterase (Cartwright blood group)
		Q13705	ACVR2B	activin A receptor type 2B
		O95841	ANGPTL1	angiopoietin like 1
		Q5T5U3	ARHGAP21	Rho GTPase activating protein 21
		Q68CP9	ARID2	AT-rich interaction domain 2
		P98194	ATP2C1	ATPase secretory pathway Ca2+ transporting 1
		Q9UI12	ATP6V1H	ATPase H+ transporting V1 subunit H

		P12694	BCKDHA	branched chain keto acid dehydrogenase E1, alpha polypeptide
		Q8TD16	BICD2	BICD cargo adaptor 2
		Q12982	BNIP2	BCL2 interacting protein 2
		Q5PSV4	BRMS1L	BRMS1 like transcriptional repressor
		Q9P203	BTBD7	BTB domain containing 7
		Q96B23	C18orf25	chromosome 18 open reading frame 25
		Q8TCD1	C18orf32	chromosome 18 open reading frame 32
		Q9NYK6	C21orf91	chromosome 21 open reading frame 91
		Q6ZTY9	C7orf65	chromosome 7 open reading frame 65
		Q9BY67	CADM1	cell adhesion molecule 1
		Q9BXY5	CAPS2	calcyphosine 2
		O14936	CASK	calcium/calmodulin dependent serine protein kinase
		O94921	CDK14	cyclin dependent kinase 14
		Q5SW79	CEP170	centrosomal protein 170
		O14646	CHD1	chromodomain helicase DNA binding protein 1
		Q14839	CHD4	chromodomain helicase DNA binding protein 4
		Q8TD26	CHD6	chromodomain helicase DNA binding protein 6
		Q9UHD1	CHORDC1	cysteine and histidine rich domain containing 1
		Q9C0C6	CIPC	CLOCK interacting pacemaker
		P51795	CLCN5	chloride voltage-gated channel 5
		Q6UVW9	CLEC2A	C-type lectin domain family 2 member A
		Q3B7I2	CNPY1	canopy FGF signaling regulator 1
		P21554	CNR1	cannabinoid receptor 1
		A4D0V7	CPED1	cadherin like and PC-esterase domain containing 1
		Q05D32	CTDSPL2	CTD small phosphatase like 2
		Q13620	CUL4B	cullin 4B
		P19876	CXCL3	C-X-C motif chemokine ligand 3
		Q9UI36	DACH1	dachshund family transcription factor 1
		Q9NRI5	DISC1	DISC1 scaffold protein

		Q8NF50	DOCK8	dedicator of cytokinesis 8
		Q6XUX3	DSTYK	dual serine/threonine and tyrosine protein kinase
		Q13627	DYRK1A	dual specificity tyrosine phosphorylation regulated kinase 1A
		Q6PJG2	ELMSAN1	ELM2 and Myb/SANT domain containing 1
		Q9ULC0	EMCN	endomucin
		Q52LR7	EPC2	enhancer of polycomb homolog 2
		P54756	EPHA5	EPH receptor A5
		P11308	ERG	ETS transcription factor ERG
		Q96RQ1	ERGIC2	ERGIC and golgi 2
		Q86UK5	EVC2	EvC ciliary complex subunit 2
		Q9Y6X4	FAM169A	family with sequence similarity 169 member A
		Q969H0	FBXW7	F-box and WD repeat domain containing 7
		P02671	FGA	fibrinogen alpha chain
		Q01543	FLI1	Fli-1 proto-oncogene, ETS transcription factor
		O00358	FOXE1	forkhead box E1
		O60353	FZD6	frizzled class receptor 6
		Q5T7V8	GORAB	golgin, RAB6 interacting
		P00505	GOT2	glutamic-oxaloacetic transaminase 2
		Q9NW75	GPATCH2	G-patch domain containing 2
		Q9NWQ4	GPATCH2L	G-patch domain containing 2 like
		Q6ISB3	GRHL2	grainyhead like transcription factor 2
		P46976	GYG1	glycogenin 1
		Q99075	HBEGF	heparin binding EGF like growth factor
		Q5U5R9	HECTD2	HECT domain E3 ubiquitin protein ligase 2
		Q9UM44	HHLA2	HERV-H LTR-associating 2
		P35680	HNF1B	HNF1 homeobox B
		Q86VS8	HOOK3	hook microtubule tethering protein 3
		O95757	HSPA4L	heat shock protein family A (Hsp70) member 4 like
		P08069	IGF1R	insulin like growth factor 1 receptor

		Q5DX21	IGSF11	immunoglobulin superfamily member 11
		P27987	ITPKB	inositol-trisphosphate 3-kinase B
		Q15811	ITSN1	intersectin 1
		Q9Y6Y0	IVNS1ABP	influenza virus NS1A binding protein
		Q6IE81	JADE1	jade family PHD finger 1
		Q8N5Z5	KCTD17	potassium channel tetramerization domain containing 17
		P29375	KDM5A	lysine demethylase 5A
		Q5VWX1	KHDRBS2	KH RNA binding domain containing, signal transduction associated 2
		Q9ULL0	KIAA1210	KIAA1210
		Q9Y4X4	KLF12	Kruppel like factor 12
		Q8NEZ4	KMT2C	lysine methyltransferase 2C
		Q9NQR1	KMT5A	lysine methyltransferase 5A
		Q7Z3Z0	KRT25	keratin 25
		P13473	LAMP2	lysosomal associated membrane protein 2
		Q96JN0	LCOR	ligand dependent nuclear receptor corepressor
		Q52LA3	LIN52	lin-52 DREAM MuvB core complex component
		Q9NUP9	LIN7C	lin-7 homolog C, crumbs cell polarity complex component
		Q7L985	LINGO2	leucine rich repeat and Ig domain containing 2
		O60711	LPXN	leupaxin
		Q5VUJ6	LRCH2	leucine rich repeats and calponin homology domain containing 2
		Q32MZ4	LRRFIP1	LRR binding FLII interacting protein 1
		Q13449	LSAMP	limbic system associated membrane protein
		O95232	LUC7L3	LUC7 like 3 pre-mRNA splicing factor
		Q8IV03	LURAP1L	leucine rich adaptor protein 1 like
		P07948	LYN	LYN proto-oncogene, Src family tyrosine kinase
		P11137	MAP2	microtubule associated protein 2
		Q16819	MEP1A	meprin A subunit alpha

		P50579	METAP2	methionyl aminopeptidase 2
		Q9UM21	MGAT4A	alpha-1,3-mannosyl-glycoprotein 4-beta-N-acetylglucosaminyltransferase A
		Q9NPA3	MID1IP1	MID1 interacting protein 1
		Q8NBR6	MINDY2	MINDY lysine 48 deubiquitinase 2
		Q9HD23	MRS2	magnesium transporter MRS2
		Q9Y217	MTMR6	myotubularin related protein 6
		O43312	MTSS1	MTSS1, I-BAR domain containing
		Q9GZZ1	NAA50	N(alpha)-acetyltransferase 50, NatE catalytic subunit
		Q13765	NACA	nascent polypeptide associated complex subunit alpha
		O14524	NEMP1	nuclear envelope integral membrane protein 1
		O94916	NFAT5	nuclear factor of activated T cells 5
		Q9BS92	NIPSNAP3B	nipsnap homolog 3B
		P08949	NMB	neuromedin B
		Q9NPD7	NRN1	neuritin 1
		Q9Y2I2	NTNG1	netrin G1
		Q9Y266	NUDC	nuclear distribution C, dynein complex regulator
		Q9BZD4	NUF2	NDC80 kinetochore complex component NUF2
		Q9ULI1	NWD2	NACHT and WD repeat domain containing 2
		O95948	ONECUT2	one cut homeobox 2
		Q9H255	OR51E2	olfactory receptor family 51 subfamily E member 2
		Q9BXB4	OSBPL11	oxysterol binding protein like 11
		Q9BZF1	OSBPL8	oxysterol binding protein like 8
		Q86WC4	OSTM1	osteoclastogenesis associated transmembrane protein 1
		Q96BN8	OTULIN	OTU deubiquitinase with linear linkage specificity
		Q9BPZ3	PAIP2	poly(A) binding protein interacting protein 2
		Q9NVD7	PARVA	parvin alpha
		P11177	PDHB	pyruvate dehydrogenase E1 beta subunit
		Q15120	PDK3	pyruvate dehydrogenase kinase 3

		Q9BRX2	PELO	pelota mRNA surveillance and ribosome rescue factor
		Q8WV24	PHLDA1	pleckstrin homology like domain family A member 1
		O60346	PHLPP1	PH domain and leucine rich repeat protein phosphatase 1
		Q9BZ72	PITPNM2	phosphatidylinositol transfer protein membrane associated 2
		O43164	PJA2	praja ring finger ubiquitin ligase 2
		Q86W92	PPFIBP1	PPFIA binding protein 1
		Q08209	PPP3CA	protein phosphatase 3 catalytic subunit alpha
		Q8NI37	PPTC7	PTC7 protein phosphatase homolog
		Q7Z3G6	PRICKLE2	prickle planar cell polarity protein 2
		Q05209	PTPN12	protein tyrosine phosphatase, non-receptor type 12
		P17706	PTPN2	protein tyrosine phosphatase, non-receptor type 2
		Q4JDL3	PTPN20	protein tyrosine phosphatase, non-receptor type 20
		Q96PU8	QKI	QKI, KH domain containing RNA binding
		Q9BZG1	RAB38	RAB38, member RAS oncogene family
		P57729	RAB3B	RAB3B, member RAS oncogene family
		Q7Z5J4	RAI1	retinoic acid induced 1
		P61224	RAP1B	RAP1B, member of RAS oncogene family
		O60673	REV3L	REV3 like, DNA directed polymerase zeta catalytic subunit
		Q15382	RHEB	Ras homolog, mTORC1 binding
		Q96EQ8	RNF125	ring finger protein 125
		Q8TC41	RNF217	ring finger protein 217
		O60942	RNGTT	RNA guanylyltransferase and 5'-phosphatase
		Q6PCB5	RSBN1L	round spermatid basic protein 1 like
		Q96T23	RSF1	remodeling and spacing factor 1
		Q8WXA3	RUFY2	RUN and FYVE domain containing 2
		P19793	RXRA	retinoid X receptor alpha
		Q9NSC2	SALL1	spalt like transcription factor 1
		Q9UPU9	SAMD4A	sterile alpha motif domain containing 4A
		Q01826	SATB1	SATB homeobox 1

		P31431	SDC4	syndecan 4
		O95487	SEC24B	SEC24 homolog B, COPII coat complex component
		P49903	SEPHS1	selenophosphate synthetase 1
		Q9C0A6	SETD5	SET domain containing 5
		Q96HF1	SFRP2	secreted frizzled related protein 2
		Q9BWM7	SFXN3	sideroflexin 3
		O43556	SGCE	sarcoglycan epsilon
		Q0GE19	SLC10A7	solute carrier family 10 member 7
		P55011	SLC12A2	solute carrier family 12 member 2
		Q9UHW9	SLC12A6	solute carrier family 12 member 6
		P43003	SLC1A3	solute carrier family 1 member 3
		O95847	SLC25A27	solute carrier family 25 member 27
		O00337	SLC28A1	solute carrier family 28 member 1
		Q9Y6M5	SLC30A1	solute carrier family 30 member 1
		Q01650	SLC7A5	solute carrier family 7 member 5
		Q9H2G2	SLK	STE20 like kinase
		H3BMG3	SMKR1	small lysine rich protein 1
		O60641	SNAP91	synaptosome associated protein 91
		P35711	SOX5	SRY-box 5
		Q7Z699	SPRED1	sprouty related EVH1 domain containing 1
		Q68D10	SPTY2D1	SPT2 chromatin protein domain containing 1
		Q76I76	SSH2	slingshot protein phosphatase 2
		Q9NUL3	STAU2	staufen double-stranded RNA binding protein 2
		Q8NFT2	STEAP2	STEAP2 metalloreductase
		Q9UMX1	SUFU	SUFU negative regulator of hedgehog signaling
		P61956	SUMO2	small ubiquitin-like modifier 2
		O00268	TAF4	TATA-box binding protein associated factor 4
		Q7L7X3	TAOK1	TAO kinase 1
		Q3YBR2	TBRG1	transforming growth factor beta regulator 1

		Q96EI5	TCEAL4	transcription elongation factor A like 4
		Q96IP4	TENT5A	terminal nucleotidyltransferase 5A
		Q9HC24	TMBIM4	transmembrane BAX inhibitor motif containing 4
		Q9Y3A6	TMED5	transmembrane p24 trafficking protein 5
		Q6UXF1	TMEM108	transmembrane protein 108
		P36406	TRIM23	tripartite motif containing 23
		O60635	TSPAN1	tetraspanin 1
		P41732	TSPAN7	tetraspanin 7
		Q86TV6	TTC7B	tetratricopeptide repeat domain 7B
		O15042	U2SURP	U2 snRNP associated SURP domain containing
		Q9H0E7	USP44	ubiquitin specific peptidase 44
		Q92738	USP6NL	USP6 N-terminal like
		Q7Z5K2	WAPL	WAPL cohesin release factor
		Q9Y2I8	WDR37	WD repeat domain 37
		Q9H0M0	WWP1	WW domain containing E3 ubiquitin protein ligase 1
		Q9NQH7	XPNPEP3	X-prolyl aminopeptidase 3
		P46937	YAP1	Yes associated protein 1
		Q9BYJ9	YTHDF1	YTH N6-methyladenosine RNA binding protein 1
		O76080	ZFAND5	zinc finger AN1-type containing 5
		Q15911	ZFHX3	zinc finger homeobox 3
		Q86UP3	ZFHX4	zinc finger homeobox 4
		Q92618	ZNF516	zinc finger protein 516
		Q9HCE3	ZNF532	zinc finger protein 532
		Q8N8J6	ZNF615	zinc finger protein 615
		Q9NQZ8	ZNF71	zinc finger protein 71
**TargetScan**	**miR-200c-3p Upregulated 6 hours**	Q9BUR5	APOO	MICOS complex subunit MIC26 (Apolipoprotein O) (MICOS complex subunit MIC23) (Protein FAM121B)
		Q86WC4	OSTM1	Osteopetrosis-associated transmembrane protein 1 (Chloride channel 7 beta subunit)

		Q9BPZ3	PAIP2	Polyadenylate-binding protein-interacting protein 2 (PABP-interacting protein 2) (PAIP-2) (Poly(A)-binding protein-interacting protein 2)
**miRDB**		Q9NQ94	A1CF	APOBEC1 complementation factor
		Q8NER5	ACVR1C	activin A receptor type 1C
		O15072	ADAMTS3	ADAM metallopeptidase with thrombospondin type 1 motif 3
		P00325	ADH1B	alcohol dehydrogenase 1B (class I), beta polypeptide
		Q86V24	ADIPOR2	adiponectin receptor 2
		P51826	AFF3	AF4/FMR2 family member 3
		P52594	AGFG1	ArfGAP with FG repeats 1
		O43687, Q9P0M2	AKAP7	A-kinase anchoring protein 7
		P00352	ALDH1A1	aldehyde dehydrogenase 1 family member A1
		Q9UKV5	AMFR	autocrine motility factor receptor
		Q12955	ANK3	ankyrin 3
		Q6AI12	ANKRD40	ankyrin repeat domain 40
		Q8N8A2	ANKRD44	ankyrin repeat domain 44
		P56377	AP1S2	adaptor related protein complex 1 subunit sigma 2
		Q9BUR5	APOO	apolipoprotein O
		Q9P2F6	ARHGAP20	Rho GTPase activating protein 20
		O43182	ARHGAP6	Rho GTPase activating protein 6
		Q4LE39	ARID4B	AT-rich interaction domain 4B
		Q9Y4X5	ARIH1	ariadne RBR E3 ubiquitin protein ligase 1
		Q9Y2Y0	ARL2BP	ADP ribosylation factor like GTPase 2 binding protein
		Q9Y689	ARL5A	ADP ribosylation factor like GTPase 5A
		Q13510	ASAP1	ArfGAP with SH3 domain, ankyrin repeat and PH domain 1
		Q9ULI0	ATAD2B	ATPase family, AAA domain containing 2B
		Q8NHH9	ATL2	atlastin GTPase 2
		Q8NB49	ATP11C	ATPase phospholipid transporting 11C
		P06576	ATP5F1B	ATP synthase F1 subunit beta

		Q9Y487	ATP6V0A2	ATPase H+ transporting V0 subunit a2
		P54253	ATXN1	ataxin 1
		Q6Y288	B3GLCT	beta 3-glucosyltransferase
		Q9NY97	B3GNT2	UDP-GlcNAc:betaGal beta-1,3-N-acetylglucosaminyltransferase 2
		O43505	B4GAT1	beta-1,4-glucuronyltransferase 1
		Q92560	BAP1	BRCA1 associated protein 1
		Q9C0K0	BCL11B	BCL11B, BAF complex component
		A6H8Y1	BDP1	B double prime 1, subunit of RNA polymerase III transcription initiation factor IIIB
		Q9H694	BICC1	BicC family RNA binding protein 1
		P62952	BLCAP	BLCAP, apoptosis inducing factor
		Q6ZN30	BNC2	basonuclin 2
		Q6RI45	BRWD3	bromodomain and WD repeat domain containing 3
		Q6NUJ2	C11orf87	chromosome 11 open reading frame 87
		C9JLR9	C11orf95	chromosome 11 open reading frame 95
		Q14CZ0	C16orf72	chromosome 16 open reading frame 72
		Q9Y376	CAB39	calcium binding protein 39
		Q08AD1	CAMSAP2	calmodulin regulated spectrin associated protein family member 2
		P41180	CASR	calcium sensing receptor
		Q86V15	CASZ1	castor zinc finger 1
		P22681	CBL	Cbl proto-oncogene
		O00257	CBX4	chromobox 4
		Q9NQR7	CCDC177	coiled-coil domain containing 177
		Q8N4S0	CCDC82	coiled-coil domain containing 82
		O96020	CCNE2	cyclin E2
		Q5T5M9	CCNJ	cyclin J
		Q8N7R7	CCNYL1	cyclin Y like 1

		P55287	CDH11	cadherin 11
		Q9HBT6	CDH20	cadherin 20
		Q00537	CDK17	cyclin dependent kinase 17
		Q9Y232	CDYL	chromodomain Y like
		Q8N8U2	CDYL2	chromodomain Y like 2
		Q9BXF3	CECR2	CECR2, histone acetyl-lysine reader
		Q5VT06	CEP350	centrosomal protein 350
		Q9BYV8	CEP41	centrosomal protein 41
		Q5SZL2	CEP85L	centrosomal protein 85 like
		Q8IW35	CEP97	centrosomal protein 97
		Q9Y281	CFL2	cofilin 2
		Q9NZZ3	CHMP5	charged multivesicular body protein 5
		P52757	CHN2	chimerin 2
		Q9BU40	CHRDL1	chordin like 1
		P08172	CHRM2	cholinergic receptor muscarinic 2
		Q15825	CHRNA6	cholinergic receptor nicotinic alpha 6 subunit
		Q86X52	CHSY1	chondroitin sulfate synthase 1
		Q07065	CKAP4	cytoskeleton associated protein 4
		Q7Z460	CLASP1	cytoplasmic linker associated protein 1
		O75122	CLASP2	cytoplasmic linker associated protein 2
		Q9Y696	CLIC4	chloride intracellular channel 4
		P30622	CLIP1	CAP-Gly domain containing linker protein 1
		Q5SYC1	CLVS2	clavesin 2
		Q8N9A8	CNEP1R1	CTD nuclear envelope phosphatase 1 regulatory subunit 1
		Q6P9H4	CNKSR3	CNKSR family member 3
		Q9ULM6	CNOT6	CCR4-NOT transcription complex subunit 6
		Q92600	CNOT9	CCR4-NOT transcription complex subunit 9
		Q12860	CNTN1	contactin 1
		Q9Y5P4	COL4A3BP	collagen type IV alpha 3 binding protein

		Q99627	COPS8	COP9 signalosome subunit 8
		Q9ULV4	CORO1C	coronin 1C
		A4D0V7	CPED1	cadherin like and PC-esterase domain containing 1
		P06850	CRH	corticotropin releasing hormone
		P46109	CRKL	CRK like proto-oncogene, adaptor protein
		O75718	CRTAP	cartilage associated protein
		Q7Z407	CSMD3	CUB and Sushi multiple domains 3
		Q9Y6M4	CSNK1G3	casein kinase 1 gamma 3
		Q05D32	CTDSPL2	CTD small phosphatase like 2
		P39880	CUX1	cut like homeobox 1
		Q15438	CYTH1	cytohesin 1
		O43739	CYTH3	cytohesin 3
		Q9UI36	DACH1	dachshund family transcription factor 1
		Q96PD2	DCBLD2	discoidin, CUB and LCCL domain containing 2
		Q9BTE7	DCUN1D5	defective in cullin neddylation 1 domain containing 5
		Q96D03	DDIT4L	DNA damage inducible transcript 4 like
		Q6P3S1	DENND1B	DENN domain containing 1B
		Q6IQ26	DENND5A	DENN domain containing 5A
		Q6ZUT9	DENND5B	DENN domain containing 5B
		Q6ICB0	DESI1	desumoylating isopeptidase 1
		P23743	DGKA	diacylglycerol kinase alpha
		Q86XP1	DGKH	diacylglycerol kinase eta
		Q155Q3	DIXDC1	DIX domain containing 1
		P63167	DLC1	DLC1 Rho GTPase activating protein
		P51530	DNA2	DNA replication helicase/nuclease 2
		Q8TBM8	DNAJB14	DnaJ heat shock protein family (Hsp40) member B14
		O75953	DNAJB5	DnaJ heat shock protein family (Hsp40) member B5
		Q9UBS3	DNAJB9	DnaJ heat shock protein family (Hsp40) member B9
		Q13217	DNAJC3	DnaJ heat shock protein family (Hsp40) member C3

		Q9UBC3	DNMT3B	DNA methyltransferase 3 beta
		Q7L8W6	DPH6	diphthamine biosynthesis 6
		Q2PZI1	DPY19L1	dpy-19 like C-mannosyltransferase 1
		Q6ZPD9	DPY19L3	dpy-19 like C-mannosyltransferase 3
		Q9Y4J8	DTNA	dystrobrevin alpha
		P28562	DUSP1	dual specificity phosphatase 1
		Q86YF9	DZIP1	DAZ interacting zinc finger protein 1
		Q9BZQ6	EDEM3	ER degradation enhancing alpha-mannosidase like protein 3
		P52799	EFNB2	ephrin B2
		Q9GZT9	EGLN1	egl-9 family hypoxia inducible factor 1
		Q13144	EIF2B5	eukaryotic translation initiation factor 2B subunit epsilon
		O60573	EIF4E2	eukaryotic translation initiation factor 4E family member 2
		Q12926	ELAVL2	ELAV like RNA binding protein 2
		P41970	ELK3	ELK3, ETS transcription factor
		P28324	ELK4	ELK4, ETS transcription factor
		O00472	ELL2	elongation factor for RNA polymerase II 2
		Q8IZ81	ELMOD2	ELMO domain containing 2
		Q15369	ELOC	elongin C
		A6NNW6	ENO4	enolase 4
		Q12929	EPS8	epidermal growth factor receptor pathway substrate 8
		P11308	ERG	ETS transcription factor ERG
		A6NGS2	ERICH4	glutamate rich 4
		Q9UJM3	ERRFI1	ERBB receptor feedback inhibitor 1
		P62508	ESRRG	estrogen related receptor gamma
		P14921	ETS1	ETS proto-oncogene 1, transcription factor
		O60447	EVI5	ecotropic viral integration site 5
		Q9NVH0	EXD2	exonuclease 3'-5' domain containing 2
		Q9Y2C4	EXOG	exo/endonuclease G
		Q9BPY3	FAM118B	family with sequence similarity 118 member B

		Q6P4D5	FAM122C	family with sequence similarity 122C
		Q8IXS8	FAM126B	family with sequence similarity 126 member B
		Q96M60	FAM227B	family with sequence similarity 227 member B
		Q9UBU6	FAM8A1	family with sequence similarity 8 member A1
		Q9Y4F1	FARP1	FERM, ARH/RhoGEF and pleckstrin domain protein 1
		Q8TDW7	FAT3	FAT atypical cadherin 3
		Q8TB52	FBXO30	F-box protein 30
		Q7Z6M2	FBXO33	F-box protein 33
		Q969H0	FBXW7	F-box and WD repeat domain containing 7
		Q96AC1	FERMT2	fermitin family member 2
		Q9UHY8	FEZ2	fasciculation and elongation protein zeta 2
		Q13642	FHL1	four and a half LIM domains 1
		Q5HY92	FIGN	fidgetin, microtubule severing factor
		A6NMB9	FIGNL2	fidgetin like 2
		Q01543	FLI1	Fli-1 proto-oncogene, ETS transcription factor
		Q13045	FLII	FLII, actin remodeling protein
		P02751	FN1	fibronectin 1
		Q53EP0	FNDC3B	fibronectin type III domain containing 3B
		Q12946	FOXF1	forkhead box F1
		P55316	FOXG1	forkhead box G1
		P32314	FOXN2	forkhead box N2
		Q9Y2L6	FRMD4B	FERM domain containing 4B
		Q96NE9	FRMD6	FERM domain containing 6
		Q16658	FSCN1	fascin actin-bundling protein 1
		Q9BXM9	FSD1L	fibronectin type III and SPRY domain containing 1 like
		Q12841	FSTL1	follistatin like 1
		O75899	GABBR2	gamma-aminobutyric acid type B receptor subunit 2
		Q86XJ1	GAS2L3	growth arrest specific 2 like 3
		P43694	GATA4	GATA binding protein 4

		P55040	GEM	GTP binding protein overexpressed in skeletal muscle
		Q14161	GIT2	GIT ArfGAP 2
		P36383	GJC1	gap junction protein gamma 1
		Q86VQ1	GLCCI1	glucocorticoid induced 1
		P10071	GLI3	GLI family zinc finger 3
		P50148	GNAQ	G protein subunit alpha q
		Q92805	GOLGA1	golgin A1
		Q7Z5G4	GOLGA7	golgin A7
		Q9UKJ3	GPATCH8	G-patch domain containing 8
		P51674	GPM6A	glycoprotein M6A
		Q5T848	GPR158	G protein-coupled receptor 158
		Q86V85	GPR180	G protein-coupled receptor 180
		Q96D09	GPRASP2	G protein-coupled receptor associated sorting protein 2
		O15217	GSTA4	glutathione S-transferase alpha 4
		Q9UKN8	GTF3C4	general transcription factor IIIC subunit 4
		A4D1E9	GTPBP10	GTP binding protein 10
		Q02108	GUCY1A1	guanylate cyclase 1 soluble subunit alpha 1
		Q4G148	GXYLT1	glucoside xylosyltransferase 1
		Q9UKV0	HDAC9	histone deacetylase 9
		Q9H0R4	HDHD2	haloacid dehalogenase like hydrolase domain containing 2
		Q5U5R9	HECTD2	HECT domain E3 ubiquitin protein ligase 2
		Q86Z02	HIPK1	homeodomain interacting protein kinase 1
		Q9H422	HIPK3	homeodomain interacting protein kinase 3
		Q6NT76	HMBOX1	homeobox containing 1
		O15347	HMGB3	high mobility group box 3
		Q14103	HNRNPD	heterogeneous nuclear ribonucleoprotein D
		Q9UJC3	HOOK1	hook microtubule tethering protein 1
		Q86VS8	HOOK3	hook microtubule tethering protein 3
		Q7LGA3	HS2ST1	heparan sulfate 2-O-sulfotransferase 1

		O14792	HS3ST1	heparan sulfate-glucosamine 3-sulfotransferase 1
		Q9Y663	HS3ST3A1	heparan sulfate-glucosamine 3-sulfotransferase 3A1
		P48723	HSPA13	heat shock protein family A (Hsp70) member 13
		Q5VY09	IER5	immediate early response 5
		P11717	IGF2R	insulin like growth factor 2 receptor
		Q6WRI0	IGSF10	immunoglobulin superfamily member 10
		Q9UKS7	IKZF2	IKAROS family zinc finger 2
		Q96T52	IMMP2L	inner mitochondrial membrane peptidase subunit 2
		Q96T92	INSM2	INSM transcriptional repressor 2
		Q75QN2	INTS8	integrator complex subunit 8
		O95373	IPO7	importin 7
		P56199	ITGA1	integrin subunit alpha 1
		Q96AA8	JAKMIP2	janus kinase and microtubule interacting protein 2
		Q86VZ6	JAZF1	JAZF zinc finger 1
		Q9P266	JCAD	junctional cadherin 5 associated
		Q9P055	JKAMP	JNK1/MAPK8 associated membrane protein
		P05412	JUN	Jun proto-oncogene, AP-1 transcription factor subunit
		Q63ZY3	KANK2	KN motif and ankyrin repeat domains 2
		Q9NZV8	KCND2	potassium voltage-gated channel subfamily D member 2
		O43525	KCNQ3	potassium voltage-gated channel subfamily Q member 3
		Q6ZWB6	KCTD8	potassium channel tetramerization domain containing 8
		Q6UW63	KDELC1	KDEL motif containing 1
		Q6ZMT4	KDM7A	lysine demethylase 7A
		P35968	KDR	kinase insert domain receptor
		Q07666	KHDRBS1	KH RNA binding domain containing, signal transduction associated 1
		O15063	KIAA0355	KIAA0355
		Q15058	KIF14	kinesin family member 14
		O43474	KLF4	Kruppel like factor 4

		Q99612	KLF6	Kruppel like factor 6
		Q6KB66	KRT80	keratin 80
		Q16719	KYNU	kynureninase
		Q14739	LBR	lamin B receptor
		P48357	LEPR	leptin receptor
		Q9Y693	LHFPL6	LHFPL tetraspan subfamily member 6
		O14910	LIN7A	lin-7 homolog A, crumbs cell polarity complex component
		P28300	LOX	lysyl oxidase
		Q07954	LRP1	LDL receptor related protein 1
		Q9NZR2	LRP1B	LDL receptor related protein 1B
		Q8IWT6	LRRC8A	leucine rich repeat containing 8 VRAC subunit A
		P11137	MAP2	microtubule associated protein 2
		Q13233	MAP3K1	mitogen-activated protein kinase kinase kinase 1
		Q8IVH8	MAP4K3	mitogen-activated protein kinase kinase kinase kinase 3
		O95819	MAP4K4	mitogen-activated protein kinase kinase kinase kinase 4
		Q9Y4K4	MAP4K5	mitogen-activated protein kinase kinase kinase kinase 5
		Q13164	MAPK7	mitogen-activated protein kinase 7
		Q15691	MAPRE1	microtubule associated protein RP/EB family member 1
		O60337	MARCH6	membrane associated ring-CH-type finger 6
		P29966	MARCKS	myristoylated alanine rich protein kinase C substrate
		Q9Y4F3	MARF1	meiosis regulator and mRNA stability factor 1
		P43243	MATR3	matrin 3
		Q5VZF2	MBNL2	muscleblind like splicing regulator 2
		Q9NUK0	MBNL3	muscleblind like splicing regulator 3
		Q6ZWT7	MBOAT2	membrane bound O-acyltransferase domain containing 2
		Q8NI22	MCFD2	multiple coagulation factor deficiency 2
		Q9UHV7	MED13	mediator complex subunit 13
		Q86XN8	MEX3D	mex-3 RNA binding family member D
		Q13361	MFAP5	microfibril associated protein 5

		Q10469	MGAT2	mannosyl (alpha-1,6-)-glycoprotein beta-1,2-N-acetylglucosaminyltransferase
		Q86YT6	MIB1	mindbomb E3 ubiquitin protein ligase 1
		Q9NQG6	MIEF1	mitochondrial elongation factor 1
		Q8NBR6	MINDY2	MINDY lysine 48 deubiquitinase 2
		Q15546	MMD	monocyte to macrophage differentiation associated
		Q8NHV5	MOSMO	modulator of smoothened
		O75970	MPDZ	multiple PDZ domain crumbs cell polarity complex component
		Q6WCQ1	MPRIP	myosin phosphatase Rho interacting protein
		Q9HCI7	MSL2	MSL complex subunit 2
		P26038	MSN	moesin
		Q9Y483	MTF2	metal response element binding transcription factor 2
		Q765P7	MTSS1L	MTSS1L, I-BAR domain containing
		P10242	MYB	MYB proto-oncogene, transcription factor
		P0CAP1	MYZAP	myocardial zonula adherens protein
		Q13506	NAB1	NGFI-A binding protein 1
		Q8WY41	NANOS1	nanos C2HC-type zinc finger 1
		Q96NT1	NAP1L5	nucleosome assembly protein 1 like 5
		Q14596	NBR1	NBR1, autophagy cargo receptor
		O43639	NCK2	NCK adaptor protein 2
		Q15596	NCOA2	nuclear receptor coactivator 2
		P62166	NCS1	neuronal calcium sensor 1
		Q96NY8	NECTIN4	nectin cell adhesion molecule 4
		Q8NHV4	NEDD1	neural precursor cell expressed, developmentally down-regulated 1
		Q12857	NFIA	nuclear factor I A
		Q13253	NOG	noggin
		P46531	NOTCH1	notch 1
		P51513	NOVA1	NOVA alternative splicing regulator 1

		Q9UNW9	NOVA2	NOVA alternative splicing regulator 2
		O15118	NPC1	NPC intracellular cholesterol transporter 1
		P06748	NPM1	nucleophosmin 1
		P04150	NR3C1	nuclear receptor subfamily 3 group C member 1
		O00482	NR5A2	nuclear receptor subfamily 5 group A member 2
		Q9UHY1	NRBP1	nuclear receptor binding protein 1
		Q02297	NRG1	neuregulin 1
		P20783	NTF3	neurotrophin 3
		Q16620	NTRK2	neurotrophic receptor tyrosine kinase 2
		Q9NZJ9	NUDT4	nudix hydrolase 4
		Q7Z417	NUFIP2	nuclear FMR1 interacting protein 2
		Q6ZVC0	NYAP1	neuronal tyrosine phosphorylated phosphoinositide-3-kinase adaptor 1
		Q16625	OCLN	occludin
		Q9BXB4	OSBPL11	oxysterol binding protein like 11
		Q86WC4	OSTM1	osteoclastogenesis associated transmembrane protein 1
		Q01804	OTUD4	OTU deubiquitinase 4
		Q8N573	OXR1	oxidation resistance 1
		Q9NWQ8	PAG1	phosphoprotein membrane anchor with glycosphingolipid microdomains 1
		Q8TAB3	PCDH19	protocadherin 19
		Q15004	PCLAF	PCNA clamp associated factor
		Q96MG8	PCMTD1	protein-L-isoaspartate (D-aspartate) O-methyltransferase domain containing 1
		Q8N165	PDIK1L	PDLIM1 interacting kinase 1 like
		Q9NTI5	PDS5B	PDS5 cohesin associated factor B
		Q6PCE3	PGM2L1	phosphoglucomutase 2 like 1
		Q96KR7	PHACTR3	phosphatase and actin regulator 3
		P78562	PHEX	phosphate regulating endopeptidase homolog X-linked

		Q96EK2	PHF21B	PHD finger protein 21B
		Q8N3S3	PHTF2	putative homeodomain transcription factor 2
		Q8TCG2	PI4K2B	phosphatidylinositol 4-kinase type 2 beta
		Q9UBF8	PI4KB	phosphatidylinositol 4-kinase beta
		Q13492	PICALM	phosphatidylinositol binding clathrin assembly protein
		P42336	PIK3CA	phosphatidylinositol-4,5-bisphosphate 3-kinase catalytic subunit alpha
		Q9Y2I7	PIKFYVE	phosphoinositide kinase, FYVE-type zinc finger containing
		Q9P1W9	PIM2	Pim-2 proto-oncogene, serine/threonine kinase
		Q9BZ71	PITPNM3	PITPNM family member 3
		P98161	PKD1	polycystin 1, transient receptor potential channel interacting
		P61925	PKIA	cAMP-dependent protein kinase inhibitor alpha
		Q15111	PLCL1	phospholipase C like 1 (inactive)
		O14495	PLPP3	phospholipid phosphatase 3
		Q7Z2D5	PLPPR4	phospholipid phosphatase related 4
		O60486	PLXNC1	plexin C1
		Q13794	PMAIP1	phorbol-12-myristate-13-acetate-induced protein 1
		Q9UBT6	POLK	DNA polymerase kappa
		Q13136	PPFIA1	PTPRF interacting protein alpha 1
		Q8NEY8	PPHLN1	periphilin 1
		O75688	PPM1B	protein phosphatase, Mg2+/Mn2+ dependent 1B
		Q8WY54	PPM1E	protein phosphatase, Mg2+/Mn2+ dependent 1E
		P49593	PPM1F	protein phosphatase, Mg2+/Mn2+ dependent 1F
		Q96QC0	PPP1R10	protein phosphatase 1 regulatory subunit 10
		Q6NYC8	PPP1R18	protein phosphatase 1 regulatory subunit 18
		Q9ULJ8	PPP1R9A	protein phosphatase 1 regulatory subunit 9A
		Q96SB3	PPP1R9B	protein phosphatase 1 regulatory subunit 9B
		P30154	PPP2R1B	protein phosphatase 2 scaffold subunit Abeta
		Q16537	PPP2R5E	protein phosphatase 2 regulatory subunit B'epsilon

		Q9NY27	PPP4R2	protein phosphatase 4 regulatory subunit 2
		O75626	PRDM1	PR/SET domain 1
		Q9HAZ2	PRDM16	PR/SET domain 16
		P22694	PRKACB	protein kinase cAMP-activated catalytic subunit beta
		Q13976	PRKG1	protein kinase cGMP-dependent 1
		Q9HC23	PROK2	prokineticin 2
		Q2VWP7	PRTG	protogenin
		Q9Y617	PSAT1	phosphoserine aminotransferase 1
		O75475	PSIP1	PC4 and SFRS1 interacting protein 1
		Q7Z6K3	PTAR1	protein prenyltransferase alpha subunit repeat containing 1
		O95758	PTBP3	polypyrimidine tract binding protein 3
		P12272	PTHLH	parathyroid hormone like hormone
		Q05209	PTPN12	protein tyrosine phosphatase, non-receptor type 12
		Q15678	PTPN14	protein tyrosine phosphatase, non-receptor type 14
		Q16825	PTPN21	protein tyrosine phosphatase, non-receptor type 21
		P23471	PTPRZ1	protein tyrosine phosphatase, receptor type Z1
		Q8TB72	PUM2	pumilio RNA binding family member 2
		Q96PU8	QKI	QKI, KH domain containing RNA binding
		Q7L804	RAB11FIP2	RAB11 family interacting protein 2
		Q9UL25	RAB21	RAB21, member RAS oncogene family
		Q6VN20	RANBP10	RAN binding protein 10
		Q96S59	RANBP9	RAN binding protein 9
		P61224	RAP1B	RAP1B, member of RAS oncogene family
		Q9Y3L5	RAP2C	RAP2C, member of RAS oncogene family
		Q15283	RASA2	RAS p21 protein activator 2
		Q8NHQ8	RASSF8	Ras association domain family member 8
		O43251	RBFOX2	RNA binding fox-1 homolog 2
		A6NFN3	RBFOX3	RNA binding fox-1 homolog 3
		Q9H1K0	RBSN	rabenosyn, RAB effector

		Q8IZV5	RDH10	retinol dehydrogenase 10
		P35241	RDX	radixin
		O95980	RECK	reversion inducing cysteine rich protein with kazal motifs
		Q9H902	REEP1	receptor accessory protein 1
		Q9NZL6	RGL1	ral guanine nucleotide dissociation stimulator like 1
		Q4ADV7	RIC1	RIC1 homolog, RAB6A GEF complex partner 1
		Q9UQ26	RIMS2	regulating synaptic membrane exocytosis 2
		O43353	RIPK2	receptor interacting serine/threonine kinase 2
		P61587	RND3	Rho family GTPase 3
		Q9NV58	RNF19A	ring finger protein 19A, RBR E3 ubiquitin protein ligase
		Q99496	RNF2	ring finger protein 2
		P10155	RO60	Ro60, Y RNA binding protein
		O75116	ROCK2	Rho associated coiled-coil containing protein kinase 2
		P51812	RPS6KA3	ribosomal protein S6 kinase A3
		P23443	RPS6KB1	ribosomal protein S6 kinase B1
		Q9Y3B9	RRP15	ribosomal RNA processing 15 homolog
		Q92541	RTF1	RTF1 homolog, Paf1/RNA polymerase II complex component
		Q8IZC4	RTKN2	rhotekin 2
		Q8N2Y8	RUSC2	RUN and SH3 domain containing 2
		Q96BU1	S100PBP	S100P binding protein
		Q8IVN8	SBSPON	somatomedin B and thrombospondin type 1 domain containing
		Q8N9R8	SCAI	suppressor of cancer cell invasion
		O15126	SCAMP1	secretory carrier membrane protein 1
		O00767	SCD	stearoyl-CoA desaturase
		Q14524	SCN5A	sodium voltage-gated channel alpha subunit 5
		Q9UQD0	SCN8A	sodium voltage-gated channel alpha subunit 8
		Q9NQ03	SCRT2	scratch family transcriptional repressor 2
		P34741	SDC2	syndecan 2
		Q15436	SEC23A	Sec23 homolog A, coat complex II component

		Q93073	SECISBP2L	SECIS binding protein 2 like
		Q13275	SEMA3F	semaphorin 3F
		Q8NFY4	SEMA6D	semaphorin 6D
		Q9NRX5	SERINC1	serine incorporator 1
		Q99574	SERPINI1	serpin family I member 1
		Q9Y6P5	SESN1	sestrin 1
		P58005	SESN3	sestrin 3
		Q9H9B4	SFXN1	sideroflexin 1
		O43556	SGCE	sarcoglycan epsilon
		Q9BQI5	SGIP1	SH3 domain GRB2 like endophilin interacting protein 1
		Q9BRV8	SIKE1	suppressor of IKBKE 1
		Q15475	SIX1	SIX homeobox 1
		Q13336	SLC14A1	solute carrier family 14 member 1 (Kidd blood group)
		P43004	SLC1A2	solute carrier family 1 member 2
		Q8NFF2	SLC24A4	solute carrier family 24 member 4
		Q96CQ1	SLC25A36	solute carrier family 25 member 36
		Q8NEW0	SLC30A7	solute carrier family 30 member 7
		Q969S0	SLC35B4	solute carrier family 35 member B4
		P0CK97	SLC35E2A	solute carrier family 35 member E2A
		P0CK96	SLC35E2B	solute carrier family 35 member E2B
		Q15043	SLC39A14	solute carrier family 39 member 14
		Q9Y6M7	SLC4A7	solute carrier family 4 member 7
		P30531	SLC6A1	solute carrier family 6 member 1
		P48066	SLC6A11	solute carrier family 6 member 11
		Q8IX21	SLF2	SMC5-SMC6 complex localization factor 2
		O94813	SLIT2	slit guidance ligand 2
		Q96PX8	SLITRK1	SLIT and NTRK like family member 1
		Q9H2G2	SLK	STE20 like kinase

		Q96GM5	SMARCD1	SWI/SNF related, matrix associated, actin dependent regulator of chromatin, subfamily d, member 1
		P08579	SNRPB2	small nuclear ribonucleoprotein polypeptide B2
		P57768	SNX16	sorting nexin 16
		P48431	SOX2	SRY-box 2
		O60271	SPAG9	sperm associated antigen 9
		Q7Z6B7	SRGAP1	SLIT-ROBO Rho GTPase activating protein 1
		P30626	SRI	sorcin
		Q13043	STK4	serine/threonine kinase 4
		Q9C0K7	STRADB	STE20 related adaptor beta
		O43815	STRN	striatin
		Q8IWU6	SULF1	sulfatase 1
		O60279	SUSD5	sushi domain containing 5
		Q15022	SUZ12	SUZ12, polycomb repressive complex 2 subunit
		Q6ZW31	SYDE1	synapse defective Rho GTPase homolog 1
		O43426	SYNJ1	synaptojanin 1
		Q86TM6	SYVN1	synoviolin 1
		Q16514	TAF12	TATA-box binding protein associated factor 12
		Q03519	TAP2	transporter 2, ATP binding cassette subfamily B member
		O60347	TBC1D12	TBC1 domain family member 12
		O75347	TBCA	tubulin folding cofactor A
		Q9UHD2	TBK1	TANK binding kinase 1
		Q9BZK7	TBL1XR1	transducin beta like 1 X-linked receptor 1
		O95935	TBX18	T-box 18
		Q8N3R3	TCAIM	T cell activation inhibitor, mitochondrial
		Q8NDF8	TENT4B	terminal nucleotidyltransferase 4B
		P05549	TFAP2A	transcription factor AP-2 alpha
		Q9NVV9	THAP1	THAP domain containing 1
		Q9H0W7	THAP2	THAP domain containing 2

		P16035	TIMP2	TIMP metallopeptidase inhibitor 2
		Q9Y490	TLN1	talin 1
		Q9Y4G6	TLN2	talin 2
		Q9UIK5	TMEFF2	transmembrane protein with EGF like and two follistatin like domains 2
		Q6ZRR5	TMEM136	transmembrane protein 136
		Q86X19	TMEM17	transmembrane protein 17
		Q9NYL9	TMOD3	tropomodulin 3
		Q9H1E5	TMX4	thioredoxin related transmembrane protein 4
		P50616	TOB1	transducer of ERBB2, 1
		Q9Y4F4	TOGARAM1	TOG array regulator of axonemal microtubules 1
		Q9Y2L5	TRAPPC8	trafficking protein particle complex 8
		Q9UKU6	TRHDE	thyrotropin releasing hormone degrading enzyme
		P36406	TRIM23	tripartite motif containing 23
		Q9UPN9	TRIM33	tripartite motif containing 33
		Q9P272	TRMT9B	tRNA methyltransferase 9B (putative)
		Q15714	TSC22D1	TSC22 domain family member 1
		O75157	TSC22D2	TSC22 domain family member 2
		Q9BXA7	TSSK1B	testis specific serine kinase 1B
		P07437	TUBB	tubulin beta class I
		Q712K3	UBE2R2	ubiquitin conjugating enzyme E2 R2
		Q96B02	UBE2W	ubiquitin conjugating enzyme E2 W
		Q6BDS2	UHRF1BP1	UHRF1 binding protein 1
		Q8IYT8	ULK2	unc-51 like autophagy activating kinase 2
		Q9UHP3	USP25	ubiquitin specific peptidase 25
		A6NNY8	USP27X	ubiquitin specific peptidase 27 X-linked
		Q92738	USP6NL	USP6 N-terminal like
		Q7L8A9	VASH1	vasohibin 1
		Q86V25	VASH2	vasohibin 2

		Q9HCJ6	VAT1L	vesicle amine transport 1 like
		P15692	VEGFA	vascular endothelial growth factor A
		P98155	VLDLR	very low density lipoprotein receptor
		Q7Z5K2	WAPL	WAPL cohesin release factor
		Q92558	WASF1	WAS protein family member 1
		Q9UPY6	WASF3	WAS protein family member 3
		Q6UXN9	WDR82	WD repeat domain 82
		O43516	WIPF1	WAS/WASL interacting protein family member 1
		Q9UBV4	WNT16	Wnt family member 16
		Q9ULE0	WWC3	WWC family member 3
		Q5GH76	XKR4	XK related 4
		Q9H6D3	XKR8	XK related 8
		Q96QA6	YPEL2	yippee like 2
		P31946	YWHAB	tyrosine 3-monooxygenase/tryptophan 5-monooxygenase activation protein beta
		P61981	YWHAG	tyrosine 3-monooxygenase/tryptophan 5-monooxygenase activation protein gamma
		Q96DT7	ZBTB10	zinc finger and BTB domain containing 10
		Q8NAP3	ZBTB38	zinc finger and BTB domain containing 38
		Q9UPT8	ZC3H4	zinc finger CCCH-type containing 4
		P61129	ZC3H6	zinc finger CCCH-type containing 6
		Q8N2G6	ZCCHC24	zinc finger CCHC-type containing 24
		Q8IVQ6	ZDHHC21	zinc finger DHHC-type containing 21
		P37275	ZEB1	zinc finger E-box binding homeobox 1
		O60315	ZEB2	zinc finger E-box binding homeobox 2
		Q6FIF0	ZFAND6	zinc finger AN1-type containing 6
		Q8WW38	ZFPM2	zinc finger protein, FOG family member 2
		P17010	ZFX	zinc finger protein X-linked
		Q15776	ZKSCAN8	zinc finger with KRAB and SCAN domains 8

		Q9HA38	ZMAT3	zinc finger matrin-type 3
		P52739	ZNF131	zinc finger protein 131
		O75362	ZNF217	zinc finger protein 217
		Q9NZL3	ZNF224	zinc finger protein 224
		Q5BKZ1	ZNF326	zinc finger protein 326
		Q9HCE3	ZNF532	zinc finger protein 532
		Q9Y462	ZNF711	zinc finger protein 711
		Q9H7M6	ZSWIM4	zinc finger SWIM-type containing 4
		Q9C0D3	ZYG11B	zyg-11 family member B, cell cycle regulator
**TargetScan**	**miR-222-5p Downregulated 24 hours**	A0A024R3P9	ACBD3	Acyl-Coenzyme A binding domain containing 3, isoform CRA_a
		Q99996	AKAP9	A-kinase anchor protein 9 (AKAP-9) (A-kinase anchor protein 350 kDa) (AKAP 350) (hgAKAP 350) (A-kinase anchor protein 450 kDa) (AKAP 450) (AKAP 120-like protein) (Centrosome- and Golgi-localized PKN-associated protein) (CG-NAP) (Protein hyperion) (Protein kinase A-anchoring protein 9) (PRKA9) (Protein yotiao)
		P51648	ALDH3A2	Aldehyde dehydrogenase family 3 member A2 (EC 1.2.1.3) (EC 1.2.1.94) (Aldehyde dehydrogenase 10) (Fatty aldehyde dehydrogenase) (Microsomal aldehyde dehydrogenase)
		Q86XS5	ANGPTL5	Angiopoietin-related protein 5 (Angiopoietin-like protein 5)
		A6NGH8	ANKRD61	Ankyrin repeat domain-containing protein 61
		P02749	APOH	Beta-2-glycoprotein 1 (APC inhibitor) (Activated protein C-binding protein) (Anticardiolipin cofactor) (Apolipoprotein H) (Apo-H) (Beta-2-glycoprotein I) (B2GPI) (Beta(2)GPI)

		P24539	ATP5F1	ATP synthase F(0) complex subunit C3, mitochondrial (ATP synthase lipid-binding protein) (ATP synthase membrane subunit c locus 3) (ATP synthase proteolipid P3) (ATP synthase proton-transporting mitochondrial F(0) complex subunit C3) (ATPase protein 9) (ATPase subunit c)
		Q9BXK5	BCL2L13	Bcl-2-like protein 13 (Bcl2-L-13) (Bcl-rambo) (Protein Mil1)
		Q9Y5K6	CD2AP	Cyclin-G1 (Cyclin-G)
		Q49AH0	CDNF	CDKN2A-interacting protein (Collaborator of ARF)
		Q8N0S6	CENPL	CENPB DNA-binding domain-containing protein 1
		Q9Y281	CFL2	Cofilin-2 (Cofilin, muscle isoform)
		P20309	CHRM3	Serine/threonine-protein kinase Chk2 (EC 2.7.11.1) (CHK2 checkpoint homolog) (Cds1 homolog) (Hucds1) (hCds1) (Checkpoint kinase 2)
		P61024	CKS1B	Cytoskeleton-associated protein 4 (63-kDa cytoskeleton-linking membrane protein) (Climp-63) (p63)
		Q8IUQ0	CLVS1	Calsyntenin-1 (Alcadein-alpha) (Alc-alpha) (Alzheimer-related cadherin-like protein) (Non-classical cadherin XB31alpha) [Cleaved into: Soluble Alc-alpha (SAlc-alpha); CTF1-alpha (C-terminal fragment 1-alpha)]
		A4D2H0	CTAGE15	cTAGE family member 15
		Q86UF2	CTAGE6	cTAGE family member 6
		O14625	CXCL11	C-X-C motif chemokine 11
		Q6ZMK1	CYHR1	Cysteine and histidine-rich protein 1
		O95786	DDX58	Antiviral innate immune response receptor RIG-I
		Q8WUY9	DEPDC1B	DEP domain-containing protein 1B
		Q9NYP3	DONSON	Protein downstream neighbor of Son (B17)
		Q92784	DPF3	Zinc finger protein DPF3
		Q7L2H7	EIF3M	Eukaryotic translation initiation factor 3 subunit M
		Q6IPR1	ETFRF1	lectron transfer flavoprotein regulatory factor 1

		O60447	EVI5	Ecotropic viral integration site 5 protein homolog
		O43320	FGF16	Fibroblast growth factor 16 (FGF-16)
		P21781	FGF7	Fibroblast growth factor 7 (FGF-7) (Heparin-binding growth factor 7) (HBGF-7) (Keratinocyte growth factor)
		P14867	GABRA1	Gamma-aminobutyric acid receptor subunit alpha-1 (GABA(A) receptor subunit alpha-1)
		Q9UG22	GIMAP2	GTPase IMAP family member 2 (Immunity-associated protein 2) (hIMAP2)
		Q03113	GNA12	Guanine nucleotide-binding protein subunit alpha-12 (G alpha-12) (G-protein subunit alpha-12)
		Q3T906	GNPTAB	N-acetylglucosamine-1-phosphotransferase subunits alpha/beta (EC 2.7.8.17) (GlcNAc-1-phosphotransferase subunits alpha/beta) (Stealth protein GNPTAB) (UDP-N-acetylglucosamine-1-phosphotransferase subunits alpha/beta) [Cleaved into: N-acetylglucosamine-1-phosphotransferase subunit alpha; N-acetylglucosamine-1-phosphotransferase subunit beta]
		O95249	GOSR1	Golgi SNAP receptor complex member 1 (28 kDa Golgi SNARE protein) (28 kDa cis-Golgi SNARE p28) (GOS-28)
		Q53EU6	GPAT3	Glycerol-3-phosphate acyltransferase 3 (GPAT-3) (EC 2.3.1.15) (1-acyl-sn-glycerol-3-phosphate O-acyltransferase 10) (AGPAT 10) (1-acyl-sn-glycerol-3-phosphate O-acyltransferase 9) (1-AGP acyltransferase 9) (1-AGPAT 9) (EC 2.3.1.51) (Acyl-CoA:glycerol-3-phosphate acyltransferase 3) (hGPAT3) (Lung cancer metastasis-associated protein 1) (Lysophosphatidic acid acyltransferase theta) (LPAAT-theta) (MAG-1)
		Q9NYM4	GPR83	Probable G-protein coupled receptor 83 (G-protein coupled receptor 72)
		Q6UWX4	HHIPL2	HHIP-like protein 2

		P01889	HLA-B	HLA class I histocompatibility antigen, B alpha chain (Human leukocyte antigen B) (HLA-B)
		Q8IZT8	HS3ST5	Heparan sulfate glucosamine 3-O-sulfotransferase 5 (EC 2.8.2.23) (Heparan sulfate D-glucosaminyl 3-O-sulfotransferase 5) (3-OST-5) (Heparan sulfate 3-O-sulfotransferase 5) (h3-OST-5)
		Q3SXM5	HSDL1	Inactive hydroxysteroid dehydrogenase-like protein 1 (Short chain dehydrogenase/reductase family 12C member 3)
		Q02363	ID2	DNA-binding protein inhibitor ID-2 (Class B basic helix-loop-helix protein 26) (bHLHb26) (Inhibitor of DNA binding 2) (Inhibitor of differentiation 2)
		O95239	KIF4A	Chromosome-associated kinesin KIF4A
		Q3LI77	KRTAP13-4	Keratin-associated protein 5-3 (Keratin-associated protein 5-9) (Keratin-associated protein 5.3) (Keratin-associated protein 5.9) (UHS KerB-like) (Ultrahigh sulfur keratin-associated protein 5.3)
		A6NK58	LIPT2	Putative lipoyltransferase 2, mitochondrial (EC 2.3.1.181) (Lipoate-protein ligase B) (Lipoyl/octanoyl transferase) (Octanoyl-[acyl-carrier-protein]-protein N-octanoyltransferase)
		Q8TAP4	LMO3	LIM domain only protein 3 (LMO-3) (Neuronal-specific transcription factor DAT1) (Rhombotin-3)
		Q5VT66	MARC1	Mitochondrial amidoxime-reducing component 1 (mARC1) (EC 1.7.-.-) (Molybdenum cofactor sulfurase C-terminal domain-containing protein 1) (MOSC domain-containing protein 1) (Moco sulfurase C-terminal domain-containing protein 1)
		P11226	MBL2	Mannose-binding protein C (MBP-C) (Collectin-1) (MBP1) (Mannan-binding protein) (Mannose-binding lectin)
		Q96LB0	MRGPRX3	Mas-related G-protein coupled receptor member X3 (Sensory neuron-specific G-protein coupled receptor 1/2)

		Q9NX20	MRPL16	39S ribosomal protein L16, mitochondrial (L16mt) (MRP-L16) (Mitochondrial large ribosomal subunit protein uL16m)
		P13995	MTHFD2	Bifunctional methylenetetrahydrofolate dehydrogenase/cyclohydrolase, mitochondrial [Includes: NAD-dependent methylenetetrahydrofolate dehydrogenase (EC 1.5.1.15); Methenyltetrahydrofolate cyclohydrolase (EC 3.5.4.9)]
		Q9UBE8	NLK	Neuropilin and tolloid-like protein 1 (Brain-specific transmembrane protein containing 2 CUB and 1 LDL-receptor class A domains protein 1)
		Q9BZ95	NSD3	Histone-lysine N-methyltransferase NSD3 (EC 2.1.1.-) (Nuclear SET domain-containing protein 3) (Protein whistle) (WHSC1-like 1 isoform 9 with methyltransferase activity to lysine) (Wolf-Hirschhorn syndrome candidate 1-like protein 1) (WHSC1-like protein 1)
		P49757	NUMB	Protein numb homolog (h-Numb) (Protein S171)
		Q9P0S3	ORMDL1	ORM1-like protein 1 (Adoplin-1)
		Q96FC7	PHYHIPL	Phosphoinositide 3-kinase regulatory subunit 5 (PI3-kinase regulatory subunit 5) (PI3-kinase p101 subunit) (Phosphatidylinositol 4,5-bisphosphate 3-kinase regulatory subunit) (PtdIns-3-kinase regulatory subunit) (Protein FOAP-2) (PtdIns-3-kinase p101) (p101-PI3K)
		Q12796	PNRC1	Protein O-glucosyltransferase 1 (EC 2.4.1.-) (CAP10-like 46 kDa protein) (hCLP46) (KTEL motif-containing protein 1) (Myelodysplastic syndromes relative protein) (O-glucosyltransferase Rumi homolog) (hRumi) (Protein O-xylosyltransferase POGLUT1) (EC 2.4.2.26)
		Q8WVI7	PPP1R1C	Protein phosphatase 1 regulatory subunit 1C
		Q96BW5	PTER	Phosphotriesterase-related protein
		Q9H0N0	RAB6C	Ras-related protein Rab-6C

		O60896	RAMP3	Receptor activity-modifying protein 3
		P10114	RAP2A	Ras-related protein Rap-2a
		P06400	RB1	Retinoblastoma-associated protein
		Q8WVD5	RNF141	RING finger protein 141
		Q9H6T3	RPAP3	RNA polymerase II-associated protein 3
		O00767	SCD	Stearoyl-CoA desaturase
		P49908	SELENOP	Selenoprotein P
		P07093	SERPINE2	Glia-derived nexin
		Q8NCS7	SLC44A5	Choline transporter-like protein 5
		Q8TCU3	SLC7A13	Solute carrier family 7 member 13
		A7E240	SLC8A3	SLC8A3 protein
		Q6IEE8	SLFN12L	Schlafen family member 12-like
		Q9BQ49	SMIM7	Small integral membrane protein 7
		O14508	SOCS2	Suppressor of cytokine signaling 2
		P0C7L1	SPINK8	Serine protease inhibitor Kazal-type 8
		O95793	STAU1	Double-stranded RNA-binding protein Staufen homolog 1
		Q9ULQ0	STRIP2	Striatin-interacting protein 2
		Q96NH3	TBC1D32	Protein broad-minded
		Q0IIM8	TBC1D8B	TBC1 domain family member 8B
		Q96QE5	TEFM	Transcription elongation factor, mitochondrial
		O14948	TFEC	Transcription factor EC (TFE-C) (Class E basic helix-loop-helix protein 34) (bHLHe34) (Transcription factor EC-like) (hTFEC-L)
		Q9NUM4	TMEM106B	Transmembrane protein 106B
		Q9NS68	TNFRSF19	Tumor necrosis factor receptor superfamily member 19 (TRADE) (Toxicity and JNK inducer)
		P50591	TNFSF10	Tumor necrosis factor ligand superfamily member 10 (Apo-2 ligand) (Apo-2L) (TNF-related apoptosis-inducing ligand) (Protein TRAIL) (CD antigen CD253)

		P62995	TRA2B	Transformer-2 protein homolog beta (TRA-2 beta) (TRA2-beta) (hTRA2-beta) (Splicing factor, arginine/serine-rich 10) (Transformer-2 protein homolog B)
		Q6PF06	TRMT10B	tRNA methyltransferase 10 homolog B (EC 2.1.1.221) (RNA (guanine-9-)-methyltransferase domain-containing protein 3) (tRNA (guanine(9)-N(1))-methyltransferase TRMT10B)
		Q9BVS5	TRMT61B	tRNA (adenine(58)-N(1))-methyltransferase, mitochondrial (EC 2.1.1.220) (mRNA methyladenosine-N(1)-methyltransferase) (EC 2.1.1.-)
		Q8IYU4	UBQLNL	Ubiquilin-like protein
		Q96BW1	UPRT	Uracil phosphoribosyltransferase homolog
		Q8IUA0	WFDC8	WAP four-disulfide core domain protein 8 (Putative protease inhibitor WAP8)
		Q14593	ZNF273	Zinc finger protein 273 (Zinc finger protein HZF9)
		P35789	ZNF93	Zinc finger protein 93 (Zinc finger protein 505) (Zinc finger protein HTF34)
		Q62005	Zp1	Zona pellucida sperm-binding protein 1 (Zona pellucida glycoprotein 1) (Zp-1) [Cleaved into: Processed zona pellucida sperm-binding protein 1]
		Q15019	SEPT2	septin 2
**miRDB**		O94929	ABLIM3	actin binding LIM protein family member 3
		O15072	ADAMTS3	ADAM metallopeptidase with thrombospondin type 1 motif 3
		P82987	ADAMTSL3	ADAMTS like 3
		Q09666	AHNAK	AHNAK nucleoprotein
		Q9UIJ7	AK3	adenylate kinase 3
		Q99996	AKAP9	A-kinase anchoring protein 9
		P51648	ALDH3A2	aldehyde dehydrogenase 3 family member A2
		Q9H161	ALX4	ALX homeobox 4
		Q86XS5	ANGPTL5	angiopoietin like 5

		O75179	ANKRD17	ankyrin repeat domain 17
		Q8N8A2	ANKRD44	ankyrin repeat domain 44
		Q86W74	ANKRD46	ankyrin repeat domain 46
		Q9BZZ5	API5	apoptosis inhibitor 5
		P02749	APOH	apolipoprotein H
		Q15041	ARL6IP1	ADP ribosylation factor like GTPase 6 interacting protein 1
		Q8N6S5	ARL6IP6	ADP ribosylation factor like GTPase 6 interacting protein 6
		P15848	ARSB	arylsulfatase B
		Q12797	ATAD1	ATPase family, AAA domain containing 1
		P48201	ATP5MC3	ATP synthase membrane subunit c locus 3
		Q8NEY4	ATP6V1C2	ATPase H+ transporting V1 subunit C2
		Q9H7T9	AUNIP	aurora kinase A and ninein interacting protein
		O75815	BCAR3	BCAR3, NSP family adaptor protein
		Q9NYF8	BCLAF1	BCL2 associated transcription factor 1
		A8MU93	C17orf100	chromosome 17 open reading frame 100
		Q8IXQ3	C9orf40	chromosome 9 open reading frame 40
		Q05682	CALD1	caldesmon 1
		Q9Y6Q1	CAPN6	calpain 6
		Q14444	CAPRIN1	cell cycle associated protein 1
		A6NKD9	CCDC85C	coiled-coil domain containing 85C
		Q3V6T2	CCDC88A	coiled-coil domain containing 88A
		Q9Y5K6	CD2AP	CD2 associated protein
		Q9HCU4	CELSR2	cadherin EGF LAG seven-pass G-type receptor 2
		Q5SZL2	CEP85L	centrosomal protein 85 like
		Q9Y281	CFL2	cofilin 2
		Q70JA7	CHSY3	chondroitin sulfate synthase 3
		P21554	CNR1	cannabinoid receptor 1
		Q99715	COL12A1	collagen type XII alpha 1 chain
		Q92793	CREBBP	CREB binding protein

		Q13617	CUL2	cullin 2
		P11511	CYP19A1	cytochrome P450 family 19 subfamily A member 1
		Q9NX09	DDIT4	DNA damage inducible transcript 4
		O95786	DDX58	DExD/H-box helicase 58
		Q8WUY9	DEPDC1B	DEP domain containing 1B
		Q86Y56	DNAAF5	dynein axonemal assembly factor 5
		Q96M86	DNHD1	dynein heavy chain domain 1
		O94769	ECM2	extracellular matrix protein 2
		Q92611	EDEM1	ER degradation enhancing alpha-mannosidase like protein 1
		Q96C19	EFHD2	EF-hand domain family member D2
		Q8N766	EMC1	ER membrane protein complex subunit 1
		Q09472	EP300	E1A binding protein p300
		P54756	EPHA5	EPH receptor A5
		Q8IV48	ERI1	exoribonuclease 1
		O60447	EVI5	ecotropic viral integration site 5
		Q9BXW9	FANCD2	FA complementation group D2
		Q96IG2	FBXL20	F-box and leucine rich repeat protein 20
		P10109	FDX1	ferredoxin 1
		P09038	FGF2	fibroblast growth factor 2
		P21781	FGF7	fibroblast growth factor 7
		Q5TBA9	FRY	FRY microtubule binding protein
		A1L4K1	FSD2	fibronectin type III and SPRY domain containing 2
		P51114	FXR1	FMR1 autosomal homolog 1
		Q9UN86	G3BP2	G3BP stress granule assembly factor 2
		Q9NQR9	G6PC2	glucose-6-phosphatase catalytic subunit 2
		Q86SF2	GALNT7	polypeptide N-acetylgalactosaminyltransferase 7
		Q02742	GCNT1	glucosaminyl (N-acetyl) transferase 1
		Q9UG22	GIMAP2	GTPase, IMAP family member 2
		Q03113	GNA12	G protein subunit alpha 12

		Q3T906	GNPTAB	N-acetylglucosamine-1-phosphate transferase subunits alpha and beta
		O00461	GOLIM4	golgi integral membrane protein 4
		Q8N3F9	GPR137C	G protein-coupled receptor 137C
		Q9NYM4	GPR83	G protein-coupled receptor 83
		P52655	GTF2A1	general transcription factor IIA subunit 1
		P33402	GUCY1A2	guanylate cyclase 1 soluble subunit alpha 2
		O14792	HS2ST1	heparan sulfate 2-O-sulfotransferase 1
		Q8IZT8	HS3ST5	heparan sulfate-glucosamine 3-sulfotransferase 5
		Q4G112	HSF5	heat shock transcription factor 5
		P11021	HSPA5	heat shock protein family A (Hsp70) member 5
		Q9ULR0	ISY1	ISY1 splicing factor homolog
		P26006	ITGA3	integrin subunit alpha 3
		P23229	ITGA6	integrin subunit alpha 6
		Q13572	ITPK1	inositol-tetrakisphosphate 1-kinase
		Q9H079	KATNBL1	katanin regulatory subunit B1 like 1
		O15550	KDM6A	lysine demethylase 6A
		Q9UH77	KLHL3	kelch like family member 3
		A6PVL3	KNCN	kinocilin
		Q6ZN17	LIN28B	lin-28 homolog B
		Q6P1M3	LLGL2	LLGL scribble cell polarity complex component 2
		Q99677	LPAR4	lysophosphatidic acid receptor 4
		P98164	LRP2	LDL receptor related protein 2
		O43300	LRRTM2	leucine rich repeat transmembrane neuronal 2
		Q9NU23	LYRM2	LYR motif containing 2
		Q99683	MAP3K5	mitogen-activated protein kinase kinase kinase 5
		P49137	MAPKAPK2	mitogen-activated protein kinase-activated protein kinase 2
		O95243	MBD4	methyl-CpG binding domain 4, DNA glycosylase

		P13995	MTHFD2	methylenetetrahydrofolate dehydrogenase (NADP+ dependent) 2, methenyltetrahydrofolate cyclohydrolase
		Q96BF6	NACC2	NACC family member 2
		Q15788	NCOA1	nuclear receptor coactivator 1
		O95139	NDUFB6	NADH:ubiquinone oxidoreductase subunit B6
		Q92692	NECTIN2	nectin cell adhesion molecule 2
		Q9UBE8	NLK	nemo like kinase
		Q9Y5X4	NR2E3	nuclear receptor subfamily 2 group E member 3
		Q02297	NRG1	neuregulin 1
		Q7Z417	NUFIP2	nuclear FMR1 interacting protein 2
		P61970	NUTF2	nuclear transport factor 2
		Q9ULI1	NWD2	NACHT and WD repeat domain containing 2
		Q9P0S3	ORMDL1	ORMDL sphingolipid biosynthesis regulator 1
		Q8N6M0	OTUD6B	OTU domain containing 6B
		O96013	PAK4	p21 (RAC1) activated kinase 4
		Q96IZ0	PAWR	pro-apoptotic WT1 regulator
		O14917	PCDH17	protocadherin 17
		O60245	PCDH7	protocadherin 7
		Q86SE9	PCGF5	polycomb group ring finger 5
		Q07343	PDE4B	phosphodiesterase 4B
		Q29RF7	PDS5A	PDS5 cohesin associated factor A
		Q96FC7	PHYHIPL	phytanoyl-CoA 2-hydroxylase interacting protein like
		P37287	PIGA	phosphatidylinositol glycan anchor biosynthesis class A
		O14495	PLPP3	phospholipid phosphatase 3
		Q12796	PNRC1	proline rich nuclear receptor coactivator 1
		Q15165	PON2	paraoxonase 2
		Q8N3J5	PPM1K	protein phosphatase, Mg2+/Mn2+ dependent 1K
		P63151	PPP2R2A	protein phosphatase 2 regulatory subunit Balpha
		Q16537	PPP2R5E	protein phosphatase 2 regulatory subunit B'epsilon

		P51817	PRKX	protein kinase X-linked
		P11464	PSG1	pregnancy specific beta-1-glycoprotein 1
		Q96BW5	PTER	phosphotriesterase related
		P26045	PTPN3	protein tyrosine phosphatase, non-receptor type 3
		Q96AH8	RABEP1	rabaptin, RAB GTPase binding effector protein 1
		Q2PPJ7	RALGAPA2	Ral GTPase activating protein catalytic alpha subunit 2
		Q86X27	RALGPS2	Ral GEF with PH domain and SH3 binding motif 2
		P10114	RAP2A	RAP2A, member of RAS oncogene family
		P06400	RB1	RB transcriptional corepressor 1
		Q06330	RBPJ	recombination signal binding protein for immunoglobulin kappa J region
		P48380	RFX3	regulatory factor X3
		Q5EBL4	RILPL1	Rab interacting lysosomal protein like 1
		O60942	RNGTT	RNA guanylyltransferase and 5'-phosphatase
		Q01973	ROCK2	Rho associated coiled-coil containing protein kinase 2
		Q9H6T3	RPAP3	RNA polymerase II associated protein 3
		Q01196	RUNX1	runt related transcription factor 1
		Q99590	SCAF11	SR-related CTD associated factor 11
		O00767	SCD	stearoyl-CoA desaturase
		P34741	SDC2	syndecan 2
		Q96JE7	SEC16B	SEC16 homolog B, endoplasmic reticulum export factor
		Q99442	SEC62	SEC62 homolog, preprotein translocation factor
		Q14563	SEMA3A	semaphorin 3A
		P07093	SERPINE2	serpin family E member 2
		P58005	SESN3	sestrin 3
		Q86VW0	SESTD1	SEC14 and spectrin domain containing 1
		Q9BYW2	SETD2	SET domain containing 2, histone lysine methyltransferase
		Q8IWX5	SGPP2	sphingosine-1-phosphate phosphatase 2
		Q9Y371	SH3GLB1	SH3 domain containing GRB2 like, endophilin B1

		Q9NP50	SINHCAF	SIN3-HDAC complex associated factor
		Q9UHW9	SLC12A6	solute carrier family 12 member 6
		Q9UI40	SLC24A2	solute carrier family 24 member 2
		Q9ULF5	SLC39A10	solute carrier family 39 member 10
		Q9Y6M7	SLC4A7	solute carrier family 4 member 7
		P30825	SLC7A1	solute carrier family 7 member 1
		Q8TCU3	SLC7A13	solute carrier family 7 member 13
		Q9H5Y7	SLITRK6	SLIT and NTRK like family member 6
		P0C7L1	SPINK8	serine peptidase inhibitor, Kazal type 8 (putative)
		O95793	STAU1	staufen double-stranded RNA binding protein 1
		Q9ULQ0	STRIP2	striatin interacting protein 2
		Q9H2B2	SYT4	synaptotagmin 4
		Q9NYJ8	TAB2	TGF-beta activated kinase 1 (MAP3K7) binding protein 2
		Q7L7X3	TAOK1	TAO kinase 1
		Q8WVE7	TMEM170A	transmembrane protein 170A
		Q0P6H9	TMEM62	transmembrane protein 62
		Q9NS68	TNFRSF19	TNF receptor superfamily member 19
		P50591	TNFSF10	TNF superfamily member 10
		O94811	TPPP	tubulin polymerization promoting protein
		P62995	TRA2B	transformer 2 beta homolog
		Q8TDR0	TRAF3IP1	TRAF3 interacting protein 1
		Q9C040	TRIM2	tripartite motif containing 2
		Q6ZMU5	TRIM72	tripartite motif containing 72
		Q15643	TRIP11	thyroid hormone receptor interactor 11
		Q8NG11	TSPAN14	tetraspanin 14
		Q9Y385	UBE2J1	ubiquitin conjugating enzyme E2 J1
		Q8N2C7	UNC80	unc-80 homolog, NALCN channel complex subunit
		Q96BW1	UPRT	uracil phosphoribosyltransferase homolog
		P14927	UQCRB	ubiquinol-cytochrome c reductase binding protein

		Q86T82	USP37	ubiquitin specific peptidase 37
		Q8IUA0	WFDC8	WAP four-disulfide core domain 8
		Q9H4A3	WNK1	WNK lysine deficient protein kinase 1
		Q9HC78	ZBTB20	zinc finger and BTB domain containing 20
		Q9P1Z0	ZBTB4	zinc finger and BTB domain containing 4
		O43298	ZBTB43	zinc finger and BTB domain containing 43
		Q96EF9	ZHX1-C8orf76	ZHX1-C8orf76 readthrough
		O75362	ZNF217	zinc finger protein 217
		Q96SE7	ZNF347	zinc finger protein 347
		Q8NDX6	ZNF740	zinc finger protein 740
		A8MXY4	ZNF99	zinc finger protein 99
		Q6PCB6	ABHD17C	Alpha/beta hydrolase domain-containing protein 17C (Abhydrolase domain-containing protein 17C) (EC 3.1.2.22)
**TargetScan**	**miR-27a-3p Upregulated 24 hours**	P62736	ACTA2	Actin, aortic smooth muscle (Alpha-actin-2) (Cell growth-inhibiting gene 46 protein) [Cleaved into: Actin, aortic smooth muscle, intermediate form]
		P29275	ADORA2B	Adenosine receptor A2b
		Q9UHB7	AFF4	AF4/FMR2 family member 4 (ALL1-fused gene from chromosome 5q31 protein) (Protein AF-5q31) (Major CDK9 elongation factor-associated protein)
		Q9H9L7	AKIRIN1	Akirin-1
		P61204	ARF3	ADP-ribosylation factor 3
		P48201	ATP5G3	ATP synthase F(0) complex subunit B1, mitochondrial (ATP synthase peripheral stalk-membrane subunit b) (ATP synthase proton-transporting mitochondrial F(0) complex subunit B1) (ATP synthase subunit b) (ATPase subunit b)
		Q9NPZ5	B3GAT2	Galactosylgalactosylxylosylprotein 3-beta-glucuronosyltransferase 2 (EC 2.4.1.135) (Beta-1,3-glucuronyltransferase 2) (GlcAT-D) (UDP-

				glucuronosyltransferase S) (GlcAT-S) (Glucuronosyltransferase S)
		Q8NFL0	B3GNT7	UDP-GlcNAc:betaGal beta-1,3-N-acetylglucosaminyltransferase 7 (BGnT-7) (Beta-1,3-Gn-T7) (Beta-1,3-N-acetylglucosaminyltransferase 7) (Beta3Gn-T7) (EC 2.4.1.-)
		O60512	B4GALT3	Beta-1,4-galactosyltransferase 3 (Beta-1,4-GalTase 3) (Beta4Gal-T3) (b4Gal-T3) (EC 2.4.1.-) (Beta-N-acetylglucosaminyl-glycolipid beta-1,4-galactosyltransferase) (Beta-N-acetylglucosaminylglycopeptide beta-1,4-galactosyltransferase) (EC 2.4.1.38) (N-acetyllactosamine synthase) (EC 2.4.1.90) (Nal synthase) (UDP-Gal:beta-GlcNAc beta-1,4-galactosyltransferase 3) (UDP-galactose:beta-N-acetylglucosamine beta-1,4-galactosyltransferase 3)
		P20749	BCL3	B-cell lymphoma 3 protein (BCL-3) (Proto-oncogene BCL3)
		P78543	BTG2	Protein BTG2 (BTG family member 2) (NGF-inducible anti-proliferative protein PC3)
		Q9NS85	CA10	Carbonic anhydrase-related protein 10 (Carbonic anhydrase-related protein X) (CA-RP X) (CARP X) (Cerebral protein 15)
		Q8IZS8	CACNA2D3	Voltage-dependent calcium channel subunit alpha-2/delta-3
		P51959	CCNG1	G1/S-specific cyclin-D1 (B-cell lymphoma 1 protein) (BCL-1) (BCL-1 oncogene) (PRAD1 oncogene)
		Q9H305	CDIP1	Cell death-inducing p53-target protein 1
		Q92903	CDS1	Phosphatidate cytidylyltransferase 1
		Q07065	CKAP4	Cytoskeleton-associated protein 4
		Q16740	CLPP	ATP-dependent Clp protease proteolytic subunit, mitochondrial
		Q15417	CNN3	Calponin-3
		Q9UIV1	CNOT7	CCR4-NOT transcription complex subunit 7

		Q8NE35	CPEB3	Cytoplasmic polyadenylation element-binding protein 3
		Q8IUR6	CREBRF	CREB3 regulatory factor
		Q92564	DCUN1D4	DCN1-like protein 4
		D3DT02	EPB41L4A	Erythrocyte membrane protein band 4.1 like 4A, isoform CRA_a
		O95677	EYA4	Eyes absent homolog 4
		Q5BKY9	FAM133B	Protein FAM133B
		Q8NB25	FAM184A	Protein FAM184A
		Q96PV7	FAM193B	Protein FAM193B
		Q96KN1	FAM84B	Protein LRATD2
		Q9UK96	FBXO10	F-box only protein 10
		Q969H0	FBXW7	F-box/WD repeat-containing protein 7 (Archipelago homolog) (hAgo) (F-box and WD-40 domain-containing protein 7) (F-box protein FBX30) (SEL-10) (hCdc4)
		Q9NZU0	FLRT3	Leucine-rich repeat transmembrane protein FLRT3 (Fibronectin-like domain-containing leucine-rich transmembrane protein 3)
		Q13480	GAB1	GRB2-associated-binding protein 1 (GRB2-associated binder 1) (Growth factor receptor bound protein 2-associated protein 1)
		Q86SF2	GALNT7	N-acetylgalactosaminyltransferase 7 (EC 2.4.1.41) (Polypeptide GalNAc transferase 7) (GalNAc-T7) (pp-GaNTase 7) (Protein-UDP acetylgalactosaminyltransferase 7) (UDP-GalNAc:polypeptide N-acetylgalactosaminyltransferase 7)
		O43716	GATC	Glutamyl-tRNA(Gln) amidotransferase subunit C, mitochondrial (Glu-AdT subunit C) (EC 6.3.5.-) (Protein 15E1.2)
		Q8IWJ2	GCC2	GRIP and coiled-coil domain-containing protein 2 (185 kDa Golgi coiled-coil protein) (GCC185) (CLL-associated antigen KW-11) (CTCL tumor antigen se1-1) (Ran-binding protein 2-like 4) (RanBP2L4) (Renal carcinoma antigen NY-REN-53)

		O94808	GFPT2	Glutamine--fructose-6-phosphate aminotransferase [isomerizing] 2 (EC 2.6.1.16) (D-fructose-6-phosphate amidotransferase 2) (Glutamine:fructose-6-phosphate amidotransferase 2) (GFAT 2) (GFAT2) (Hexosephosphate aminotransferase 2)
		P15586	GNS	N-acetylglucosamine-6-sulfatase (EC 3.1.6.14) (Glucosamine-6-sulfatase) (G6S)
		Q8NBJ4	GOLM1	Golgi membrane protein 1 (Golgi membrane protein GP73) (Golgi phosphoprotein 2)
		O14653	GOSR2	Golgi SNAP receptor complex member 2 (27 kDa Golgi SNARE protein) (Membrin)
		Q9HCL2	GPAM	Glycerol-3-phosphate acyltransferase 1, mitochondrial (GPAT-1) (EC 2.3.1.15)
		Q9NWQ4	GPATCH2L	G patch domain-containing protein 2-like
		P62993	GRB2	Growth factor receptor-bound protein 2 (Adapter protein GRB2) (Protein Ash) (SH2/SH3 adapter GRB2)
		O15399	GRIN2D	Glutamate receptor ionotropic, NMDA 2D (GluN2D) (EB11) (Glutamate [NMDA] receptor subunit epsilon-4) (N-methyl D-aspartate receptor subtype 2D) (NMDAR2D) (NR2D)
		Q4G148	GXYLT1	Glucoside xylosyltransferase 1 (EC 2.4.2.42) (Glycosyltransferase 8 domain-containing protein 3)
		Q99075	HBEGF	Proheparin-binding EGF-like growth factor [Cleaved into: Heparin-binding EGF-like growth factor (HB-EGF) (HBEGF) (Diphtheria toxin receptor) (DT-R)]
		P31260	HOXA10	Homeobox protein Hox-A10 (Homeobox protein Hox-1.8) (Homeobox protein Hox-1H) (PL)
		P20719	HOXA5	Homeobox protein Hox-A5 (Homeobox protein Hox-1C)
		P17481	HOXB8	Homeobox protein Hox-B8 (Homeobox protein Hox-2.4) (Homeobox protein Hox-2D)

		Q02535	ID3	DNA-binding protein inhibitor ID-3 (Class B basic helix-loop-helix protein 25) (bHLHb25) (Helix-loop-helix protein HEIR-1) (ID-like protein inhibitor HLH 1R21) (Inhibitor of DNA binding 3) (Inhibitor of differentiation 3)
		Q8WYH8	ING5	Inhibitor of growth protein 5 (p28ING5)
		Q9H8E8	KAT14	Potassium voltage-gated channel subfamily A member 4 (HPCN2) (Voltage-gated K(+) channel HuKII) (Voltage-gated potassium channel HBK4) (Voltage-gated potassium channel HK1) (Voltage-gated potassium channel subunit Kv1.4)
		P22459	KCNA4	Inward rectifier potassium channel 2 (Cardiac inward rectifier potassium channel) (Inward rectifier K(+) channel Kir2.1) (IRK-1) (hIRK1) (Potassium channel, inwardly rectifying subfamily J member 2)
		Q2LD37	KIAA1109	Chromosome-associated kinesin KIF4A (Chromokinesin-A)
		P21583	KITLG	GTPase KRas (K-Ras 2) (Ki-Ras) (c-K-ras) (c-Ki-ras) [Cleaved into: GTPase KRas, N-terminally processed]
		P53667	LIMK1	LIM domain kinase 1 (LIMK-1) (EC 2.7.11.1)
		Q17RB8	LONRF1	LON peptidase N-terminal domain and RING finger protein 1 (RING finger protein 191)
		Q16539	MAPK14	Mitogen-activated protein kinase 14 (MAP kinase 14) (MAPK 14) (EC 2.7.11.24) (Cytokine suppressive anti-inflammatory drug-binding protein) (CSAID-binding protein) (CSBP) (MAP kinase MXI2) (MAX-interacting protein 2) (Mitogen-activated protein kinase p38 alpha) (MAP kinase p38 alpha) (Stress-activated protein kinase 2a) (SAPK2a)
		O15232	MATN3	Matrilin-3
		Q8NA29	MFSD2A	Sodium-dependent lysophosphatidylcholine symporter 1 (NLS1) (Sodium-dependent LPC symporter 1) (Major facilitator superfamily domain-containing protein 2A)

		Q7Z3K6	MIER3	Mesoderm induction early response protein 3 (Mi-er3)
		Q15546	MMD	Monocyte to macrophage differentiation factor (Progestin and adipoQ receptor family member 11) (Progestin and adipoQ receptor family member XI)
		Q96AH0	NABP1	SOSS complex subunit B2 (Nucleic acid-binding protein 1) (Oligonucleotide/oligosaccharide-binding fold-containing protein 2A) (Sensor of single-strand DNA complex subunit B2) (Sensor of ssDNA subunit B2) (SOSS-B2) (Single-stranded DNA-binding protein 2) (hSSB2)
		Q8NI08	NCOA7	Nuclear receptor coactivator 7 (140 kDa estrogen receptor-associated protein) (Estrogen nuclear receptor coactivator 1)
		P46934	NEDD4	E3 ubiquitin-protein ligase NEDD4 (EC 2.3.2.26) (Cell proliferation-inducing gene 53 protein) (HECT-type E3 ubiquitin transferase NEDD4) (Neural precursor cell expressed developmentally down-regulated protein 4) (NEDD-4)
		Q8TDF5	NETO1	Neuropilin and tolloid-like protein 1
		Q14991	NGFRAP1	NGFRAP1
		P51513	NOVA1	RNA-binding protein Nova-1
		P24468	NR2F2	COUP transcription factor 2
		P10588	NR2F6	Nuclear receptor subfamily 2 group F member 6 (V-erbA-related protein 2) (EAR-2)
		O60462	NRP2	Neuropilin-2 (Vascular endothelial cell growth factor 165 receptor 2)
		O95948	ONECUT2	One cut domain family member 2 (Hepatocyte nuclear factor 6-beta) (HNF-6-beta) (One cut homeobox 2) (Transcription factor ONECUT-2) (OC-2)
		Q9BPZ3	PAIP2	Polyadenylate-binding protein-interacting protein 2 (PABP-interacting protein 2) (PAIP-2) (Poly(A)-binding protein-interacting protein 2)

		Q8TE04	PANK1	Pantothenate kinase 1
		Q9HC56	PCDH9	Protocadherin-9
		Q9NP56	PDE7B	cAMP-specific 3',5'-cyclic phosphodiesterase 7B
		Q14554	PDIA5	Protein disulfide-isomerase A5
		Q9NTI5	PDS5B	Sister chromatid cohesion protein PDS5 homolog B
		P35232	PHB	Prohibitin
		P61925	PKIA	cAMP-dependent protein kinase inhibitor alpha
		Q9UPR0	PLCL2	Inactive phospholipase C-like protein 2
		Q9ULM0	PLEKHH1	Pleckstrin homology domain-containing family H member 1
		Q9NYY3	PLK2	Serine/threonine-protein kinase PLK2
		Q8NBL1	POGLUT1	Protein O-glucosyltransferase 1
		P37231	PPARG	Peroxisome proliferator-activated receptor gamma
		P30405	PPIF	Peptidyl-prolyl cis-trans isomerase F, mitochondrial
		Q9NY27	PPP4R2	Serine/threonine-protein phosphatase 4 regulatory subunit 2
		P51817	PRKX	cAMP-dependent protein kinase catalytic subunit PRKX
		P79522	PRR3	Proline-rich protein 3
		Q8WXF1	PSPC1	Paraspeckle component 1
		P49023	PXN	Paxillin
		Q9UJF2	RASAL2	Ras GTPase-activating protein nGAP
		Q6ZRY4	RBPMS2	RNA-binding protein with multiple splicing 2
		Q14206	RCAN2	Calcipressin-2
		P78509	RELN	Reelin
		Q99666	RGPD5	RANBP2-like and GRIP domain-containing protein 5/6
		Q9UGC6	RGS17	Regulator of G-protein signaling 17
		Q9Y4F9	RIPOR2	Rho family-interacting cell polarization regulator 2
		Q8WU17	RNF139	E3 ubiquitin-protein ligase RNF139
		Q01973	ROR1	Inactive tyrosine-protein kinase transmembrane receptor ROR1
		O75582	RPS6KA5	Ribosomal protein S6 kinase alpha-5
		Q92766	RREB1	Ras-responsive element-binding protein 1

		Q01196	RUNX1	Runt-related transcription factor 1
		Q96IW7	SEC22A	Vesicle-trafficking protein SEC22a
		Q96NB2	SFXN2	Sideroflexin-2
		Q8TF71	SLC16A10	Monocarboxylate transporter 10
		Q8N1S5	SLC39A11	Zinc transporter ZIP11
		P30531	SLC6A1	Sodium- and chloride-dependent GABA transporter 1
		Q96PX8	SLITRK1	SLIT and NTRK-like protein 1
		O15198	SMAD9	Mothers against decapentaplegic homolog 9
		P60880	SNAP25	Synaptosomal-associated protein 25
		Q9UMY4	SNX12	Sorting nexin-12
		O43597	SPRY2	Protein sprouty homolog 2
		P12931	SRC	Proto-oncogene tyrosine-protein kinase Src (EC 2.7.10.2) (Proto-oncogene c-Src) (pp60c-src) (p60-Src)
		Q8NDV1	ST6GALNAC3	Alpha-N-acetylgalactosaminide alpha-2,6-sialyltransferase 5 (EC 2.4.99.-) (GD1 alpha synthase) (GalNAc alpha-2,6-sialyltransferase V) (ST6GalNAc V) (ST6GalNAcV) (Sialyltransferase 7E) (SIAT7-E)
		Q8WWQ8	STAB2	Stabilin-2
		Q7Z422	SZRD1	SUZ domain-containing protein 1
		Q96FV9	THOC1	THO complex subunit 1 (Tho1) (Nuclear matrix protein p84) (p84N5) (hTREX84)
		O94876	TMCC1	Transmembrane and coiled-coil domains protein 1
		P63313	TMSB10	Thymosin beta-10
		Q92973	TNPO1	Transportin-1 (Importin beta-2) (Karyopherin beta-2) (M9 region interaction protein) (MIP)
		O15417	TNRC18	Trinucleotide repeat-containing gene 18 protein (Long CAG trinucleotide repeat-containing gene 79 protein)

		P36406	TRIM23	E3 ubiquitin-protein ligase TRIM23 (EC 2.3.2.27) (ADP-ribosylation factor domain-containing protein 1) (GTP-binding protein ARD-1) (RING finger protein 46) (RING-type E3 ubiquitin transferase TRIM23) (Tripartite motif-containing protein 23)
		Q16222	UAP1	UDP-N-acetylhexosamine pyrophosphorylase (Antigen X) (AGX) (Sperm-associated antigen 2) [Includes: UDP-N-acetylgalactosamine pyrophosphorylase (EC 2.7.7.83) (AGX-1); UDP-N-acetylglucosamine pyrophosphorylase (EC 2.7.7.23) (AGX-2)]
		P51668	UBE2D1	Ubiquitin-conjugating enzyme E2 D1 (EC 2.3.2.23) ((E3-independent) E2 ubiquitin-conjugating enzyme D1) (EC 2.3.2.24) (E2 ubiquitin-conjugating enzyme D1) (Stimulator of Fe transport) (SFT) (UBC4/5 homolog) (UbcH5) (Ubiquitin carrier protein D1) (Ubiquitin-conjugating enzyme E2(17)KB 1) (Ubiquitin-conjugating enzyme E2-17 kDa 1) (Ubiquitin-protein ligase D1)
		Q5JXB2	UBE2NL	Putative ubiquitin-conjugating enzyme E2 N-like (Epididymis tissue protein Li 174)
		Q13404	UBE2V1	Ubiquitin-conjugating enzyme E2 variant 1 (UEV-1) (CROC-1) (TRAF6-regulated IKK activator 1 beta Uev1A)
		Q9H9P5	UNKL	Putative E3 ubiquitin-protein ligase UNKL (EC 2.3.2.-) (RING finger protein unkempt-like) (Zinc finger CCCH domain-containing protein 5-like)
		P62068	USP46	Ubiquitin carboxyl-terminal hydrolase 46 (EC 3.4.19.12) (Deubiquitinating enzyme 46) (Ubiquitin thioesterase 46) (Ubiquitin-specific-processing protease 46)
		P49767	VEGFC	Vascular endothelial growth factor C (VEGF-C) (Flt4 ligand) (Flt4-L) (Vascular endothelial growth factor-related protein) (VRP)

		B2RUY7	VWC2L	von Willebrand factor C domain-containing protein 2-like (Brorin-like)
		Q8NCN2	ZBTB34	Zinc finger and BTB domain-containing protein 34
		Q5TC79	ZBTB37	Zinc finger and BTB domain-containing protein 37
		Q9H6B1	ZNF385D	Zinc finger protein 385D (Zinc finger protein 659)
		Q9ULD9	ZNF608	Zinc finger protein 608 (Renal carcinoma antigen NY-REN-36)
		O95477	ABCA1	ATP binding cassette subfamily A member 1
**miRDB**		Q9NP78	ABCB9	ATP binding cassette subfamily B member 9
		Q5VST6	ABHD17B	abhydrolase domain containing 17B
		Q6PCB6	ABHD17C	abhydrolase domain containing 17C
		Q9BV23	ABHD6	abhydrolase domain containing 6
		Q9NYB9	ABL2	ABL proto-oncogene 2, non-receptor tyrosine kinase
		P62736	ACTA2	actin, alpha 2, smooth muscle, aorta
		Q8NER5	ACVR1C	activin A receptor type 1C
		Q9H324	ADAMTS10	ADAM metallopeptidase with thrombospondin type 1 motif 10
		Q8N6G6	ADAMTSL1	ADAMTS like 1
		P82987	ADAMTSL3	ADAMTS like 3
		O43306	ADCY6	adenylate cyclase 6
		Q8IWK6	ADGRA3	adhesion G protein-coupled receptor A3
		P29275	ADORA2B	adenosine A2b receptor
		Q8N556	AFAP1	actin filament associated protein 1
		Q9UHB7	AFF4	AF4/FMR2 family member 4
		P52594	AGFG1	ArfGAP with FG repeats 1
		O00468	AGRN	agrin
		Q719I0	AHSA2P	activator of HSP90 ATPase homolog 2, pseudogene
		P27144	AK4	adenylate kinase 4
		Q9H9L7	AKIRIN1	akirin 1
		Q53H80	AKIRIN2	akirin 2
		P51649	ALDH5A1	aldehyde dehydrogenase 5 family member A1

		Q9H6U8	ALG9	ALG9, alpha-1,2-mannosyltransferase
		P17707	AMD1	adenosylmethionine decarboxylase 1
		Q01432	AMPD3	adenosine monophosphate deaminase 3
		P16157	ANK1	ankyrin 1
		Q92625	ANKS1A	ankyrin repeat and sterile alpha motif domain containing 1A
		Q6UXV4	APOOL	apolipoprotein O like
		Q92624	APPBP2	amyloid beta precursor protein binding protein 2
		Q8NBQ7	AQP11	aquaporin 11
		P61204	ARF3	ADP ribosylation factor 3
		Q9Y6D6	ARFGEF1	ADP ribosylation factor guanine nucleotide exchange factor 1
		A7KAX9	ARHGAP32	Rho GTPase activating protein 32
		Q96DR7	ARHGEF26	Rho guanine nucleotide exchange factor 26
		Q14155	ARHGEF7	Rho guanine nucleotide exchange factor 7
		P56559	ARL4C	ADP ribosylation factor like GTPase 4C
		Q8IUR7	ARMC8	armadillo repeat containing 8
		Q8NCT1	ARRDC4	arrestin domain containing 4
		Q96QS3	ARX	aristaless related homeobox
		Q13510	ASAH1	N-acylsphingosine amidohydrolase 1
		Q9ULH1	ASPH	aspartate beta-hydroxylase
		Q8NBU5	ATAD2B	ATPase family, AAA domain containing 2B
		Q8NB49	ATP11C	ATPase phospholipid transporting 11C
		P50993	ATP1A2	ATPase Na+/K+ transporting subunit alpha 2
		P20020	ATP2B1	ATPase plasma membrane Ca2+ transporting 1
		Q8NFL0	B3GNT7	UDP-GlcNAc:betaGal beta-1,3-N-acetylglucosaminyltransferase 7
		O60512	B4GALT3	beta-1,4-galactosyltransferase 3
		Q6ZU67	BEND4	BEN domain containing 4
		O76090	BEST1	bestrophin 1
		Q00994	BEX3	brain expressed X-linked 3

		Q9H694	BICC1	BicC family RNA binding protein 1
		P12645	BMP3	bone morphogenetic protein 3
		Q12982	BNIP2	BCL2 interacting protein 2
		Q12983	BNIP3	BCL2 interacting protein 3
		Q9ULD4	BRPF3	bromodomain and PHD finger containing 3
		Q6RI45	BRWD3	bromodomain and WD repeat domain containing 3
		P62324	BTG1	BTG anti-proliferation factor 1
		P78543	BTG2	BTG anti-proliferation factor 2
		O00193	C11orf58	chromosome 11 open reading frame 58
		Q8N6N3	C1orf52	chromosome 1 open reading frame 52
		Q5TEA3	C20orf194	chromosome 20 open reading frame 194
		Q9Y426	C2CD2	C2 calcium dependent domain containing 2
		Q9NS85	CA10	carbonic anhydrase 10
		Q8IZS8	CACNA2D3	calcium voltage-gated channel auxiliary subunit alpha2delta 3
		Q9Y698	CACNG2	calcium voltage-gated channel auxiliary subunit gamma 2
		Q05682	CALD1	caldesmon 1
		Q8IU85	CAMK1D	calcium/calmodulin dependent protein kinase ID
		Q9Y6Y1	CAMTA1	calmodulin binding transcription activator 1
		Q86VP6	CAND1	cullin associated and neddylation dissociated 1
		O75808	CAPN15	calpain 15
		O75081	CBFA2T3	CBFA2/RUNX1 translocation partner 3
		Q13191	CBLB	Cbl proto-oncogene B
		Q9BUN5	CCDC28B	coiled-coil domain containing 28B
		Q9BSQ5	CCM2	CCM2 scaffold protein
		O95388	CCN4	cellular communication network factor 4
		P24863	CCNC	cyclin C
		P51959	CCNG1	cyclin G1
		Q5T5M9	CCNJ	cyclin J
		O75909	CCNK	cyclin K

		O60583	CCNT2	cyclin T2
		Q8ND76	CCNY	cyclin Y
		Q9UKI2	CDC42EP3	CDC42 effector protein 3
		P55287	CDH11	cadherin 11
		P33151	CDH5	cadherin 5
		Q9H305	CDIP1	cell death inducing p53 target 1
		Q07002	CDK18	cyclin dependent kinase 18
		Q01850	CDR2	cerebellar degeneration related protein 2
		Q92903	CDS1	CDP-diacylglycerol synthase 1
		Q9BXF3	CECR2	CECR2, histone acetyl-lysine reader
		O95319	CELF2	CUGBP Elav-like family member 2
		Q8WUJ3	CEMIP	cell migration inducing hyaluronidase 1
		Q66GS9	CEP135	centrosomal protein 135
		O14647	CHD2	chromodomain helicase DNA binding protein 2
		Q9P2D1	CHD7	chromodomain helicase DNA binding protein 7
		P35790	CHKA	choline kinase alpha
		O00533	CHL1	cell adhesion molecule L1 like
		Q9C0C6	CIPC	CLOCK interacting pacemaker
		Q07065	CKAP4	cytoskeleton associated protein 4
		P51790	CLCN3	chloride voltage-gated channel 3
		P51795	CLCN5	chloride voltage-gated channel 5
		P49760	CLK2	CDC like kinase 2
		Q9H4D0	CLSTN2	calsyntenin 2
		Q99788	CMKLR1	chemerin chemokine-like receptor 1
		Q15417	CNN3	calponin 3
		P83436	COG7	component of oligomeric golgi complex 7
		Q14993	COL19A1	collagen type XIX alpha 1 chain
		Q96P44	COL21A1	collagen type XXI alpha 1 chain
		Q9Y6Z7	COLEC10	collectin subfamily member 10

		Q8IYK4	COLGALT2	collagen beta(1-O)galactosyltransferase 2
		P61923	COPZ1	coatomer protein complex subunit zeta 1
		Q9BRF8	CPPED1	calcineurin like phosphoesterase domain containing 1
		P16220	CREB1	cAMP responsive element binding protein 1
		Q8IUR6	CREBRF	CREB3 regulatory factor
		P09603	CSF1	colony stimulating factor 1
		Q16527	CSRP2	cysteine and glycine rich protein 2
		P32929	CTH	cystathionine gamma-lyase
		Q7Z7A3	CTU1	cytosolic thiouridylase subunit 1
		Q9NYL5	CYP39A1	cytochrome P450 family 39 subfamily A member 1
		Q5T6F0	DCAF12	DDB1 and CUL4 associated factor 12
		P61962	DCAF7	DDB1 and CUL4 associated factor 7
		Q9NPI6	DCP1A	decapping mRNA 1A
		Q92564	DCUN1D4	defective in cullin neddylation 1 domain containing 4
		P17844	DDX5	DEAD-box helicase 5
		Q8N2C3	DEPDC4	DEP domain containing 4
		Q5T7M9	DIPK1A	divergent protein kinase domain 1A
		Q9UBU2	DKK2	dickkopf WNT signaling pathway inhibitor 2
		Q9NR61	DLL4	delta like canonical Notch ligand 4
		O75165	DNAJC13	DnaJ heat shock protein family (Hsp40) member C13
		Q9NZQ0	DNAJC27	DnaJ heat shock protein family (Hsp40) member C27
		Q8TEK3	DOT1L	DOT1 like histone lysine methyltransferase
		Q9Y4J8	DTNA	dystrobrevin alpha
		Q8TCX1	DYNC2LI1	dynein cytoplasmic 2 light intermediate chain 1
		Q96FJ2	DYNLL2	dynein light chain LC8-type 2
		Q13627	DYRK1A	dual specificity tyrosine phosphorylation regulated kinase 1A
		Q96AV8	E2F7	E2F transcription factor 7
		P0DPD6	ECE2	endothelin converting enzyme 2
		Q9UNE0	EDAR	ectodysplasin A receptor

		Q9BZQ6	EDEM3	ER degradation enhancing alpha-mannosidase like protein 3
		Q3B7T1	EDRF1	erythroid differentiation regulatory factor 1
		P0DPD8	EEF1AKMT4-ECE2	EEF1AKMT4-ECE2 readthrough
		P52799	EFNB2	ephrin B2
		P00533	EGFR	epidermal growth factor receptor
		Q9NZC4	EHF	ETS homologous factor
		Q9GZV4	EIF5A2	eukaryotic translation initiation factor 5A2
		Q12926	ELAVL2	ELAV like RNA binding protein 2
		Q5R3F8	ELFN2	extracellular leucine rich repeat and fibronectin type III domain containing 2
		P19622	EN2	engrailed homeobox 2
		P21128	ENDOU	endonuclease, poly(U) specific
		P11171	EPB41	erythrocyte membrane protein band 4.1
		Q9HCS5	EPB41L4A	erythrocyte membrane protein band 4.1 like 4A
		Q12929	EPS8	epidermal growth factor receptor pathway substrate 8
		P11308	ERG	ETS transcription factor ERG
		Q5RHP9	ERICH3	glutamate rich 3
		Q99502	EYA1	EYA transcriptional coactivator and phosphatase 1
		O95677	EYA4	EYA transcriptional coactivator and phosphatase 4
		Q5T9C2	FAM102A	family with sequence similarity 102 member A
		Q9BYI3	FAM126A	family with sequence similarity 126 member A
		Q5BKY9	FAM133B	family with sequence similarity 133 member B
		Q5VUB5	FAM171A1	family with sequence similarity 171 member A1
		Q8NB25	FAM184A	family with sequence similarity 184 member A
		Q9NX38	FAM206A	family with sequence similarity 206 member A
		Q9NTX9	FAM217B	family with sequence similarity 217 member B
		Q5HYJ3	FAM76B	family with sequence similarity 76 member B
		Q5JUQ0	FAM78A	family with sequence similarity 78 member A

		Q9UBX5	FBLN5	fibulin 5
		Q9UK96	FBXO10	F-box protein 10
		Q9NWN3	FBXO34	F-box protein 34
		P0C2W1	FBXO45	F-box protein 45
		Q969H0	FBXW7	F-box and WD repeat domain containing 7
		Q96LA6	FCRL1	Fc receptor like 1
		Q96LA5	FCRL2	Fc receptor like 2
		Q6ZV73	FGD6	FYVE, RhoGEF and PH domain containing 6
		P21781	FGF7	fibroblast growth factor 7
		Q9NZU0	FLRT3	fibronectin leucine rich transmembrane protein 3
		P02751	FN1	fibronectin 1
		P53539	FOSB	FosB proto-oncogene, AP-1 transcription factor subunit
		P55318	FOXA3	forkhead box A3
		Q12778	FOXO1	forkhead box O1
		O43524	FOXO3	forkhead box O3
		Q8IVH2	FOXP4	forkhead box P4
		Q96NE9	FRMD6	FERM domain containing 6
		O43559	FRS3	fibroblast growth factor receptor substrate 3
		Q96I24	FUBP3	far upstream element binding protein 3
		Q9ULV1	FZD4	frizzled class receptor 4
		Q13480	GAB1	GRB2 associated binding protein 1
		Q14435	GALNT3	polypeptide N-acetylgalactosaminyltransferase 3
		Q86SF2	GALNT7	polypeptide N-acetylgalactosaminyltransferase 7
		Q9H706	GAREM1	GRB2 associated regulator of MAPK1 subtype 1
		P23769	GATA2	GATA binding protein 2
		P23771	GATA3	GATA binding protein 3
		Q92908	GATA6	GATA binding protein 6
		Q86YP4	GATAD2A	GATA zinc finger domain containing 2A
		O43716	GATC	glutamyl-tRNA amidotransferase subunit C

		Q8IWJ2	GCC2	GRIP and coiled-coil domain containing 2
		P55040	GEM	GTP binding protein overexpressed in skeletal muscle
		O94808	GFPT2	glutamine-fructose-6-phosphate transaminase 2
		P23416	GLRA2	glycine receptor alpha 2
		Q9NZD2	GLTP	glycolipid transfer protein
		Q9UBI6	GNG12	G protein subunit gamma 12
		P15586	GNS	glucosamine (N-acetyl)-6-sulfatase
		Q92805	GOLGA1	golgin A1
		Q8NBJ4	GOLM1	golgi membrane protein 1
		Q9HCL2	GPAM	glycerol-3-phosphate acyltransferase, mitochondrial
		P43304	GPD2	glycerol-3-phosphate dehydrogenase 2
		P46095	GPR6	G protein-coupled receptor 6
		Q8TD30	GPT2	glutamic--pyruvic transaminase 2
		Q3KR37	GRAMD1B	GRAM domain containing 1B
		P62993	GRB2	growth factor receptor bound protein 2
		P42263	GRIA3	glutamate ionotropic receptor AMPA type subunit 3
		P48058	GRIA4	glutamate ionotropic receptor AMPA type subunit 4
		O15399	GRIN2D	glutamate ionotropic receptor NMDA type subunit 2D
		P41594	GRM5	glutamate metabotropic receptor 5
		P15170	GSPT1	G1 to S phase transition 1
		Q4G148	GXYLT1	glucoside xylosyltransferase 1
		P10915	HAPLN1	hyaluronan and proteoglycan link protein 1
		Q99075	HBEGF	heparin binding EGF like growth factor
		Q9H0R4	HDHD2	haloacid dehalogenase like hydrolase domain containing 2
		Q9ULI3	HEG1	heart development protein with EGF like domains 1
		Q14526	HIC1	HIC ZBTB transcriptional repressor 1
		O00291	HIP1	huntingtin interacting protein 1
		Q9H2X6	HIPK2	homeodomain interacting protein kinase 2

		Q5T1R4	HIVEP3	human immunodeficiency virus type I enhancer binding protein 3
		P04035	HMGCR	3-hydroxy-3-methylglutaryl-CoA reductase
		P31260	HOXA10	homeobox A10
		P20719	HOXA5	homeobox A5
		P09630	HOXC6	homeobox C6
		Q7LGA3	HS2ST1	heparan sulfate 2-O-sulfotransferase 1
		Q53GQ0	HSD17B12	hydroxysteroid 17-beta dehydrogenase 12
		Q3SXM5	HSDL1	hydroxysteroid dehydrogenase like 1
		P10809	HSPD1	heat shock protein family D (Hsp60) member 1
		Q92598	HSPH1	heat shock protein family H (Hsp110) member 1
		Q9Y4L1	HYOU1	hypoxia up-regulated 1
		P47928	ID4	inhibitor of DNA binding 4, HLH protein
		Q9UKS7	IKZF2	IKAROS family zinc finger 2
		Q16352	INA	internexin neuronal intermediate filament protein alpha
		Q8WYH8	ING5	inhibitor of growth family member 5
		Q53TQ3	INO80D	INO80 complex subunit D
		P49441	INPP1	inositol polyphosphate-1-phosphatase
		Q15735	INPP5J	inositol polyphosphate-5-phosphatase J
		Q96T92	INSM2	INSM transcriptional repressor 2
		P06213	INSR	insulin receptor
		Q8NFU5	IPMK	inositol polyphosphate multikinase
		Q15306	IRF4	interferon regulatory factor 4
		P61371	ISL1	ISL LIM homeobox 1
		P08648	ITGA5	integrin subunit alpha 5
		P53708	ITGA8	integrin subunit alpha 8
		Q9NZM3	ITSN2	intersectin 2
		Q92831	KAT2B	lysine acetyltransferase 2B
		Q8NFY9	KBTBD8	kelch repeat and BTB domain containing 8

		Q9P0J7	KCMF1	potassium channel modulatory factor 1
		P22459	KCNA4	potassium voltage-gated channel subfamily A member 4
		P48549	KCNJ3	potassium voltage-gated channel subfamily J member 3
		O95069	KCNK2	potassium two pore domain channel subfamily K member 2
		Q13303	KCNK5	potassium two pore domain channel subfamily K member 5
		O95279	KCNN3	potassium calcium-activated channel subfamily N member 3
		Q9BQ13	KCTD14	potassium channel tetramerization domain containing 14
		Q6ZWB6	KCTD8	potassium channel tetramerization domain containing 8
		Q9Y4C1	KDM3A	lysine demethylase 3A
		Q6ZMT4	KDM7A	lysine demethylase 7A
		Q92945	KHSRP	KH-type splicing regulatory protein
		Q8IZA0	KIAA0319L	KIAA0319 like
		Q2LD37	KIAA1109	KIAA1109
		Q6NV74	KIAA1211L	KIAA1211 like
		P21583	KITLG	KIT ligand
		P57682	KLF3	Kruppel like factor 3
		Q96CT2	KLHL29	kelch like family member 29
		Q9H511	KLHL31	kelch like family member 31
		Q8NEZ4	KMT2C	lysine methyltransferase 2C
		Q9NQR1	KMT5A	lysine methyltransferase 5A
		Q4FZB7	KMT5B	lysine methyltransferase 5B
		O00505	KPNA3	karyopherin subunit alpha 3
		Q14974	KPNB1	karyopherin subunit beta 1
		Q71RC2	LARP4	La ribonucleoprotein domain family member 4
		Q96JN0	LCOR	ligand dependent nuclear receptor corepressor
		P42702	LIFR	LIF receptor alpha
		P53667	LIMK1	LIM domain kinase 1
		A6NK58	LIPT2	lipoyl(octanoyl) transferase 2
		Q99732	LITAF	lipopolysaccharide induced TNF factor

		Q86WA8	LONP2	lon peptidase 2, peroxisomal
		Q17RB8	LONRF1	LON peptidase N-terminal domain and ring finger 1
		P28300	LOX	lysyl oxidase
		P43657	LPAR6	lysophosphatidic acid receptor 6
		Q8NF37	LPCAT1	lysophosphatidylcholine acyltransferase 1
		Q14693	LPIN1	lipin 1
		Q7Z3D4	LYSMD3	LysM domain containing 3
		Q5TCQ9	MAGI3	membrane associated guanylate kinase, WW and PDZ domain containing 3
		P46821	MAP1B	microtubule associated protein 1B
		P45985	MAP2K4	mitogen-activated protein kinase kinase 4
		O14733	MAP2K7	mitogen-activated protein kinase kinase 7
		Q9Y6R4	MAP3K4	mitogen-activated protein kinase kinase kinase 4
		Q16539	MAPK14	mitogen-activated protein kinase 14
		Q5VT66	MARC1	mitochondrial amidoxime reducing component 1
		Q9P0L2	MARK1	microtubule affinity regulating kinase 1
		O15232	MATN3	matrilin 3
		Q05BQ5	MBTD1	mbt domain containing 1
		O15151	MDM4	MDM4, p53 regulator
		Q9NU22	MDN1	midasin AAA ATPase 1
		Q86YW9	MED12L	mediator complex subunit 12 like
		O60244	MED14	mediator complex subunit 14
		Q9UM21	MGAT4A	alpha-1,3-mannosyl-glycoprotein 4-beta-N-acetylglucosaminyltransferase A
		Q8N344	MIER2	MIER family member 2
		Q7Z3K6	MIER3	MIER family member 3
		Q7L4E1	MIGA2	mitoguardin 2
		Q9UL63	MKLN1	muskelin 1
		Q9HBH9	MKNK2	MAP kinase interacting serine/threonine kinase 2

		Q15546	MMD	monocyte to macrophage differentiation associated
		O95396	MOCS3	molybdenum cofactor synthesis 3
		O60783	MRPS14	mitochondrial ribosomal protein S14
		Q9GZW8	MS4A7	membrane spanning 4-domains A7
		Q68DK7	MSL1	MSL complex subunit 1
		Q9NYA4	MTMR4	myotubularin related protein 4
		Q8N3F0	MTURN	maturin, neural progenitor differentiation regulator homolog
		P35580	MYH10	myosin heavy chain 10
		Q01538	MYT1	myelin transcription factor 1
		Q96AH0	NABP1	nucleic acid binding protein 1
		Q8IVL1	NAV2	neuron navigator 2
		P61601	NCALD	neurocalcin delta
		P13591	NCAM1	neural cell adhesion molecule 1
		Q9HCD5	NCOA5	nuclear receptor coactivator 5
		Q8NI08	NCOA7	nuclear receptor coactivator 7
		E9PQ53	NDUFC2-KCTD14	NDUFC2-KCTD14 readthrough
		O43181	NDUFS4	NADH:ubiquinone oxidoreductase subunit S4
		Q8N987	NECAB1	N-terminal EF-hand calcium binding protein 1
		Q8NC96	NECAP1	NECAP endocytosis associated 1
		P46934	NEDD4	neural precursor cell expressed, developmentally down-regulated 4, E3 ubiquitin protein ligase
		Q9HC98	NEK6	NIMA related kinase 6
		A6NFY4	NEMP2	nuclear envelope integral membrane protein 2
		Q8TDF5	NETO1	neuropilin and tolloid like 1
		A8MQ27	NEURL1B	neuralized E3 ubiquitin protein ligase 1B
		Q96JN8	NEURL4	neuralized E3 ubiquitin protein ligase 4
		P21359	NF1	neurofibromin 1
		P08138	NGFR	nerve growth factor receptor
		Q02577	NHLH2	nescient helix-loop-helix 2

		Q0D2K0	NIPAL4	NIPA like domain containing 4
		Q4KMZ8	NKAIN1	sodium/potassium transporting ATPase interacting 1
		P51513	NOVA1	NOVA alternative splicing regulator 1
		Q8IXF0	NPAS3	neuronal PAS domain protein 3
		P55786	NPEPPS	aminopeptidase puromycin sensitive
		Q9Y639	NPTN	neuroplastin
		Q14995	NR1D2	nuclear receptor subfamily 1 group D member 2
		P49116	NR2C2	nuclear receptor subfamily 2 group C member 2
		P24468	NR2F2	nuclear receptor subfamily 2 group F member 2
		P10588	NR2F6	nuclear receptor subfamily 2 group F member 6
		P04150	NR3C1	nuclear receptor subfamily 3 group C member 1
		O00482	NR5A2	nuclear receptor subfamily 5 group A member 2
		Q7Z6K4	NRARP	NOTCH regulated ankyrin repeat protein
		Q96F24	NRBF2	nuclear receptor binding factor 2
		Q16612	NREP	neuronal regeneration related protein
		Q7Z2Y5	NRK	Nik related kinase
		O60462	NRP2	neuropilin 2
		Q96L73	NSD1	nuclear receptor binding SET domain protein 1
		Q8TEM1	NUP210	nucleoporin 210
		Q9UBU9	NXF1	nuclear RNA export factor 1
		Q9NPJ8	NXT2	nuclear transport factor 2 like export factor 2
		Q86UD1	OAF	out at first homolog
		O60502	OGA	O-GlcNAcase
		O95948	ONECUT2	one cut homeobox 2
		Q96BN8	OTULIN	OTU deubiquitinase with linear linkage specificity
		P32243	OTX2	orthodenticle homeobox 2
		Q9BPZ3	PAIP2	poly(A) binding protein interacting protein 2
		Q9ULR5	PAIP2B	poly(A) binding protein interacting protein 2B
		Q8IXS6	PALM2	paralemmin 2

		Q8TE04	PANK1	pantothenate kinase 1
		Q6ZVX9	PAQR9	progestin and adipoQ receptor family member 9
		Q9BYG5	PARD6B	par-6 family cell polarity regulator beta
		Q9HBE1	PATZ1	POZ/BTB and AT hook containing zinc finger 1
		P55771	PAX9	paired box 9
		Q9HC56	PCDH9	protocadherin 9
		Q9Y6V0	PCLO	piccolo presynaptic cytomatrix protein
		Q96RV3	PCNX1	pecanex 1
		Q9Y233	PDE10A	phosphodiesterase 10A
		Q13370	PDE3B	phosphodiesterase 3B
		Q9NP56	PDE7B	phosphodiesterase 7B
		O00330	PDHX	pyruvate dehydrogenase complex component X
		Q14554	PDIA5	protein disulfide isomerase family A member 5
		Q16654	PDK4	pyruvate dehydrogenase kinase 4
		O15530	PDPK1	3-phosphoinositide dependent protein kinase 1
		Q9NTI5	PDS5B	PDS5 cohesin associated factor B
		Q9H792	PEAK1	pseudopodium enriched atypical kinase 1
		Q86TG7	PEG10	paternally expressed 10
		Q9HAT8	PELI2	pellino E3 ubiquitin protein ligase family member 2
		P02776	PF4	platelet factor 4
		P10720	PF4V1	platelet factor 4 variant 1
		P35232	PHB	prohibitin
		Q6ZVD8	PHLPP2	PH domain and leucine rich repeat protein phosphatase 2
		P42336	PIK3CA	phosphatidylinositol-4,5-bisphosphate 3-kinase catalytic subunit alpha
		P61925	PKIA	cAMP-dependent protein kinase inhibitor alpha
		Q6DJT9	PLAG1	PLAG1 zinc finger
		Q4KWH8	PLCH1	phospholipase C eta 1
		Q9UPR0	PLCL2	phospholipase C like 2

		Q9HAU0	PLEKHA5	pleckstrin homology domain containing A5
		Q9ULM0	PLEKHH1	pleckstrin homology, MyTH4 and FERM domain containing H1
		Q9NW61	PLEKHJ1	pleckstrin homology domain containing J1
		Q9NYY3	PLK2	polo like kinase 2
		O14495	PLPP3	phospholipid phosphatase 3
		Q8TBJ4	PLPPR1	phospholipid phosphatase related 1
		Q9Y4D7	PLXND1	plexin D1
		Q8TF01	PNISR	PNN interacting serine and arginine rich protein
		Q8N490	PNKD	PNKD, MBL domain containing
		Q6ZV29	PNPLA7	patatin like phospholipase domain containing 7
		Q8NBL1	POGLUT1	protein O-glucosyltransferase 1
		Q9UKI9	POU2F3	POU class 2 homeobox 3
		P20265	POU3F2	POU class 3 homeobox 2
		P37231	PPARG	peroxisome proliferator activated receptor gamma
		P30405	PPIF	peptidylprolyl isomerase F
		P36873	PPP1CC	protein phosphatase 1 catalytic subunit gamma
		Q9NY27	PPP4R2	protein phosphatase 4 regulatory subunit 2
		P54646	PRKAA2	protein kinase AMP-activated catalytic subunit alpha 2
		P05771	PRKCB	protein kinase C beta
		Q13976	PRKG1	protein kinase cGMP-dependent 1
		P51817	PRKX	protein kinase X-linked
		P16471	PRLR	prolactin receptor
		Q9NR22	PRMT8	protein arginine methyltransferase 8
		P79522	PRR3	proline rich 3
		P49768	PSEN1	presenilin 1
		P25786	PSMA1	proteasome subunit alpha 1
		Q8WXF1	PSPC1	paraspeckle component 1
		Q13635	PTCH1	patched 1
		Q13258	PTGDR	prostaglandin D2 receptor

		P43115	PTGER3	prostaglandin E receptor 3
		Q96PU8	QKI	QKI, KH domain containing RNA binding
		Q6WKZ4	RAB11FIP1	RAB11 family interacting protein 1
		Q2PPJ7	RALGAPA2	Ral GTPase activating protein catalytic alpha subunit 2
		P10114	RAP2A	RAP2A, member of RAS oncogene family
		Q9Y4G8	RAPGEF2	Rap guanine nucleotide exchange factor 2
		Q70E73	RAPH1	Ras association (RalGDS/AF-6) and pleckstrin homology domains 1
		P10276	RARA	retinoic acid receptor alpha
		Q86WH2	RASSF3	Ras association domain family member 3
		Q6ZRY4	RBPMS2	RNA binding protein, mRNA processing factor 2
		Q5TC82	RC3H1	ring finger and CCCH-type domains 1
		Q14206	RCAN2	regulator of calcineurin 2
		Q8NDN9	RCBTB1	RCC1 and BTB domain containing protein 1
		Q9P2K3	RCOR3	REST corepressor 3
		P78509	RELN	reelin
		Q96D71	REPS1	RALBP1 associated Eps domain containing 1
		O15211	RGL2	ral guanine nucleotide dissociation stimulator like 2
		Q7Z3J3	RGPD4	RANBP2-like and GRIP domain containing 4
		Q99666	RGPD5	RANBP2-like and GRIP domain containing 5
		Q99666	RGPD6	RANBP2-like and GRIP domain containing 6
		O14715	RGPD8	RANBP2-like and GRIP domain containing 8
		P57771	RGS8	regulator of G protein signaling 8
		Q9Y4F9	RIPOR2	RHO family interacting cell polarization regulator 2
		Q9H871	RMND5A	required for meiotic nuclear division 5 homolog A
		Q8WU17	RNF139	ring finger protein 139
		Q8WVD5	RNF141	ring finger protein 141
		Q8N6D2	RNF182	ring finger protein 182
		Q9H0F5	RNF38	ring finger protein 38

		O76064	RNF8	ring finger protein 8
		O60942	RNGTT	RNA guanylyltransferase and 5'-phosphatase
		P10155	RO60	Ro60, Y RNA binding protein
		Q01973	ROR1	receptor tyrosine kinase like orphan receptor 1
		Q68CZ1	RPGRIP1L	RPGRIP1 like
		P04844	RPN2	ribophorin II
		O75582	RPS6KA5	ribosomal protein S6 kinase A5
		P23443	RPS6KB1	ribosomal protein S6 kinase B1
		Q92766	RREB1	ras responsive element binding protein 1
		Q6PCB5	RSBN1L	round spermatid basic protein 1 like
		Q9BXY4	RSPO3	R-spondin 3
		Q7L099	RUFY3	RUN and FYVE domain containing 3
		Q01196	RUNX1	runt related transcription factor 1
		Q8N488	RYBP	RING1 and YY1 binding protein
		Q9UPW6	SATB2	SATB homeobox 2
		Q86WG5	SBF2	SET binding factor 2
		Q99590	SCAF11	SR-related CTD associated factor 11
		Q8N9R8	SCAI	suppressor of cancer cell invasion
		P35498	SCN1A	sodium voltage-gated channel alpha subunit 1
		Q99442	SEC62	SEC62 homolog, preprotein translocation factor
		Q96EE3	SEH1L	SEH1 like nucleoporin
		Q9H2E6	SEMA6A	semaphorin 6A
		Q8NFY4	SEMA6D	semaphorin 6D
		O75326	SEMA7A	semaphorin 7A (John Milton Hagen blood group)
		Q8NC51	SERBP1	SERPINE1 mRNA binding protein 1
		Q9C0A6	SETD5	SET domain containing 5
		Q8N474	SFRP1	secreted frizzled related protein 1
		Q12872	SFSWAP	splicing factor SWAP
		Q9BQI5	SGIP1	SH3 domain GRB2 like endophilin interacting protein 1

		Q99963	SH3GL3	SH3 domain containing GRB2 like 3, endophilin A3
		Q6S5L8	SHC4	SHC adaptor protein 4
		Q5VZ18	SHE	Src homology 2 domain containing E
		P57059	SIK1	salt inducible kinase 1
		A0A0B4J2F2	SIK1B	salt inducible kinase 1B (putative)
		Q15475	SIX1	SIX homeobox 1
		Q8WWT9	SLC13A3	solute carrier family 13 member 3
		A1A5C7	SLC22A23	solute carrier family 22 member 23
		O60721	SLC24A1	solute carrier family 24 member 1
		Q8NFF2	SLC24A4	solute carrier family 24 member 4
		P16260	SLC25A16	solute carrier family 25 member 16
		Q6KCM7	SLC25A25	solute carrier family 25 member 25
		Q8NEW0	SLC30A7	solute carrier family 30 member 7
		Q9Y2D2	SLC35A3	solute carrier family 35 member A3
		Q5T1Q4	SLC35F1	solute carrier family 35 member F1
		A4IF30	SLC35F4	solute carrier family 35 member F4
		Q8N1S5	SLC39A11	solute carrier family 39 member 11
		Q9GZV3	SLC5A7	solute carrier family 5 member 7
		P30531	SLC6A1	solute carrier family 6 member 1
		Q9UPY5	SLC7A11	solute carrier family 7 member 11
		P52569	SLC7A2	solute carrier family 7 member 2
		Q6AI14	SLC9A4	solute carrier family 9 member A4
		Q96T83	SLC9A7	solute carrier family 9 member A7
		Q4ZJI4	SLC9B1	solute carrier family 9 member B1
		Q9H2Y9	SLCO5A1	solute carrier organic anion transporter family member 5A1
		Q08AF3	SLFN5	schlafen family member 5
		O94813	SLIT2	slit guidance ligand 2
		Q96PX8	SLITRK1	SLIT and NTRK like family member 1
		Q13485	SMAD4	SMAD family member 4

		Q99717	SMAD5	SMAD family member 5
		O15198	SMAD9	SMAD family member 9
		Q9H3U7	SMOC2	SPARC related modular calcium binding 2
		P60880	SNAP25	synaptosome associated protein 25
		O75324	SNN	stannin
		Q8WVK2	SNRNP27	small nuclear ribonucleoprotein U4/U6.U5 subunit 27
		Q9Y5X0	SNX10	sorting nexin 10
		O94964	SOGA1	suppressor of glucose, autophagy associated 1
		Q92673	SORL1	sortilin related receptor 1
		Q07889	SOS1	SOS Ras/Rac guanine nucleotide exchange factor 1
		Q2M3V2	SOWAHA	sosondowah ankyrin repeat domain family member A
		P35716	SOX11	SRY-box 11
		Q9BT81	SOX7	SRY-box 7
		Q96N96	SPATA13	spermatogenesis associated 13
		O43597	SPRY2	sprouty RTK signaling antagonist 2
		O15270	SPTLC2	serine palmitoyltransferase long chain base subunit 2
		O75044	SRGAP2	SLIT-ROBO Rho GTPase activating protein 2
		Q86TD4	SRL	sarcalumenin
		P09132	SRP19	signal recognition particle 19
		O75177	SS18L1	SS18L1, nBAF chromatin remodeling complex subunit
		Q8WYL5	SSH1	slingshot protein phosphatase 1
		Q9Y5Y6	ST14	suppression of tumorigenicity 14
		Q9Y274	ST3GAL6	ST3 beta-galactoside alpha-2,3-sialyltransferase 6
		Q8NDV1	ST6GALNAC3	ST6 N-acetylgalactosaminide alpha-2,6-sialyltransferase 3
		Q8WWQ8	STAB2	stabilin 2
		Q99469	STAC	SH3 and cysteine rich domain
		Q8WVM7	STAG1	stromal antigen 1
		Q9UEW8	STK39	serine/threonine kinase 39
		Q93045	STMN2	stathmin 2

		O14662	STX16	syntaxin 16
		Q6J9G0	STYK1	serine/threonine/tyrosine kinase 1
		Q9UBS9	SUCO	SUN domain containing ossification factor
		O43426	SYNJ1	synaptojanin 1
		Q9UMZ2	SYNRG	synergin gamma
		Q7Z422	SZRD1	SUZ RNA binding domain containing 1
		Q8N5C8	TAB3	TGF-beta activated kinase 1 (MAP3K7) binding protein 3
		O75529	TAF5L	TATA-box binding protein associated factor 5 like
		Q6NXT6	TAPT1	transmembrane anterior posterior transformation 1
		Q16650	TBR1	T-box, brain 1
		Q9NR00	TCIM	transcriptional and immune response regulator
		P28347	TEAD1	TEA domain transcription factor 1
		Q8NHR7	TERB2	telomere repeat binding bouquet formation protein 2
		Q8NFU7	TET1	tet methylcytosine dioxygenase 1
		Q92481	TFAP2B	transcription factor AP-2 beta
		Q03167	TGFBR3	transforming growth factor beta receptor 3
		P10828	THRB	thyroid hormone receptor beta
		Q7Z2Z1	TICRR	TOPBP1 interacting checkpoint and replication regulator
		Q86UE8	TLK2	tousled like kinase 2
		Q9Y2S6	TMA7	translation machinery associated 7 homolog
		P55061	TMBIM6	transmembrane BAX inhibitor motif containing 6
		O94876	TMCC1	transmembrane and coiled-coil domain family 1
		Q8WVE7	TMEM170A	transmembrane protein 170A
		Q5T4T1	TMEM170B	transmembrane protein 170B
		Q6ZMB5	TMEM184A	transmembrane protein 184A
		Q96MH6	TMEM68	transmembrane protein 68
		Q6ZNR0	TMEM91	transmembrane protein 91
		Q9NQ34	TMEM9B	TMEM9 domain family member B
		Q7RTY8	TMPRSS7	transmembrane serine protease 7

		Q92973	TNPO1	transportin 1
		O15417	TNRC18	trinucleotide repeat containing 18
		Q9UPQ9	TNRC6B	trinucleotide repeat containing 6B
		O75674	TOM1L1	target of myb1 like 1 membrane trafficking protein
		Q5JU69	TOR2A	torsin family 2 member A
		P12270	TPR	translocated promoter region, nuclear basket protein
		Q13114	TRAF3	TNF receptor associated factor 3
		Q9Y228	TRAF3IP3	TRAF3 interacting protein 3
		Q9Y2L5	TRAPPC8	trafficking protein particle complex 8
		Q96PN7	TRERF1	transcriptional regulating factor 1
		Q7L0X0	TRIL	TLR4 interactor with leucine rich repeats
		P36406	TRIM23	tripartite motif containing 23
		Q86XT4	TRIM50	tripartite motif containing 50
		Q8NET8	TRPV3	transient receptor potential cation channel subfamily V member 3
		Q92574	TSC1	TSC complex subunit 1
		O75157	TSC22D2	TSC22 domain family member 2
		Q9H0U9	TSPYL1	TSPY like 1
		Q5SRH9	TTC39A	tetratricopeptide repeat domain 39A
		Q9NUQ3	TXLNG	taxilin gamma
		P61088	UBE2N	ubiquitin conjugating enzyme E2 N
		Q13404	UBE2V1	ubiquitin conjugating enzyme E2 V1
		O95071	UBR5	ubiquitin protein ligase E3 component n-recognin 5
		Q16739	UGCG	UDP-glucose ceramide glucosyltransferase
		Q6BDS2	UHRF1BP1	UHRF1 binding protein 1
		Q8NB66	UNC13C	unc-13 homolog C
		Q6UXZ4	UNC5D	unc-5 netrin receptor D
		Q9H9P5	UNKL	unk like zinc finger
		Q68DE3	USF3	upstream transcription factor family member 3

		Q9UHP3	USP25	ubiquitin specific peptidase 25
		Q70CQ4	USP31	ubiquitin specific peptidase 31
		Q9H9J4	USP42	ubiquitin specific peptidase 42
		P62068	USP46	ubiquitin specific peptidase 46
		O95292	VAPB	VAMP associated protein B and C
		P52735	VAV2	vav guanine nucleotide exchange factor 2
		Q9UKW4	VAV3	vav guanine nucleotide exchange factor 3
		Q8N0Z9	VSIG10	V-set and immunoglobulin domain containing 10
		Q9NX94	WBP1L	WW domain binding protein 1 like
		Q9H6R7	WDCP	WD repeat and coiled coil containing
		Q9Y2I8	WDR37	WD repeat domain 37
		Q8TF74	WIPF2	WAS/WASL interacting protein family member 2
		Q9BYP7	WNK3	WNK lysine deficient protein kinase 3
		Q9Y6I7	WSB1	WD repeat and SOCS box containing 1
		O14980	XPO1	exportin 1
		P31946	YWHAB	tyrosine 3-monooxygenase/tryptophan 5-monooxygenase activation protein beta
		P27348	YWHAQ	tyrosine 3-monooxygenase/tryptophan 5-monooxygenase activation protein theta
		Q9HC78	ZBTB20	zinc finger and BTB domain containing 20
		Q8NCN2	ZBTB34	zinc finger and BTB domain containing 34
		O15060	ZBTB39	zinc finger and BTB domain containing 39
		B2RXF5	ZBTB42	zinc finger and BTB domain containing 42
		Q8N2G6	ZCCHC24	zinc finger CCHC-type containing 24
		Q8IUH5	ZDHHC17	zinc finger DHHC-type containing 17
		Q9H8U3	ZFAND3	zinc finger AN1-type containing 3
		Q15911	ZFHX3	zinc finger homeobox 3
		P16415	ZFP36	ZFP36 ring finger protein
		Q9UKY1	ZHX1	zinc fingers and homeoboxes 1

		Q96T25	ZIC5	Zic family member 5
		Q9HA38	ZMAT3	zinc finger matrin-type 3
		Q5VZL5	ZMYM4	zinc finger MYM-type containing 4
		Q14587	ZNF268	zinc finger protein 268
		Q86UD4	ZNF329	zinc finger protein 329
		Q9BUY5	ZNF426	zinc finger protein 426
		Q9ULD9	ZNF608	zinc finger protein 608
		A8MWA4	ZNF705E	zinc finger protein 705E
		P17019	ZNF708	zinc finger protein 708
		P51504	ZNF80	zinc finger protein 80
		Q2TB10	ZNF800	zinc finger protein 800
		P51523	ZNF84	zinc finger protein 84
		Q16670	ZSCAN26	zinc finger and SCAN domain containing 26
		P26378	ELAVL4	Eukaryotic translation initiation factor 3 subunit M (eIF3m) (Fetal lung protein B5) (hFL-B5) (PCI domain-containing protein 1)
**TargetScan**	**miR-375 Upregulated 24 hours**	Q92870	APBB2	amyloid beta precursor protein binding family B member 2
**miRDB**		O15265	ATXN7	ataxin 7
		Q9NXV6	CDKN2AIP	CDKN2A interacting protein
		Q9NSP4	CENPM	centromere protein M
		A8K830	COLCA2	colorectal cancer associated 2
		Q9NS37	CREBZF	CREB/ATF bZIP transcription factor
		Q9Y2E4	DIP2C	disco interacting protein 2 homolog C
		P26378	ELAVL4	ELAV like RNA binding protein 4
		Q9H4A6	GOLPH3	golgi phosphoprotein 3
		P35680	HNF1B	HNF1 homeobox B
		P31249	HOXD3	homeobox D3
		Q6ZRS4	ITPRID1	ITPR interacting domain containing 1

		Q14722	KCNAB1	potassium voltage-gated channel subfamily A member regulatory beta subunit 1
		Q13887	KLF5	Kruppel like factor 5
		P07195	LDHB	lactate dehydrogenase B
		Q99677	LPAR4	lysophosphatidic acid receptor 4
		Q15546	MMD	monocyte to macrophage differentiation associated
		Q8N387	MUC15	mucin 15, cell surface associated
		Q14938	NFIX	nuclear factor I X
		Q9HB20	PLEKHA3	pleckstrin homology domain containing A3
		Q8TC44	POC1B	POC1 centriolar protein B
		Q03052	POU3F1	POU class 3 homeobox 1
		Q96PU8	QKI	QKI, KH domain containing RNA binding
		Q06330	RBPJ	recombination signal binding protein for immunoglobulin kappa J region
		Q13129	RLF	rearranged L-myc fusion
		Q8WU17	RNF139	ring finger protein 139
		Q15436	SEC23A	Sec23 homolog A, coat complex II component
		P36021	SLC16A2	solute carrier family 16 member 2
		O75159	SOCS5	suppressor of cytokine signaling 5
		O60271	SPAG9	sperm associated antigen 9
		Q1W4C9	SPINK13	serine peptidase inhibitor, Kazal type 13 (putative)
		Q08629	SPOCK1	SPARC (osteonectin), cwcv and kazal like domains proteoglycan 1
		Q92574	TSC1	TSC complex subunit 1
		Q05086	UBE3A	ubiquitin protein ligase E3A
		Q8NFA0	USP32	ubiquitin specific peptidase 32
		Q9NX94	WBP1L	WW domain binding protein 1 like
		Q6GPH4	XAF1	XIAP associated factor 1
		Q9HC78	ZBTB20	zinc finger and BTB domain containing 20

		P47974	ZFP36L2	ZFP36 ring finger protein like 2
		Q15915	ZIC1	Zic family member 1
		Q709F0	ACAD11	Acyl-CoA dehydrogenase family member 11 (ACAD-11) (EC 1.3.8.-)
**TargetScan**	**miR-505-5p Upregulated 6 hours**	Q96B36	AKT1S1	Proline-rich AKT1 substrate 1 (40 kDa proline-rich AKT substrate)
		Q6NS38	ALKBH2	DNA oxidative demethylase ALKBH2 (EC 1.14.11.33) (Alkylated DNA repair protein alkB homolog 2) (Alpha-ketoglutarate-dependent dioxygenase alkB homolog 2) (Oxy DC1)
		Q8N9V6	ANKRD53	Ankyrin repeat domain-containing protein 53
		E5RJM6	ANKRD65	Ankyrin repeat domain-containing protein 65
		Q9UH17	APOBEC3B	DNA dC->dU-editing enzyme APOBEC-3B (A3B) (EC 3.5.4.38) (Phorbolin-1-related protein) (Phorbolin-2/3)
		O14520	AQP7	Aquaporin-7 (AQP-7) (Aquaglyceroporin-7) (Aquaporin adipose) (AQPap) (Aquaporin-7-like)
		P0C7U1	ASAH2B	Putative inactive neutral ceramidase B (ASAH2-like protein) (Putative inactive N-acylsphingosine amidohydrolase 2B) (Putative inactive non-lysosomal ceramidase B)
		Q8NHE4	ATP6V0E2	V-type proton ATPase subunit e 2 (V-ATPase subunit e 2) (Lysosomal 9 kDa H(+)-transporting ATPase V0 subunit e2) (Vacuolar proton pump subunit e 2)
		O75531	BANF1	Barrier-to-autointegration factor (Breakpoint cluster region protein 1) [Cleaved into: Barrier-to-autointegration factor, N-terminally processed]
		Q6QNY0	BLOC1S3	Biogenesis of lysosome-related organelles complex 1 subunit 3 (BLOC-1 subunit 3)
		Q9NYX4	CALY	Neuron-specific vesicular protein calcyon
		O94983	CAMTA2	Calmodulin-binding transcription activator 2
		Q8WWF8	CAPSL	Calcyphosin-like protein

		Q9UIB8	CD84	SLAM family member 5 (Cell surface antigen MAX.3) (Hly9-beta) (Leukocyte differentiation antigen CD84) (Signaling lymphocytic activation molecule 5) (CD antigen CD84)
		Q00535	CDK5	Cyclin-dependent-like kinase 5 (EC 2.7.11.1) (Cell division protein kinase 5) (Serine/threonine-protein kinase PSSALRE) (Tau protein kinase II catalytic subunit) (TPKII catalytic subunit)
		Q96T59	CDRT15	CMT1A duplicated region transcript 15 protein
		A8MXV6	CDRT15L2	CMT1A duplicated region transcript 15 protein-like protein
		Q4G0U5	CFAP221	Cilia- and flagella-associated protein 221 (Primary ciliary dyskinesia protein 1)
		P0CG37	CFC1	Cryptic protein (Cryptic family protein 1)
		P0CG36	CFC1B	Cryptic family protein 1B
		Q9GZX3	CHST6	Carbohydrate sulfotransferase 6 (EC 2.8.2.-) (Corneal N-acetylglucosamine-6-O-sulfotransferase) (C-GlcNAc6ST) (hCGn6ST) (Galactose/N-acetylglucosamine/N-acetylglucosamine 6-O-sulfotransferase 4-beta) (GST4-beta) (N-acetylglucosamine 6-O-sulfotransferase 5) (GlcNAc6ST-5) (Gn6st-5)
		P0C7P0	CISD3	CDGSH iron-sulfur domain-containing protein 3, mitochondrial (MitoNEET-related protein 2) (Miner2) (Mitochondrial inner NEET protein) (MiNT)
		O95484	CLDN9	Claudin-9
		Q6UVW9	CLEC2A	C-type lectin domain family 2 member A (Keratinocyte-associated C-type lectin) (KACL) (Proliferation-induced lymphocyte-associated receptor) (PILAR)
		Q6YFQ2	COX6B2	Cytochrome c oxidase subunit 6B2 (Cancer/testis antigen 59) (CT59) (Cytochrome c oxidase subunit VIb isoform 2) (COX VIb-2) (Cytochrome c oxidase subunit VIb, testis-specific isoform)

		Q32M00	Crebl2	cAMP-responsive element-binding protein-like 2
		O75629	CREG1	Protein CREG1 (Cellular repressor of E1A-stimulated genes 1)
		Q6Q6R5	CRIP3	Cysteine-rich protein 3 (CRP-3) (Chromosome 6 LIM domain only protein) (h6LIMo)
		Q8N8Q1	CYB561D1	Cytochrome b561 domain-containing protein 1
		P32321	DCTD	Deoxycytidylate deaminase (EC 3.5.4.12) (dCMP deaminase)
		Q18PE1	DOK7	Protein Dok-7 (Downstream of tyrosine kinase 7)
		Q6P2I7	EBLN2	Endogenous Bornavirus-like nucleoprotein 2 (Endogenous Borna-like N element-2) (EBLN-2)
		Q96G04	EEF2KMT	Protein-lysine N-methyltransferase EEF2KMT (EC 2.1.1.-) (eEF2-lysine methyltransferase) (eEF2-KMT)
		Q9UKW6	ELF5	ETS-related transcription factor Elf-5 (E74-like factor 5) (Epithelium-restricted ESE-1-related Ets factor) (Epithelium-specific Ets transcription factor 2) (ESE-2)
		Q6UWT2	ENHO	Adropin (Energy homeostasis-associated protein)
		Q8N3H0	FAM19A2	
		Q9NVL1	FAM86C1	Protein FAM86C1 (EC 2.1.1.-) (Protein FAM86C)
		Q9NXK8	FBXL12	F-box/LRR-repeat protein 12 (F-box and leucine-rich repeat protein 12) (F-box protein FBL12)
		Q9BVA6	FICD	Protein adenylyltransferase FICD (EC 2.7.7.n1) (AMPylator FICD) (De-AMPylase FICD) (EC 3.1.4.-) (FIC domain-containing protein) (Huntingtin yeast partner E) (Huntingtin-interacting protein 13) (HIP-13) (Huntingtin-interacting protein E)
		Q96CU9	FOXRED1	FAD-dependent oxidoreductase domain-containing protein 1 (EC 1.-.-.-)
		Q96C23	GALM	Aldose 1-epimerase (EC 5.1.3.3) (Galactose mutarotase)

		Q8WTR4	GDPD5	Glycerophosphodiester phosphodiesterase domain-containing protein 5 (EC 3.1.4.11) (Glycerophosphodiester phosphodiesterase 2)
		Q9BZE0	GLIS2	Zinc finger protein GLIS2 (GLI-similar 2) (Neuronal Krueppel-like protein)
		Q8WP90	Gp-9	G-protein coupled receptor 26
		Q8NDV2	GPR26	
		Q00960	Grin2b	Glutamate receptor ionotropic, NMDA 2B (GluN2B) (Glutamate [NMDA] receptor subunit epsilon-2) (N-methyl D-aspartate receptor subtype 2B) (NMDAR2B) (NR2B) (N-methyl-D-aspartate receptor subunit 3) (NR3) (hNR3)
		Q9Y2Q3	GSTK1	Glutathione S-transferase kappa 1 (EC 2.5.1.18) (GST 13-13) (GST class-kappa) (GSTK1-1) (hGSTK1) (Glutathione S-transferase subunit 13)
		Q9Y5R4	HEMK1	MTRF1L release factor glutamine methyltransferase (EC 2.1.1.297) (HemK methyltransferase family member 1) (M.HsaHemKP)
		P04440	HLA-DPB1	HLA class II histocompatibility antigen, DP beta 1 chain (HLA class II histocompatibility antigen, DP(W4) beta chain) (MHC class II antigen DPB1)
		Q30154	HLA-DRB5	HLA class II histocompatibility antigen, DR beta 5 chain (DR beta-5) (DR2-beta-2) (Dw2) (MHC class II antigen DRB5)
		P29460	IL12B	Interleukin-12 subunit beta (IL-12B) (Cytotoxic lymphocyte maturation factor 40 kDa subunit) (CLMF p40) (IL-12 subunit p40) (NK cell stimulatory factor chain 2) (NKSF2)
		Q9ULR0	ISY1	Pre-mRNA-splicing factor ISY1 homolog
		Q6ZU35	KIAA1211	
		P62502	LCN6	Epididymal-specific lipocalin-6 (Lipocalin-5)
		Q96DT0	LGALS12	Galectin-12 (Gal-12) (Galectin-related inhibitor of proliferation)

		O95867	LY6G6C	Lymphocyte antigen 6 complex locus protein G6c
		Q9H063	MAF1	Repressor of RNA polymerase III transcription MAF1 homolog
		Q8IVH8	MAP4K3	Mitogen-activated protein kinase kinase kinase kinase 3 (EC 2.7.11.1) (Germinal center kinase-related protein kinase) (GLK) (MAPK/ERK kinase kinase kinase 3) (MEK kinase kinase 3) (MEKKK 3)
		P14174	MIF	Macrophage migration inhibitory factor (MIF) (EC 5.3.2.1) (Glycosylation-inhibiting factor) (GIF) (L-dopachrome isomerase) (L-dopachrome tautomerase) (EC 5.3.3.12) (Phenylpyruvate tautomerase)
		Q95460	MR1	Major histocompatibility complex class I-related gene protein (MHC class I-related gene protein) (Class I histocompatibility antigen-like protein)
		Q8IXM3	MRPL41	39S ribosomal protein L41, mitochondrial (L41mt) (MRP-L41) (39S ribosomal protein L27 homolog) (Bcl-2-interacting mitochondrial ribosomal protein L41) (Cell proliferation-inducing gene 3 protein) (MRP-L27 homolog) (Mitochondrial large ribosomal subunit protein mL41)
		O15235	MRPS12	28S ribosomal protein S12, mitochondrial (MRP-S12) (S12mt) (MT-RPS12) (Mitochondrial small ribosomal subunit protein uS12m)
		Q96EZ4	MYEOV	Myeloma-overexpressed gene protein (Oncogene in multiple myeloma)
		Q9NP98	MYOZ1	Myozenin-1 (Calsarcin-2) (Filamin-, actinin- and telethonin-binding protein) (Protein FATZ)
		Q92692	NECTIN2	Nectin-2 (Herpes virus entry mediator B) (Herpesvirus entry mediator B) (HveB) (Nectin cell adhesion molecule 2) (Poliovirus receptor-related protein 2) (CD antigen CD112)
		Q9Y3C1	NOP16	Nucleolar protein 16 (HBV pre-S2 trans-regulated protein 3)
		Q96PH1	NOX5	NADPH oxidase 5 (EC 1.6.3.-)

		Q9Y328	NSG2	Neuronal vesicle trafficking-associated protein 2 (Neuron-specific protein family member 2) (Protein p19) (Hmp19)
		P49902	NT5C2	Cytosolic purine 5'-nucleotidase (EC 3.1.3.5) (Cytosolic 5'-nucleotidase II)
		P30954	OR10J1	Olfactory receptor 10J1 (Olfactory receptor OR1-26) (Olfactory receptor-like protein HGMP07J)
		Q9HC10	OTOF	Otoferlin (Fer-1-like protein 2)
		Q8WXA2	PATE1	Prostate and testis expressed protein 1
		P39877	PLA2G5	Calcium-dependent phospholipase A2 (EC 3.1.1.4) (Group V phospholipase A2) (PLA2-10) (Phosphatidylcholine 2-acylhydrolase 5)
		P24158	PRTN3	Myeloblastin (EC 3.4.21.76) (AGP7) (C-ANCA antigen) (Leukocyte proteinase 3) (PR-3) (PR3) (Neutrophil proteinase 4) (NP-4) (P29) (Wegener autoantigen)
		Q9Z1X2	Ptdss2	Phosphatidylserine synthase 2 (PSS-2) (PtdSer synthase 2) (EC 2.7.8.29) (Serine-exchange enzyme II)
		E9PB15	PTGES3L	Putative protein PTGES3L (Prostaglandin E synthase 3-like)
		P49190	PTH2R	Parathyroid hormone 2 receptor (PTH2 receptor)
		Q96A99	PTX4	Pentraxin-4
		Q8K459	Pxt1	Peroxisomal testis-specific protein 1 (Small testis-specific peroxisomal protein)
		Q9H2L5	RASSF4	Ras association domain-containing protein 4
		Q8TAI7	RHEBL1	GTPase RhebL1 (Ras homolog enriched in brain like-1 c) (RhebL1c) (Ras homolog enriched in brain-like protein 1) (Rheb-like protein 1) (Rheb2)
		P08134	RHOC	Rho-related GTP-binding protein RhoC (Rho cDNA clone 9) (h9)
		P39019	RPS19	40S ribosomal protein S19 (Small ribosomal subunit protein eS19)

		Q5SSQ6	SAPCD1	Suppressor APC domain-containing protein 1 (Protein G7d)
		Q9H7N4	SCAF1	Splicing factor, arginine/serine-rich 19 (SR-related C-terminal domain-associated factor 1) (SR-related and CTD-associated factor 1) (SR-related-CTD-associated factor) (SCAF) (Serine arginine-rich pre-mRNA splicing factor SR-A1) (SR-A1)
		Q9NZV5	SELENON	Selenoprotein N (SelN)
		Q9NR46	SH3GLB2	Endophilin-B2 (SH3 domain-containing GRB2-like protein B2)
		O15389	SIGLEC5	Sialic acid-binding Ig-like lectin 5 (Siglec-5) (CD33 antigen-like 2) (Obesity-binding protein 2) (OB-BP2) (OB-binding protein 2) (CD antigen CD170)
		Q9P1W8	SIRPG	Signal-regulatory protein gamma (SIRP-gamma) (CD172 antigen-like family member B) (Signal-regulatory protein beta-2) (SIRP-b2) (SIRP-beta-2) (CD antigen CD172g)
		Q9NQ25	SLAMF7	SLAM family member 7 (CD2 subset 1) (CD2-like receptor-activating cytotoxic cells) (CRACC) (Membrane protein FOAP-12) (Novel Ly9) (Protein 19A) (CD antigen CD319)
		Q9BYW1	SLC2A11	Solute carrier family 2, facilitated glucose transporter member 11 (Glucose transporter type 11) (GLUT-11)
		P22732	SLC2A5	Solute carrier family 2, facilitated glucose transporter member 5 (Fructose transporter) (Glucose transporter type 5, small intestine) (GLUT-5)
		P03973	SLPI	Antileukoproteinase (ALP) (BLPI) (HUSI-1) (Mucus proteinase inhibitor) (MPI) (Protease inhibitor WAP4) (Secretory leukocyte protease inhibitor) (Seminal proteinase inhibitor) (WAP four-disulfide core domain protein 4)
		P55000	SLURP1	Secreted Ly-6/uPAR-related protein 1 (SLURP-1) (ARS component B) (ARS(component B)-81/S) (Anti-neoplastic urinary protein) (ANUP)

		Q0VAQ4	SMAGP	Small cell adhesion glycoprotein (Small transmembrane and glycosylated protein)
		P0DI80	SMIM6	Small integral membrane protein 6
		Q53HV7	SMUG1	Single-strand selective monofunctional uracil DNA glycosylase (EC 3.2.2.-)
		O75971	SNAPC5	snRNA-activating protein complex subunit 5 (SNAPc subunit 5) (Small nuclear RNA-activating complex polypeptide 5) (snRNA-activating protein complex 19 kDa subunit) (SNAPc 19 kDa subunit)
		Q13425	SNTB2	Beta-2-syntrophin (59 kDa dystrophin-associated protein A1 basic component 2) (Syntrophin-3) (SNT3) (Syntrophin-like) (SNTL)
		Q9Y675	SNURF	SNRPN upstream reading frame protein
		Q04888	Sox10	Transcription factor SOX-10
		Q9UNL2	SSR3	Translocon-associated protein subunit gamma (TRAP-gamma) (Signal sequence receptor subunit gamma) (SSR-gamma)
		O43173	ST8SIA3	Sia-alpha-2,3-Gal-beta-1,4-GlcNAc-R:alpha 2,8-sialyltransferase (EC 2.4.99.-) (Alpha-2,8-sialyltransferase 8C) (Alpha-2,8-sialyltransferase III) (ST8 alpha-N-acetyl-neuraminide alpha-2,8-sialyltransferase 3) (Sialyltransferase 8C) (SIAT8-C) (Sialyltransferase St8Sia III) (ST8SiaIII)
		P26639	TARS	Threonine--tRNA ligase 1, cytoplasmic (EC 6.1.1.3) (Threonyl-tRNA synthetase) (ThrRS) (Threonyl-tRNA synthetase 1)
		Q96PF1	TGM7	Protein-glutamine gamma-glutamyltransferase Z (Transglutaminase Z) (TG(Z)) (TGZ) (TGase Z) (EC 2.3.2.13) (Transglutaminase-7) (TGase-7)
		C9JI98	TMEM238	Transmembrane protein 238
		Q8K288	Tnfaip8l1	Tumor necrosis factor alpha-induced protein 8-like protein 1 (TIPE1) (TNF alpha-induced protein 8-like protein 1)

				(TNFAIP8-like protein 1) (Oxidative stress-regulated gene-beta) (Oxy-beta)
		Q9NP84	TNFRSF12A	Tumor necrosis factor receptor superfamily member 12A (Fibroblast growth factor-inducible immediate-early response protein 14) (FGF-inducible 14) (Tweak-receptor) (TweakR) (CD antigen CD266)
		O95150	TNFSF15	Tumor necrosis factor ligand superfamily member 15 (TNF ligand-related molecule 1) (Vascular endothelial cell growth inhibitor) [Cleaved into: Tumor necrosis factor ligand superfamily member 15, membrane form; Tumor necrosis factor ligand superfamily member 15, secreted form]
		Q15025	TNIP1	TNFAIP3-interacting protein 1 (A20-binding inhibitor of NF-kappa-B activation 1) (ABIN-1) (HIV-1 Nef-interacting protein) (Nef-associated factor 1) (Naf1) (Nip40-1) (Virion-associated nuclear shuttling protein) (VAN) (hVAN)
		Q9HCN2	TP53AIP1	p53-regulated apoptosis-inducing protein 1 (p53AIP1)
		Q8NEE8	TTC16	Tetratricopeptide repeat protein 16 (TPR repeat protein 16)
		Q8NA23	WDR31	WD repeat-containing protein 31
		Q93097	WNT2B	Protein Wnt-2b (Protein Wnt-13)
		Q7L2R6	ZNF765	Zinc finger protein 765
		Q2QGD7	ZXDC	Zinc finger protein ZXDC (ZXD-like zinc finger protein)
		Q709F0	ACAD11	acyl-CoA dehydrogenase family member 11
**miRDB**		Q96B36	AKT1S1	AKT1 substrate 1
		Q9UH17	APOBEC3B	apolipoprotein B mRNA editing enzyme catalytic subunit 3B
		Q8NHH9	ATL2	atlastin GTPase 2
		P14415	ATP1B2	ATPase Na+/K+ transporting subunit beta 2
		Q9NTI2	ATP8A2	ATPase phospholipid transporting 8A2
		O75531	BANF1	barrier to autointegration factor 1

		Q6UWT4	C5orf46	chromosome 5 open reading frame 46
		O15484	CAPN5	calpain 5
		Q8WWF8	CAPSL	calcyphosine like
		Q00535	CDK5	cyclin dependent kinase 5
		Q6UVW9	CLEC2A	C-type lectin domain family 2 member A
		O60519	CREBL2	cAMP responsive element binding protein like 2
		O75629	CREG1	cellular repressor of E1A stimulated genes 1
		Q15700	DLG2	discs large MAGUK scaffold protein 2
		P07992	ERCC1	ERCC excision repair 1, endonuclease non-catalytic subunit
		P50395	GDI2	GDP dissociation inhibitor 2
		Q9BZE0	GLIS2	GLIS family zinc finger 2
		P09471	GNAO1	G protein subunit alpha o1
		Q8NDV2	GPR26	G protein-coupled receptor 26
		O60381	HBP1	HMG-box transcription factor 1
		O43365	HOXA3	homeobox A3
		P11279	LAMP1	lysosomal associated membrane protein 1
		Q9P1T7	MDFIC	MyoD family inhibitor domain containing
		P51608	MECP2	methyl-CpG binding protein 2
		Q9Y5R2	MMP24	matrix metallopeptidase 24
		Q95460	MR1	major histocompatibility complex, class I-related
		Q6NW34	NEPRO	nucleolus and neural progenitor protein
		Q9Y328	NSG2	neuronal vesicle trafficking associated 2
		P49902	NT5C2	5'-nucleotidase, cytosolic II
		Q9BZ71	PITPNM3	PITPNM family member 3
		Q9HBU9	POPDC2	popeye domain containing 2
		O00231	PSMD11	proteasome 26S subunit, non-ATPase 11
		Q8NFP0	PXT1	peroxisomal testis enriched protein 1
		P20337	RAB7B	RAB7B, member RAS oncogene family
		P13631	RARG	retinoic acid receptor gamma

		Q86VR2	RETREG3	reticulophagy regulator family member 3
		Q5SSQ6	SAPCD1	suppressor APC domain containing 1
		O75056	SDC3	syndecan 3
		Q9P0U3	SENP1	SUMO specific peptidase 1
		P07988	SFTPB	surfactant protein B
		O15389	SIGLEC5	sialic acid binding Ig like lectin 5
		Q9NQ25	SLAMF7	SLAM family member 7
		P22732	SLC2A5	solute carrier family 2 member 5
		Q9ULF5	SLC39A10	solute carrier family 39 member 10
		P57103	SLC8A3	solute carrier family 8 member A3
		Q0VAQ4	SMAGP	small cell adhesion glycoprotein
		Q92922	SMARCC1	SWI/SNF related, matrix associated, actin dependent regulator of chromatin subfamily c member 1
		Q11201	ST3GAL1	ST3 beta-galactoside alpha-2,3-sialyltransferase 1
		O43173	ST8SIA3	ST8 alpha-N-acetyl-neuraminide alpha-2,8-sialyltransfe
		Q9BQS2	SYT15	synaptotagmin 15
		Q96PF1	TGM7	transglutaminase 7
		Q9BTF0	THUMPD2	THUMP domain containing 2
		Q15025	TNIP1	TNFAIP3 interacting protein 1
		Q9HCF6	TRPM3	transient receptor potential cation channel subfamily M member 3
		Q86VN1	VPS36	vacuolar protein sorting 36 homolog
		Q8N966	ZDHHC22	zinc finger DHHC-type containing 22
		Q5VUY2	AADACL4	Arylacetamide deacetylase-like 4 (EC 3.1.1.-)
**TargetScan**	**miR-512-3p Downregulated 24 hours**	P50052	AGTR2	Type-2 angiotensin II receptor (Angiotensin II type-2 receptor) (AT2)
		O43572	AKAP10	A-kinase anchor protein 10, mitochondrial (AKAP-10) (Dual specificity A kinase-anchoring protein 2) (D-AKAP-2) (Protein kinase A-anchoring protein 10) (PRKA10)

		Q9H8T0	AKTIP	AKT-interacting protein (Ft1) (Fused toes protein homolog)
		Q96PC3	AP1S3	AP-1 complex subunit sigma-3 (Adaptor protein complex AP-1 subunit sigma-1C) (Adaptor-related protein complex 1 subunit sigma-1C) (Clathrin assembly protein complex 1 sigma-1C small chain) (Golgi adaptor HA1/AP1 adaptin sigma-1C subunit) (Sigma 1C subunit of AP-1 clathrin) (Sigma-adaptin 1C) (Sigma1C-adaptin)
		Q96KC2	ARL5B	ADP-ribosylation factor-like protein 5B (ADP-ribosylation factor-like protein 8)
		Q16204	CCDC6	Coiled-coil domain-containing protein 6
		Q9UNH5	CDC14A	Dual specificity protein phosphatase CDC14A
		Q8IVW4	CDKL3	Cyclin-dependent kinase-like 3
		Q9NXV6	CDKN2AIP	CDKN2A-interacting protein
		B2RD01	CENPBD1	Putative CENPB DNA-binding domain-containing protein 1
		Q2UY09	COL28A1	Collagen alpha-1(XXVIII) chain
		Q8N6G5	CSGALNACT2	Chondroitin sulfate N-acetylgalactosaminyltransferase 2
		Q9H4G1	CST9L	Cystatin-9-like
		Q9H1C7	CYSTM1	Cysteine-rich and transmembrane domain-containing protein 1
		Q8WTU0	DDI1	Protein DDI1 homolog 1
		Q9Y315	DERA	Deoxyribose-phosphate aldolase
		Q8IX18	DHX40	Probable ATP-dependent RNA helicase DHX40
		Q7Z6W7	DNAJB7	DnaJ homolog subfamily B member 7
		Q9NR20	DYRK4	Dual specificity tyrosine-phosphorylation-regulated kinase 4
		O95278	EPM2A	Laforin
		Q6P587	FAHD1	Acylpyruvase FAHD1, mitochondrial
		Q1W6H9	FAM110C	Protein FAM110C
		Q5T036	FAM120AOS	Uncharacterized protein FAM120AOS
		Q8WU58	FAM222B	Protein FAM222B

		Q9UF56	FBXL17	F-box/LRR-repeat protein 17 (F-box and leucine-rich repeat protein 17) (F-box only protein 13)
		Q92913	FGF13	Fibroblast growth factor 13 (FGF-13) (Fibroblast growth factor homologous factor 2) (FHF-2)
		Q5T0N5	FNBP1L	Formin-binding protein 1-like (Transducer of Cdc42-dependent actin assembly protein 1) (Toca-1)
		P19526	FUT1	Galactoside 2-alpha-L-fucosyltransferase 1 (EC 2.4.1.344) (Alpha(1,2)FT 1) (Blood group H alpha 2-fucosyltransferase) (Fucosyltransferase 1) (GDP-L-fucose:beta-D-galactoside 2-alpha-L-fucosyltransferase 1)
		O43903	GAS2	Growth arrest-specific protein 2 (GAS-2)
		Q9NZD2	GLTP	Glycolipid transfer protein (GLTP)
		P00492	HPRT1	Hypoxanthine-guanine phosphoribosyltransferase (HGPRT) (HGPRTase) (EC 2.4.2.8)
		Q8IY31	IFT20	Intraflagellar transport protein 20 homolog (hIFT20)
		Q8N5I3	KCNRG	Potassium channel regulatory protein
		B2RXH2	KDM4E	Lysine-specific demethylase 4E
		Q14695	KIAA0087	Uncharacterized protein KIAA0087
		Q96MU8	KREMEN1	Kremen protein 1
		P35900	KRT20	Keratin, type I cytoskeletal 20
		P26371	KRTAP5-9	Keratin-associated protein 5-9 (Keratin, cuticle, ultrahigh sulfur 1) (Keratin, ultra high-sulfur matrix protein A) (Keratin-associated protein 5.9) (UHS keratin A) (UHS KerA) (Ultrahigh sulfur keratin-associated protein 5.9)
		Q96JN0	LCOR	Ligand-dependent corepressor (LCoR) (Mblk1-related protein 2)

		P28482	MAPK1	Mitogen-activated protein kinase 1 (MAP kinase 1) (MAPK 1) (EC 2.7.11.24) (ERT1) (Extracellular signal-regulated kinase 2) (ERK-2) (MAP kinase isoform p42) (p42-MAPK) (Mitogen-activated protein kinase 2) (MAP kinase 2) (MAPK 2)
		Q96EN8	MOCOS	Molybdenum cofactor sulfurase (MCS) (MOS) (MoCo sulfurase) (hMCS) (EC 2.8.1.9) (Molybdenum cofactor sulfurtransferase)
		Q86T75	NBPF11	Neuroblastoma breakpoint family member 11
		Q9BQ51	PDCD1LG2	Programmed cell death 1 ligand 2
		Q8N165	PDIK1L	Serine/threonine-protein kinase PDIK1L
		Q8IZ21	PHACTR4	Phosphatase and actin regulator 4
		Q8WYR1	PIK3R5	Phosphoinositide 3-kinase regulatory subunit 5
		Q8WZA1	POMGNT1	Protein O-linked-mannose beta-1,2-N-acetylglucosaminyltransferase 1
		Q07869	PPARA	Peroxisome proliferator-activated receptor alpha
		Q00LT1	PRCD	Photoreceptor disk component PRCD
		P13861	PRKAR2A	cAMP-dependent protein kinase type II-alpha regulatory subunit
		Q8IV56	PRR15	Proline-rich protein 15
		P20337	RAB3B	Ras-related protein Rab-3B
		P61587	RND3	Rho-related GTP-binding protein RhoE
		Q9BXT8	RNF17	RING finger protein 17
		Q7Z769	SLC35E3	Solute carrier family 35 member E3
		O15198	SMAD2	Mothers against decapentaplegic homolog 9
		Q16637	SMN1	Survival motor neuron protein
		O43791	SPOP	Speckle-type POZ protein
		Q16384	SSX1	Protein SSX1
		O60224	SSX4	Protein SSX4
		Q7RTT5	SSX7	Protein SSX7

		Q92186	ST8SIA2	Alpha-2,8-sialyltransferase 8B
		P56279	TCL1A	T-cell leukemia/lymphoma protein 1A
		P10828	THRB	Thyroid hormone receptor beta (Nuclear receptor subfamily 1 group A member 2) (c-erbA-2) (c-erbA-beta)
		Q96DC7	TMCO6	Transmembrane and coiled-coil domain-containing protein 6
		A2RRL7	TMEM213	Transmembrane protein 213
		Q13829	TNFAIP1	BTB/POZ domain-containing adapter for CUL3-mediated RhoA degradation protein 2 (hBACURD2) (BTB/POZ domain-containing protein TNFAIP1) (Protein B12) (Tumor necrosis factor, alpha-induced protein 1, endothelial)
		Q9H4B7	TUBB1	Tubulin beta-1 chain
		O95164	UBL3	Ubiquitin-like protein 3 (Membrane-anchored ubiquitin-fold protein) (HsMUB) (MUB) (Protein HCG-1)
		Q9Y6I4	USP3	Ubiquitin carboxyl-terminal hydrolase 3 (EC 3.4.19.12) (Deubiquitinating enzyme 3) (Ubiquitin thioesterase 3) (Ubiquitin-specific-processing protease 3)
		P34547	usp-46	Ubiquitin carboxyl-terminal hydrolase 46
		Q8TCV5	WFDC5	WAP four-disulfide core domain protein 5 (Putative protease inhibitor WAP1) (p53-responsive gene 5 protein)
		P17014	ZNF12	Zinc finger protein 12 (Gonadotropin-inducible ovary transcription repressor 3) (GIOT-3) (Zinc finger protein 325) (Zinc finger protein KOX3)
		O14771	ZNF213	Zinc finger protein 213 (Putative transcription factor CR53) (Zinc finger protein with KRAB and SCAN domains 21)
		Q9Y462	ZNF711	Zinc finger protein 711 (Zinc finger protein 6)
		Q3KNS6	ZNF829	Zinc finger protein 829
		Q0P651	ABHD18	abhydrolase domain containing 18
**miRDB**		P42684	ABL2	ABL proto-oncogene 2, non-receptor tyrosine kinase
		Q9Y3Q7	ADAM18	ADAM metallopeptidase domain 18

		P82987	ADAMTSL3	ADAMTS like 3
		Q08828	ADCY1	adenylate cyclase 1
		P50052	AGTR2	angiotensin II receptor type 2
		Q09666	AHNAK	AHNAK nucleoprotein
		A9YTQ3	AHRR	aryl-hydrocarbon receptor repressor
		Q9H8T0	AKTIP	AKT interacting protein
		O15013	ARHGEF10	Rho guanine nucleotide exchange factor 10
		Q8IVW1	ARL17A	ADP ribosylation factor like GTPase 17A
		Q96KC2	ARL5B	ADP ribosylation factor like GTPase 5B
		P54253	ATXN1	ataxin 1
		O15265	ATXN7	ataxin 7
		Q9H694	BICC1	BicC family RNA binding protein 1
		Q9H246	C1orf21	chromosome 1 open reading frame 21
		Q8N3J6	CADM2	cell adhesion molecule 2
		Q9Y6Y1	CAMTA1	calmodulin binding transcription activator 1
		Q6IMN6	CAPRIN2	caprin family member 2
		Q16204	CCDC6	coiled-coil domain containing 6
		Q9BWU1	CDK19	cyclin dependent kinase 19
		P14384	CPM	carboxypeptidase M
		O60494	CUBN	cubilin
		P39880	CUX1	cut like homeobox 1
		Q7LFL8	CXXC5	CXXC finger protein 5
		Q7Z449	CYP2U1	cytochrome P450 family 2 subfamily U member 1
		Q9H1C7	CYSTM1	cysteine rich transmembrane module containing 1
		Q8WTU0	DDI1	DNA damage inducible 1 homolog 1
		Q8IZD9	DOCK3	dedicator of cytokinesis 3
		Q92630	DYRK2	dual specificity tyrosine phosphorylation regulated kinase 2
		A4FU69	EFCAB5	EF-hand calcium binding domain 5
		Q9H2F5	EPC1	enhancer of polycomb homolog 1

		Q15303	ERBB3	erb-b2 receptor tyrosine kinase 3
		Q9H6T0	ESRP2	epithelial splicing regulatory protein 2
		Q6V0I7	FAT4	FAT atypical cadherin 4
		Q96JP0	FEM1C	fem-1 homolog C
		O95684	FGFR1OP	FGFR1 oncogene partner
		Q14314	FGL2	fibrinogen like 2
		Q01167	FOXK2	forkhead box K2
		O43903	GAS2	growth arrest specific 2
		Q8TF65	GIPC2	GIPC PDZ domain containing family member 2
		Q3T8J9	GON4L	gon-4 like
		Q4G148	GXYLT1	glucoside xylosyltransferase 1
		Q16534	HLF	HLF, PAR bZIP transcription factor
		P00492	HPRT1	hypoxanthine phosphoribosyltransferase 1
		P40189	IL6ST	interleukin 6 signal transducer
		Q15652	JMJD1C	jumonji domain containing 1C
		Q92794	KAT6A	lysine acetyltransferase 6A
		Q86Z14	KLB	klotho beta
		Q6P9F7	LRRC8B	leucine rich repeat containing 8 VRAC subunit B
		P53779	MAPK10	mitogen-activated protein kinase 10
		Q5T0T0	MARCH8	membrane associated ring-CH-type finger 8
		O15021	MAST4	microtubule associated serine/threonine kinase family member 4
		P55198	MLLT6	MLLT6, PHD finger containing
		Q9UL68	MYT1L	myelin transcription factor 1 like
		O00712	NFIB	nuclear factor I B
		Q9Y239	NOD1	nucleotide binding oligomerization domain containing 1
		Q9Y466	NR2E1	nuclear receptor subfamily 2 group E member 1
		Q9Y5K3	PCYT1B	phosphate cytidylyltransferase 1, choline, beta
		Q8N165	PDIK1L	PDLIM1 interacting kinase 1 like

		Q01813	PFKP	phosphofructokinase, platelet
		P35080	PFN2	profilin 2
		Q8IZ21	PHACTR4	phosphatase and actin regulator 4
		Q8WYR1	PIK3R5	phosphoinositide-3-kinase regulatory subunit 5
		Q5VZY2	PLPP4	phospholipid phosphatase 4
		Q07869	PPARA	peroxisome proliferator activated receptor alpha
		Q08209	PPP3CA	protein phosphatase 3 catalytic subunit alpha
		Q5MIZ7	PPP4R3B	protein phosphatase 4 regulatory subunit 3B
		P13861	PRKAR2A	protein kinase cAMP-dependent type II regulatory subunit alpha
		Q9UKA9	PTBP2	polypyrimidine tract binding protein 2
		Q96NR3	PTCHD1	patched domain containing 1
		P29074	PTPN4	protein tyrosine phosphatase, non-receptor type 4
		Q2KHR3	QSER1	glutamine and serine rich 1
		Q15276	RABEP1	rabaptin, RAB GTPase binding effector protein 1
		P61587	RND3	Rho family GTPase 3
		Q9H0F5	RNF38	ring finger protein 38
		P62341	SELENOT	selenoprotein T
		Q9UHJ3	SFMBT1	Scm like with four mbt domains 1
		Q9P270	SLAIN2	SLAIN motif family member 2
		O15403	SLC16A6	solute carrier family 16 member 6
		P48066	SLC6A11	solute carrier family 6 member 11
		O15198	SMAD9	SMAD family member 9
		O75643	SNRNP200	small nuclear ribonucleoprotein U5 subunit 200
		O43791	SPOP	speckle type BTB/POZ protein
		Q9C0D5	TANC1	tetratricopeptide repeat, ankyrin repeat and coiled-coil containing 1
		P10828	THRB	thyroid hormone receptor beta
		Q9BRN9	TM2D3	TM2 domain containing 3

		Q9H2K2	TNKS2	tankyrase 2
		Q96A56	TP53INP1	tumor protein p53 inducible nuclear protein 1
		Q9H3D4	TP63	tumor protein p63
		Q9UPV9	TRAK1	trafficking kinesin protein 1
		Q9UKU6	TRHDE	thyrotropin releasing hormone degrading enzyme
		Q5W5X9	TTC23	tetratricopeptide repeat domain 23
		Q9BTX7	TTPAL	alpha tocopherol transfer protein like
		Q9Y6I4	USP3	ubiquitin specific peptidase 3
		P62068	USP46	ubiquitin specific peptidase 46
		Q9C0D7	ZC3H12C	zinc finger CCCH-type containing 12C
		Q9NYG2	ZDHHC3	zinc finger DHHC-type containing 3
		Q15911	ZFHX3	zinc finger homeobox 3
		Q96KR1	ZFR	zinc finger RNA binding protein
		Q7Z3T8	ZFYVE16	zinc finger FYVE-type containing 16
		P17014	ZNF12	zinc finger protein 12
		O14771	ZNF213	zinc finger protein 213
		P17028	ZNF24	zinc finger protein 24
		Q9Y462	ZNF711	zinc finger protein 711
		Q17R98	ZNF827	zinc finger protein 827
		Q49A17	GALNTL6	Polypeptide N-acetylgalactosaminyltransferase-like 6 (EC 2.4.1.41) (Polypeptide GalNAc transferase 17) (GalNAc-T17) (pp-GaNTase 17) (Protein-UDP acetylgalactosaminyltransferase 17) (Putative polypeptide N-acetylgalactosaminyltransferase 17) (UDP-GalNAc:polypeptide N-acetylgalactosaminyltransferase 17)
**TargetScan**	**miR-551a Downregulated 6 hours**	Q9NQ87	HEYL	Hairy/enhancer-of-split related with YRPW motif-like protein (hHeyL) (Class B basic helix-loop-helix protein 33) (bHLHb33) (Hairy-related transcription factor 3) (HRT-3) (hHRT3)
		O94910	ADGRL1	adhesion G protein-coupled receptor L1

miRDB		Q5IJ48	CRB2	crumbs cell polarity complex component 2
		Q15303	ERBB4	erb-b2 receptor tyrosine kinase 4
		Q9NRS4	TMPRSS4	transmembrane serine protease 4
		P16415	ZFP36	ZFP36 ring finger protein

Table S3. Risk evaluation of miRNA levels at baseline in disease progression (at 30 and 60 days) and death according to BCLC stage in the study cohort (n=36; BCLC-B n=9; BCLC-C n=27). Hazard Ratio models were adjusted to prognostic factors Child-Pugh and Performance Status (PS) stages. Hazard Ratio, HR; Confidence Intervals, CI; *p*, *p*-value.

BCLC	Event	miRNA	Prognostic factor	HR, CI (95%)	*p*
**BCLC-B**	*ns*				
**BCLC-C**	**Progression 30 days**	miR-194-5p	none	1.0041 (1.0003-1.0079)	*p*=0.0348
	**Death**	miR-194-5p	Child-Pugh/PS Stage	0.8670 (0.7536-0.9973)	*p*=0.0458
		miR-200c-3p	Child-Pugh/PS Stage	0.7710 (0.5994-0.9917)	*p*=0.0429
		miR-505-5p	Child-Pugh/PS Stage	0.3751 (0.1662-0.8465)	*p*=0.0182

miR-XXX, Baseline miRNA value; *ns*, non-significant

Table S4. Risk evaluation of miRNA time-dependent values upon Sorafenib treatment in disease progression (at 30 and 60 days) and death according to BCLC stage in the study cohort (n=36; BCLC-B n=9; BCLC-C n=27). Hazard Ratio models were adjusted to prognostic factors Child-Pugh and Performance Status (PS) stages. Hazard Ratio, HR; Confidence Intervals, CI; *p*, *p*-value.

BCLC	Event	miRNA	Prognostic factor	HR, CI (95%)	*p*
**BCLC-B**	*Ns*				
**BCLC-C**	**Progression 30 days**	miR-194-5p	PS Stage	1.0040 (1.0005-1.0080)	*p*=0.0256
		miR-375	PS Stage	1.0230 (1.0004-1.0460)	*p*=0.0457
	**Progression 60 days**	*ns*			
	**Death**	miR-194-5p	PS Stage	1.0020 (1.0001-1.0030)	*p*=0.0423
		miR-222-5p	PS Stage	1.0830 (1.0078-1.1640)	*p*=0.0299
		miR-512-3p	PS Stage	1.0660 (1.0095-1.1260)	*p*=0.0214

miR-XXX, miRNA time-dependent value; *ns*, non-significant

Table S5. Risk evaluation of miRNA levels at baseline in disease progression (at 30 and 60 days) and death according to BCLC stage in the validation cohort (n=81; BCLC-B n=34; BCLC-C n=47). Hazard Ratio models were adjusted to prognostic factors Child-Pugh and Performance Status (PS) stages. Hazard Ratio, HR; Confidence Intervals, CI; *p*, *p*-value.

BCLC	Event	miRNA	Prognostic factor	HR, CI (95%)	*p*
**BCLC-B**	**Progression 30 days**	miR-122-5p	None	1.0886 (1.0071-1.1724)	*p*=0.0322
		miR-193b-3p	Child-Pugh Stage	2.1047 (1.1643-3.8048)	*p*=0.0138
		miR-200c-3p	Child-Pugh Stage	12.4978 (1.9487-80.1549)	*p*=0.0077
		miR-222-5p	Child-Pugh	3.6020 (1.2961-10.0106)	*p*=0.0140
		miR-27a-3p	Child-Pugh Stage	1.2170 (1.0300-1.4381)	*p*=0.0211
		miR-505-5p	Child-Pugh Stage	61371 (1.3443- 2801672036)	*p*=0.0440
	**Progression 60 days**	miR-122-5p	Child-Pugh Stage	1. 0857 (1.0110-1.1658)	*p*=0.0237
		miR-193b-3p	Child-Pugh Stage	2.0347 (1.1498-3.6008)	*p*=0.0147
		miR-200c-3p	Child-Pugh Stage	17.3488 (2.5919-116.1300)	*p*=0.0033
		miR-222-5p	Child-Pugh Stage	3.4207 (1.2698-9.2147)	*p*=0.0150

		miR-27a-3p	Child-Pugh Stage	1.2008 (1.0226-1.4099)	*p*=0.0255
		miR-505-5p	Child-Pugh Stage	302.5600 (2.1436-42705)	*p*=0.0237
	**Death**	miR-148b-3p	Child-Pugh Stage	3.5165 (1.6369-7.5527)	*p*=0.0013
**BCLC-C**	**Progression 30 days**	miR-194-5p	Child-Pugh/PS Stage	1.7422 (1.1580-2.6213)	*p*=0.0077
		miR-222-5p	Child-Pugh/PS Stage	10.0573 (1.1956-84.6014)	*p*=0.0336
		miR-375	Child-Pugh/PS Stage	3.2191 (1.3467-7.6946)	*p*=0.0086
	**Progression 60 days**	miR-194-5p	Child-Pugh/PS Stage	1.4009 (1.0432-1.8813)	*p*=0.0250
		miR-375	Child-Pugh/PS Stage	2.5611 (1.2032-5.4516)	*p*=0.0147
	**Death**	miR-200c-3p	Child-Pugh Stage	0.2205 (0.05046-0.9632)	*p*=0.0445

miR-XXX, Baseline miRNA value

Table S6. Risk evaluation of miRNA time-dependent values upon Sorafenib treatment in disease progression (at 30 and 60 days) and death according to BCLC stage in the validation cohort (n=81; BCLC-B n=34; BCLC-C n=47). Hazard Ratio models were adjusted to prognostic factors Child-Pugh and Performance Status (PS) stages. Hazard Ratio, HR; Confidence Intervals, CI; *p*, *p*-value.

BCLC	Event	miRNA	Prognostic factor	HR, CI (95%)	*p*
**BCLC-B**	*ns*				
**BCLC-C**	**Progression 30 days**	miR-222-5p	Child-Pugh/PS Stage	10.9683 (1.3405-89.7455)	*p*=0.0255
	**Death**	miR-222-5p	Child-Pugh Stage/PS Stage	40.1822 (5.5213-292.4300)	*p*=0.0003
		miR-512-3p	Child-Pugh/PS Stage	50.1449 (1.5718-1599.7400)	*p*=0.0267

miR-XXX, miRNA time-dependent value; *ns*, non-significant

Table S7. Expression data of regulated genes by miR-200c-3p. Direct predicted miRNA targets are highlighted in orange.

		I-miR-200c-3p (Sorafenib-Control)				Transfection Control (Sorafenib-control)			
**Ensembl ID**	**Gene name**	**Base mean**	**log_2 (Fold-Change)**	**p-value**	**p-adj**	**Base mean**	**log_2 (Fold-Change)**	**p-value**	**p-adj**
ENSG00000099860	GADD45B	856.575	3.148	1.231E-67	3.635E-64	556.157	0.380	0.0185	7.703E-01
ENSG00000169688	MT1B	150.877	4.035	5.081E-51	1.000E-47	189.137	0.073	0.3577	9.992E-01
ENSG00000205364	MT1M	132.002	2.439	8.718E-21	1.030E-17	192.340	0.155	0.1702	9.992E-01
ENSG00000275993	SIK1B	45.693	1.738	4.150E-10	1.691E-07	24.322	0.531	0.0016	1.967E-01
ENSG00000210077	MT-TV	216.376	1.388	7.836E-08	2.153E-05	173.938	0.338	0.0624	9.992E-01
ENSG00000103460	TOX3	453.941	0.973	1.458E-07	3.559E-05	325.003	0.292	0.0487	9.992E-01
ENSG00000204388	HSPA1B	311.691	1.070	3.934E-07	8.449E-05	302.114	0.169	0.3006	9.992E-01
ENSG00000132677	RHBG	810.682	0.795	1.569E-06	2.808E-04	550.386	0.331	0.0202	7.780E-01
ENSG00000196756	SNHG17	535.232	0.801	3.619E-06	5.857E-04	350.414	0.460	0.0019	2.170E-01
ENSG00000160972	PPP1R16A	300.748	0.972	8.791E-06	1.251E-03	233.439	0.381	0.0246	8.413E-01
ENSG00000205362	MT1A	154.314	1.046	1.290E-05	1.732E-03	87.935	0.373	0.0364	9.340E-01
ENSG00000198355	PIM3	2235.756	0.749	1.616E-05	2.052E-03	1505.591	0.365	0.0075	5.147E-01
ENSG00000135111	TBX3	1632.003	0.584	1.659E-05	2.066E-03	931.210	0.479	0.0000	2.001E-02
ENSG00000168209	DDIT4	281.018	0.838	3.018E-05	3.496E-03	191.002	0.487	0.0032	3.113E-01
ENSG00000186301	MST1P2	219.707	0.932	3.147E-05	3.554E-03	143.099	0.356	0.0313	8.939E-01
ENSG00000187837	HIST1H1C	1562.764	0.906	3.159E-05	3.554E-03	1156.529	0.157	0.3400	9.992E-01
ENSG00000157827	FMNL2	689.755	0.674	6.307E-05	6.314E-03	446.991	0.294	0.0315	8.940E-01
ENSG00000122085	MTERF4	305.978	0.813	6.964E-05	6.856E-03	193.567	0.158	0.3114	9.992E-01
ENSG00000188529	SRSF10	1316.842	0.566	7.841E-05	7.470E-03	805.400	0.321	0.0125	6.403E-01
ENSG00000267519	AC020916.1	41.889	1.104	8.814E-05	8.199E-03	26.090	0.220	0.2126	9.992E-01
ENSG00000169155	ZBTB43	265.885	0.805	9.125E-05	8.356E-03	176.862	0.396	0.0175	7.566E-01
ENSG00000274605	AL355338.1	109.370	0.983	1.357E-04	1.152E-02	73.643	0.301	0.0940	9.992E-01
ENSG00000176046	NUPR1	515.604	0.702	1.366E-04	1.152E-02	431.528	0.370	0.0078	5.180E-01

ENSG00000113318	MSH3	216.663	0.847	1.518E-04	1.245E-02	137.280	0.073	0.6765	9.992E-01
ENSG00000165416	SUGT1	893.418	0.617	1.511E-04	1.245E-02	601.514	0.198	0.1047	9.992E-01
ENSG00000143153	ATP1B1	3581.934	0.522	1.721E-04	1.383E-02	2520.418	0.206	0.0737	9.992E-01
ENSG00000272888	LINC01578	390.829	0.705	2.140E-04	1.610E-02	254.854	0.294	0.0672	9.992E-01
ENSG00000171970	ZNF57	33.976	1.000	2.317E-04	1.700E-02	23.273	0.119	0.5076	9.992E-01
ENSG00000114127	XRN1	151.073	0.876	2.398E-04	1.748E-02	122.749	0.352	0.0504	9.992E-01
ENSG00000197647	ZNF433	54.525	1.032	2.441E-04	1.769E-02	29.906	0.797	0.0000	8.425E-03
ENSG00000130529	TRPM4	44.749	1.019	2.937E-04	2.006E-02	31.005	0.388	0.0275	8.800E-01
ENSG00000138336	TET1	383.523	0.678	3.139E-04	2.103E-02	270.331	0.173	0.2859	9.992E-01
ENSG00000153914	SREK1	1517.370	0.607	3.196E-04	2.103E-02	1037.944	0.298	0.0139	6.687E-01
ENSG00000182809	CRIP2	137.499	0.889	3.696E-04	2.348E-02	104.342	0.197	0.2793	9.992E-01
ENSG00000116044	NFE2L2	2254.523	0.536	3.864E-04	2.390E-02	1355.198	0.319	0.0117	6.281E-01
ENSG00000204387	C6orf48	1298.937	0.568	4.207E-04	2.549E-02	809.379	0.419	0.0008	1.288E-01
ENSG00000185551	NR2F2	953.358	0.664	4.316E-04	2.577E-02	634.102	0.240	0.0919	9.992E-01
ENSG00000137204	SLC22A7	57.814	0.978	4.721E-04	2.775E-02	40.624	0.215	0.2418	9.992E-01
ENSG00000121067	SPOP	1553.150	0.502	5.150E-04	2.953E-02	1219.345	0.241	0.0714	9.992E-01
ENSG00000152382	TADA1	140.382	0.854	5.434E-04	3.057E-02	87.916	0.194	0.2788	9.992E-01
ENSG00000077463	SIRT6	72.518	0.943	6.391E-04	3.479E-02	58.294	0.249	0.1764	9.992E-01
ENSG00000197905	TEAD4	464.063	0.652	7.855E-04	4.124E-02	319.690	-0.017	0.9039	9.992E-01
ENSG00000108375	RNF43	900.415	0.579	9.276E-04	4.703E-02	500.043	0.446	0.0015	1.925E-01
ENSG00000274976	AC087588.2	33.186	0.882	9.983E-04	4.955E-02	18.771	0.274	0.0862	9.992E-01
ENSG00000163602	RYBP	884.182	0.548	1.039E-03	5.091E-02	660.804	0.416	0.0063	4.640E-01
ENSG00000198563	DDX39B	50.445	0.921	1.046E-03	5.105E-02	32.036	0.333	0.0690	9.992E-01
ENSG00000118246	FASTKD2	376.753	0.615	1.124E-03	5.354E-02	217.249	-0.058	0.7104	9.992E-01
ENSG00000153140	CETN3	2430.659	0.524	1.150E-03	5.456E-02	1722.011	0.213	0.0937	9.992E-01
ENSG00000175575	PAAF1	159.239	0.754	1.215E-03	5.688E-02	113.662	0.208	0.2144	9.992E-01
ENSG00000082438	COBLL1	606.761	0.568	1.218E-03	5.688E-02	413.744	0.132	0.3393	9.992E-01
ENSG00000245848	CEBPA	1193.792	0.523	1.212E-03	5.688E-02	725.931	0.252	0.0518	9.992E-01
ENSG00000081913	PHLPP1	260.444	0.679	1.243E-03	5.757E-02	187.667	0.184	0.2292	9.992E-01

ENSG00000102710	SUPT20H	346.186	0.664	1.266E-03	5.818E-02	205.102	0.283	0.0812	9.992E-01
ENSG00000115718	PROC	596.740	0.541	1.383E-03	6.212E-02	377.204	0.184	0.1799	9.992E-01
ENSG00000171729	TMEM51	499.433	0.611	1.557E-03	6.664E-02	320.638	0.180	0.2495	9.992E-01
ENSG00000189046	ALKBH2	176.602	0.810	1.568E-03	6.688E-02	133.409	0.072	0.6735	9.992E-01
ENSG00000120694	HSPH1	1569.327	0.517	1.626E-03	6.812E-02	1084.010	0.158	0.2390	9.992E-01
ENSG00000163516	ANKZF1	246.999	0.673	1.710E-03	7.062E-02	163.355	0.249	0.1204	9.992E-01
ENSG00000168564	CDKN2AIP	182.576	0.736	1.759E-03	7.191E-02	121.578	0.190	0.2724	9.992E-01
ENSG00000183337	BCOR	325.442	0.703	1.768E-03	7.203E-02	233.971	0.163	0.2845	9.992E-01
ENSG00000129484	PARP2	307.236	0.641	1.804E-03	7.248E-02	216.072	0.104	0.4919	9.992E-01
ENSG00000120616	EPC1	743.153	0.559	1.831E-03	7.331E-02	538.288	0.365	0.0034	3.168E-01
ENSG00000124177	CHD6	467.537	0.674	1.853E-03	7.393E-02	383.736	0.323	0.0290	8.861E-01
ENSG00000171227	TMEM37	623.056	0.682	1.918E-03	7.603E-02	384.189	0.256	0.0919	9.992E-01
ENSG00000165030	NFIL3	448.290	0.556	2.015E-03	7.882E-02	309.309	0.350	0.0212	7.869E-01
ENSG00000186204	CYP4F12	63.061	0.864	2.045E-03	7.948E-02	41.722	0.129	0.4869	9.992E-01
ENSG00000067606	PRKCZ	224.581	0.670	2.268E-03	8.615E-02	162.044	0.103	0.5658	9.992E-01
ENSG00000068120	COASY	678.313	0.549	2.337E-03	8.820E-02	454.750	0.334	0.0187	7.703E-01
ENSG00000103245	NARFL	61.048	0.826	2.653E-03	9.763E-02	42.053	0.183	0.3244	9.992E-01
ENSG00000188290	HES4	213.886	0.751	2.738E-03	9.853E-02	109.974	0.295	0.1016	9.992E-01
ENSG00000102390	PBDC1	411.876	0.578	2.749E-03	9.853E-02	322.286	0.276	0.0491	9.992E-01
ENSG00000198408	MGEA5	505.852	0.550	2.712E-03	9.853E-02	319.967	0.395	0.0075	5.147E-01
ENSG00000116717	GADD45A	546.253	0.510	2.778E-03	9.915E-02	416.814	0.385	0.0067	4.845E-01
ENSG00000128228	SDF2L1	768.675	0.568	2.799E-03	9.951E-02	496.321	0.082	0.5623	9.992E-01
ENSG00000087074	PPP1R15A	156.907	0.706	2.848E-03	1.006E-01	128.727	0.498	0.0058	4.460E-01
ENSG00000174953	DHX36	979.757	0.517	2.906E-03	1.013E-01	635.854	0.118	0.4219	9.992E-01
ENSG00000107554	DNMBP	110.524	0.769	2.931E-03	1.018E-01	84.908	0.129	0.4770	9.992E-01
ENSG00000197329	PELI1	54.899	0.822	3.061E-03	1.048E-01	47.235	-0.096	0.6069	9.992E-01
ENSG00000129347	KRI1	174.728	0.687	3.091E-03	1.055E-01	116.665	0.268	0.1285	9.992E-01
ENSG00000072682	P4HA2	293.765	0.678	3.141E-03	1.069E-01	179.176	0.160	0.3106	9.992E-01
ENSG00000126778	**SIX1**	276.395	0.584	3.302E-03	1.106E-01	187.172	0.302	0.0527	9.992E-01

ENSG00000251562	MALAT1	25480.818	0.503	3.461E-03	1.141E-01	18181.725	0.122	0.3560	9.992E-01
ENSG00000143921	ABCG8	56.616	0.813	3.527E-03	1.157E-01	32.435	0.331	0.0712	9.992E-01
ENSG00000182749	PAQR7	114.208	0.761	3.677E-03	1.197E-01	58.747	-0.199	0.2837	9.992E-01
ENSG00000214753	HNRNPUL2	631.085	0.603	3.695E-03	1.198E-01	383.912	0.060	0.6714	9.992E-01
ENSG00000145703	IQGAP2	913.711	0.512	3.701E-03	1.198E-01	589.530	-0.014	0.9211	9.992E-01
ENSG00000118369	USP35	43.962	0.814	3.822E-03	1.219E-01	33.577	0.131	0.4769	9.992E-01
ENSG00000162664	**ZNF326**	569.566	0.551	3.841E-03	1.220E-01	351.606	0.121	0.4202	9.992E-01
ENSG00000153094	BCL2L11	236.135	0.644	3.867E-03	1.225E-01	140.540	0.432	0.0113	6.232E-01
ENSG00000164180	TMEM161B	221.832	0.689	3.932E-03	1.235E-01	136.321	0.114	0.4963	9.992E-01
ENSG00000184897	H1FX	1001.821	0.535	4.041E-03	1.266E-01	604.391	0.363	0.0048	4.013E-01
ENSG00000140386	SCAPER	223.570	0.704	4.067E-03	1.268E-01	124.668	0.053	0.7552	9.992E-01
ENSG00000170802	**FOXN2**	116.143	0.757	4.619E-03	1.381E-01	83.526	0.204	0.2625	9.992E-01
ENSG00000167081	PBX3	268.593	0.616	4.716E-03	1.407E-01	193.073	0.239	0.1422	9.992E-01
ENSG00000149262	INTS4	129.566	0.711	4.809E-03	1.413E-01	84.962	-0.087	0.6300	9.992E-01
ENSG00000196208	GREB1	418.071	0.520	4.900E-03	1.427E-01	240.691	0.307	0.0413	9.525E-01
ENSG00000119844	AFTPH	389.390	0.532	5.387E-03	1.541E-01	251.844	0.162	0.2686	9.992E-01
ENSG00000267296	CEBPA-AS1	77.895	0.751	5.759E-03	1.619E-01	58.295	-0.003	0.9864	9.995E-01
ENSG00000175745	NR2F1	730.653	0.512	5.773E-03	1.619E-01	426.707	0.134	0.3313	9.992E-01
ENSG00000160888	IER2	824.639	0.546	5.826E-03	1.623E-01	584.136	0.181	0.1770	9.992E-01
ENSG00000097096	SYDE2	39.988	0.773	6.012E-03	1.655E-01	31.510	0.328	0.0716	9.992E-01
ENSG00000170439	METTL7B	72.662	0.761	6.106E-03	1.670E-01	51.201	0.161	0.3825	9.992E-01
ENSG00000141391	PRELID3A	54.358	0.769	6.207E-03	1.680E-01	30.540	-0.128	0.4849	9.992E-01
ENSG00000145390	USP53	196.821	0.659	6.381E-03	1.709E-01	117.455	0.455	0.0129	6.407E-01
ENSG00000011376	LARS2	176.313	0.610	6.479E-03	1.724E-01	100.427	-0.121	0.4885	9.992E-01
ENSG00000278909	AC007608.4	430.924	0.642	6.562E-03	1.734E-01	282.080	0.490	0.0044	3.728E-01
ENSG00000151135	TMEM263	291.700	0.550	7.213E-03	1.864E-01	203.857	0.077	0.6139	9.992E-01
ENSG00000103168	TAF1C	222.281	0.579	7.522E-03	1.923E-01	137.947	0.175	0.2759	9.992E-01
ENSG00000100150	DEPDC5	33.622	0.749	7.782E-03	1.964E-01	22.541	0.419	0.0181	7.636E-01
ENSG00000169249	ZRSR2	87.195	0.733	7.919E-03	1.995E-01	65.773	0.314	0.0858	9.992E-01

ENSG00000060566	CREB3L3	123.744	0.726	8.021E-03	2.012E-01	61.490	0.489	0.0084	5.370E-01
ENSG00000146776	ATXN7L1	66.542	0.721	8.014E-03	2.012E-01	58.033	0.145	0.4292	9.992E-01
ENSG00000237149	ZNF503-AS2	79.260	0.734	8.735E-03	2.144E-01	50.520	0.215	0.2478	9.992E-01
ENSG00000105755	ETHE1	306.004	0.550	8.767E-03	2.144E-01	170.835	0.261	0.0986	9.992E-01
ENSG00000158528	**PPP1R9A**	460.642	0.527	9.125E-03	2.191E-01	314.760	0.108	0.4966	9.992E-01
ENSG00000166140	ZFYVE19	98.151	0.713	9.249E-03	2.212E-01	68.683	0.272	0.1304	9.992E-01
ENSG00000214595	EML6	198.944	0.673	9.369E-03	2.231E-01	114.751	0.090	0.6198	9.992E-01
ENSG00000171723	GPHN	94.995	0.703	9.674E-03	2.272E-01	71.986	-0.026	0.8853	9.992E-01
ENSG00000040487	PQLC2	86.414	0.678	9.720E-03	2.278E-01	49.518	0.188	0.3126	9.992E-01
ENSG00000224272	AC131097.3	68.768	0.717	9.852E-03	2.300E-01	33.239	0.092	0.6159	9.992E-01
ENSG00000111727	HCFC2	45.467	0.726	9.928E-03	2.304E-01	43.845	-0.016	0.9323	9.992E-01
ENSG00000073050	XRCC1	246.506	0.622	1.016E-02	2.326E-01	178.149	0.209	0.2200	9.992E-01
ENSG00000247400	DNAJC3-AS1	126.332	0.671	1.029E-02	2.347E-01	69.728	0.197	0.2790	9.992E-01
ENSG00000094631	HDAC6	223.871	0.566	1.043E-02	2.359E-01	130.659	0.069	0.6778	9.992E-01
ENSG00000180423	HARBI1	33.617	0.713	1.047E-02	2.361E-01	16.232	-0.113	0.5019	9.992E-01
ENSG00000198382	UVRAG	275.983	0.564	1.055E-02	2.361E-01	192.520	0.441	0.0072	5.006E-01
ENSG00000173531	MST1	347.314	0.531	1.061E-02	2.362E-01	206.096	0.409	0.0080	5.253E-01
ENSG00000163032	VSNL1	319.121	0.507	1.091E-02	2.400E-01	234.059	-0.010	0.9442	9.992E-01
ENSG00000067836	ROGDI	155.058	0.613	1.095E-02	2.400E-01	102.742	0.081	0.6442	9.992E-01
ENSG00000144655	CSRNP1	142.747	0.672	1.102E-02	2.404E-01	89.762	0.325	0.0715	9.992E-01
ENSG00000148335	NTMT1	209.408	0.621	1.142E-02	2.442E-01	185.595	0.081	0.6420	9.992E-01
ENSG00000237729	AC002075.2	33.745	0.687	1.152E-02	2.451E-01	22.640	0.048	0.7829	9.992E-01
ENSG00000109911	ELP4	75.119	0.686	1.167E-02	2.462E-01	39.150	-0.006	0.9724	9.992E-01
ENSG00000149503	INCENP	321.191	0.504	1.212E-02	2.524E-01	228.041	0.093	0.5802	9.992E-01
ENSG00000161835	GRASP	39.701	0.704	1.237E-02	2.537E-01	17.427	0.149	0.3775	9.992E-01
ENSG00000179456	ZBTB18	56.648	0.703	1.256E-02	2.549E-01	34.546	0.111	0.5469	9.992E-01
ENSG00000169727	GPS1	787.424	0.510	1.280E-02	2.573E-01	475.577	0.102	0.4289	9.992E-01
ENSG00000016864	GLT8D1	202.458	0.537	1.304E-02	2.599E-01	146.459	0.303	0.0677	9.992E-01
ENSG00000172602	RND1	34.714	0.687	1.314E-02	2.608E-01	18.294	0.252	0.1203	9.992E-01

ENSG00000100764	PSMC1	762.251	0.548	1.346E-02	2.639E-01	417.745	0.079	0.6015	9.992E-01
ENSG00000156239	N6AMT1	52.759	0.687	1.370E-02	2.646E-01	46.290	0.047	0.8018	9.992E-01
ENSG00000102996	MMP15	117.518	0.632	1.367E-02	2.646E-01	61.607	-0.134	0.4617	9.992E-01
ENSG00000139546	TARBP2	186.274	0.620	1.369E-02	2.646E-01	127.045	0.097	0.5613	9.992E-01
ENSG00000110172	CHORDC1	320.046	0.542	1.405E-02	2.659E-01	221.404	0.033	0.8252	9.992E-01
ENSG00000119720	NRDE2	210.710	0.522	1.400E-02	2.659E-01	144.786	0.260	0.1099	9.992E-01
ENSG00000116117	PARD3B	104.087	0.624	1.448E-02	2.697E-01	79.187	0.054	0.7634	9.992E-01
ENSG00000235169	SMIM1	125.129	0.615	1.455E-02	2.704E-01	85.326	0.142	0.4276	9.992E-01
ENSG00000205323	SARNP	95.561	0.657	1.463E-02	2.708E-01	72.063	0.225	0.2172	9.992E-01
ENSG00000140548	ZNF710	73.966	0.683	1.509E-02	2.750E-01	52.413	0.139	0.4530	9.992E-01
ENSG00000124216	SNAI1	35.493	0.661	1.534E-02	2.754E-01	26.061	0.310	0.0809	9.992E-01
ENSG00000186529	CYP4F3	98.496	0.642	1.527E-02	2.754E-01	70.372	0.394	0.0313	8.939E-01
ENSG00000164414	SLC35A1	126.119	0.598	1.617E-02	2.830E-01	56.011	0.158	0.3888	9.992E-01
ENSG00000148219	ASTN2	103.973	0.628	1.628E-02	2.831E-01	67.198	0.045	0.8024	9.992E-01
ENSG00000119446	RBM18	172.001	0.567	1.649E-02	2.832E-01	110.966	0.144	0.4026	9.992E-01
ENSG00000273142	AC073335.2	33.852	0.668	1.700E-02	2.864E-01	17.213	0.153	0.3661	9.992E-01
ENSG00000163214	DHX57	180.167	0.537	1.703E-02	2.866E-01	100.033	0.214	0.2393	9.992E-01
ENSG00000117408	IPO13	48.571	0.667	1.739E-02	2.898E-01	39.519	-0.010	0.9563	9.992E-01
ENSG00000187634	SAMD11	72.934	0.667	1.767E-02	2.913E-01	41.972	0.393	0.0346	9.212E-01
ENSG00000101049	SGK2	159.262	0.551	1.787E-02	2.927E-01	93.042	0.349	0.0471	9.986E-01
ENSG00000198929	NOS1AP	132.521	0.597	1.793E-02	2.929E-01	89.573	0.303	0.0909	9.992E-01
ENSG00000069399	BCL3	304.593	0.522	1.802E-02	2.933E-01	180.604	0.459	0.0032	3.113E-01
ENSG00000224597	SVIL-AS1	147.126	0.581	1.824E-02	2.944E-01	112.775	0.133	0.4477	9.992E-01
ENSG00000141556	TBCD	404.072	0.564	1.908E-02	3.007E-01	249.815	0.119	0.4656	9.992E-01
ENSG00000176371	ZSCAN2	200.283	0.516	1.906E-02	3.007E-01	115.943	0.174	0.3174	9.992E-01
ENSG00000004766	VPS50	37.931	0.658	1.957E-02	3.051E-01	37.108	-0.073	0.6937	9.992E-01
ENSG00000198551	ZNF627	34.951	0.638	1.953E-02	3.051E-01	29.421	0.233	0.2017	9.992E-01
ENSG00000205212	CCDC144NL	30.565	0.650	1.969E-02	3.057E-01	18.387	0.244	0.1549	9.992E-01
ENSG00000167702	KIFC2	37.926	0.656	1.980E-02	3.057E-01	41.303	-0.006	0.9746	9.992E-01

ENSG00000133104	SPART	99.985	0.597	2.067E-02	3.113E-01	64.213	-0.001	0.9968	9.999E-01
ENSG00000033170	FUT8	80.948	0.630	2.105E-02	3.142E-01	48.407	-0.024	0.8987	9.992E-01
ENSG00000173681	BCLAF3	59.142	0.640	2.159E-02	3.189E-01	37.267	0.141	0.4476	9.992E-01
ENSG00000081923	ATP8B1	50.512	0.639	2.164E-02	3.191E-01	27.799	0.329	0.0657	9.992E-01
ENSG00000139998	RAB15	216.671	0.513	2.263E-02	3.257E-01	147.399	0.004	0.9814	9.992E-01
ENSG00000066827	ZFAT	44.376	0.639	2.310E-02	3.296E-01	16.482	0.241	0.1455	9.992E-01
ENSG00000168916	ZNF608	44.117	0.635	2.407E-02	3.349E-01	22.410	0.002	0.9912	9.995E-01
ENSG00000138050	THUMPD2	148.112	0.543	2.391E-02	3.349E-01	72.763	0.091	0.6174	9.992E-01
ENSG00000130158	DOCK6	88.767	0.584	2.446E-02	3.362E-01	52.008	0.260	0.1618	9.992E-01
ENSG00000166439	RNF169	275.310	0.504	2.472E-02	3.376E-01	209.329	0.072	0.6419	9.992E-01
ENSG00000125772	GPCPD1	256.868	0.532	2.540E-02	3.430E-01	143.953	0.396	0.0181	7.636E-01
ENSG00000184979	USP18	37.234	0.623	2.561E-02	3.446E-01	35.038	0.238	0.1965	9.992E-01
ENSG00000059728	MXD1	118.245	0.597	2.690E-02	3.559E-01	85.907	0.357	0.0432	9.716E-01
ENSG00000109103	UNC119	74.846	0.607	2.723E-02	3.593E-01	59.768	0.226	0.2182	9.992E-01
ENSG00000134899	ERCC5	146.572	0.546	2.737E-02	3.600E-01	111.779	-0.247	0.1453	9.992E-01
ENSG00000196456	ZNF775	35.712	0.617	2.769E-02	3.620E-01	19.015	0.272	0.1159	9.992E-01
ENSG00000169683	LRRC45	43.980	0.617	2.769E-02	3.620E-01	24.351	0.047	0.7887	9.992E-01
ENSG00000046647	GEMIN8	41.261	0.619	2.801E-02	3.629E-01	29.194	0.134	0.4545	9.992E-01
ENSG00000162924	REL	142.828	0.542	2.795E-02	3.629E-01	102.767	0.149	0.4179	9.992E-01
ENSG00000100116	GCAT	117.587	0.552	2.872E-02	3.682E-01	79.705	0.286	0.1108	9.992E-01
ENSG00000109099	PMP22	171.674	0.569	2.884E-02	3.683E-01	88.887	0.129	0.4854	9.992E-01
ENSG00000171174	RBKS	106.284	0.562	2.919E-02	3.694E-01	59.571	-0.031	0.8635	9.992E-01
ENSG00000168952	STXBP6	195.996	0.519	2.931E-02	3.694E-01	119.690	0.122	0.4724	9.992E-01
ENSG00000111670	GNPTAB	239.035	0.538	2.985E-02	3.715E-01	162.974	0.039	0.8212	9.992E-01
ENSG00000169692	AGPAT2	42.829	0.606	3.038E-02	3.755E-01	36.207	-0.075	0.6842	9.992E-01
ENSG00000165175	MID1IP1	58.883	0.600	3.188E-02	3.821E-01	37.077	0.198	0.2849	9.992E-01
ENSG00000104957	CCDC130	220.559	0.517	3.257E-02	3.840E-01	131.911	0.112	0.5201	9.992E-01
ENSG00000113916	BCL6	138.393	0.535	3.269E-02	3.847E-01	88.163	0.418	0.0202	7.780E-01
ENSG00000197857	ZNF44	121.340	0.554	3.302E-02	3.855E-01	74.752	0.262	0.1545	9.992E-01

ENSG00000121989	ACVR2A	113.958	0.559	3.308E-02	3.856E-01	77.538	0.243	0.1866	9.992E-01
ENSG00000226742	HSBP1L1	111.201	0.544	3.338E-02	3.885E-01	84.070	0.029	0.8737	9.992E-01
ENSG00000011243	AKAP8L	42.749	0.594	3.381E-02	3.912E-01	27.128	0.086	0.6364	9.992E-01
ENSG00000120725	SIL1	145.458	0.551	3.529E-02	4.020E-01	73.593	0.124	0.4906	9.992E-01
ENSG00000188051	TMEM221	34.866	0.591	3.577E-02	4.063E-01	28.360	0.020	0.9105	9.992E-01
ENSG00000184678	HIST2H2BE	32.719	0.553	3.642E-02	4.105E-01	18.752	-0.202	0.2428	9.992E-01
ENSG00000054690	PLEKHH1	33.228	0.583	3.704E-02	4.140E-01	23.038	0.017	0.9244	9.992E-01
ENSG00000106384	MOGAT3	176.778	0.528	3.704E-02	4.140E-01	117.133	0.037	0.8239	9.992E-01
ENSG00000105483	CARD8	102.469	0.554	3.737E-02	4.160E-01	73.444	0.022	0.9027	9.992E-01
ENSG00000173212	MAB21L3	48.138	0.583	3.768E-02	4.176E-01	27.110	0.032	0.8617	9.992E-01
ENSG00000011422	PLAUR	52.453	0.582	3.760E-02	4.176E-01	33.563	0.069	0.7047	9.992E-01
ENSG00000171163	ZNF692	53.259	0.585	3.782E-02	4.177E-01	37.511	-0.069	0.7073	9.992E-01
ENSG00000130173	ANGPTL8	117.716	0.568	3.898E-02	4.211E-01	68.078	0.122	0.5076	9.992E-01
ENSG00000134324	LPIN1	103.579	0.563	3.942E-02	4.221E-01	79.581	0.144	0.4234	9.992E-01
ENSG00000177854	TMEM187	44.786	0.577	4.031E-02	4.256E-01	28.809	0.019	0.9157	9.992E-01
ENSG00000131171	SH3BGRL	41.368	0.577	4.036E-02	4.256E-01	19.418	-0.050	0.7688	9.992E-01
ENSG00000184178	SCFD2	131.522	0.503	4.028E-02	4.256E-01	81.719	0.075	0.6748	9.992E-01
ENSG00000099821	POLRMT	126.405	0.537	4.051E-02	4.262E-01	86.425	-0.054	0.7631	9.992E-01
ENSG00000258457	AL132780.2	47.008	0.576	4.083E-02	4.266E-01	32.602	0.258	0.1558	9.992E-01
ENSG00000157077	ZFYVE9	138.160	0.529	4.113E-02	4.277E-01	107.812	0.075	0.6652	9.992E-01
ENSG00000104375	STK3	44.296	0.570	4.280E-02	4.368E-01	44.829	0.197	0.2890	9.992E-01
ENSG00000120054	CPN1	97.075	0.514	4.421E-02	4.415E-01	66.412	-0.117	0.5184	9.992E-01
ENSG00000120370	GORAB	39.109	0.557	4.480E-02	4.451E-01	25.747	0.299	0.0974	9.992E-01
ENSG00000243364	EFNA4	79.668	0.549	4.554E-02	4.499E-01	55.258	-0.116	0.5313	9.992E-01
ENSG00000023892	DEF6	68.112	0.560	4.605E-02	4.525E-01	47.428	0.083	0.6546	9.992E-01
ENSG00000105829	BET1	77.448	0.534	4.672E-02	4.546E-01	61.382	0.259	0.1629	9.992E-01
ENSG00000168874	ATOH8	67.782	0.541	4.728E-02	4.569E-01	43.714	0.157	0.3994	9.992E-01
ENSG00000174989	FBXW8	47.188	0.552	4.877E-02	4.650E-01	22.272	0.356	0.0440	9.762E-01
ENSG00000069667	RORA	66.066	0.533	4.880E-02	4.650E-01	48.957	0.140	0.4512	9.992E-01

ENSG00000125846	ZNF133	101.594	0.521	4.891E-02	4.656E-01	64.503	0.446	0.0159	7.130E-01
ENSG00000115137	DNAJC27	34.053	0.536	4.942E-02	4.665E-01	22.139	-0.154	0.3892	9.992E-01
ENSG00000186020	ZNF529	37.509	0.541	4.958E-02	4.668E-01	29.882	0.061	0.7373	9.992E-01
ENSG00000121671	CRY2	102.336	0.504	4.972E-02	4.668E-01	71.719	0.200	0.2757	9.992E-01
ENSG00000188785	ZNF548	59.294	0.538	5.023E-02	4.685E-01	29.904	0.421	0.0210	7.865E-01
ENSG00000108239	**TBC1D12**	73.454	0.529	5.060E-02	4.696E-01	58.109	0.120	0.5121	9.992E-01
ENSG00000136840	ST6GALNAC4	141.247	0.513	5.059E-02	4.696E-01	88.126	0.232	0.1960	9.992E-01
ENSG00000196646	ZNF136	37.631	0.548	5.176E-02	4.735E-01	29.398	0.222	0.2150	9.992E-01
ENSG00000113070	HBEGF	48.405	0.546	5.209E-02	4.738E-01	29.364	0.135	0.4586	9.992E-01
ENSG00000102743	SLC25A15	55.799	0.538	5.251E-02	4.753E-01	43.645	0.164	0.3762	9.992E-01
ENSG00000169507	SLC38A11	34.926	0.543	5.303E-02	4.771E-01	18.326	-0.009	0.9543	9.992E-01
ENSG00000172331	BPGM	32.836	0.536	5.539E-02	4.857E-01	21.816	-0.155	0.3852	9.992E-01
ENSG00000106100	NOD1	39.228	0.504	5.584E-02	4.881E-01	25.097	0.137	0.4455	9.992E-01
ENSG00000196843	ARID5A	70.931	0.532	5.716E-02	4.965E-01	47.232	-0.203	0.2744	9.992E-01
ENSG00000114745	GORASP1	94.177	0.514	5.825E-02	4.999E-01	60.410	0.245	0.1801	9.992E-01
ENSG00000087116	ADAMTS2	43.264	0.531	5.918E-02	5.044E-01	26.610	0.080	0.6555	9.992E-01
ENSG00000130270	ATP8B3	46.988	0.530	5.990E-02	5.066E-01	32.525	0.015	0.9333	9.992E-01
ENSG00000109323	MANBA	89.174	0.513	5.978E-02	5.066E-01	70.087	-0.192	0.3019	9.992E-01
ENSG00000146232	NFKBIE	105.159	0.505	6.029E-02	5.083E-01	60.035	0.147	0.4262	9.992E-01
ENSG00000162129	CLPB	77.398	0.516	6.102E-02	5.123E-01	62.453	0.283	0.1222	9.992E-01
ENSG00000132256	TRIM5	51.309	0.525	6.224E-02	5.160E-01	34.625	-0.270	0.1461	9.992E-01
ENSG00000149582	TMEM25	41.217	0.521	6.422E-02	5.272E-01	28.448	-0.041	0.8194	9.992E-01
ENSG00000189057	FAM111B	368.557	-0.360	6.473E-02	5.279E-01	231.327	-0.683	0.0000	1.247E-02
ENSG00000215067	ALOX12-AS1	45.722	0.519	6.506E-02	5.286E-01	26.697	-0.081	0.6496	9.992E-01
ENSG00000239382	ALKBH6	56.164	0.513	6.778E-02	5.374E-01	39.248	-0.181	0.3303	9.992E-01
ENSG00000175895	PLEKHF2	40.103	0.514	6.788E-02	5.378E-01	44.350	-0.050	0.7863	9.992E-01
ENSG00000112367	FIG4	39.292	0.509	6.831E-02	5.404E-01	34.253	0.206	0.2661	9.992E-01
ENSG00000184903	**IMMP2L**	49.095	0.509	7.073E-02	5.454E-01	29.543	0.031	0.8651	9.992E-01
ENSG00000132563	REEP2	49.935	0.506	7.171E-02	5.476E-01	31.905	0.158	0.3885	9.992E-01

ENSG00000143995	MEIS1	30.631	0.503	7.191E-02	5.478E-01	20.914	0.234	0.1797	9.992E-01
ENSG00000240616	RPS6P25	42.573	0.501	7.533E-02	5.592E-01	31.196	0.086	0.6350	9.992E-01
ENSG00000120675	DNAJC15	329.355	-0.299	1.309E-01	6.615E-01	194.121	-0.544	0.0011	1.582E-01
ENSG00000006576	**PHTF2**	96.604	-0.172	5.086E-01	8.956E-01	67.740	-0.542	0.0032	3.113E-01

(Direct) predicted targets

Table S8. STRING clusters analysis of targets regulated by miR-200c-3p.

Cluster number	Protein name
1	ABCG8
1	ADAMTS2
1	AFTPH
1	AGPAT2
1	ATP1B1
1	ATP8B1
1	ATP8B3
1	C19orf80
1	CDKN2AIP
1	CETN3
1	COASY
1	COBLL1
1	CYP4F12
1	CYP4F3
1	DDX39B
1	DEF6
1	DHX57
1	DOCK6
1	FMNL2
1	FUT8
1	GCAT
1	GEMIN8
1	GPHN
1	GPS1
1	GRASP
1	GREB1
1	HARBI1
1	HES4
1	HSBP1L1
1	LPIN1
1	MAB21L3
1	MEIS1
1	MOGAT3
1	NOS1AP
1	P4HA2
1	PBX3
1	PHTF2
1	PIM3
1	PLEKHF2
1	RAB15
1	REEP2

1	SAMD11
1	SARNP
1	SCAPER
1	SLC22A7
1	SLC35A1
1	SLC38A11
1	SLMO1
1	SMIM1
1	SPG20
1	SPOP
1	SREK1
1	SRSF10
1	STK3
1	TARBP2
1	TBC1D12
1	TEAD4
1	TMEM187
1	TMEM51
1	TOX3
1	USP35
1	USP53
1	XRN1
1	ZFAT
1	ZNF326
1	ZNF433
1	ZNF608
1	ZNF627
2	ACVR2A
2	ALKBH2
2	ALKBH6
2	ANKZF1
2	BCL3
2	CARD8
2	CHD6
2	CHORDC1
2	CLPB
2	CPN1
2	CRIP2
2	DNAJC15
2	EFNA4
2	ELP4
2	ERCC5
2	ETHE1

2	FAM111B
2	HDAC6
2	HIST2H2BE
2	HSPA1B
2	HSPH1
2	IMMP2L
2	INTS4
2	KRI1
2	MSH3
2	MST1
2	N6AMT1
2	NFE2L2
2	NFKBIE
2	NOD1
2	NRDE2
2	NTMT1
2	PAAF1
2	PARD3B
2	PARP2
2	PHLPP1
2	PMP22
2	PROC
2	PSMC1
2	SDF2L1
2	SIL1
2	SIRT6
2	SLC25A15
2	SUGT1
2	TBCD
2	THUMPD2
2	TMEM161B
2	TMEM221
2	TMEM263
2	TMEM37
2	TRIM5
2	VSNL1
2	XRCC1
2	ZBTB18
2	ZNF133
2	ZNF44
2	ZNF57
2	ZNF775
3	ARID5A

3	ASTN2
3	ATXN7L1
3	BET1
3	BPGM
3	C6orf48
3	CCDC130
3	CCDC132
3	CCDC144NL
3	DEPDC5
3	DHX36
3	DNAJC27
3	DNMBP
3	EML6
3	EPC1
3	FASTKD2
3	FBXW8
3	GLT8D1
3	GORAB
3	GORASP1
3	H1FX
3	HCFC2
3	HIST1H1C
3	INCENP
3	IPO13
3	KIFC2
3	LARS2
3	LRRC45
3	METTL7B
3	MT1A
3	MT1B
3	MTERF4
3	NARFL
3	NR2F1
3	NUPR1
3	PAQR7
3	PBDC1
3	PLEKHH1
3	POLRMT
3	PPP1R16A
3	PPP1R9A
3	PQLC2
3	PRKCZ
3	RBKS

3	RBM18
3	RNF169
3	ROGDI
3	SCFD2
3	SGK2
3	SH3BGRL
3	SIK1B
3	STXBP6
3	SUPT20H
3	SYDE2
3	TADA1
3	TAF1C
3	TMEM25
3	UNC119
3	ZBTB43
3	ZNF529
3	ZNF710
4	AKAP8L
4	ATOH8
4	BCL2L11
4	BCL6
4	BCOR
4	CEBPA
4	CREB3L3
4	CRY2
4	CSRNP1
4	CXorf23
4	DDIT4
4	FIG4
4	FOXN2
4	GADD45A
4	GADD45B
4	GNPTAB
4	GPCPD1
4	HBEGF
4	HNRNPUL2
4	IER2
4	IQGAP2
4	MANBA
4	MGEA5
4	MID1IP1
4	MMP15
4	MT1M

4	MXD1
4	NFIL3
4	NR2F2
4	PELI1
4	PLAUR
4	PPP1R15A
4	REL
4	RHBG
4	RND1
4	RNF43
4	RORA
4	RYBP
4	SIX1
4	SNAI1
4	ST6GALNAC4
4	TBX3
4	TET1
4	TRPM4
4	USP18
4	UVRAG
4	ZFYVE19
4	ZFYVE9
4	ZNF136
4	ZNF548
4	ZNF692
4	ZRSR2
4	ZSCAN2

Table S9. GO terms and KEGG pathways of STRING clusters of mRNAs regulated by miR-200c-3p.

STRING Cluster	Category	Term	Description	p-value	Genes
1	**GO Term Biological Process**	GO:0019432	triglyceride biosynthetic process	0.00457888	MOGAT3, LPIN1, AGPAT2
	**KEGG Pathway**	hsa04975	Fat digestion and absorption	0.00535349	ABCG8, MOGAT3, AGPAT2
	**KEGG Pathway**	hsa00561	Glycerolipid metabolism	0.01401815	MOGAT3, LPIN1, AGPAT2
	**GO Term Biological Process**	GO:0036101	leukotriene B4 catabolic process	0.01449003	CYP4F3, CYP4F12
	**GO Term Molecular Function**	GO:0050051	leukotriene-B4 20-monooxygenase activity	0.01580528	CYP4F3, CYP4F12
	**KEGG Pathway**	hsa04976	Bile secretion	0.0174584	ABCG8, ATP1B1, SLC22A7
	**GO Term Cellular Component**	GO:0005783	endoplasmic reticulum	0.04191604	PLEKHF2, PHTF2, ATP8B1, P4HA2, REEP2, SLC38A11, AGPAT2, SCAPER

	**GO Term Biological Process**	GO:0006284	base-excision repair	0.01077764	PARP2, XRCC1, SIRT6
	**KEGG Pathway**	hsa04621	NOD-like receptor signaling pathway	0.01604278	CARD8, NOD1, SUGT1
	**GO Term Biological Process**	GO:0036091	positive regulation of transcription from RNA polymerase II promoter in response to oxidative stress	0.01790665	CHD6, NFE2L2
	**GO Term Molecular Function**	GO:0001221	transcription cofactor binding	0.02201624	CHD6, NFE2L2
**2**	**GO Term Molecular Function**	GO:0008276	protein methyltransferase activity	0.02636209	N6AMT1, NTMT1
	**KEGG Pathway**	hsa04141	Protein processing in endoplasmic reticulum	0.02919867	HSPH1, SIL1, HSPA1B, c
	**GO Term Biological Process**	GO:0042981	regulation of apoptotic process	0.03925872	PHLPP1, SDF2L1, CARD8, NOD1
	**GO Term Biological Process**	GO:1900034	regulation of cellular response to heat	0.04272021	HSPH1, CHORDC1, HSPA1B
	**GO Term Molecular Function**	GO:0051787	misfolded protein binding	0.04780884	SDF2L1, HDAC6
**3**	**GO Term Biological Process**	GO:0006390	transcription from mitochondrial promoter	0.04084359	MTERF4, POLRMT

4	**GO Term Biological Process**	GO:0000122	negative regulation of transcription from RNA polymerase II promoter	1.81E-05	CEBPA, RYBP, BCL6, NFIL3, CRY2, SNAI1, REL, SIX1, BCOR, MXD1, ZNF136, TBX3
	**GO Term Molecular Function**	GO:0000977	RNA polymerase II regulatory region sequence-specific DNA binding	3.57E-05	CREB3L3, BCL6, NFIL3, SNAI1, ZNF548, RORA, ZSCAN2
	**GO Term Molecular Function**	GO:0003700	transcription factor activity, sequence-specific DNA binding	5.26E-05	CSRNP1, CEBPA, SIX1, RORA, FOXN2, TBX3, NFIL3, BCL6, ATOH8, ZNF548, REL, MXD1, ZSCAN2

	**GO Term Cellular Component**	GO:0005634	nucleus	8.57E-04	CSRNP1, CEBPA, ZNF692, MT1M, AKAP8L, SIX1, RORA, USP18, CREB3L3, NFIL3, ATOH8, ZNF548, IER2, MGEA5, GADD45B, GADD45A, MID1IP1, TET1, FOXN2, TBX3, BCL6, HNRNPUL2, CRY2, PELI1, REL, SNAI1, BCOR, MXD1, ZNF136, ZSCAN2
	**GO Term Biological Process**	GO:0060021	palate development	0.00153302	CSRNP1, SNAI1, BCOR, TBX3

	**GO Term Molecular Function**	GO:0043565	sequence-specific DNA binding	0.00263852	CSRNP1, BCL6, NFIL3, SIX1, RORA, FOXN2, TBX3
	**GO Term Biological Process**	GO:0006351	transcription, DNA-templated	0.00357154	CEBPA, ZNF692, AKAP8L, TET1, RORA, FOXN2, TBX3, RYBP, BCL6, CRY2, ATOH8, ZNF548, BCOR, MXD1, ZSCAN2
	**GO Term Molecular Function**	GO:0001078	transcriptional repressor activity, RNA polymerase II core promoter proximal region sequence-specific binding	0.0046153	NFIL3, SNAI1, MXD1, TBX3
	**GO Term Biological Process**	GO:0006366	transcription from RNA polymerase II promoter	0.00492488	CEBPA, CSRNP1, CREB3L3, NFIL3, REL, SIX1, ZNF136
	**GO Term Molecular Function**	GO:0003714	transcription corepressor activity	0.00496159	RYBP, NFIL3, BCOR, MXD1, ZNF136

	**GO Term Biological Process**	GO:0006915	apoptotic process	0.01103731	PPP1R15A, CSRNP1, BCL2L11, RYBP, GADD45B, GADD45A, SIX1
	**KEGG Pathway**	hsa04068	FoxO signaling pathway	0.02064351	BCL2L11, GADD45B, BCL6, GADD45A
	**KEGG Pathway**	hsa05202	Transcriptional misregulation in cancer	0.02420337	CEBPA, BCL6, REL, SIX1
	**GO Term Molecular Function**	GO:0001228	transcriptional activator activity, RNA polymerase II transcription regulatory region sequence-specific binding	0.02457282	CEBPA, CSRNP1, CREB3L3
	**GO Term Biological Process**	GO:0045892	negative regulation of transcription, DNA-templated	0.02593007	CEBPA, BCL6, CRY2, ATOH8, BCOR, TBX3
	**GO Term Biological Process**	GO:0032515	negative regulation of phosphoprotein phosphatase activity	0.0293568	PPP1R15A, CRY2
	**GO Term Biological Process**	GO:0030502	negative regulation of bone mineralization	0.0293568	BCOR, TRPM4

	**GO Term Biological Process**	GO:0045944	positive regulation of transcription from RNA polymerase II promoter	0.02961083	CEBPA, CSRNP1, CREB3L3, AKAP8L, REL, TET1, SIX1, RORA
	**GO Term Biological Process**	GO:0045893	positive regulation of transcription, DNA-templated	0.03007585	ATOH8, SNAI1, SIX1, RORA, FOXN2, TBX3
	**GO Term Biological Process**	GO:0035148	tube formation	0.03348111	BCL2L11, ATOH8
	**GO Term Biological Process**	GO:1900745	positive regulation of p38MAPK cascade	0.03758825	GADD45B, GADD45A
	**GO Term Biological Process**	GO:0031065	positive regulation of histone deacetylation	0.0457513	BCL6, AKAP8L
	**GO Term Biological Process**	GO:0035518	histone H2A monoubiquitination	0.0457513	RYBP, BCOR
	**GO Term Biological Process**	GO:0000185	activation of MAPKKK activity	0.0457513	GADD45B, GADD45A

	**GO Term Molecular Function**	GO:0003677	DNA binding	0.04863604	CEBPA, ZNF692, RYBP, NFIL3, CRY2, AKAP8L, SIX1, RORA, ZNF136, IER2, TBX3

Table S10. Expression data of regulated genes by miR-222-5p. Direct predicted miRNA targets are highlighted in orange

		M-miR-222-5p (Sorafenib-Control)				Transfection Control (Sorafenib-Control)			
**Ensembl ID**	**Gene name**	**Base mean**	**log2 (Fold-Change)**	**p-value**	**p-adj**	**Base mean**	**log2 (Fold-Change)**	**p-value**	**p-adj**
ENSG00000210049	MT-TF	4742.611	-1.187	1.970E-09	4.367E-07	4251.805	-0.458	4.789E-02	2.379E-01
ENSG00000175745	NR2F1	525.445	-0.934	3.225E-06	1.963E-04	490.637	-0.330	9.344E-02	3.392E-01
ENSG00000182810	DDX28	117.299	-1.551	5.607E-06	3.108E-04	112.201	-0.426	2.044E-01	5.062E-01
ENSG00000100350	FOXRED2	1060.344	-0.792	8.301E-06	4.316E-04	1038.487	-0.429	2.853E-02	1.788E-01
ENSG00000198938	MT-CO3	107358.661	-0.745	1.059E-05	5.116E-04	109696.284	-0.439	4.087E-03	5.641E-02
ENSG00000109084	TMEM97	1104.867	-0.800	2.648E-05	1.098E-03	1105.823	-0.495	2.077E-02	1.514E-01
ENSG00000102921	N4BP1	237.785	-1.138	2.816E-05	1.146E-03	307.386	-0.399	1.108E-01	3.723E-01
ENSG00000168517	HEXIM2	220.412	-1.154	3.077E-05	1.238E-03	186.252	-0.384	1.626E-01	4.534E-01
ENSG00000189306	RRP7A	366.993	-0.902	3.151E-05	1.263E-03	302.024	-0.472	6.955E-02	2.888E-01
ENSG00000146830	GIGYF1	173.493	-1.221	3.684E-05	1.435E-03	179.492	-0.257	4.089E-01	7.033E-01
ENSG00000174804	FZD4	422.517	-1.025	4.396E-05	1.647E-03	458.381	-0.478	3.178E-02	1.905E-01
ENSG00000100104	SRRD	113.113	-1.421	4.501E-05	1.675E-03	114.801	-0.208	4.768E-01	7.494E-01
ENSG00000177225	GATD1	175.341	-1.137	4.586E-05	1.684E-03	148.995	-0.430	1.465E-01	4.326E-01
ENSG00000124217	MOCS3	68.177	-1.542	5.302E-05	1.858E-03	59.152	-0.274	4.247E-01	7.133E-01
ENSG00000167034	NKX3-1	107.194	-1.279	7.000E-05	2.303E-03	72.147	-0.488	1.494E-01	4.367E-01
ENSG00000173894	CBX2	89.985	-1.282	7.655E-05	2.475E-03	71.295	-0.386	2.764E-01	5.867E-01
ENSG00000107819	SFXN3	107.867	-1.392	1.147E-04	3.454E-03	151.600	-0.450	1.482E-01	4.353E-01
ENSG00000167258	CDK12	955.334	-0.677	1.568E-04	4.357E-03	1044.457	-0.480	1.561E-02	1.278E-01
ENSG00000080839	RBL1	167.826	-1.103	1.627E-04	4.475E-03	195.646	-0.290	3.088E-01	6.185E-01
ENSG00000149948	HMGA2	2654.131	-0.658	1.752E-04	4.722E-03	2011.393	-0.296	1.010E-01	3.556E-01
ENSG00000068654	POLR1A	245.926	-0.891	1.798E-04	4.823E-03	251.565	-0.351	1.803E-01	4.780E-01
ENSG00000213588	ZBTB9	47.149	-1.455	1.899E-04	5.044E-03	50.393	-0.448	2.392E-01	5.465E-01

ENSG00000159202	UBE2Z	1144.733	-0.727	1.984E-04	5.208E-03	1271.739	-0.422	4.840E-02	2.390E-01
ENSG00000064393	HIPK2	616.275	-0.738	2.008E-04	5.257E-03	888.612	-0.385	3.538E-02	2.023E-01
ENSG00000107984	DKK1	562.484	-1.079	2.562E-04	6.384E-03	398.448	-0.364	3.832E-01	6.828E-01
ENSG00000278133	AC135050.6	87.735	-1.187	2.575E-04	6.401E-03	69.214	-0.316	3.641E-01	6.689E-01
ENSG00000100650	SRSF5	933.290	-0.654	2.605E-04	6.447E-03	954.029	-0.306	1.775E-01	4.737E-01
ENSG00000125871	MGME1	336.409	-0.798	2.620E-04	6.461E-03	351.306	-0.274	2.035E-01	5.056E-01
ENSG00000135744	AGT	3897.554	-0.538	2.741E-04	6.723E-03	3829.611	-0.499	5.436E-03	6.731E-02
ENSG00000117632	STMN1	1429.239	-0.791	2.783E-04	6.764E-03	1998.171	-0.268	9.516E-02	3.426E-01
ENSG00000180767	CHST13	805.950	-0.928	2.875E-04	6.942E-03	692.704	-0.271	4.872E-01	7.532E-01
ENSG00000168906	MAT2A	329.206	-0.800	3.312E-04	7.789E-03	311.060	-0.496	8.630E-02	3.250E-01
ENSG00000245848	CEBPA	699.184	-0.713	3.466E-04	8.064E-03	669.885	-0.163	5.099E-01	7.678E-01
ENSG00000108924	HLF	1465.401	-0.660	3.519E-04	8.151E-03	1419.633	-0.436	1.697E-02	1.334E-01
ENSG00000108375	RNF43	407.890	-0.822	3.895E-04	8.779E-03	430.778	-0.426	4.798E-02	2.381E-01
ENSG00000179922	ZNF784	69.718	-1.348	4.139E-04	9.216E-03	43.979	0.046	8.996E-01	9.629E-01
ENSG00000149573	MPZL2	651.151	-0.714	4.448E-04	9.645E-03	478.439	-0.066	7.980E-01	9.188E-01
ENSG00000204444	APOM	369.859	-0.740	4.918E-04	1.044E-02	373.720	-0.266	2.229E-01	5.276E-01
ENSG00000180425	C11orf71	64.617	-1.267	5.379E-04	1.117E-02	64.387	-0.374	3.306E-01	6.380E-01
ENSG00000114491	UMPS	361.368	-0.815	5.535E-04	1.143E-02	329.663	-0.452	5.766E-02	2.633E-01
ENSG00000205730	ITPRIPL2	245.291	-0.887	5.574E-04	1.149E-02	235.247	-0.428	8.122E-02	3.152E-01
ENSG00000123353	ORMDL2	95.118	-1.172	6.184E-04	1.241E-02	151.121	-0.230	4.266E-01	7.144E-01
ENSG00000172831	CES2	717.496	-0.641	6.313E-04	1.254E-02	721.398	-0.392	3.223E-02	1.916E-01
ENSG00000266402	SNHG25	2786.536	-0.627	6.288E-04	1.254E-02	1768.011	-0.448	2.512E-01	5.602E-01
ENSG00000198700	IPO9	542.092	-0.628	6.475E-04	1.277E-02	490.359	-0.161	4.337E-01	7.186E-01
ENSG00000167670	CHAF1A	302.089	-0.754	6.712E-04	1.316E-02	255.734	-0.316	2.396E-01	5.469E-01
ENSG00000123131	PRDX4	468.707	-0.693	7.668E-04	1.451E-02	524.397	-0.028	8.984E-01	9.626E-01
ENSG00000236104	ZBTB22	30.619	-1.462	8.160E-04	1.518E-02	31.731	-0.317	4.244E-01	7.132E-01
ENSG00000003436	TFPI	1513.542	-0.640	8.147E-04	1.518E-02	1417.713	-0.187	2.779E-01	5.882E-01

ENSG00000112146	FBXO9	451.207	-0.726	8.199E-04	1.523E-02	495.009	-0.422	3.676E-02	2.063E-01
ENSG00000185127	C6orf120	108.133	-1.036	8.407E-04	1.556E-02	83.519	-0.414	2.324E-01	5.377E-01
ENSG00000123562	MORF4L2	1129.165	-0.591	8.474E-04	1.566E-02	1331.210	-0.216	2.128E-01	5.165E-01
ENSG00000185219	ZNF445	136.731	-0.955	9.017E-04	1.625E-02	144.674	-0.471	1.259E-01	4.004E-01
ENSG00000210191	MT-TL2	33.987	-1.452	9.574E-04	1.700E-02	27.070	-1.108	8.291E-03	8.718E-02
ENSG00000149357	LAMTOR1	1299.938	-0.593	1.009E-03	1.768E-02	1368.936	-0.270	1.757E-01	4.715E-01
ENSG00000101003	GINS1	178.643	-0.913	1.117E-03	1.905E-02	195.926	-0.445	9.680E-02	3.462E-01
ENSG00000100387	RBX1	687.616	-0.730	1.117E-03	1.905E-02	613.732	-0.411	6.458E-02	2.785E-01
ENSG00000144034	TPRKB	146.367	-1.080	1.141E-03	1.938E-02	255.173	-0.114	6.493E-01	8.485E-01
ENSG00000250799	PRODH2	47.808	-1.324	1.152E-03	1.946E-02	44.969	-0.406	2.977E-01	6.079E-01
ENSG00000169951	ZNF764	49.129	-1.265	1.184E-03	1.981E-02	62.052	0.071	8.458E-01	9.409E-01
ENSG00000181896	ZNF101	106.176	-0.998	1.187E-03	1.982E-02	102.219	-0.246	4.302E-01	7.167E-01
ENSG00000198736	MSRB1	322.260	-0.793	1.195E-03	1.985E-02	356.325	-0.428	8.741E-02	3.279E-01
ENSG00000108679	LGALS3BP	768.639	-0.580	1.201E-03	1.992E-02	656.215	-0.492	8.453E-02	3.213E-01
ENSG00000126062	TMEM115	115.864	-1.022	1.208E-03	1.998E-02	98.272	-0.038	9.069E-01	9.656E-01
ENSG00000134419	RPS15A	6075.332	0.498	1.232E-03	2.024E-02	6136.382	0.521	2.658E-03	4.173E-02
ENSG00000184232	OAF	631.179	-0.596	1.245E-03	2.037E-02	511.316	-0.333	1.378E-01	4.195E-01
ENSG00000101361	NOP56	3480.746	-0.531	1.296E-03	2.095E-02	3274.251	-0.275	1.230E-01	3.936E-01
ENSG00000078142	PIK3C3	121.365	-1.082	1.317E-03	2.122E-02	152.293	-0.322	2.678E-01	5.776E-01
ENSG00000081913	PHLPP1	213.828	-0.854	1.384E-03	2.195E-02	277.579	-0.218	4.152E-01	7.077E-01
ENSG00000117899	MESD	1312.161	-0.602	1.405E-03	2.218E-02	942.079	-0.358	1.472E-01	4.339E-01
ENSG00000103257	SLC7A5	645.677	-0.602	1.425E-03	2.244E-02	1066.055	-0.072	7.809E-01	9.110E-01
ENSG00000125398	SOX9	1224.268	-0.748	1.450E-03	2.271E-02	1021.969	-0.387	5.968E-02	2.691E-01
ENSG00000237649	KIFC1	359.784	-0.778	1.462E-03	2.285E-02	267.084	-0.348	1.540E-01	4.423E-01
ENSG00000130511	SSBP4	243.009	-0.749	1.471E-03	2.292E-02	277.096	-0.250	2.811E-01	5.921E-01
ENSG00000170439	METTL7B	66.914	-1.123	1.512E-03	2.350E-02	66.735	-0.457	2.245E-01	5.291E-01
ENSG00000167700	MFSD3	260.060	-0.854	1.624E-03	2.485E-02	262.235	-0.360	1.961E-01	4.975E-01

ENSG00000166997	CNPY4	39.570	-1.287	1.657E-03	2.515E-02	39.548	-0.076	8.535E-01	9.443E-01
ENSG00000134057	CCNB1	492.148	-0.670	1.660E-03	2.515E-02	501.523	-0.140	5.618E-01	7.999E-01
ENSG00000198734	F5	297.619	-0.759	1.749E-03	2.610E-02	373.899	-0.222	3.125E-01	6.222E-01
ENSG00000250722	SELENOP	2465.808	-0.654	1.767E-03	2.623E-02	1868.120	-0.205	2.675E-01	5.775E-01
ENSG00000114993	RTKN	446.456	-0.631	1.785E-03	2.633E-02	380.410	-0.289	2.513E-01	5.602E-01
ENSG00000177700	POLR2L	1000.450	-0.617	1.803E-03	2.633E-02	943.608	-0.310	1.889E-01	4.878E-01
ENSG00000198642	KLHL9	208.124	-0.856	1.828E-03	2.635E-02	193.724	-0.388	1.803E-01	4.780E-01
ENSG00000130193	THEM6	457.575	-0.687	1.820E-03	2.635E-02	461.962	-0.475	2.935E-02	1.819E-01
ENSG00000015532	XYLT2	78.747	-1.101	1.937E-03	2.736E-02	66.791	-0.477	1.510E-01	4.384E-01
ENSG00000197062	ZSCAN26	44.013	-1.226	1.993E-03	2.791E-02	34.908	-0.157	6.536E-01	8.507E-01
ENSG00000115275	MOGS	242.284	-0.991	1.994E-03	2.791E-02	347.258	-0.429	6.860E-02	2.870E-01
ENSG00000103266	STUB1	312.397	-0.824	2.008E-03	2.807E-02	289.479	0.088	7.485E-01	8.955E-01
ENSG00000170522	ELOVL6	116.074	-0.903	2.132E-03	2.943E-02	111.235	-0.398	1.788E-01	4.755E-01
ENSG00000007968	E2F2	63.955	-1.096	2.150E-03	2.961E-02	49.943	-0.122	7.394E-01	8.920E-01
ENSG00000063322	MED29	86.949	-0.978	2.187E-03	2.997E-02	117.251	-0.254	4.191E-01	7.095E-01
ENSG00000159166	LAD1	416.028	-0.681	2.191E-03	2.998E-02	317.183	0.133	6.385E-01	8.432E-01
ENSG00000244509	APOBEC3C	595.521	-0.555	2.249E-03	3.055E-02	479.772	-0.308	1.213E-01	3.907E-01
ENSG00000102934	PLLP	303.738	-0.817	2.256E-03	3.061E-02	256.481	0.006	9.861E-01	9.945E-01
ENSG00000174851	YIF1A	485.482	-0.667	2.272E-03	3.078E-02	515.426	-0.322	1.632E-01	4.540E-01
ENSG00000224186	C5orf66	31.180	-1.285	2.290E-03	3.094E-02	23.037	-0.282	4.963E-01	7.587E-01
ENSG00000090889	KIF4A	342.431	-0.695	2.316E-03	3.122E-02	374.999	-0.336	1.290E-01	4.049E-01
ENSG00000130770	ATP5IF1	1071.793	-0.531	2.413E-03	3.210E-02	916.487	-0.052	7.703E-01	9.054E-01
ENSG00000161981	SNRNP25	378.961	-0.654	2.422E-03	3.214E-02	360.682	-0.111	6.308E-01	8.398E-01
ENSG00000118785	SPP1	1353.291	-0.700	2.450E-03	3.232E-02	938.999	-0.013	9.507E-01	9.818E-01
ENSG00000174243	DDX23	298.658	-0.753	2.511E-03	3.271E-02	292.728	-0.181	4.606E-01	7.386E-01
ENSG00000117335	CD46	296.189	-0.712	2.550E-03	3.310E-02	260.162	-0.490	3.887E-02	2.127E-01
ENSG00000139291	TMEM19	107.575	-0.933	2.581E-03	3.345E-02	110.915	0.156	5.841E-01	8.131E-01

ENSG00000215271	HOMEZ	33.805	-1.314	2.610E-03	3.368E-02	28.741	-0.936	1.944E-02	1.465E-01
ENSG00000165478	HEPACAM	54.243	-1.214	2.655E-03	3.401E-02	28.123	-0.617	1.352E-01	4.154E-01
ENSG00000103061	SLC7A6OS	317.451	-0.635	2.662E-03	3.407E-02	299.530	0.028	9.075E-01	9.658E-01
ENSG00000272565	RP11-485G4.2	34.164	-1.225	2.796E-03	3.541E-02	27.438	-0.664	1.139E-01	3.779E-01
ENSG00000239306	RBM14	192.790	-0.837	3.019E-03	3.748E-02	216.624	-0.237	3.413E-01	6.489E-01
ENSG00000117601	SERPINC1	225.386	-0.828	3.059E-03	3.782E-02	307.813	-0.201	4.420E-01	7.229E-01
ENSG00000070718	AP3M2	107.936	-1.099	3.141E-03	3.865E-02	121.100	-0.458	1.055E-01	3.631E-01
ENSG00000104442	ARMC1	457.338	-0.652	3.152E-03	3.874E-02	564.634	-0.137	5.586E-01	7.979E-01
ENSG00000278540	ACACA	270.587	-0.710	3.253E-03	3.949E-02	281.619	-0.324	1.648E-01	4.571E-01
ENSG00000090905	TNRC6A	934.726	-0.541	3.292E-03	3.987E-02	883.896	-0.270	1.374E-01	4.188E-01
ENSG00000160209	PDXK	612.500	-0.586	3.317E-03	4.000E-02	509.687	-0.337	9.795E-02	3.485E-01
ENSG00000134575	ACP2	158.680	-0.895	3.365E-03	4.034E-02	164.139	-0.481	1.290E-01	4.049E-01
ENSG00000198888	MT-ND1	32059.948	0.488	3.371E-03	4.034E-02	28784.811	0.589	1.917E-03	3.369E-02
ENSG00000243955	GSTA1	130.878	-0.835	3.463E-03	4.094E-02	112.277	-0.446	1.649E-01	4.571E-01
ENSG00000168802	CHTF8	525.797	-0.582	3.464E-03	4.094E-02	490.433	-0.400	4.929E-02	2.414E-01
ENSG00000185088	RPS27L	3165.038	-0.558	3.452E-03	4.094E-02	3442.531	-0.307	1.877E-01	4.870E-01
ENSG00000166135	HIF1AN	317.493	-0.682	3.607E-03	4.227E-02	176.218	-0.405	1.924E-01	4.932E-01
ENSG00000144045	DQX1	144.372	-0.888	3.798E-03	4.379E-02	111.381	-0.290	3.568E-01	6.628E-01
ENSG00000177971	IMP3	1172.652	-0.730	3.798E-03	4.379E-02	1843.622	-0.470	9.916E-03	9.753E-02
ENSG00000139437	TCHP	216.850	-0.706	3.790E-03	4.379E-02	220.369	-0.381	1.210E-01	3.906E-01
ENSG00000244187	TMEM141	880.169	-0.545	3.830E-03	4.394E-02	766.919	-0.463	1.896E-02	1.440E-01
ENSG00000178988	MRFAP1L1	1274.383	-0.718	3.842E-03	4.403E-02	1453.757	-0.139	7.234E-01	8.855E-01
ENSG00000136478	TEX2	172.154	-0.769	3.877E-03	4.434E-02	166.261	-0.453	1.039E-01	3.607E-01
ENSG00000155850	SLC26A2	101.119	-0.987	4.049E-03	4.582E-02	125.792	-0.358	2.038E-01	5.056E-01
ENSG00000162063	CCNF	267.175	-0.720	4.208E-03	4.687E-02	215.469	-0.393	1.463E-01	4.323E-01
ENSG00000163882	POLR2H	260.069	-0.740	4.310E-03	4.789E-02	351.366	-0.011	9.639E-01	9.849E-01
ENSG00000136848	DAB2IP	174.632	-0.784	4.318E-03	4.793E-02	146.635	-0.280	3.495E-01	6.555E-01

ENSG00000198585	NUDT16	169.204	-0.786	4.354E-03	4.806E-02	166.158	-0.009	9.729E-01	9.894E-01
ENSG00000186468	RPS23	2531.538	0.456	4.353E-03	4.806E-02	2393.495	0.577	7.667E-04	1.901E-02
ENSG00000121068	TBX2	144.357	-0.894	4.373E-03	4.809E-02	164.226	-0.430	1.297E-01	4.063E-01
ENSG00000144283	PKP4	735.669	-0.574	4.407E-03	4.832E-02	838.440	-0.490	3.675E-02	2.063E-01
ENSG00000160606	TLCD1	95.081	-0.941	4.412E-03	4.832E-02	79.782	-0.327	3.001E-01	6.103E-01
ENSG00000205213	LGR4	224.005	-0.741	4.431E-03	4.846E-02	242.480	-0.480	4.696E-02	2.361E-01
ENSG00000125843	AP5S1	175.955	-0.736	4.467E-03	4.863E-02	195.925	-0.370	1.446E-01	4.299E-01
ENSG00000221944	TIGD1	53.014	-1.053	4.496E-03	4.881E-02	49.015	-0.349	3.475E-01	6.535E-01
ENSG00000184117	NIPSNAP1	460.872	-0.624	4.497E-03	4.881E-02	509.808	-0.406	1.216E-01	3.912E-01
ENSG00000161203	AP2M1	641.121	-0.631	4.606E-03	4.970E-02	585.511	-0.076	7.250E-01	8.861E-01
ENSG00000081692	JMJD4	102.015	-0.951	4.655E-03	5.008E-02	120.250	-0.438	1.593E-01	4.503E-01
ENSG00000171161	ZNF672	62.809	-1.081	4.780E-03	5.127E-02	51.192	-0.432	2.658E-01	5.754E-01
ENSG00000125247	TMTC4	137.384	-0.870	4.794E-03	5.131E-02	165.630	-0.250	3.234E-01	6.311E-01
ENSG00000171227	TMEM37	442.910	-0.661	4.792E-03	5.131E-02	510.161	-0.263	3.689E-01	6.726E-01
ENSG00000204237	OXLD1	153.423	-0.885	4.919E-03	5.218E-02	184.455	-0.240	3.356E-01	6.434E-01
ENSG00000198890	PRMT6	54.772	-1.084	4.939E-03	5.225E-02	43.781	-0.226	5.486E-01	7.916E-01
ENSG00000115282	TTC31	144.083	-0.846	4.966E-03	5.234E-02	179.584	-0.432	1.497E-01	4.372E-01
ENSG00000163257	DCAF16	334.586	-0.653	4.966E-03	5.234E-02	406.771	-0.294	1.876E-01	4.870E-01
ENSG00000137038	DMAC1	413.615	-0.601	4.966E-03	5.234E-02	378.452	-0.085	7.000E-01	8.731E-01
ENSG00000099864	PALM	205.969	-0.802	5.090E-03	5.338E-02	269.261	-0.349	2.690E-01	5.785E-01
ENSG00000116771	AGMAT	875.458	-0.607	5.089E-03	5.338E-02	990.651	-0.439	5.180E-02	2.484E-01
ENSG00000079432	CIC	104.805	-0.872	5.156E-03	5.392E-02	85.877	-0.275	4.001E-01	6.977E-01
ENSG00000118418	HMGN3	481.107	-0.659	5.229E-03	5.452E-02	542.679	-0.289	1.777E-01	4.738E-01
ENSG00000153904	DDAH1	411.478	-0.596	5.285E-03	5.495E-02	490.272	-0.326	1.433E-01	4.285E-01
ENSG00000157911	PEX10	326.364	-0.664	5.318E-03	5.514E-02	317.206	-0.293	2.721E-01	5.822E-01
ENSG00000095787	WAC	1342.806	0.491	5.376E-03	5.545E-02	1431.072	0.531	2.105E-03	3.582E-02
ENSG00000164087	POC1A	323.997	-0.639	5.491E-03	5.613E-02	343.700	-0.314	2.005E-01	5.028E-01

ENSG00000162521	RBBP4	443.968	-0.559	5.565E-03	5.661E-02	430.611	-0.295	2.072E-01	5.100E-01
ENSG00000231172	AC007099.1	36.141	-1.105	5.572E-03	5.663E-02	6.436	-0.189	5.976E-01	NA
ENSG00000198780	FAM169A	303.677	-0.692	5.587E-03	5.673E-02	270.256	-0.359	1.368E-01	4.180E-01
ENSG00000184857	TMEM186	191.392	-0.846	5.793E-03	5.834E-02	167.236	-0.298	3.232E-01	6.311E-01
ENSG00000130702	LAMA5	387.634	-0.667	5.995E-03	5.965E-02	474.096	-0.390	6.815E-02	2.858E-01
ENSG00000063245	EPN1	642.855	-0.571	6.272E-03	6.150E-02	671.757	-0.187	4.818E-01	7.517E-01
ENSG00000104290	FZD3	64.991	-0.964	6.341E-03	6.185E-02	60.504	-0.230	5.066E-01	7.649E-01
ENSG00000188486	H2AFX	457.254	-0.815	6.358E-03	6.190E-02	313.109	-0.057	8.146E-01	9.256E-01
ENSG00000110011	DNAJC4	54.269	-1.019	6.452E-03	6.233E-02	60.108	0.131	7.260E-01	8.861E-01
ENSG00000156990	RPUSD3	308.819	-0.590	6.445E-03	6.233E-02	306.351	-0.253	2.893E-01	5.997E-01
ENSG00000177963	RIC8A	277.305	-0.647	6.499E-03	6.260E-02	287.409	-0.259	2.307E-01	5.364E-01
ENSG00000186174	BCL9L	263.044	-0.696	6.533E-03	6.271E-02	254.592	-0.435	1.472E-01	4.339E-01
ENSG00000107890	ANKRD26	217.824	-0.810	6.590E-03	6.303E-02	306.569	-0.300	2.286E-01	5.340E-01
ENSG00000259494	MRPL46	345.774	-0.635	6.601E-03	6.303E-02	361.989	-0.137	5.161E-01	7.717E-01
ENSG00000071794	HLTF	142.390	-0.798	6.649E-03	6.343E-02	138.068	-0.413	1.571E-01	4.473E-01
ENSG00000168040	FADD	146.943	-0.755	6.747E-03	6.425E-02	155.344	-0.372	1.548E-01	4.433E-01
ENSG00000163993	S100P	1491.894	0.480	6.803E-03	6.467E-02	1301.268	0.538	7.255E-03	7.927E-02
ENSG00000027847	B4GALT7	155.148	-0.815	6.846E-03	6.482E-02	126.860	-0.368	2.385E-01	5.456E-01
ENSG00000123243	ITIH5	146.765	-0.746	6.854E-03	6.482E-02	171.326	-0.069	7.936E-01	9.176E-01
ENSG00000110200	ANAPC15	121.861	-0.949	6.975E-03	6.550E-02	131.402	-0.335	2.847E-01	5.960E-01
ENSG00000200090	RF00019	36.333	-1.164	7.023E-03	6.583E-02	44.455	-0.186	6.294E-01	8.395E-01
ENSG00000129292	PHF20L1	308.086	-0.693	7.177E-03	6.702E-02	327.583	-0.384	6.643E-02	2.830E-01
ENSG00000104870	FCGRT	556.728	-0.574	7.204E-03	6.713E-02	663.101	-0.181	4.483E-01	7.275E-01
ENSG00000182272	B4GALNT4	90.226	-0.933	7.215E-03	6.718E-02	87.683	-0.162	5.997E-01	8.219E-01
ENSG00000102081	FMR1	234.086	-0.705	7.494E-03	6.935E-02	366.241	-0.449	4.162E-02	2.206E-01
ENSG00000148341	SH3GLB2	369.022	-0.596	7.533E-03	6.962E-02	402.329	-0.485	5.470E-02	2.560E-01
ENSG00000107779	BMPR1A	43.648	-1.021	7.552E-03	6.965E-02	40.236	0.240	5.393E-01	7.874E-01

ENSG00000142039	CCDC97	60.399	-0.969	7.581E-03	6.987E-02	54.330	-0.470	2.019E-01	5.042E-01
ENSG00000258289	CHURC1	90.764	-0.953	7.628E-03	7.023E-02	142.073	-0.367	1.765E-01	4.724E-01
ENSG00000023516	AKAP11	112.566	-0.906	7.783E-03	7.130E-02	116.496	-0.386	1.973E-01	4.986E-01
ENSG00000143921	ABCG8	32.247	-1.101	7.868E-03	7.190E-02	37.328	-0.492	2.147E-01	5.190E-01
ENSG00000167461	RAB8A	396.142	-0.584	7.885E-03	7.199E-02	413.034	-0.283	1.925E-01	4.932E-01
ENSG00000132031	MATN3	110.940	-0.924	8.015E-03	7.269E-02	54.340	0.291	4.392E-01	7.216E-01
ENSG00000166189	HPS6	54.758	-1.032	8.169E-03	7.335E-02	54.465	-0.062	8.747E-01	9.523E-01
ENSG00000143476	DTL	212.620	-0.746	8.147E-03	7.335E-02	227.510	-0.132	6.093E-01	8.279E-01
ENSG00000169714	CNBP	637.179	0.488	8.155E-03	7.335E-02	568.125	0.643	1.078E-03	2.372E-02
ENSG00000176542	USF3	178.535	-0.793	8.351E-03	7.459E-02	253.723	-0.160	4.921E-01	7.559E-01
ENSG00000148356	LRSAM1	88.525	-0.879	8.372E-03	7.462E-02	107.297	-0.156	6.517E-01	8.498E-01
ENSG00000157933	SKI	351.496	-0.557	8.420E-03	7.487E-02	378.367	-0.350	8.589E-02	3.243E-01
ENSG00000066248	NGEF	124.083	-0.798	8.506E-03	7.529E-02	104.801	-0.073	8.113E-01	9.241E-01
ENSG00000103479	RBL2	126.782	-0.802	8.599E-03	7.580E-02	160.278	-0.344	2.147E-01	5.190E-01
ENSG00000137936	BCAR3	29.762	1.120	8.808E-03	7.680E-02	53.676	1.158	1.468E-03	2.810E-02
ENSG00000224870	AL391244.1	151.758	-0.836	8.932E-03	7.740E-02	181.659	-0.361	1.867E-01	4.857E-01
ENSG00000014138	POLA2	67.801	-0.965	8.942E-03	7.742E-02	67.412	-0.433	2.217E-01	5.271E-01
ENSG00000100865	CINP	97.254	-0.877	9.070E-03	7.815E-02	104.268	-0.027	9.344E-01	9.761E-01
ENSG00000226419	SLC16A1-AS1	111.065	-0.898	9.098E-03	7.833E-02	129.307	-0.124	6.599E-01	8.532E-01
ENSG00000166197	NOLC1	956.768	0.470	9.173E-03	7.872E-02	817.115	0.571	1.337E-02	1.160E-01
ENSG00000010626	LRRC23	36.643	-1.078	9.227E-03	7.900E-02	38.590	-0.441	2.819E-01	5.925E-01
ENSG00000175137	SH3BP5L	221.972	-0.763	9.296E-03	7.953E-02	258.316	-0.087	7.762E-01	9.088E-01
ENSG00000164967	RPP25L	113.626	-0.821	9.512E-03	8.080E-02	98.359	-0.185	5.651E-01	8.014E-01
ENSG00000132773	TOE1	146.008	-0.739	9.527E-03	8.080E-02	111.664	0.018	9.577E-01	9.840E-01
ENSG00000115274	INO80B	149.037	-0.915	9.569E-03	8.082E-02	102.111	-0.393	2.985E-01	6.088E-01
ENSG00000106384	MOGAT3	141.953	-0.721	9.575E-03	8.082E-02	153.312	-0.139	6.324E-01	8.407E-01
ENSG00000277972	CISD3	385.957	-0.557	9.873E-03	8.252E-02	355.429	-0.192	3.876E-01	6.866E-01

ENSG00000111596	CNOT2	377.169	-0.521	1.008E-02	8.345E-02	396.880	-0.269	1.977E-01	4.990E-01
ENSG00000108639	SYNGR2	562.772	-0.563	1.022E-02	8.430E-02	996.485	0.071	7.780E-01	9.100E-01
ENSG00000124766	SOX4	534.361	-0.506	1.031E-02	8.493E-02	566.678	-0.080	7.008E-01	8.733E-01
ENSG00000177946	CENPBD1	136.536	-0.954	1.033E-02	8.506E-02	148.119	-0.357	2.678E-01	5.776E-01
ENSG00000171792	RHNO1	180.172	-0.846	1.039E-02	8.542E-02	195.748	-0.448	9.374E-02	3.398E-01
ENSG00000101911	PRPS2	93.202	-0.915	1.049E-02	8.582E-02	74.599	-0.414	2.294E-01	5.348E-01
ENSG00000175782	SLC35E3	335.364	-0.599	1.046E-02	8.582E-02	352.471	-0.319	1.541E-01	4.423E-01
ENSG00000167513	CDT1	228.230	-0.672	1.061E-02	8.639E-02	192.145	-0.259	4.112E-01	7.051E-01
ENSG00000100504	PYGL	192.362	-0.682	1.081E-02	8.729E-02	283.017	-0.367	1.386E-01	4.202E-01
ENSG00000179933	C14orf119	466.001	-0.526	1.085E-02	8.739E-02	470.417	-0.263	1.884E-01	4.873E-01
ENSG00000095002	MSH2	205.352	-0.745	1.091E-02	8.781E-02	258.511	-0.106	6.448E-01	8.462E-01
ENSG00000100410	PHF5A	385.049	-0.525	1.096E-02	8.801E-02	312.266	-0.404	8.384E-02	3.200E-01
ENSG00000121579	NAA50	823.509	0.455	1.096E-02	8.801E-02	779.877	0.507	1.481E-02	1.233E-01
ENSG00000169955	ZNF747	51.382	-0.944	1.116E-02	8.954E-02	58.564	-0.430	2.566E-01	5.668E-01
ENSG00000108840	HDAC5	77.295	-0.912	1.139E-02	9.065E-02	86.012	-0.182	5.842E-01	8.131E-01
ENSG00000116353	MECR	146.854	-0.768	1.151E-02	9.133E-02	182.607	0.370	2.538E-01	5.638E-01
ENSG00000156968	MPV17L	304.627	-0.638	1.153E-02	9.136E-02	288.845	-0.448	6.298E-02	2.751E-01
ENSG00000068366	ACSL4	917.247	-0.654	1.157E-02	9.153E-02	944.067	-0.316	1.474E-01	4.340E-01
ENSG00000105707	HPN	166.552	-0.709	1.162E-02	9.169E-02	206.855	-0.375	1.721E-01	4.676E-01
ENSG00000115365	LANCL1	279.871	-0.687	1.164E-02	9.176E-02	296.063	-0.362	1.205E-01	3.906E-01
ENSG00000112306	RPS12	27721.405	0.489	1.167E-02	9.195E-02	24997.618	0.611	2.985E-04	9.653E-03
ENSG00000166340	TPP1	311.589	-0.603	1.174E-02	9.233E-02	381.172	-0.167	4.715E-01	7.467E-01
ENSG00000140259	MFAP1	376.030	-0.601	1.177E-02	9.247E-02	390.809	-0.274	2.759E-01	5.864E-01
ENSG00000198055	GRK6	158.560	-0.737	1.199E-02	9.378E-02	129.543	-0.125	6.834E-01	8.644E-01
ENSG00000176909	MAMSTR	34.872	-1.041	1.201E-02	9.379E-02	51.527	-0.369	3.340E-01	6.414E-01
ENSG00000136231	IGF2BP3	409.168	-0.552	1.211E-02	9.415E-02	434.610	-0.391	5.023E-02	2.435E-01
ENSG00000119421	NDUFA8	455.071	-0.540	1.218E-02	9.427E-02	547.698	-0.263	1.733E-01	4.693E-01

ENSG00000121060	TRIM25	372.474	-0.669	1.238E-02	9.524E-02	426.720	-0.448	4.481E-02	2.297E-01
ENSG00000197948	FCHSD1	45.427	-1.022	1.248E-02	9.591E-02	56.477	-0.161	6.451E-01	8.463E-01
ENSG00000167680	SEMA6B	141.168	-0.839	1.263E-02	9.646E-02	113.035	-0.146	6.611E-01	8.536E-01
ENSG00000175283	DOLK	68.526	-0.911	1.264E-02	9.650E-02	49.878	-0.328	3.912E-01	6.894E-01
ENSG00000011454	RABGAP1	288.769	-0.603	1.265E-02	9.650E-02	484.806	-0.361	8.836E-02	3.299E-01
ENSG00000086504	MRPL28	32.082	-1.051	1.289E-02	9.773E-02	28.010	-0.559	1.666E-01	4.597E-01
ENSG00000146433	TMEM181	308.637	-0.583	1.296E-02	9.809E-02	258.455	-0.456	6.676E-02	2.835E-01
ENSG00000148396	SEC16A	264.953	-0.578	1.298E-02	9.809E-02	281.182	-0.409	1.477E-01	4.344E-01
ENSG00000155438	NIFK	630.133	0.445	1.303E-02	9.839E-02	594.046	0.591	4.948E-03	6.328E-02
ENSG00000123080	CDKN2C	110.980	-0.813	1.335E-02	1.001E-01	66.910	-0.264	4.367E-01	7.205E-01
ENSG00000205808	PLPP6	53.546	-0.968	1.341E-02	1.004E-01	52.386	-0.212	5.483E-01	7.916E-01
ENSG00000062650	WAPL	393.802	-0.578	1.348E-02	1.009E-01	521.036	0.085	6.543E-01	8.507E-01
ENSG00000183856	IQGAP3	81.377	-0.863	1.375E-02	1.022E-01	59.857	-0.163	6.564E-01	8.519E-01
ENSG00000132383	RPA1	530.253	-0.578	1.375E-02	1.022E-01	490.222	-0.403	6.300E-02	2.751E-01
ENSG00000104427	ZC2HC1A	97.953	-0.884	1.380E-02	1.025E-01	104.059	-0.261	3.872E-01	6.865E-01
ENSG00000120054	CPN1	71.236	-0.878	1.396E-02	1.033E-01	79.749	-0.043	8.999E-01	9.629E-01
ENSG00000001629	ANKIB1	171.887	-0.677	1.398E-02	1.033E-01	166.148	-0.427	1.480E-01	4.350E-01
ENSG00000168569	TMEM223	280.750	-0.621	1.400E-02	1.033E-01	289.249	-0.123	6.168E-01	8.320E-01
ENSG00000167283	ATP5MG	6061.705	0.456	1.407E-02	1.036E-01	5580.421	0.547	1.033E-03	2.317E-02
ENSG00000140365	COMMD4	225.639	-0.669	1.409E-02	1.036E-01	236.064	-0.442	1.449E-01	4.299E-01
ENSG00000145703	IQGAP2	566.894	-0.536	1.427E-02	1.044E-01	743.693	-0.390	5.195E-02	2.487E-01
ENSG00000100023	PPIL2	108.310	-0.740	1.466E-02	1.069E-01	150.779	-0.424	1.527E-01	4.405E-01
ENSG00000087274	ADD1	509.774	-0.524	1.469E-02	1.070E-01	534.723	-0.255	2.160E-01	5.213E-01
ENSG00000174173	TRMT10C	153.624	-0.748	1.477E-02	1.074E-01	135.419	-0.428	1.623E-01	4.528E-01
ENSG00000198743	SLC5A3	70.888	-0.973	1.495E-02	1.084E-01	96.094	-0.353	2.680E-01	5.777E-01
ENSG00000114988	LMAN2L	169.219	-0.734	1.497E-02	1.085E-01	169.706	-0.299	3.302E-01	6.378E-01
ENSG00000069702	TGFBR3	205.300	-0.726	1.498E-02	1.085E-01	238.091	-0.367	2.295E-01	5.350E-01

ENSG00000077514	POLD3	150.429	-0.831	1.505E-02	1.087E-01	144.435	-0.134	6.596E-01	8.532E-01
ENSG00000079785	DDX1	1400.209	-0.531	1.505E-02	1.087E-01	1833.442	-0.219	2.232E-01	5.282E-01
ENSG00000142252	GEMIN7	75.223	-0.828	1.507E-02	1.087E-01	43.830	0.048	8.988E-01	9.628E-01
ENSG00000236287	ZBED5	53.448	-1.011	1.515E-02	1.092E-01	64.041	-0.466	2.551E-01	5.656E-01
ENSG00000225439	BOLA3-AS1	86.763	-0.834	1.516E-02	1.092E-01	105.182	-0.293	3.265E-01	6.345E-01
ENSG00000211445	GPX3	150.323	-0.731	1.518E-02	1.092E-01	167.055	-0.405	1.934E-01	4.942E-01
ENSG00000188242	AC010442.1	48.306	-1.010	1.538E-02	1.104E-01	57.222	-0.396	2.819E-01	5.925E-01
ENSG00000074842	MYDGF	1502.130	0.480	1.542E-02	1.106E-01	1101.915	0.503	6.605E-03	7.511E-02
ENSG00000210112	MT-TM	107.470	-0.879	1.553E-02	1.111E-01	80.472	-0.470	2.260E-01	5.309E-01
ENSG00000169764	UGP2	656.506	0.468	1.561E-02	1.115E-01	671.972	0.553	7.149E-03	7.876E-02
ENSG00000146416	AIG1	389.774	-0.530	1.563E-02	1.115E-01	405.376	-0.448	3.934E-02	2.147E-01
ENSG00000138160	KIF11	202.999	-0.640	1.568E-02	1.116E-01	189.123	-0.106	6.877E-01	8.669E-01
ENSG00000172830	SSH3	125.465	-0.814	1.574E-02	1.120E-01	136.759	-0.142	6.208E-01	8.344E-01
ENSG00000125826	RBCK1	2183.192	0.449	1.593E-02	1.129E-01	2056.165	0.592	2.055E-03	3.538E-02
ENSG00000169047	IRS1	146.630	-0.667	1.629E-02	1.147E-01	159.382	-0.374	1.554E-01	4.445E-01
ENSG00000102996	MMP15	79.091	-0.867	1.673E-02	1.170E-01	74.242	-0.426	2.134E-01	5.170E-01
ENSG00000117906	RCN2	317.541	-0.664	1.728E-02	1.196E-01	209.941	-0.421	1.535E-01	4.419E-01
ENSG00000118137	APOA1	5355.652	-0.531	1.733E-02	1.199E-01	5163.023	-0.296	2.349E-01	5.411E-01
ENSG00000169592	INO80E	327.548	-0.570	1.753E-02	1.209E-01	318.312	-0.142	6.275E-01	8.392E-01
ENSG00000197852	FAM212B	122.208	-0.793	1.761E-02	1.210E-01	122.495	-0.153	5.937E-01	8.183E-01
ENSG00000178772	CPN2	299.269	-0.614	1.760E-02	1.210E-01	284.059	-0.079	7.296E-01	8.882E-01
ENSG00000141756	FKBP10	298.341	-0.711	1.772E-02	1.211E-01	277.022	-0.423	1.751E-01	4.707E-01
ENSG00000204356	NELFE	409.614	-0.526	1.771E-02	1.211E-01	384.694	-0.442	8.234E-02	3.173E-01
ENSG00000118873	RAB3GAP2	189.451	-0.759	1.798E-02	1.224E-01	251.692	0.025	9.260E-01	9.731E-01
ENSG00000138308	PLA2G12B	41.734	-0.932	1.802E-02	1.225E-01	77.900	-0.475	1.590E-01	4.498E-01
ENSG00000037757	MRI1	111.882	-0.768	1.802E-02	1.225E-01	104.816	-0.056	8.667E-01	9.495E-01
ENSG00000090861	AARS	1006.180	0.490	1.811E-02	1.230E-01	1056.293	0.527	5.479E-03	6.747E-02

ENSG00000040633	PHF23	164.304	-0.641	1.838E-02	1.243E-01	182.039	-0.255	3.366E-01	6.445E-01
ENSG00000117054	ACADM	310.812	-0.534	1.844E-02	1.245E-01	348.211	-0.416	6.121E-02	2.725E-01
ENSG00000080618	CPB2	171.028	-0.725	1.859E-02	1.252E-01	154.484	-0.166	5.368E-01	7.866E-01
ENSG00000103174	NAGPA	322.155	-0.530	1.865E-02	1.254E-01	326.161	-0.193	4.729E-01	7.468E-01
ENSG00000126603	GLIS2	29.198	0.997	1.865E-02	1.254E-01	47.053	0.569	1.388E-01	4.208E-01
ENSG00000184207	PGP	294.002	-0.549	1.876E-02	1.260E-01	303.315	0.014	9.513E-01	9.821E-01
ENSG00000146063	TRIM41	110.222	-0.710	1.901E-02	1.268E-01	104.215	-0.485	1.007E-01	3.546E-01
ENSG00000148773	MKI67	1321.006	-0.515	1.902E-02	1.268E-01	1324.531	-0.463	1.708E-02	1.340E-01
ENSG00000088992	TESC	363.472	-0.644	1.914E-02	1.273E-01	211.009	0.186	5.042E-01	7.642E-01
ENSG00000115756	HPCAL1	774.743	0.466	1.935E-02	1.281E-01	687.986	0.598	1.241E-03	2.550E-02
ENSG00000109971	HSPA8	904.679	-0.508	1.942E-02	1.283E-01	993.037	-0.470	5.492E-02	2.564E-01
ENSG00000127564	PKMYT1	282.549	-0.549	1.946E-02	1.285E-01	251.805	0.081	7.698E-01	9.054E-01
ENSG00000037897	METTL1	213.782	-0.659	1.952E-02	1.287E-01	211.973	-0.199	4.874E-01	7.532E-01
ENSG00000197858	GPAA1	416.900	-0.513	1.955E-02	1.288E-01	404.245	-0.269	1.962E-01	4.976E-01
ENSG00000169957	ZNF768	168.355	-0.709	1.999E-02	1.309E-01	125.312	-0.417	1.987E-01	4.997E-01
ENSG00000127220	ABHD8	48.097	-0.915	2.003E-02	1.311E-01	42.246	-0.298	4.476E-01	7.271E-01
ENSG00000106351	AGFG2	212.935	-0.569	2.019E-02	1.319E-01	187.829	-0.179	5.148E-01	7.709E-01
ENSG00000182841	RRP7BP	56.076	-0.879	2.027E-02	1.320E-01	67.099	-0.312	3.832E-01	6.828E-01
ENSG00000272994	AC012360.3	52.627	-0.888	2.058E-02	1.336E-01	86.172	-0.285	4.004E-01	6.978E-01
ENSG00000152147	GEMIN6	132.842	-0.709	2.067E-02	1.341E-01	129.385	-0.203	5.255E-01	7.794E-01
ENSG00000250321	AC079140.2	27.845	-1.012	2.093E-02	1.352E-01	31.601	1.002	1.607E-02	1.298E-01
ENSG00000213463	SYNJ2BP	137.649	-0.779	2.097E-02	1.354E-01	186.556	-0.409	1.273E-01	4.028E-01
ENSG00000198105	ZNF248	57.955	-0.927	2.114E-02	1.362E-01	61.233	-0.450	1.979E-01	4.992E-01
ENSG00000140832	MARVELD3	106.975	-0.776	2.138E-02	1.372E-01	109.868	-0.020	9.509E-01	9.819E-01
ENSG00000054654	SYNE2	194.841	-0.648	2.147E-02	1.376E-01	283.752	-0.424	6.535E-02	2.799E-01
ENSG00000164403	SHROOM1	200.357	-0.648	2.157E-02	1.379E-01	185.291	-0.150	6.107E-01	8.288E-01
ENSG00000085998	POMGNT1	73.342	-0.799	2.175E-02	1.387E-01	76.840	-0.271	4.098E-01	7.035E-01

ENSG00000136720	HS6ST1	108.889	-0.824	2.179E-02	1.388E-01	79.129	-0.383	2.499E-01	5.593E-01
ENSG00000189339	SLC35E2B	161.450	-0.780	2.185E-02	1.388E-01	289.529	-0.498	4.129E-02	2.199E-01
ENSG00000164096	C4orf3	388.696	-0.588	2.205E-02	1.394E-01	400.691	-0.349	1.047E-01	3.618E-01
ENSG00000212978	AC016747.1	128.336	-0.646	2.219E-02	1.402E-01	156.194	-0.457	1.741E-01	4.696E-01
ENSG00000179523	EIF3J-AS1	56.354	-0.930	2.229E-02	1.407E-01	94.076	-0.493	9.900E-02	3.508E-01
ENSG00000181396	OGFOD3	295.746	-0.566	2.248E-02	1.415E-01	333.659	-0.318	1.829E-01	4.819E-01
ENSG00000147804	SLC39A4	424.424	-0.522	2.250E-02	1.416E-01	471.444	-0.166	5.428E-01	7.887E-01
ENSG00000143363	PRUNE1	156.589	-0.681	2.305E-02	1.441E-01	186.207	-0.290	2.904E-01	6.007E-01
ENSG00000139679	LPAR6	37.563	-0.950	2.322E-02	1.448E-01	39.873	-0.300	4.289E-01	7.160E-01
ENSG00000196743	GM2A	277.377	-0.509	2.321E-02	1.448E-01	279.467	-0.326	1.522E-01	4.398E-01
ENSG00000117834	SLC5A9	61.663	-0.817	2.326E-02	1.450E-01	92.880	-0.287	4.227E-01	7.122E-01
ENSG00000198863	RUNDC1	91.206	-0.833	2.330E-02	1.450E-01	140.993	-0.324	2.853E-01	5.965E-01
ENSG00000204220	PFDN6	108.376	-0.756	2.329E-02	1.450E-01	110.361	0.032	9.293E-01	9.744E-01
ENSG00000197165	SULT1A2	174.335	-0.656	2.387E-02	1.477E-01	200.105	-0.293	3.607E-01	6.659E-01
ENSG00000158528	PPP1R9A	302.665	-0.545	2.392E-02	1.477E-01	431.972	-0.261	2.168E-01	5.218E-01
ENSG00000103111	MON1B	306.166	-0.502	2.393E-02	1.477E-01	240.707	-0.493	7.805E-02	3.073E-01
ENSG00000132254	ARFIP2	132.955	-0.716	2.431E-02	1.494E-01	127.561	-0.187	5.420E-01	7.887E-01
ENSG00000034533	ASTE1	44.435	-0.894	2.443E-02	1.499E-01	28.443	-0.260	5.312E-01	7.836E-01
ENSG00000160214	RRP1	162.560	-0.669	2.468E-02	1.505E-01	148.117	-0.186	5.581E-01	7.974E-01
ENSG00000185298	CCDC137	342.116	-0.541	2.465E-02	1.505E-01	301.821	-0.357	1.866E-01	4.857E-01
ENSG00000102786	INTS6	165.934	-0.657	2.489E-02	1.513E-01	191.135	-0.221	4.217E-01	7.118E-01
ENSG00000249115	HAUS5	60.716	-0.804	2.492E-02	1.514E-01	72.830	-0.422	2.060E-01	5.086E-01
ENSG00000248092	NNT-AS1	186.502	-0.589	2.527E-02	1.530E-01	195.855	-0.167	5.365E-01	7.866E-01
ENSG00000176623	RMDN1	229.849	-0.599	2.534E-02	1.531E-01	242.350	-0.401	1.435E-01	4.289E-01
ENSG00000175567	UCP2	158.963	-0.581	2.574E-02	1.550E-01	140.715	-0.214	5.164E-01	7.719E-01
ENSG00000164850	GPER1	226.859	-0.627	2.577E-02	1.551E-01	231.944	-0.310	2.100E-01	5.130E-01
ENSG00000137944	KYAT3	95.800	-0.787	2.584E-02	1.554E-01	116.126	-0.131	6.549E-01	8.510E-01

ENSG00000130347	RTN4IP1	61.219	-0.884	2.589E-02	1.556E-01	49.621	-0.204	5.974E-01	8.202E-01
ENSG00000112167	SAYSD1	108.118	-0.688	2.610E-02	1.564E-01	127.327	-0.094	7.343E-01	8.898E-01
ENSG00000106665	CLIP2	44.005	-0.895	2.616E-02	1.566E-01	38.750	-0.373	3.355E-01	6.433E-01
ENSG00000103066	PLA2G15	110.953	-0.664	2.618E-02	1.566E-01	92.698	0.100	7.854E-01	9.132E-01
ENSG00000064545	TMEM161A	384.607	-0.555	2.655E-02	1.581E-01	350.101	0.059	8.333E-01	9.345E-01
ENSG00000130312	MRPL34	770.411	0.472	2.665E-02	1.584E-01	619.742	0.518	2.809E-02	1.769E-01
ENSG00000114125	RNF7	378.653	0.457	2.680E-02	1.589E-01	315.866	0.551	1.985E-02	1.480E-01
ENSG00000149179	C11orf49	58.495	-0.854	2.699E-02	1.595E-01	53.009	-0.028	9.391E-01	9.777E-01
ENSG00000198919	DZIP3	106.130	-0.692	2.708E-02	1.595E-01	183.765	-0.193	4.819E-01	7.517E-01
ENSG00000187017	ESPN	216.681	-0.577	2.709E-02	1.595E-01	163.434	-0.491	8.781E-02	3.285E-01
ENSG00000133226	SRRM1	3043.154	0.396	2.707E-02	1.595E-01	3046.631	0.531	3.656E-03	5.202E-02
ENSG00000171135	JAGN1	316.512	-0.579	2.745E-02	1.606E-01	333.383	-0.450	5.636E-02	2.600E-01
ENSG00000164171	ITGA2	127.500	-0.760	2.754E-02	1.609E-01	155.199	-0.369	1.832E-01	4.824E-01
ENSG00000046647	GEMIN8	47.821	-0.847	2.766E-02	1.613E-01	45.822	-0.233	5.488E-01	7.916E-01
ENSG00000055163	CYFIP2	277.033	-0.549	2.783E-02	1.617E-01	314.235	-0.196	4.374E-01	7.205E-01
ENSG00000134905	CARS2	228.193	-0.566	2.818E-02	1.627E-01	195.182	-0.236	3.627E-01	6.674E-01
ENSG00000213462	ERV3-1	108.485	-0.752	2.823E-02	1.629E-01	148.855	-0.302	2.788E-01	5.894E-01
ENSG00000147099	HDAC8	99.212	-0.764	2.866E-02	1.646E-01	92.337	-0.326	3.205E-01	6.294E-01
ENSG00000175832	ETV4	424.060	-0.519	2.872E-02	1.648E-01	291.207	0.139	6.017E-01	8.236E-01
ENSG00000199753	SNORD104	41.144	-0.848	2.884E-02	1.652E-01	37.467	-0.269	4.860E-01	7.530E-01
ENSG00000137274	BPHL	84.001	-0.848	2.910E-02	1.661E-01	84.778	-0.379	2.867E-01	5.977E-01
ENSG00000137040	RANBP6	77.519	-0.718	2.924E-02	1.666E-01	92.672	-0.007	9.833E-01	9.929E-01
ENSG00000131323	TRAF3	104.237	-0.686	2.946E-02	1.671E-01	112.890	0.046	8.763E-01	9.525E-01
ENSG00000113161	HMGCR	519.452	-0.538	2.964E-02	1.680E-01	480.221	-0.459	2.322E-02	1.607E-01
ENSG00000168734	PKIG	169.354	-0.595	2.969E-02	1.682E-01	226.800	-0.479	7.196E-02	2.939E-01
ENSG00000141655	TNFRSF11A	49.071	-0.808	2.972E-02	1.683E-01	38.010	-0.417	2.986E-01	6.088E-01
ENSG00000145912	NHP2	205.004	-0.528	3.003E-02	1.694E-01	199.188	-0.026	9.227E-01	9.716E-01

ENSG00000130713	EXOSC2	140.703	-0.730	3.039E-02	1.705E-01	130.682	-0.293	3.333E-01	6.408E-01
ENSG00000165868	HSPA12A	32.118	-0.915	3.043E-02	1.706E-01	32.548	-0.258	5.183E-01	7.737E-01
ENSG00000006118	TMEM132A	38.681	-0.866	3.042E-02	1.706E-01	46.367	-0.416	2.709E-01	5.807E-01
ENSG00000178105	DDX10	495.063	0.497	3.045E-02	1.706E-01	510.425	0.694	1.306E-03	2.611E-02
ENSG00000096080	MRPS18A	252.883	-0.615	3.056E-02	1.710E-01	230.826	-0.395	1.556E-01	4.447E-01
ENSG00000033011	ALG1	302.680	-0.567	3.066E-02	1.712E-01	280.413	-0.332	1.478E-01	4.348E-01
ENSG00000060762	MPC1	381.499	-0.512	3.080E-02	1.718E-01	331.937	-0.256	2.665E-01	5.760E-01
ENSG00000153767	GTF2E1	58.019	-0.773	3.097E-02	1.721E-01	61.215	0.124	7.299E-01	8.882E-01
ENSG00000107789	MINPP1	184.666	-0.618	3.099E-02	1.721E-01	158.241	-0.237	4.146E-01	7.075E-01
ENSG00000143971	ETAA1	42.828	-0.843	3.126E-02	1.733E-01	34.612	-0.149	7.203E-01	8.834E-01
ENSG00000087338	GMCL1	232.649	-0.525	3.165E-02	1.749E-01	297.536	-0.448	5.335E-02	2.524E-01
ENSG00000104695	PPP2CB	677.340	0.446	3.167E-02	1.750E-01	597.073	0.553	5.467E-03	6.746E-02
ENSG00000121274	PAPD5	1510.327	0.437	3.194E-02	1.758E-01	2102.661	0.718	2.155E-03	3.624E-02
ENSG00000276293	PIP4K2B	308.825	-0.526	3.247E-02	1.776E-01	172.970	-0.488	1.616E-01	4.519E-01
ENSG00000198246	SLC29A3	289.183	-0.515	3.257E-02	1.778E-01	254.164	-0.424	9.687E-02	3.462E-01
ENSG00000149930	TAOK2	123.695	-0.711	3.278E-02	1.781E-01	140.106	-0.180	5.332E-01	7.846E-01
ENSG00000180822	PSMG4	304.129	-0.512	3.301E-02	1.790E-01	271.716	-0.184	4.854E-01	7.530E-01
ENSG00000134291	TMEM106C	249.667	-0.592	3.311E-02	1.793E-01	228.368	-0.286	2.684E-01	5.781E-01
ENSG00000119227	PIGZ	39.663	-0.920	3.317E-02	1.796E-01	51.041	-0.301	4.426E-01	7.229E-01
ENSG00000134824	FADS2	134.362	-0.750	3.334E-02	1.799E-01	109.479	-0.411	2.605E-01	5.702E-01
ENSG00000119559	C19orf25	114.328	-0.708	3.357E-02	1.805E-01	142.186	-0.253	4.117E-01	7.054E-01
ENSG00000163684	RPP14	98.285	-0.714	3.362E-02	1.807E-01	95.866	-0.131	6.688E-01	8.574E-01
ENSG00000124201	ZNFX1	346.998	-0.572	3.404E-02	1.819E-01	324.341	-0.498	8.235E-02	3.173E-01
ENSG00000185379	RAD51D	109.398	-0.689	3.407E-02	1.819E-01	98.081	-0.047	8.843E-01	9.556E-01
ENSG00000127774	EMC6	714.306	-0.551	3.419E-02	1.823E-01	1419.846	0.026	8.803E-01	9.541E-01
ENSG00000174990	CA5A	41.115	-0.861	3.423E-02	1.824E-01	80.234	-0.310	3.950E-01	6.935E-01
ENSG00000130717	UCK1	64.635	-0.751	3.430E-02	1.826E-01	60.042	-0.431	2.210E-01	5.265E-01

ENSG00000110871	COQ5	182.058	-0.597	3.448E-02	1.832E-01	195.494	-0.438	1.218E-01	3.915E-01
ENSG00000158402	CDC25C	276.472	-0.518	3.484E-02	1.845E-01	253.233	-0.489	6.913E-02	2.880E-01
ENSG00000105953	OGDH	67.942	-0.771	3.497E-02	1.850E-01	91.661	-0.385	2.196E-01	5.249E-01
ENSG00000167880	EVPL	74.909	-0.713	3.503E-02	1.850E-01	66.677	0.024	9.432E-01	9.789E-01
ENSG00000172922	RNASEH2C	191.339	-0.540	3.522E-02	1.856E-01	208.417	-0.346	2.006E-01	5.028E-01
ENSG00000143819	EPHX1	109.300	-0.755	3.554E-02	1.867E-01	108.982	-0.349	3.230E-01	6.311E-01
ENSG00000197070	ARRDC1	32.776	-0.893	3.570E-02	1.869E-01	22.017	-0.005	9.898E-01	NA
ENSG00000135632	SMYD5	197.489	-0.576	3.577E-02	1.872E-01	182.584	-0.384	1.855E-01	4.846E-01
ENSG00000171155	C1GALT1C1	62.414	-0.831	3.582E-02	1.872E-01	38.453	-0.026	9.466E-01	9.804E-01
ENSG00000157557	ETS2	693.478	0.425	3.582E-02	1.872E-01	653.441	0.604	1.194E-03	2.509E-02
ENSG00000132004	FBXW9	78.732	-0.692	3.587E-02	1.873E-01	110.767	-0.237	4.549E-01	7.339E-01
ENSG00000118762	PKD2	68.088	-0.796	3.609E-02	1.879E-01	55.149	-0.406	2.629E-01	5.730E-01
ENSG00000137700	SLC37A4	160.008	-0.583	3.632E-02	1.883E-01	149.021	0.181	5.345E-01	7.854E-01
ENSG00000167113	COQ4	155.577	-0.565	3.648E-02	1.887E-01	146.547	-0.049	8.552E-01	9.448E-01
ENSG00000054611	TBC1D22A	192.933	-0.538	3.649E-02	1.887E-01	268.889	-0.287	2.865E-01	5.976E-01
ENSG00000058668	ATP2B4	30.532	-0.888	3.672E-02	1.891E-01	19.193	-0.101	8.101E-01	NA
ENSG00000128989	ARPP19	416.363	0.418	3.673E-02	1.891E-01	376.869	0.565	9.881E-03	9.744E-02
ENSG00000187796	CARD9	34.609	-0.900	3.736E-02	1.913E-01	29.200	0.073	8.605E-01	9.475E-01
ENSG00000188211	NCR3LG1	90.039	-0.689	3.744E-02	1.916E-01	81.504	-0.164	6.552E-01	8.510E-01
ENSG00000148219	ASTN2	88.013	-0.654	3.829E-02	1.948E-01	87.362	-0.237	4.484E-01	7.275E-01
ENSG00000179761	PIPOX	324.868	-0.516	3.827E-02	1.948E-01	280.829	-0.319	1.525E-01	4.401E-01
ENSG00000033100	CHPF2	63.586	-0.731	3.843E-02	1.950E-01	66.071	-0.221	5.276E-01	7.813E-01
ENSG00000155008	APOOL	158.972	-0.600	3.842E-02	1.950E-01	130.584	-0.422	1.595E-01	4.503E-01
ENSG00000167693	NXN	639.912	0.415	3.861E-02	1.954E-01	646.955	0.597	7.076E-03	7.823E-02
ENSG00000130560	UBAC1	303.966	-0.514	3.865E-02	1.955E-01	344.809	-0.326	1.542E-01	4.423E-01
ENSG00000115364	MRPL19	361.287	0.452	3.869E-02	1.956E-01	328.963	0.647	1.651E-02	1.314E-01
ENSG00000197879	MYO1C	311.289	-0.619	3.900E-02	1.963E-01	340.160	-0.300	2.356E-01	5.420E-01

ENSG00000181523	SGSH	39.471	-0.834	3.906E-02	1.965E-01	44.591	-0.467	2.354E-01	5.418E-01
ENSG00000198142	SOWAHC	231.074	-0.528	3.917E-02	1.969E-01	334.800	-0.262	2.423E-01	5.500E-01
ENSG00000139266	MARCH9	33.862	-0.838	3.961E-02	1.984E-01	45.071	-0.374	3.213E-01	6.294E-01
ENSG00000177106	EPS8L2	553.169	-0.507	3.969E-02	1.987E-01	602.887	-0.420	7.790E-02	3.072E-01
ENSG00000101544	ADNP2	399.843	0.457	4.023E-02	2.011E-01	407.375	0.586	1.271E-02	1.131E-01
ENSG00000115750	TAF1B	96.812	-0.650	4.056E-02	2.017E-01	139.300	-0.339	2.180E-01	5.232E-01
ENSG00000198468	FLVCR1-AS1	52.715	-0.743	4.077E-02	2.024E-01	57.896	-0.197	5.848E-01	8.136E-01
ENSG00000113594	LIFR	95.445	-0.737	4.091E-02	2.029E-01	121.070	-0.081	7.935E-01	9.176E-01
ENSG00000071462	BUD23	428.116	0.409	4.106E-02	2.033E-01	392.357	0.704	1.391E-03	2.711E-02
ENSG00000101004	NINL	202.551	-0.548	4.157E-02	2.050E-01	215.243	-0.131	6.683E-01	8.572E-01
ENSG00000198324	FAM109A	123.813	-0.626	4.171E-02	2.056E-01	120.683	-0.179	6.037E-01	8.250E-01
ENSG00000176155	CCDC57	75.224	-0.733	4.179E-02	2.059E-01	85.515	-0.285	4.086E-01	7.033E-01
ENSG00000111665	CDCA3	350.109	-0.504	4.200E-02	2.067E-01	216.060	-0.266	3.120E-01	6.220E-01
ENSG00000129810	SGO1	83.838	-0.729	4.206E-02	2.067E-01	95.329	0.098	7.571E-01	9.007E-01
ENSG00000103423	DNAJA3	224.051	-0.514	4.213E-02	2.067E-01	220.330	-0.082	7.701E-01	9.054E-01
ENSG00000189266	PNRC2	183.661	-0.529	4.229E-02	2.069E-01	179.200	-0.125	6.924E-01	8.684E-01
ENSG00000153208	MERTK	170.384	-0.532	4.242E-02	2.074E-01	111.641	-0.327	2.729E-01	5.826E-01
ENSG00000171861	MRM3	83.923	-0.841	4.263E-02	2.080E-01	86.549	0.054	8.615E-01	9.475E-01
ENSG00000253719	ATXN7L3B	322.270	-0.500	4.288E-02	2.090E-01	344.587	-0.191	4.043E-01	7.008E-01
ENSG00000135164	DMTF1	53.620	-0.752	4.345E-02	2.108E-01	60.413	-0.437	2.218E-01	5.272E-01
ENSG00000135211	TMEM60	73.100	-0.677	4.397E-02	2.126E-01	77.204	-0.314	3.346E-01	6.422E-01
ENSG00000147536	GINS4	99.889	-0.672	4.470E-02	2.141E-01	91.224	0.423	1.924E-01	4.932E-01
ENSG00000025434	NR1H3	93.676	-0.622	4.489E-02	2.144E-01	92.381	-0.020	9.498E-01	9.815E-01
ENSG00000253710	ALG11	185.318	-0.530	4.487E-02	2.144E-01	162.717	-0.394	1.837E-01	4.825E-01
ENSG00000164050	PLXNB1	164.533	-0.569	4.512E-02	2.152E-01	172.958	-0.213	4.937E-01	7.569E-01
ENSG00000177595	PIDD1	91.837	-0.685	4.524E-02	2.156E-01	87.433	0.073	8.284E-01	9.321E-01
ENSG00000214022	REPIN1	279.786	-0.532	4.542E-02	2.161E-01	288.768	-0.167	4.854E-01	7.530E-01

ENSG00000134222	PSRC1	194.212	-0.548	4.550E-02	2.161E-01	146.797	-0.335	1.998E-01	5.017E-01
ENSG00000254901	BORCS8	164.067	-0.616	4.567E-02	2.162E-01	119.112	-0.171	5.603E-01	7.992E-01
ENSG00000272502	AC104958.2	136.976	-0.566	4.580E-02	2.166E-01	113.477	-0.256	3.714E-01	6.746E-01
ENSG00000179403	VWA1	364.424	-0.510	4.586E-02	2.166E-01	310.102	-0.425	1.185E-01	3.861E-01
ENSG00000196976	LAGE3	111.774	-0.611	4.595E-02	2.168E-01	119.176	0.023	9.397E-01	9.777E-01
ENSG00000100814	CCNB1IP1	295.796	0.487	4.599E-02	2.169E-01	292.276	0.588	2.279E-02	1.590E-01
ENSG00000130810	PPAN	103.545	-0.599	4.609E-02	2.172E-01	74.894	-0.405	2.298E-01	5.353E-01
ENSG00000107815	TWNK	185.125	-0.530	4.610E-02	2.172E-01	163.879	-0.250	3.759E-01	6.772E-01
ENSG00000090520	DNAJB11	449.949	0.437	4.625E-02	2.174E-01	514.260	0.613	3.032E-03	4.586E-02
ENSG00000273344	PAXIP1-AS1	83.970	-0.694	4.670E-02	2.188E-01	93.077	-0.169	5.950E-01	8.190E-01
ENSG00000186767	SPIN4	155.815	-0.562	4.683E-02	2.193E-01	133.199	-0.388	2.082E-01	5.110E-01
ENSG00000187024	PTRH1	50.149	-0.746	4.697E-02	2.199E-01	43.754	-0.107	7.724E-01	9.067E-01
ENSG00000054277	OPN3	93.520	-0.629	4.705E-02	2.200E-01	140.960	-0.395	1.504E-01	4.377E-01
ENSG00000130021	PUDP	183.617	-0.518	4.721E-02	2.204E-01	188.317	-0.264	3.033E-01	6.145E-01
ENSG00000129317	PUS7L	166.583	-0.556	4.743E-02	2.211E-01	156.348	-0.106	6.986E-01	8.720E-01
ENSG00000162735	PEX19	182.648	-0.522	4.809E-02	2.233E-01	183.345	-0.043	8.774E-01	9.526E-01
ENSG00000188493	C19orf54	75.667	-0.742	4.815E-02	2.233E-01	63.681	-0.363	3.072E-01	6.170E-01
ENSG00000256269	HMBS	210.601	-0.514	4.881E-02	2.252E-01	198.616	-0.351	1.773E-01	4.735E-01
ENSG00000104177	MYEF2	49.036	-0.784	4.915E-02	2.262E-01	76.281	-0.198	5.479E-01	7.916E-01
ENSG00000143771	CNIH4	369.562	0.427	4.994E-02	2.287E-01	357.565	0.549	1.337E-02	1.160E-01
ENSG00000139618	BRCA2	148.508	-0.610	5.015E-02	2.293E-01	216.446	-0.367	1.675E-01	4.603E-01
ENSG00000185090	MANEAL	169.958	-0.571	5.018E-02	2.293E-01	183.419	-0.281	2.960E-01	6.060E-01
ENSG00000167601	AXL	60.729	-0.755	5.030E-02	2.295E-01	49.708	0.336	3.824E-01	6.824E-01
ENSG00000164252	AGGF1	165.583	-0.575	5.044E-02	2.299E-01	222.074	-0.399	1.181E-01	3.856E-01
ENSG00000089050	RBBP9	150.405	-0.599	5.074E-02	2.309E-01	193.663	-0.270	3.410E-01	6.489E-01
ENSG00000214013	GANC	83.295	-0.666	5.087E-02	2.313E-01	64.472	-0.041	9.105E-01	9.677E-01
ENSG00000016864	GLT8D1	194.738	-0.605	5.130E-02	2.329E-01	224.494	-0.500	4.701E-02	2.361E-01

ENSG00000175556	LONRF3	29.306	0.844	5.158E-02	2.337E-01	35.398	0.784	5.155E-02	2.481E-01
ENSG00000132376	INPP5K	39.323	-0.765	5.239E-02	2.358E-01	41.236	-0.251	5.091E-01	7.670E-01
ENSG00000181026	AEN	1134.766	0.385	5.305E-02	2.379E-01	1014.752	0.514	1.215E-02	1.099E-01
ENSG00000171853	TRAPPC12	111.473	-0.675	5.369E-02	2.399E-01	119.413	-0.042	9.029E-01	9.639E-01
ENSG00000078902	TOLLIP	584.845	0.443	5.401E-02	2.404E-01	590.890	0.516	3.273E-02	1.932E-01
ENSG00000184500	PROS1	90.502	-0.598	5.460E-02	2.420E-01	116.775	-0.142	6.263E-01	8.386E-01
ENSG00000177337	DLGAP1-AS1	66.183	-0.690	5.481E-02	2.421E-01	58.349	-0.316	3.773E-01	6.781E-01
ENSG00000225697	SLC26A6	75.758	-0.666	5.492E-02	2.421E-01	89.057	0.002	9.954E-01	9.983E-01
ENSG00000177082	WDR73	90.539	-0.634	5.491E-02	2.421E-01	124.387	-0.276	3.292E-01	6.370E-01
ENSG00000169188	APEX2	135.468	-0.554	5.481E-02	2.421E-01	144.893	-0.320	2.696E-01	5.793E-01
ENSG00000122787	AKR1D1	52.309	-0.724	5.505E-02	2.424E-01	58.647	-0.343	3.476E-01	6.536E-01
ENSG00000176410	DNAJC30	101.605	-0.613	5.510E-02	2.425E-01	94.235	-0.319	3.016E-01	6.120E-01
ENSG00000111885	MAN1A1	598.285	-0.534	5.517E-02	2.427E-01	455.489	-0.019	9.308E-01	9.750E-01
ENSG00000138002	IFT172	35.869	-0.775	5.537E-02	2.430E-01	34.930	-0.088	8.188E-01	9.271E-01
ENSG00000107882	SUFU	56.242	-0.742	5.553E-02	2.430E-01	73.508	-0.431	1.862E-01	4.855E-01
ENSG00000240771	ARHGEF25	70.886	-0.667	5.549E-02	2.430E-01	61.489	-0.080	8.333E-01	9.345E-01
ENSG00000075407	ZNF37A	173.010	-0.658	5.548E-02	2.430E-01	223.267	-0.295	2.352E-01	5.414E-01
ENSG00000160325	CACFD1	106.903	-0.613	5.542E-02	2.430E-01	95.922	-0.212	5.347E-01	7.856E-01
ENSG00000133935	ERG28	221.013	-0.537	5.562E-02	2.432E-01	300.195	-0.493	3.769E-02	2.090E-01
ENSG00000115286	NDUFS7	118.971	-0.615	5.576E-02	2.435E-01	94.754	0.097	7.936E-01	9.176E-01
ENSG00000240695	AC117382.1	19.527	0.840	5.584E-02	2.438E-01	32.515	0.663	1.016E-01	3.564E-01
ENSG00000185019	UBOX5	75.881	-0.634	5.603E-02	2.441E-01	75.597	-0.396	2.713E-01	5.811E-01
ENSG00000100266	PACSIN2	412.082	0.482	5.628E-02	2.448E-01	423.807	0.622	2.047E-02	1.501E-01
ENSG00000143384	MCL1	1095.964	0.338	5.728E-02	2.478E-01	1292.565	0.534	7.769E-03	8.304E-02
ENSG00000138801	PAPSS1	303.784	-0.512	5.743E-02	2.481E-01	295.883	-0.365	1.417E-01	4.260E-01
ENSG00000180385	EMC3-AS1	57.266	-0.676	5.762E-02	2.486E-01	61.338	-0.263	4.822E-01	7.519E-01
ENSG00000130787	HIP1R	26.123	0.813	5.772E-02	2.487E-01	31.484	1.112	5.967E-03	7.083E-02

ENSG00000273559	CWC25	273.999	0.499	5.788E-02	2.492E-01	264.822	0.665	1.832E-02	1.405E-01
ENSG00000166200	COPS2	408.178	0.399	5.809E-02	2.495E-01	398.152	0.787	1.208E-03	2.517E-02
ENSG00000266680	AL135905.1	50.423	-0.724	5.851E-02	2.508E-01	26.169	-0.881	3.573E-02	2.028E-01
ENSG00000171793	CTPS1	225.939	-0.502	5.894E-02	2.514E-01	217.558	0.254	3.043E-01	6.153E-01
ENSG00000148175	STOM	228.346	0.452	5.918E-02	2.521E-01	211.074	0.678	5.332E-03	6.642E-02
ENSG00000141219	C17orf80	98.761	-0.698	5.973E-02	2.534E-01	129.136	-0.104	7.075E-01	8.767E-01
ENSG00000116984	MTR	246.970	-0.500	6.029E-02	2.547E-01	254.507	-0.327	1.695E-01	4.641E-01
ENSG00000235954	TTC28-AS1	83.618	-0.734	6.054E-02	2.552E-01	93.602	-0.429	2.103E-01	5.135E-01
ENSG00000120647	CCDC77	70.200	-0.679	6.076E-02	2.556E-01	68.628	-0.352	3.498E-01	6.557E-01
ENSG00000254682	AP002387.2	79.457	-0.642	6.125E-02	2.567E-01	55.809	-0.198	5.740E-01	8.066E-01
ENSG00000158555	GDPD5	120.989	-0.531	6.124E-02	2.567E-01	117.892	-0.355	2.304E-01	5.360E-01
ENSG00000116212	LRRC42	505.128	0.414	6.129E-02	2.567E-01	484.056	0.622	7.989E-03	8.492E-02
ENSG00000158793	NIT1	67.217	-0.680	6.159E-02	2.577E-01	75.430	-0.113	7.410E-01	8.923E-01
ENSG00000267317	AC027307.2	68.495	-0.742	6.247E-02	2.599E-01	69.316	-0.320	4.043E-01	7.008E-01
ENSG00000147457	CHMP7	99.877	-0.589	6.250E-02	2.599E-01	86.526	-0.288	3.620E-01	6.670E-01
ENSG00000178802	MPI	141.965	-0.524	6.307E-02	2.615E-01	110.353	-0.448	1.541E-01	4.423E-01
ENSG00000140093	SERPINA10	54.045	-0.740	6.335E-02	2.623E-01	41.841	-0.379	3.668E-01	6.712E-01
ENSG00000158480	SPATA2	185.752	0.490	6.340E-02	2.624E-01	211.363	0.535	4.479E-02	2.297E-01
ENSG00000145220	LYAR	970.176	0.359	6.369E-02	2.630E-01	842.151	0.542	8.993E-03	9.125E-02
ENSG00000169856	ONECUT1	31.143	-0.798	6.394E-02	2.635E-01	48.983	-0.442	2.565E-01	5.668E-01
ENSG00000100092	SH3BP1	167.922	-0.546	6.408E-02	2.635E-01	129.418	-0.077	8.045E-01	9.221E-01
ENSG00000145293	ENOPH1	252.768	0.464	6.410E-02	2.635E-01	236.462	0.535	2.597E-02	1.704E-01
ENSG00000131558	EXOC4	137.334	-0.535	6.461E-02	2.647E-01	126.752	-0.337	2.369E-01	5.433E-01
ENSG00000110906	KCTD10	209.703	0.500	6.473E-02	2.650E-01	212.674	0.528	3.915E-02	2.138E-01
ENSG00000173614	NMNAT1	40.227	-0.753	6.506E-02	2.655E-01	42.416	-0.354	3.688E-01	6.726E-01
ENSG00000140939	NOL3	18.056	0.810	6.550E-02	2.666E-01	37.191	0.739	5.953E-02	2.688E-01
ENSG00000104853	CLPTM1	289.729	-0.615	6.572E-02	2.669E-01	208.917	-0.257	4.138E-01	7.072E-01

ENSG00000173349	SFT2D3	70.807	-0.672	6.586E-02	2.673E-01	68.332	-0.298	3.961E-01	6.949E-01
ENSG00000170175	CHRNB1	45.378	-0.745	6.591E-02	2.673E-01	56.069	-0.059	8.741E-01	9.523E-01
ENSG00000132688	NES	84.544	-0.673	6.629E-02	2.678E-01	117.226	-0.223	5.045E-01	7.642E-01
ENSG00000169131	ZNF354A	27.255	0.779	6.624E-02	2.678E-01	34.743	0.817	3.715E-02	2.072E-01
ENSG00000173706	HEG1	151.621	-0.575	6.640E-02	2.680E-01	181.152	-0.265	3.042E-01	6.153E-01
ENSG00000137996	RTCA	226.118	0.480	6.655E-02	2.684E-01	220.776	0.719	6.636E-03	7.522E-02
ENSG00000204599	TRIM39	44.064	-0.749	6.671E-02	2.687E-01	78.685	-0.300	4.093E-01	7.033E-01
ENSG00000139921	TMX1	145.315	-0.541	6.707E-02	2.697E-01	159.611	-0.166	5.605E-01	7.993E-01
ENSG00000163320	CGGBP1	207.463	0.498	6.726E-02	2.700E-01	174.945	0.529	6.736E-02	2.850E-01
ENSG00000138180	CEP55	203.856	-0.542	6.730E-02	2.700E-01	283.212	-0.144	5.780E-01	8.091E-01
ENSG00000105137	SYDE1	57.489	-0.658	6.744E-02	2.702E-01	49.753	-0.469	2.506E-01	5.594E-01
ENSG00000085185	BCORL1	73.569	-0.683	6.827E-02	2.727E-01	87.024	-0.370	2.287E-01	5.340E-01
ENSG00000168268	NT5DC2	385.554	-0.509	6.850E-02	2.732E-01	326.057	-0.443	1.249E-01	3.979E-01
ENSG00000008086	CDKL5	59.309	-0.700	6.960E-02	2.757E-01	73.837	-0.316	3.881E-01	6.866E-01
ENSG00000133943	DGLUCY	49.907	-0.672	6.946E-02	2.757E-01	59.196	-0.429	2.202E-01	5.253E-01
ENSG00000110074	FOXRED1	102.192	-0.604	6.959E-02	2.757E-01	88.715	-0.423	2.039E-01	5.056E-01
ENSG00000198315	ZKSCAN8	88.537	-0.594	6.958E-02	2.757E-01	82.312	-0.293	3.691E-01	6.728E-01
ENSG00000139718	SETD1B	98.567	-0.557	6.951E-02	2.757E-01	87.119	-0.242	4.595E-01	7.377E-01
ENSG00000244300	GATA2-AS1	36.124	-0.753	6.993E-02	2.765E-01	32.592	0.040	9.180E-01	9.693E-01
ENSG00000068079	IFI35	56.694	-0.669	7.052E-02	2.780E-01	37.335	-0.460	2.562E-01	5.664E-01
ENSG00000174791	RIN1	51.719	-0.669	7.061E-02	2.783E-01	49.678	-0.130	7.327E-01	8.888E-01
ENSG00000146463	ZMYM4	178.699	-0.527	7.084E-02	2.786E-01	243.729	-0.399	1.309E-01	4.084E-01
ENSG00000148660	CAMK2G	90.323	-0.646	7.159E-02	2.804E-01	78.447	0.405	2.641E-01	5.739E-01
ENSG00000214530	STARD10	103.432	-0.554	7.239E-02	2.823E-01	97.511	-0.436	1.744E-01	4.696E-01
ENSG00000188811	NHLRC3	62.025	-0.665	7.250E-02	2.825E-01	54.260	-0.145	6.958E-01	8.702E-01
ENSG00000236924	AL162411.1	36.333	-0.768	7.403E-02	2.863E-01	32.964	-0.453	2.688E-01	5.782E-01
ENSG00000108961	RANGRF	387.099	0.377	7.413E-02	2.866E-01	366.924	0.640	2.874E-03	4.405E-02

ENSG00000261324	AC010168.2	43.594	-0.728	7.461E-02	2.876E-01	40.464	-0.377	3.454E-01	6.514E-01
ENSG00000104679	R3HCC1	75.893	-0.628	7.469E-02	2.878E-01	100.127	0.135	6.458E-01	8.469E-01
ENSG00000115255	REEP6	61.515	-0.691	7.473E-02	2.878E-01	63.710	-0.472	2.328E-01	5.380E-01
ENSG00000116514	RNF19B	29.210	0.782	7.497E-02	2.884E-01	37.906	0.726	7.173E-02	2.933E-01
ENSG00000158636	EMSY	100.586	-0.599	7.526E-02	2.886E-01	74.881	-0.067	8.374E-01	9.360E-01
ENSG00000163322	ABRAXAS1	56.093	-0.686	7.564E-02	2.895E-01	59.397	-0.283	4.190E-01	7.095E-01
ENSG00000163701	IL17RE	88.291	-0.684	7.571E-02	2.895E-01	69.692	-0.136	7.242E-01	8.860E-01
ENSG00000130844	ZNF331	104.807	-0.549	7.634E-02	2.910E-01	126.004	-0.195	4.886E-01	7.536E-01
ENSG00000048162	NOP16	421.784	0.412	7.700E-02	2.924E-01	315.660	0.580	2.158E-02	1.542E-01
ENSG00000136950	ARPC5L	567.515	0.379	7.745E-02	2.937E-01	592.995	0.568	7.162E-03	7.876E-02
ENSG00000228716	DHFR	139.241	-0.534	7.752E-02	2.938E-01	125.911	-0.222	4.736E-01	7.471E-01
ENSG00000197355	UAP1L1	57.431	-0.641	7.847E-02	2.959E-01	41.707	0.007	9.847E-01	9.936E-01
ENSG00000167965	MLST8	59.452	-0.623	7.845E-02	2.959E-01	47.169	0.146	7.160E-01	8.820E-01
ENSG00000100299	ARSA	45.678	-0.724	7.864E-02	2.963E-01	28.951	-0.299	4.700E-01	7.453E-01
ENSG00000180096	SEPTIN1	44.551	-0.696	7.898E-02	2.971E-01	29.026	-0.601	1.485E-01	4.359E-01
ENSG00000100154	TTC28	54.617	-0.666	7.900E-02	2.971E-01	72.350	0.115	7.477E-01	8.951E-01
ENSG00000136931	NR5A1	193.399	-0.625	7.927E-02	2.976E-01	190.946	-0.154	6.264E-01	8.386E-01
ENSG00000136935	GOLGA1	102.721	-0.560	7.918E-02	2.976E-01	183.792	-0.488	5.994E-02	2.696E-01
ENSG00000106077	ABHD11	39.473	-0.695	7.980E-02	2.988E-01	52.879	-0.371	3.164E-01	6.266E-01
ENSG00000099246	RAB18	528.786	0.345	8.005E-02	2.995E-01	496.079	0.513	1.066E-02	1.011E-01
ENSG00000197763	TXNRD3	51.529	-0.650	8.026E-02	3.000E-01	37.349	-0.294	4.156E-01	7.077E-01
ENSG00000141026	MED9	106.900	-0.516	8.036E-02	3.001E-01	99.837	-0.207	5.234E-01	7.781E-01
ENSG00000268104	SLC6A14	45.911	-0.703	8.058E-02	3.007E-01	17.928	0.075	8.529E-01	NA
ENSG00000128039	SRD5A3	98.066	-0.555	8.085E-02	3.012E-01	109.709	-0.124	6.880E-01	8.669E-01
ENSG00000151806	GUF1	103.970	-0.631	8.262E-02	3.055E-01	204.432	-0.019	9.405E-01	9.779E-01
ENSG00000108641	B9D1	39.648	-0.746	8.302E-02	3.065E-01	42.682	-0.262	4.890E-01	7.541E-01
ENSG00000087088	BAX	130.556	-0.555	8.327E-02	3.071E-01	111.946	-0.234	5.389E-01	7.874E-01

ENSG00000197766	CFD	45.827	-0.719	8.353E-02	3.075E-01	82.836	-0.453	1.951E-01	4.961E-01
ENSG00000130544	ZNF557	61.963	-0.627	8.401E-02	3.079E-01	56.179	-0.360	3.219E-01	6.298E-01
ENSG00000155959	VBP1	166.634	0.469	8.401E-02	3.079E-01	183.655	0.513	4.075E-02	2.185E-01
ENSG00000124209	RAB22A	260.406	0.407	8.404E-02	3.080E-01	245.254	0.581	1.904E-02	1.441E-01
ENSG00000158863	FAM160B2	92.797	-0.540	8.410E-02	3.081E-01	98.844	-0.192	5.530E-01	7.942E-01
ENSG00000279088	AC022400.7	134.786	0.485	8.462E-02	3.096E-01	126.397	0.544	6.142E-02	2.727E-01
ENSG00000120159	CAAP1	131.142	-0.521	8.508E-02	3.106E-01	139.752	-0.341	2.275E-01	5.327E-01
ENSG00000148572	NRBF2	203.880	0.481	8.538E-02	3.111E-01	172.607	0.589	3.682E-02	2.065E-01
ENSG00000119401	TRIM32	51.946	-0.676	8.566E-02	3.116E-01	60.366	-0.103	7.885E-01	9.151E-01
ENSG00000150457	LATS2	91.550	-0.629	8.595E-02	3.123E-01	133.970	-0.210	4.853E-01	7.530E-01
ENSG00000114790	ARHGEF26	49.416	-0.661	8.616E-02	3.128E-01	56.716	-0.166	6.465E-01	8.472E-01
ENSG00000154920	EME1	67.800	-0.705	8.688E-02	3.140E-01	72.219	-0.471	1.890E-01	4.880E-01
ENSG00000146477	SLC22A3	73.919	-0.573	8.737E-02	3.148E-01	63.173	0.036	9.145E-01	9.684E-01
ENSG00000173209	AHSA2P	190.156	-0.501	8.736E-02	3.148E-01	268.861	-0.309	2.132E-01	5.169E-01
ENSG00000136827	TOR1A	107.688	-0.535	8.777E-02	3.161E-01	101.090	-0.411	1.907E-01	4.903E-01
ENSG00000147133	TAF1	283.741	-0.513	8.862E-02	3.183E-01	238.392	-0.340	2.809E-01	5.921E-01
ENSG00000135900	MRPL44	393.416	0.396	8.899E-02	3.192E-01	336.065	0.577	1.659E-02	1.319E-01
ENSG00000167487	KLHL26	43.022	-0.670	8.955E-02	3.201E-01	64.922	-0.333	3.362E-01	6.438E-01
ENSG00000136158	SPRY2	28.993	0.734	8.965E-02	3.203E-01	33.275	0.965	2.040E-02	1.497E-01
ENSG00000090432	MUL1	149.082	-0.531	9.011E-02	3.213E-01	162.322	-0.079	7.712E-01	9.063E-01
ENSG00000095637	SORBS1	63.994	-0.592	9.035E-02	3.219E-01	99.473	-0.132	6.903E-01	8.680E-01
ENSG00000247077	PGAM5	232.727	0.460	9.036E-02	3.219E-01	179.285	0.556	3.726E-02	2.074E-01
ENSG00000110422	HIPK3	339.388	0.425	9.131E-02	3.241E-01	300.793	0.890	2.579E-04	8.882E-03
ENSG00000149582	TMEM25	43.709	-0.702	9.201E-02	3.254E-01	41.287	0.249	5.375E-01	7.870E-01
ENSG00000140326	CDAN1	52.305	-0.655	9.212E-02	3.254E-01	48.530	-0.200	5.898E-01	8.162E-01
ENSG00000196912	ANKRD36B	49.019	-0.625	9.208E-02	3.254E-01	52.799	-0.393	2.670E-01	5.767E-01
ENSG00000103657	HERC1	158.536	-0.509	9.208E-02	3.254E-01	182.062	-0.350	1.889E-01	4.878E-01

ENSG00000100239	PPP6R2	79.423	-0.580	9.225E-02	3.255E-01	93.234	-0.300	4.042E-01	7.008E-01
ENSG00000167280	ENGASE	58.372	-0.619	9.290E-02	3.268E-01	63.806	-0.355	3.246E-01	6.319E-01
ENSG00000151689	INPP1	137.375	0.461	9.441E-02	3.301E-01	128.948	0.618	3.952E-02	2.150E-01
ENSG00000197579	TOPORS	306.857	0.446	9.495E-02	3.311E-01	323.605	0.667	9.434E-03	9.448E-02
ENSG00000164916	FOXK1	304.290	0.466	9.493E-02	3.311E-01	186.755	0.945	1.611E-03	2.983E-02
ENSG00000145743	FBXL17	101.618	-0.559	9.498E-02	3.311E-01	79.323	-0.275	4.144E-01	7.075E-01
ENSG00000106477	CEP41	88.144	-0.579	9.502E-02	3.311E-01	89.165	-0.132	6.966E-01	8.709E-01
ENSG00000145337	PYURF	272.049	0.413	9.514E-02	3.314E-01	251.050	0.530	2.616E-02	1.713E-01
ENSG00000055208	TAB2	239.926	0.432	9.583E-02	3.326E-01	245.785	0.820	1.955E-03	3.406E-02
ENSG00000158769	F11R	503.721	0.395	9.662E-02	3.335E-01	357.186	0.543	2.526E-02	1.679E-01
ENSG00000134108	ARL8B	292.145	0.399	9.698E-02	3.343E-01	407.262	0.619	5.992E-03	7.097E-02
ENSG00000165684	SNAPC4	47.109	-0.637	9.736E-02	3.348E-01	53.980	0.462	2.167E-01	5.218E-01
ENSG00000133818	RRAS2	218.595	0.407	9.784E-02	3.361E-01	245.023	0.530	2.910E-02	1.811E-01
ENSG00000114126	TFDP2	45.938	-0.634	9.799E-02	3.365E-01	50.327	-0.444	2.367E-01	5.431E-01
ENSG00000162600	OMA1	39.062	-0.672	9.839E-02	3.371E-01	37.718	0.069	8.596E-01	9.473E-01
ENSG00000159256	MORC3	157.951	0.482	9.854E-02	3.375E-01	101.573	0.532	8.031E-02	3.131E-01
ENSG00000214855	APOC1P1	75.247	-0.598	9.866E-02	3.377E-01	77.497	-0.041	8.997E-01	9.629E-01
ENSG00000155903	RASA2	54.091	-0.623	9.912E-02	3.387E-01	51.904	-0.101	8.011E-01	9.197E-01
ENSG00000099290	WASHC2A	93.445	-0.542	1.003E-01	3.414E-01	97.645	-0.418	2.091E-01	5.121E-01
ENSG00000197969	VPS13A	150.656	-0.510	1.003E-01	3.414E-01	242.195	-0.358	1.528E-01	4.405E-01
ENSG00000166348	USP54	35.362	-0.720	1.015E-01	3.434E-01	46.181	-0.082	8.291E-01	9.322E-01
ENSG00000228801	AC064807.1	43.635	-0.656	1.018E-01	3.441E-01	30.755	-0.223	5.874E-01	8.145E-01
ENSG00000257742	RP11-350F4.2	109.470	-0.584	1.019E-01	3.442E-01	96.059	-0.131	6.898E-01	8.680E-01
ENSG00000221838	AP4M1	59.272	-0.586	1.028E-01	3.459E-01	43.453	-0.174	6.594E-01	8.531E-01
ENSG00000184083	FAM120C	36.180	-0.714	1.030E-01	3.462E-01	27.051	-0.874	3.684E-02	2.065E-01
ENSG00000171320	ESCO2	103.305	-0.593	1.030E-01	3.462E-01	85.517	-0.409	2.610E-01	5.708E-01
ENSG00000114115	RBP1	154.608	-0.563	1.033E-01	3.469E-01	163.143	0.056	8.688E-01	9.507E-01

ENSG00000119318	RAD23B	1356.902	0.322	1.041E-01	3.486E-01	1263.475	0.504	1.998E-02	1.486E-01
ENSG00000232838	PET117	70.000	-0.607	1.050E-01	3.497E-01	73.323	0.113	7.383E-01	8.914E-01
ENSG00000125968	ID1	5208.596	0.379	1.050E-01	3.497E-01	4104.769	0.724	1.751E-06	1.754E-04
ENSG00000120029	C10orf76	105.847	0.495	1.053E-01	3.502E-01	92.928	0.798	1.000E-02	9.753E-02
ENSG00000229539	AL353194.1	44.298	-0.630	1.054E-01	3.502E-01	35.088	-0.384	3.422E-01	6.489E-01
ENSG00000131469	RPL27	6579.516	0.276	1.068E-01	3.522E-01	5420.429	0.555	1.961E-02	1.472E-01
ENSG00000131773	KHDRBS3	210.545	0.477	1.068E-01	3.522E-01	164.370	0.613	2.754E-02	1.745E-01
ENSG00000137070	IL11RA	34.959	-0.644	1.075E-01	3.535E-01	29.251	0.167	6.829E-01	8.640E-01
ENSG00000164961	WASHC5	57.438	-0.616	1.082E-01	3.548E-01	52.812	0.257	4.765E-01	7.493E-01
ENSG00000127993	RBM48	42.890	-0.627	1.088E-01	3.560E-01	41.274	-0.273	4.685E-01	7.440E-01
ENSG00000100058	CRYBB2P1	105.120	0.495	1.087E-01	3.560E-01	105.173	0.883	7.759E-03	8.303E-02
ENSG00000167005	NUDT21	885.982	0.407	1.090E-01	3.564E-01	623.795	0.578	2.639E-02	1.714E-01
ENSG00000182749	PAQR7	55.522	-0.612	1.095E-01	3.574E-01	82.772	0.339	3.836E-01	6.829E-01
ENSG00000123178	SPRYD7	127.276	0.465	1.099E-01	3.582E-01	137.954	0.505	1.010E-01	3.556E-01
ENSG00000169684	CHRNA5	47.686	-0.598	1.101E-01	3.583E-01	41.809	-0.174	6.166E-01	8.320E-01
ENSG00000233429	HOTAIRM1	23.686	0.693	1.107E-01	3.598E-01	39.516	0.705	6.510E-02	2.793E-01
ENSG00000129657	SEC14L1	334.223	0.374	1.110E-01	3.604E-01	307.357	0.514	4.039E-02	2.177E-01
ENSG00000120686	UFM1	316.641	0.416	1.112E-01	3.609E-01	233.043	0.701	5.908E-03	7.037E-02
ENSG00000131188	PRR7	77.155	-0.547	1.118E-01	3.622E-01	80.836	-0.177	6.435E-01	8.453E-01
ENSG00000154146	NRGN	36.460	-0.663	1.125E-01	3.633E-01	29.273	0.072	8.615E-01	9.475E-01
ENSG00000117751	PPP1R8	461.267	0.355	1.141E-01	3.664E-01	388.750	0.537	1.122E-02	1.045E-01
ENSG00000205356	TECPR1	42.917	-0.621	1.144E-01	3.666E-01	53.333	-0.469	2.114E-01	5.147E-01
ENSG00000136280	CCM2	63.713	-0.590	1.147E-01	3.670E-01	56.179	-0.460	2.285E-01	5.340E-01
ENSG00000204267	TAP2	387.277	0.359	1.155E-01	3.682E-01	308.918	0.548	6.471E-02	2.787E-01
ENSG00000101413	RPRD1B	265.674	0.444	1.161E-01	3.692E-01	225.682	0.553	2.484E-02	1.668E-01
ENSG00000163703	CRELD1	89.206	-0.515	1.165E-01	3.698E-01	78.716	-0.394	2.679E-01	5.776E-01
ENSG00000147854	UHRF2	237.372	0.399	1.169E-01	3.702E-01	191.554	0.612	2.087E-02	1.519E-01

ENSG00000162105	SHANK2	81.090	-0.579	1.174E-01	3.708E-01	85.580	-0.240	4.610E-01	7.388E-01
ENSG00000134250	NOTCH2	36.224	-0.657	1.177E-01	3.713E-01	33.573	-0.032	9.371E-01	9.770E-01
ENSG00000108984	MAP2K6	72.894	-0.628	1.177E-01	3.713E-01	72.540	-0.141	6.691E-01	8.575E-01
ENSG00000114933	INO80D	184.780	0.451	1.179E-01	3.717E-01	144.032	0.564	4.505E-02	2.302E-01
ENSG00000065970	FOXJ2	113.713	0.493	1.189E-01	3.739E-01	101.208	0.847	6.939E-03	7.746E-02
ENSG00000173588	CEP83	85.074	-0.541	1.194E-01	3.748E-01	87.493	0.038	9.116E-01	9.679E-01
ENSG00000155304	HSPA13	141.608	0.486	1.196E-01	3.750E-01	101.922	1.291	1.038E-04	4.675E-03
ENSG00000138658	ZGRF1	33.426	-0.658	1.205E-01	3.768E-01	22.381	-0.299	4.689E-01	NA
ENSG00000171202	TMEM126A	135.128	0.449	1.221E-01	3.791E-01	144.106	0.659	2.536E-02	1.685E-01
ENSG00000257017	HP	79.228	-0.551	1.235E-01	3.809E-01	94.509	-0.209	5.133E-01	7.696E-01
ENSG00000132275	RRP8	119.971	0.477	1.236E-01	3.811E-01	162.022	0.624	3.424E-02	1.991E-01
ENSG00000114735	HEMK1	51.989	-0.560	1.240E-01	3.816E-01	44.540	-0.252	5.021E-01	7.624E-01
ENSG00000106404	CLDN15	73.512	-0.534	1.241E-01	3.816E-01	70.401	-0.375	2.770E-01	5.876E-01
ENSG00000198816	ZNF358	57.627	-0.595	1.245E-01	3.821E-01	49.033	0.002	9.964E-01	9.989E-01
ENSG00000174456	C12orf76	78.146	-0.503	1.250E-01	3.832E-01	95.886	-0.097	7.654E-01	9.039E-01
ENSG00000188313	PLSCR1	67.811	-0.537	1.251E-01	3.833E-01	59.005	-0.130	7.401E-01	8.922E-01
ENSG00000107443	CCNJ	134.931	0.437	1.252E-01	3.834E-01	135.385	0.533	5.726E-02	2.624E-01
ENSG00000005059	MCUB	45.356	-0.614	1.259E-01	3.849E-01	49.107	-0.279	4.673E-01	7.433E-01
ENSG00000086696	HSD17B2	48.654	-0.572	1.260E-01	3.849E-01	61.675	-0.396	2.720E-01	5.822E-01
ENSG00000167566	NCKAP5L	54.784	-0.563	1.266E-01	3.864E-01	58.848	-0.359	3.125E-01	6.222E-01
ENSG00000146067	FAM193B	39.737	-0.601	1.270E-01	3.868E-01	32.309	-0.457	2.472E-01	5.552E-01
ENSG00000130813	C19orf66	55.712	-0.562	1.269E-01	3.868E-01	76.631	-0.261	4.259E-01	7.138E-01
ENSG00000115568	ZNF142	57.344	-0.607	1.277E-01	3.883E-01	74.653	0.248	4.521E-01	7.306E-01
ENSG00000170445	HARS	141.026	-0.527	1.277E-01	3.883E-01	180.991	0.020	9.360E-01	9.765E-01
ENSG00000188725	SMIM15	289.586	0.342	1.279E-01	3.884E-01	279.454	0.589	1.894E-02	1.440E-01
ENSG00000011332	DPF1	65.331	-0.536	1.284E-01	3.893E-01	53.795	-0.028	9.361E-01	9.765E-01
ENSG00000135845	PIGC	198.263	0.431	1.286E-01	3.893E-01	167.458	0.816	1.654E-03	3.030E-02

ENSG00000077152	UBE2T	276.068	-0.446	1.291E-01	3.899E-01	200.627	0.548	4.072E-02	2.185E-01
ENSG00000115128	SF3B6	420.363	0.364	1.290E-01	3.899E-01	406.885	0.530	3.520E-02	2.016E-01
ENSG00000091157	WDR7	37.829	-0.607	1.293E-01	3.899E-01	36.240	-0.156	7.024E-01	8.743E-01
ENSG00000198081	ZBTB14	31.775	-0.633	1.303E-01	3.921E-01	49.571	-0.254	5.190E-01	7.744E-01
ENSG00000152223	EPG5	33.150	-0.649	1.311E-01	3.935E-01	40.552	-0.244	5.271E-01	7.810E-01
ENSG00000137842	TMEM62	49.233	-0.591	1.326E-01	3.965E-01	74.685	0.256	4.351E-01	7.198E-01
ENSG00000188070	C11orf95	100.749	-0.503	1.327E-01	3.965E-01	112.060	-0.245	4.072E-01	7.025E-01
ENSG00000141376	BCAS3	53.996	-0.604	1.334E-01	3.973E-01	68.599	-0.116	7.359E-01	8.906E-01
ENSG00000145022	TCTA	56.176	-0.549	1.347E-01	3.993E-01	72.694	0.111	7.462E-01	8.942E-01
ENSG00000157450	RNF111	55.679	-0.543	1.361E-01	4.015E-01	59.323	0.430	2.479E-01	5.564E-01
ENSG00000171960	PPIH	445.511	0.312	1.363E-01	4.015E-01	394.820	0.630	4.111E-03	5.648E-02
ENSG00000160602	NEK8	55.075	-0.563	1.366E-01	4.016E-01	71.488	-0.335	3.458E-01	6.516E-01
ENSG00000157800	SLC37A3	93.426	-0.542	1.364E-01	4.016E-01	96.772	0.094	7.869E-01	9.142E-01
ENSG00000197302	ZNF720	90.767	-0.506	1.365E-01	4.016E-01	133.934	-0.183	5.462E-01	7.908E-01
ENSG00000269834	ZNF528-AS1	25.876	0.645	1.372E-01	4.027E-01	42.812	0.816	3.335E-02	1.959E-01
ENSG00000057757	PITHD1	197.455	0.365	1.376E-01	4.034E-01	170.264	0.508	5.748E-02	2.629E-01
ENSG00000036672	USP2	49.158	-0.575	1.387E-01	4.053E-01	63.163	-0.217	5.716E-01	8.059E-01
ENSG00000101049	SGK2	92.050	-0.510	1.395E-01	4.070E-01	89.124	0.055	8.550E-01	9.447E-01
ENSG00000184307	ZDHHC23	46.056	-0.623	1.396E-01	4.071E-01	43.136	-0.207	5.886E-01	8.153E-01
ENSG00000130427	EPO	45.754	-0.574	1.397E-01	4.072E-01	25.166	-0.181	6.640E-01	8.547E-01
ENSG00000105676	ARMC6	71.364	-0.529	1.398E-01	4.075E-01	78.188	0.319	3.597E-01	6.649E-01
ENSG00000123411	IKZF4	50.714	-0.547	1.400E-01	4.077E-01	58.327	-0.473	1.968E-01	4.979E-01
ENSG00000163374	YY1AP1	248.246	0.382	1.413E-01	4.099E-01	261.366	0.603	1.008E-02	9.784E-02
ENSG00000090924	PLEKHG2	31.359	-0.641	1.427E-01	4.121E-01	46.196	-0.114	7.702E-01	9.054E-01
ENSG00000166908	PIP4K2C	317.949	0.355	1.429E-01	4.121E-01	213.609	0.549	4.946E-02	2.417E-01
ENSG00000198874	TYW1	104.544	0.452	1.427E-01	4.121E-01	91.794	0.622	4.829E-02	2.389E-01
ENSG00000103260	METRN	83.525	-0.514	1.432E-01	4.125E-01	83.626	-0.164	6.756E-01	8.605E-01

ENSG00000087299	L2HGDH	33.392	-0.641	1.448E-01	4.147E-01	59.514	-0.482	2.286E-01	5.340E-01
ENSG00000168394	TAP1	180.695	0.381	1.450E-01	4.150E-01	188.090	0.633	1.899E-02	1.440E-01
ENSG00000135476	ESPL1	44.912	-0.589	1.453E-01	4.152E-01	37.686	-0.053	8.905E-01	9.586E-01
ENSG00000100575	TIMM9	303.983	0.367	1.456E-01	4.156E-01	245.341	0.710	2.401E-03	3.909E-02
ENSG00000180098	TRNAU1AP	64.881	-0.587	1.459E-01	4.161E-01	89.480	-0.361	2.547E-01	5.650E-01
ENSG00000180228	PRKRA	132.521	0.408	1.459E-01	4.161E-01	227.041	0.543	3.186E-02	1.907E-01
ENSG00000264229	RNU4ATAC	2397.821	-0.628	1.460E-01	4.162E-01	2808.727	0.064	8.778E-01	9.526E-01
ENSG00000141577	CEP131	36.910	-0.588	1.464E-01	4.171E-01	39.483	-0.307	4.359E-01	7.202E-01
ENSG00000167114	SLC27A4	45.867	-0.572	1.467E-01	4.173E-01	67.164	-0.168	6.356E-01	8.417E-01
ENSG00000111364	DDX55	292.009	0.371	1.465E-01	4.173E-01	281.289	0.518	6.557E-02	2.805E-01
ENSG00000170085	SIMC1	47.480	-0.555	1.484E-01	4.209E-01	49.297	0.034	9.235E-01	9.719E-01
ENSG00000066813	ACSM2B	38.641	-0.620	1.501E-01	4.245E-01	57.984	-0.378	3.169E-01	6.270E-01
ENSG00000135100	HNF1A	73.482	-0.515	1.511E-01	4.262E-01	83.311	-0.058	8.711E-01	9.512E-01
ENSG00000076554	TPD52	197.277	0.361	1.512E-01	4.262E-01	165.293	0.517	6.545E-02	2.802E-01
ENSG00000175606	TMEM70	156.213	0.416	1.511E-01	4.262E-01	140.110	0.540	7.553E-02	3.023E-01
ENSG00000131470	PSMC3IP	107.812	-0.547	1.517E-01	4.271E-01	88.872	0.154	6.447E-01	8.462E-01
ENSG00000139687	RB1	73.145	-0.591	1.527E-01	4.293E-01	124.465	0.243	4.738E-01	7.471E-01
ENSG00000126461	SCAF1	103.959	-0.519	1.530E-01	4.297E-01	75.586	0.084	8.270E-01	9.315E-01
ENSG00000113971	NPHP3	54.386	-0.578	1.534E-01	4.303E-01	66.751	-0.421	2.199E-01	5.250E-01
ENSG00000241935	HOGA1	32.998	-0.573	1.537E-01	4.305E-01	42.973	-0.286	4.730E-01	7.468E-01
ENSG00000162437	RAVER2	90.950	-0.541	1.537E-01	4.305E-01	82.949	-0.408	2.418E-01	5.495E-01
ENSG00000220205	VAMP2	144.419	0.477	1.537E-01	4.305E-01	112.582	0.675	4.956E-02	2.419E-01
ENSG00000114784	EIF1B	362.534	0.339	1.546E-01	4.323E-01	335.115	0.690	4.112E-03	5.648E-02
ENSG00000214189	ZNF788	34.861	-0.574	1.558E-01	4.338E-01	29.223	-0.177	6.596E-01	8.532E-01
ENSG00000276291	FRG1HP	57.456	-0.517	1.570E-01	4.358E-01	82.484	0.067	8.428E-01	9.393E-01
ENSG00000004700	RECQL	93.332	-0.502	1.578E-01	4.368E-01	72.822	-0.088	7.879E-01	9.148E-01
ENSG00000107874	CUEDC2	36.702	-0.559	1.582E-01	4.371E-01	62.427	-0.435	2.368E-01	5.433E-01

ENSG00000148120	C9orf3	110.137	0.448	1.586E-01	4.377E-01	123.023	0.767	8.328E-03	8.740E-02
ENSG00000146215	CRIP3	37.088	-0.594	1.595E-01	4.386E-01	23.039	-0.816	5.067E-02	2.451E-01
ENSG00000120685	PROSER1	151.556	0.393	1.603E-01	4.396E-01	150.811	0.578	4.827E-02	2.389E-01
ENSG00000080371	RAB21	279.668	0.368	1.607E-01	4.401E-01	193.450	0.572	5.770E-02	2.633E-01
ENSG00000137806	NDUFAF1	61.382	-0.508	1.609E-01	4.403E-01	52.407	0.164	6.542E-01	8.507E-01
ENSG00000078487	ZCWPW1	29.153	0.603	1.616E-01	4.411E-01	38.009	0.671	9.288E-02	3.381E-01
ENSG00000014123	UFL1	243.337	0.337	1.620E-01	4.417E-01	248.412	0.506	4.805E-02	2.383E-01
ENSG00000116157	GPX7	45.154	-0.529	1.623E-01	4.422E-01	52.905	-0.275	4.421E-01	7.229E-01
ENSG00000152520	PAN3	77.832	0.468	1.627E-01	4.427E-01	92.271	0.841	9.384E-03	9.407E-02
ENSG00000275457	AL117332.1	34.497	-0.576	1.645E-01	4.456E-01	29.883	0.439	2.958E-01	6.057E-01
ENSG00000103888	CEMIP	61.950	-0.541	1.645E-01	4.456E-01	60.447	-0.148	7.027E-01	8.744E-01
ENSG00000249673	NOP14-AS1	48.464	-0.585	1.650E-01	4.462E-01	38.977	-0.200	6.095E-01	8.279E-01
ENSG00000179041	RRS1	232.597	0.413	1.661E-01	4.479E-01	185.186	0.815	3.279E-03	4.846E-02
ENSG00000212283	SNORD89	36.234	-0.592	1.678E-01	4.505E-01	54.872	0.575	1.618E-01	4.519E-01
ENSG00000175073	VCPIP1	38.688	-0.585	1.680E-01	4.506E-01	46.891	-0.295	4.639E-01	7.409E-01
ENSG00000100439	ABHD4	96.962	0.452	1.680E-01	4.506E-01	97.119	0.567	6.762E-02	2.853E-01
ENSG00000162227	TAF6L	34.530	-0.587	1.690E-01	4.519E-01	19.883	-0.079	8.504E-01	NA
ENSG00000069943	PIGB	32.151	-0.585	1.692E-01	4.521E-01	38.884	0.001	9.988E-01	9.997E-01
ENSG00000141543	EIF4A3	889.746	0.283	1.692E-01	4.521E-01	910.886	0.508	7.322E-03	7.959E-02
ENSG00000146757	ZNF92	33.021	-0.579	1.700E-01	4.537E-01	31.752	-0.166	6.910E-01	8.681E-01
ENSG00000173530	TNFRSF10D	207.457	0.374	1.713E-01	4.554E-01	168.477	0.538	4.539E-02	2.311E-01
ENSG00000259642	ST20-AS1	30.777	-0.595	1.714E-01	4.554E-01	35.185	-0.199	6.145E-01	8.308E-01
ENSG00000205707	ETFRF1	30.292	-0.565	1.729E-01	4.577E-01	34.533	-0.471	2.599E-01	5.702E-01
ENSG00000253797	UTP14C	85.923	-0.501	1.730E-01	4.577E-01	99.178	-0.079	8.070E-01	9.231E-01
ENSG00000102384	CENPI	48.761	-0.575	1.733E-01	4.580E-01	51.286	-0.231	5.416E-01	7.884E-01
ENSG00000141985	SH3GL1	674.226	0.295	1.734E-01	4.580E-01	747.726	0.672	1.143E-02	1.055E-01
ENSG00000153574	RPIA	394.235	0.342	1.738E-01	4.585E-01	275.861	0.755	6.073E-03	7.173E-02

ENSG00000124786	SLC35B3	48.169	-0.561	1.781E-01	4.651E-01	46.107	-0.054	8.874E-01	9.571E-01
ENSG00000122644	ARL4A	263.708	0.359	1.782E-01	4.651E-01	280.564	0.570	2.115E-02	1.527E-01
ENSG00000175634	RPS6KB2	76.869	-0.533	1.785E-01	4.654E-01	35.845	-0.294	4.646E-01	7.413E-01
ENSG00000043143	JADE2	62.155	-0.504	1.787E-01	4.656E-01	39.969	0.093	8.107E-01	9.241E-01
ENSG00000236810	ELOA-AS1	44.818	-0.545	1.788E-01	4.657E-01	46.221	0.444	2.445E-01	5.531E-01
ENSG00000153046	CDYL	240.587	0.314	1.789E-01	4.657E-01	255.183	0.530	2.881E-02	1.802E-01
ENSG00000137942	FNBP1L	195.639	0.426	1.794E-01	4.666E-01	271.096	0.552	2.409E-02	1.648E-01
ENSG00000188290	HES4	108.900	0.444	1.802E-01	4.676E-01	105.259	0.780	2.738E-02	1.741E-01
ENSG00000122778	KIAA1549	30.430	-0.588	1.807E-01	4.685E-01	28.254	0.351	3.829E-01	6.825E-01
ENSG00000065357	DGKA	48.383	-0.522	1.810E-01	4.687E-01	55.492	-0.171	6.296E-01	8.395E-01
ENSG00000174939	ASPHD1	81.468	-0.524	1.816E-01	4.690E-01	84.537	0.159	6.592E-01	8.531E-01
ENSG00000101639	CEP192	40.588	-0.536	1.819E-01	4.693E-01	47.905	-0.218	5.856E-01	8.137E-01
ENSG00000145214	DGKQ	46.729	-0.552	1.825E-01	4.696E-01	90.770	0.160	6.381E-01	8.430E-01
ENSG00000042088	TDP1	66.108	-0.503	1.831E-01	4.704E-01	101.131	-0.187	5.487E-01	7.916E-01
ENSG00000100077	GRK3	32.743	-0.577	1.835E-01	4.705E-01	69.782	0.343	3.178E-01	6.275E-01
ENSG00000260231	JHDM1D-AS1	38.465	-0.538	1.833E-01	4.705E-01	31.878	-0.021	9.571E-01	9.839E-01
ENSG00000144959	NCEH1	50.195	-0.523	1.835E-01	4.705E-01	50.375	-0.376	2.950E-01	6.049E-01
ENSG00000171453	POLR1C	252.048	0.352	1.835E-01	4.705E-01	328.876	0.639	4.935E-03	6.328E-02
ENSG00000109107	ALDOC	31.867	-0.582	1.838E-01	4.708E-01	51.333	-0.153	6.774E-01	8.610E-01
ENSG00000138686	BBS7	93.009	0.418	1.846E-01	4.722E-01	104.055	0.667	4.936E-02	2.415E-01
ENSG00000164823	OSGIN2	76.603	0.454	1.858E-01	4.738E-01	87.908	0.669	3.838E-02	2.115E-01
ENSG00000135655	USP15	122.236	0.391	1.860E-01	4.740E-01	193.019	0.718	1.035E-02	9.898E-02
ENSG00000141956	PRDM15	32.209	-0.557	1.861E-01	4.740E-01	25.684	-0.718	8.273E-02	3.181E-01
ENSG00000172732	MUS81	96.787	0.446	1.861E-01	4.740E-01	78.728	0.797	2.350E-02	1.621E-01
ENSG00000137955	RABGGTB	125.257	0.426	1.864E-01	4.743E-01	116.099	0.533	7.003E-02	2.898E-01
ENSG00000172766	NAA16	115.864	0.412	1.870E-01	4.753E-01	138.724	0.636	2.729E-02	1.740E-01
ENSG00000104228	TRIM35	95.753	0.415	1.881E-01	4.764E-01	94.785	0.705	2.945E-02	1.822E-01

ENSG00000184697	CLDN6	85.833	0.488	1.883E-01	4.764E-01	110.878	0.528	1.378E-01	4.195E-01
ENSG00000007255	TRAPPC6A	33.317	-0.568	1.888E-01	4.770E-01	39.637	-0.392	2.492E-01	5.582E-01
ENSG00000158373	HIST1H2BD	32.307	-0.562	1.913E-01	4.795E-01	22.423	-0.108	7.970E-01	NA
ENSG00000132356	PRKAA1	101.367	0.445	1.922E-01	4.811E-01	119.345	0.982	1.737E-03	3.133E-02
ENSG00000151876	FBXO4	69.879	-0.555	1.930E-01	4.825E-01	85.739	-0.239	4.457E-01	7.255E-01
ENSG00000007062	PROM1	42.010	-0.531	1.934E-01	4.830E-01	53.253	0.258	5.064E-01	7.649E-01
ENSG00000135119	RNFT2	35.760	-0.555	1.935E-01	4.830E-01	53.812	-0.080	8.260E-01	9.308E-01
ENSG00000112210	RAB23	31.423	-0.560	1.944E-01	4.841E-01	34.582	-0.369	3.519E-01	6.576E-01
ENSG00000077721	UBE2A	228.353	0.318	1.945E-01	4.843E-01	238.164	0.528	2.733E-02	1.740E-01
ENSG00000213339	QTRT1	64.087	-0.514	1.950E-01	4.847E-01	70.320	0.550	1.494E-01	4.367E-01
ENSG00000188483	IER5L	59.051	0.469	1.970E-01	4.874E-01	52.227	1.033	7.286E-03	7.945E-02
ENSG00000121716	PILRB	30.927	-0.554	1.978E-01	4.878E-01	29.110	0.122	7.654E-01	9.039E-01
ENSG00000213398	LCAT	45.224	-0.546	1.982E-01	4.883E-01	54.079	-0.106	7.886E-01	9.151E-01
ENSG00000114648	KLHL18	124.075	0.369	1.985E-01	4.886E-01	98.167	0.722	3.107E-02	1.880E-01
ENSG00000167840	ZNF232	48.779	-0.517	1.986E-01	4.886E-01	81.233	-0.360	2.742E-01	5.842E-01
ENSG00000008282	SYPL1	303.802	0.303	1.988E-01	4.887E-01	299.057	0.579	2.089E-02	1.519E-01
ENSG00000263563	AC138761.1	32.434	-0.529	2.005E-01	4.906E-01	43.162	0.205	5.870E-01	8.142E-01
ENSG00000031691	CENPQ	131.574	-0.503	2.009E-01	4.912E-01	158.838	0.041	8.734E-01	9.523E-01
ENSG00000164327	RICTOR	62.069	0.467	2.030E-01	4.932E-01	79.529	0.546	1.110E-01	3.724E-01
ENSG00000189227	C15orf61	369.447	0.307	2.032E-01	4.933E-01	216.771	0.602	1.679E-02	1.328E-01
ENSG00000066379	ZNRD1	300.867	0.303	2.055E-01	4.959E-01	215.202	0.695	1.789E-02	1.383E-01
ENSG00000090975	PITPNM2	71.637	0.439	2.056E-01	4.960E-01	71.784	0.516	1.342E-01	4.137E-01
ENSG00000066427	ATXN3	191.808	0.327	2.087E-01	5.000E-01	159.679	0.778	5.434E-03	6.731E-02
ENSG00000161980	POLR3K	277.716	0.295	2.097E-01	5.017E-01	255.384	0.606	9.546E-03	9.541E-02
ENSG00000143190	POU2F1	292.726	0.302	2.100E-01	5.017E-01	361.149	0.828	1.224E-03	2.534E-02
ENSG00000112242	E2F3	367.248	0.272	2.110E-01	5.028E-01	341.040	0.527	2.471E-02	1.666E-01
ENSG00000133731	IMPA1	107.011	0.416	2.114E-01	5.030E-01	109.687	0.789	8.707E-03	9.006E-02

ENSG00000171097	KYAT1	43.258	-0.544	2.125E-01	5.044E-01	52.856	-0.271	4.594E-01	7.377E-01
ENSG00000151239	TWF1	59.795	0.461	2.126E-01	5.046E-01	76.978	0.760	4.009E-02	2.167E-01
ENSG00000184056	VPS33B	43.426	-0.503	2.136E-01	5.059E-01	51.851	0.064	8.583E-01	9.465E-01
ENSG00000107566	ERLIN1	474.845	0.338	2.153E-01	5.085E-01	343.846	0.518	9.349E-02	3.392E-01
ENSG00000138780	GSTCD	49.228	-0.505	2.169E-01	5.102E-01	50.541	-0.490	2.054E-01	5.075E-01
ENSG00000131876	SNRPA1	123.886	0.408	2.202E-01	5.148E-01	115.292	0.781	8.905E-03	9.112E-02
ENSG00000064726	BTBD1	48.352	0.464	2.217E-01	5.164E-01	40.188	0.684	9.433E-02	3.406E-01
ENSG00000143390	RFX5	45.114	-0.513	2.248E-01	5.196E-01	54.779	-0.119	7.397E-01	8.922E-01
ENSG00000089060	SLC8B1	30.310	-0.523	2.253E-01	5.200E-01	47.646	-0.246	5.050E-01	7.642E-01
ENSG00000144026	ZNF514	33.349	-0.512	2.259E-01	5.204E-01	59.684	-0.465	2.398E-01	5.470E-01
ENSG00000171617	ENC1	612.379	0.254	2.280E-01	5.220E-01	579.198	0.659	7.837E-04	1.918E-02
ENSG00000243989	ACY1	32.661	-0.513	2.285E-01	5.222E-01	42.381	-0.189	6.158E-01	8.314E-01
ENSG00000138802	SEC24B	93.378	0.403	2.284E-01	5.222E-01	104.220	0.602	7.288E-02	2.959E-01
ENSG00000109861	CTSC	222.550	0.407	2.285E-01	5.222E-01	154.569	0.767	2.143E-02	1.536E-01
ENSG00000108010	GLRX3	416.471	0.271	2.301E-01	5.244E-01	392.653	0.538	2.320E-02	1.606E-01
ENSG00000104312	RIPK2	93.462	0.426	2.352E-01	5.307E-01	93.438	0.981	4.832E-03	6.275E-02
ENSG00000114942	EEF1B2	3907.280	0.181	2.363E-01	5.318E-01	3394.411	0.507	2.481E-03	3.980E-02
ENSG00000178996	SNX18	44.561	0.448	2.362E-01	5.318E-01	80.334	0.590	8.464E-02	3.215E-01
ENSG00000151576	QTRT2	175.059	0.333	2.365E-01	5.318E-01	172.155	0.520	6.976E-02	2.892E-01
ENSG00000166886	NAB2	61.897	0.429	2.377E-01	5.334E-01	58.600	0.528	1.567E-01	4.470E-01
ENSG00000169902	TPST1	51.292	0.448	2.379E-01	5.337E-01	60.801	0.600	1.050E-01	3.621E-01
ENSG00000151304	SRFBP1	156.514	0.330	2.387E-01	5.344E-01	176.806	0.634	3.685E-02	2.065E-01
ENSG00000132522	GPS2	96.627	0.357	2.394E-01	5.351E-01	132.919	0.730	1.600E-02	1.295E-01
ENSG00000274605	AL355338.1	66.163	0.462	2.405E-01	5.361E-01	73.277	0.622	6.248E-02	2.750E-01
ENSG00000070423	RNF126	1982.039	0.250	2.414E-01	5.369E-01	1688.254	0.689	2.083E-03	3.568E-02
ENSG00000206562	METTL6	36.067	-0.513	2.417E-01	5.370E-01	37.191	0.252	5.059E-01	7.646E-01
ENSG00000174365	SNHG11	217.807	0.295	2.419E-01	5.370E-01	253.045	0.682	1.173E-02	1.074E-01

ENSG00000169607	CKAP2L	286.672	0.309	2.452E-01	5.403E-01	255.206	0.686	4.392E-03	5.907E-02
ENSG00000119682	AREL1	62.080	-0.429	2.463E-01	5.411E-01	102.069	0.642	3.261E-02	1.931E-01
ENSG00000091483	FH	304.739	0.314	2.475E-01	5.427E-01	240.930	0.514	2.195E-01	5.248E-01
ENSG00000169136	ATF5	186.300	0.307	2.494E-01	5.432E-01	181.079	0.611	5.196E-02	2.487E-01
ENSG00000196453	ZNF777	95.837	0.399	2.505E-01	5.440E-01	91.009	0.650	4.648E-02	2.345E-01
ENSG00000165006	UBAP1	208.698	0.316	2.520E-01	5.456E-01	177.250	0.792	2.538E-03	4.058E-02
ENSG00000102471	NDFIP2	122.203	0.342	2.547E-01	5.483E-01	107.853	0.656	4.275E-02	2.237E-01
ENSG00000131981	LGALS3	75.282	0.395	2.547E-01	5.483E-01	71.046	0.583	8.165E-02	3.159E-01
ENSG00000162616	DNAJB4	62.413	0.424	2.555E-01	5.494E-01	46.017	0.879	3.417E-02	1.990E-01
ENSG00000116701	NCF2	37.432	0.426	2.573E-01	5.511E-01	49.715	1.372	3.524E-04	1.085E-02
ENSG00000166855	CLPX	192.398	0.308	2.577E-01	5.514E-01	179.672	0.596	3.420E-02	1.990E-01
ENSG00000007372	PAX6	30.584	-0.483	2.592E-01	5.527E-01	32.854	0.652	1.096E-01	3.702E-01
ENSG00000125846	ZNF133	67.636	0.424	2.594E-01	5.530E-01	94.994	0.655	6.706E-02	2.844E-01
ENSG00000092208	GEMIN2	82.759	0.434	2.600E-01	5.534E-01	60.339	0.542	1.475E-01	4.342E-01
ENSG00000168476	REEP4	470.305	0.268	2.631E-01	5.563E-01	404.044	0.600	5.180E-02	2.484E-01
ENSG00000149564	ESAM	57.011	0.469	2.631E-01	5.563E-01	42.654	0.838	3.141E-02	1.891E-01
ENSG00000272886	DCP1A	225.132	0.297	2.650E-01	5.579E-01	161.511	0.608	3.608E-02	2.038E-01
ENSG00000128487	SPECC1	195.473	0.304	2.668E-01	5.600E-01	242.264	0.767	2.593E-03	4.103E-02
ENSG00000188612	SUMO2	159.931	0.301	2.680E-01	5.612E-01	180.898	0.510	5.157E-02	2.481E-01
ENSG00000105063	PPP6R1	39.633	0.451	2.684E-01	5.614E-01	37.357	0.511	2.120E-01	5.157E-01
ENSG00000010017	RANBP9	140.011	0.393	2.701E-01	5.628E-01	119.572	0.846	1.644E-02	1.313E-01
ENSG00000043514	TRIT1	83.983	0.384	2.711E-01	5.638E-01	81.045	0.708	4.663E-02	2.350E-01
ENSG00000167771	RCOR2	81.019	0.424	2.740E-01	5.669E-01	63.884	1.029	1.135E-02	1.050E-01
ENSG00000074935	TUBE1	46.418	0.422	2.778E-01	5.706E-01	46.888	1.019	7.580E-03	8.187E-02
ENSG00000165156	ZHX1	197.329	0.315	2.842E-01	5.776E-01	224.603	0.752	2.806E-03	4.333E-02
ENSG00000234912	SNHG20	65.635	0.401	2.882E-01	5.818E-01	67.090	0.698	3.662E-02	2.063E-01
ENSG00000104221	BRF2	53.096	0.428	2.886E-01	5.819E-01	53.504	0.827	2.778E-02	1.754E-01

ENSG00000113595	TRIM23	63.445	0.382	2.897E-01	5.830E-01	99.497	0.909	6.687E-03	7.564E-02
ENSG00000079332	SAR1A	774.626	0.203	2.936E-01	5.872E-01	677.467	0.596	4.230E-03	5.763E-02
ENSG00000198799	LRIG2	38.292	0.409	3.004E-01	5.951E-01	39.102	0.554	1.758E-01	4.715E-01
ENSG00000120526	NUDCD1	104.878	0.346	3.039E-01	5.986E-01	91.256	0.508	9.202E-02	3.369E-01
ENSG00000188177	ZC3H6	35.636	0.435	3.057E-01	6.008E-01	55.283	0.744	3.845E-02	2.116E-01
ENSG00000279457	FO538757.1	60.526	0.400	3.073E-01	6.022E-01	61.996	0.595	9.067E-02	3.340E-01
ENSG00000185262	UBALD2	235.165	0.267	3.076E-01	6.024E-01	152.937	0.586	6.769E-02	2.853E-01
ENSG00000138750	NUP54	99.749	0.326	3.082E-01	6.031E-01	102.670	0.570	1.043E-01	3.614E-01
ENSG00000205336	ADGRG1	149.827	0.276	3.119E-01	6.055E-01	172.174	0.946	1.290E-03	2.589E-02
ENSG00000196646	ZNF136	39.681	0.402	3.134E-01	6.069E-01	34.023	0.928	2.699E-02	1.731E-01
ENSG00000120913	PDLIM2	296.898	0.243	3.144E-01	6.078E-01	288.380	0.573	6.445E-02	2.785E-01
ENSG00000105732	ZNF574	80.851	0.358	3.172E-01	6.107E-01	91.821	0.796	2.163E-02	1.544E-01
ENSG00000187118	CMC1	116.214	0.326	3.202E-01	6.124E-01	120.450	0.767	1.163E-02	1.068E-01
ENSG00000176845	METRNL	35.615	0.422	3.200E-01	6.124E-01	33.336	1.120	5.125E-03	6.495E-02
ENSG00000233971	RPS20P10	33.505	0.420	3.217E-01	6.135E-01	36.377	0.632	1.134E-01	3.767E-01
ENSG00000183963	SMTN	73.460	0.350	3.219E-01	6.135E-01	90.201	0.676	8.567E-02	3.243E-01
ENSG00000175354	PTPN2	255.926	0.224	3.241E-01	6.150E-01	196.033	0.627	2.792E-02	1.761E-01
ENSG00000247844	CCAT1	32.409	0.425	3.234E-01	6.150E-01	55.714	0.637	7.509E-02	3.015E-01
ENSG00000210184	MT-TS2	61.249	-0.398	3.254E-01	6.161E-01	44.499	0.663	NA	NA
ENSG00000105321	CCDC9	57.263	0.391	3.259E-01	6.164E-01	42.003	0.566	1.492E-01	4.367E-01
ENSG00000237550	RPL9P9	98.299	0.425	3.264E-01	6.170E-01	70.977	0.760	4.747E-02	2.374E-01
ENSG00000185269	NOTUM	795.546	0.430	3.275E-01	6.170E-01	386.892	0.750	7.295E-02	2.959E-01
ENSG00000184182	UBE2F	279.488	0.256	3.312E-01	6.199E-01	296.051	0.612	1.397E-02	1.190E-01
ENSG00000082068	WDR70	128.590	0.287	3.344E-01	6.234E-01	133.969	0.597	5.191E-02	2.487E-01
ENSG00000123136	DDX39A	106.917	0.312	3.371E-01	6.264E-01	101.726	0.632	7.125E-02	2.922E-01
ENSG00000006704	GTF2IRD1	157.703	0.259	3.391E-01	6.278E-01	189.062	0.649	1.658E-02	1.319E-01
ENSG00000117519	CNN3	578.702	0.404	3.434E-01	6.317E-01	366.364	0.715	8.227E-03	8.689E-02

ENSG00000101216	GMEB2	266.535	0.246	3.445E-01	6.329E-01	263.176	0.608	1.348E-02	1.169E-01
ENSG00000198964	SGMS1	87.323	0.310	3.466E-01	6.341E-01	63.737	0.854	1.047E-02	9.974E-02
ENSG00000172493	AFF1	94.016	0.339	3.500E-01	6.370E-01	76.123	0.514	1.317E-01	4.099E-01
ENSG00000095397	WHRN	39.813	0.358	3.521E-01	6.393E-01	35.757	0.679	8.939E-02	3.311E-01
ENSG00000151532	VTI1A	144.748	0.288	3.541E-01	6.412E-01	156.962	0.530	4.628E-02	2.340E-01
ENSG00000172728	FUT10	53.995	0.348	3.548E-01	6.413E-01	50.672	1.045	6.314E-03	7.293E-02
ENSG00000135919	SERPINE2	175.150	0.299	3.550E-01	6.416E-01	338.554	0.954	1.794E-05	1.142E-03
ENSG00000095739	BAMBI	81.818	0.307	3.565E-01	6.428E-01	100.382	0.979	5.078E-03	6.466E-02
ENSG00000186432	KPNA4	862.523	0.175	3.571E-01	6.431E-01	759.072	0.547	6.226E-03	7.256E-02
ENSG00000071282	LMCD1	65.067	0.320	3.582E-01	6.443E-01	74.965	0.549	9.221E-02	3.372E-01
ENSG00000228288	PCAT6	58.607	-0.325	3.600E-01	6.460E-01	54.037	0.741	6.184E-02	2.734E-01
ENSG00000049860	HEXB	334.899	0.220	3.611E-01	6.474E-01	299.229	0.542	3.435E-02	1.994E-01
ENSG00000156374	PCGF6	50.330	0.350	3.630E-01	6.492E-01	31.981	0.839	3.831E-02	2.112E-01
ENSG00000137393	RNF144B	75.059	0.343	3.635E-01	6.494E-01	59.309	0.903	1.327E-02	1.155E-01
ENSG00000164169	PRMT9	29.001	0.391	3.636E-01	6.494E-01	33.904	0.630	1.163E-01	3.823E-01
ENSG00000186481	ANKRD20A5P	17.815	0.397	3.648E-01	6.506E-01	47.151	0.597	1.120E-01	3.741E-01
ENSG00000182117	NOP10	1051.408	0.202	3.656E-01	6.508E-01	929.129	0.650	2.836E-03	4.365E-02
ENSG00000132155	RAF1	133.007	0.270	3.658E-01	6.508E-01	117.223	0.599	4.729E-02	2.368E-01
ENSG00000130699	TAF4	125.567	0.289	3.725E-01	6.576E-01	122.916	0.672	2.098E-02	1.522E-01
ENSG00000051382	PIK3CB	73.479	0.307	3.732E-01	6.582E-01	97.989	0.525	9.483E-02	3.420E-01
ENSG00000105186	ANKRD27	118.794	0.292	3.752E-01	6.597E-01	110.785	0.816	8.277E-03	8.712E-02
ENSG00000156232	WHAMM	143.710	0.292	3.803E-01	6.637E-01	194.070	0.556	2.538E-02	1.685E-01
ENSG00000116213	WRAP73	161.556	0.292	3.819E-01	6.649E-01	123.662	0.602	9.519E-02	3.426E-01
ENSG00000157107	FCHO2	59.608	0.314	3.820E-01	6.650E-01	52.231	1.035	5.680E-03	6.885E-02
ENSG00000121210	TMEM131L	54.194	0.343	3.887E-01	6.706E-01	78.694	0.953	5.522E-03	6.780E-02
ENSG00000148925	BTBD10	163.163	0.243	3.900E-01	6.719E-01	146.437	0.571	5.528E-02	2.576E-01
ENSG00000153989	NUS1	61.229	0.305	3.922E-01	6.742E-01	60.545	0.679	5.546E-02	2.579E-01

ENSG00000076864	RAP1GAP	80.486	-0.292	3.972E-01	6.786E-01	75.876	0.685	6.284E-02	2.750E-01
ENSG00000164211	STARD4	62.629	0.304	3.981E-01	6.794E-01	80.021	0.546	1.138E-01	3.778E-01
ENSG00000116809	ZBTB17	79.353	0.295	3.983E-01	6.795E-01	81.457	0.841	1.727E-02	1.350E-01
ENSG00000144744	UBA3	66.745	-0.293	4.041E-01	6.837E-01	81.118	0.715	3.873E-02	2.124E-01
ENSG00000225648	SBDSP1	73.653	0.334	4.055E-01	6.847E-01	74.939	0.586	1.468E-01	4.329E-01
ENSG00000199377	RNU5F-1	31.188	0.347	4.076E-01	6.864E-01	63.662	1.031	9.374E-03	9.407E-02
ENSG00000165282	PIGO	48.135	-0.337	4.112E-01	6.892E-01	36.041	0.525	1.986E-01	4.997E-01
ENSG00000117650	NEK2	179.725	0.240	4.120E-01	6.897E-01	174.757	0.660	2.498E-02	1.668E-01
ENSG00000123908	AGO2	199.653	0.229	4.160E-01	6.918E-01	163.822	1.025	3.608E-04	1.098E-02
ENSG00000160685	ZBTB7B	36.103	0.318	4.221E-01	6.974E-01	35.538	0.838	4.143E-02	2.203E-01
ENSG00000101986	ABCD1	48.574	-0.301	4.238E-01	6.986E-01	40.017	0.721	6.715E-02	2.847E-01
ENSG00000164164	OTUD4	183.171	0.218	4.255E-01	6.990E-01	222.125	0.804	2.148E-03	3.620E-02
ENSG00000149923	PPP4C	203.138	0.343	4.261E-01	6.994E-01	108.348	0.830	2.647E-02	1.716E-01
ENSG00000172932	ANKRD13D	48.097	-0.320	4.277E-01	7.002E-01	69.127	0.708	4.892E-02	2.403E-01
ENSG00000253833	AC022868.1	20.297	0.343	4.296E-01	7.020E-01	30.922	0.574	1.567E-01	4.470E-01
ENSG00000122882	ECD	181.660	0.201	4.308E-01	7.028E-01	181.523	0.655	2.096E-02	1.521E-01
ENSG00000168944	CEP120	103.969	0.237	4.372E-01	7.080E-01	122.554	0.508	8.362E-02	3.196E-01
ENSG00000126215	XRCC3	91.302	-0.277	4.378E-01	7.084E-01	68.784	0.504	1.848E-01	4.839E-01
ENSG00000075188	NUP37	217.853	0.261	4.380E-01	7.085E-01	171.439	0.541	6.088E-02	2.719E-01
ENSG00000106484	MEST	144.826	0.252	4.394E-01	7.099E-01	109.565	0.504	1.799E-01	4.772E-01
ENSG00000169826	CSGALNACT2	40.131	0.294	4.393E-01	7.099E-01	56.992	0.959	1.026E-02	9.847E-02
ENSG00000204469	PRRC2A	117.777	0.244	4.424E-01	7.125E-01	99.034	0.574	7.089E-02	2.914E-01
ENSG00000198382	UVRAG	205.876	0.197	4.453E-01	7.154E-01	234.442	0.512	2.955E-02	1.823E-01
ENSG00000038274	MAT2B	204.904	0.249	4.465E-01	7.167E-01	136.733	0.561	5.478E-02	2.560E-01
ENSG00000237238	BMS1P10	24.876	0.334	4.476E-01	7.173E-01	42.681	1.096	7.579E-03	8.187E-02
ENSG00000181722	ZBTB20	92.751	0.255	4.485E-01	7.178E-01	153.617	0.525	6.373E-02	2.773E-01
ENSG00000221968	FADS3	120.052	0.243	4.494E-01	7.183E-01	101.761	0.606	8.306E-02	3.185E-01

ENSG00000113716	HMGXB3	86.267	0.239	4.570E-01	7.245E-01	92.810	0.943	6.210E-03	7.246E-02
ENSG00000168014	C2CD3	33.237	0.301	4.586E-01	7.250E-01	48.664	0.546	1.503E-01	4.377E-01
ENSG00000146410	MTFR2	68.103	0.250	4.618E-01	7.271E-01	71.757	0.512	1.281E-01	4.038E-01
ENSG00000111859	NEDD9	557.917	0.148	4.648E-01	7.290E-01	420.914	0.510	3.093E-02	1.875E-01
ENSG00000189171	S100A13	426.838	0.189	4.706E-01	7.327E-01	331.861	0.665	8.975E-03	9.125E-02
ENSG00000115163	CENPA	220.833	0.211	4.712E-01	7.332E-01	149.258	0.795	6.612E-03	7.511E-02
ENSG00000106089	STX1A	26.644	0.303	4.764E-01	7.371E-01	40.178	1.459	2.556E-04	8.846E-03
ENSG00000100599	RIN3	50.892	0.261	4.788E-01	7.379E-01	53.756	0.516	1.581E-01	4.484E-01
ENSG00000123427	EEF1AKMT3	41.752	0.274	4.788E-01	7.379E-01	34.034	0.727	7.406E-02	2.991E-01
ENSG00000110218	PANX1	84.986	0.245	4.805E-01	7.384E-01	83.036	0.794	2.776E-02	1.754E-01
ENSG00000146909	NOM1	335.036	0.155	4.812E-01	7.387E-01	317.848	0.830	1.077E-03	2.372E-02
ENSG00000186073	C15orf41	49.254	0.277	4.814E-01	7.387E-01	32.076	0.781	4.575E-02	2.321E-01
ENSG00000152382	TADA1	91.348	0.221	4.871E-01	7.422E-01	100.600	0.602	4.253E-02	2.229E-01
ENSG00000164631	ZNF12	86.118	0.235	4.883E-01	7.427E-01	134.215	0.552	8.317E-02	3.185E-01
ENSG00000118046	STK11	70.409	-0.275	4.902E-01	7.436E-01	77.650	0.617	9.044E-02	3.336E-01
ENSG00000127870	RNF6	51.587	-0.275	4.919E-01	7.446E-01	97.828	0.553	7.011E-02	2.898E-01
ENSG00000213995	NAXD	103.228	0.239	4.967E-01	7.465E-01	104.678	0.616	5.265E-02	2.509E-01
ENSG00000119912	IDE	136.571	0.201	4.980E-01	7.476E-01	186.045	0.549	4.004E-02	2.167E-01
ENSG00000137767	SQOR	137.395	-0.199	5.008E-01	7.492E-01	105.090	0.601	6.475E-02	2.787E-01
ENSG00000149313	AASDHPPT	230.589	0.161	5.013E-01	7.495E-01	243.311	0.539	2.906E-02	1.811E-01
ENSG00000144182	LIPT1	21.684	0.292	5.038E-01	7.513E-01	35.098	0.569	1.412E-01	4.250E-01
ENSG00000102393	GLA	60.391	-0.252	5.041E-01	7.514E-01	60.533	0.755	3.760E-02	2.087E-01
ENSG00000153044	CENPH	143.136	0.186	5.095E-01	7.560E-01	135.978	0.661	2.705E-02	1.731E-01
ENSG00000175711	B3GNTL1	35.075	-0.262	5.105E-01	7.563E-01	44.620	0.515	1.805E-01	4.780E-01
ENSG00000134569	LRP4	60.761	-0.251	5.121E-01	7.572E-01	64.302	0.500	1.706E-01	4.655E-01
ENSG00000108518	PFN1	2710.860	0.254	5.159E-01	7.600E-01	2257.574	0.545	2.266E-03	3.770E-02
ENSG00000131507	NDFIP1	250.226	0.162	5.171E-01	7.611E-01	277.959	0.542	3.677E-02	2.063E-01

ENSG00000142544	CTU1	37.187	0.257	5.415E-01	7.795E-01	32.880	0.520	2.068E-01	5.094E-01
ENSG00000189190	ZNF600	58.388	0.234	5.435E-01	7.806E-01	53.022	0.554	1.445E-01	4.299E-01
ENSG00000247498	GPRC5D-AS1	41.901	-0.239	5.439E-01	7.808E-01	49.868	0.619	1.056E-01	3.631E-01
ENSG00000075131	TIPIN	46.363	0.263	5.455E-01	7.816E-01	58.533	0.519	1.542E-01	4.423E-01
ENSG00000169299	PGM2	83.588	0.200	5.465E-01	7.820E-01	86.961	0.672	4.933E-02	2.415E-01
ENSG00000027001	MIPEP	104.376	0.195	5.546E-01	7.884E-01	75.002	0.877	1.193E-02	1.087E-01
ENSG00000168488	ATXN2L	354.734	0.257	5.571E-01	7.894E-01	272.147	0.670	3.056E-02	1.865E-01
ENSG00000231312	AC007388.1	109.719	0.185	5.576E-01	7.895E-01	100.720	0.698	3.236E-02	1.922E-01
ENSG00000158079	PTPDC1	37.897	0.235	5.605E-01	7.909E-01	79.846	0.597	8.596E-02	3.243E-01
ENSG00000147789	ZNF7	38.124	0.236	5.591E-01	7.909E-01	58.918	0.693	5.568E-02	2.584E-01
ENSG00000167967	E4F1	55.668	0.215	5.655E-01	7.940E-01	64.393	0.639	9.218E-02	3.372E-01
ENSG00000136940	PDCL	132.185	0.183	5.671E-01	7.948E-01	124.611	0.832	6.908E-03	7.739E-02
ENSG00000180953	ST20	24.370	-0.245	5.686E-01	7.958E-01	32.939	0.607	1.228E-01	3.936E-01
ENSG00000146574	CCZ1B	74.126	0.193	5.690E-01	7.959E-01	81.948	0.625	8.437E-02	3.212E-01
ENSG00000180758	GPR157	144.821	-0.156	5.700E-01	7.968E-01	134.866	0.619	3.191E-02	1.908E-01
ENSG00000172345	STARD5	32.413	-0.230	5.738E-01	7.976E-01	33.991	0.869	3.196E-02	1.909E-01
ENSG00000141198	TOM1L1	91.250	0.182	5.796E-01	8.018E-01	128.235	0.549	6.881E-02	2.873E-01
ENSG00000106537	TSPAN13	38.712	0.227	5.793E-01	8.018E-01	30.436	0.571	1.628E-01	4.536E-01
ENSG00000101452	DHX35	66.015	-0.203	5.858E-01	8.063E-01	85.923	0.550	9.385E-02	3.399E-01
ENSG00000163866	SMIM12	175.249	0.145	5.901E-01	8.090E-01	179.419	0.596	2.172E-02	1.544E-01
ENSG00000151247	EIF4E	258.374	-0.128	5.957E-01	8.118E-01	334.894	0.750	5.007E-03	6.384E-02
ENSG00000223802	CERS1	38.618	0.202	6.165E-01	8.229E-01	32.207	0.674	8.768E-02	3.282E-01
ENSG00000178202	KDELC2	78.841	0.174	6.225E-01	8.271E-01	73.308	0.587	8.420E-02	3.209E-01
ENSG00000104859	CLASRP	200.957	0.150	6.242E-01	8.284E-01	172.508	0.757	3.331E-02	1.958E-01
ENSG00000273611	ZNHIT3	258.410	0.140	6.266E-01	8.300E-01	225.368	0.810	2.356E-03	3.851E-02
ENSG00000137312	FLOT1	720.533	-0.097	6.274E-01	8.303E-01	718.847	0.691	1.264E-02	1.129E-01
ENSG00000145041	DCAF1	165.877	0.173	6.296E-01	8.315E-01	116.706	0.662	5.970E-02	2.691E-01

ENSG00000237190	CDKN2AIPNL	106.287	0.150	6.302E-01	8.316E-01	114.459	0.543	1.040E-01	3.609E-01
ENSG00000151748	SAV1	105.825	-0.148	6.334E-01	8.337E-01	115.518	0.527	1.075E-01	3.664E-01
ENSG00000159658	EFCAB14	236.653	0.130	6.472E-01	8.419E-01	172.066	0.845	1.707E-03	3.100E-02
ENSG00000123094	RASSF8	20.012	0.198	6.502E-01	8.437E-01	31.865	0.796	5.777E-02	2.634E-01
ENSG00000168538	TRAPPC11	67.516	0.173	6.520E-01	8.447E-01	80.975	0.546	8.253E-02	3.178E-01
ENSG00000196636	SDHAF3	61.600	0.188	6.526E-01	8.452E-01	48.586	0.644	7.369E-02	2.983E-01
ENSG00000138050	THUMPD2	96.098	-0.140	6.587E-01	8.465E-01	74.064	0.584	1.211E-01	3.907E-01
ENSG00000165506	DNAAF2	55.253	-0.170	6.611E-01	8.479E-01	64.944	0.967	8.161E-03	8.652E-02
ENSG00000010404	IDS	42.472	-0.184	6.633E-01	8.489E-01	72.480	0.742	3.037E-02	1.856E-01
ENSG00000147316	MCPH1	104.229	0.146	6.645E-01	8.496E-01	86.081	0.627	5.750E-02	2.629E-01
ENSG00000118197	DDX59	56.460	0.159	6.645E-01	8.496E-01	73.099	0.677	5.405E-02	2.540E-01
ENSG00000134996	OSTF1	78.871	0.163	6.703E-01	8.528E-01	54.782	0.528	1.759E-01	4.715E-01
ENSG00000105290	APLP1	33.463	0.177	6.710E-01	8.528E-01	37.346	0.634	1.093E-01	3.700E-01
ENSG00000164758	MED30	48.700	0.166	6.715E-01	8.530E-01	36.301	0.987	1.492E-02	1.238E-01
ENSG00000178971	CTC1	46.878	0.174	6.716E-01	8.530E-01	65.264	0.509	1.638E-01	4.550E-01
ENSG00000177888	ZBTB41	35.505	0.171	6.717E-01	8.531E-01	64.127	0.632	6.385E-02	2.776E-01
ENSG00000157538	VPS26C	96.952	0.139	6.757E-01	8.550E-01	81.494	0.545	9.859E-02	3.497E-01
ENSG00000124140	SLC12A5	56.882	0.149	6.761E-01	8.550E-01	62.574	0.650	1.004E-01	3.544E-01
ENSG00000153551	CMTM7	373.709	0.089	6.810E-01	8.587E-01	401.307	0.591	2.010E-02	1.487E-01
ENSG00000215421	ZNF407	48.914	0.169	6.850E-01	8.607E-01	55.193	0.546	1.205E-01	3.905E-01
ENSG00000198901	PRC1	786.373	0.080	6.959E-01	8.682E-01	699.665	0.571	1.304E-02	1.146E-01
ENSG00000180488	MIGA1	45.490	-0.160	6.969E-01	8.688E-01	71.476	0.693	5.903E-02	2.669E-01
ENSG00000154889	MPPE1	43.474	-0.156	6.978E-01	8.692E-01	39.450	0.519	1.974E-01	4.986E-01
ENSG00000143919	CAMKMT	79.439	0.125	7.020E-01	8.714E-01	61.349	0.626	1.034E-01	3.602E-01
ENSG00000157214	STEAP2	74.377	-0.130	7.052E-01	8.723E-01	73.645	0.535	1.057E-01	3.631E-01
ENSG00000084636	COL16A1	44.325	0.159	7.076E-01	8.742E-01	51.394	0.639	1.023E-01	3.585E-01
ENSG00000142528	ZNF473	82.809	0.122	7.090E-01	8.753E-01	73.977	0.602	7.397E-02	2.990E-01

ENSG00000105926	MPP6	122.500	0.111	7.100E-01	8.759E-01	137.652	0.569	5.580E-02	2.585E-01
ENSG00000141642	ELAC1	26.903	-0.154	7.179E-01	8.809E-01	38.352	0.683	8.600E-02	3.243E-01
ENSG00000175376	EIF1AD	121.500	0.125	7.240E-01	8.846E-01	86.607	0.623	7.243E-02	2.948E-01
ENSG00000188002	AC026412.1	107.294	-0.107	7.273E-01	8.862E-01	140.982	0.575	6.269E-02	2.750E-01
ENSG00000167470	MIDN	877.243	0.067	7.276E-01	8.862E-01	764.157	0.643	1.134E-02	1.050E-01
ENSG00000147119	CHST7	25.095	0.151	7.302E-01	8.876E-01	30.364	0.775	5.193E-02	2.487E-01
ENSG00000123728	RAP2C	93.172	0.118	7.371E-01	8.909E-01	72.390	0.510	1.355E-01	4.159E-01
ENSG00000137575	SDCBP	423.814	0.141	7.387E-01	8.919E-01	338.513	0.693	2.667E-02	1.721E-01
ENSG00000105329	TGFB1	29.749	0.137	7.448E-01	8.947E-01	42.760	0.525	1.673E-01	4.598E-01
ENSG00000132581	SDF2	137.345	-0.102	7.462E-01	8.953E-01	146.178	0.567	6.098E-02	2.721E-01
ENSG00000163933	RFT1	222.203	0.095	7.513E-01	8.979E-01	171.526	0.504	7.612E-02	3.039E-01
ENSG00000173540	GMPPB	71.832	0.126	7.512E-01	8.979E-01	63.179	0.525	1.384E-01	4.202E-01
ENSG00000159433	STARD9	45.044	-0.122	7.519E-01	8.980E-01	69.643	0.558	1.094E-01	3.701E-01
ENSG00000269578	AC008764.7	22.003	0.135	7.555E-01	8.997E-01	52.202	1.295	1.267E-03	2.577E-02
ENSG00000164105	SAP30	93.775	-0.100	7.568E-01	9.002E-01	98.263	0.691	2.639E-02	1.714E-01
ENSG00000183605	SFXN4	428.524	0.072	7.580E-01	9.006E-01	385.896	0.663	5.644E-03	6.865E-02
ENSG00000152332	UHMK1	175.031	0.083	7.587E-01	9.009E-01	182.360	0.587	2.275E-02	1.588E-01
ENSG00000012171	SEMA3B	510.221	0.072	7.634E-01	9.042E-01	418.599	0.566	8.806E-03	9.042E-02
ENSG00000088179	PTPN4	80.165	0.101	7.647E-01	9.046E-01	78.349	0.579	7.295E-02	2.959E-01
ENSG00000118242	MREG	33.361	0.121	7.678E-01	9.058E-01	57.391	0.740	3.359E-02	1.964E-01
ENSG00000248909	HMGB1P21	36.987	0.121	7.709E-01	9.078E-01	46.962	0.839	2.653E-02	1.717E-01
ENSG00000163874	ZC3H12A	102.218	0.092	7.722E-01	9.081E-01	98.836	1.045	7.756E-04	1.905E-02
ENSG00000154059	IMPACT	58.929	0.104	7.741E-01	9.094E-01	68.052	0.718	2.963E-02	1.825E-01
ENSG00000156858	PRR14	121.685	-0.085	7.793E-01	9.117E-01	105.334	0.795	1.781E-02	1.380E-01
ENSG00000040199	PHLPP2	28.623	0.115	7.859E-01	9.151E-01	38.828	0.605	1.158E-01	3.816E-01
ENSG00000146426	TIAM2	37.681	0.108	7.875E-01	9.160E-01	62.356	0.532	1.229E-01	3.936E-01
ENSG00000172954	LCLAT1	84.194	0.091	7.934E-01	9.182E-01	106.670	0.759	1.436E-02	1.209E-01

ENSG00000165689	SDCCAG3	450.953	0.069	7.985E-01	9.197E-01	391.143	0.545	1.013E-02	9.810E-02
ENSG00000171723	GPHN	68.993	0.090	8.008E-01	9.211E-01	85.877	0.621	5.689E-02	2.614E-01
ENSG00000002919	SNX11	89.131	0.087	8.028E-01	9.220E-01	81.962	0.716	2.153E-02	1.539E-01
ENSG00000026103	FAS	36.943	0.101	8.032E-01	9.220E-01	51.691	1.009	8.968E-03	9.125E-02
ENSG00000184669	OR7E14P	66.584	0.086	8.092E-01	9.245E-01	78.800	0.695	3.476E-02	2.003E-01
ENSG00000184162	NR2C2AP	153.987	0.069	8.108E-01	9.252E-01	119.713	0.609	3.265E-02	1.931E-01
ENSG00000011198	ABHD5	45.542	0.086	8.189E-01	9.301E-01	35.743	0.639	1.094E-01	3.701E-01
ENSG00000112578	BYSL	288.423	0.050	8.258E-01	9.331E-01	273.564	0.537	1.597E-02	1.294E-01
ENSG00000088298	EDEM2	153.368	0.069	8.286E-01	9.336E-01	118.625	0.582	6.138E-02	2.727E-01
ENSG00000213598	AL049873.1	33.043	0.080	8.296E-01	9.339E-01	44.653	0.520	1.820E-01	4.805E-01
ENSG00000231940	RPS7P3	45.094	-0.079	8.337E-01	9.349E-01	34.667	0.702	7.721E-02	3.057E-01
ENSG00000198855	FICD	46.040	0.082	8.380E-01	9.372E-01	53.439	0.594	9.975E-02	3.526E-01
ENSG00000197122	SRC	141.221	-0.061	8.438E-01	9.394E-01	115.682	0.583	7.774E-02	3.071E-01
ENSG00000134056	MRPS36	237.386	-0.053	8.475E-01	9.401E-01	215.932	0.587	5.810E-02	2.643E-01
ENSG00000110104	CCDC86	536.246	0.047	8.553E-01	9.438E-01	440.106	0.879	1.549E-04	6.120E-03
ENSG00000143537	ADAM15	249.869	-0.047	8.568E-01	9.443E-01	275.142	0.642	4.765E-02	2.375E-01
ENSG00000171492	LRRC8D	100.448	0.059	8.569E-01	9.443E-01	134.409	0.543	6.983E-02	2.894E-01
ENSG00000214367	HAUS3	47.046	-0.068	8.577E-01	9.445E-01	59.460	0.920	1.440E-02	1.209E-01
ENSG00000179588	ZFPM1	41.436	0.072	8.609E-01	9.455E-01	45.127	0.529	1.607E-01	4.510E-01
ENSG00000170634	ACYP2	29.501	0.073	8.631E-01	9.462E-01	46.545	0.518	1.876E-01	4.870E-01
ENSG00000135127	BICDL1	18.278	-0.072	8.679E-01	9.481E-01	35.827	1.069	9.278E-03	9.337E-02
ENSG00000141391	PRELID3A	29.736	0.062	8.785E-01	9.527E-01	46.128	0.521	1.715E-01	4.668E-01
ENSG00000104219	ZDHHC2	38.936	0.063	8.827E-01	9.543E-01	50.882	0.736	4.998E-02	2.432E-01
ENSG00000186577	SMIM29	165.444	0.043	8.869E-01	9.564E-01	172.762	0.614	5.124E-02	2.471E-01
ENSG00000244687	UBE2V1	43.253	-0.057	8.876E-01	9.567E-01	40.784	0.944	1.611E-02	1.300E-01
ENSG00000131759	RARA	86.413	0.052	8.899E-01	9.577E-01	85.927	0.570	1.328E-01	4.114E-01
ENSG00000145979	TBC1D7	146.765	-0.041	8.926E-01	9.582E-01	79.404	0.500	1.379E-01	4.196E-01

ENSG00000186908	ZDHHC17	68.026	-0.045	8.980E-01	9.608E-01	71.696	0.755	2.616E-02	1.713E-01
ENSG00000234147	AL035446.1	89.001	0.042	9.032E-01	9.637E-01	74.556	0.704	4.494E-02	2.300E-01
ENSG00000139793	MBNL2	191.789	-0.032	9.037E-01	9.640E-01	158.938	0.568	4.856E-02	2.394E-01
ENSG00000121350	PYROXD1	28.110	0.051	9.066E-01	9.656E-01	36.573	0.602	1.200E-01	3.900E-01
ENSG00000198918	RPL39	1816.994	0.044	9.076E-01	9.658E-01	1530.161	0.510	2.150E-01	5.197E-01
ENSG00000198176	TFDP1	1229.936	0.027	9.079E-01	9.659E-01	1096.923	0.624	1.096E-03	2.389E-02
ENSG00000072401	UBE2D1	99.887	-0.034	9.092E-01	9.665E-01	107.278	0.503	1.163E-01	3.823E-01
ENSG00000130772	MED18	77.111	0.037	9.178E-01	9.698E-01	79.055	0.543	9.835E-02	3.493E-01
ENSG00000162676	GFI1	49.193	-0.040	9.223E-01	9.719E-01	44.738	0.652	1.012E-01	3.558E-01
ENSG00000262251	AC087388.1	28.553	0.040	9.257E-01	9.737E-01	41.590	0.501	1.882E-01	4.872E-01
ENSG00000131941	RHPN2	44.840	-0.038	9.260E-01	9.739E-01	56.672	0.732	4.173E-02	2.207E-01
ENSG00000125485	DDX31	60.818	0.032	9.321E-01	9.761E-01	78.170	0.759	2.712E-02	1.732E-01
ENSG00000115421	PAPOLG	58.904	-0.032	9.338E-01	9.765E-01	42.575	0.592	1.155E-01	3.811E-01
ENSG00000166860	ZBTB39	69.744	-0.029	9.337E-01	9.765E-01	85.158	0.630	4.156E-02	2.205E-01
ENSG00000101391	CDK5RAP1	263.668	-0.016	9.460E-01	9.815E-01	272.129	0.580	1.326E-02	1.155E-01
ENSG00000175104	TRAF6	39.270	0.025	9.486E-01	9.826E-01	47.324	0.581	1.285E-01	4.041E-01
ENSG00000108515	ENO3	149.566	0.020	9.495E-01	9.828E-01	162.349	0.516	5.635E-02	2.600E-01
ENSG00000203883	SOX18	72.247	-0.022	9.565E-01	9.855E-01	34.199	0.589	1.575E-01	4.479E-01
ENSG00000011485	PPP5C	126.352	-0.016	9.619E-01	9.877E-01	113.333	0.667	4.960E-02	2.419E-01
ENSG00000050820	BCAR1	272.248	-0.010	9.692E-01	9.893E-01	301.028	0.520	1.960E-02	1.472E-01
ENSG00000227036	LINC00511	124.047	0.013	9.677E-01	9.893E-01	165.818	0.605	1.924E-02	1.452E-01
ENSG00000132603	NIP7	236.125	-0.009	9.706E-01	9.894E-01	212.080	0.528	4.239E-02	2.227E-01
ENSG00000188215	DCUN1D3	69.364	-0.012	9.726E-01	9.900E-01	70.160	0.540	1.324E-01	4.108E-01
ENSG00000143753	DEGS1	46.536	0.012	9.748E-01	9.906E-01	59.772	0.739	3.493E-02	2.006E-01
ENSG00000173762	CD7	137.734	-0.005	9.898E-01	9.968E-01	144.538	0.641	7.780E-02	3.071E-01
ENSG00000167543	TP53I13	164.136	-0.004	9.908E-01	9.971E-01	151.675	0.610	4.383E-02	2.276E-01
ENSG00000042429	MED17	95.194	0.003	9.935E-01	9.977E-01	101.821	0.588	4.969E-02	2.421E-01

ENSG00000196437	ZNF569	33.920	-0.002	9.962E-01	9.985E-01	35.427	0.558	1.645E-01	4.567E-01
ENSG00000110429	FBXO3	117.749	0.001	9.983E-01	9.995E-01	93.532	0.521	8.969E-02	3.317E-01
ENSG00000066583	ISOC1	272.384	0.000	9.995E-01	1.000E+00	172.937	1.031	2.931E-03	4.474E-02
ENSG00000178445	GLDC	103.256	-0.711	NA	NA	76.467	-0.054	8.817E-01	9.547E-01
ENSG00000280138	AC027290.2	12.588	0.482	2.545E-01	NA	36.351	0.640	1.182E-01	3.856E-01
ENSG00000119689	DLST	7.838	0.543	1.881E-01	NA	30.795	1.017	1.255E-02	1.123E-01

(Direct) predicted targets

Table S11. STRING clusters analysis of targets down-regulated by miR-222-5p.

Cluster number	Protein name
1	ACSM2B
1	ADNP2
1	ADRBK2
1	AHSA2
1	ANKRD13D
1	ANKRD27
1	ARHGEF25
1	ARMC6
1	ASTN2
1	ATXN2L
1	ATXN7L3B
1	BCORL1
1	C10orf2
1	C10orf76
1	C11orf30
1	C15orf61
1	C19orf25
1	C19orf54
1	C19orf66
1	C4orf3
1	C6orf120
1	CAAP1
1	CACFD1
1	CARD9
1	CCDC57
1	CCM2
1	CCZ1B
1	CDKL5
1	CENPBD1
1	CEP131
1	CGGBP1
1	CNBP
1	CNOT2
1	CNPY4
1	CUEDC2
1	DCP1A
1	DDX1
1	DDX23
1	DNAJB4
1	DNAJC4

1	DP2
1	DPF1
1	E4F1
1	ECD
1	EIF1B
1	EPG5
1	FAM109A
1	FAM160B2
1	FAM175A
1	FAM193B
1	FMR1
1	FOXJ2
1	FOXRED2
1	GEMIN2
1	GIGYF1
1	GLRX3
1	GLT8D1
1	GRK6
1	GTF2E1
1	HAUS3
1	HAUS5
1	HEG1
1	HES4
1	HEXIM2
1	HIPK3
1	HMBS
1	HMGN3
1	HOMEZ
1	HSPA12A
1	IFI35
1	IKZF4
1	IL17RE
1	INTS6
1	IPO9
1	ITPRIPL2
1	LAGE3
1	LMCD1
1	LRIG2
1	LRRC8D
1	MAMSTR
1	MBNL2
1	MCPH1
1	MED17

1	MED18
1	MED29
1	MED30
1	MED9
1	MEF2BNB
1	MFAP1
1	MGME1
1	MIDN
1	MORF4L2
1	MPV17L
1	MRFAP1L1
1	MYEF2
1	NAA16
1	NAA50
1	NHLRC3
1	NOL3
1	NOTCH2
1	NR2C2AP
1	NR2F1
1	NRBF2
1	NUDT16
1	NUDT21
1	PAN3
1	PAPD5
1	PAPOLG
1	PCGF6
1	PFDN6
1	PKIG
1	PLEKHG2
1	POU2F1
1	PPIH
1	PROSER1
1	PRR14
1	PSMC3IP
1	PTPDC1
1	RAB22A
1	RABGAP1
1	RAD51D
1	RANGRF
1	RASSF8
1	RBCK1
1	RBM14
1	RCOR2

1	REEP6
1	RFX5
1	RIPK2
1	RNF111
1	RNF144B
1	RUNDC1
1	S100A13
1	S100P
1	SCAF1
1	SEC14L1
1	sep-01
1	SIMC1
1	SKI
1	SLC35E2B
1	SLC5A9
1	SPATA2
1	SPRYD7
1	SRRM1
1	SRSF5
1	ST20
1	TADA1
1	TAF1
1	TAF4
1	TAF6L
1	TBC1D22A
1	TBX2
1	TMEM115
1	TMEM141
1	TMEM186
1	TMEM223
1	TMEM70
1	TNFRSF10D
1	TOLLIP
1	TPRKB
1	TPST1
1	TRNAU1AP
1	TTC28
1	TTC31
1	TWF1
1	TYW1
1	UCK1
1	USP2
1	USP54

1	VCPIP1
1	VWA1
1	WAC
1	WDR73
1	WHAMM
1	ZBTB20
1	ZBTB9
1	ZC3H12A
1	ZC3H6
1	ZNF12
1	ZNF136
1	ZNF407
1	ZNF600
1	ZNF672
1	ZNF720
1	ZNF768
1	ZNF784
1	ZNFX1
2	AASDHPPT
2	ACYP2
2	ADAM15
2	AGGF1
2	AGO2
2	AGT
2	AP2M1
2	AP4M1
2	APOA1
2	ARFIP2
2	ARL8B
2	ARPC5L
2	ATF5
2	AXL
2	BCAR1
2	BCAR3
2	BCAS3
2	BMPR1A
2	BPHL
2	C9orf3
2	CAMK2G
2	CCBL1
2	CCBL2
2	CD46
2	CD7

2	CEBPA
2	CEP83
2	CFD
2	CHMP7
2	CHRNB1
2	CIC
2	CLDN6
2	CLIP2
2	COL16A1
2	CPB2
2	CYFIP2
2	DAB2IP
2	DDAH1
2	DGKA
2	DKK1
2	DNAAF2
2	DSCR3
2	EIF4E
2	EPO
2	ESAM
2	ETS2
2	ETV4
2	EVPL
2	EXOC4
2	F11R
2	F5
2	FADD
2	FAS
2	FBXO3
2	FCGRT
2	FCHSD1
2	FKBP10
2	FLOT1
2	GFI1
2	GLDC
2	GLIS2
2	GOLGA1
2	GPER1
2	GPHN
2	GPX7
2	HERC1
2	HIP1R
2	HIPK2

2	HNF1A
2	HP
2	HS6ST1
2	HSPA13
2	HSPA8
2	ID1
2	IRS1
2	ITGA2
2	ITIH5
2	KIAA1549
2	LAMA5
2	LAMTOR1
2	LGALS3
2	LGALS3BP
2	MAT2B
2	MERTK
2	MFSD3
2	MLST8
2	MON1B
2	MORC3
2	MPZL2
2	MSRB1
2	MTR
2	MUL1
2	NAB2
2	NCF2
2	NCR3LG1
2	NEDD9
2	NES
2	NGEF
2	NKX3-1
2	NR5A1
2	NUDCD1
2	ONECUT1
2	OSTF1
2	PACSIN2
2	PANX1
2	PAPSS1
2	PAX6
2	PDCL
2	PDXK
2	PFN1
2	PIK3C3

2	PIK3CB
2	PIP4K2C
2	PKP4
2	PLSCR1
2	PNRC2
2	PPP2CB
2	PPP5C
2	PRDX4
2	PRKAA1
2	PROM1
2	PROS1
2	PRR7
2	PRRC2A
2	PTPN2
2	RAB23
2	RAB8A
2	RAF1
2	RANBP9
2	RARA
2	RBP1
2	REPIN1
2	RIC8A
2	RICTOR
2	RMDN1
2	RPS6KB2
2	RRAS2
2	RTKN
2	SAP30
2	SDCBP
2	SEPP1
2	SERPINE2
2	SFXN3
2	SGK2
2	SH3GL1
2	SHANK2
2	SLC29A3
2	SLC35B3
2	SLC35E3
2	SLC6A14
2	SLC7A5
2	SORBS1
2	SOX18
2	SOX4

2	SOX9
2	SPP1
2	SPRY2
2	SRC
2	STK11
2	STOM
2	STUB1
2	STX1A
2	SUFU
2	TAB2
2	TAP1
2	TAP2
2	TESC
2	TFPI
2	TGFB1
2	TLCD1
2	TMEM25
2	TMX1
2	TNFRSF11A
2	TNRC6A
2	TOM1L1
2	TOPORS
2	TOR1A
2	TRAF3
2	TRAF6
2	TRIM25
2	TRIM39
2	TXNRD3
2	UAP1L1
2	UBAC1
2	UBAP1
2	UHMK1
2	UVRAG
2	VAMP2
2	VPS33B
2	VTI1A
2	ZBTB14
2	ZBTB17
2	ZBTB22
2	ZBTB7B
2	ZDHHC17
2	ZFPM1
3	ABCG8

3	ACACA
3	ACADM
3	ACP2
3	ACSL4
3	ACY1
3	ADD1
3	AKAP11
3	ANKIB1
3	ANKRD26
3	AP3M2
3	AP5S1
3	APLP1
3	APOM
3	AREL1
3	ARPP19
3	ARRDC1
3	ATP2B4
3	BAMBI
3	C11orf49
3	C11orf71
3	C11orf95
3	C14orf1
3	C19orf10
3	C1GALT1C1
3	C5orf66
3	C6orf1
3	CHURC1
3	CINP
3	CLDN15
3	CPN1
3	CPN2
3	CRIP3
3	DGKQ
3	DNAJB11
3	EFCAB14
3	ELOVL6
3	EPHX1
3	EPN1
3	ERV3-1
3	FADS2
3	FADS3
3	FCHO2
3	FNBP1L

3	FZD3
3	FZD4
3	GDPD5
3	GPR56
3	GPX3
3	GSTA1
3	GTF2IRD1
3	HIF1AN
3	HMGCR
3	HPS6
3	IDE
3	IDS
3	IL11RA
3	IMPA1
3	INPP1
3	INPP5K
3	IQGAP2
3	JAGN1
3	JMJD4
3	LAD1
3	LCAT
3	LCLAT1
3	LGR4
3	LIFR
3	LONRF3
3	LPAR6
3	LRP4
3	LRRC23
3	MARVELD3
3	MESDC2
3	MINPP1
3	MMP15
3	MOGAT3
3	NCEH1
3	NCKAP5L
3	NEK8
3	NIT1
3	NOTUM
3	NR1H3
3	NRGN
3	OSGIN2
3	PALM
3	PAQR7

3	PET117
3	PGP
3	PHLPP1
3	PHLPP2
3	PIPOX
3	PITPNM2
3	PLA2G12B
3	PLA2G15
3	PLLP
3	PLXNB1
3	PPAPDC2
3	PRMT9
3	PTPN4
3	RAB18
3	RAB3GAP2
3	RABGGTB
3	RAP1GAP
3	RAP2C
3	RASA2
3	RBM48
3	RNF43
3	RNF6
3	RNFT2
3	SDF2
3	SEMA3B
3	SEMA6B
3	SERPINA10
3	SERPINC1
3	SFT2D3
3	SGSH
3	SH3BP1
3	SLC12A5
3	SLC27A4
3	SLC37A3
3	SLC37A4
3	SLC5A3
3	SLMO1
3	SNX18
3	SOWAHC
3	SPIN4
3	STARD10
3	STARD4
3	STARD5

3	SULT1A2
3	SYDE1
3	SYNGR2
3	SYPL1
3	TBC1D7
3	TEX2
3	TGFBR3
3	THEM6
3	TIAM2
3	TMEM161A
3	TMEM37
3	TPD52
3	TUBE1
3	UCP2
3	VPS13A
3	WDR7
3	XYLT2
3	YIF1A
3	ZDHHC2
3	ZNF142
3	ZNF331
3	ZNF37A
3	ZNF7
3	ZNF92
4	AARS
4	ABCD1
4	ABHD11
4	ABHD4
4	ABHD5
4	ABHD8
4	ACN9
4	AFF1
4	AGMAT
4	AIG1
4	AKR1D1
4	ALDOC
4	ALG1
4	ALG11
4	APOOL
4	ARL4A
4	ARMC1
4	ARSA
4	B3GNTL1

4	B4GALT7
4	B9D1
4	BBS7
4	BCL9L
4	BTBD1
4	BTBD10
4	C12orf76
4	C15orf41
4	C2CD3
4	CARS2
4	CCDC64
4	CDAN1
4	CEMIP
4	CEP120
4	CEP192
4	CEP41
4	CERS1
4	CES2
4	CHPF2
4	CHRNA5
4	CHST13
4	CHST7
4	CHTF8
4	CLPTM1
4	CMTM7
4	CNIH4
4	CNN3
4	COMMD4
4	COQ4
4	COQ5
4	CRELD1
4	CSGALNACT2
4	CTSC
4	DEGS1
4	DFNB31
4	DLST
4	DNAJC30
4	DOLK
4	DZIP3
4	EDEM2
4	EMC6
4	ENGASE
4	ENO3

4	ERLIN1
4	FAM120C
4	FAM73A
4	FH
4	GANC
4	GLA
4	GMCL1
4	GMPPB
4	GPAA1
4	GPR157
4	GSTCD
4	HARS
4	HEPACAM
4	HEXB
4	HLF
4	HMGXB3
4	HOGA1
4	HPN
4	HSD17B2
4	IFT172
4	IMPACT
4	ISOC1
4	JADE2
4	KDELC2
4	KIAA2018
4	KLHL18
4	KLHL9
4	L2HGDH
4	LIPT1
4	LMAN2L
4	LRRC42
4	LRSAM1
4	MAN1A1
4	MANEAL
4	MARCH9
4	MATN3
4	MECR
4	MEST
4	METRN
4	METRNL
4	METTL21B
4	METTL6
4	MIPEP

4	MOGS
4	MPI
4	MPPE1
4	MREG
4	MRPS36
4	NAGPA
4	NDUFS7
4	NINL
4	NIPSNAP1
4	NMNAT1
4	NPHP3
4	NT5DC2
4	NUS1
4	NXN
4	OGDH
4	OGFOD3
4	OMA1
4	OPN3
4	OXLD1
4	PDLIM2
4	PEX10
4	PEX19
4	PGM2
4	PIGB
4	PIGC
4	PIGO
4	PIGZ
4	PILRB
4	PKD2
4	POC1A
4	POMGNT1
4	PRDM15
4	PRMT6
4	PRODH2
4	PRPS2
4	PRUNE
4	PSMG4
4	PTRH1
4	PYGL
4	PYURF
4	RAVER2
4	REEP4
4	RFT1

4	RHPN2
4	RIN1
4	RIN3
4	RPIA
4	RPUSD3
4	RTN4IP1
4	SAR1A
4	SEC24B
4	SGMS1
4	SH3BP5L
4	SH3GLB2
4	SHROOM1
4	SLC26A2
4	SLC26A6
4	SLC39A4
4	SLC8B1
4	SMIM12
4	SMIM15
4	SMTN
4	SMYD5
4	SNX11
4	SQRDL
4	SRD5A3
4	SSBP4
4	SSH3
4	STARD9
4	SYNE2
4	SYNJ2BP
4	TECPR1
4	TMEM126A
4	TMEM132A
4	TMEM181
4	TMEM261
4	TMEM60
4	TMEM62
4	TMTC4
4	TP53I13
4	TPP1
4	TRAPPC11
4	TRAPPC12
4	TRAPPC6A
4	TRIM35
4	TSPAN13

4	UBALD2
4	UFL1
4	UGP2
4	YY1AP1
4	ZBTB39
4	ZBTB41
4	ZCWPW1
4	ZKSCAN8
4	ZMYM4
4	ZNF133
4	ZNF232
4	ZNF248
4	ZNF354A
4	ZNF569
4	ZNF747
4	ZNF764
4	ZSCAN26
5	ANAPC15
5	APEX2
5	ATXN3
5	BAX
5	BRCA2
5	CBX2
5	CCNB1
5	CCNB1IP1
5	CCNF
5	CCNJ
5	CDC25C
5	CDCA3
5	CDK12
5	CDKN2C
5	CDT1
5	CENPA
5	CENPH
5	CENPI
5	CEP55
5	CHAF1A
5	CKAP2L
5	CLASRP
5	CLPX
5	COPS2
5	CTC1
5	CWC25

5	DDX39A
5	DHFR
5	DNAJA3
5	DTL
5	E2F2
5	E2F3
5	EME1
5	ENOPH1
5	ESCO2
5	ESPL1
5	FBXL17
5	FOXK1
5	GEMIN7
5	GINS1
5	GPS2
5	H2AFX
5	HDAC5
5	HDAC8
5	HDHD1
5	HIST1H2BD
5	HLTF
5	HMGA2
5	IQGAP3
5	KIF11
5	KIF4A
5	KIFC1
5	KPNA4
5	LANCL1
5	MAP2K6
5	MAT2A
5	MCL1
5	MKI67
5	MOCS3
5	MSH2
5	MT-ND1
5	MTFR2
5	MUS81
5	MYO1C
5	NDUFAF1
5	NEK2
5	NOLC1
5	NUP37
5	NUP54

5	OTUD4
5	PHF5A
5	PIP4K2B
5	PKMYT1
5	POLA2
5	PPIL2
5	PPP4C
5	PRC1
5	PRKRA
5	PSRC1
5	RAD23B
5	RB1
5	RBBP4
5	RBBP9
5	RBL1
5	RBL2
5	RBX1
5	RECQL
5	RHNO1
5	RNASEH2C
5	RPA1
5	SEC16A
5	SETD1B
5	SGOL1
5	SNRNP25
5	SNRPA1
5	STMN1
5	SUMO2
5	TAF1B
5	TCHP
5	TDP1
5	TFDP1
5	TIPIN
5	TRIM23
5	TRIM32
5	UBA3
5	UBE2A
5	UBE2D1
5	UBE2T
5	UBE2V1
5	UBE2Z
5	UBOX5
5	UHRF2

5	UMPS
5	USP15
5	WAPAL
5	XRCC3
6	AEN
6	AGFG2
6	ANKRD36B
6	APOBEC3C
6	ARHGEF26
6	ASPHD1
6	ASTE1
6	ATP5L
6	ATPIF1
6	B4GALNT4
6	BRF2
6	BYSL
6	C14orf119
6	C14orf159
6	C17orf80
6	CA5A
6	CAMKMT
6	CARKD
6	CCDC109B
6	CCDC137
6	CCDC77
6	CCDC86
6	CCDC9
6	CCDC97
6	CDK5RAP1
6	CDKN2AIPNL
6	CDYL
6	CENPQ
6	CISD3
6	CMC1
6	CTPS1
6	CTU1
6	DCAF16
6	DCUN1D3
6	DDX10
6	DDX28
6	DDX31
6	DDX55
6	DDX59

6	DHX35
6	DMTF1
6	DQX1
6	EEF1B2
6	EIF1AD
6	EIF4A3
6	ELAC1
6	ENC1
6	EPS8L2
6	ESPN
6	ETAA1
6	EXOSC2
6	FAM169A
6	FAM212B
6	FAM21A
6	FBXO4
6	FBXO9
6	FBXW9
6	FICD
6	FOXRED1
6	FUT10
6	GEMIN6
6	GEMIN8
6	GINS4
6	GM2A
6	GMEB2
6	GUF1
6	HEMK1
6	HPCAL1
6	IER5L
6	IGF2BP3
6	IMP3
6	INO80B
6	INO80D
6	INO80E
6	KCTD10
6	KHDRBS3
6	KIAA0196
6	KIAA0922
6	KLHL26
6	LATS2
6	LYAR
6	LYRM5

6	METTL1
6	METTL7B
6	MPC1
6	MPP6
6	MRI1
6	MRPL19
6	MRPL28
6	MRPL34
6	MRPL44
6	MRPL46
6	MRPS18A
6	MT-CO3
6	N4BP1
6	NDFIP1
6	NDFIP2
6	NDUFA8
6	NELFE
6	NHP2
6	NIFK
6	NIP7
6	NOM1
6	NOP10
6	NOP16
6	NOP56
6	OAF
6	ORMDL2
6	PDDC1
6	PGAM5
6	PHF20L1
6	PHF23
6	PIDD1
6	PITHD1
6	POLD3
6	POLR1A
6	POLR1C
6	POLR2H
6	POLR2L
6	POLR3K
6	PPAN
6	PPP1R8
6	PPP1R9A
6	PPP6R1
6	PPP6R2

6	PUS7L
6	PYROXD1
6	QTRT1
6	QTRTD1
6	R3HCC1
6	RAB21
6	RANBP6
6	RCN2
6	RNF126
6	RNF19B
6	RNF7
6	RNMTL1
6	RPL27
6	RPL39
6	RPP14
6	RPP25L
6	RPRD1B
6	RPS12
6	RPS15A
6	RPS23
6	RPS27L
6	RRP1
6	RRP7A
6	RRP8
6	RRS1
6	RTCA
6	SAV1
6	SAYSD1
6	SDCCAG3
6	SF3B6
6	SFXN4
6	SLC22A3
6	SLC7A6OS
6	SNAPC4
6	SPECC1
6	SRFBP1
6	SRRD
6	STEAP2
6	TAOK2
6	TCTA
6	THUMPD2
6	TIGD1
6	TIMM9

6	TMEM106C
6	TMEM19
6	TMEM97
6	TOE1
6	TRIM41
6	TRIT1
6	TRMT10C
6	UBBP4
6	UBE2F
6	UFM1
6	UTP14C
6	VBP1
6	VPRBP
6	WASH2P
6	WBSCR22
6	WDR70
6	WRAP73
6	ZBED5
6	ZC2HC1A
6	ZDHHC23
6	ZGRF1
6	ZHX1
6	ZNF101
6	ZNF358
6	ZNF445
6	ZNF473
6	ZNF514
6	ZNF557
6	ZNF574
6	ZNF777
6	ZNF788
6	ZNHIT3
6	ZNRD1

Table S12. GO terms and KEGG pathways of STRING clusters of mRNAs regulated by miR-222-5p.

STRING Cluster	Category	Term	Description	p-value	Genes
1	**GO Term Cellular Component**	GO:0016592	mediator complex	6.13E-05	RBM14, MED30, MED9, MED29, MED18, MED17
	**GO Term Molecular Function**	GO:0001104	RNA polymerase II transcription cofactor activity	6.98E-05	RBM14, MED30, CNOT2, MED9, MED18, MED17
	**GO Term Biological Process**	GO:0006397	mRNA processing	1.40E-04	NUDT21, MBNL2, PAN3, GEMIN2, PAPD5, FMR1, ECD, SRSF5, SCAF1, SRRM1

	**GO Term Cellular Component**	GO:0005634	nucleus	9.43E-04	FMR1, GEMIN2, ZBTB20, IFI35, PTPDC1, IKZF4, MED17, ZNF407, PAPOLG, ADNP2, ZNF600, NUDT16, LMCD1, HMGN3, RNF111, RBM14, CDKL5, MBNL2, GLRX3, MAMSTR, CEP131, ZNF12, TRNAU1AP, SRRM1, TAF6L, RAD51D, MORF4L2, RFX5, WAC, FAM193B, SRSF5, C19ORF66, CGGBP1, ZNF672, NOTCH2, HOMEZ, DDX1, DDX23, SPATA2, GTF2E1, LAGE3, PSMC3IP, MED30, S100A13, ZNF784, HES4, HEXIM2, PCGF6, POU2F1, MYEF2, CNBP, ECD, FOXJ2, TPRKB, NR2F1, SCAF1, HIPK3, TBX2, ZBTB9, SKI, NUDT21, ZNFX1, TMEM115, CNOT2, FAM175A, RANGRF, S100P, NAA16, CENPBD1, REEP6, PKIG, DCP1A, ZNF136, RCOR2, TAF1

	**GO Term Biological Process**	GO:0006351	transcription, DNA-templated	0.00114391	ZNF672, HOMEZ, DDX1, ZBTB20, IKZF4, ZNF407, DPF1, ADNP2, E4F1, ZNF600, LMCD1, ZNF784, HES4, HEXIM2, RBM14, PCGF6, POU2F1, MYEF2, MAMSTR, CNBP, NR2F1, MED9, ZNF12, MED29, TBX2, ZBTB9, SKI, MORF4L2, CNOT2, RFX5, WAC, TADA1, BCORL1, RCOR2, CGGBP1

	**GO Term Cellular Component**	GO:0005654	nucleoplasm	0.00119285	CUEDC2, FMR1, GEMIN2, ZBTB20, PTPDC1, IKZF4, MED17, PAPOLG, NUDT16, LMCD1, HMGN3, RNF111, RBM14, CDKL5, USP2, HAUS3, ZNF12, MED29, SRRM1, RAD51D, MORF4L2, WAC, FAM193B, PPIH, SRSF5, BCORL1, CGGBP1, MCPH1, NOTCH2, DDX1, DDX23, GTF2E1, TMEM70, MED30, DNAJB4, E4F1, NRBF2, NR2C2AP, HEXIM2, POU2F1, MYEF2, ECD, NR2F1, SKI, NUDT21, FAM175A, TADA1, RANGRF, INTS6, TAF1
	**KEGG Pathway**	hsa03018	RNA degradation	0.00121774	PAN3, CNOT2, PAPD5, NUDT16, DCP1A

	**GO Term Biological Process**	GO:0006367	transcription initiation from RNA polymerase II promoter	0.00158464	NOTCH2, MED30, NR2F1, NRBF2, GTF2E1, MED17, TAF1, NR2C2AP
	**GO Term Biological Process**	GO:0008380	RNA splicing	0.00295772	MBNL2, GEMIN2, FMR1, ECD, DDX23, NOL3, SCAF1, SRRM1
	**GO Term Biological Process**	GO:0000122	negative regulation of transcription from RNA polymerase II promoter	0.00634109	HOMEZ, HEXIM2, PCGF6, USP2, NR2F1, ZBTB20, TBX2, SKI, CNOT2, RFX5, E4F1, PKIG, LMCD1, ZNF136, RCOR2, CGGBP1
	**GO Term Molecular Function**	GO:0003677	DNA binding	0.00854655	ZNF672, HOMEZ, RABGAP1, DDX1, ZBTB20, PSMC3IP, ZNF407, ZNF768, ADNP2, E4F1, ZNF600, HMGN3, ZNF784, HES4, PCGF6, MYEF2, CNBP, ZNF12, SRRM1, TBX2, ZBTB9, TAF6L, RAD51D, PAPD5, RFX5, CENPBD1, ZNF136, RCOR2

	**GO Term Cellular Component**	GO:0005667	transcription factor complex	0.00858483	SKI, RBM14, NAA16, RCOR2, MED17, TAF1, TBX2
	**GO Term Biological Process**	GO:0043330	response to exogenous dsRNA	0.01419145	RIPK2, DDX1, CARD9
	**GO Term Molecular Function**	GO:0030374	ligand-dependent nuclear receptor transcription coactivator activity	0.02404127	PSMC3IP, RBM14, MED30, MED17
	**KEGG Pathway**	hsa03022	Basal transcription factors	0.0302392	TAF6L, GTF2E1, TAF1

	**GO Term Cellular Component**	GO:0005737	cytoplasm	0.03364923	CUEDC2, ANKRD13D, FMR1, GEMIN2, MPV17L, PTPDC1, TTC28, IPO9, PAPOLG, DPF1, EPG5, NUDT16, LMCD1, HMGN3, RNF111, RBM14, CDKL5, MBNL2, RIPK2, TRNAU1AP, IL17RE, ATXN2L, RNF144B, RAD51D, PAPD5, HMBS, FAM193B, PPIH, SRSF5, C19ORF66, BCORL1, MCPH1, HOMEZ, DDX1, SPATA2, TWF1, NOL3, GTF2E1, VCPIP1, SEC14L1, DNAJB4, S100A13, LRRC8D, E4F1, LRIG2, NRBF2, WHAMM, NAA50, HEXIM2, ANKRD27, MYEF2, CCZ1B, CARD9, ECD, TPRKB, HIPK3, SKI, TOLLIP, CNOT2, TADA1, RANGRF, S100P, CCM2, NAA16, PKIG, DCP1A

	**GO Term Cellular Component**	GO:0005813	centrosome	0.03424058	SKI, NUDT21, RAD51D, RABGAP1, CEP131, USP2, HAUS3, ZNF12, HAUS5, IKZF4, TTC28
	**GO Term Molecular Function**	GO:0046966	thyroid hormone receptor binding	0.03812749	MED30, HMGN3, MED17
	**GO Term Biological Process**	GO:0048845	venous blood vessel morphogenesis	0.03907754	HEG1, CCM2
	**GO Term Biological Process**	GO:0000375	RNA splicing, via transesterification reactions	0.039507	GEMIN2, DDX23, SRRM1
	**GO Term Molecular Function**	GO:0042802	identical protein binding	0.04280087	HEXIM2, MRFAP1L1, RIPK2, FMR1, USP2, FOXJ2, NOL3, NUDT21, TMEM115, VWA1, NUDT16, DCP1A, CGGBP1, MCPH1
	**GO Term Biological Process**	GO:0006369	termination of RNA polymerase II transcription	0.04654127	NUDT21, SRSF5, MED18, SRRM1

2	**GO Term Molecular Function**	GO:0005515	protein binding	3.97E-10	CYFIP2, COL16A1, RTKN, SERPINE2, EPO, NCF2, IRS1, GFI1, NAB2, HP, ZBTB22, F11R, ETS2, LGALS3, STK11, GOLGA1, TRIM25, ARFIP2, FBXO3, SOX9, OSTF1, SOX4, AP2M1, SH3GL1, DNAAF2, VPS33B, TAP2, TAP1, MUL1, ACYP2, HNF1A, DKK1, SAP30, UHMK1, TOM1L1, ADAM15, PKP4, RAF1, ATF5, PFN1, ZFPM1, STX1A, ARL8B, TNFRSF11A, GLIS2, TMEM25, RIC8A, SDCBP, PRDX4, VTI1A, PACSIN2, FADD, ZBTB7B, PROM1, ZBTB17, UVRAG, HSPA8, TOR1A, TGFB1, ZBTB14, UBAC1, PRRC2A, PPP5C, AGO2, ID1, PDCL, CEP83, TAB2, MON1B, BMPR1A, BCAR3, MTR, PIK3CB, GPHN, AP4M1, NUDCD1, GPER1, SUFU, MLST8, FLOT1, CIC, RAB8A, NKX3-1, BCAS3, ITGA2, TESC, RRAS2, GPX7, APOA1, PAX6, ETV4, ZDHHC17, TOPORS, F5, NR5A1, PLSCR1, TRAF3, TRAF6, RARA, EXOC4, PIK3C3, CD46, CHMP7, SHANK2, VAMP2, CEBPA, PRKAA1, SRC, NEDD9, MAT2B, HSPA13, PPP2CB, RAB23, SPP1, STOM, RICTOR, CAMK2G, EIF4E, AASDHPPT, MPZL2, DAB2IP, HIP1R, RANBP9, SORBS1, MERTK, AGT, HIPK2, PNRC2, TRIM39, AXL, SPRY2, AGGF1, UBAP1, FAS, STUB1, LAMTOR1, BCAR1, PTPN2, TNRC6A

	**GO Term Cellular Component**	GO:0005829	cytosol	1.36E-09	CYFIP2, RTKN, SERPINE2, GLDC, NCF2, IRS1, DGKA, MTR, PIK3CB, AP4M1, STK11, HERC1, SUFU, GOLGA1, MLST8, TRIM25, FBXO3, ARFIP2, PIP4K2C, AP2M1, RAB8A, APOA1, TOM1L1, SLC7A5, PLSCR1, DDAH1, TRAF3, TRAF6, RBP1, EXOC4, PIK3C3, RAF1, PFN1, CHMP7, SGK2, STX1A, VAMP2, NGEF, PRKAA1, PDXK, SRC, MAT2B, PAPSS1, PPP2CB, SDCBP, PRDX4, UAP1L1, VTI1A, PACSIN2, RICTOR, FADD, CAMK2G, EIF4E, AASDHPPT, HSPA8, DAB2IP, RANBP9, SORBS1, PPP5C, TRIM39, AGO2, PDCL, SPRY2, CEP83, UBAP1, FAS, STUB1, TAB2, TNRC6A, BCAR1, MSRB1

	**GO Term Cellular Component**	GO:0070062	extracellular exosome	9.09E-07	CYFIP2, LGALS3BP, PROS1, HP, ARPC5L, F11R, AP4M1, LGALS3, STK11, FLOT1, OSTF1, PIP4K2C, AP2M1, RAB8A, SLC6A14, RRAS2, APOA1, TOM1L1, SLC7A5, PLSCR1, DDAH1, ADAM15, CD46, PFN1, VAMP2, ARL8B, CFD, LAMA5, CPB2, PDXK, SRC, MAT2B, BPHL, EVPL, HSPA13, PPP2CB, SDCBP, PRDX4, RAB23, SPP1, PACSIN2, STOM, PROM1, EIF4E, AASDHPPT, HSPA8, TOR1A, DAB2IP, UBAC1, AGT, PRRC2A, AXL, CD7, FAS, STUB1, ESAM, LAMTOR1

	**KEGG Pathway**	hsa04150	mTOR signaling pathway	1.21E-05	STK11, PRKAA1, IRS1, RPS6KB2, MLST8, RICTOR, PIK3CB, EIF4E
	**GO Term Molecular Function**	GO:0019900	kinase binding	1.86E-05	CEBPA, PRKAA1, SRC, DAB2IP, FAS, STUB1, ATF5, STX1A
	**GO Term Biological Process**	GO:0043491	protein kinase B signaling	2.84E-05	TGFB1, AXL, RPS6KB2, SOX9, MERTK, NKX3-1
	**GO Term Cellular Component**	GO:0072562	blood microparticle	3.40E-05	LGALS3BP, HSPA8, SDCBP, TGFB1, PROS1, HP, STOM, APOA1, PFN1, AGT
	**GO Term Biological Process**	GO:0045893	positive regulation of transcription, DNA-templated	5.72E-05	ZBTB17, TGFB1, EPO, SRC, TESC, PAX6, HNF1A, ETS2, TOPORS, AGT, HIPK2, GLIS2, SOX18, RARA, SOX9, ATF5, SOX4, NKX3-1

	**GO Term Biological Process**	GO:0045944	positive regulation of transcription from RNA polymerase II promoter	7.73E-05	CEBPA, ONECUT1, ETS2, GLIS2, SOX18, GPER1, FADD, SOX9, SOX4, NKX3-1, BCAS3, TGFB1, DAB2IP, PAX6, HNF1A, ETV4, HIPK2, NR5A1, PLSCR1, TRAF6, AGO2, RARA, RAF1, ATF5, PFN1, BMPR1A
	**GO Term Biological Process**	GO:0007229	integrin-mediated signaling pathway	1.11E-04	LAMA5, COL16A1, ADAM15, SRC, ITGA2, NEDD9, APOA1, BCAR1

	**GO Term Cellular Component**	GO:0005886	plasma membrane	1.25E-04	GLDC, IRS1, DGKA, PROS1, PIK3CB, F11R, GPHN, ETS2, PANX1, LGALS3, FCGRT, GPER1, FLOT1, ARFIP2, AP2M1, RAB8A, TESC, ITGA2, SLC6A14, RRAS2, APOA1, DKK1, F5, SLC7A5, PLSCR1, ADAM15, TRAF6, EXOC4, PKP4, TLCD1, C9ORF3, RAF1, CD46, STX1A, SHANK2, VAMP2, SRC, TNFRSF11A, TFPI, TMEM25, RIC8A, SDCBP, RAB23, NCR3LG1, FADD, PROM1, CAMK2G, HSPA8, TGFB1, DAB2IP, RANBP9, SORBS1, MERTK, UBAC1, PPP5C, AXL, CD7, SPRY2, UBAP1, FAS, STUB1, ESAM, TAB2, LAMTOR1, PTPN2, BCAR1, BMPR1A

	**GO Term Biological Process**	GO:0042593	glucose homeostasis	1.26E-04	CEBPA, STK11, PRKAA1, IRS1, PAX6, HNF1A, SOX4, PTPN2

	**GO Term Cellular Component**	GO:0005737	cytoplasm	1.30E-04	CYFIP2, NCF2, IRS1, ARPC5L, MTR, GPHN, ETS2, LGALS3, STK11, NUDCD1, HERC1, GPER1, SUFU, MLST8, TRIM25, FBXO3, ARFIP2, OSTF1, SOX4, SH3GL1, BCAS3, DNAAF2, TESC, VPS33B, MUL1, PAX6, HNF1A, ZDHHC17, TOM1L1, SLC7A5, TRAF6, RBP1, RARA, EXOC4, PKP4, C9ORF3, RAF1, ATF5, PFN1, ZFPM1, SHANK2, ARL8B, PRKAA1, PDXK, SRC, NEDD9, BPHL, EVPL, GLIS2, RIC8A, PPP2CB, SDCBP, RAB23, PACSIN2, STOM, FADD, EIF4E, UVRAG, TGFB1, ZBTB14, DAB2IP, HIP1R, RANBP9, SORBS1, MERTK, UBAC1, AGT, HIPK2, PRRC2A, PPP5C, AGO2, RPS6KB2, PDCL, AGGF1, UBAP1, FAS, STUB1, NES, BCAR1, MON1B

	**GO Term Biological Process**	GO:0000122	negative regulation of transcription from RNA polymerase II promoter	1.37E-04	CEBPA, TGFB1, ZBTB14, EPO, GFI1, DAB2IP, PAX6, HNF1A, DKK1, ETS2, HIPK2, SAP30, GLIS2, SOX18, SUFU, TRAF6, ID1, RARA, CIC, ZFPM1, PTPN2
	**GO Term Biological Process**	GO:0032940	secretion by cell	1.49E-04	SERPINE2, AXL, MERTK, STX1A
	**GO Term Biological Process**	GO:0010628	positive regulation of gene expression	1.59E-04	PRKAA1, PLSCR1, TGFB1, GPER1, TESC, ID1, SPRY2, PAX6, PIK3CB, CD46, ZBTB7B, NKX3-1
	**GO Term Cellular Component**	GO:0005913	cell-cell adherens junction	1.68E-04	HSPA8, SRC, DAB2IP, SORBS1, F11R, EVPL, SDCBP, FLOT1, PACSIN2, TRIM25, ARFIP2, PFN1, SH3GL1
	**GO Term Biological Process**	GO:0030168	platelet activation	2.83E-04	PLSCR1, SRC, AXL, DGKA, PIK3CB, RAF1, MERTK, F5

	**GO Term Molecular Function**	GO:0001077	transcriptional activator activity, RNA polymerase II core promoter proximal region sequence-specific binding	2.95E-04	CEBPA, PLSCR1, SOX18, ONECUT1, PAX6, SOX9, HNF1A, ZBTB7B, ETV4, SOX4, GLIS2
	**GO Term Molecular Function**	GO:0019899	enzyme binding	3.32E-04	HSPA8, TGFB1, GLDC, SRC, RANBP9, APOA1, MAT2B, NR5A1, PLSCR1, RARA, STUB1, RAF1, EIF4E
	**GO Term Molecular Function**	GO:0017124	SH3 domain binding	3.49E-04	TOM1L1, UVRAG, PLSCR1, ADAM15, DAB2IP, OSTF1, BCAR1, SHANK2
	**GO Term Biological Process**	GO:0008285	negative regulation of cell proliferation	4.58E-04	CEBPA, TGFB1, SERPINE2, TESC, DAB2IP, STK11, GPER1, RARA, SPRY2, RAF1, ATF5, SOX4, PTPN2, NKX3-1
	**GO Term Biological Process**	GO:0007179	transforming growth factor beta receptor signaling pathway	5.37E-04	TGFB1, SRC, ID1, APOA1, F11R, BMPR1A, HIPK2

	**KEGG Pathway**	hsa04151	PI3K-Akt signaling pathway	7.62E-04	LAMA5, PRKAA1, EPO, IRS1, ITGA2, PIK3CB, PPP2CB, STK11, RPS6KB2, SPP1, MLST8, RAF1, EIF4E, SGK2
	**GO Term Biological Process**	GO:0071260	cellular response to mechanical stimulus	0.001112	TGFB1, ITGA2, FAS, FADD, SOX9, AGT
	**GO Term Biological Process**	GO:0048015	phosphatidylinositol-mediated signaling	0.00113095	IRS1, AGO2, MLST8, PIK3C3, RICTOR, PIK3CB, TNRC6A
	**GO Term Biological Process**	GO:0048010	vascular endothelial growth factor receptor signaling pathway	0.00118447	CYFIP2, NCF2, SRC, AXL, PIK3CB, BCAR1
	**GO Term Biological Process**	GO:0034260	negative regulation of GTPase activity	0.00125831	BCAS3, RTKN, DAB2IP, SPRY2, F11R
	**GO Term Biological Process**	GO:0045892	negative regulation of transcription, DNA-templated	0.0012911	HSPA8, CEBPA, TGFB1, ZBTB14, SRC, GFI1, DAB2IP, GLIS2, SOX18, TRAF6, ID1, RARA, SOX9, ATF5, NKX3-1
	**GO Term Molecular Function**	GO:0098641	cadherin binding involved in cell-cell adhesion	0.001442	HSPA8, SDCBP, SRC, DAB2IP, PACSIN2, TRIM25, ARFIP2, PFN1, F11R, EVPL, SH3GL1

	**GO Term Biological Process**	GO:0032147	activation of protein kinase activity	0.00149342	TOM1L1, STK11, EPO, TRAF6, NKX3-1
	**GO Term Biological Process**	GO:0071222	cellular response to lipopolysaccharide	0.00157241	SRC, AXL, GFI1, TRAF6, DAB2IP, RARA, TFPI
	**GO Term Molecular Function**	GO:0004871	signal transducer activity	0.00193641	PPP5C, TRAF3, IRS1, SUFU, FAS, MUL1, ZDHHC17, DKK1, BCAR1
	**GO Term Biological Process**	GO:0050731	positive regulation of peptidyl-tyrosine phosphorylation	0.00211465	STK11, TGFB1, SRC, MLST8, RICTOR, AGT
	**GO Term Biological Process**	GO:0007165	signal transduction	0.00213841	BCAR3, LGALS3BP, PRKAA1, RTKN, EPO, IRS1, SRC, NEDD9, TNFRSF11A, PIK3CB, SUFU, FADD, SOX9, OSTF1, SH3GL1, CHRNB1, ZDHHC17, TOM1L1, TRAF3, AXL, RPS6KB2, RARA, PDCL, FAS, RAF1
	**KEGG Pathway**	hsa04810	Regulation of actin cytoskeleton	0.00217939	CYFIP2, SRC, ITGA2, ARPC5L, RRAS2, PIK3CB, PIP4K2C, PFN1, RAF1, BCAR1

	**GO Term Biological Process**	GO:0050900	leukocyte migration	0.00231956	SLC7A5, SRC, PROS1, ESAM, PIK3CB, F11R, MERTK
	**KEGG Pathway**	hsa04610	Complement and coagulation cascades	0.00241456	CFD, CPB2, PROS1, CD46, TFPI, F5
	**GO Term Cellular Component**	GO:0048471	perinuclear region of cytoplasm	0.0024623	CYFIP2, SRC, ITGA2, VPS33B, HIP1R, GPER1, TRAF6, GOLGA1, SPP1, VTI1A, RARA, AGGF1, STOM, PKP4, EIF4E, VAMP2
	**GO Term Biological Process**	GO:0042981	regulation of apoptotic process	0.00252828	TGFB1, TRAF3, FAS, FADD, SOX9, TNFRSF11A, RAF1, SGK2, BCAR1
	**GO Term Biological Process**	GO:0018105	peptidyl-serine phosphorylation	0.00261995	MORC3, EPO, SRC, RICTOR, SGK2, HIPK2, UHMK1
	**KEGG Pathway**	hsa04910	Insulin signaling pathway	0.00269082	PRKAA1, IRS1, RPS6KB2, FLOT1, SORBS1, PIK3CB, RAF1, EIF4E
	**GO Term Biological Process**	GO:0031018	endocrine pancreas development	0.00283855	ONECUT1, SOX9, HNF1A, SOX4
	**GO Term Molecular Function**	GO:0030295	protein kinase activator activity	0.00283959	TOM1L1, STK11, EPO, NKX3-1

	**GO Term Cellular Component**	GO:0005925	focal adhesion	0.00294828	HSPA8, SDCBP, ITGA2, ARPC5L, FLOT1, PACSIN2, RRAS2, NEDD9, SORBS1, PFN1, CD46, BCAR1
	**GO Term Biological Process**	GO:0098609	cell-cell adhesion	0.00317127	HSPA8, SDCBP, DAB2IP, PACSIN2, TRIM25, ARFIP2, PFN1, F11R, EVPL, SH3GL1
	**GO Term Biological Process**	GO:0050680	negative regulation of epithelial cell proliferation	0.00335173	TGFB1, DAB2IP, PAX6, SOX9, NKX3-1
	**GO Term Biological Process**	GO:0070373	negative regulation of ERK1 and ERK2 cascade	0.00380636	GPER1, DAB2IP, RANBP9, SPRY2, PTPN2
	**GO Term Biological Process**	GO:0008584	male gonad development	0.00382756	NR5A1, PRDX4, ADAM15, TESC, SOX9, NKX3-1
	**GO Term Biological Process**	GO:0050821	protein stabilization	0.00397595	MORC3, TESC, HIP1R, MUL1, APOA1, PFN1, SOX4
	**GO Term Biological Process**	GO:0060627	regulation of vesicle-mediated transport	0.00413048	UVRAG, PRKAA1, VAMP2
	**GO Term Molecular Function**	GO:0047485	protein N-terminus binding	0.00437962	SDCBP, TGFB1, TRAF6, ID1, EXOC4, STX1A

	**GO Term Cellular Component**	GO:0005901	caveola	0.00490035	IRS1, SRC, FLOT1, PACSIN2, BMPR1A
	**GO Term Biological Process**	GO:0043534	blood vessel endothelial cell migration	0.00512582	SOX18, ID1, APOA1
	**GO Term Biological Process**	GO:0016337	single organismal cell-cell adhesion	0.00519388	CYFIP2, SRC, PKP4, ESAM, SOX9, MPZL2
	**GO Term Molecular Function**	GO:0046982	protein heterodimerization activity	0.00551704	TGFB1, ITGA2, HNF1A, PANX1, SDCBP, SOX18, AXL, FLOT1, RARA, SOX9, RAF1, SOX4, STX1A
	**GO Term Biological Process**	GO:0010634	positive regulation of epithelial cell migration	0.00563005	SRC, ITGA2, SOX9, PFN1
	**GO Term Biological Process**	GO:0070555	response to interleukin-1	0.00563005	EPO, SRC, TRAF6, TNFRSF11A
	**GO Term Biological Process**	GO:0002576	platelet degranulation	0.00564042	LGALS3BP, CFD, TGFB1, PROS1, APOA1, F5
	**GO Term Cellular Component**	GO:0044798	nuclear transcription factor complex	0.00570506	SOX18, SOX9, SOX4

	**KEGG Pathway**	hsa05205	Proteoglycans in cancer	0.00581863	TGFB1, SRC, ITGA2, RPS6KB2, RRAS2, FAS, PIK3CB, RAF1, CAMK2G
	**GO Term Cellular Component**	GO:0005856	cytoskeleton	0.00610518	SDCBP, TOR1A, HIP1R, PACSIN2, STOM, SPRY2, PKP4, PFN1, EVPL, GPHN, MPZL2
	**GO Term Molecular Function**	GO:0005178	integrin binding	0.0061171	LAMA5, COL16A1, ADAM15, SRC, ITGA2, PTPN2
	**GO Term Biological Process**	GO:0043065	positive regulation of apoptotic process	0.00613628	TGFB1, SRC, GPER1, TRAF6, DAB2IP, HIP1R, FAS, FADD, SOX4, NGEF
	**GO Term Cellular Component**	GO:0045121	membrane raft	0.00628098	SDCBP, PLSCR1, FLOT1, FAS, STOM, FADD, SORBS1, LAMTOR1
	**GO Term Cellular Component**	GO:0043231	intracellular membrane-bounded organelle	0.00634239	IRS1, HIP1R, HSPA13, BPHL, ZDHHC17, PRRC2A, SLC7A5, PPP5C, GOLGA1, PACSIN2, LAMTOR1, PROM1, VAMP2, TNRC6A

	**KEGG Pathway**	hsa04152	AMPK signaling pathway	0.00654154	PPP2CB, STK11, PRKAA1, IRS1, RPS6KB2, PIK3CB, RAB8A
	**GO Term Biological Process**	GO:0071902	positive regulation of protein serine/threonine kinase activity	0.00664613	STK11, SRC, DAB2IP, SPRY2
	**GO Term Biological Process**	GO:0016192	vesicle-mediated transport	0.00686345	AP4M1, BCAS3, VPS33B, STX1A, AP2M1, VAMP2, MON1B
	**KEGG Pathway**	hsa05200	Pathways in cancer	0.00691859	LAMA5, CEBPA, TGFB1, ITGA2, PIK3CB, TRAF3, SUFU, TRAF6, RARA, FAS, FADD, RAF1, NKX3-1
	**KEGG Pathway**	hsa05221	Acute myeloid leukemia	0.00698314	CEBPA, RPS6KB2, RARA, PIK3CB, RAF1
	**GO Term Biological Process**	GO:0042059	negative regulation of epidermal growth factor receptor signaling pathway	0.00719235	DAB2IP, SPRY2, AP2M1, PTPN2
	**GO Term Biological Process**	GO:0001944	vasculature development	0.00740982	RIC8A, STK11, SOX18

	**GO Term Biological Process**	GO:0045087	innate immune response	0.00824553	LGALS3, ADAM15, NCF2, TRAF3, SRC, AXL, GPER1, DAB2IP, TRIM25, FADD, CD46, MSRB1
	**GO Term Biological Process**	GO:0034446	substrate adhesion-dependent cell spreading	0.00836261	LAMA5, SRC, AXL, MERTK
	**GO Term Biological Process**	GO:0001942	hair follicle development	0.00836261	LAMA5, SOX18, SOX9, DKK1
	**GO Term Biological Process**	GO:0007398	ectoderm development	0.00869399	ZBTB17, ZBTB7B, BMPR1A
	**GO Term Biological Process**	GO:0051457	maintenance of protein location in nucleus	0.00869399	NR5A1, MORC3, TOPORS
	**GO Term Molecular Function**	GO:0042802	identical protein binding	0.00877864	NAB2, DAB2IP, MUL1, APOA1, ZDHHC17, CLDN6, SDCBP, PPP5C, TRIM39, TRAF6, PACSIN2, FAS, ARFIP2, FADD, PIP4K2C, RAF1, SH3GL1

	**GO Term Molecular Function**	GO:0019901	protein kinase binding	0.00898384	TOM1L1, TRAF3, SUFU, TRAF6, DAB2IP, SPRY2, PAX6, RICTOR, RAB8A, PTPN2, BCAR1
	**GO Term Biological Process**	GO:0043123	positive regulation of I-kappaB kinase/NF-kappaB signaling	0.00899884	PPP5C, TRAF6, TRIM25, MUL1, TAB2, FADD, ZDHHC17
	**GO Term Molecular Function**	GO:0044212	transcription regulatory region DNA binding	0.00943173	CEBPA, ZBTB14, SOX18, GFI1, HNF1A, ATF5, GLIS2, NKX3-1
	**GO Term Cellular Component**	GO:0030054	cell junction	0.0095807	CYFIP2, CHRNB1, PRR7, TOR1A, GPER1, PKP4, F11R, ZDHHC17, GPHN, STX1A, VAMP2, SHANK2
	**GO Term Biological Process**	GO:0010508	positive regulation of autophagy	0.00963834	UVRAG, STK11, PRKAA1, PIK3CB
	**GO Term Biological Process**	GO:0030218	erythrocyte differentiation	0.00963834	EPO, ZFPM1, PTPN2, HIPK2
	**KEGG Pathway**	hsa04068	FoxO signaling pathway	0.00980777	STK11, PRKAA1, TGFB1, IRS1, PIK3CB, RAF1, SGK2

	**GO Term Biological Process**	GO:0032869	cellular response to insulin stimulus	0.01033473	IRS1, SRC, SORBS1, RAB8A, VAMP2
	**GO Term Biological Process**	GO:0001843	neural tube closure	0.01033473	TGFB1, SUFU, TRAF6, RARA, PFN1
	**GO Term Biological Process**	GO:0030308	negative regulation of cell growth	0.01094225	STK11, TGFB1, SERPINE2, ADAM15, MUL1, AGT
	**GO Term Cellular Component**	GO:0032587	ruffle membrane	0.01103063	SRC, TESC, HIP1R, PACSIN2, SPRY2
	**GO Term Biological Process**	GO:0043277	apoptotic cell clearance	0.01153569	AXL, RARA, MERTK
	**GO Term Biological Process**	GO:0001503	ossification	0.01177618	TRAF6, SPP1, OSTF1, SOX9, TNFRSF11A
	**GO Term Biological Process**	GO:0046718	viral entry into host cell	0.01177618	UVRAG, AXL, ITGA2, F11R, CD46
	**GO Term Biological Process**	GO:0001525	angiogenesis	0.01191742	LAMA5, BCAS3, ADAM15, SOX18, ID1, DAB2IP, AGGF1, HS6ST1
	**KEGG Pathway**	hsa04550	Signaling pathways regulating pluripotency of stem cells	0.01201301	ID1, ONECUT1, PAX6, PIK3CB, HNF1A, RAF1, BMPR1A

	**GO Term Biological Process**	GO:0006468	protein phosphorylation	0.01241461	STK11, PRKAA1, MORC3, TGFB1, RPS6KB2, RARA, PIK3C3, RAF1, MERTK, SGK2, BMPR1A, HIPK2
	**GO Term Cellular Component**	GO:0043005	neuron projection	0.01302665	CYFIP2, SRC, FAS, FADD, PFN1, STX1A, VAMP2, SHANK2
	**GO Term Biological Process**	GO:0048011	neurotrophin TRK receptor signaling pathway	0.01308894	SRC, RAF1, BCAR1
	**GO Term Biological Process**	GO:0032418	lysosome localization	0.01308894	VPS33B, LAMTOR1, ARL8B
	**GO Term Biological Process**	GO:0006919	activation of cysteine-type endopeptidase activity involved in apoptotic process	0.01334079	HIP1R, FAS, MUL1, FADD, NKX3-1
	**GO Term Cellular Component**	GO:0014069	postsynaptic density	0.0134235	SRC, GPER1, HIP1R, PKP4, CAMK2G, RAB8A, SHANK2
	**GO Term Cellular Component**	GO:0031012	extracellular matrix	0.0134369	LGALS3BP, HSPA8, LGALS3, LAMA5, PLSCR1, TGFB1, SERPINE2, NES, RAB8A

	**GO Term Molecular Function**	GO:0008134	transcription factor binding	0.01359587	CEBPA, BCAS3, SUFU, ID1, NAB2, RARA, PAX6, ZFPM1, NKX3-1
	**KEGG Pathway**	hsa05142	Chagas disease (American trypanosomiasis)	0.01360992	PPP2CB, TGFB1, TRAF6, FAS, FADD, PIK3CB
	**GO Term Biological Process**	GO:0051897	positive regulation of protein kinase B signaling	0.01389029	TGFB1, SRC, AXL, SPRY2, RICTOR
	**GO Term Molecular Function**	GO:0000149	SNARE binding	0.01411986	UVRAG, VTI1A, STX1A, VAMP2
	**GO Term Biological Process**	GO:0008284	positive regulation of cell proliferation	0.0143883	SDCBP, PRKAA1, TGFB1, IRS1, EPO, GPER1, RARA, SOX9, TNFRSF11A, SOX4, HIPK2, NKX3-1
	**KEGG Pathway**	hsa04620	Toll-like receptor signaling pathway	0.01468338	TRAF3, TRAF6, SPP1, TAB2, FADD, PIK3CB
	**GO Term Biological Process**	GO:0048535	lymph node development	0.01472767	TGFB1, FADD, TNFRSF11A
	**GO Term Biological Process**	GO:1902041	regulation of extrinsic apoptotic signaling pathway via death domain receptors	0.01472767	LGALS3, FAS, FADD

	**GO Term Molecular Function**	GO:0008022	protein C-terminus binding	0.01567183	SDCBP, PPP2CB, PRKAA1, NCF2, SRC, ID1, AGO2
	**GO Term Cellular Component**	GO:1903561	extracellular vesicle	0.01569962	SDCBP, SERPINE2, APOA1, F5
	**GO Term Biological Process**	GO:0051092	positive regulation of NF-kappaB transcription factor activity	0.01593499	TGFB1, TRAF6, TRIM25, TAB2, TNFRSF11A, AGT
	**GO Term Biological Process**	GO:0007507	heart development	0.0161555	TGFB1, ID1, TAB2, RAF1, ZFPM1, SOX4, NKX3-1
	**GO Term Cellular Component**	GO:0008021	synaptic vesicle	0.01625673	TOR1A, VTI1A, STX1A, RAB8A, VAMP2
	**GO Term Biological Process**	GO:0033280	response to vitamin D	0.01644983	TGFB1, SPP1, TRIM25
	**GO Term Biological Process**	GO:0033189	response to vitamin A	0.01644983	EPO, RBP1, RARA
	**GO Term Biological Process**	GO:0007596	blood coagulation	0.01655609	CPB2, SERPINE2, ITGA2, PROS1, TFPI, ZFPM1, F5
	**GO Term Biological Process**	GO:0007565	female pregnancy	0.01685269	TGFB1, COL16A1, ITGA2, RARA, AGT
	**GO Term Biological Process**	GO:0042127	regulation of cell proliferation	0.01696084	LAMA5, SRC, FAS, SOX9, SGK2, TOPORS, AGT

	**GO Term Cellular Component**	GO:0045111	intermediate filament cytoskeleton	0.0174343	BCAS3, STUB1, NES, EVPL
	**GO Term Cellular Component**	GO:0001750	photoreceptor outer segment	0.0174343	HNF1A, PROM1, MERTK, SHANK2
	**GO Term Molecular Function**	GO:0016301	kinase activity	0.01758181	STK11, SRC, DGKA, PACSIN2, PIK3C3, TAB2, PIK3CB, RAF1
	**GO Term Biological Process**	GO:0045879	negative regulation of smoothened signaling pathway	0.0182534	SERPINE2, SUFU, GLIS2
	**GO Term Biological Process**	GO:0090190	positive regulation of branching involved in ureteric bud morphogenesis	0.0182534	TGFB1, SOX9, AGT
	**GO Term Molecular Function**	GO:0043548	phosphatidylinositol 3-kinase binding	0.01825739	IRS1, AXL, DAB2IP
	**GO Term Cellular Component**	GO:0043025	neuronal cell body	0.0187927	TGFB1, DAB2IP, VTI1A, HIP1R, RARA, MUL1, RAB8A, SHANK2, BMPR1A
	**GO Term Biological Process**	GO:0007050	cell cycle arrest	0.01999412	STK11, PRKAA1, TGFB1, MLST8, LAMTOR1, UHMK1
	**KEGG Pathway**	hsa04670	Leukocyte transendothelial migration	0.02021782	CLDN6, NCF2, ESAM, PIK3CB, F11R, BCAR1

	**GO Term Cellular Component**	GO:0031093	platelet alpha granule lumen	0.02022904	CFD, TGFB1, PROS1, F5
	**GO Term Biological Process**	GO:0030198	extracellular matrix organization	0.02158625	LAMA5, PRDX4, COL16A1, ITGA2, SPP1, SOX9, F11R
	**GO Term Biological Process**	GO:0097049	motor neuron apoptotic process	0.02191357	FAS, FADD
	**GO Term Biological Process**	GO:0046967	cytosol to ER transport	0.02191357	TAP2, TAP1
	**GO Term Biological Process**	GO:0030852	regulation of granulocyte differentiation	0.02191357	RBP1, RARA
	**KEGG Pathway**	hsa00450	Selenocompound metabolism	0.02198991	TXNRD3, MTR, PAPSS1
	**GO Term Biological Process**	GO:1900182	positive regulation of protein localization to nucleus	0.02209687	STK11, SRC, GLIS2
	**GO Term Cellular Component**	GO:0031902	late endosome membrane	0.0220981	VPS33B, VTI1A, LAMTOR1, SLC29A3, ARL8B
	**GO Term Cellular Component**	GO:0030136	clathrin-coated vesicle	0.02325584	VPS33B, VTI1A, HIP1R, VAMP2
	**GO Term Biological Process**	GO:0007173	epidermal growth factor receptor signaling pathway	0.02380344	TGFB1, SRC, SOX9, BCAR1

	**GO Term Biological Process**	GO:0045453	bone resorption	0.0241329	SRC, TRAF6, HNF1A
	**GO Term Biological Process**	GO:0010842	retina layer formation	0.0241329	PROM1, TOPORS, HIPK2
	**GO Term Biological Process**	GO:0032091	negative regulation of protein binding	0.02492729	STUB1, ZFPM1, NES, DKK1
	**GO Term Molecular Function**	GO:0008289	lipid binding	0.02614999	PPP5C, RBP1, PACSIN2, APOA1, AP2M1, SH3GL1
	**GO Term Cellular Component**	GO:0005615	extracellular space	0.02673206	CFD, LGALS3BP, LAMA5, HSPA8, CPB2, TGFB1, SERPINE2, EPO, PROS1, HP, APOA1, TFPI, MERTK, DKK1, AGT, F5, SDCBP, LGALS3, PRDX4, AXL, SPP1, STOM, PROM1
	**GO Term Biological Process**	GO:0006366	transcription from RNA polymerase II promoter	0.0269782	NR5A1, CEBPA, PLSCR1, ONECUT1, PAX6, SOX9, HNF1A, ZBTB7B, ATF5, ETV4, SOX4, GLIS2
	**KEGG Pathway**	hsa04350	TGF-beta signaling pathway	0.02751185	PPP2CB, TGFB1, ID1, RPS6KB2, BMPR1A

	**GO Term Cellular Component**	GO:0000922	spindle pole	0.02824185	PPP2CB, NEDD9, PKP4, RMDN1, TOPORS
	**GO Term Biological Process**	GO:0030511	positive regulation of transforming growth factor beta receptor signaling pathway	0.02842416	SDCBP, STK11, HIPK2
	**GO Term Biological Process**	GO:0045742	positive regulation of epidermal growth factor receptor signaling pathway	0.02842416	GPER1, HIP1R, AGT
	**GO Term Biological Process**	GO:0070371	ERK1 and ERK2 cascade	0.02842416	APOA1, SOX9, AGT
	**GO Term Biological Process**	GO:0048041	focal adhesion assembly	0.02842416	LAMA5, ITGA2, SORBS1
	**GO Term Biological Process**	GO:0050679	positive regulation of epithelial cell proliferation	0.0284679	TGFB1, ID1, SOX9, BMPR1A
	**KEGG Pathway**	hsa05222	Small cell lung cancer	0.02857709	LAMA5, TRAF3, TRAF6, ITGA2, PIK3CB
	**GO Term Biological Process**	GO:0043066	negative regulation of apoptotic process	0.02953355	PRKAA1, SRC, TRAF6, ID1, RARA, FAS, SPRY2, SOX9, RAF1, ATF5, TOPORS

	**GO Term Molecular Function**	GO:0004842	ubiquitin-protein transferase activity	0.02965677	HERC1, TRAF3, TRAF6, FBXO3, TRIM25, MUL1, STUB1, PAX6, TOPORS
	**GO Term Biological Process**	GO:0001933	negative regulation of protein phosphorylation	0.02970431	PPP5C, TGFB1, DAB2IP, PAX6
	**GO Term Biological Process**	GO:2001235	positive regulation of apoptotic signaling pathway	0.03067571	TRIM39, DAB2IP, NKX3-1
	**GO Term Biological Process**	GO:0046580	negative regulation of Ras protein signal transduction	0.03067571	PPP2CB, DAB2IP, SPRY2
	**KEGG Pathway**	hsa04064	NF-kappa B signaling pathway	0.0307807	TRAF3, TRAF6, TRIM25, TAB2, TNFRSF11A
	**KEGG Pathway**	hsa04012	ErbB signaling pathway	0.0307807	SRC, RPS6KB2, PIK3CB, RAF1, CAMK2G
	**GO Term Cellular Component**	GO:0005899	insulin receptor complex	0.03127655	IRS1, SORBS1
	**GO Term Cellular Component**	GO:0042825	TAP complex	0.03127655	TAP2, TAP1
	**GO Term Cellular Component**	GO:0070033	synaptobrevin 2-SNAP-25-syntaxin-1a-complexin II complex	0.03127655	STX1A, VAMP2

	**GO Term Molecular Function**	GO:0042803	protein homodimerization activity	0.03142267	CEBPA, PDXK, TGFB1, TESC, DAB2IP, HIP1R, TAP1, HNF1A, SDCBP, PRDX4, ID1, STOM, STUB1, CAMK2G, BMPR1A
	**GO Term Biological Process**	GO:0030154	cell differentiation	0.03229511	NR5A1, LAMA5, SLC7A5, TXNRD3, ONECUT1, ZBTB7B, RAF1, CAMK2G, ETV4, ETS2, PRRC2A
	**GO Term Biological Process**	GO:0003331	positive regulation of extracellular matrix constituent secretion	0.03269058	CPB2, AGT
	**GO Term Biological Process**	GO:0048858	cell projection morphogenesis	0.03269058	PACSIN2, NES
	**GO Term Biological Process**	GO:0051250	negative regulation of lymphocyte activation	0.03269058	AXL, MERTK
	**GO Term Molecular Function**	GO:0015440	peptide-transporting ATPase activity	0.03269396	TAP2, TAP1
	**GO Term Molecular Function**	GO:0023029	MHC class Ib protein binding	0.03269396	TAP2, TAP1

	**GO Term Molecular Function**	GO:0015433	peptide antigen-transporting ATPase activity	0.03269396	TAP2, TAP1
	**GO Term Molecular Function**	GO:0015197	peptide transporter activity	0.03269396	TAP2, TAP1
	**GO Term Biological Process**	GO:2001238	positive regulation of extrinsic apoptotic signaling pathway	0.03299547	GPER1, FADD, AGT
	**GO Term Biological Process**	GO:0006904	vesicle docking involved in exocytosis	0.03299547	VPS33B, EXOC4, RAB8A
	**KEGG Pathway**	hsa04380	Osteoclast differentiation	0.03312089	TGFB1, NCF2, TRAF6, TAB2, TNFRSF11A, PIK3CB
	**KEGG Pathway**	hsa05152	Tuberculosis	0.03369677	TGFB1, SRC, TRAF6, PIK3C3, FADD, RAF1, CAMK2G
	**GO Term Biological Process**	GO:0043087	regulation of GTPase activity	0.03492925	DAB2IP, MLST8, RICTOR, NGEF
	**GO Term Biological Process**	GO:0014068	positive regulation of phosphatidylinositol 3-kinase signaling	0.03492925	GPER1, SOX9, AGT, NKX3-1
	**GO Term Molecular Function**	GO:0001948	glycoprotein binding	0.03494009	SDCBP, TGFB1, STX1A, BMPR1A
	**KEGG Pathway**	hsa05160	Hepatitis C	0.03502566	CLDN6, PPP2CB, TRAF3, TRAF6, PIK3CB, RAF1

	**KEGG Pathway**	hsa05162	Measles	0.03502566	HSPA8, TRAF6, FAS, TAB2, PIK3CB, CD46
	**GO Term Biological Process**	GO:0032008	positive regulation of TOR signaling	0.03538169	MLST8, RICTOR, LAMTOR1
	**GO Term Cellular Component**	GO:0016020	membrane	0.0361892	CYFIP2, LGALS3BP, DGKA, PANX1, SDCBP, LGALS3, STK11, HERC1, FLOT1, STOM, PIP4K2C, CAMK2G, HSPA8, TOR1A, TAP2, RRAS2, TAP1, MUL1, F5, PRRC2A, SLC7A5, PLSCR1, AGO2, CD7, EXOC4, SFXN3, SPRY2, FAS, PIK3C3, PFN1, VAMP2, NGEF, ARL8B

	**GO Term Cellular Component**	GO:0005634	nucleus	0.03620605	CYFIP2, GLDC, IRS1, GFI1, NAB2, ONECUT1, PIK3CB, ZBTB22, ETS2, LGALS3, STK11, NUDCD1, SOX18, GPER1, SUFU, SOX9, CIC, SOX4, RAB8A, NKX3-1, BCAS3, TESC, ITGA2, APOA1, PAX6, HNF1A, ETV4, TOPORS, UHMK1, NR5A1, PLSCR1, TRAF6, RARA, RAF1, ATF5, PFN1, ZFPM1, SGK2, CEBPA, LAMA5, PRKAA1, PDXK, SRC, NEDD9, MAT2B, BPHL, GLIS2, REPIN1, PPP2CB, DSCR3, SDCBP, PRDX4, ZBTB7B, HSPA8, TGFB1, TXNRD3, ZBTB14, RANBP9, SORBS1, HIPK2, PNRC2, PRRC2A, PPP5C, AGO2, RPS6KB2, ID1, SPRY2, FAS, PTPN2, MSRB1

	**GO Term Molecular Function**	GO:0042605	peptide antigen binding	0.03784053	SLC7A5, FCGRT, TAP1
	**KEGG Pathway**	hsa05168	Herpes simplex infection	0.03870393	TRAF3, TRAF6, TAP2, FAS, TAP1, TAB2, FADD
	**GO Term Molecular Function**	GO:0005524	ATP binding	0.03940311	PDXK, PRKAA1, SRC, DGKA, PIK3CB, HSPA13, GPHN, PAPSS1, STK11, PIP4K2C, CAMK2G, HSPA8, TOR1A, TAP2, TAP1, MERTK, HIPK2, UHMK1, PPP5C, AXL, RPS6KB2, PIK3C3, RAF1, SGK2, BMPR1A
	**GO Term Biological Process**	GO:0006461	protein complex assembly	0.03943268	TRAF6, RANBP9, FAS, SOX9, VAMP2
	**GO Term Cellular Component**	GO:0005770	late endosome	0.04019831	HSPA8, UVRAG, SRC, VPS33B, PIK3C3
	**GO Term Molecular Function**	GO:0005164	tumor necrosis factor receptor binding	0.04035499	TRAF3, TRAF6, FADD

	**GO Term Cellular Component**	GO:0070044	synaptobrevin 2-SNAP-25-syntaxin-1a complex	0.04148427	STX1A, VAMP2
	**GO Term Cellular Component**	GO:0070032	synaptobrevin 2-SNAP-25-syntaxin-1a-complexin I complex	0.04148427	STX1A, VAMP2
	**GO Term Cellular Component**	GO:0005622	intracellular	0.04161022	BCAR3, HSPA8, PRKAA1, DGKA, RRAS2, MTR, MAT2B, PIK3CB, TOM1L1, SDCBP, TRIM39, AXL, RAB23, GPER1, GOLGA1, PIK3C3, OSTF1, RAF1, SGK2, STX1A, ARL8B, NKX3-1
	**KEGG Pathway**	hsa04066	HIF-1 signaling pathway	0.04191534	EPO, RPS6KB2, PIK3CB, CAMK2G, EIF4E
	**GO Term Biological Process**	GO:0098869	cellular oxidant detoxification	0.04208168	PRDX4, TXNRD3, HP, GPX7
	**GO Term Biological Process**	GO:0033138	positive regulation of peptidyl-serine phosphorylation	0.04208168	BCAR3, TGFB1, SPRY2, RAF1
	**GO Term Biological Process**	GO:0031647	regulation of protein stability	0.04208168	HSPA8, MUL1, STUB1, SOX4

	**GO Term Molecular Function**	GO:0001618	virus receptor activity	0.04209446	AXL, ITGA2, F11R, CD46
	**GO Term Biological Process**	GO:0034504	protein localization to nucleus	0.04292196	TOR1A, SOX9, TOPORS
	**GO Term Biological Process**	GO:0051387	negative regulation of neurotrophin TRK receptor signaling pathway	0.04334948	SPRY2, AGT
	**GO Term Biological Process**	GO:0050882	voluntary musculoskeletal movement	0.04334948	VTI1A, HIPK2
	**GO Term Biological Process**	GO:1901203	positive regulation of extracellular matrix assembly	0.04334948	TGFB1, SOX9
	**GO Term Biological Process**	GO:0042992	negative regulation of transcription factor import into nucleus	0.04334948	RAB23, SUFU
	**GO Term Biological Process**	GO:0071677	positive regulation of mononuclear cell migration	0.04334948	LGALS3, TGFB1
	**GO Term Biological Process**	GO:0003170	heart valve development	0.04334948	ZBTB14, SOX9
	**GO Term Biological Process**	GO:0060534	trachea cartilage development	0.04334948	RARA, SOX9
	**GO Term Biological Process**	GO:0030858	positive regulation of epithelial cell differentiation	0.04334948	PAX6, SOX9

	**GO Term Molecular Function**	GO:0046978	TAP1 binding	0.04335392	TAP2, TAP1
	**GO Term Biological Process**	GO:0071347	cellular response to interleukin-1	0.04359383	DAB2IP, SOX9, TFPI, NKX3-1
	**GO Term Cellular Component**	GO:0030659	cytoplasmic vesicle membrane	0.04437487	TOR1A, GPER1, PACSIN2, ZDHHC17, RAB8A
	**GO Term Biological Process**	GO:0010951	negative regulation of endopeptidase activity	0.0448821	ITIH5, SERPINE2, PROS1, TFPI, AGT
	**GO Term Biological Process**	GO:0043507	positive regulation of JUN kinase activity	0.04555701	TRAF6, DAB2IP, TNFRSF11A
	**GO Term Biological Process**	GO:0043552	positive regulation of phosphatidylinositol 3-kinase activity	0.04555701	TGFB1, IRS1, SRC
	**GO Term Molecular Function**	GO:0005158	insulin receptor binding	0.04556637	IRS1, SRC, SORBS1
	**KEGG Pathway**	hsa04915	Estrogen signaling pathway	0.04607262	HSPA8, SRC, GPER1, PIK3CB, RAF1
	**KEGG Pathway**	hsa04144	Endocytosis	0.04616609	HSPA8, SRC, TRAF6, ARPC5L, CHMP7, AP2M1, RAB8A, SH3GL1
	**GO Term Cellular Component**	GO:0090575	RNA polymerase II transcription factor complex	0.04709657	NR5A1, CEBPA, HIPK2

	**GO Term Cellular Component**	GO:0030496	midbody	0.04766121	UVRAG, PKP4, PIK3C3, TOPORS, ARL8B
	**KEGG Pathway**	hsa05161	Hepatitis B	0.04787304	TGFB1, SRC, FAS, FADD, PIK3CB, RAF1
	**GO Term Biological Process**	GO:0007032	endosome organization	0.04825018	VPS33B, PIK3C3, LAMTOR1
	**GO Term Biological Process**	GO:0001889	liver development	0.048291	CEBPA, ONECUT1, RARA, HNF1A
	**GO Term Biological Process**	GO:0030334	regulation of cell migration	0.048291	LAMA5, TGFB1, SERPINE2, PAX6
	**GO Term Biological Process**	GO:0042384	cilium assembly	0.04834418	LAMA5, RAB23, ONECUT1, CEP83, RAB8A
	**KEGG Pathway**	hsa04950	Maturity onset diabetes of the young	0.0485564	ONECUT1, PAX6, HNF1A
	**GO Term Cellular Component**	GO:0000932	cytoplasmic mRNA processing body	0.04930052	AGO2, EIF4E, TNRC6A, PNRC2
	**GO Term Biological Process**	GO:0006915	apoptotic process	0.04959696	CYFIP2, PLSCR1, RTKN, TRIM39, ADAM15, TRAF3, EPO, HIP1R, FAS, MUL1, FADD, RAF1

	**GO Term Cellular Component**	GO:0005875	microtubule associated complex	0.04970148	CLIP2, DNAAF2, RANBP9
**3**	**GO Term Cellular Component**	GO:0016021	integral component of membrane	1.25E-04	SFT2D3, MOGAT3, XYLT2, ZDHHC2, LCLAT1, FADS3, FADS2, C1GALT1C1, C14ORF1, ACP2, SLC37A4, SLC37A3, SEMA6B, RNF43, ABCG8, JAGN1, AREL1, PAQR7, IL11RA, PLLP, APLP1, ACSL4, LIFR, ELOVL6, TEX2, TGFBR3, TMEM37, SYPL1, CLDN15, PITPNM2, SLC27A4, RNFT2, TMEM161A, LRP4, LCAT, HMGCR, GDPD5, SLC5A3, ZNF7, SYNGR2, UCP2, MARVELD3, FZD3, SLC12A5, FZD4, EPHX1, ATP2B4, YIF1A, NCEH1, LPAR6, BAMBI, LGR4

	**GO Term Biological Process**	GO:0007584	response to nutrient	5.37E-04	ABCG8, SERPINC1, ACSL4, HMGCR, SLC37A4, SLC27A4
	**GO Term Cellular Component**	GO:0005789	endoplasmic reticulum membrane	5.56E-04	RNF43, JAGN1, MOGAT3, EPHX1, XYLT2, ACSL4, ELOVL6, HMGCR, LCLAT1, YIF1A, FADS3, FADS2, NCEH1, C14ORF1, RAB18, RAB3GAP2, SDF2, SLC37A4, SLC27A4
	**KEGG Pathway**	hsa01212	Fatty acid metabolism	0.00110203	FADS2, ACSL4, ELOVL6, ACADM, ACACA
	**GO Term Cellular Component**	GO:0005887	integral component of plasma membrane	0.0013585	SEMA6B, RNF43, SLC12A5, FZD4, IL11RA, ATP2B4, LIFR, ZDHHC2, SLC5A3, TGFBR3, FADS2, APOM, SYPL1, MMP15, LPAR6, PALM, PLXNB1, LGR4
	**GO Term Biological Process**	GO:0006629	lipid metabolic process	0.00179596	PLA2G15, FADS3, FADS2, LCAT, ACSL4, GDPD5, SLC27A4

	**KEGG Pathway**	hsa03320	PPAR signaling pathway	0.0025009	FADS2, NR1H3, ACSL4, ACADM, SLC27A4
	**GO Term Biological Process**	GO:0043087	regulation of GTPase activity	0.00263199	SYDE1, RAB3GAP2, PLXNB1, IQGAP2, RAP1GAP
	**GO Term Biological Process**	GO:0042632	cholesterol homeostasis	0.00282656	ABCG8, APOM, LCAT, NR1H3, SLC37A4
	**GO Term Biological Process**	GO:0008152	metabolic process	0.00335912	NCEH1, PITPNM2, GSTA1, IDS, ACSL4, LCLAT1, SGSH
	**GO Term Biological Process**	GO:0046855	inositol phosphate dephosphorylation	0.00437766	IMPA1, INPP1, INPP5K
	**GO Term Biological Process**	GO:0006636	unsaturated fatty acid biosynthetic process	0.00543151	FADS3, FADS2, ELOVL6
	**GO Term Molecular Function**	GO:0008374	O-acyltransferase activity	0.00545666	PLA2G15, LCAT, LCLAT1
	**GO Term Molecular Function**	GO:0008289	lipid binding	0.00664714	STARD4, PITPNM2, STARD10, FNBP1L, TEX2, EPN1
	**GO Term Cellular Component**	GO:0030424	axon	0.0066958	FZD3, PALM, RNF6, ACADM, GDPD5, RAP1GAP, NRGN
	**KEGG Pathway**	hsa00564	Glycerophospholipid metabolism	0.00822832	PLA2G15, PLA2G12B, DGKQ, LCAT, LCLAT1

	**GO Term Molecular Function**	GO:0017127	cholesterol transporter activity	0.00924924	ABCG8, STARD4, STARD5
	**KEGG Pathway**	hsa04976	Bile secretion	0.01088936	ABCG8, NCEH1, EPHX1, HMGCR
	**GO Term Biological Process**	GO:0006805	xenobiotic metabolic process	0.01805956	NCEH1, ACY1, EPHX1, SULT1A2
	**KEGG Pathway**	hsa01100	Metabolic pathways	0.01812926	PLA2G12B, MOGAT3, ACY1, INPP1, XYLT2, PIPOX, ACSL4, HMGCR, LCLAT1, ACACA, SGSH, MINPP1, C1GALT1C1, IMPA1, DGKQ, PGP, INPP5K, IDS, ACADM
	**GO Term Biological Process**	GO:0006810	transport	0.01844254	AP3M2, SLC12A5, SYPL1, PITPNM2, UCP2, SLC37A4, SLC5A3, SLC27A4

	**GO Term Cellular Component**	GO:0005886	plasma membrane	0.02187989	PHLPP1, SERPINC1, IDE, FNBP1L, ZDHHC2, SLC5A3, ADD1, PALM, FCHO2, INPP5K, MESDC2, RNF43, ABCG8, FZD3, PAQR7, SLC12A5, FZD4, IL11RA, ARRDC1, APLP1, ATP2B4, LIFR, RABGGTB, EPN1, RAP2C, CLDN15, BAMBI, MMP15, LPAR6, DGKQ, RAB18, RAB3GAP2, PLXNB1, LGR4, SLC27A4
	**KEGG Pathway**	hsa00562	Inositol phosphate metabolism	0.02754626	MINPP1, IMPA1, INPP1, INPP5K
	**GO Term Cellular Component**	GO:0043025	neuronal cell body	0.02892627	FZD3, SLC12A5, LRP4, ACSL4, GDPD5, RAP1GAP, NRGN
	**GO Term Molecular Function**	GO:0030165	PDZ domain binding	0.03085817	TGFBR3, FZD3, FZD4, ATP2B4
	**KEGG Pathway**	hsa04975	Fat digestion and absorption	0.03459033	ABCG8, PLA2G12B, MOGAT3

	**GO Term Biological Process**	GO:0001942	hair follicle development	0.0348561	FZD3, LRP4, LGR4
	**KEGG Pathway**	hsa04142	Lysosome	0.03538716	AP3M2, PLA2G15, IDS, ACP2, SGSH
	**GO Term Biological Process**	GO:0006897	endocytosis	0.03551937	SNX18, APLP1, LRP4, FNBP1L, EPN1
	**GO Term Molecular Function**	GO:0005096	GTPase activator activity	0.03839216	TIAM2, SYDE1, SH3BP1, RASA2, RAB3GAP2, PLXNB1, RAP1GAP
	**GO Term Cellular Component**	GO:0070062	extracellular exosome	0.0403175	LAD1, ACY1, SERPINC1, SERPINA10, ERV3-1, LCAT, FNBP1L, IQGAP2, CPN2, ACACA, SGSH, C1GALT1C1, APOM, SYNGR2, IMPA1, ACP2, ACADM, FZD4, PLLP, ARRDC1, GPX3, ACSL4, LIFR, TGFBR3, RAP2C, PLA2G15, TIAM2, MINPP1, SNX18, SYPL1, GSTA1, RAB18, NIT1

	**GO Term Cellular Component**	GO:0043235	receptor complex	0.04421737	TGFBR3, ABCG8, NR1H3, LIFR
	**GO Term Molecular Function**	GO:0061513	glucose 6-phosphate:inorganic phosphate antiporter activity	0.04478983	SLC37A4, SLC37A3
	**GO Term Biological Process**	GO:0016311	dephosphorylation	0.0452492	MINPP1, PGP, INPP5K, ACP2
	**GO Term Molecular Function**	GO:0008270	zinc ion binding	0.04677881	RNFT2, ZNF331, RNF43, ANKIB1, CHURC1, ACY1, NR1H3, RNF6, CRIP3, IDE, RABGGTB, ZDHHC2, CPN1, ZNF92, MMP15, LONRF3, HIF1AN

**4**	**KEGG Pathway**	hsa01100	Metabolic pathways	6.09E-10	PRPS2, LIPT1, FH, SGMS1, PIGO, GPAA1, HEXB, AKR1D1, MPI, DLST, PYGL, ENO3, GMPPB, UGP2, HSD17B2, PGM2, MAN1A1, MECR, CHPF2, CSGALNACT2, MOGS, DOLK, ALG11, HOGA1, ALG1, PRODH2, GANC, AGMAT, COQ5, RPIA, PIGC, PIGB, NDUFS7, NMNAT1, OGDH, ALDOC, DEGS1, B4GALT7, CERS1
	**GO Term Biological Process**	GO:0006506	GPI anchor biosynthetic process	2.80E-05	PIGC, PIGB, PYURF, PIGO, PIGZ, MPPE1

	**GO Term Cellular Component**	GO:0016021	integral component of membrane	3.28E-05	PIGO, OGFOD3, PIGZ, TMEM181, PKD2, NAGPA, TMEM261, SYNE2, MAN1A1, ABCD1, SMIM12, SLC39A4, ARSA, SMIM15, CHST7, CHPF2, CSGALNACT2, MOGS, HOGA1, MANEAL, ALG11, ALG1, POMGNT1, PIGC, PIGB, DNAJC30, RFT1, TMEM126A, CRELD1, L2HGDH, MPPE1, B4GALT7, CERS1, SLC26A2, TP53I13, LMAN2L, SGMS1, CHRNA5, HEPACAM, HPN, OMA1, TMTC4, HSD17B2, CLPTM1, CHST13, EMC6, MEST, GPR157, CMTM7, NUS1, OPN3, PEX19, SRD5A3, TMEM132A, ERLIN1, TMEM62, MARCH9, DOLK, AIG1, SLC8B1, TMEM60, C12ORF76, RPIA, CDAN1, TSPAN13, REEP4, DEGS1, CNIH4, SSBP4, TECPR1

	**GO Term Cellular Component**	GO:0005789	endoplasmic reticulum membrane	5.82E-05	ABHD4, LMAN2L, PIGO, GPAA1, HPN, PIGZ, PKD2, UFL1, HSD17B2, MEST, NUS1, SEC24B, SRD5A3, TMEM132A, ERLIN1, MOGS, DOLK, ALG11, ALG1, PIGC, PYURF, PIGB, REEP4, RFT1, DEGS1, CERS1
	**KEGG Pathway**	hsa00532	Glycosaminoglycan biosynthesis - chondroitin sulfate / dermatan sulfate	1.21E-04	CHST7, CHPF2, CSGALNACT2, CHST13, B4GALT7
	**KEGG Pathway**	hsa00563	Glycosylphosphatidylinositol(GPI)-anchor biosynthesis	3.94E-04	PIGC, PIGB, PIGO, GPAA1, PIGZ
	**GO Term Molecular Function**	GO:0016787	hydrolase activity	6.43E-04	ABHD11, ABHD4, ABHD8, TMEM62, MPPE1, ABHD5, MEST, GLA, CTSC, CES2
	**GO Term Biological Process**	GO:0097502	mannosylation	7.23E-04	PIGB, PIGZ, ALG1, ALG11
	**KEGG Pathway**	hsa00600	Sphingolipid metabolism	0.00233606	ARSA, SGMS1, DEGS1, GLA, CERS1

	**GO Term Cellular Component**	GO:0005783	endoplasmic reticulum	0.00246943	CEMIP, SAR1A, SGMS1, TMEM132A, SRD5A3, EDEM2, ERLIN1, PIGZ, MOGS, ALG1, PKD2, UFL1, TRAPPC6A, REEP4, DEGS1, MAN1A1, MPPE1, MEST, CNIH4, CES2, CERS1
	**GO Term Biological Process**	GO:0030206	chondroitin sulfate biosynthetic process	0.00250355	CHST7, CHPF2, CSGALNACT2, CHST13
	**GO Term Cellular Component**	GO:0005739	mitochondrion	0.00251695	MIPEP, LIPT1, FH, MRPS36, DLST, SYNE2, GMPPB, ABHD11, HARS, ABCD1, MECR, APOOL, HOGA1, SYNJ2BP, PRODH2, NIPSNAP1, AGMAT, COQ5, COQ4, ARMC1, PYURF, OXLD1, DNAJC30, RPUSD3, TMEM126A, OGDH, PTRH1, ALDOC, TPP1, DEGS1, L2HGDH, AARS

	**GO Term Biological Process**	GO:0005975	carbohydrate metabolic process	0.00270925	HEXB, MPI, PGM2, PYGL, NAGPA, B4GALT7, GLA, GANC
	**GO Term Cellular Component**	GO:0036064	ciliary basal body	0.00274304	C2CD3, IFT172, POC1A, B9D1, CEP41, BBS7, PKD2
	**KEGG Pathway**	hsa01200	Carbon metabolism	0.00326684	PRPS2, RPIA, FH, OGDH, DLST, ALDOC, ENO3
	**KEGG Pathway**	hsa00500	Starch and sucrose metabolism	0.0035459	UGP2, PGM2, PYGL, GANC
	**KEGG Pathway**	hsa00520	Amino sugar and nucleotide sugar metabolism	0.00369185	GMPPB, UGP2, HEXB, MPI, PGM2
	**GO Term Biological Process**	GO:0001947	heart looping	0.00426957	C2CD3, IFT172, NPHP3, BBS7, PKD2
	**KEGG Pathway**	hsa00510	N-Glycan biosynthesis	0.00435813	MOGS, MAN1A1, DOLK, ALG1, ALG11
	**GO Term Biological Process**	GO:0006489	dolichyl diphosphate biosynthetic process	0.00474908	NUS1, SRD5A3, DOLK
	**GO Term Molecular Function**	GO:0016757	transferase activity, transferring glycosyl groups	0.00520579	PIGB, PIGZ, ALG1, B4GALT7, B3GNTL1
	**KEGG Pathway**	hsa00052	Galactose metabolism	0.00522541	UGP2, PGM2, GLA, GANC

	**KEGG Pathway**	hsa01130	Biosynthesis of antibiotics	0.00547694	PRPS2, RPIA, FH, UGP2, OGDH, PGM2, DLST, ALDOC, ENO3
	**GO Term Biological Process**	GO:0046487	glyoxylate metabolic process	0.00580169	LIPT1, OGDH, DLST, HOGA1
	**KEGG Pathway**	hsa00030	Pentose phosphate pathway	0.00587475	PRPS2, RPIA, PGM2, ALDOC
	**GO Term Cellular Component**	GO:0000139	Golgi membrane	0.00707982	SEC24B, CHST7, LMAN2L, SGMS1, CHPF2, TMEM132A, CSGALNACT2, MARCH9, MANEAL, POMGNT1, TRAPPC6A, MAN1A1, CHST13, B4GALT7, CTSC, CERS1
	**GO Term Biological Process**	GO:0006629	lipid metabolic process	0.0081392	LIPT1, ABHD4, SRD5A3, NPHP3, TPP1, OMA1, ABHD5
	**GO Term Biological Process**	GO:0032259	methylation	0.0137802	SMYD5, PRDM15, METTL6, METTL21B, COQ5
	**GO Term Cellular Component**	GO:0005793	endoplasmic reticulum-Golgi intermediate compartment	0.01561923	LMAN2L, TRAPPC12, MAN1A1, MPPE1, CNIH4

	**GO Term Molecular Function**	GO:0000030	mannosyltransferase activity	0.01619593	PIGB, PIGZ, ALG1
	**GO Term Cellular Component**	GO:0005814	centriole	0.02293206	C2CD3, CEP120, STARD9, POC1A, CEP192, CEP41
	**KEGG Pathway**	hsa04142	Lysosome	0.02391508	ARSA, HEXB, TPP1, NAGPA, GLA, CTSC
	**GO Term Biological Process**	GO:0007031	peroxisome organization	0.02477138	PEX19, PEX10, ABCD1
	**GO Term Biological Process**	GO:0006491	N-glycan processing	0.02779288	EDEM2, MAN1A1, ENGASE
	**GO Term Biological Process**	GO:0006103	2-oxoglutarate metabolic process	0.02779288	MRPS36, OGDH, L2HGDH
	**GO Term Biological Process**	GO:0006486	protein glycosylation	0.03129545	SRD5A3, MPI, ALG1, NAGPA, POMGNT1
	**GO Term Molecular Function**	GO:0008168	methyltransferase activity	0.03526982	SMYD5, PRDM15, METTL6, METTL21B, COQ5
	**GO Term Molecular Function**	GO:0003824	catalytic activity	0.0360155	PIGC, ARSA, FH, ISOC1, MEST, GLA, GANC
	**GO Term Biological Process**	GO:0046513	ceramide biosynthetic process	0.04122621	SGMS1, DEGS1, CERS1

	**GO Term Biological Process**	GO:0006015	5-phosphoribose 1-diphosphate biosynthetic process	0.04570164	PRPS2, PYGL
	**GO Term Biological Process**	GO:0008152	metabolic process	0.04850076	ARSA, UGP2, STARD9, ISOC1, DLST, HOGA1

5	**GO Term Cellular Component**	GO:0005654	nucleoplasm	4.84E-21	RB1, MUS81, UBE2D1, CWC25, BRCA2, UBE2Z, EME1, SUMO2, KPNA4, PIP4K2B, TIPIN, H2AFX, RECQL, ESCO2, CDC25C, RAD23B, CLPX, RBX1, RBL2, RBL1, DDX39A, TFDP1, MSH2, SNRPA1, SNRNP25, GEMIN7, DTL, HIST1H2BD, RHNO1, HDAC5, ANAPC15, UHRF2, NOLC1, GPS2, PKMYT1, HDAC8, CENPA, ATXN3, CCNB1, PPP4C, RBBP4, TAF1B, HLTF, E2F2, E2F3, MCL1, MAP2K6, GINS1, CDT1, PPIL2, SETD1B, CBX2, XRCC3, RPA1, HMGA2, DHFR, POLA2, MYO1C, PHF5A, CENPH, CENPI, PRC1, PRKRA, UBE2T, KIF4A, COPS2, CDK12, NUP37

	**GO Term Biological Process**	GO:0051726	regulation of cell cycle	3.88E-10	RB1, UHRF2, CCNF, PKMYT1, CDC25C, RBL2, CCNB1, RBL1, RBBP4, UBA3, E2F2, CDK12, DTL

	**GO Term Cellular Component**	GO:0005634	nucleus	4.60E-10	RB1, MUS81, CCNJ, CCNF, BRCA2, MKI67, CHAF1A, EME1, SUMO2, NEK2, ENOPH1, TRIM23, TIPIN, H2AFX, RECQL, ESCO2, CDC25C, RAD23B, RBL2, RBL1, DDX39A, TFDP1, ESPL1, KIFC1, SNRNP25, GEMIN7, UBE2V1, UMPS, DTL, HIST1H2BD, RHNO1, HDAC5, RNASEH2C, UHRF2, FOXK1, HDAC8, CENPA, ATXN3, CCNB1, PPP4C, RBBP4, TAF1B, HLTF, RBBP9, E2F3, MCL1, CTC1, GINS1, CDT1, CDKN2C, PPIL2, SETD1B, CBX2, XRCC3, RPA1, HMGA2, APEX2, UBOX5, CENPH, CENPI, PRC1, DNAJA3, UBE2T, TDP1, UBA3, BAX, CDK12, CLASRP, TRIM32, NUP37

	**GO Term Molecular Function**	GO:0005515	protein binding	5.18E-09	RB1, OTUD4, MUS81, MOCS3, CCNF, UBE2D1, BRCA2, MKI67, UBE2Z, TCHP, CHAF1A, EME1, SUMO2, KPNA4, NEK2, PIP4K2B, CEP55, TRIM23, TIPIN, H2AFX, LANCL1, RECQL, CDC25C, RAD23B, CCNB1IP1, CLPX, RBX1, RBL2, RBL1, DDX39A, TFDP1, PSRC1, MSH2, ESPL1, SNRPA1, NUP54, SNRNP25, GEMIN7, UBE2V1, MTFR2, DTL, RHNO1, HDAC5, CDCA3, UHRF2, NOLC1, GPS2, FOXK1, PKMYT1, HDAC8, CENPA, ATXN3, CCNB1, PPP4C, RBBP4, MAT2A, TAF1B, HLTF, E2F2, RBBP9, E2F3, MCL1, MAP2K6, CTC1, CDT1, CDKN2C, PPIL2, SETD1B, SEC16A, CBX2, XRCC3, RPA1, HMGA2, UBE2A, UBOX5, MYO1C, CENPH, CENPI, PRC1, PRKRA, DNAJA3, KIF4A, TDP1, COPS2, UBA3, BAX, NDUFAF1, CDK12, TRIM32, NUP37

	**KEGG Pathway**	hsa04110	Cell cycle	7.27E-08	RB1, RBL2, CCNB1, TFDP1, CDKN2C, RBL1, ESPL1, E2F2, E2F3, PKMYT1, CDC25C, RBX1
	**GO Term Biological Process**	GO:0007049	cell cycle	1.48E-06	RBL2, TIPIN, CHAF1A, RBL1, UHRF2, RBBP4, NOLC1, APEX2, GPS2, E2F2, E2F3, ESCO2
	**GO Term Biological Process**	GO:0007067	mitotic nuclear division	8.42E-06	TIPIN, ANAPC15, CDCA3, CCNF, NOLC1, HMGA2, NEK2, KIF11, PKMYT1, CDC25C, CEP55, NUP37
	**GO Term Molecular Function**	GO:0061630	ubiquitin protein ligase activity	1.47E-05	PPIL2, UHRF2, UBE2T, HLTF, UBE2A, UBE2V1, UBE2Z, TRIM32, CCNB1IP1, RBX1
	**GO Term Biological Process**	GO:0000082	G1/S transition of mitotic cell cycle	2.74E-05	RB1, DHFR, CDT1, POLA2, CDKN2C, RPA1, PKMYT1, IQGAP3

	**GO Term Biological Process**	GO:0006281	DNA repair	3.30E-05	MUS81, CHAF1A, MSH2, EME1, UBE2T, TDP1, XRCC3, RPA1, RECQL, APEX2, UBE2A
	**GO Term Biological Process**	GO:0051301	cell division	4.00E-05	RB1, TIPIN, ANAPC15, CDCA3, CCNF, HMGA2, KIF11, CDC25C, CCNB1, PSRC1, KIFC1, NEK2, NUP37
	**GO Term Biological Process**	GO:0006260	DNA replication	5.98E-05	CDT1, POLA2, TIPIN, CHAF1A, RBBP4, RPA1, CDC25C, DTL, RHNO1
	**GO Term Molecular Function**	GO:0003697	single-stranded DNA binding	7.00E-05	CTC1, MSH2, TDP1, XRCC3, RPA1, BRCA2, RAD23B

	**GO Term Molecular Function**	GO:0005524	ATP binding	1.13E-04	MOCS3, NOLC1, UBE2D1, KIF11, PKMYT1, MKI67, UBE2Z, MAT2A, HLTF, PIP4K2B, NEK2, MAP2K6, XRCC3, RECQL, UBE2A, CLPX, MYO1C, DDX39A, MSH2, KIFC1, DNAJA3, KIF4A, UBE2T, UBA3, CDK12
	**GO Term Biological Process**	GO:0000209	protein polyubiquitination	1.72E-04	UBOX5, PPIL2, HLTF, UBE2D1, UBE2A, UBE2V1, DTL, TRIM32, RBX1
	**GO Term Biological Process**	GO:0006302	double-strand break repair	1.84E-04	MSH2, EME1, TDP1, H2AFX, ESCO2, BRCA2
	**GO Term Biological Process**	GO:0006301	postreplication repair	2.01E-04	MSH2, HLTF, UBE2A, UBE2V1
	**KEGG Pathway**	hsa03440	Homologous recombination	2.48E-04	MUS81, EME1, XRCC3, RPA1, BRCA2
	**GO Term Biological Process**	GO:0031573	intra-S DNA damage checkpoint	2.54E-04	MUS81, TIPIN, MSH2, EME1

	**GO Term Molecular Function**	GO:0031625	ubiquitin protein ligase binding	3.44E-04	RB1, ATXN3, UBOX5, UBE2T, HLTF, SUMO2, UBE2A, UBE2V1, UBE2Z, RBX1
	**GO Term Molecular Function**	GO:0003677	DNA binding	3.51E-04	MUS81, RB1, UHRF2, MKI67, CENPA, EME1, HLTF, E2F2, E2F3, CDT1, TIPIN, CBX2, H2AFX, XRCC3, RECQL, RPA1, HMGA2, APEX2, RBL2, POLA2, PHF5A, TFDP1, MSH2, KIF4A, HIST1H2BD

	**GO Term Cellular Component**	GO:0005737	cytoplasm	3.86E-04	UBE2D1, KIF11, BRCA2, MKI67, UBE2Z, TCHP, EME1, NEK2, TIPIN, CKAP2L, LANCL1, RECQL, RAD23B, CLPX, RBL2, DDX39A, PSRC1, ESPL1, SNRNP25, GEMIN7, UBE2V1, UMPS, DTL, HIST1H2BD, HDAC5, NOLC1, HDAC8, ATXN3, CCNB1, PPP4C, HLTF, RBBP9, E2F3, MCL1, MAP2K6, GINS1, CDKN2C, PPIL2, SETD1B, SEC16A, XRCC3, UBE2A, POLA2, MYO1C, CENPI, PRC1, PRKRA, DNAJA3, UBE2T, KIF4A, TDP1, COPS2, BAX, NDUFAF1, TRIM32
	**KEGG Pathway**	hsa04120	Ubiquitin mediated proteolysis	6.43E-04	UBOX5, PPIL2, UBA3, UBE2D1, UBE2A, UBE2Z, TRIM32, RBX1

	**GO Term Biological Process**	GO:0016032	viral process	6.50E-04	RB1, RBL1, H2AFX, BAX, NUP54, CDC25C, CENPA, TRIM23, RBX1, NUP37
	**GO Term Cellular Component**	GO:0005829	cytosol	7.64E-04	MOCS3, ANAPC15, CDCA3, UBE2D1, KIF11, PKMYT1, HDAC8, CENPA, IQGAP3, ATXN3, CCNB1, MAT2A, KPNA4, PIP4K2B, NEK2, ENOPH1, MCL1, MAP2K6, CDT1, CDKN2C, SEC16A, UBE2A, CDC25C, RBX1, DHFR, MYO1C, PSRC1, ESPL1, CENPH, PRC1, CENPI, PRKRA, DNAJA3, KIF4A, UBA3, BAX, GEMIN7, UBE2V1, UMPS, TRIM32, NUP37
	**GO Term Biological Process**	GO:0043550	regulation of lipid kinase activity	8.19E-04	RB1, RBL2, RBL1

	**GO Term Biological Process**	GO:0036297	interstrand cross-link repair	8.82E-04	MUS81, EME1, UBE2T, XRCC3, RPA1
	**GO Term Molecular Function**	GO:0001047	core promoter binding	0.0010669	RB1, HDAC5, HMGA2, E2F2, E2F3
	**GO Term Biological Process**	GO:0000070	mitotic sister chromatid segregation	0.00116393	ESPL1, KIFC1, NEK2, CENPA
	**GO Term Biological Process**	GO:0007088	regulation of mitotic nuclear division	0.00116393	NEK2, PKMYT1, MKI67, CDC25C
	**GO Term Molecular Function**	GO:0061631	ubiquitin conjugating enzyme activity	0.00190009	UBE2T, UBE2D1, UBE2A, UBE2Z
	**GO Term Biological Process**	GO:0006977	DNA damage response, signal transduction by p53 class mediator resulting in cell cycle arrest	0.0020696	RBL2, CCNB1, TFDP1, BAX, CDC25C
	**GO Term Cellular Component**	GO:0000922	spindle pole	0.00230082	CCNB1, PSRC1, CKAP2L, PRC1, NEK2, KIF11
	**GO Term Molecular Function**	GO:0008821	crossover junction endodeoxyribonuclease activity	0.00270352	MUS81, EME1, XRCC3
	**GO Term Biological Process**	GO:0000722	telomere maintenance via recombination	0.00307046	POLA2, XRCC3, RPA1, BRCA2
	**KEGG Pathway**	hsa03460	Fanconi anemia pathway	0.00307838	MUS81, EME1, UBE2T, RPA1, BRCA2

	**KEGG Pathway**	hsa05203	Viral carcinogenesis	0.00327146	RB1, RBL2, HDAC5, RBL1, DNAJA3, BAX, HDAC8, HIST1H2BD
	**GO Term Biological Process**	GO:0006310	DNA recombination	0.00329586	MUS81, XRCC3, RPA1, RECQL, APEX2
	**GO Term Molecular Function**	GO:0004842	ubiquitin-protein transferase activity	0.00344195	PPIL2, UHRF2, UBE2T, UBE2D1, UBE2A, DTL, TRIM32, TRIM23, RBX1
	**GO Term Biological Process**	GO:0000724	double-strand break repair via homologous recombination	0.00347749	H2AFX, XRCC3, RPA1, RECQL, BRCA2
	**GO Term Biological Process**	GO:0007080	mitotic metaphase plate congression	0.0043043	CCNB1, PSRC1, KIFC1, CEP55
	**GO Term Biological Process**	GO:0042769	DNA damage response, detection of DNA damage	0.0043043	RPA1, HMGA2, DTL, RBX1
	**GO Term Molecular Function**	GO:0016874	ligase activity	0.00458147	UBOX5, PPIL2, UHRF2, HLTF, TRIM32, CCNB1IP1, TRIM23, RBX1
	**GO Term Biological Process**	GO:0034080	CENP-A containing nucleosome assembly	0.00465317	CENPH, RBBP4, CENPI, CENPA

	**GO Term Cellular Component**	GO:0001741	XY body	0.004861	H2AFX, ESCO2, UBE2A
	**GO Term Biological Process**	GO:0006289	nucleotide-excision repair	0.00501867	ATXN3, RPA1, BRCA2, RAD23B
	**GO Term Biological Process**	GO:0009411	response to UV	0.00501867	TIPIN, UBE2A, DTL, TRIM32
	**GO Term Molecular Function**	GO:0008134	transcription factor binding	0.00562557	RB1, HDAC5, TFDP1, RBL1, DNAJA3, HMGA2, E2F2, HDAC8
	**GO Term Molecular Function**	GO:0034450	ubiquitin-ubiquitin ligase activity	0.00572343	UBOX5, PPIL2, RBX1
	**GO Term Cellular Component**	GO:0005876	spindle microtubule	0.00607151	PSRC1, PRC1, KIF4A, KIF11
	**GO Term Cellular Component**	GO:0005694	chromosome	0.00804355	KIF4A, SETD1B, RECQL, DTL, RHNO1
	**GO Term Biological Process**	GO:0006974	cellular response to DNA damage stimulus	0.00822449	CTC1, TIPIN, DDX39A, UBE2T, H2AFX, XRCC3, DTL
	**GO Term Biological Process**	GO:0042787	protein ubiquitination involved in ubiquitin-dependent protein catabolic process	0.00928422	CCNB1, ANAPC15, UBE2D1, UBE2Z, TRIM32, RBX1

	**GO Term Biological Process**	GO:0007062	sister chromatid cohesion	0.0120933	CENPH, CENPI, HDAC8, CENPA, NUP37
	**GO Term Biological Process**	GO:0010629	negative regulation of gene expression	0.01295809	RB1, RBL2, CCNB1, RBL1, IQGAP3
	**GO Term Biological Process**	GO:0016569	covalent chromatin modification	0.01386146	RB1, RBL2, RBL1, CBX2, HLTF
	**GO Term Biological Process**	GO:0000083	regulation of transcription involved in G1/S transition of mitotic cell cycle	0.01423304	RB1, DHFR, CDT1
	**GO Term Molecular Function**	GO:0003684	damaged DNA binding	0.01477205	MSH2, H2AFX, RPA1, RAD23B
	**GO Term Cellular Component**	GO:0005819	spindle	0.01602729	RB1, PSRC1, KIFC1, PRC1, KIF11
	**GO Term Cellular Component**	GO:0000784	nuclear chromosome, telomeric region	0.01602729	CTC1, MSH2, XRCC3, RPA1, BRCA2
	**GO Term Cellular Component**	GO:0000790	nuclear chromatin	0.01608572	TIPIN, CHAF1A, RBBP4, EME1, H2AFX, UBE2A
	**GO Term Molecular Function**	GO:0004518	nuclease activity	0.01609262	MUS81, EME1, APEX2
	**GO Term Biological Process**	GO:0016925	protein sumoylation	0.01680851	CBX2, SUMO2, RPA1, NUP54, NUP37

	**GO Term Biological Process**	GO:0033262	regulation of nuclear cell cycle DNA replication	0.01827161	CDT1, TIPIN
	**GO Term Biological Process**	GO:0051439	regulation of ubiquitin-protein ligase activity involved in mitotic cell cycle	0.01861764	CCNB1, ANAPC15, UBE2D1
	**GO Term Biological Process**	GO:0007131	reciprocal meiotic recombination	0.01861764	MSH2, XRCC3, CCNB1IP1
	**GO Term Biological Process**	GO:0000715	nucleotide-excision repair, DNA damage recognition	0.01861764	COPS2, RAD23B, RBX1
	**GO Term Biological Process**	GO:0007283	spermatogenesis	0.01950385	CCNB1, KIFC1, H2AFX, HMGA2, BRCA2, CDC25C, RAD23B
	**GO Term Biological Process**	GO:0006513	protein monoubiquitination	0.02181598	UBE2T, DTL, RBX1
	**GO Term Biological Process**	GO:0000281	mitotic cytokinesis	0.02181598	KIF4A, CENPA, CEP55
	**GO Term Molecular Function**	GO:0019901	protein kinase binding	0.02240305	CCNB1, CDKN2C, MSH2, PRC1, DNAJA3, KIF11, CDC25C, MAP2K6
	**GO Term Cellular Component**	GO:0030496	midbody	0.02259074	PSRC1, PRC1, KIF4A, NEK2, CEP55

	**KEGG Pathway**	hsa04114	Oocyte meiosis	0.02328026	CCNB1, ESPL1, PKMYT1, CDC25C, RBX1
	**GO Term Molecular Function**	GO:0030544	Hsp70 protein binding	0.02376055	DNAJA3, BAX, HDAC8
	**GO Term Cellular Component**	GO:0033186	CAF-1 complex	0.02639373	CHAF1A, RBBP4
	**GO Term Cellular Component**	GO:0097136	Bcl-2 family protein complex	0.02639373	BAX, MCL1
	**GO Term Biological Process**	GO:0006294	nucleotide-excision repair, preincision complex assembly	0.02883372	RPA1, RAD23B, RBX1
	**GO Term Biological Process**	GO:0043161	proteasome-mediated ubiquitin-dependent protein catabolic process	0.02934733	ATXN3, ANAPC15, UBE2D1, UBE2A, RAD23B, RBX1
	**KEGG Pathway**	hsa05212	Pancreatic cancer	0.03031852	RB1, E2F2, E2F3, BRCA2
	**GO Term Cellular Component**	GO:0000776	kinetochore	0.03201195	CENPH, CENPI, NEK2, NUP37
	**GO Term Cellular Component**	GO:0005667	transcription factor complex	0.03281361	RBL2, TFDP1, RBL1, E2F2, E2F3
	**GO Term Biological Process**	GO:0070911	global genome nucleotide-excision repair	0.03460724	SUMO2, RAD23B, RBX1

	**GO Term Molecular Function**	GO:0051434	BH3 domain binding	0.0350813	BAX, MCL1
	**GO Term Biological Process**	GO:0071922	regulation of cohesin loading	0.03621248	RB1, HDAC8
	**GO Term Biological Process**	GO:0071459	protein localization to chromosome, centromeric region	0.03621248	RB1, CENPA
	**GO Term Biological Process**	GO:0090266	regulation of mitotic cell cycle spindle assembly checkpoint	0.03621248	CCNB1, ANAPC15
	**GO Term Biological Process**	GO:0046602	regulation of mitotic centrosome separation	0.03621248	NEK2, KIF11
	**GO Term Biological Process**	GO:0072429	response to intra-S DNA damage checkpoint signaling	0.03621248	MUS81, EME1
	**GO Term Molecular Function**	GO:0031492	nucleosomal DNA binding	0.0364867	RBBP4, HMGA2, CENPA
	**GO Term Biological Process**	GO:0090307	mitotic spindle assembly	0.0366235	KIFC1, NEK2, KIF11
	**GO Term Molecular Function**	GO:0042802	identical protein binding	0.03699134	RB1, ATXN3, CHAF1A, DDX39A, MAT2A, PRC1, PRKRA, UBA3, BAX, TRIM32, TRIM23

	**GO Term Biological Process**	GO:0006325	chromatin organization	0.038684	HDAC5, HMGA2, HDAC8
	**GO Term Biological Process**	GO:0000079	regulation of cyclin-dependent protein serine/threonine kinase activity	0.038684	CDKN2C, PKMYT1, CDC25C
	**GO Term Cellular Component**	GO:0005730	nucleolus	0.03936401	MUS81, NOLC1, APEX2, MKI67, RBL2, MYO1C, CHAF1A, EME1, HLTF, RBBP9, NEK2, CDK12, DTL
	**GO Term Molecular Function**	GO:0004407	histone deacetylase activity	0.04051009	HDAC5, RBBP4, HDAC8
	**GO Term Biological Process**	GO:0006284	base-excision repair	0.04078777	RPA1, APEX2, HMGA2
	**GO Term Cellular Component**	GO:0000785	chromatin	0.04214706	RB1, HMGA2, ESCO2, UBE2A
	**GO Term Cellular Component**	GO:0031465	Cul4B-RING E3 ubiquitin ligase complex	0.04360491	DTL, RBX1
	**GO Term Cellular Component**	GO:0048476	Holliday junction resolvase complex	0.04360491	MUS81, EME1
	**GO Term Molecular Function**	GO:0016887	ATPase activity	0.04366366	DDX39A, MSH2, KIFC1, HLTF, CLPX

	**GO Term Biological Process**	GO:0031052	chromosome breakage	0.04506076	HMGA2, BRCA2
	**GO Term Biological Process**	GO:0070345	negative regulation of fat cell proliferation	0.04506076	TFDP1, E2F3
	**GO Term Molecular Function**	GO:0051082	unfolded protein binding	0.04722391	CHAF1A, DNAJA3, NDUFAF1, CLPX
**6**	**GO Term Cellular Component**	GO:0005730	nucleolus	2.38E-10	DDX28, INO80B, INO80E, NIP7, RRP1, PPAN, MRI1, RRP8, ZNRD1, ENC1, WBSCR22, RRS1, FAM21A, RPP14, LYAR, EXOSC2, N4BP1, UTP14C, TOE1, NOM1, TRIM41, NIFK, NOP16, METTL1, CCDC137, IMP3, DDX31, AEN, BYSL, RCN2, CDKN2AIPNL, POLR1A, TAOK2, NHP2, CCDC86, POLR3K, RPS23

	**GO Term Molecular Function**	GO:0044822	poly(A) RNA binding	1.00E-07	DDX28, KHDRBS3, DQX1, SF3B6, RTCA, NIP7, EIF4A3, RRP1, PPAN, RPS27L, RRP8, RRP7A, RPS15A, DHX35, WBSCR22, RRS1, IGF2BP3, LYAR, RPS12, APOBEC3C, NOM1, TRMT10C, NIFK, NOP16, CCDC137, IMP3, DDX55, DDX31, MRPL28, DDX10, RPP25L, BYSL, MRPL44, CCDC9, NHP2, CCDC86, RPL27, SRFBP1, RPS23
	**GO Term Biological Process**	GO:0006364	rRNA processing	1.31E-07	IMP3, EIF4A3, RRP1, RPS27L, RRP8, BYSL, RRP7A, RPS15A, CCDC86, RPL27, RPP14, EXOSC2, RPL39, RPS23, UTP14C, RPS12

	**KEGG Pathway**	hsa03010	Ribosome	6.28E-06	RPS15A, MRPL19, MRPS18A, RPS27L, MRPL28, RPL27, MRPL34, RPL39, RPS23, RPS12
	**KEGG Pathway**	hsa03020	RNA polymerase	1.48E-05	ZNRD1, POLR1A, POLR1C, POLR2H, POLR3K, POLR2L
	**GO Term Molecular Function**	GO:0004004	ATP-dependent RNA helicase activity	1.70E-05	DDX28, DDX59, DDX55, DHX35, DQX1, EIF4A3, DDX31, DDX10
	**GO Term Molecular Function**	GO:0001054	RNA polymerase I activity	1.93E-05	ZNRD1, POLR1A, POLR1C, POLR2H, POLR2L
	**GO Term Cellular Component**	GO:0005736	DNA-directed RNA polymerase I complex	2.73E-05	ZNRD1, POLR1A, POLR1C, POLR2H, POLR2L
	**KEGG Pathway**	hsa00240	Pyrimidine metabolism	4.25E-05	POLD3, ZNRD1, POLR1A, POLR1C, CTPS1, POLR2H, POLR3K, POLR2L
	**GO Term Biological Process**	GO:0006412	translation	8.99E-05	MRPL19, MRPS18A, MRPL28, RPS27L, MRPL34, RPS15A, NHP2, GUF1, RPL27, IGF2BP3, RPL39, RPS23, RPS12

	**GO Term Molecular Function**	GO:0003735	structural constituent of ribosome	1.56E-04	RPS15A, IMP3, MRPL19, MRPS18A, RPS27L, MRPL28, RPL27, MRPL34, RPL39, MRPL46, RPS23, RPS12
	**GO Term Molecular Function**	GO:0003899	DNA-directed RNA polymerase activity	1.58E-04	ZNRD1, POLR1A, POLR1C, POLR2H, POLR3K, POLR2L
	**GO Term Biological Process**	GO:0010501	RNA secondary structure unwinding	1.69E-04	DDX28, DDX59, DDX55, EIF4A3, DDX31, DDX10
	**GO Term Molecular Function**	GO:0003723	RNA binding	2.77E-04	NOM1, DDX59, KHDRBS3, NIFK, DDX55, RTCA, NIP7, THUMPD2, DDX31, DDX10, PUS7L, RPS15A, PPP1R8, NHP2, IGF2BP3, RPP14, EXOSC2, RPL39
	**GO Term Cellular Component**	GO:0030687	preribosome, large subunit precursor	4.22E-04	NIP7, RRP1, PPAN, RRS1, CCDC86
	**GO Term Biological Process**	GO:0006400	tRNA modification	4.23E-04	QTRT1, CTU1, METTL1, CDK5RAP1, TRIT1

	**GO Term Biological Process**	GO:0006362	transcription elongation from RNA polymerase I promoter	6.51E-04	ZNRD1, POLR1A, POLR1C, POLR2H, POLR2L
	**GO Term Biological Process**	GO:0006363	termination of RNA polymerase I transcription	7.43E-04	ZNRD1, POLR1A, POLR1C, POLR2H, POLR2L
	**KEGG Pathway**	hsa03008	Ribosome biogenesis in eukaryotes	9.06E-04	RRP7A, IMP3, NHP2, NOP10, RPP25L, UTP14C
	**GO Term Biological Process**	GO:0006383	transcription from RNA polymerase III promoter	9.54E-04	POLR1C, SNAPC4, POLR2H, POLR3K, POLR2L
	**GO Term Biological Process**	GO:0006361	transcription initiation from RNA polymerase I promoter	9.54E-04	ZNRD1, POLR1A, POLR1C, POLR2H, POLR2L
	**GO Term Cellular Component**	GO:0005666	DNA-directed RNA polymerase III complex	0.00206876	POLR1C, POLR2H, POLR3K, POLR2L
	**GO Term Molecular Function**	GO:0001056	RNA polymerase III activity	0.00207999	POLR1C, POLR2H, POLR3K, POLR2L
	**GO Term Cellular Component**	GO:0022627	cytosolic small ribosomal subunit	0.00308435	RPS15A, MRPS18A, RPS27L, RPS23, RPS12
	**GO Term Biological Process**	GO:0045815	positive regulation of gene expression, epigenetic	0.00343619	ZNRD1, POLR1A, POLR1C, POLR2H, POLR2L

	**KEGG Pathway**	hsa00230	Purine metabolism	0.00362044	POLD3, ZNRD1, POLR1A, POLR1C, POLR2H, POLR3K, POLR2L
	**KEGG Pathway**	hsa04623	Cytosolic DNA-sensing pathway	0.005803	POLR1C, POLR2H, POLR3K, POLR2L
	**GO Term Biological Process**	GO:0070125	mitochondrial translational elongation	0.00622269	MRPL19, MRPS18A, MRPL28, MRPL34, MRPL46, MRPL44
	**GO Term Biological Process**	GO:0070126	mitochondrial translational termination	0.0068666	MRPL19, MRPS18A, MRPL28, MRPL34, MRPL46, MRPL44
	**GO Term Cellular Component**	GO:0005840	ribosome	0.00753936	RPS15A, MRPL19, MRPS18A, RPS27L, RPL27, MRPL34, RPS23, MRPL44
	**GO Term Molecular Function**	GO:0003676	nucleic acid binding	0.01022307	TOE1, ZNF473, DDX59, NIFK, DDX55, EIF4A3, AEN, TRIT1, ZNRD1, DHX35, R3HCC1, ZNF514, ZNF777, IGF2BP3, ZNF788, ZNF557, ZNF358, ZNF445, POLR3K, ZNF101, ZNF574
	**GO Term Biological Process**	GO:0045116	protein neddylation	0.01156072	UBE2F, DCUN1D3, RNF7

	**GO Term Cellular Component**	GO:0071203	WASH complex	0.01248247	KIAA0196, FAM21A, WASH2P
	**GO Term Cellular Component**	GO:0005743	mitochondrial inner membrane	0.01522404	NDUFA8, TIMM9, MRPL19, MRPS18A, MRPL28, MRPL34, MRPL46, FOXRED1, ATP5L, MRPL44, MPC1, GUF1, SFXN4

	**GO Term Cellular Component**	GO:0005654	nucleoplasm	0.01904556	GMEB2, RTCA, EIF4A3, RRP8, PIDD1, ZNRD1, ENC1, SNAPC4, RPS12, METTL1, IMP3, AEN, CDYL, MRPL46, ZHX1, DMTF1, POLR1A, KCTD10, POLR1C, NHP2, GEMIN6, ZNF473, KHDRBS3, SF3B6, RPS27L, RNF7, RRP7A, POLD3, EIF1AD, RPS15A, PPP1R8, WBSCR22, RPRD1B, POLR2H, RPP14, EXOSC2, ZBED5, POLR2L, TOE1, TRMT10C, BRF2, NIFK, GINS4, BYSL, CDKN2AIPNL, POLR3K, CENPQ, NOP10, RPS23
	**GO Term Biological Process**	GO:0032481	positive regulation of type I interferon production	0.0190886	POLR1C, POLR2H, POLR3K, POLR2L
	**GO Term Biological Process**	GO:0000184	nuclear-transcribed mRNA catabolic process, nonsense-mediated decay	0.01920502	RPS15A, EIF4A3, RPL27, RPL39, RPS23, RPS12

	**GO Term Cellular Component**	GO:0005739	mitochondrion	0.0198851	DDX28, TIMM9, MRPL34, CISD3, FOXRED1, ATP5L, POLD3, RPS15A, GM2A, MPC1, CA5A, CDK5RAP1, RPP14, C14ORF159, HEMK1, NDUFA8, NDFIP2, TRMT10C, C14ORF119, ATPIF1, CMC1, MRPL28, MRPL46, TRIT1, CTU1, GUF1, PGAM5
	**KEGG Pathway**	hsa03013	RNA transport	0.0212738	ELAC1, EIF4A3, GEMIN6, GEMIN8, RPP14, RPP25L
	**GO Term Biological Process**	GO:0070900	mitochondrial tRNA modification	0.02760283	CDK5RAP1, TRIT1
	**GO Term Biological Process**	GO:0031120	snRNA pseudouridine synthesis	0.02760283	NHP2, NOP10
	**GO Term Cellular Component**	GO:0005762	mitochondrial large ribosomal subunit	0.03199537	MRPL19, MRPL28, MRPL34, MRPL46
	**GO Term Biological Process**	GO:0006413	translational initiation	0.0320004	EIF1AD, RPS15A, RPL27, RPL39, RPS23, RPS12

	**GO Term Cellular Component**	GO:0005634	nucleus	0.03353395	GMEB2, PHF23, RTCA, RRP1, PPAN, RRP8, EEF1B2, PIDD1, ENC1, SNAPC4, ZNF445, NOM1, DDX59, METTL1, IMP3, VBP1, ELAC1, AEN, CDYL, ZHX1, RPP25L, MRPL44, CAMKMT, PHF20L1, QTRT1, RNF126, LATS2, DMTF1, POLR1A, ZNF514, CCDC86, RPL27, GEMIN6, SRFBP1, ZNF557, ZNF358, GEMIN8, DDX28, INO80B, INO80E, KHDRBS3, INO80D, NIP7, MRPL19, RPS27L, RNF7, SAV1, POLD3, EIF1AD, PPP1R8, RRS1, IGF2BP3, ZNF788, RPRD1B, POLR2H, RPP14, LYAR, EXOSC2, POLR2L, ZNF101, UBE2F, APOBEC3C, SPECC1, TOE1, NOP16, GINS4, BYSL, UFM1, SLC7A6OS, PITHD1, TAOK2, ZNF777, NOP10, ZNF574

	**GO Term Cellular Component**	GO:0031463	Cul3-RING ubiquitin ligase complex	0.0337299	KCTD10, KLHL26, ENC1, RNF7
	**GO Term Biological Process**	GO:0006614	SRP-dependent cotranslational protein targeting to membrane	0.03530488	RPS15A, RPL27, RPL39, RPS23, RPS12
	**GO Term Biological Process**	GO:0031146	SCF-dependent proteasomal ubiquitin-dependent protein catabolic process	0.03696371	FBXO4, RNF7, FBXO9
	**GO Term Biological Process**	GO:0031398	positive regulation of protein ubiquitination	0.03901039	NDFIP2, NDFIP1, PHF23, FBXO4
	**GO Term Biological Process**	GO:0045903	positive regulation of translational fidelity	0.04111898	IMP3, CDK5RAP1
	**GO Term Biological Process**	GO:0031118	rRNA pseudouridine synthesis	0.04111898	NHP2, NOP10
	**GO Term Cellular Component**	GO:0031429	box H/ACA snoRNP complex	0.04277136	NHP2, NOP10
	**GO Term Cellular Component**	GO:0072589	box H/ACA scaRNP complex	0.04277136	NHP2, NOP10
	**GO Term Cellular Component**	GO:0090661	box H/ACA telomerase RNP complex	0.04277136	NHP2, NOP10
	**GO Term Molecular Function**	GO:0034513	box H/ACA snoRNA binding	0.04286212	NHP2, NOP10

	**GO Term Biological Process**	GO:0042273	ribosomal large subunit biogenesis	0.04337819	NOP16, NIP7, RRS1

Table S13. Expression data of regulated genes by miR-512-3p. Direct predicted miRNA targets are highlighted in orange.

		M-miR-512-3p (Sorafenib-Control)				Transfection Control (Sorafenib-Control)			
**Ensembl ID**	**Gene name**	**Base mean**	**log_2 (Fold-Change)**	**p-value**	**p-adj**	**Base mean**	**log_2 (Fold-Change)**	**p-value**	**p-adj**
ENSG00000146678	IGFBP1	1875.283	-3.353	4.747E-44	5.510E-41	224.846	0.108	7.449E-01	8.937E-01
ENSG00000134057	CCNB1	377.193	-1.759	6.718E-12	1.033E-09	501.523	-0.140	5.618E-01	7.999E-01
ENSG00000204444	APOM	589.692	-1.476	6.812E-12	1.035E-09	373.720	-0.266	2.229E-01	5.276E-01
ENSG00000250722	SELENOP	3485.870	-1.534	2.239E-11	3.142E-09	1868.120	-0.205	2.675E-01	5.775E-01
ENSG00000104419	NDRG1	501.022	-1.483	5.081E-11	6.553E-09	218.608	-0.188	4.316E-01	7.177E-01
ENSG00000184232	OAF	938.575	-1.365	2.028E-10	2.332E-08	511.316	-0.333	1.378E-01	4.195E-01
ENSG00000113083	LOX	31.725	-3.040	9.653E-10	9.860E-08	2.819	-0.124	6.858E-01	NA
ENSG00000006042	TMEM98	824.962	-1.220	4.029E-09	3.453E-07	772.599	-0.448	1.297E-02	1.142E-01
ENSG00000121060	TRIM25	475.873	-1.293	1.802E-08	1.358E-06	426.720	-0.448	4.481E-02	2.297E-01
ENSG00000149573	MPZL2	589.376	-1.275	1.809E-08	1.358E-06	478.439	-0.066	7.980E-01	9.188E-01
ENSG00000074964	ARHGEF10L	577.055	-1.241	2.236E-08	1.660E-06	319.662	0.117	6.827E-01	8.639E-01
ENSG00000126562	WNK4	41.010	-2.580	2.324E-08	1.703E-06	22.297	-0.945	2.321E-02	NA
ENSG00000107984	DKK1	920.573	-1.559	3.104E-08	2.226E-06	398.448	-0.364	3.832E-01	6.828E-01
ENSG00000114491	UMPS	395.040	-1.325	4.760E-08	3.166E-06	329.663	-0.452	5.766E-02	2.633E-01
ENSG00000102934	PLLP	127.236	-1.679	5.357E-08	3.490E-06	256.481	0.006	9.861E-01	9.945E-01
ENSG00000141401	IMPA2	346.431	-1.387	6.721E-08	4.227E-06	219.268	-0.273	2.963E-01	6.062E-01
ENSG00000108924	HLF	1340.476	-1.014	1.771E-07	1.042E-05	1419.633	-0.436	1.697E-02	1.334E-01
ENSG00000090615	GOLGA3	495.130	-1.206	1.901E-07	1.113E-05	465.573	-0.362	1.046E-01	3.618E-01
ENSG00000116984	MTR	269.922	-1.405	6.060E-07	3.145E-05	254.507	-0.327	1.695E-01	4.641E-01
ENSG00000122884	P4HA1	715.148	-1.314	6.371E-07	3.280E-05	482.906	-0.492	4.476E-02	2.297E-01
ENSG00000056736	IL17RB	289.476	-1.521	8.862E-07	4.403E-05	185.297	-0.078	7.687E-01	9.051E-01
ENSG00000178772	CPN2	419.756	-1.115	9.393E-07	4.630E-05	284.059	-0.079	7.296E-01	8.882E-01

ENSG00000116701	NCF2	28.543	2.333	9.486E-07	4.658E-05	49.715	1.372	3.524E-04	1.085E-02
ENSG00000175643	RMI2	441.714	-1.301	1.035E-06	4.995E-05	382.203	-0.320	1.417E-01	4.260E-01
ENSG00000064393	HIPK2	611.331	-1.218	1.037E-06	4.995E-05	888.612	-0.385	3.538E-02	2.023E-01
ENSG00000108960	MMD	525.717	-1.393	1.167E-06	5.540E-05	365.856	-0.152	5.691E-01	8.042E-01
ENSG00000158715	SLC45A3	82.958	-1.807	1.494E-06	6.835E-05	47.505	-0.004	9.911E-01	9.966E-01
ENSG00000177000	MTHFR	35.803	-2.292	1.542E-06	7.005E-05	29.191	-1.431	4.584E-04	1.336E-02
ENSG00000089057	SLC23A2	353.382	-1.234	1.575E-06	7.130E-05	370.068	-0.233	2.642E-01	5.739E-01
ENSG00000102081	FMR1	417.915	-1.140	2.554E-06	1.080E-04	366.241	-0.449	4.162E-02	2.206E-01
ENSG00000173218	VANGL1	144.859	-1.488	3.454E-06	1.391E-04	113.391	-0.492	1.370E-01	4.180E-01
ENSG00000221303	SNORA79	40.126	-2.225	4.171E-06	1.628E-04	28.241	-0.745	6.275E-02	2.750E-01
ENSG00000121068	TBX2	191.479	-1.406	4.852E-06	1.855E-04	164.226	-0.430	1.297E-01	4.063E-01
ENSG00000100023	PPIL2	152.356	-1.320	4.904E-06	1.869E-04	150.779	-0.424	1.527E-01	4.405E-01
ENSG00000137642	SORL1	345.242	-1.304	5.032E-06	1.906E-04	249.346	0.353	1.886E-01	4.876E-01
ENSG00000164733	CTSB	1849.640	-0.882	5.778E-06	2.151E-04	1635.400	-0.402	3.734E-02	2.076E-01
ENSG00000070214	SLC44A1	635.860	-1.241	7.716E-06	2.799E-04	546.276	-0.237	2.881E-01	5.990E-01
ENSG00000198265	HELZ	371.056	-1.079	8.508E-06	3.051E-04	442.222	-0.118	5.668E-01	8.026E-01
ENSG00000168906	MAT2A	373.792	-1.185	8.907E-06	3.150E-04	311.060	-0.496	8.630E-02	3.250E-01
ENSG00000135744	AGT	4012.069	-0.787	1.439E-05	4.652E-04	3829.611	-0.499	5.436E-03	6.731E-02
ENSG00000186205	MARC1	302.756	-1.218	1.634E-05	5.229E-04	166.574	-0.474	9.734E-02	3.470E-01
ENSG00000086696	HSD17B2	104.029	-1.450	1.664E-05	5.291E-04	61.675	-0.396	2.720E-01	5.822E-01
ENSG00000138413	IDH1	1332.913	-1.158	1.689E-05	5.352E-04	813.866	-0.211	3.471E-01	6.533E-01
ENSG00000143924	EML4	4003.235	-0.955	1.909E-05	5.931E-04	2569.758	0.297	1.579E-01	4.484E-01
ENSG00000188257	PLA2G2A	199.582	-1.255	2.006E-05	6.173E-04	53.757	-0.246	5.367E-01	7.866E-01
ENSG00000172986	GXYLT2	107.158	-1.394	2.069E-05	6.304E-04	135.080	-0.123	6.867E-01	8.666E-01
ENSG00000186918	ZNF395	159.618	-1.438	2.085E-05	6.339E-04	137.031	-0.086	7.722E-01	9.067E-01
ENSG00000178425	NT5DC1	181.171	-1.188	2.201E-05	6.645E-04	230.217	-0.175	4.709E-01	7.462E-01

ENSG00000197142	ACSL5	645.383	-0.969	2.811E-05	8.067E-04	384.797	-0.402	9.739E-02	3.471E-01
ENSG00000179918	SEPHS2	1283.098	-0.931	3.343E-05	9.381E-04	1077.466	-0.466	1.586E-02	1.290E-01
ENSG00000100342	APOL1	178.953	-1.131	4.360E-05	1.172E-03	131.319	-0.402	2.471E-01	5.552E-01
ENSG00000090382	LYZ	1341.213	-0.919	4.491E-05	1.200E-03	756.630	0.061	7.724E-01	9.067E-01
ENSG00000224870	AL391244.1	166.984	-1.192	4.605E-05	1.222E-03	181.659	-0.361	1.867E-01	4.857E-01
ENSG00000003436	TFPI	1603.260	-0.808	4.843E-05	1.278E-03	1417.713	-0.187	2.779E-01	5.882E-01
ENSG00000213903	LTB4R	35.405	-1.885	5.106E-05	1.339E-03	62.003	-0.340	3.313E-01	6.389E-01
ENSG00000179134	SAMD4B	486.669	-1.013	5.545E-05	1.439E-03	508.433	0.035	8.767E-01	9.525E-01
ENSG00000185127	C6orf120	89.506	-1.363	5.675E-05	1.458E-03	83.519	-0.414	2.324E-01	5.377E-01
ENSG00000116017	ARID3A	2584.696	-0.840	6.030E-05	1.528E-03	1769.262	-0.200	3.049E-01	6.153E-01
ENSG00000103018	CYB5B	808.104	-0.822	6.215E-05	1.558E-03	724.567	-0.082	6.585E-01	8.531E-01
ENSG00000168040	FADD	146.112	-1.343	6.976E-05	1.719E-03	155.344	-0.372	1.548E-01	4.433E-01
ENSG00000012504	NR1H4	314.354	-0.978	7.411E-05	1.802E-03	189.810	-0.426	1.095E-01	3.702E-01
ENSG00000143252	SDHC	461.842	-1.217	7.948E-05	1.915E-03	445.350	-0.200	4.328E-01	7.183E-01
ENSG00000134575	ACP2	203.811	-1.236	8.055E-05	1.926E-03	164.139	-0.481	1.290E-01	4.049E-01
ENSG00000116406	EDEM3	89.501	-1.421	8.125E-05	1.935E-03	76.349	-0.146	6.750E-01	8.602E-01
ENSG00000163009	C2orf48	30.293	-1.907	8.179E-05	1.941E-03	29.106	-0.379	3.584E-01	6.640E-01
ENSG00000108384	RAD51C	238.150	-1.108	8.172E-05	1.941E-03	407.619	0.049	8.210E-01	9.280E-01
ENSG00000144580	CNOT9	352.653	-0.916	8.215E-05	1.946E-03	649.761	-0.147	4.493E-01	7.280E-01
ENSG00000112146	FBXO9	377.071	-0.907	8.291E-05	1.957E-03	495.009	-0.422	3.676E-02	2.063E-01
ENSG00000166189	HPS6	68.488	-1.601	8.724E-05	2.036E-03	54.465	-0.062	8.747E-01	9.523E-01
ENSG00000272502	AC104958.2	177.792	-1.210	9.173E-05	2.115E-03	113.477	-0.256	3.714E-01	6.746E-01
ENSG00000102921	N4BP1	247.714	-0.986	9.175E-05	2.115E-03	307.386	-0.399	1.108E-01	3.723E-01
ENSG00000196502	SULT1A1	885.596	-1.006	9.604E-05	2.190E-03	635.202	-0.495	6.131E-02	2.726E-01
ENSG00000069702	TGFBR3	536.259	-0.967	9.656E-05	2.198E-03	238.091	-0.367	2.295E-01	5.350E-01
ENSG00000266412	NCOA4	1640.545	-0.784	1.003E-04	2.266E-03	1237.653	-0.274	1.609E-01	4.513E-01

ENSG00000181035	SLC25A42	163.847	-1.340	1.049E-04	2.345E-03	66.751	0.098	7.856E-01	9.133E-01
ENSG00000101003	GINS1	186.999	-1.094	1.062E-04	2.363E-03	195.926	-0.445	9.680E-02	3.462E-01
ENSG00000085733	CTTN	913.240	-0.825	1.079E-04	2.395E-03	1003.104	-0.110	5.451E-01	7.902E-01
ENSG00000171105	INSR	449.944	-0.962	1.143E-04	2.498E-03	507.265	-0.297	1.461E-01	4.322E-01
ENSG00000068366	ACSL4	1487.430	-1.214	1.197E-04	2.587E-03	944.067	-0.316	1.474E-01	4.340E-01
ENSG00000172943	PHF8	142.384	-1.277	1.207E-04	2.600E-03	161.635	-0.460	8.808E-02	3.291E-01
ENSG00000085871	MGST2	1454.044	-0.813	1.325E-04	2.796E-03	1151.202	-0.309	1.249E-01	3.979E-01
ENSG00000128335	APOL2	151.012	-1.210	1.370E-04	2.867E-03	76.323	0.192	5.698E-01	8.051E-01
ENSG00000159217	IGF2BP1	1043.109	-0.809	1.372E-04	2.867E-03	1015.946	-0.361	4.360E-02	2.269E-01
ENSG00000105514	RAB3D	124.301	-1.168	1.443E-04	2.973E-03	100.800	0.155	6.070E-01	8.269E-01
ENSG00000124422	USP22	1406.505	-0.729	1.490E-04	3.047E-03	1302.343	-0.372	4.872E-02	2.400E-01
ENSG00000151413	NUBPL	304.823	-0.918	1.564E-04	3.186E-03	352.177	-0.293	1.766E-01	4.726E-01
ENSG00000132801	ZSWIM3	35.941	-1.835	1.755E-04	3.512E-03	19.450	-0.377	3.682E-01	NA
ENSG00000276043	UHRF1	48.896	-1.554	1.784E-04	3.548E-03	71.061	-0.147	6.591E-01	8.531E-01
ENSG00000164096	C4orf3	437.068	-1.147	1.839E-04	3.621E-03	400.691	-0.349	1.047E-01	3.618E-01
ENSG00000156968	MPV17L	339.074	-1.055	1.915E-04	3.722E-03	288.845	-0.448	6.298E-02	2.751E-01
ENSG00000182810	DDX28	131.606	-1.280	1.948E-04	3.769E-03	112.201	-0.426	2.044E-01	5.062E-01
ENSG00000186318	BACE1	58.690	-1.571	2.148E-04	4.073E-03	54.825	-0.384	3.049E-01	6.153E-01
ENSG00000165507	DEPP1	135.434	-1.231	2.227E-04	4.169E-03	55.680	0.419	2.604E-01	5.702E-01
ENSG00000136295	TTYH3	713.661	-0.981	2.265E-04	4.215E-03	596.004	-0.061	7.910E-01	9.163E-01
ENSG00000164171	ITGA2	202.138	-1.069	2.282E-04	4.228E-03	155.199	-0.369	1.832E-01	4.824E-01
ENSG00000140093	SERPINA10	52.944	-1.509	2.511E-04	4.568E-03	41.841	-0.379	3.668E-01	6.712E-01
ENSG00000146376	ARHGAP18	1600.280	-0.802	2.557E-04	4.621E-03	1316.002	-0.218	3.157E-01	6.262E-01
ENSG00000133195	SLC39A11	157.871	-1.093	2.610E-04	4.686E-03	135.387	-0.156	6.107E-01	8.288E-01
ENSG00000081051	AFP	12826.571	-0.980	2.700E-04	4.834E-03	9911.816	-0.354	6.294E-02	2.751E-01
ENSG00000108375	RNF43	330.872	-0.874	2.772E-04	4.922E-03	430.778	-0.426	4.798E-02	2.381E-01

ENSG00000110721	CHKA	2381.314	-0.973	2.845E-04	5.036E-03	1774.524	-0.205	2.909E-01	6.011E-01
ENSG00000104635	SLC39A14	1455.777	-1.071	3.282E-04	5.716E-03	706.133	-0.051	8.378E-01	9.363E-01
ENSG00000048828	FAM120A	1433.175	-0.681	3.302E-04	5.740E-03	1482.803	-0.055	7.387E-01	8.915E-01
ENSG00000086061	DNAJA1	1299.883	-0.747	3.346E-04	5.763E-03	1022.342	-0.340	1.003E-01	3.542E-01
ENSG00000059573	ALDH18A1	472.319	-1.135	3.354E-04	5.764E-03	430.057	-0.040	8.743E-01	9.523E-01
ENSG00000016864	GLT8D1	143.957	-1.334	3.444E-04	5.894E-03	224.494	-0.500	4.701E-02	2.361E-01
ENSG00000117601	SERPINC1	298.733	-0.884	3.553E-04	6.026E-03	307.813	-0.201	4.420E-01	7.229E-01
ENSG00000116133	DHCR24	7575.841	-0.821	3.639E-04	6.147E-03	7509.070	-0.475	1.648E-03	3.030E-02
ENSG00000119421	NDUFA8	461.955	-0.838	3.665E-04	6.172E-03	547.698	-0.263	1.733E-01	4.693E-01
ENSG00000044446	PHKA2	186.943	-1.136	3.751E-04	6.261E-03	220.464	-0.292	2.773E-01	5.880E-01
ENSG00000165801	ARHGEF40	95.002	-1.347	3.795E-04	6.309E-03	82.607	-0.438	2.417E-01	5.495E-01
ENSG00000160785	SLC25A44	191.972	-1.012	3.992E-04	6.568E-03	182.429	-0.378	1.582E-01	4.484E-01
ENSG00000125398	SOX9	982.256	-0.920	4.266E-04	6.974E-03	1021.969	-0.387	5.968E-02	2.691E-01
ENSG00000132688	NES	74.621	-1.277	4.459E-04	7.235E-03	117.226	-0.223	5.045E-01	7.642E-01
ENSG00000063854	HAGH	511.174	-0.814	4.656E-04	7.477E-03	516.638	-0.083	7.140E-01	8.812E-01
ENSG00000182872	RBM10	340.534	-0.991	4.888E-04	7.763E-03	413.851	-0.384	1.089E-01	3.691E-01
ENSG00000174804	FZD4	602.425	-0.892	5.056E-04	7.980E-03	458.381	-0.478	3.178E-02	1.905E-01
ENSG00000120948	TARDBP	460.305	-0.867	5.165E-04	8.125E-03	363.549	-0.030	9.071E-01	9.656E-01
ENSG00000138867	GUCD1	729.808	-0.738	5.424E-04	8.508E-03	792.407	-0.294	1.052E-01	3.628E-01
ENSG00000143363	PRUNE1	186.954	-1.161	5.437E-04	8.518E-03	186.207	-0.290	2.904E-01	6.007E-01
ENSG00000234127	TRIM26	192.312	-1.106	5.635E-04	8.751E-03	221.596	-0.317	1.737E-01	4.695E-01
ENSG00000113719	ERGIC1	810.263	-0.824	6.068E-04	9.279E-03	693.494	-0.136	5.141E-01	7.700E-01
ENSG00000112118	MCM3	1703.793	-0.707	6.222E-04	9.480E-03	1300.069	-0.178	4.431E-01	7.233E-01
ENSG00000092964	DPYSL2	380.736	-0.826	6.505E-04	9.807E-03	509.444	-0.362	6.753E-02	2.851E-01
ENSG00000124523	SIRT5	221.325	-1.039	6.835E-04	1.027E-02	151.915	-0.284	3.486E-01	6.546E-01
ENSG00000128294	TPST2	57.960	-1.417	6.915E-04	1.036E-02	35.343	-0.477	2.327E-01	5.380E-01

ENSG00000257017	HP	132.789	-1.287	6.944E-04	1.039E-02	94.509	-0.209	5.133E-01	7.696E-01
ENSG00000148606	POLR3A	301.560	-0.923	7.232E-04	1.068E-02	329.858	-0.481	5.283E-02	2.511E-01
ENSG00000168246	UBTD2	165.904	-1.296	7.672E-04	1.121E-02	133.010	-0.205	5.080E-01	7.665E-01
ENSG00000120437	ACAT2	1857.549	-0.672	8.056E-04	1.167E-02	1424.468	-0.453	7.044E-03	7.805E-02
ENSG00000105810	CDK6	1680.149	-0.744	8.399E-04	1.205E-02	1212.769	-0.272	1.632E-01	4.540E-01
ENSG00000155850	SLC26A2	86.277	-1.177	8.961E-04	1.263E-02	125.792	-0.358	2.038E-01	5.056E-01
ENSG00000087338	GMCL1	315.973	-0.891	9.444E-04	1.321E-02	297.536	-0.448	5.335E-02	2.524E-01
ENSG00000115275	MOGS	313.363	-0.810	9.887E-04	1.377E-02	347.258	-0.429	6.860E-02	2.870E-01
ENSG00000175745	NR2F1	440.649	-0.823	9.952E-04	1.381E-02	490.637	-0.330	9.344E-02	3.392E-01
ENSG00000113161	HMGCR	504.738	-0.960	1.005E-03	1.391E-02	480.221	-0.459	2.322E-02	1.607E-01
ENSG00000069535	MAOB	205.612	-1.041	1.007E-03	1.393E-02	195.061	-0.417	1.209E-01	3.906E-01
ENSG00000176783	RUFY1	215.521	-0.874	1.010E-03	1.395E-02	251.135	-0.225	3.697E-01	6.734E-01
ENSG00000124253	PCK1	41.282	-1.439	1.028E-03	1.411E-02	13.883	-0.384	3.596E-01	NA
ENSG00000033100	CHPF2	54.968	-1.319	1.098E-03	1.483E-02	66.071	-0.221	5.276E-01	7.813E-01
ENSG00000176208	ATAD5	148.933	-1.021	1.096E-03	1.483E-02	133.377	-0.434	1.669E-01	4.598E-01
ENSG00000068724	TTC7A	195.270	-0.960	1.104E-03	1.488E-02	229.905	-0.290	2.289E-01	5.343E-01
ENSG00000103932	RPAP1	149.367	-0.932	1.111E-03	1.494E-02	268.124	-0.195	4.381E-01	7.213E-01
ENSG00000103187	COTL1	222.833	-0.894	1.220E-03	1.604E-02	190.008	0.229	4.322E-01	7.182E-01
ENSG00000167700	MFSD3	230.016	-0.944	1.239E-03	1.620E-02	262.235	-0.360	1.961E-01	4.975E-01
ENSG00000164252	AGGF1	185.319	-0.889	1.241E-03	1.620E-02	222.074	-0.399	1.181E-01	3.856E-01
ENSG00000180767	CHST13	845.110	-0.819	1.257E-03	1.638E-02	692.704	-0.271	4.872E-01	7.532E-01
ENSG00000149636	DSN1	740.931	-0.676	1.282E-03	1.661E-02	486.931	-0.294	2.296E-01	5.350E-01
ENSG00000205213	LGR4	383.159	-0.810	1.314E-03	1.686E-02	242.480	-0.480	4.696E-02	2.361E-01
ENSG00000162063	CCNF	296.893	-0.968	1.333E-03	1.705E-02	215.469	-0.393	1.463E-01	4.323E-01
ENSG00000123562	MORF4L2	1178.579	-0.681	1.357E-03	1.729E-02	1331.210	-0.216	2.128E-01	5.165E-01
ENSG00000143952	VPS54	202.942	-1.114	1.365E-03	1.735E-02	143.049	-0.335	3.431E-01	6.496E-01

ENSG00000115365	LANCL1	300.511	-0.940	1.370E-03	1.739E-02	296.063	-0.362	1.205E-01	3.906E-01
ENSG00000121691	CAT	379.458	-0.839	1.377E-03	1.742E-02	442.306	-0.338	9.134E-02	3.354E-01
ENSG00000056998	GYG2	304.953	-0.819	1.397E-03	1.763E-02	216.435	-0.014	9.548E-01	9.835E-01
ENSG00000138614	INTS14	97.501	-1.359	1.405E-03	1.769E-02	113.070	0.274	4.429E-01	7.231E-01
ENSG00000184787	UBE2G2	557.586	-0.694	1.413E-03	1.775E-02	465.551	-0.321	2.270E-01	5.320E-01
ENSG00000146535	GNA12	554.813	-0.671	1.423E-03	1.783E-02	393.510	-0.072	7.261E-01	8.861E-01
ENSG00000137563	GGH	338.890	-0.819	1.451E-03	1.812E-02	232.199	-0.176	6.089E-01	8.278E-01
ENSG00000106789	CORO2A	442.259	-0.797	1.473E-03	1.835E-02	396.089	-0.391	7.206E-02	2.943E-01
ENSG00000117335	CD46	264.495	-0.803	1.489E-03	1.853E-02	260.162	-0.490	3.887E-02	2.127E-01
ENSG00000158882	TOMM40L	140.196	-1.029	1.514E-03	1.875E-02	109.911	-0.196	5.114E-01	7.687E-01
ENSG00000139921	TMX1	147.515	-1.145	1.553E-03	1.911E-02	159.611	-0.166	5.605E-01	7.993E-01
ENSG00000134363	FST	608.216	-0.669	1.561E-03	1.914E-02	329.556	0.378	1.141E-01	3.782E-01
ENSG00000103423	DNAJA3	249.448	-0.901	1.581E-03	1.931E-02	220.330	-0.082	7.701E-01	9.054E-01
ENSG00000205730	ITPRIPL2	265.256	-0.856	1.582E-03	1.931E-02	235.247	-0.428	8.122E-02	3.152E-01
ENSG00000169047	IRS1	178.959	-0.874	1.606E-03	1.953E-02	159.382	-0.374	1.554E-01	4.445E-01
ENSG00000276293	PIP4K2B	376.286	-0.970	1.657E-03	2.005E-02	172.970	-0.488	1.616E-01	4.519E-01
ENSG00000142949	PTPRF	1258.798	-0.673	1.657E-03	2.005E-02	1227.051	-0.342	1.081E-01	3.672E-01
ENSG00000136720	HS6ST1	146.115	-1.185	1.662E-03	2.010E-02	79.129	-0.383	2.499E-01	5.593E-01
ENSG00000166833	NAV2	604.011	-0.680	1.676E-03	2.020E-02	643.159	-0.167	4.278E-01	7.152E-01
ENSG00000117791	MARC2	43.739	-1.349	1.681E-03	2.022E-02	47.854	-0.217	5.528E-01	7.942E-01
ENSG00000174173	TRMT10C	155.148	-0.965	1.703E-03	2.046E-02	135.419	-0.428	1.623E-01	4.528E-01
ENSG00000198056	PRIM1	107.552	-1.030	1.722E-03	2.059E-02	123.194	-0.156	5.984E-01	8.211E-01
ENSG00000135048	TMEM2	657.712	-0.885	1.822E-03	2.155E-02	587.014	-0.126	5.664E-01	8.023E-01
ENSG00000183340	JRKL	67.819	-1.274	1.849E-03	2.178E-02	73.031	-0.145	6.888E-01	8.672E-01
ENSG00000145692	BHMT	30.574	-1.607	1.861E-03	2.184E-02	8.402	-0.632	1.079E-01	NA
ENSG00000115194	SLC30A3	66.931	-1.284	1.868E-03	2.188E-02	38.667	-0.345	4.112E-01	7.051E-01

ENSG00000143971	ETAA1	37.069	-1.532	1.910E-03	2.222E-02	34.612	-0.149	7.203E-01	8.834E-01
ENSG00000168306	ACOX2	93.171	-1.133	1.967E-03	2.271E-02	59.695	-0.356	3.227E-01	6.306E-01
ENSG00000175970	UNC119B	110.265	-1.004	2.048E-03	2.350E-02	138.183	-0.251	3.991E-01	6.973E-01
ENSG00000100578	KIAA0586	133.824	-0.967	2.107E-03	2.409E-02	131.567	-0.398	1.863E-01	4.855E-01
ENSG00000278845	MRPL45	770.709	-0.749	2.113E-03	2.413E-02	545.748	-0.051	8.301E-01	9.329E-01
ENSG00000066813	ACSM2B	55.489	-1.303	2.117E-03	2.415E-02	57.984	-0.378	3.169E-01	6.270E-01
ENSG00000108821	COL1A1	37.691	-1.546	2.147E-03	2.441E-02	9.833	-0.564	1.213E-01	NA
ENSG00000099783	HNRNPM	2384.234	-0.574	2.158E-03	2.451E-02	2001.072	-0.113	4.867E-01	7.531E-01
ENSG00000152291	TGOLN2	1838.700	-0.591	2.184E-03	2.476E-02	2132.841	-0.118	5.228E-01	7.775E-01
ENSG00000119487	MAPKAP1	353.408	-0.754	2.193E-03	2.484E-02	399.037	-0.359	1.021E-01	3.580E-01
ENSG00000102054	RBBP7	606.415	-0.708	2.220E-03	2.507E-02	519.275	-0.336	1.206E-01	3.906E-01
ENSG00000135821	GLUL	715.920	-0.657	2.233E-03	2.516E-02	628.537	-0.271	1.614E-01	4.518E-01
ENSG00000077549	CAPZB	2933.026	-0.705	2.235E-03	2.517E-02	2947.463	-0.042	8.163E-01	9.264E-01
ENSG00000143740	SNAP47	108.642	-1.002	2.333E-03	2.604E-02	118.420	-0.386	2.013E-01	5.035E-01
ENSG00000091262	ABCC6	108.170	-0.988	2.364E-03	2.629E-02	146.517	-0.431	1.213E-01	3.907E-01
ENSG00000179387	ELMOD2	82.618	-1.197	2.387E-03	2.638E-02	72.395	-0.130	7.031E-01	8.745E-01
ENSG00000137869	CYP19A1	34.642	-1.510	2.652E-03	2.865E-02	36.616	0.190	6.354E-01	8.417E-01
ENSG00000198780	FAM169A	222.912	-0.895	2.668E-03	2.874E-02	270.256	-0.359	1.368E-01	4.180E-01
ENSG00000150977	RILPL2	230.977	-0.787	2.718E-03	2.921E-02	149.645	-0.472	1.592E-01	4.501E-01
ENSG00000160255	ITGB2	140.250	-1.083	2.728E-03	2.927E-02	133.688	-0.025	9.347E-01	9.761E-01
ENSG00000162600	OMA1	37.398	-1.314	2.863E-03	3.049E-02	37.718	0.069	8.596E-01	9.473E-01
ENSG00000111276	CDKN1B	211.875	-0.813	2.865E-03	3.049E-02	226.486	0.205	4.335E-01	7.186E-01
ENSG00000213699	SLC35F6	93.080	-1.096	2.876E-03	3.057E-02	291.411	-0.135	5.772E-01	8.086E-01
ENSG00000277161	PIGW	173.296	-0.819	2.923E-03	3.097E-02	137.725	0.200	5.093E-01	7.672E-01
ENSG00000168243	GNG4	298.876	-0.774	3.048E-03	3.201E-02	304.308	-0.373	1.739E-01	4.695E-01
ENSG00000163631	ALB	26266.375	-0.591	3.087E-03	3.231E-02	20157.922	-0.269	8.653E-02	3.254E-01

ENSG00000077097	TOP2B	472.690	-0.665	3.093E-03	3.234E-02	601.408	-0.300	1.038E-01	3.606E-01
ENSG00000165478	HEPACAM	34.815	-1.527	3.103E-03	3.239E-02	28.123	-0.617	1.352E-01	4.154E-01
ENSG00000173209	AHSA2P	196.623	-0.897	3.120E-03	3.255E-02	268.861	-0.309	2.132E-01	5.169E-01
ENSG00000123416	TUBA1B	11527.708	-0.608	3.157E-03	3.282E-02	9044.953	-0.255	2.225E-01	5.276E-01
ENSG00000132716	DCAF8	220.820	-0.807	3.204E-03	3.315E-02	212.948	-0.100	7.178E-01	8.828E-01
ENSG00000119227	PIGZ	39.567	-1.371	3.246E-03	3.345E-02	51.041	-0.301	4.426E-01	7.229E-01
ENSG00000198890	PRMT6	44.480	-1.459	3.254E-03	3.351E-02	43.781	-0.226	5.486E-01	7.916E-01
ENSG00000100867	DHRS2	45505.309	-0.579	3.268E-03	3.363E-02	44754.935	0.017	9.144E-01	9.684E-01
ENSG00000129317	PUS7L	171.997	-0.987	3.271E-03	3.363E-02	156.348	-0.106	6.986E-01	8.720E-01
ENSG00000099810	MTAP	228.218	-0.810	3.279E-03	3.363E-02	221.029	-0.397	1.315E-01	4.098E-01
ENSG00000126878	AIF1L	313.815	-0.818	3.348E-03	3.409E-02	214.414	-0.097	7.445E-01	8.937E-01
ENSG00000197894	ADH5	1682.529	-0.675	3.366E-03	3.425E-02	1434.958	0.026	8.995E-01	9.629E-01
ENSG00000166135	HIF1AN	300.409	-0.785	3.422E-03	3.462E-02	176.218	-0.405	1.924E-01	4.932E-01
ENSG00000089685	BIRC5	1325.116	-0.647	3.485E-03	3.506E-02	1279.879	-0.377	5.382E-02	2.536E-01
ENSG00000168496	FEN1	944.339	-0.782	3.500E-03	3.519E-02	574.307	0.186	4.646E-01	7.413E-01
ENSG00000159055	MIS18A	434.301	-0.676	3.547E-03	3.555E-02	293.110	-0.034	8.829E-01	9.550E-01
ENSG00000103042	SLC38A7	132.391	-0.902	3.558E-03	3.560E-02	138.974	-0.428	1.179E-01	3.856E-01
ENSG00000152270	PDE3B	123.899	-0.880	3.642E-03	3.628E-02	108.939	-0.215	4.565E-01	7.356E-01
ENSG00000119772	DNMT3A	427.444	-0.667	3.667E-03	3.646E-02	369.590	-0.418	5.056E-02	2.448E-01
ENSG00000170439	METTL7B	44.619	-1.302	3.685E-03	3.656E-02	66.735	-0.457	2.245E-01	5.291E-01
ENSG00000158555	GDPD5	167.895	-0.889	3.780E-03	3.712E-02	117.892	-0.355	2.304E-01	5.360E-01
ENSG00000009950	MLXIPL	1687.492	-0.672	3.775E-03	3.712E-02	1213.564	-0.069	8.518E-01	9.441E-01
ENSG00000041357	PSMA4	675.516	-0.614	3.770E-03	3.712E-02	918.604	0.123	5.135E-01	7.696E-01
ENSG00000132182	NUP210	293.639	-0.709	3.837E-03	3.751E-02	274.816	-0.362	1.131E-01	3.763E-01
ENSG00000057019	DCBLD2	538.137	-0.630	3.848E-03	3.759E-02	621.260	-0.224	2.854E-01	5.965E-01
ENSG00000257365	FNTB	207.452	-0.800	3.855E-03	3.762E-02	207.504	-0.298	2.846E-01	5.960E-01

ENSG00000167034	NKX3-1	48.884	-1.330	3.905E-03	3.792E-02	72.147	-0.488	1.494E-01	4.367E-01
ENSG00000189339	SLC35E2B	212.597	-0.823	3.916E-03	3.799E-02	289.529	-0.498	4.129E-02	2.199E-01
ENSG00000130193	THEM6	470.356	-0.859	3.994E-03	3.857E-02	461.962	-0.475	2.935E-02	1.819E-01
ENSG00000167114	SLC27A4	73.627	-1.247	4.001E-03	3.861E-02	67.164	-0.168	6.356E-01	8.417E-01
ENSG00000125843	AP5S1	192.647	-0.887	4.047E-03	3.894E-02	195.925	-0.370	1.446E-01	4.299E-01
ENSG00000115207	GTF3C2	258.141	-0.952	4.096E-03	3.934E-02	253.575	-0.404	8.887E-02	3.304E-01
ENSG00000183044	ABAT	246.860	-0.819	4.179E-03	3.985E-02	248.953	-0.430	9.612E-02	3.447E-01
ENSG00000171320	ESCO2	85.761	-1.116	4.217E-03	4.018E-02	85.517	-0.409	2.610E-01	5.708E-01
ENSG00000198130	HIBCH	285.490	-0.830	4.308E-03	4.093E-02	252.121	-0.236	3.607E-01	6.659E-01
ENSG00000108395	TRIM37	217.420	-0.761	4.345E-03	4.119E-02	248.587	0.055	8.113E-01	9.241E-01
ENSG00000170522	ELOVL6	87.804	-1.026	4.388E-03	4.147E-02	111.235	-0.398	1.788E-01	4.755E-01
ENSG00000103174	NAGPA	332.198	-0.699	4.464E-03	4.203E-02	326.161	-0.193	4.729E-01	7.468E-01
ENSG00000198873	GRK5	83.623	-1.018	4.470E-03	4.206E-02	84.838	-0.218	5.197E-01	7.753E-01
ENSG00000154920	EME1	87.122	-1.017	4.479E-03	4.212E-02	72.219	-0.471	1.890E-01	4.880E-01
ENSG00000132842	AP3B1	265.319	-0.837	4.528E-03	4.248E-02	292.108	-0.306	1.736E-01	4.693E-01
ENSG00000168310	IRF2	100.663	-1.036	4.557E-03	4.265E-02	171.143	-0.343	1.937E-01	4.947E-01
ENSG00000128512	DOCK4	107.984	-0.929	4.614E-03	4.291E-02	87.327	-0.245	4.417E-01	7.227E-01
ENSG00000011021	CLCN6	50.127	-1.273	4.643E-03	4.315E-02	61.742	-0.226	5.191E-01	7.744E-01
ENSG00000139318	DUSP6	325.839	-0.797	4.696E-03	4.348E-02	219.691	0.365	1.955E-01	4.967E-01
ENSG00000173894	CBX2	102.620	-0.936	4.743E-03	4.388E-02	71.295	-0.386	2.764E-01	5.867E-01
ENSG00000085185	BCORL1	93.686	-0.972	4.770E-03	4.394E-02	87.024	-0.370	2.287E-01	5.340E-01
ENSG00000157870	FAM213B	249.795	-0.817	4.809E-03	4.411E-02	212.148	0.166	5.174E-01	7.727E-01
ENSG00000213462	ERV3-1	113.456	-0.995	4.913E-03	4.491E-02	148.855	-0.302	2.788E-01	5.894E-01
ENSG00000167074	TEF	32.400	-1.300	4.939E-03	4.498E-02	17.487	-0.635	1.300E-01	NA
ENSG00000130349	C6orf203	38.430	-1.220	4.931E-03	4.498E-02	44.491	-0.412	3.053E-01	6.154E-01
ENSG00000107890	ANKRD26	253.397	-0.920	4.929E-03	4.498E-02	306.569	-0.300	2.286E-01	5.340E-01

ENSG00000167699	GLOD4	348.326	-0.762	4.935E-03	4.498E-02	291.546	-0.173	4.902E-01	7.551E-01
ENSG00000102910	LONP2	333.159	-0.688	4.939E-03	4.498E-02	337.674	-0.500	2.103E-02	1.523E-01
ENSG00000111269	CREBL2	154.811	-0.849	5.092E-03	4.617E-02	114.710	-0.125	6.813E-01	8.631E-01
ENSG00000257742	RP11-350F4.2	96.480	-0.946	5.124E-03	4.634E-02	96.059	-0.131	6.898E-01	8.680E-01
ENSG00000166347	CYB5A	1603.630	-0.687	5.146E-03	4.644E-02	1473.994	-0.138	4.254E-01	7.136E-01
ENSG00000058668	ATP2B4	33.969	-1.298	5.154E-03	4.644E-02	19.193	-0.101	8.101E-01	NA
ENSG00000183765	CHEK2	160.390	-0.938	5.242E-03	4.704E-02	131.474	-0.083	7.997E-01	9.193E-01
ENSG00000136240	KDELR2	1013.080	-0.681	5.274E-03	4.729E-02	978.204	-0.205	2.949E-01	6.049E-01
ENSG00000137274	BPHL	72.604	-1.118	5.342E-03	4.783E-02	84.778	-0.379	2.867E-01	5.977E-01
ENSG00000128944	KNSTRN	229.994	-0.837	5.353E-03	4.790E-02	173.662	-0.199	4.997E-01	7.605E-01
ENSG00000248092	NNT-AS1	144.746	-0.821	5.382E-03	4.803E-02	195.855	-0.167	5.365E-01	7.866E-01
ENSG00000123684	LPGAT1	231.219	-0.746	5.432E-03	4.830E-02	292.725	-0.034	8.830E-01	9.550E-01
ENSG00000115282	TTC31	124.409	-0.956	5.483E-03	4.862E-02	179.584	-0.432	1.497E-01	4.372E-01
ENSG00000197892	KIF13B	128.447	-0.923	5.509E-03	4.871E-02	122.158	-0.180	5.543E-01	7.947E-01
ENSG00000124299	PEPD	270.277	-0.844	5.740E-03	5.040E-02	250.817	0.021	9.248E-01	9.727E-01
ENSG00000100116	GCAT	117.717	-0.907	5.802E-03	5.070E-02	105.197	-0.160	5.947E-01	8.187E-01
ENSG00000183077	AFMID	879.684	-0.592	5.959E-03	5.172E-02	569.708	-0.161	4.867E-01	7.531E-01
ENSG00000130544	ZNF557	66.210	-1.068	6.109E-03	5.281E-02	56.179	-0.360	3.219E-01	6.298E-01
ENSG00000117899	MESD	1624.156	-0.629	6.117E-03	5.284E-02	942.079	-0.358	1.472E-01	4.339E-01
ENSG00000158315	RHBDL2	33.030	-1.387	6.145E-03	5.302E-02	18.562	-0.020	9.627E-01	NA
ENSG00000169951	ZNF764	66.016	-1.126	6.258E-03	5.384E-02	62.052	0.071	8.458E-01	9.409E-01
ENSG00000118707	TGIF2	370.368	-0.812	6.315E-03	5.415E-02	346.859	-0.054	7.944E-01	9.179E-01
ENSG00000176623	RMDN1	179.229	-0.882	6.378E-03	5.452E-02	242.350	-0.401	1.435E-01	4.289E-01
ENSG00000102178	UBL4A	177.814	-0.825	6.447E-03	5.481E-02	161.102	0.213	4.216E-01	7.118E-01
ENSG00000151718	WWC2	397.907	-0.710	6.441E-03	5.481E-02	502.745	-0.496	1.851E-02	1.415E-01
ENSG00000133026	MYH10	1159.877	-0.679	6.461E-03	5.481E-02	969.568	-0.407	2.687E-02	1.727E-01

ENSG00000125686	MED1	1088.139	-0.558	6.555E-03	5.517E-02	1100.486	-0.134	4.210E-01	7.116E-01
ENSG00000231074	HCG18	146.883	-0.842	6.583E-03	5.534E-02	186.309	-0.348	1.747E-01	4.700E-01
ENSG00000174990	CA5A	80.889	-1.092	6.668E-03	5.590E-02	80.234	-0.310	3.950E-01	6.935E-01
ENSG00000131732	ZCCHC9	315.672	-0.674	6.714E-03	5.617E-02	310.281	-0.365	1.178E-01	3.855E-01
ENSG00000122787	AKR1D1	45.179	-1.202	6.720E-03	5.619E-02	58.647	-0.343	3.476E-01	6.536E-01
ENSG00000138073	PREB	842.639	-0.788	6.725E-03	5.619E-02	503.086	-0.004	9.881E-01	9.957E-01
ENSG00000135341	MAP3K7	297.057	-0.669	6.762E-03	5.647E-02	279.487	-0.199	3.973E-01	6.957E-01
ENSG00000012963	UBR7	137.738	-0.940	6.811E-03	5.677E-02	130.417	-0.392	2.187E-01	5.238E-01
ENSG00000130939	UBE4B	219.941	-0.841	6.832E-03	5.687E-02	261.558	-0.246	2.901E-01	6.004E-01
ENSG00000174567	GOLT1A	186.957	-0.776	6.870E-03	5.699E-02	115.944	-0.084	7.716E-01	9.065E-01
ENSG00000079739	PGM1	344.495	-0.703	6.948E-03	5.714E-02	265.710	-0.388	9.251E-02	3.376E-01
ENSG00000171634	BPTF	1908.643	-0.698	6.943E-03	5.714E-02	2115.585	-0.441	1.687E-02	1.332E-01
ENSG00000136156	ITM2B	806.315	-0.592	7.029E-03	5.749E-02	786.564	-0.465	1.623E-02	1.307E-01
ENSG00000087502	ERGIC2	237.723	-0.767	7.101E-03	5.793E-02	130.457	-0.282	3.867E-01	6.860E-01
ENSG00000168936	TMEM129	230.877	-0.741	7.168E-03	5.826E-02	199.887	0.140	5.866E-01	8.141E-01
ENSG00000126391	FRMD8	1091.343	-0.605	7.233E-03	5.867E-02	932.678	-0.362	6.005E-02	2.697E-01
ENSG00000135148	TRAFD1	76.275	-0.994	7.238E-03	5.867E-02	69.767	-0.353	3.017E-01	6.120E-01
ENSG00000070540	WIPI1	101.817	-0.874	7.253E-03	5.876E-02	93.384	-0.496	1.230E-01	3.936E-01
ENSG00000269976	AC012065.3	120.311	-0.868	7.420E-03	5.974E-02	114.469	0.066	8.424E-01	9.390E-01
ENSG00000164638	SLC29A4	102.644	-0.961	7.527E-03	6.044E-02	128.370	-0.112	7.167E-01	8.825E-01
ENSG00000115459	ELMOD3	30.989	-1.221	7.684E-03	6.147E-02	29.219	-0.363	3.763E-01	6.776E-01
ENSG00000239887	C1orf226	63.339	-1.176	7.712E-03	6.164E-02	40.432	-0.290	4.861E-01	7.530E-01
ENSG00000177971	IMP3	2169.321	-0.576	7.798E-03	6.211E-02	1843.622	-0.470	9.916E-03	9.753E-02
ENSG00000129195	PIMREG	105.433	-0.953	7.927E-03	6.295E-02	94.544	-0.121	7.014E-01	8.737E-01
ENSG00000143198	MGST3	374.344	-0.900	8.070E-03	6.388E-02	298.031	-0.328	2.262E-01	5.311E-01
ENSG00000116771	AGMAT	1062.812	-0.652	8.175E-03	6.447E-02	990.651	-0.439	5.180E-02	2.484E-01

ENSG00000091513	TF	7723.301	-0.579	8.219E-03	6.474E-02	5606.143	-0.156	6.639E-01	8.547E-01
ENSG00000107185	RGP1	226.497	-0.720	8.236E-03	6.484E-02	215.228	-0.037	8.936E-01	9.603E-01
ENSG00000144026	ZNF514	43.274	-1.184	8.280E-03	6.510E-02	59.684	-0.465	2.398E-01	5.470E-01
ENSG00000189266	PNRC2	178.365	-0.811	8.476E-03	6.644E-02	179.200	-0.125	6.924E-01	8.684E-01
ENSG00000183688	RFLNB	244.552	-0.733	8.511E-03	6.658E-02	168.242	-0.157	5.494E-01	7.918E-01
ENSG00000125912	NCLN	457.792	-0.582	8.559E-03	6.680E-02	226.565	-0.209	4.305E-01	7.168E-01
ENSG00000168393	DTYMK	562.129	-0.590	8.621E-03	6.720E-02	532.477	-0.266	2.356E-01	5.420E-01
ENSG00000134243	SORT1	235.308	-0.708	8.633E-03	6.725E-02	246.954	0.186	4.270E-01	7.147E-01
ENSG00000114988	LMAN2L	206.535	-0.799	8.679E-03	6.741E-02	169.706	-0.299	3.302E-01	6.378E-01
ENSG00000119139	TJP2	640.490	-0.591	8.814E-03	6.815E-02	743.617	-0.129	4.990E-01	7.602E-01
ENSG00000145192	AHSG	10378.432	-0.541	8.847E-03	6.825E-02	7607.772	0.072	6.226E-01	8.361E-01
ENSG00000253719	ATXN7L3B	356.921	-0.703	8.911E-03	6.858E-02	344.587	-0.191	4.043E-01	7.008E-01
ENSG00000183486	MX2	152.622	-0.776	9.040E-03	6.928E-02	147.713	-0.346	2.286E-01	5.340E-01
ENSG00000198794	SCAMP5	235.729	-0.692	9.059E-03	6.938E-02	206.227	-0.441	2.099E-01	5.130E-01
ENSG00000170456	DENND5B	237.728	-0.677	9.213E-03	7.031E-02	291.662	-0.478	3.945E-02	2.150E-01
ENSG00000107929	LARP4B	183.787	-0.801	9.290E-03	7.075E-02	238.675	-0.159	5.247E-01	7.785E-01
ENSG00000168411	RFWD3	553.307	-0.583	9.353E-03	7.108E-02	454.756	-0.155	4.806E-01	7.508E-01
ENSG00000164818	DNAAF5	271.900	-0.844	9.402E-03	7.137E-02	295.914	0.135	5.705E-01	8.056E-01
ENSG00000175782	SLC35E3	462.738	-0.614	9.410E-03	7.139E-02	352.471	-0.319	1.541E-01	4.423E-01
ENSG00000118260	CREB1	214.120	-0.768	9.485E-03	7.183E-02	334.921	0.002	9.919E-01	9.967E-01
ENSG00000188242	AC010442.1	61.227	-1.076	9.524E-03	7.200E-02	57.222	-0.396	2.819E-01	5.925E-01
ENSG00000162433	AK4	1089.883	-0.630	9.552E-03	7.209E-02	636.641	-0.497	2.273E-02	1.588E-01
ENSG00000247556	OIP5-AS1	673.753	-0.541	9.642E-03	7.251E-02	421.872	-0.332	1.443E-01	4.297E-01
ENSG00000175220	ARHGAP1	368.977	-0.638	9.814E-03	7.348E-02	429.617	-0.263	1.883E-01	4.872E-01
ENSG00000023445	BIRC3	27.092	1.376	9.818E-03	7.348E-02	59.485	1.423	3.173E-04	1.007E-02
ENSG00000116791	CRYZ	528.404	-0.721	9.828E-03	7.351E-02	398.166	0.382	1.332E-01	4.121E-01

ENSG00000177352	CCDC71	57.517	-1.128	9.870E-03	7.378E-02	53.821	-0.454	2.077E-01	5.103E-01
ENSG00000063322	MED29	129.069	-0.831	9.909E-03	7.399E-02	117.251	-0.254	4.191E-01	7.095E-01
ENSG00000163257	DCAF16	356.460	-0.602	1.013E-02	7.522E-02	406.771	-0.294	1.876E-01	4.870E-01
ENSG00000105953	OGDH	89.920	-0.928	1.016E-02	7.532E-02	91.661	-0.385	2.196E-01	5.249E-01
ENSG00000168890	TMEM150A	206.701	-0.760	1.035E-02	7.633E-02	150.590	-0.100	7.589E-01	9.012E-01
ENSG00000108946	PRKAR1A	1405.606	-0.528	1.036E-02	7.633E-02	817.062	0.021	9.239E-01	9.721E-01
ENSG00000168874	ATOH8	26.972	1.355	1.037E-02	7.638E-02	36.048	0.749	7.108E-02	2.919E-01
ENSG00000165819	METTL3	139.918	-0.858	1.053E-02	7.734E-02	159.876	-0.192	4.689E-01	7.442E-01
ENSG00000140025	EFCAB11	95.700	-0.871	1.057E-02	7.760E-02	83.780	-0.101	7.582E-01	9.010E-01
ENSG00000142677	IL22RA1	57.525	-1.196	1.075E-02	7.863E-02	46.632	0.144	6.881E-01	8.670E-01
ENSG00000183718	TRIM52	39.162	-1.149	1.094E-02	7.960E-02	41.701	-0.420	2.907E-01	6.007E-01
ENSG00000162878	PKDCC	388.757	-1.249	1.101E-02	7.986E-02	168.918	-0.127	7.566E-01	9.007E-01
ENSG00000167840	ZNF232	78.190	-0.927	1.108E-02	8.019E-02	81.233	-0.360	2.742E-01	5.842E-01
ENSG00000165675	ENOX2	111.212	-0.950	1.122E-02	8.087E-02	104.467	-0.355	2.656E-01	5.754E-01
ENSG00000102595	UGGT2	92.631	-0.905	1.129E-02	8.131E-02	105.171	-0.497	1.513E-01	4.389E-01
ENSG00000075407	ZNF37A	196.181	-0.744	1.135E-02	8.168E-02	223.267	-0.295	2.352E-01	5.414E-01
ENSG00000111077	TNS2	71.452	-0.926	1.157E-02	8.294E-02	51.068	-0.206	5.690E-01	8.042E-01
ENSG00000197977	ELOVL2	436.723	-0.582	1.164E-02	8.342E-02	587.071	-0.341	7.694E-02	3.050E-01
ENSG00000170542	SERPINB9	46.327	-1.071	1.165E-02	8.342E-02	26.554	-0.295	4.638E-01	7.409E-01
ENSG00000236287	ZBED5	58.791	-0.955	1.186E-02	8.437E-02	64.041	-0.466	2.551E-01	5.656E-01
ENSG00000108100	CCNY	661.980	-0.623	1.186E-02	8.437E-02	560.064	0.154	4.751E-01	7.480E-01
ENSG00000131462	TUBG1	650.633	-0.573	1.184E-02	8.437E-02	742.203	-0.020	9.116E-01	9.679E-01
ENSG00000118762	PKD2	58.276	-1.041	1.187E-02	8.442E-02	55.149	-0.406	2.629E-01	5.730E-01
ENSG00000130347	RTN4IP1	58.759	-1.034	1.189E-02	8.445E-02	49.621	-0.204	5.974E-01	8.202E-01
ENSG00000091039	OSBPL8	316.259	-0.660	1.197E-02	8.481E-02	291.180	-0.163	5.824E-01	8.119E-01
ENSG00000088986	DYNLL1	1681.621	-0.595	1.200E-02	8.497E-02	1711.349	-0.290	1.046E-01	3.618E-01

ENSG00000162139	NEU3	178.164	-0.726	1.202E-02	8.504E-02	220.554	-0.398	1.207E-01	3.906E-01
ENSG00000261324	AC010168.2	54.592	-1.170	1.213E-02	8.557E-02	40.464	-0.377	3.454E-01	6.514E-01
ENSG00000136935	GOLGA1	162.131	-0.712	1.218E-02	8.582E-02	183.792	-0.488	5.994E-02	2.696E-01
ENSG00000151348	EXT2	127.552	-0.826	1.222E-02	8.596E-02	118.123	-0.113	7.038E-01	8.747E-01
ENSG00000174243	DDX23	280.097	-0.645	1.229E-02	8.630E-02	292.728	-0.181	4.606E-01	7.386E-01
ENSG00000102096	PIM2	300.660	-0.669	1.230E-02	8.632E-02	246.600	-0.015	9.572E-01	9.839E-01
ENSG00000183145	RIPPLY3	58.372	-0.956	1.240E-02	8.678E-02	34.543	0.114	7.761E-01	9.088E-01
ENSG00000163110	PDLIM5	1431.994	-0.501	1.252E-02	8.743E-02	1027.859	-0.094	6.388E-01	8.432E-01
ENSG00000151665	PIGF	99.711	-0.969	1.264E-02	8.788E-02	74.811	-0.174	6.510E-01	8.495E-01
ENSG00000021776	AQR	203.463	-0.687	1.270E-02	8.816E-02	187.156	-0.127	6.543E-01	8.507E-01
ENSG00000244005	NFS1	254.991	-0.693	1.271E-02	8.821E-02	297.402	-0.484	2.740E-02	1.741E-01
ENSG00000182108	DEXI	651.717	-0.546	1.273E-02	8.832E-02	872.368	-0.492	1.057E-02	1.005E-01
ENSG00000152818	UTRN	550.155	-0.750	1.277E-02	8.841E-02	589.532	-0.332	1.111E-01	3.725E-01
ENSG00000145287	PLAC8	35.687	-1.166	1.290E-02	8.904E-02	62.518	0.342	3.443E-01	6.510E-01
ENSG00000148773	MKI67	1519.686	-0.688	1.299E-02	8.939E-02	1324.531	-0.463	1.708E-02	1.340E-01
ENSG00000119523	ALG2	338.917	-0.597	1.298E-02	8.939E-02	266.060	-0.260	2.891E-01	5.997E-01
ENSG00000172534	HCFC1	1070.923	-0.510	1.307E-02	8.984E-02	1014.008	-0.253	1.978E-01	4.991E-01
ENSG00000163884	KLF15	30.723	-1.220	1.319E-02	9.028E-02	9.590	0.717	7.197E-02	NA
ENSG00000090612	ZNF268	106.288	-0.852	1.316E-02	9.028E-02	97.730	-0.150	6.331E-01	8.411E-01
ENSG00000099864	PALM	256.386	-0.714	1.319E-02	9.028E-02	269.261	-0.349	2.690E-01	5.785E-01
ENSG00000243364	EFNA4	44.955	-1.232	1.333E-02	9.107E-02	47.786	-0.229	5.683E-01	8.036E-01
ENSG00000134453	RBM17	1569.364	0.466	1.344E-02	9.153E-02	1088.474	0.628	2.839E-03	4.365E-02
ENSG00000035499	DEPDC1B	227.914	-0.790	1.346E-02	9.160E-02	153.784	-0.134	6.353E-01	8.417E-01
ENSG00000214290	COLCA2	76.532	-0.989	1.368E-02	9.269E-02	68.549	0.128	7.439E-01	8.937E-01
ENSG00000172164	SNTB1	388.377	-0.828	1.373E-02	9.287E-02	264.620	0.246	3.774E-01	6.781E-01
ENSG00000129562	DAD1	657.538	-0.521	1.376E-02	9.299E-02	852.685	-0.290	9.618E-02	3.447E-01

ENSG00000144677	CTDSPL	352.135	-0.602	1.384E-02	9.334E-02	276.648	-0.128	6.042E-01	8.251E-01
ENSG00000198585	NUDT16	170.029	-0.720	1.395E-02	9.384E-02	166.158	-0.009	9.729E-01	9.894E-01
ENSG00000107789	MINPP1	160.383	-0.714	1.413E-02	9.463E-02	158.241	-0.237	4.146E-01	7.075E-01
ENSG00000146215	CRIP3	41.092	-1.081	1.415E-02	9.464E-02	23.039	-0.816	5.067E-02	2.451E-01
ENSG00000115884	SDC1	859.669	-0.539	1.422E-02	9.500E-02	491.412	-0.183	4.422E-01	7.229E-01
ENSG00000144045	DQX1	134.095	-0.843	1.430E-02	9.528E-02	111.381	-0.290	3.568E-01	6.628E-01
ENSG00000135317	SNX14	278.320	-0.657	1.438E-02	9.548E-02	308.062	-0.423	6.952E-02	2.888E-01
ENSG00000171848	RRM2	961.900	-0.525	1.438E-02	9.548E-02	725.154	-0.225	3.200E-01	6.291E-01
ENSG00000104687	GSR	1456.324	0.497	1.438E-02	9.548E-02	1016.910	0.697	5.052E-04	1.424E-02
ENSG00000267010	RP11-108P20.1	35.159	-1.131	1.441E-02	9.559E-02	27.340	-0.509	2.065E-01	5.091E-01
ENSG00000160209	PDXK	645.657	-0.525	1.449E-02	9.582E-02	509.687	-0.337	9.795E-02	3.485E-01
ENSG00000158186	MRAS	126.640	-0.751	1.463E-02	9.647E-02	230.929	-0.192	4.993E-01	7.602E-01
ENSG00000221890	NPTXR	194.269	-0.771	1.475E-02	9.702E-02	207.861	-0.172	4.982E-01	7.599E-01
ENSG00000014216	CAPN1	211.751	-0.673	1.480E-02	9.718E-02	236.726	-0.256	3.051E-01	6.153E-01
ENSG00000079385	CEACAM1	88.615	-0.970	1.489E-02	9.742E-02	70.028	-0.076	8.355E-01	9.350E-01
ENSG00000136231	IGF2BP3	312.563	-0.714	1.489E-02	9.742E-02	434.610	-0.391	5.023E-02	2.435E-01
ENSG00000139651	ZNF740	56.621	-1.080	1.515E-02	9.842E-02	49.236	-0.314	4.124E-01	7.061E-01
ENSG00000163297	ANTXR2	48.707	-1.006	1.518E-02	9.854E-02	20.242	-0.230	5.813E-01	NA
ENSG00000178764	ZHX2	122.991	-0.934	1.519E-02	9.854E-02	97.007	-0.163	6.451E-01	8.463E-01
ENSG00000178921	PFAS	172.958	-0.730	1.524E-02	9.875E-02	157.060	-0.200	4.739E-01	7.471E-01
ENSG00000157379	DHRS1	1284.959	-0.544	1.527E-02	9.885E-02	1499.429	-0.436	2.514E-02	1.672E-01
ENSG00000124217	MOCS3	65.732	-1.062	1.548E-02	9.988E-02	59.152	-0.274	4.247E-01	7.133E-01
ENSG00000102763	VWA8	50.302	-1.010	1.558E-02	1.003E-01	58.190	-0.294	3.989E-01	6.972E-01
ENSG00000140406	TLNRD1	261.605	-0.676	1.558E-02	1.003E-01	195.615	-0.280	3.101E-01	6.196E-01
ENSG00000166133	RPUSD2	183.183	-0.681	1.576E-02	1.012E-01	146.637	-0.073	7.967E-01	9.187E-01
ENSG00000154269	ENPP3	44.863	-1.176	1.597E-02	1.021E-01	43.175	0.194	6.096E-01	8.279E-01

ENSG00000130713	EXOSC2	130.662	-0.765	1.602E-02	1.023E-01	130.682	-0.293	3.333E-01	6.408E-01
ENSG00000175283	DOLK	58.226	-0.920	1.611E-02	1.026E-01	49.878	-0.328	3.912E-01	6.894E-01
ENSG00000241935	HOGA1	72.276	-0.900	1.610E-02	1.026E-01	42.973	-0.286	4.730E-01	7.468E-01
ENSG00000055130	CUL1	280.884	-0.623	1.611E-02	1.026E-01	275.271	-0.375	1.286E-01	4.042E-01
ENSG00000168517	HEXIM2	186.752	-0.706	1.613E-02	1.026E-01	186.252	-0.384	1.626E-01	4.534E-01
ENSG00000135108	FBXO21	218.145	-0.627	1.622E-02	1.031E-01	206.664	-0.201	4.370E-01	7.205E-01
ENSG00000186635	ARAP1	409.293	-0.717	1.624E-02	1.031E-01	295.552	0.022	9.442E-01	9.794E-01
ENSG00000105176	URI1	1007.447	-0.642	1.650E-02	1.043E-01	921.612	-0.361	7.020E-02	2.899E-01
ENSG00000166562	SEC11C	740.343	-0.628	1.651E-02	1.043E-01	617.798	0.025	9.087E-01	9.666E-01
ENSG00000164068	RNF123	184.247	-0.769	1.686E-02	1.062E-01	190.281	-0.480	6.266E-02	2.750E-01
ENSG00000189306	RRP7A	350.466	-0.586	1.695E-02	1.065E-01	302.024	-0.472	6.955E-02	2.888E-01
ENSG00000185298	CCDC137	433.608	-0.645	1.699E-02	1.067E-01	301.821	-0.357	1.866E-01	4.857E-01
ENSG00000087274	ADD1	463.297	-0.569	1.706E-02	1.069E-01	534.723	-0.255	2.160E-01	5.213E-01
ENSG00000052795	FNIP2	283.512	-0.629	1.720E-02	1.077E-01	224.414	-0.233	3.632E-01	6.680E-01
ENSG00000143815	LBR	814.857	-0.542	1.740E-02	1.087E-01	660.984	-0.268	2.271E-01	5.320E-01
ENSG00000075711	DLG1	507.993	-0.614	1.743E-02	1.089E-01	510.978	-0.384	7.239E-02	2.948E-01
ENSG00000072310	SREBF1	74.685	-0.878	1.768E-02	1.102E-01	59.578	0.056	8.740E-01	9.523E-01
ENSG00000152284	TCF7L1	35.687	-1.068	1.772E-02	1.102E-01	44.769	-0.302	4.243E-01	7.132E-01
ENSG00000101138	CSTF1	237.331	-0.610	1.780E-02	1.105E-01	235.310	-0.151	5.288E-01	7.821E-01
ENSG00000139266	MARCH9	41.068	-1.030	1.782E-02	1.105E-01	45.071	-0.374	3.213E-01	6.294E-01
ENSG00000160094	ZNF362	172.679	-0.702	1.783E-02	1.105E-01	260.959	-0.251	2.996E-01	6.100E-01
ENSG00000101004	NINL	164.713	-0.709	1.792E-02	1.108E-01	215.243	-0.131	6.683E-01	8.572E-01
ENSG00000163939	PBRM1	418.601	-0.551	1.799E-02	1.109E-01	399.219	-0.383	7.687E-02	3.049E-01
ENSG00000119559	C19orf25	123.959	-0.731	1.811E-02	1.114E-01	142.186	-0.253	4.117E-01	7.054E-01
ENSG00000173064	HECTD4	69.950	-0.871	1.819E-02	1.118E-01	73.597	-0.265	4.188E-01	7.095E-01
ENSG00000163624	CDS1	53.817	-1.018	1.823E-02	1.119E-01	117.638	-0.207	5.035E-01	7.637E-01

ENSG00000239382	ALKBH6	44.896	-1.179	1.827E-02	1.120E-01	44.504	-0.253	4.969E-01	7.593E-01
ENSG00000140396	NCOA2	125.999	-0.735	1.826E-02	1.120E-01	182.799	-0.167	5.236E-01	7.782E-01
ENSG00000159228	CBR1	71.738	-0.857	1.829E-02	1.121E-01	98.536	-0.303	3.424E-01	6.489E-01
ENSG00000106399	RPA3	422.921	-0.664	1.864E-02	1.138E-01	421.056	-0.213	2.993E-01	6.098E-01
ENSG00000139636	LMBR1L	53.363	-0.994	1.872E-02	1.140E-01	55.515	-0.434	2.591E-01	5.693E-01
ENSG00000166140	ZFYVE19	81.153	-0.838	1.872E-02	1.140E-01	102.579	0.081	8.059E-01	9.226E-01
ENSG00000130338	TULP4	262.924	-0.596	1.937E-02	1.168E-01	342.033	0.007	9.775E-01	9.909E-01
ENSG00000119906	SLF2	255.581	-0.628	1.947E-02	1.173E-01	265.222	-0.481	4.940E-02	2.416E-01
ENSG00000100523	DDHD1	79.657	-0.871	1.951E-02	1.175E-01	91.739	-0.111	7.260E-01	8.861E-01
ENSG00000198099	ADH4	155.807	-0.798	1.960E-02	1.179E-01	86.426	-0.438	1.941E-01	4.953E-01
ENSG00000188938	FAM120AOS	128.914	-0.699	1.973E-02	1.183E-01	211.330	-0.290	2.785E-01	5.891E-01
ENSG00000122692	SMU1	200.038	-0.642	2.003E-02	1.195E-01	173.263	-0.069	7.949E-01	9.181E-01
ENSG00000198734	F5	322.453	-0.575	2.008E-02	1.197E-01	373.899	-0.222	3.125E-01	6.222E-01
ENSG00000112167	SAYSD1	89.720	-0.794	2.020E-02	1.202E-01	127.327	-0.094	7.343E-01	8.898E-01
ENSG00000070814	TCOF1	1437.394	0.485	2.029E-02	1.207E-01	819.748	0.693	4.718E-04	1.356E-02
ENSG00000160208	RRP1B	586.221	-0.506	2.034E-02	1.209E-01	653.661	0.216	3.001E-01	6.103E-01
ENSG00000224331	AC019181.1	33.305	-1.071	2.045E-02	1.213E-01	29.291	-0.551	1.877E-01	4.870E-01
ENSG00000115970	THADA	203.513	-0.653	2.047E-02	1.214E-01	186.704	0.112	6.717E-01	8.586E-01
ENSG00000261061	AC092718.4	186.022	-0.716	2.054E-02	1.218E-01	170.447	-0.002	9.946E-01	9.981E-01
ENSG00000111665	CDCA3	241.266	-0.610	2.055E-02	1.218E-01	216.060	-0.266	3.120E-01	6.220E-01
ENSG00000006715	VPS41	147.918	-0.699	2.067E-02	1.224E-01	212.698	-0.427	7.983E-02	3.123E-01
ENSG00000153363	LINC00467	39.495	-0.999	2.070E-02	1.225E-01	73.453	-0.338	3.009E-01	6.113E-01
ENSG00000068654	POLR1A	276.752	-0.675	2.083E-02	1.230E-01	251.565	-0.351	1.803E-01	4.780E-01
ENSG00000121957	GPSM2	46.900	-0.960	2.097E-02	1.236E-01	87.253	-0.317	3.340E-01	6.414E-01
ENSG00000116127	ALMS1	122.635	-0.751	2.115E-02	1.244E-01	151.850	-0.057	8.294E-01	9.324E-01
ENSG00000196975	ANXA4	517.662	-0.785	2.135E-02	1.253E-01	433.661	-0.183	4.335E-01	7.186E-01

ENSG00000175387	SMAD2	300.490	-0.556	2.157E-02	1.262E-01	364.819	-0.088	6.762E-01	8.607E-01
ENSG00000169856	ONECUT1	35.486	-1.135	2.178E-02	1.271E-01	48.983	-0.442	2.565E-01	5.668E-01
ENSG00000240225	ZNF542P	85.678	-1.001	2.188E-02	1.274E-01	55.084	0.052	8.835E-01	9.551E-01
ENSG00000174516	PELI3	34.699	-1.054	2.194E-02	1.277E-01	43.071	-0.429	2.404E-01	5.476E-01
ENSG00000158792	SPATA2L	40.148	-1.027	2.196E-02	1.277E-01	33.859	-0.279	5.020E-01	7.624E-01
ENSG00000186715	MST1L	178.684	-0.765	2.199E-02	1.279E-01	158.317	-0.072	8.306E-01	9.330E-01
ENSG00000163781	TOPBP1	275.309	-0.638	2.203E-02	1.279E-01	255.961	-0.282	2.190E-01	5.243E-01
ENSG00000118600	RXYLT1	36.353	-1.160	2.213E-02	1.283E-01	93.004	0.049	8.835E-01	9.551E-01
ENSG00000010818	HIVEP2	29.994	1.109	2.211E-02	1.283E-01	69.661	1.177	1.315E-03	2.614E-02
ENSG00000211445	GPX3	226.564	-0.598	2.239E-02	1.292E-01	167.055	-0.405	1.934E-01	4.942E-01
ENSG00000196236	XPNPEP3	108.631	-0.837	2.259E-02	1.301E-01	120.399	-0.409	2.001E-01	5.023E-01
ENSG00000113384	GOLPH3	523.119	-0.506	2.261E-02	1.301E-01	447.668	0.133	5.637E-01	8.011E-01
ENSG00000137944	KYAT3	103.792	-0.767	2.268E-02	1.302E-01	116.126	-0.131	6.549E-01	8.510E-01
ENSG00000249550	LINC01234	33.150	-1.035	2.270E-02	1.303E-01	37.065	-0.203	5.942E-01	8.187E-01
ENSG00000108219	TSPAN14	367.015	-0.642	2.270E-02	1.303E-01	183.089	-0.247	3.381E-01	6.463E-01
ENSG00000135916	ITM2C	112.056	-0.929	2.278E-02	1.306E-01	127.646	-0.119	6.726E-01	8.593E-01
ENSG00000106133	NSUN5P2	48.838	-0.930	2.281E-02	1.307E-01	41.703	-0.306	4.396E-01	7.218E-01
ENSG00000131504	DIAPH1	652.517	-0.559	2.284E-02	1.307E-01	773.080	-0.457	1.674E-02	1.326E-01
ENSG00000198792	TMEM184B	144.129	-0.761	2.294E-02	1.310E-01	105.623	-0.015	9.609E-01	9.844E-01
ENSG00000159063	ALG8	316.832	-0.596	2.302E-02	1.313E-01	323.357	-0.134	5.784E-01	8.093E-01
ENSG00000213463	SYNJ2BP	159.107	-0.722	2.312E-02	1.317E-01	186.556	-0.409	1.273E-01	4.028E-01
ENSG00000127603	MACF1	867.038	-0.502	2.313E-02	1.317E-01	993.793	-0.339	6.379E-02	2.774E-01
ENSG00000253797	UTP14C	135.588	-0.832	2.326E-02	1.321E-01	99.178	-0.079	8.070E-01	9.231E-01
ENSG00000088812	ATRN	276.386	-0.620	2.337E-02	1.325E-01	205.358	-0.097	7.364E-01	8.906E-01
ENSG00000148396	SEC16A	225.600	-0.678	2.345E-02	1.328E-01	281.182	-0.409	1.477E-01	4.344E-01
ENSG00000133740	E2F5	49.109	-1.072	2.351E-02	1.330E-01	45.216	-0.391	3.322E-01	6.399E-01

ENSG00000206052	DOK6	205.481	-0.625	2.354E-02	1.330E-01	209.190	0.362	1.355E-01	4.159E-01
ENSG00000120159	CAAP1	136.826	-0.728	2.364E-02	1.333E-01	139.752	-0.341	2.275E-01	5.327E-01
ENSG00000136450	SRSF1	1336.232	-1.043	2.372E-02	1.336E-01	888.961	-0.007	9.783E-01	9.912E-01
ENSG00000027847	B4GALT7	170.920	-0.720	2.398E-02	1.344E-01	126.860	-0.368	2.385E-01	5.456E-01
ENSG00000102100	SLC35A2	267.569	-0.586	2.393E-02	1.344E-01	222.031	0.378	1.294E-01	4.057E-01
ENSG00000167395	ZNF646	62.304	-0.903	2.417E-02	1.351E-01	55.449	-0.415	2.387E-01	5.459E-01
ENSG00000136237	RAPGEF5	146.935	-0.752	2.420E-02	1.351E-01	129.884	0.032	9.171E-01	9.692E-01
ENSG00000151715	TMEM45B	160.570	-0.742	2.423E-02	1.351E-01	147.861	-0.322	2.512E-01	5.602E-01
ENSG00000123472	ATPAF1	226.778	-0.572	2.419E-02	1.351E-01	189.037	-0.287	2.869E-01	5.979E-01
ENSG00000135775	COG2	95.817	-0.785	2.439E-02	1.359E-01	120.828	-0.158	6.026E-01	8.240E-01
ENSG00000125520	SLC2A4RG	1335.514	-0.506	2.455E-02	1.366E-01	1602.466	-0.172	3.389E-01	6.469E-01
ENSG00000112984	KIF20A	280.539	-0.633	2.463E-02	1.370E-01	187.842	0.109	6.915E-01	8.684E-01
ENSG00000242193	CRYZL2P	51.777	-0.970	2.499E-02	1.383E-01	31.152	-0.119	7.701E-01	9.054E-01
ENSG00000198736	MSRB1	461.023	-0.517	2.503E-02	1.384E-01	356.325	-0.428	8.741E-02	3.279E-01
ENSG00000113594	LIFR	117.001	-0.776	2.541E-02	1.398E-01	121.070	-0.081	7.935E-01	9.176E-01
ENSG00000110042	DTX4	188.883	-0.618	2.541E-02	1.398E-01	153.341	-0.431	1.156E-01	3.813E-01
ENSG00000210191	MT-TL2	30.275	-1.163	2.547E-02	1.400E-01	27.070	-1.108	8.291E-03	8.718E-02
ENSG00000105707	HPN	179.251	-0.747	2.566E-02	1.409E-01	206.855	-0.375	1.721E-01	4.676E-01
ENSG00000170485	NPAS2	101.414	-0.902	2.597E-02	1.421E-01	95.896	-0.046	8.798E-01	9.538E-01
ENSG00000065029	ZNF76	103.390	-0.762	2.615E-02	1.426E-01	133.334	-0.268	4.088E-01	7.033E-01
ENSG00000130309	COLGALT1	822.642	-0.719	2.626E-02	1.430E-01	473.739	-0.468	2.608E-01	5.707E-01
ENSG00000090520	DNAJB11	797.146	0.489	2.642E-02	1.435E-01	514.260	0.613	3.032E-03	4.586E-02
ENSG00000172340	SUCLG2	263.049	-0.648	2.646E-02	1.436E-01	261.883	-0.045	8.523E-01	9.443E-01
ENSG00000115756	HPCAL1	899.310	0.471	2.660E-02	1.441E-01	687.986	0.598	1.241E-03	2.550E-02
ENSG00000226419	SLC16A1-AS1	106.327	-0.791	2.673E-02	1.446E-01	129.307	-0.124	6.599E-01	8.532E-01
ENSG00000108406	DHX40	111.703	-0.773	2.684E-02	1.450E-01	361.880	-0.442	5.130E-02	2.473E-01

ENSG00000106351	AGFG2	211.257	-0.663	2.691E-02	1.453E-01	187.829	-0.179	5.148E-01	7.709E-01
ENSG00000176658	MYO1D	121.317	-0.744	2.703E-02	1.457E-01	93.263	-0.217	4.740E-01	7.471E-01
ENSG00000168140	VASN	46.084	-1.021	2.715E-02	1.460E-01	26.781	-1.351	8.041E-04	1.952E-02
ENSG00000149792	MRPL49	419.118	-0.595	2.725E-02	1.462E-01	293.377	-0.071	7.775E-01	9.097E-01
ENSG00000103479	RBL2	148.921	-0.753	2.752E-02	1.473E-01	160.278	-0.344	2.147E-01	5.190E-01
ENSG00000204876	AC021218.1	46.279	-0.990	2.758E-02	1.475E-01	73.852	-0.370	3.208E-01	6.294E-01
ENSG00000237649	KIFC1	316.541	-0.554	2.762E-02	1.476E-01	267.084	-0.348	1.540E-01	4.423E-01
ENSG00000107798	LIPA	115.695	-0.772	2.773E-02	1.480E-01	124.906	-0.360	2.298E-01	5.353E-01
ENSG00000103111	MON1B	333.866	-0.625	2.782E-02	1.482E-01	240.707	-0.493	7.805E-02	3.073E-01
ENSG00000102038	SMARCA1	1528.007	-0.550	2.818E-02	1.496E-01	1513.059	-0.498	5.200E-03	6.550E-02
ENSG00000101337	TM9SF4	372.339	-0.616	2.822E-02	1.497E-01	234.379	0.123	6.413E-01	8.446E-01
ENSG00000080986	NDC80	278.367	-0.607	2.828E-02	1.499E-01	230.384	0.070	7.759E-01	9.088E-01
ENSG00000054654	SYNE2	208.340	-0.791	2.836E-02	1.500E-01	283.752	-0.424	6.535E-02	2.799E-01
ENSG00000168802	CHTF8	494.740	-0.574	2.859E-02	1.508E-01	490.433	-0.400	4.929E-02	2.414E-01
ENSG00000163558	PRKCI	126.319	-0.784	2.871E-02	1.509E-01	128.277	-0.097	7.435E-01	8.937E-01
ENSG00000100413	POLR3H	206.011	-0.730	2.870E-02	1.509E-01	175.408	-0.479	9.171E-02	3.362E-01
ENSG00000204580	DDR1	512.889	0.493	2.865E-02	1.509E-01	534.571	0.620	2.300E-03	3.798E-02
ENSG00000119318	RAD23B	1264.710	0.475	2.885E-02	1.512E-01	1263.475	0.504	1.998E-02	1.486E-01
ENSG00000149782	PLCB3	130.973	-0.706	2.938E-02	1.534E-01	110.185	-0.375	2.777E-01	5.882E-01
ENSG00000259494	MRPL46	485.217	-0.642	2.946E-02	1.535E-01	361.989	-0.137	5.161E-01	7.717E-01
ENSG00000272931	AC099568.2	46.746	-0.897	2.986E-02	1.547E-01	46.147	0.455	2.447E-01	5.533E-01
ENSG00000010539	ZNF200	81.811	-0.747	3.002E-02	1.554E-01	59.872	-0.499	1.511E-01	4.384E-01
ENSG00000101049	SGK2	109.992	-0.771	3.014E-02	1.559E-01	89.124	0.055	8.550E-01	9.447E-01
ENSG00000215252	GOLGA8B	338.862	0.495	3.021E-02	1.562E-01	387.472	0.539	1.014E-02	9.810E-02
ENSG00000167258	CDK12	1058.424	-0.540	3.029E-02	1.562E-01	1044.457	-0.480	1.561E-02	1.278E-01
ENSG00000204310	AGPAT1	523.047	-0.511	3.044E-02	1.568E-01	594.974	-0.360	1.516E-01	4.393E-01

ENSG00000054277	OPN3	136.032	-0.702	3.054E-02	1.572E-01	140.960	-0.395	1.504E-01	4.377E-01
ENSG00000117834	SLC5A9	47.426	-0.903	3.059E-02	1.574E-01	92.880	-0.287	4.227E-01	7.122E-01
ENSG00000036257	CUL3	246.487	-0.642	3.071E-02	1.579E-01	209.546	-0.223	4.222E-01	7.120E-01
ENSG00000120137	PANK3	338.410	-0.566	3.084E-02	1.583E-01	168.458	0.049	8.633E-01	9.480E-01
ENSG00000099889	ARVCF	50.196	-0.983	3.086E-02	1.584E-01	61.344	-0.199	5.635E-01	8.011E-01
ENSG00000188486	H2AFX	438.154	-0.578	3.104E-02	1.590E-01	313.109	-0.057	8.146E-01	9.256E-01
ENSG00000130427	EPO	80.348	-0.820	3.114E-02	1.593E-01	25.166	-0.181	6.640E-01	8.547E-01
ENSG00000117139	KDM5B	221.617	-0.601	3.117E-02	1.594E-01	272.507	-0.395	7.472E-02	3.006E-01
ENSG00000129460	NGDN	1755.829	0.458	3.120E-02	1.595E-01	1269.339	1.014	4.634E-06	3.794E-04
ENSG00000130429	ARPC1B	453.048	-0.605	3.128E-02	1.596E-01	533.381	-0.363	7.892E-02	3.094E-01
ENSG00000162881	OXER1	30.351	-1.107	3.135E-02	1.599E-01	21.712	-0.576	1.689E-01	NA
ENSG00000100246	DNAL4	76.296	-0.809	3.175E-02	1.614E-01	76.198	-0.393	2.233E-01	5.282E-01
ENSG00000134461	ANKRD16	63.171	-0.820	3.178E-02	1.615E-01	63.093	0.256	4.733E-01	7.468E-01
ENSG00000126787	DLGAP5	281.895	-0.717	3.216E-02	1.628E-01	199.336	-0.221	3.666E-01	6.710E-01
ENSG00000113273	ARSB	142.279	-0.716	3.217E-02	1.628E-01	72.248	0.223	5.048E-01	7.642E-01
ENSG00000141424	SLC39A6	316.348	-0.553	3.263E-02	1.643E-01	309.029	-0.176	4.286E-01	7.158E-01
ENSG00000049883	PTCD2	46.858	-0.898	3.279E-02	1.648E-01	81.511	-0.051	8.767E-01	9.525E-01
ENSG00000137960	GIPC2	110.249	-0.740	3.291E-02	1.651E-01	148.562	-0.300	3.357E-01	6.435E-01
ENSG00000099330	OCEL1	72.931	-0.832	3.295E-02	1.652E-01	83.517	-0.441	2.238E-01	5.285E-01
ENSG00000080839	RBL1	139.291	-0.671	3.308E-02	1.658E-01	195.646	-0.290	3.088E-01	6.185E-01
ENSG00000152413	HOMER1	249.136	-0.607	3.328E-02	1.666E-01	218.032	0.005	9.823E-01	9.925E-01
ENSG00000182158	CREB3L2	379.321	-0.508	3.341E-02	1.669E-01	500.170	-0.315	1.342E-01	4.137E-01
ENSG00000272565	RP11-485G4.2	44.594	-0.913	3.356E-02	1.675E-01	27.438	-0.664	1.139E-01	3.779E-01
ENSG00000175595	ERCC4	113.704	-0.679	3.369E-02	1.679E-01	155.209	-0.099	7.050E-01	8.754E-01
ENSG00000103404	USP31	390.619	0.498	3.416E-02	1.695E-01	300.423	0.513	5.304E-02	2.519E-01
ENSG00000109066	TMEM104	103.860	-0.727	3.425E-02	1.699E-01	75.184	-0.229	5.083E-01	7.666E-01

ENSG00000257093	KIAA1147	72.897	-0.825	3.428E-02	1.700E-01	65.079	-0.163	6.581E-01	8.528E-01
ENSG00000273247	AC097376.2	69.285	-0.788	3.434E-02	1.700E-01	63.031	-0.123	7.254E-01	8.861E-01
ENSG00000104131	EIF3J	1730.056	0.440	3.468E-02	1.712E-01	1941.939	0.593	1.413E-03	2.741E-02
ENSG00000003400	CASP10	94.807	-0.727	3.473E-02	1.713E-01	94.470	0.131	7.001E-01	8.731E-01
ENSG00000108819	PPP1R9B	124.973	-0.671	3.481E-02	1.715E-01	115.543	0.455	1.759E-01	4.715E-01
ENSG00000228288	PCAT6	20.936	1.109	3.484E-02	1.716E-01	54.037	0.741	6.184E-02	2.734E-01
ENSG00000064225	ST3GAL6	142.416	-0.651	3.486E-02	1.716E-01	72.231	0.001	9.977E-01	9.994E-01
ENSG00000230269	LINC02525	48.749	-0.874	3.489E-02	1.717E-01	85.093	-0.037	9.217E-01	9.712E-01
ENSG00000169895	SYAP1	684.491	-0.542	3.492E-02	1.717E-01	853.888	-0.446	1.014E-02	9.810E-02
ENSG00000155438	NIFK	718.715	0.448	3.496E-02	1.718E-01	594.046	0.591	4.948E-03	6.328E-02
ENSG00000121073	SLC35B1	587.745	0.471	3.522E-02	1.728E-01	570.245	0.766	2.679E-04	9.059E-03
ENSG00000178028	DMAP1	83.913	-0.760	3.541E-02	1.734E-01	47.619	-0.168	6.567E-01	8.519E-01
ENSG00000111859	NEDD9	540.882	-0.498	3.541E-02	1.734E-01	420.914	0.510	3.093E-02	1.875E-01
ENSG00000173120	KDM2A	504.386	-0.530	3.548E-02	1.736E-01	542.275	-0.095	6.688E-01	8.574E-01
ENSG00000146833	TRIM4	165.939	-0.729	3.550E-02	1.736E-01	251.860	-0.397	9.551E-02	3.430E-01
ENSG00000204054	LINC00963	79.332	-0.813	3.565E-02	1.738E-01	114.626	-0.205	5.403E-01	7.878E-01
ENSG00000151849	CENPJ	194.696	-0.696	3.606E-02	1.751E-01	211.394	0.071	7.752E-01	9.084E-01
ENSG00000145604	SKP2	612.919	-0.567	3.626E-02	1.759E-01	384.760	-0.050	8.460E-01	9.409E-01
ENSG00000131899	LLGL1	113.175	-0.831	3.635E-02	1.762E-01	100.973	0.325	4.075E-01	7.026E-01
ENSG00000272821	U62317.3	93.546	-0.894	3.645E-02	1.762E-01	46.699	-0.026	9.489E-01	9.810E-01
ENSG00000108984	MAP2K6	92.252	-0.720	3.668E-02	1.769E-01	72.540	-0.141	6.691E-01	8.575E-01
ENSG00000224189	HAGLR	36.470	-0.965	3.670E-02	1.769E-01	28.379	0.566	1.567E-01	4.470E-01
ENSG00000081087	OSTM1	50.297	-0.878	3.697E-02	1.775E-01	64.782	-0.050	8.890E-01	9.580E-01
ENSG00000100104	SRRD	106.149	-0.690	3.700E-02	1.775E-01	114.801	-0.208	4.768E-01	7.494E-01
ENSG00000111364	DDX55	372.665	0.494	3.724E-02	1.783E-01	281.289	0.518	6.557E-02	2.805E-01
ENSG00000129933	MAU2	192.527	-0.571	3.780E-02	1.805E-01	216.695	-0.456	1.059E-01	3.634E-01

ENSG00000161904	LEMD2	266.353	-0.591	3.810E-02	1.812E-01	255.799	0.023	9.305E-01	9.750E-01
ENSG00000170144	HNRNPA3	1131.489	-0.566	3.831E-02	1.819E-01	821.108	0.100	6.542E-01	8.507E-01
ENSG00000063601	MTMR1	64.029	-0.840	3.899E-02	1.840E-01	56.569	0.270	4.594E-01	7.377E-01
ENSG00000205808	PLPP6	61.405	-0.871	3.904E-02	1.841E-01	52.386	-0.212	5.483E-01	7.916E-01
ENSG00000115042	FAHD2A	48.885	-0.890	3.912E-02	1.845E-01	45.726	-0.479	2.184E-01	5.236E-01
ENSG00000135476	ESPL1	43.196	-1.031	3.928E-02	1.849E-01	37.686	-0.053	8.905E-01	9.586E-01
ENSG00000189114	BLOC1S3	63.931	-0.792	3.927E-02	1.849E-01	68.006	-0.394	2.728E-01	5.826E-01
ENSG00000214022	REPIN1	172.953	-0.794	3.931E-02	1.849E-01	288.768	-0.167	4.854E-01	7.530E-01
ENSG00000140263	SORD	97.571	-0.714	3.948E-02	1.855E-01	102.245	0.007	9.811E-01	9.920E-01
ENSG00000114739	ACVR2B	151.041	-0.613	3.969E-02	1.860E-01	95.446	-0.061	8.476E-01	9.419E-01
ENSG00000066135	KDM4A	120.897	-0.741	3.974E-02	1.861E-01	225.290	-0.398	1.023E-01	3.584E-01
ENSG00000144451	SPAG16	222.786	-0.543	3.995E-02	1.870E-01	312.643	0.140	5.723E-01	8.061E-01
ENSG00000126522	ASL	30.115	-1.014	3.997E-02	1.870E-01	30.220	-0.173	6.699E-01	8.578E-01
ENSG00000107669	ATE1	188.248	-0.572	4.034E-02	1.879E-01	209.438	-0.066	7.970E-01	9.188E-01
ENSG00000138286	FAM149B1	44.268	-0.893	4.047E-02	1.884E-01	42.194	-0.148	7.024E-01	8.743E-01
ENSG00000139438	FAM222A	50.093	-0.934	4.052E-02	1.885E-01	67.629	0.317	3.509E-01	6.569E-01
ENSG00000248527	MTATP6P1	4094.505	0.428	4.067E-02	1.889E-01	3337.800	0.940	7.348E-07	9.188E-05
ENSG00000164087	POC1A	390.979	-0.536	4.097E-02	1.897E-01	343.700	-0.314	2.005E-01	5.028E-01
ENSG00000122912	SLC25A16	60.055	-0.836	4.100E-02	1.897E-01	60.263	0.030	9.306E-01	9.750E-01
ENSG00000160447	PKN3	103.050	-0.855	4.112E-02	1.901E-01	88.312	-0.414	2.371E-01	5.436E-01
ENSG00000204130	RUFY2	80.532	-0.722	4.112E-02	1.901E-01	93.059	-0.159	6.408E-01	8.446E-01
ENSG00000144959	NCEH1	86.716	-0.724	4.126E-02	1.907E-01	50.375	-0.376	2.950E-01	6.049E-01
ENSG00000163904	SENP2	144.654	-0.621	4.137E-02	1.909E-01	139.765	-0.253	3.721E-01	6.755E-01
ENSG00000136897	MRPL50	331.241	-0.563	4.150E-02	1.911E-01	272.201	-0.097	6.740E-01	8.595E-01
ENSG00000180938	ZNF572	51.682	-0.832	4.152E-02	1.911E-01	29.141	-0.433	2.862E-01	5.975E-01
ENSG00000101447	FAM83D	443.795	-0.574	4.158E-02	1.913E-01	379.337	0.166	4.172E-01	7.083E-01

ENSG00000144647	POMGNT2	191.457	-0.558	4.174E-02	1.917E-01	167.995	0.315	2.454E-01	5.537E-01
ENSG00000182022	CHST15	62.194	-0.867	4.203E-02	1.923E-01	44.248	-0.473	2.492E-01	5.582E-01
ENSG00000162174	ASRGL1	970.931	-0.629	4.205E-02	1.924E-01	747.850	-0.274	1.571E-01	4.473E-01
ENSG00000169925	BRD3	725.221	-0.505	4.220E-02	1.928E-01	890.919	-0.349	8.842E-02	3.299E-01
ENSG00000180178	FAR2P1	30.115	-0.941	4.248E-02	1.938E-01	41.766	-0.122	7.572E-01	9.007E-01
ENSG00000089048	ESF1	2028.125	0.448	4.276E-02	1.946E-01	1760.922	0.547	4.372E-03	5.889E-02
ENSG00000107815	TWNK	172.917	-0.621	4.288E-02	1.949E-01	163.879	-0.250	3.759E-01	6.772E-01
ENSG00000125249	RAP2A	190.463	-0.785	4.305E-02	1.955E-01	156.414	0.059	8.472E-01	9.416E-01
ENSG00000138777	PPA2	111.150	-0.686	4.318E-02	1.958E-01	110.068	-0.333	2.560E-01	5.663E-01
ENSG00000198814	GK	46.450	-0.880	4.335E-02	1.961E-01	15.161	-0.024	9.522E-01	NA
ENSG00000230551	AC021078.1	52.302	-0.907	4.337E-02	1.961E-01	72.900	-0.003	9.921E-01	9.967E-01
ENSG00000165389	SPTSSA	543.389	-0.602	4.381E-02	1.973E-01	478.381	-0.079	7.385E-01	8.914E-01
ENSG00000130202	NECTIN2	247.502	-0.629	4.414E-02	1.980E-01	201.756	-0.381	2.162E-01	5.213E-01
ENSG00000100485	SOS2	83.446	-0.705	4.418E-02	1.981E-01	74.529	0.346	2.970E-01	6.071E-01
ENSG00000163626	COX18	94.854	-0.778	4.437E-02	1.986E-01	90.015	0.106	7.589E-01	9.012E-01
ENSG00000075790	BCAP29	192.154	-0.591	4.448E-02	1.989E-01	191.687	-0.305	2.320E-01	5.375E-01
ENSG00000259959	AC107068.1	30.246	-1.034	4.490E-02	2.002E-01	25.940	-0.092	8.265E-01	9.312E-01
ENSG00000095787	WAC	1379.584	0.383	4.509E-02	2.007E-01	1431.072	0.531	2.105E-03	3.582E-02
ENSG00000134698	AGO4	39.019	-0.943	4.533E-02	2.015E-01	53.447	0.215	5.611E-01	7.995E-01
ENSG00000187688	TRPV2	30.183	-0.956	4.553E-02	2.022E-01	21.543	-0.469	2.584E-01	NA
ENSG00000086015	MAST2	139.783	-0.617	4.566E-02	2.026E-01	187.542	-0.197	4.336E-01	7.186E-01
ENSG00000122966	CIT	80.196	-0.816	4.574E-02	2.027E-01	94.666	-0.243	4.773E-01	7.494E-01
ENSG00000138796	HADH	291.658	-0.603	4.585E-02	2.030E-01	251.082	-0.200	4.454E-01	7.253E-01
ENSG00000074201	CLNS1A	759.644	-0.540	4.592E-02	2.031E-01	870.539	-0.038	8.328E-01	9.345E-01
ENSG00000135677	GNS	202.373	-0.547	4.596E-02	2.033E-01	228.947	-0.493	5.178E-02	2.484E-01
ENSG00000117528	ABCD3	632.777	-0.502	4.618E-02	2.039E-01	519.969	-0.214	3.794E-01	6.799E-01

ENSG00000132004	FBXW9	49.825	-0.810	4.623E-02	2.040E-01	110.767	-0.237	4.549E-01	7.339E-01
ENSG00000100304	TTLL12	115.200	-0.671	4.660E-02	2.050E-01	71.306	0.072	8.334E-01	9.345E-01
ENSG00000042088	TDP1	121.945	-0.661	4.660E-02	2.050E-01	101.131	-0.187	5.487E-01	7.916E-01
ENSG00000168175	MAPK1IP1L	308.036	-0.550	4.662E-02	2.050E-01	291.816	-0.233	3.426E-01	6.491E-01
ENSG00000109466	KLHL2	44.964	-0.876	4.675E-02	2.051E-01	27.568	0.025	9.515E-01	9.821E-01
ENSG00000107186	MPDZ	112.237	-0.721	4.675E-02	2.051E-01	105.106	-0.124	6.835E-01	8.644E-01
ENSG00000163006	CCDC138	63.040	-0.771	4.689E-02	2.055E-01	48.317	0.073	8.548E-01	9.447E-01
ENSG00000149483	TMEM138	174.487	-0.570	4.738E-02	2.073E-01	144.994	-0.162	5.955E-01	8.195E-01
ENSG00000132846	ZBED3	162.884	-0.623	4.758E-02	2.081E-01	146.107	-0.214	4.428E-01	7.231E-01
ENSG00000089063	TMEM230	469.064	-0.693	4.829E-02	2.102E-01	543.940	0.065	7.413E-01	8.925E-01
ENSG00000136848	DAB2IP	154.297	-0.666	4.851E-02	2.102E-01	146.635	-0.280	3.495E-01	6.555E-01
ENSG00000272047	GTF2H5	631.688	-0.608	4.864E-02	2.106E-01	619.188	-0.069	7.453E-01	8.939E-01
ENSG00000157426	AASDH	79.773	-0.817	4.875E-02	2.109E-01	60.808	-0.348	3.450E-01	6.514E-01
ENSG00000007968	E2F2	42.137	-0.890	4.925E-02	2.127E-01	49.943	-0.122	7.394E-01	8.920E-01
ENSG00000117054	ACADM	251.138	-0.588	4.941E-02	2.130E-01	348.211	-0.416	6.121E-02	2.725E-01
ENSG00000275052	PPP4R3B	295.434	-0.508	4.945E-02	2.130E-01	335.841	-0.068	8.037E-01	9.218E-01
ENSG00000197128	ZNF772	70.485	-0.773	4.993E-02	2.143E-01	57.930	-0.324	3.690E-01	6.728E-01
ENSG00000122483	CCDC18	151.996	-0.627	4.987E-02	2.143E-01	141.719	-0.089	7.586E-01	9.010E-01
ENSG00000166130	IKBIP	233.414	-0.512	4.990E-02	2.143E-01	234.042	-0.071	8.101E-01	9.241E-01
ENSG00000148935	GAS2	72.993	-0.835	5.007E-02	2.145E-01	72.508	-0.155	6.377E-01	8.430E-01
ENSG00000161395	PGAP3	44.169	-0.824	5.031E-02	2.152E-01	38.770	-0.479	2.438E-01	5.521E-01
ENSG00000146281	PM20D2	148.845	-0.627	5.059E-02	2.160E-01	156.104	-0.088	7.546E-01	8.996E-01
ENSG00000204623	ZNRD1ASP	44.800	-0.831	5.080E-02	2.166E-01	55.138	-0.198	6.051E-01	8.257E-01
ENSG00000131375	CAPN7	138.846	-0.622	5.099E-02	2.170E-01	188.109	-0.006	9.824E-01	9.925E-01
ENSG00000010072	SPRTN	60.876	-0.834	5.104E-02	2.171E-01	71.909	0.042	9.028E-01	9.639E-01
ENSG00000100031	GGT1	32.407	-0.934	5.133E-02	2.179E-01	30.159	-0.032	9.393E-01	9.777E-01

ENSG00000172936	MYD88	116.903	-0.812	5.135E-02	2.179E-01	60.181	-0.040	9.092E-01	9.671E-01
ENSG00000146729	NIPSNAP2	145.021	-0.623	5.194E-02	2.194E-01	242.062	-0.379	1.488E-01	4.364E-01
ENSG00000188206	HNRNPU-AS1	64.714	-0.742	5.230E-02	2.203E-01	65.624	-0.448	1.844E-01	4.832E-01
ENSG00000124920	MYRF	185.935	-0.617	5.237E-02	2.203E-01	148.255	0.085	8.351E-01	9.350E-01
ENSG00000162441	LZIC	307.609	-0.503	5.232E-02	2.203E-01	291.288	-0.139	5.820E-01	8.119E-01
ENSG00000119401	TRIM32	45.740	-0.847	5.259E-02	2.205E-01	60.366	-0.103	7.885E-01	9.151E-01
ENSG00000171469	ZNF561	60.142	-0.793	5.269E-02	2.208E-01	69.356	-0.270	4.493E-01	7.280E-01
ENSG00000165898	ISCA2	214.789	-0.691	5.277E-02	2.208E-01	178.471	-0.015	9.557E-01	9.838E-01
ENSG00000177485	ZBTB33	196.782	-0.560	5.276E-02	2.208E-01	191.770	-0.065	8.034E-01	9.216E-01
ENSG00000184465	WDR27	90.927	-0.714	5.301E-02	2.210E-01	137.584	0.025	9.320E-01	9.753E-01
ENSG00000065328	MCM10	310.083	-0.507	5.298E-02	2.210E-01	303.202	0.187	4.817E-01	7.517E-01
ENSG00000151247	EIF4E	434.267	0.441	5.291E-02	2.210E-01	334.894	0.750	5.007E-03	6.384E-02
ENSG00000168291	PDHB	294.457	-0.568	5.331E-02	2.219E-01	215.632	0.152	5.531E-01	7.942E-01
ENSG00000068784	SRBD1	163.726	-0.583	5.339E-02	2.221E-01	192.610	0.003	9.899E-01	9.961E-01
ENSG00000157985	AGAP1	220.305	-0.583	5.344E-02	2.223E-01	282.852	0.270	2.290E-01	5.343E-01
ENSG00000037757	MRI1	112.448	-0.598	5.355E-02	2.226E-01	104.816	-0.056	8.667E-01	9.495E-01
ENSG00000015532	XYLT2	130.647	-0.592	5.376E-02	2.230E-01	66.791	-0.477	1.510E-01	4.384E-01
ENSG00000182749	PAQR7	82.378	-0.754	5.398E-02	2.236E-01	82.772	0.339	3.836E-01	6.829E-01
ENSG00000112159	MDN1	659.912	-0.514	5.420E-02	2.240E-01	566.695	-0.005	9.791E-01	9.914E-01
ENSG00000071073	MGAT4A	302.930	-0.510	5.420E-02	2.240E-01	204.870	-0.296	2.723E-01	5.824E-01
ENSG00000117133	RPF1	463.581	0.447	5.419E-02	2.240E-01	371.309	0.557	1.372E-02	1.177E-01
ENSG00000135686	KLHL36	227.385	-0.541	5.426E-02	2.242E-01	257.652	-0.296	2.221E-01	5.272E-01
ENSG00000096092	TMEM14A	131.726	-0.635	5.471E-02	2.256E-01	345.553	-0.104	6.316E-01	8.402E-01
ENSG00000080815	PSEN1	162.210	-0.580	5.501E-02	2.265E-01	199.060	-0.165	5.033E-01	7.636E-01
ENSG00000124615	MOCS1	102.026	-0.634	5.541E-02	2.274E-01	58.520	-0.173	6.478E-01	8.475E-01
ENSG00000269343	ZNF587B	74.666	-0.706	5.554E-02	2.276E-01	58.619	-0.165	6.423E-01	8.449E-01

ENSG00000068650	ATP11A	175.478	-0.547	5.555E-02	2.276E-01	180.221	-0.354	1.950E-01	4.960E-01
ENSG00000111203	ITFG2	70.681	-0.825	5.603E-02	2.286E-01	66.964	-0.028	9.353E-01	9.762E-01
ENSG00000106392	C1GALT1	299.665	-0.524	5.594E-02	2.286E-01	297.705	-0.300	2.278E-01	5.330E-01
ENSG00000114942	EEF1B2	4828.945	0.380	5.598E-02	2.286E-01	3394.411	0.507	2.481E-03	3.980E-02
ENSG00000121058	COIL	482.991	0.479	5.605E-02	2.286E-01	396.092	0.600	1.233E-02	1.106E-01
ENSG00000138346	DNA2	40.981	-0.842	5.621E-02	2.288E-01	33.157	-0.165	6.937E-01	8.687E-01
ENSG00000126767	ELK1	96.170	-0.734	5.617E-02	2.288E-01	80.929	0.226	4.762E-01	7.490E-01
ENSG00000176974	SHMT1	176.923	-0.695	5.645E-02	2.295E-01	145.094	0.094	7.290E-01	8.880E-01
ENSG00000088035	ALG6	35.830	-0.883	5.663E-02	2.301E-01	38.229	0.154	7.022E-01	8.743E-01
ENSG00000156239	N6AMT1	44.674	-0.833	5.678E-02	2.305E-01	54.877	-0.240	4.951E-01	7.577E-01
ENSG00000117308	GALE	214.531	-0.520	5.704E-02	2.312E-01	214.559	-0.064	7.997E-01	9.193E-01
ENSG00000007923	DNAJC11	197.951	-0.549	5.723E-02	2.317E-01	142.369	-0.317	2.753E-01	5.857E-01
ENSG00000149196	HIKESHI	197.740	-0.647	5.748E-02	2.322E-01	205.506	0.115	6.923E-01	8.684E-01
ENSG00000182095	TNRC18	82.640	-0.673	5.755E-02	2.323E-01	67.058	-0.223	5.496E-01	7.918E-01
ENSG00000078902	TOLLIP	703.443	0.461	5.762E-02	2.325E-01	590.890	0.516	3.273E-02	1.932E-01
ENSG00000092201	SUPT16H	2420.890	-0.505	5.774E-02	2.328E-01	2113.182	-0.429	2.495E-02	1.668E-01
ENSG00000205476	CCDC85C	287.829	-0.526	5.791E-02	2.333E-01	183.709	-0.294	2.471E-01	5.552E-01
ENSG00000169105	CHST14	77.412	-0.764	5.813E-02	2.338E-01	68.229	-0.124	7.390E-01	8.916E-01
ENSG00000185219	ZNF445	150.724	-0.541	5.834E-02	2.342E-01	144.674	-0.471	1.259E-01	4.004E-01
ENSG00000263731	AC145207.5	46.724	-0.880	5.853E-02	2.345E-01	26.736	-0.142	7.271E-01	8.866E-01
ENSG00000142188	TMEM50B	113.954	-0.605	5.854E-02	2.345E-01	160.059	-0.148	5.664E-01	8.023E-01
ENSG00000139865	TTC6	31.494	-0.880	5.897E-02	2.357E-01	39.098	-0.453	2.518E-01	5.609E-01
ENSG00000099290	WASHC2A	91.855	-0.641	5.932E-02	2.365E-01	97.645	-0.418	2.091E-01	5.121E-01
ENSG00000143157	POGK	59.604	-0.758	5.935E-02	2.365E-01	104.986	-0.431	1.412E-01	4.250E-01
ENSG00000171097	KYAT1	59.950	-0.742	5.936E-02	2.365E-01	52.856	-0.271	4.594E-01	7.377E-01
ENSG00000164403	SHROOM1	233.877	-0.545	5.938E-02	2.365E-01	185.291	-0.150	6.107E-01	8.288E-01

ENSG00000213066	FGFR1OP	215.395	-0.562	5.963E-02	2.370E-01	210.901	0.370	1.707E-01	4.656E-01
ENSG00000160221	C21orf33	32.913	-0.933	5.972E-02	2.373E-01	26.094	-0.580	1.539E-01	4.423E-01
ENSG00000180098	TRNAU1AP	77.766	-0.767	5.993E-02	2.377E-01	89.480	-0.361	2.547E-01	5.650E-01
ENSG00000141034	GID4	71.420	-0.691	6.020E-02	2.382E-01	31.093	0.258	5.323E-01	7.843E-01
ENSG00000106080	FKBP14	167.802	-0.594	6.070E-02	2.394E-01	151.061	-0.174	5.450E-01	7.902E-01
ENSG00000104765	BNIP3L	156.319	-0.584	6.077E-02	2.396E-01	122.637	-0.009	9.759E-01	9.909E-01
ENSG00000145439	CBR4	222.057	-0.509	6.101E-02	2.404E-01	182.448	0.114	6.642E-01	8.548E-01
ENSG00000165424	ZCCHC24	51.073	-0.816	6.114E-02	2.408E-01	37.393	-0.309	4.485E-01	7.276E-01
ENSG00000179889	PDXDC1	692.246	-0.561	6.201E-02	2.430E-01	610.650	0.278	2.556E-01	5.661E-01
ENSG00000155592	ZKSCAN2	84.386	-0.697	6.217E-02	2.432E-01	55.192	-0.136	7.187E-01	8.828E-01
ENSG00000146463	ZMYM4	211.408	-0.529	6.320E-02	2.460E-01	243.729	-0.399	1.309E-01	4.084E-01
ENSG00000075539	FRYL	92.292	-0.633	6.326E-02	2.461E-01	135.461	-0.323	2.580E-01	5.685E-01
ENSG00000124160	NCOA5	160.368	-0.626	6.366E-02	2.471E-01	136.879	-0.109	7.506E-01	8.968E-01
ENSG00000150527	CTAGE5	468.246	0.474	6.398E-02	2.479E-01	431.628	0.669	1.234E-02	1.106E-01
ENSG00000167283	ATP5MG	7099.291	0.378	6.448E-02	2.493E-01	5580.421	0.547	1.033E-03	2.317E-02
ENSG00000009724	MASP2	32.018	-0.904	6.455E-02	2.494E-01	44.818	-0.489	2.436E-01	5.521E-01
ENSG00000120158	RCL1	487.459	0.467	6.460E-02	2.495E-01	409.164	0.771	6.036E-04	1.584E-02
ENSG00000168461	RAB31	151.347	-0.544	6.479E-02	2.498E-01	154.031	-0.438	1.382E-01	4.200E-01
ENSG00000064787	BCAS1	192.884	-0.535	6.479E-02	2.498E-01	230.978	-0.465	4.771E-02	2.375E-01
ENSG00000166813	KIF7	88.682	-0.754	6.641E-02	2.546E-01	78.153	0.167	5.999E-01	8.219E-01
ENSG00000062725	APPBP2	152.690	-0.672	6.648E-02	2.547E-01	209.086	-0.253	3.595E-01	6.649E-01
ENSG00000180953	ST20	32.866	-0.908	6.688E-02	2.556E-01	32.939	0.607	1.228E-01	3.936E-01
ENSG00000169188	APEX2	126.691	-0.583	6.745E-02	2.569E-01	144.893	-0.320	2.696E-01	5.793E-01
ENSG00000115255	REEP6	88.326	-0.830	6.807E-02	2.583E-01	63.710	-0.472	2.328E-01	5.380E-01
ENSG00000155008	APOOL	120.790	-0.620	6.808E-02	2.583E-01	130.584	-0.422	1.595E-01	4.503E-01
ENSG00000163684	RPP14	90.012	-0.673	6.879E-02	2.601E-01	95.866	-0.131	6.688E-01	8.574E-01

ENSG00000138744	NAAA	101.844	-0.663	6.911E-02	2.610E-01	84.016	0.062	8.443E-01	9.400E-01
ENSG00000164967	RPP25L	86.603	-0.657	6.914E-02	2.610E-01	98.359	-0.185	5.651E-01	8.014E-01
ENSG00000070476	ZXDC	101.531	-0.611	6.945E-02	2.617E-01	97.900	-0.084	7.829E-01	9.120E-01
ENSG00000256771	ZNF253	37.047	-0.937	6.993E-02	2.627E-01	17.303	-0.990	1.830E-02	NA
ENSG00000119689	DLST	29.827	0.894	7.048E-02	2.640E-01	30.795	1.017	1.255E-02	1.123E-01
ENSG00000156958	GALK2	64.390	-0.761	7.056E-02	2.640E-01	46.969	-0.150	6.901E-01	8.680E-01
ENSG00000147180	ZNF711	16.163	0.962	7.086E-02	2.649E-01	37.174	0.768	4.966E-02	2.421E-01
ENSG00000101187	SLCO4A1	144.230	-0.573	7.125E-02	2.658E-01	81.647	-0.184	6.440E-01	8.457E-01
ENSG00000057252	SOAT1	155.286	-0.628	7.135E-02	2.659E-01	118.226	-0.239	3.931E-01	6.917E-01
ENSG00000160124	CCDC58	247.114	-0.528	7.135E-02	2.659E-01	226.878	0.335	2.225E-01	5.276E-01
ENSG00000125503	PPP1R12C	131.280	-0.553	7.185E-02	2.672E-01	139.741	-0.171	6.003E-01	8.220E-01
ENSG00000043093	DCUN1D1	59.637	-0.762	7.228E-02	2.680E-01	75.904	-0.212	5.043E-01	7.642E-01
ENSG00000116171	SCP2	222.885	-0.529	7.270E-02	2.692E-01	263.572	-0.182	4.486E-01	7.276E-01
ENSG00000125247	TMTC4	103.796	-0.610	7.316E-02	2.703E-01	165.630	-0.250	3.234E-01	6.311E-01
ENSG00000145703	IQGAP2	703.171	-0.532	7.345E-02	2.710E-01	743.693	-0.390	5.195E-02	2.487E-01
ENSG00000131469	RPL27	7974.476	0.382	7.390E-02	2.718E-01	5420.429	0.555	1.961E-02	1.472E-01
ENSG00000126705	AHDC1	99.141	-0.759	7.403E-02	2.722E-01	88.751	-0.330	2.948E-01	6.049E-01
ENSG00000023516	AKAP11	68.220	-0.690	7.407E-02	2.722E-01	116.496	-0.386	1.973E-01	4.986E-01
ENSG00000176444	CLK2	110.525	-0.599	7.415E-02	2.725E-01	124.874	-0.237	4.302E-01	7.167E-01
ENSG00000221829	FANCG	182.275	-0.520	7.425E-02	2.726E-01	154.020	-0.203	4.481E-01	7.274E-01
ENSG00000138385	SSB	2412.690	0.370	7.454E-02	2.731E-01	1985.321	0.526	7.726E-03	8.275E-02
ENSG00000173706	HEG1	146.590	-0.562	7.511E-02	2.745E-01	181.152	-0.265	3.042E-01	6.153E-01
ENSG00000143921	ABCG8	54.521	-0.815	7.554E-02	2.757E-01	37.328	-0.492	2.147E-01	5.190E-01
ENSG00000131981	LGALS3	117.562	-0.684	7.587E-02	2.764E-01	71.046	0.583	8.165E-02	3.159E-01
ENSG00000197256	KANK2	125.177	-0.564	7.661E-02	2.784E-01	125.324	-0.013	9.630E-01	9.847E-01
ENSG00000205659	LIN52	126.700	-0.563	7.679E-02	2.789E-01	173.426	0.088	7.326E-01	8.888E-01

ENSG00000247400	DNAJC3-AS1	79.691	-0.639	7.682E-02	2.790E-01	80.588	-0.487	1.960E-01	4.974E-01
ENSG00000114978	MOB1A	236.919	-0.580	7.749E-02	2.804E-01	210.580	-0.026	9.160E-01	9.692E-01
ENSG00000168386	FILIP1L	155.696	-0.559	7.837E-02	2.819E-01	217.165	-0.374	1.610E-01	4.515E-01
ENSG00000108506	INTS2	68.688	-0.654	7.897E-02	2.835E-01	70.816	-0.154	6.744E-01	8.600E-01
ENSG00000250799	PRODH2	43.835	-0.793	7.921E-02	2.837E-01	44.969	-0.406	2.977E-01	6.079E-01
ENSG00000103855	CD276	239.746	-0.525	7.925E-02	2.837E-01	237.928	0.093	7.347E-01	8.899E-01
ENSG00000186185	KIF18B	107.874	-0.715	7.935E-02	2.839E-01	56.199	0.297	4.604E-01	7.386E-01
ENSG00000070366	SMG6	117.836	-0.599	7.960E-02	2.846E-01	176.399	-0.242	3.891E-01	6.879E-01
ENSG00000189007	ADAT2	204.438	-0.511	7.971E-02	2.847E-01	221.093	0.143	6.337E-01	8.411E-01
ENSG00000171960	PPIH	479.957	0.455	8.064E-02	2.867E-01	394.820	0.630	4.111E-03	5.648E-02
ENSG00000069966	GNB5	62.388	-0.743	8.071E-02	2.869E-01	90.828	-0.278	3.822E-01	6.824E-01
ENSG00000130702	LAMA5	443.144	-0.545	8.090E-02	2.870E-01	474.096	-0.390	6.815E-02	2.858E-01
ENSG00000143156	NME7	37.176	-0.834	8.116E-02	2.873E-01	49.492	-0.316	4.008E-01	6.979E-01
ENSG00000137414	FAM8A1	167.688	-0.594	8.116E-02	2.873E-01	207.318	-0.259	2.816E-01	5.925E-01
ENSG00000145362	ANK2	48.750	-0.793	8.125E-02	2.875E-01	46.848	-0.464	2.604E-01	5.702E-01
ENSG00000058673	ZC3H11A	172.134	-0.523	8.172E-02	2.886E-01	203.026	-0.250	3.057E-01	6.156E-01
ENSG00000158636	EMSY	69.617	-0.646	8.197E-02	2.890E-01	74.881	-0.067	8.374E-01	9.360E-01
ENSG00000096872	IFT74	57.852	-0.717	8.239E-02	2.898E-01	114.718	0.013	9.636E-01	9.849E-01
ENSG00000128191	DGCR8	117.661	-0.655	8.304E-02	2.913E-01	124.058	-0.127	6.908E-01	8.681E-01
ENSG00000008283	CYB561	38.992	-0.812	8.331E-02	2.917E-01	28.461	-0.495	2.226E-01	5.276E-01
ENSG00000172292	CERS6	282.503	-0.521	8.379E-02	2.928E-01	282.501	-0.243	3.184E-01	6.282E-01
ENSG00000103037	SETD6	158.763	-0.575	8.390E-02	2.929E-01	165.022	0.028	9.171E-01	9.692E-01
ENSG00000079387	SENP1	161.736	-0.583	8.416E-02	2.936E-01	145.675	-0.466	1.028E-01	3.592E-01
ENSG00000137996	RTCA	216.206	0.472	8.462E-02	2.949E-01	220.776	0.719	6.636E-03	7.522E-02
ENSG00000069943	PIGB	47.034	-0.785	8.517E-02	2.960E-01	38.884	0.001	9.988E-01	9.997E-01
ENSG00000117724	CENPF	1457.618	-0.537	8.519E-02	2.960E-01	2255.375	-0.308	8.203E-02	3.165E-01

ENSG00000198888	MT-ND1	38191.147	0.342	8.532E-02	2.962E-01	28784.811	0.589	1.917E-03	3.369E-02
ENSG00000077782	FGFR1	511.836	-0.509	8.552E-02	2.966E-01	420.438	-0.009	9.686E-01	9.871E-01
ENSG00000147471	PLPBP	280.096	-0.511	8.589E-02	2.974E-01	316.225	-0.116	6.228E-01	8.362E-01
ENSG00000134247	PTGFRN	32.181	-0.788	8.600E-02	2.976E-01	28.375	-0.095	8.190E-01	9.271E-01
ENSG00000241127	YAE1D1	54.936	-0.750	8.651E-02	2.986E-01	55.368	-0.114	7.595E-01	9.017E-01
ENSG00000177683	THAP5	89.204	-0.616	8.666E-02	2.989E-01	70.136	-0.379	2.897E-01	6.001E-01
ENSG00000173221	GLRX	147.825	-0.617	8.675E-02	2.990E-01	93.586	0.273	3.642E-01	6.690E-01
ENSG00000197702	PARVA	119.799	-0.575	8.744E-02	3.002E-01	139.102	0.312	2.658E-01	5.754E-01
ENSG00000123607	TTC21B	159.662	-0.508	8.767E-02	3.007E-01	131.987	0.028	9.218E-01	9.712E-01
ENSG00000024862	CCDC28A	178.471	-0.526	8.786E-02	3.012E-01	126.636	-0.414	1.763E-01	4.721E-01
ENSG00000037897	METTL1	232.893	-0.561	8.815E-02	3.019E-01	211.973	-0.199	4.874E-01	7.532E-01
ENSG00000109475	RPL34	10273.175	0.408	8.820E-02	3.019E-01	7768.440	0.561	2.599E-03	4.106E-02
ENSG00000125968	ID1	5188.718	0.487	8.835E-02	3.022E-01	4104.769	0.724	1.751E-06	1.754E-04
ENSG00000105171	POP4	121.699	-0.532	8.877E-02	3.031E-01	132.928	-0.264	3.602E-01	6.655E-01
ENSG00000166436	TRIM66	49.727	-0.748	8.898E-02	3.036E-01	46.722	-0.448	2.652E-01	5.750E-01
ENSG00000172339	ALG14	54.845	-0.729	8.911E-02	3.038E-01	58.818	-0.282	4.457E-01	7.255E-01
ENSG00000247077	PGAM5	329.418	0.470	8.918E-02	3.039E-01	179.285	0.556	3.726E-02	2.074E-01
ENSG00000171017	LRRC8E	122.885	-0.561	8.923E-02	3.040E-01	107.765	-0.047	8.810E-01	9.543E-01
ENSG00000158006	PAFAH2	90.687	-0.622	8.956E-02	3.046E-01	89.543	-0.277	3.786E-01	6.789E-01
ENSG00000185650	ZFP36L1	1713.378	0.374	8.955E-02	3.046E-01	1401.290	0.722	1.350E-04	5.614E-03
ENSG00000198732	SMOC1	237.399	-0.506	8.976E-02	3.052E-01	152.096	-0.041	9.011E-01	9.631E-01
ENSG00000153574	RPIA	276.201	0.479	9.011E-02	3.059E-01	275.861	0.755	6.073E-03	7.173E-02
ENSG00000279078	SND1-IT1	42.733	-0.800	9.027E-02	3.061E-01	62.322	0.056	8.712E-01	9.512E-01
ENSG00000188603	CLN3	157.671	-0.521	9.030E-02	3.061E-01	115.982	-0.436	1.767E-01	4.726E-01
ENSG00000089682	RBM41	112.654	-0.612	9.049E-02	3.061E-01	117.481	0.063	8.274E-01	9.315E-01
ENSG00000116704	SLC35D1	252.929	-0.510	9.068E-02	3.065E-01	315.734	-0.051	8.136E-01	9.249E-01

ENSG00000106009	BRAT1	102.037	-0.586	9.088E-02	3.067E-01	97.202	0.160	6.003E-01	8.220E-01
ENSG00000181523	SGSH	50.223	-0.749	9.101E-02	3.069E-01	44.591	-0.467	2.354E-01	5.418E-01
ENSG00000038210	PI4K2B	91.244	-0.657	9.112E-02	3.071E-01	77.611	0.060	8.481E-01	9.422E-01
ENSG00000197081	IGF2R	677.889	-0.555	9.191E-02	3.087E-01	685.808	-0.015	9.429E-01	9.787E-01
ENSG00000166181	API5	170.506	-0.504	9.207E-02	3.091E-01	103.226	-0.208	5.242E-01	7.782E-01
ENSG00000213390	ARHGAP19	58.259	-0.691	9.227E-02	3.093E-01	39.563	-0.321	4.010E-01	6.979E-01
ENSG00000146477	SLC22A3	87.876	-0.650	9.253E-02	3.099E-01	63.173	0.036	9.145E-01	9.684E-01
ENSG00000137040	RANBP6	88.602	-0.628	9.297E-02	3.107E-01	92.672	-0.007	9.833E-01	9.929E-01
ENSG00000254004	ZNF260	45.092	-0.703	9.336E-02	3.115E-01	42.580	-0.437	2.620E-01	5.718E-01
ENSG00000276672	AL161891.1	44.949	-0.821	9.343E-02	3.116E-01	29.713	-1.072	1.067E-02	1.011E-01
ENSG00000166529	ZSCAN21	39.263	-0.782	9.359E-02	3.121E-01	36.456	0.241	5.527E-01	7.942E-01
ENSG00000110400	NECTIN1	104.329	-0.598	9.440E-02	3.137E-01	63.689	-0.050	8.844E-01	9.556E-01
ENSG00000171234	UGT2B7	175.159	-0.566	9.441E-02	3.137E-01	187.457	-0.106	6.968E-01	8.709E-01
ENSG00000056972	TRAF3IP2	50.625	-0.675	9.457E-02	3.140E-01	57.616	-0.488	1.778E-01	4.739E-01
ENSG00000077152	UBE2T	264.424	-0.587	9.503E-02	3.152E-01	200.627	0.548	4.072E-02	2.185E-01
ENSG00000078804	TP53INP2	83.547	-0.760	9.650E-02	3.184E-01	58.148	-0.499	2.220E-01	5.272E-01
ENSG00000146842	TMEM209	35.040	-0.745	9.670E-02	3.187E-01	41.120	-0.448	2.407E-01	5.481E-01
ENSG00000173153	ESRRA	189.610	-0.627	9.686E-02	3.188E-01	116.117	0.286	3.848E-01	6.840E-01
ENSG00000205356	TECPR1	63.009	-0.645	9.690E-02	3.189E-01	53.333	-0.469	2.114E-01	5.147E-01
ENSG00000157212	PAXIP1	119.882	-0.534	9.702E-02	3.190E-01	116.272	-0.034	9.102E-01	9.677E-01
ENSG00000173530	TNFRSF10D	202.093	0.459	9.707E-02	3.191E-01	168.477	0.538	4.539E-02	2.311E-01
ENSG00000147592	LACTB2	93.439	-0.592	9.737E-02	3.197E-01	98.910	-0.284	3.644E-01	6.691E-01
ENSG00000158863	FAM160B2	79.714	-0.569	9.787E-02	3.209E-01	98.844	-0.192	5.530E-01	7.942E-01
ENSG00000196865	NHLRC2	75.654	-0.643	9.812E-02	3.215E-01	98.427	0.012	9.668E-01	9.866E-01
ENSG00000155111	CDK19	90.383	-0.649	9.853E-02	3.223E-01	158.691	-0.273	3.237E-01	6.316E-01
ENSG00000164172	MOCS2	168.000	-0.563	9.854E-02	3.223E-01	118.797	-0.348	2.340E-01	5.394E-01

ENSG00000196850	PPTC7	281.392	0.488	9.865E-02	3.224E-01	200.130	1.263	1.585E-04	6.188E-03
ENSG00000038532	CLEC16A	108.903	-0.538	9.877E-02	3.226E-01	94.171	-0.448	1.601E-01	4.508E-01
ENSG00000160410	SHKBP1	112.553	-0.546	9.913E-02	3.234E-01	90.156	-0.025	9.443E-01	9.794E-01
ENSG00000106603	COA1	257.395	-0.559	9.932E-02	3.237E-01	181.640	0.256	3.782E-01	6.788E-01
ENSG00000213516	RBMXL1	159.312	-0.518	9.942E-02	3.238E-01	144.510	-0.177	5.050E-01	7.642E-01
ENSG00000185090	MANEAL	150.987	-0.526	9.961E-02	3.241E-01	183.419	-0.281	2.960E-01	6.060E-01
ENSG00000147133	TAF1	309.947	-0.523	1.001E-01	3.251E-01	238.392	-0.340	2.809E-01	5.921E-01
ENSG00000100575	TIMM9	288.379	0.477	1.002E-01	3.255E-01	245.341	0.710	2.401E-03	3.909E-02
ENSG00000272578	AP000347.1	36.266	-0.769	1.005E-01	3.262E-01	24.941	-0.833	4.380E-02	2.276E-01
ENSG00000132437	DDC	203.535	-0.605	1.018E-01	3.292E-01	195.763	-0.272	2.808E-01	5.921E-01
ENSG00000148572	NRBF2	198.803	0.493	1.020E-01	3.294E-01	172.607	0.589	3.682E-02	2.065E-01
ENSG00000143373	ZNF687	106.017	-0.525	1.022E-01	3.297E-01	86.457	-0.019	9.622E-01	9.846E-01
ENSG00000125870	SNRPB2	4419.296	0.324	1.024E-01	3.297E-01	3594.996	0.509	6.604E-03	7.511E-02
ENSG00000169964	TMEM42	54.484	-0.687	1.031E-01	3.308E-01	77.967	-0.110	7.312E-01	8.886E-01
ENSG00000114735	HEMK1	62.456	-0.683	1.031E-01	3.308E-01	44.540	-0.252	5.021E-01	7.624E-01
ENSG00000081692	JMJD4	101.685	-0.579	1.030E-01	3.308E-01	120.250	-0.438	1.593E-01	4.503E-01
ENSG00000103888	CEMIP	92.571	-0.560	1.031E-01	3.308E-01	60.447	-0.148	7.027E-01	8.744E-01
ENSG00000127946	HIP1	53.172	-0.662	1.034E-01	3.311E-01	100.614	-0.170	6.046E-01	8.254E-01
ENSG00000078142	PIK3C3	132.400	-0.550	1.038E-01	3.318E-01	152.293	-0.322	2.678E-01	5.776E-01
ENSG00000080371	RAB21	290.807	0.481	1.040E-01	3.322E-01	193.450	0.572	5.770E-02	2.633E-01
ENSG00000188542	DUSP28	32.168	-0.769	1.048E-01	3.339E-01	37.809	0.386	3.189E-01	6.285E-01
ENSG00000269688	AC008982.2	55.803	-0.711	1.051E-01	3.342E-01	24.999	-0.093	8.204E-01	9.275E-01
ENSG00000123473	STIL	267.605	-0.546	1.051E-01	3.342E-01	183.603	0.472	8.783E-02	3.285E-01
ENSG00000139618	BRCA2	245.487	-0.501	1.052E-01	3.345E-01	216.446	-0.367	1.675E-01	4.603E-01
ENSG00000120539	MASTL	71.239	-0.584	1.057E-01	3.355E-01	41.547	-0.306	4.463E-01	7.260E-01
ENSG00000107882	SUFU	52.889	-0.752	1.059E-01	3.356E-01	73.508	-0.431	1.862E-01	4.855E-01

ENSG00000115687	PASK	82.546	-0.593	1.060E-01	3.358E-01	74.409	-0.459	1.870E-01	4.861E-01
ENSG00000170085	SIMC1	38.604	-0.700	1.062E-01	3.359E-01	49.297	0.034	9.235E-01	9.719E-01
ENSG00000235381	AL596202.1	33.028	-0.754	1.066E-01	3.360E-01	25.966	0.293	4.813E-01	7.513E-01
ENSG00000168078	PBK	137.188	-0.547	1.066E-01	3.360E-01	199.976	0.013	9.637E-01	9.849E-01
ENSG00000101216	GMEB2	348.157	0.374	1.066E-01	3.360E-01	263.176	0.608	1.348E-02	1.169E-01
ENSG00000167487	KLHL26	44.237	-0.812	1.075E-01	3.374E-01	64.922	-0.333	3.362E-01	6.438E-01
ENSG00000008869	HEATR5B	101.157	-0.592	1.075E-01	3.374E-01	59.511	-0.169	6.259E-01	8.382E-01
ENSG00000173588	CEP83	87.581	-0.575	1.075E-01	3.374E-01	87.493	0.038	9.116E-01	9.679E-01
ENSG00000158169	FANCC	67.432	-0.676	1.084E-01	3.393E-01	66.345	-0.073	8.421E-01	9.389E-01
ENSG00000165406	MARCH8	106.398	-0.513	1.085E-01	3.393E-01	119.897	-0.125	6.756E-01	8.605E-01
ENSG00000177058	SLC38A9	36.468	-0.773	1.085E-01	3.393E-01	33.288	0.252	5.404E-01	7.878E-01
ENSG00000112343	TRIM38	40.351	-0.719	1.086E-01	3.395E-01	104.126	0.016	9.589E-01	9.842E-01
ENSG00000204634	TBC1D8	154.875	-0.501	1.089E-01	3.400E-01	222.624	0.185	5.397E-01	7.878E-01
ENSG00000123178	SPRYD7	134.812	0.479	1.088E-01	3.400E-01	137.954	0.505	1.010E-01	3.556E-01
ENSG00000136710	CCDC115	206.153	-0.500	1.096E-01	3.414E-01	301.848	0.057	7.922E-01	9.172E-01
ENSG00000166197	NOLC1	1050.736	0.309	1.096E-01	3.414E-01	817.115	0.571	1.337E-02	1.160E-01
ENSG00000127995	CASD1	69.251	-0.680	1.099E-01	3.420E-01	60.016	-0.402	2.559E-01	5.663E-01
ENSG00000188811	NHLRC3	60.653	-0.670	1.104E-01	3.433E-01	54.260	-0.145	6.958E-01	8.702E-01
ENSG00000105289	TJP3	37.069	-0.763	1.106E-01	3.439E-01	29.950	-0.692	9.147E-02	3.357E-01
ENSG00000139835	GRTP1	131.826	-0.538	1.110E-01	3.446E-01	88.724	-0.187	5.471E-01	7.909E-01
ENSG00000087299	L2HGDH	31.714	-0.830	1.112E-01	3.449E-01	59.514	-0.482	2.286E-01	5.340E-01
ENSG00000214194	SMIM30	60.325	-0.768	1.114E-01	3.453E-01	41.570	0.155	7.044E-01	8.748E-01
ENSG00000101843	PSMD10	112.748	-0.550	1.116E-01	3.459E-01	99.540	0.119	6.931E-01	8.685E-01
ENSG00000066933	MYO9A	126.293	-0.512	1.118E-01	3.462E-01	118.663	-0.097	7.381E-01	8.914E-01
ENSG00000213588	ZBTB9	70.999	-0.617	1.119E-01	3.463E-01	50.393	-0.448	2.392E-01	5.465E-01
ENSG00000243955	GSTA1	203.319	-0.556	1.120E-01	3.465E-01	112.277	-0.446	1.649E-01	4.571E-01

ENSG00000103199	ZNF500	75.580	-0.591	1.121E-01	3.466E-01	83.253	-0.262	4.353E-01	7.198E-01
ENSG00000169379	ARL13B	106.015	-0.602	1.126E-01	3.476E-01	195.108	0.202	4.519E-01	7.306E-01
ENSG00000181896	ZNF101	115.949	-0.540	1.127E-01	3.477E-01	102.219	-0.246	4.302E-01	7.167E-01
ENSG00000166855	CLPX	244.519	0.403	1.127E-01	3.478E-01	179.672	0.596	3.420E-02	1.990E-01
ENSG00000166579	NDEL1	273.654	0.437	1.129E-01	3.482E-01	314.223	0.523	2.135E-02	1.534E-01
ENSG00000198468	FLVCR1-AS1	49.090	-0.642	1.131E-01	3.485E-01	57.896	-0.197	5.848E-01	8.136E-01
ENSG00000023697	DERA	104.330	-0.618	1.139E-01	3.501E-01	138.489	0.139	6.937E-01	8.687E-01
ENSG00000158769	F11R	633.851	0.380	1.144E-01	3.508E-01	357.186	0.543	2.526E-02	1.679E-01
ENSG00000132205	EMILIN2	39.460	-0.731	1.147E-01	3.512E-01	19.345	-0.656	1.122E-01	NA
ENSG00000173349	SFT2D3	62.549	-0.616	1.151E-01	3.519E-01	68.332	-0.298	3.961E-01	6.949E-01
ENSG00000121039	RDH10	54.518	-0.662	1.159E-01	3.531E-01	61.722	-0.232	5.105E-01	7.681E-01
ENSG00000163611	SPICE1	33.077	-0.743	1.162E-01	3.536E-01	45.497	0.045	9.036E-01	9.643E-01
ENSG00000165055	METTL2B	101.979	-0.665	1.163E-01	3.537E-01	100.954	-0.438	1.717E-01	4.671E-01
ENSG00000224597	SVIL-AS1	178.934	0.494	1.165E-01	3.540E-01	148.069	0.633	4.207E-02	2.217E-01
ENSG00000180089	TMEM86B	163.141	-0.564	1.176E-01	3.557E-01	113.051	-0.180	5.970E-01	8.201E-01
ENSG00000272994	AC012360.3	56.708	-0.620	1.181E-01	3.569E-01	86.172	-0.285	4.004E-01	6.978E-01
ENSG00000143842	SOX13	139.560	-0.544	1.182E-01	3.569E-01	136.721	0.227	4.090E-01	7.033E-01
ENSG00000125629	INSIG2	135.315	-0.556	1.186E-01	3.577E-01	72.614	-0.046	8.997E-01	9.629E-01
ENSG00000077157	PPP1R12B	77.561	-0.547	1.196E-01	3.594E-01	108.156	-0.212	5.363E-01	7.865E-01
ENSG00000198001	IRAK4	32.140	-0.706	1.197E-01	3.595E-01	51.811	-0.315	3.908E-01	6.891E-01
ENSG00000163975	MELTF	138.777	-0.605	1.199E-01	3.597E-01	93.483	-0.046	8.990E-01	9.629E-01
ENSG00000268713	AC005261.3	35.422	-0.781	1.200E-01	3.597E-01	32.664	-0.001	9.984E-01	9.997E-01
ENSG00000112578	BYSL	355.466	0.367	1.203E-01	3.601E-01	273.564	0.537	1.597E-02	1.294E-01
ENSG00000102882	MAPK3	90.535	-0.674	1.206E-01	3.606E-01	69.431	-0.499	2.162E-01	5.213E-01
ENSG00000165282	PIGO	57.465	-0.602	1.207E-01	3.607E-01	36.041	0.525	1.986E-01	4.997E-01
ENSG00000280206	AC026401.3	218.952	0.456	1.207E-01	3.608E-01	194.408	0.558	3.585E-02	2.032E-01

ENSG00000107816	LZTS2	159.709	-0.561	1.211E-01	3.610E-01	93.426	0.128	6.701E-01	8.578E-01
ENSG00000129810	SGO1	127.458	-0.504	1.211E-01	3.610E-01	95.329	0.098	7.571E-01	9.007E-01
ENSG00000139718	SETD1B	101.033	-0.522	1.213E-01	3.615E-01	87.119	-0.242	4.595E-01	7.377E-01
ENSG00000012817	KDM5D	64.793	-0.588	1.216E-01	3.617E-01	88.693	0.225	4.862E-01	7.530E-01
ENSG00000198863	RUNDC1	111.355	-0.541	1.215E-01	3.617E-01	140.993	-0.324	2.853E-01	5.965E-01
ENSG00000120647	CCDC77	74.981	-0.622	1.218E-01	3.618E-01	68.628	-0.352	3.498E-01	6.557E-01
ENSG00000057757	PITHD1	167.939	0.467	1.218E-01	3.620E-01	170.264	0.508	5.748E-02	2.629E-01
ENSG00000196730	DAPK1	78.843	-0.616	1.234E-01	3.650E-01	52.524	0.125	7.258E-01	8.861E-01
ENSG00000143776	CDC42BPA	116.632	-0.576	1.235E-01	3.650E-01	143.698	-0.191	4.718E-01	7.468E-01
ENSG00000115084	SLC35F5	134.329	-0.577	1.249E-01	3.672E-01	156.972	0.308	2.504E-01	5.594E-01
ENSG00000172765	TMCC1	155.791	-0.507	1.251E-01	3.675E-01	152.857	-0.375	1.929E-01	4.935E-01
ENSG00000165527	ARF6	328.531	0.403	1.255E-01	3.682E-01	332.980	0.559	1.367E-02	1.174E-01
ENSG00000177646	ACAD9	128.152	-0.565	1.271E-01	3.712E-01	128.516	-0.394	1.608E-01	4.510E-01
ENSG00000171700	RGS19	49.278	-0.684	1.293E-01	3.758E-01	36.636	0.173	6.610E-01	8.536E-01
ENSG00000115392	FANCL	140.004	-0.537	1.295E-01	3.760E-01	125.888	-0.442	1.710E-01	4.659E-01
ENSG00000122958	VPS26A	257.191	0.460	1.299E-01	3.767E-01	245.345	0.675	6.923E-03	7.746E-02
ENSG00000262758	CTD-3195I5.1	37.545	-0.686	1.300E-01	3.770E-01	35.669	-0.295	4.689E-01	7.442E-01
ENSG00000040933	INPP4A	41.959	-0.708	1.302E-01	3.771E-01	40.773	-0.330	4.051E-01	7.010E-01
ENSG00000182944	EWSR1	953.162	0.335	1.303E-01	3.771E-01	785.810	0.815	2.820E-04	9.261E-03
ENSG00000160284	SPATC1L	58.046	-0.630	1.305E-01	3.774E-01	56.833	-0.062	8.716E-01	9.512E-01
ENSG00000100823	APEX1	928.047	-0.500	1.305E-01	3.774E-01	663.194	0.099	6.967E-01	8.709E-01
ENSG00000160408	ST6GALNAC6	41.921	-0.643	1.313E-01	3.791E-01	16.169	-0.101	7.032E-01	NA
ENSG00000143390	RFX5	50.770	-0.613	1.322E-01	3.811E-01	54.779	-0.119	7.397E-01	8.922E-01
ENSG00000025434	NR1H3	100.067	-0.528	1.329E-01	3.823E-01	92.381	-0.020	9.498E-01	9.815E-01
ENSG00000185278	ZBTB37	59.090	-0.646	1.332E-01	3.826E-01	51.800	-0.100	7.854E-01	9.132E-01
ENSG00000120054	CPN1	77.120	-0.635	1.333E-01	3.826E-01	79.749	-0.043	8.999E-01	9.629E-01

ENSG00000263956	NBPF11	44.334	-0.724	1.336E-01	3.833E-01	43.459	0.612	1.274E-01	4.029E-01
ENSG00000138308	PLA2G12B	74.342	-0.571	1.336E-01	3.833E-01	77.900	-0.475	1.590E-01	4.498E-01
ENSG00000196912	ANKRD36B	36.191	-0.697	1.347E-01	3.853E-01	52.799	-0.393	2.670E-01	5.767E-01
ENSG00000126603	GLIS2	22.292	0.797	1.352E-01	3.865E-01	47.053	0.569	1.388E-01	4.208E-01
ENSG00000142892	PIGK	42.769	-0.670	1.356E-01	3.872E-01	45.984	0.175	6.453E-01	8.463E-01
ENSG00000184162	NR2C2AP	190.911	0.464	1.359E-01	3.880E-01	119.713	0.609	3.265E-02	1.931E-01
ENSG00000014123	UFL1	234.553	0.393	1.370E-01	3.898E-01	248.412	0.506	4.805E-02	2.383E-01
ENSG00000102781	KATNAL1	63.572	-0.583	1.376E-01	3.906E-01	56.162	-0.037	9.167E-01	9.692E-01
ENSG00000064999	ANKS1A	119.153	-0.539	1.376E-01	3.906E-01	116.609	-0.098	7.648E-01	9.036E-01
ENSG00000135622	SEMA4F	49.666	-0.618	1.383E-01	3.916E-01	34.051	-0.355	3.671E-01	6.712E-01
ENSG00000198464	ZNF480	67.916	-0.562	1.388E-01	3.925E-01	83.736	-0.218	5.032E-01	7.636E-01
ENSG00000198176	TFDP1	1112.433	0.323	1.389E-01	3.927E-01	1096.923	0.624	1.096E-03	2.389E-02
ENSG00000171867	PRNP	527.626	0.466	1.390E-01	3.927E-01	453.902	0.618	8.731E-03	9.006E-02
ENSG00000166478	ZNF143	57.521	-0.608	1.391E-01	3.930E-01	46.035	0.132	7.421E-01	8.930E-01
ENSG00000089775	ZBTB25	31.404	-0.695	1.397E-01	3.938E-01	33.779	0.180	6.510E-01	8.495E-01
ENSG00000008282	SYPL1	333.374	0.397	1.397E-01	3.938E-01	299.057	0.579	2.089E-02	1.519E-01
ENSG00000110104	CCDC86	811.440	0.445	1.398E-01	3.938E-01	440.106	0.879	1.549E-04	6.120E-03
ENSG00000102871	TRADD	88.387	-0.514	1.411E-01	3.963E-01	89.869	-0.222	4.837E-01	7.530E-01
ENSG00000105866	SP4	32.310	-0.688	1.416E-01	3.968E-01	34.675	0.432	2.756E-01	5.860E-01
ENSG00000180198	RCC1	459.231	-0.511	1.418E-01	3.970E-01	286.405	0.462	7.782E-02	3.071E-01
ENSG00000108961	RANGRF	450.491	0.368	1.418E-01	3.970E-01	366.924	0.640	2.874E-03	4.405E-02
ENSG00000162378	ZYG11B	128.213	-0.520	1.420E-01	3.973E-01	151.969	-0.372	2.169E-01	5.220E-01
ENSG00000153107	ANAPC1	162.463	0.484	1.424E-01	3.979E-01	134.047	0.921	1.118E-03	2.418E-02
ENSG00000246273	SBF2-AS1	37.241	-0.673	1.426E-01	3.982E-01	31.942	0.200	6.133E-01	8.302E-01
ENSG00000197056	ZMYM1	30.165	-0.697	1.432E-01	3.993E-01	30.737	-0.062	8.776E-01	9.526E-01
ENSG00000007062	PROM1	46.271	-0.618	1.436E-01	4.000E-01	53.253	0.258	5.064E-01	7.649E-01

ENSG00000169504	CLIC4	336.592	0.433	1.438E-01	4.002E-01	276.259	0.573	6.422E-02	2.784E-01
ENSG00000170270	GON7	69.704	-0.676	1.448E-01	4.027E-01	54.239	-0.062	8.768E-01	9.525E-01
ENSG00000111879	FAM184A	62.570	-0.611	1.450E-01	4.027E-01	92.174	-0.193	5.406E-01	7.878E-01
ENSG00000183475	ASB7	89.516	-0.567	1.451E-01	4.028E-01	76.863	0.057	8.615E-01	9.475E-01
ENSG00000079974	RABL2B	159.158	-0.528	1.451E-01	4.028E-01	244.623	-0.188	5.346E-01	7.856E-01
ENSG00000108518	PFN1	2261.782	0.412	1.451E-01	4.028E-01	2257.574	0.545	2.266E-03	3.770E-02
ENSG00000101986	ABCD1	45.592	-0.691	1.452E-01	4.029E-01	40.017	0.721	6.715E-02	2.847E-01
ENSG00000205138	SDHAF1	85.492	-0.519	1.452E-01	4.029E-01	122.088	0.476	1.177E-01	3.855E-01
ENSG00000163453	IGFBP7	32.885	-0.743	1.454E-01	4.031E-01	24.792	-0.573	1.723E-01	4.682E-01
ENSG00000140950	TLDC1	81.458	-0.547	1.456E-01	4.033E-01	77.042	-0.289	4.007E-01	6.979E-01
ENSG00000106591	MRPL32	216.606	0.488	1.456E-01	4.033E-01	138.384	1.085	4.429E-04	1.298E-02
ENSG00000126775	ATG14	193.992	0.493	1.462E-01	4.038E-01	155.183	0.502	8.845E-02	3.299E-01
ENSG00000172766	NAA16	139.876	0.500	1.462E-01	4.038E-01	138.724	0.636	2.729E-02	1.740E-01
ENSG00000267317	AC027307.2	39.115	-0.643	1.462E-01	4.038E-01	69.316	-0.320	4.043E-01	7.008E-01
ENSG00000144134	RABL2A	34.776	-0.662	1.463E-01	4.039E-01	49.460	-0.323	4.080E-01	7.028E-01
ENSG00000132906	CASP9	47.841	-0.674	1.471E-01	4.050E-01	41.656	0.016	9.690E-01	9.871E-01
ENSG00000184445	KNTC1	76.211	-0.579	1.473E-01	4.054E-01	75.809	-0.013	9.678E-01	9.870E-01
ENSG00000280138	AC027290.2	21.377	0.750	1.490E-01	4.084E-01	36.351	0.640	1.182E-01	3.856E-01
ENSG00000115750	TAF1B	90.055	-0.574	1.496E-01	4.092E-01	139.300	-0.339	2.180E-01	5.232E-01
ENSG00000051382	PIK3CB	122.455	-0.573	1.499E-01	4.093E-01	97.989	0.525	9.483E-02	3.420E-01
ENSG00000131873	CHSY1	92.619	-0.502	1.503E-01	4.094E-01	90.020	0.219	4.837E-01	7.530E-01
ENSG00000163697	APBB2	95.649	-0.538	1.506E-01	4.098E-01	105.952	-0.477	1.152E-01	3.803E-01
ENSG00000115107	STEAP3	78.034	-0.533	1.519E-01	4.122E-01	61.988	0.180	6.112E-01	8.292E-01
ENSG00000225921	NOL7	1516.833	0.312	1.520E-01	4.124E-01	1098.530	0.554	3.165E-03	4.731E-02
ENSG00000139687	RB1	137.703	-0.550	1.522E-01	4.125E-01	124.465	0.243	4.738E-01	7.471E-01
ENSG00000116212	LRRC42	637.325	0.345	1.528E-01	4.131E-01	484.056	0.622	7.989E-03	8.492E-02

ENSG00000241878	PISD	301.627	0.492	1.538E-01	4.150E-01	203.406	0.589	2.420E-02	1.652E-01
ENSG00000204237	OXLD1	169.800	-0.517	1.539E-01	4.151E-01	184.455	-0.240	3.356E-01	6.434E-01
ENSG00000269947	AC135178.5	30.014	-0.653	1.540E-01	4.152E-01	22.173	-0.518	2.146E-01	NA
ENSG00000089022	MAPKAPK5	327.946	0.408	1.552E-01	4.171E-01	238.206	0.566	5.909E-02	2.671E-01
ENSG00000189171	S100A13	493.926	0.367	1.564E-01	4.184E-01	331.861	0.665	8.975E-03	9.125E-02
ENSG00000143756	FBXO28	56.699	-0.543	1.569E-01	4.193E-01	49.804	0.439	2.317E-01	5.375E-01
ENSG00000138658	ZGRF1	40.202	-0.670	1.572E-01	4.199E-01	22.381	-0.299	4.689E-01	NA
ENSG00000047634	SCML1	146.029	-0.578	1.575E-01	4.200E-01	87.108	0.069	8.281E-01	9.320E-01
ENSG00000155906	RMND1	132.789	0.470	1.575E-01	4.200E-01	107.998	0.951	8.210E-03	8.686E-02
ENSG00000185730	ZNF696	60.506	-0.610	1.586E-01	4.214E-01	51.998	0.091	8.190E-01	9.271E-01
ENSG00000153046	CDYL	249.744	0.357	1.592E-01	4.223E-01	255.183	0.530	2.881E-02	1.802E-01
ENSG00000180758	GPR157	101.876	-0.466	1.594E-01	4.226E-01	134.866	0.619	3.191E-02	1.908E-01
ENSG00000158457	TSPAN33	45.463	-0.588	1.595E-01	4.227E-01	51.814	0.211	5.676E-01	8.032E-01
ENSG00000169683	LRRC45	42.690	-0.617	1.607E-01	4.243E-01	23.119	-0.077	8.533E-01	9.443E-01
ENSG00000109971	HSPA8	1269.154	-0.710	1.609E-01	4.244E-01	993.037	-0.470	5.492E-02	2.564E-01
ENSG00000141564	RPTOR	73.537	-0.552	1.616E-01	4.256E-01	77.296	-0.381	2.280E-01	5.334E-01
ENSG00000121578	B4GALT4	128.027	-0.569	1.622E-01	4.260E-01	94.188	0.919	6.078E-03	7.173E-02
ENSG00000066379	ZNRD1	281.011	0.412	1.631E-01	4.275E-01	215.202	0.695	1.789E-02	1.383E-01
ENSG00000070423	RNF126	1851.780	0.338	1.641E-01	4.289E-01	1688.254	0.689	2.083E-03	3.568E-02
ENSG00000108061	SHOC2	194.836	0.469	1.642E-01	4.291E-01	221.944	0.501	6.770E-02	2.853E-01
ENSG00000116774	OLFML3	36.882	-0.659	1.643E-01	4.292E-01	41.581	-0.110	7.858E-01	9.133E-01
ENSG00000123545	NDUFAF4	263.828	0.431	1.647E-01	4.296E-01	185.814	0.736	7.305E-03	7.957E-02
ENSG00000259417	CTXND1	32.255	-0.631	1.647E-01	4.297E-01	18.533	-0.816	5.084E-02	NA
ENSG00000172159	FRMD3	61.178	-0.545	1.676E-01	4.338E-01	61.863	0.260	4.677E-01	7.436E-01
ENSG00000104133	SPG11	48.708	-0.642	1.681E-01	4.340E-01	58.177	0.048	8.920E-01	9.592E-01
ENSG00000142632	ARHGEF19	45.158	-0.583	1.680E-01	4.340E-01	57.309	0.414	2.638E-01	5.739E-01

ENSG00000060642	PIGV	39.134	-0.713	1.686E-01	4.347E-01	51.927	-0.324	3.828E-01	6.825E-01
ENSG00000181938	GINS3	79.169	-0.530	1.687E-01	4.348E-01	69.376	-0.288	4.189E-01	7.095E-01
ENSG00000115317	HTRA2	123.024	-0.506	1.698E-01	4.366E-01	99.160	0.262	4.248E-01	7.133E-01
ENSG00000198105	ZNF248	38.107	-0.587	1.701E-01	4.371E-01	61.233	-0.450	1.979E-01	4.992E-01
ENSG00000153406	NMRAL1	43.356	-0.654	1.705E-01	4.377E-01	25.362	-1.178	4.486E-03	5.971E-02
ENSG00000212385	RNU6-817P	30.345	-0.618	1.715E-01	4.388E-01	23.918	-0.428	3.045E-01	6.153E-01
ENSG00000166997	CNPY4	41.557	-0.596	1.716E-01	4.391E-01	39.548	-0.076	8.535E-01	9.443E-01
ENSG00000135069	PSAT1	1726.034	-0.361	1.731E-01	4.412E-01	1218.252	0.552	7.669E-03	8.248E-02
ENSG00000189308	LIN54	172.826	-0.519	1.740E-01	4.423E-01	103.047	0.473	1.817E-01	4.803E-01
ENSG00000119682	AREL1	70.317	-0.517	1.750E-01	4.435E-01	102.069	0.642	3.261E-02	1.931E-01
ENSG00000106366	SERPINE1	382.518	0.448	1.758E-01	4.444E-01	251.640	1.101	4.657E-04	1.349E-02
ENSG00000172785	CBWD1	64.633	-0.540	1.760E-01	4.447E-01	48.923	-0.198	5.933E-01	8.182E-01
ENSG00000147316	MCPH1	94.002	0.482	1.762E-01	4.448E-01	86.081	0.627	5.750E-02	2.629E-01
ENSG00000133816	MICAL2	34.855	-0.638	1.763E-01	4.449E-01	32.364	0.149	7.174E-01	8.827E-01
ENSG00000263563	AC138761.1	34.226	-0.677	1.766E-01	4.453E-01	43.162	0.205	5.870E-01	8.142E-01
ENSG00000241399	CD302	30.715	-0.635	1.766E-01	4.453E-01	26.242	-0.174	6.729E-01	8.594E-01
ENSG00000153561	RMND5A	600.258	0.347	1.766E-01	4.453E-01	486.495	0.601	8.943E-03	9.125E-02
ENSG00000085491	SLC25A24	70.759	-0.608	1.769E-01	4.457E-01	73.592	-0.267	4.721E-01	7.468E-01
ENSG00000110906	KCTD10	184.014	0.423	1.770E-01	4.457E-01	212.674	0.528	3.915E-02	2.138E-01
ENSG00000006459	KDM7A	132.571	0.458	1.773E-01	4.462E-01	128.143	0.901	1.189E-02	1.084E-01
ENSG00000043514	TRIT1	96.063	0.490	1.776E-01	4.465E-01	81.045	0.708	4.663E-02	2.350E-01
ENSG00000064651	SLC12A2	101.854	-0.516	1.779E-01	4.465E-01	82.090	-0.491	1.582E-01	4.484E-01
ENSG00000163933	RFT1	211.523	-0.400	1.786E-01	4.475E-01	171.526	0.504	7.612E-02	3.039E-01
ENSG00000085998	POMGNT1	64.044	-0.501	1.791E-01	4.479E-01	76.840	-0.271	4.098E-01	7.035E-01
ENSG00000273456	AC064836.3	35.538	-0.592	1.796E-01	4.486E-01	55.295	0.069	8.518E-01	9.441E-01
ENSG00000176532	PRR15	47.700	-0.589	1.795E-01	4.486E-01	77.702	-0.007	9.847E-01	9.936E-01

ENSG00000120008	WDR11	61.745	-0.615	1.797E-01	4.487E-01	81.471	-0.116	7.323E-01	8.888E-01
ENSG00000103067	ESRP2	77.950	-0.561	1.804E-01	4.498E-01	123.204	-0.095	7.488E-01	8.957E-01
ENSG00000119723	COQ6	102.450	-0.505	1.816E-01	4.514E-01	89.586	0.095	7.976E-01	9.188E-01
ENSG00000055208	TAB2	253.352	0.427	1.818E-01	4.516E-01	245.785	0.820	1.955E-03	3.406E-02
ENSG00000231607	DLEU2	80.011	-0.543	1.820E-01	4.519E-01	75.793	0.020	9.518E-01	9.821E-01
ENSG00000183309	ZNF623	33.838	-0.611	1.825E-01	4.526E-01	28.643	-0.719	8.019E-02	3.128E-01
ENSG00000153989	NUS1	105.232	-0.512	1.833E-01	4.539E-01	60.545	0.679	5.546E-02	2.579E-01
ENSG00000151576	QTRT2	222.998	0.462	1.839E-01	4.547E-01	172.155	0.520	6.976E-02	2.892E-01
ENSG00000145740	SLC30A5	63.996	-0.525	1.844E-01	4.557E-01	54.189	0.280	4.364E-01	7.203E-01
ENSG00000188725	SMIM15	330.508	0.401	1.851E-01	4.567E-01	279.454	0.589	1.894E-02	1.440E-01
ENSG00000008323	PLEKHG6	40.739	-0.581	1.856E-01	4.575E-01	33.700	0.163	6.842E-01	8.648E-01
ENSG00000023330	ALAS1	125.645	0.464	1.861E-01	4.580E-01	86.068	0.662	3.242E-02	1.924E-01
ENSG00000127870	RNF6	75.295	0.471	1.864E-01	4.585E-01	97.828	0.553	7.011E-02	2.898E-01
ENSG00000198898	CAPZA2	274.335	0.404	1.865E-01	4.586E-01	196.485	0.537	4.993E-02	2.431E-01
ENSG00000143771	CNIH4	413.014	0.404	1.865E-01	4.586E-01	357.565	0.549	1.337E-02	1.160E-01
ENSG00000154124	OTULIN	173.720	0.396	1.876E-01	4.604E-01	144.897	0.542	5.177E-02	2.484E-01
ENSG00000065970	FOXJ2	97.221	0.465	1.877E-01	4.605E-01	101.208	0.847	6.939E-03	7.746E-02
ENSG00000130810	PPAN	91.635	-0.518	1.888E-01	4.620E-01	74.894	-0.405	2.298E-01	5.353E-01
ENSG00000117625	RCOR3	144.633	0.393	1.893E-01	4.625E-01	170.708	0.805	2.291E-03	3.793E-02
ENSG00000167721	TSR1	840.762	0.276	1.895E-01	4.626E-01	745.763	0.688	7.060E-04	1.785E-02
ENSG00000197766	CFD	56.037	-0.524	1.896E-01	4.627E-01	82.836	-0.453	1.951E-01	4.961E-01
ENSG00000013573	DDX11	58.077	-0.503	1.899E-01	4.630E-01	59.020	-0.373	2.885E-01	5.993E-01
ENSG00000155034	FBXL18	30.993	-0.612	1.907E-01	4.641E-01	28.660	0.147	7.197E-01	8.832E-01
ENSG00000105879	CBLL1	79.837	-0.502	1.912E-01	4.647E-01	67.981	0.065	8.645E-01	9.489E-01
ENSG00000010278	CD9	33.908	-0.674	1.914E-01	4.649E-01	20.845	0.107	7.946E-01	NA
ENSG00000175893	ZDHHC21	77.720	-0.517	1.918E-01	4.651E-01	79.404	-0.436	1.715E-01	4.669E-01

ENSG00000135473	PAN2	35.072	-0.609	1.922E-01	4.656E-01	39.078	-0.270	5.093E-01	7.672E-01
ENSG00000172354	GNB2	226.223	0.490	1.929E-01	4.662E-01	127.203	0.985	7.877E-03	8.394E-02
ENSG00000143919	CAMKMT	53.569	-0.570	1.937E-01	4.675E-01	61.349	0.626	1.034E-01	3.602E-01
ENSG00000123191	ATP7B	85.897	-0.581	1.958E-01	4.703E-01	77.273	-0.105	7.518E-01	8.973E-01
ENSG00000164074	ABHD18	78.080	-0.514	1.960E-01	4.704E-01	56.761	0.209	5.699E-01	8.051E-01
ENSG00000177854	TMEM187	38.184	-0.556	1.966E-01	4.713E-01	29.535	-0.421	2.969E-01	6.071E-01
ENSG00000187790	FANCM	43.838	-0.615	1.972E-01	4.719E-01	34.153	-0.162	6.951E-01	8.696E-01
ENSG00000163320	CGGBP1	186.650	0.369	1.988E-01	4.739E-01	174.945	0.529	6.736E-02	2.850E-01
ENSG00000169062	UPF3A	358.678	0.293	1.995E-01	4.746E-01	281.428	0.564	2.019E-02	1.488E-01
ENSG00000179041	RRS1	284.436	0.372	1.993E-01	4.746E-01	185.186	0.815	3.279E-03	4.846E-02
ENSG00000103657	HERC1	155.490	-0.519	2.000E-01	4.750E-01	182.062	-0.350	1.889E-01	4.878E-01
ENSG00000236255	AC009404.1	44.357	-0.548	2.010E-01	4.760E-01	37.103	-0.290	4.612E-01	7.389E-01
ENSG00000174791	RIN1	48.611	-0.518	2.019E-01	4.769E-01	49.678	-0.130	7.327E-01	8.888E-01
ENSG00000107362	ABHD17B	107.111	0.462	2.022E-01	4.774E-01	82.851	0.773	2.666E-02	1.721E-01
ENSG00000178445	GLDC	134.190	-0.677	2.027E-01	4.777E-01	76.467	-0.054	8.817E-01	9.547E-01
ENSG00000104177	MYEF2	45.585	-0.535	2.026E-01	4.777E-01	76.281	-0.198	5.479E-01	7.916E-01
ENSG00000144867	SRPRB	812.183	0.258	2.032E-01	4.783E-01	682.858	0.775	3.006E-05	1.742E-03
ENSG00000148057	IDNK	33.068	-0.579	2.034E-01	4.787E-01	34.542	0.105	7.955E-01	9.183E-01
ENSG00000100154	TTC28	70.089	-0.529	2.035E-01	4.787E-01	72.350	0.115	7.477E-01	8.951E-01
ENSG00000163170	BOLA3	857.994	0.331	2.055E-01	4.814E-01	603.171	0.830	3.182E-03	4.749E-02
ENSG00000156162	DPY19L4	48.352	-0.537	2.057E-01	4.816E-01	52.964	-0.331	3.582E-01	6.640E-01
ENSG00000198833	UBE2J1	289.694	0.340	2.069E-01	4.835E-01	226.559	0.683	9.307E-03	9.357E-02
ENSG00000135919	SERPINE2	305.585	0.320	2.084E-01	4.858E-01	338.554	0.954	1.794E-05	1.142E-03
ENSG00000161980	POLR3K	360.833	0.291	2.087E-01	4.861E-01	255.384	0.606	9.546E-03	9.541E-02
ENSG00000107140	TESK1	33.683	-0.598	2.089E-01	4.862E-01	15.366	-0.498	2.257E-01	NA
ENSG00000048471	SNX29	54.510	-0.520	2.091E-01	4.862E-01	39.622	-0.058	8.829E-01	9.550E-01

ENSG00000116957	TBCE	231.737	0.374	2.097E-01	4.867E-01	170.134	0.550	4.651E-02	2.346E-01
ENSG00000107779	BMPR1A	52.002	-0.526	2.113E-01	4.887E-01	40.236	0.240	5.393E-01	7.874E-01
ENSG00000081014	AP4E1	48.225	-0.578	2.128E-01	4.911E-01	48.121	0.318	4.061E-01	7.017E-01
ENSG00000279059	AC007485.2	45.933	-0.528	2.128E-01	4.911E-01	48.835	-0.322	3.810E-01	6.812E-01
ENSG00000240616	RPS6P25	38.044	-0.625	2.141E-01	4.923E-01	24.675	-0.411	3.270E-01	6.352E-01
ENSG00000148824	MTG1	45.349	-0.533	2.155E-01	4.943E-01	40.492	-0.367	3.329E-01	6.406E-01
ENSG00000159899	NPR2	48.361	-0.524	2.164E-01	4.955E-01	27.464	-0.353	3.903E-01	6.889E-01
ENSG00000069345	DNAJA2	728.995	0.357	2.165E-01	4.955E-01	693.750	0.832	2.247E-05	1.382E-03
ENSG00000184669	OR7E14P	68.283	0.490	2.167E-01	4.959E-01	78.800	0.695	3.476E-02	2.003E-01
ENSG00000179979	CRIPAK	34.153	-0.607	2.174E-01	4.968E-01	47.787	-0.114	7.579E-01	9.010E-01
ENSG00000164209	SLC25A46	66.266	-0.653	2.192E-01	4.997E-01	91.150	-0.162	6.336E-01	8.411E-01
ENSG00000275234	AC010503.4	33.858	-0.597	2.198E-01	5.006E-01	26.776	-0.305	4.547E-01	7.336E-01
ENSG00000166925	TSC22D4	42.757	-0.589	2.207E-01	5.014E-01	26.011	-0.001	9.973E-01	9.993E-01
ENSG00000085978	ATG16L1	193.985	0.340	2.207E-01	5.014E-01	163.122	0.582	3.853E-02	2.117E-01
ENSG00000007255	TRAPPC6A	31.868	-0.582	2.210E-01	5.017E-01	39.637	-0.392	2.492E-01	5.582E-01
ENSG00000186063	AIDA	39.489	-0.580	2.233E-01	5.051E-01	30.316	0.362	3.734E-01	6.761E-01
ENSG00000156232	WHAMM	189.600	0.381	2.243E-01	5.063E-01	194.070	0.556	2.538E-02	1.685E-01
ENSG00000270172	RP5-1024G6.8	44.798	-0.567	2.255E-01	5.077E-01	33.542	-0.046	9.071E-01	9.656E-01
ENSG00000198040	ZNF84	62.682	-0.574	2.262E-01	5.085E-01	62.371	-0.155	6.612E-01	8.536E-01
ENSG00000120306	CYSTM1	141.079	0.428	2.267E-01	5.091E-01	353.350	0.847	2.003E-03	3.462E-02
ENSG00000091651	ORC6	334.906	0.367	2.268E-01	5.093E-01	238.194	0.632	9.131E-03	9.207E-02
ENSG00000259429	UBE2Q2P2	29.168	0.586	2.277E-01	5.106E-01	37.813	0.916	2.905E-02	1.811E-01
ENSG00000132623	ANKEF1	31.275	-0.629	2.287E-01	5.114E-01	26.065	-0.775	6.019E-02	2.700E-01
ENSG00000184307	ZDHHC23	43.488	-0.563	2.291E-01	5.117E-01	43.136	-0.207	5.886E-01	8.153E-01
ENSG00000165689	SDCCAG3	263.967	-0.392	2.291E-01	5.117E-01	391.143	0.545	1.013E-02	9.810E-02
ENSG00000137075	RNF38	79.944	0.418	2.295E-01	5.118E-01	106.553	0.844	4.642E-03	6.100E-02

ENSG00000136943	CTSV	46.307	-0.603	2.296E-01	5.120E-01	12.570	0.292	4.803E-01	NA
ENSG00000140743	CDR2	252.248	0.413	2.303E-01	5.128E-01	266.181	0.934	7.243E-04	1.827E-02
ENSG00000186908	ZDHHC17	83.207	0.409	2.308E-01	5.133E-01	71.696	0.755	2.616E-02	1.713E-01
ENSG00000155975	VPS37A	138.468	0.472	2.313E-01	5.140E-01	114.621	1.083	1.414E-03	2.741E-02
ENSG00000164291	ARSK	52.942	-0.516	2.316E-01	5.145E-01	46.327	0.123	7.423E-01	8.930E-01
ENSG00000152332	UHMK1	153.647	0.381	2.321E-01	5.151E-01	182.360	0.587	2.275E-02	1.588E-01
ENSG00000174365	SNHG11	265.842	0.303	2.326E-01	5.155E-01	253.045	0.682	1.173E-02	1.074E-01
ENSG00000054598	FOXC1	199.891	0.436	2.334E-01	5.160E-01	236.648	0.669	1.025E-02	9.847E-02
ENSG00000198912	C1orf174	180.044	0.499	2.346E-01	5.173E-01	85.692	0.700	7.619E-02	3.039E-01
ENSG00000204315	FKBPL	32.642	-0.559	2.361E-01	5.190E-01	30.583	-0.378	3.643E-01	6.690E-01
ENSG00000178105	DDX10	632.072	0.334	2.377E-01	5.215E-01	510.425	0.694	1.306E-03	2.611E-02
ENSG00000075188	NUP37	249.170	-0.409	2.389E-01	5.225E-01	171.439	0.541	6.088E-02	2.719E-01
ENSG00000074842	MYDGF	1638.703	0.227	2.389E-01	5.225E-01	1101.915	0.503	6.605E-03	7.511E-02
ENSG00000104299	INTS9	53.922	-0.518	2.424E-01	5.272E-01	30.186	-0.136	7.448E-01	8.937E-01
ENSG00000176142	TMEM39A	176.478	0.357	2.424E-01	5.272E-01	175.365	0.678	9.154E-03	9.221E-02
ENSG00000245556	SCAMP1-AS1	41.592	-0.520	2.431E-01	5.283E-01	41.999	-0.129	7.325E-01	8.888E-01
ENSG00000091009	RBM27	208.095	0.385	2.444E-01	5.297E-01	199.292	0.866	1.063E-03	2.363E-02
ENSG00000090581	GNPTG	100.405	0.489	2.452E-01	5.307E-01	76.560	0.935	7.025E-03	7.795E-02
ENSG00000132522	GPS2	111.554	0.429	2.455E-01	5.310E-01	132.919	0.730	1.600E-02	1.295E-01
ENSG00000123268	ATF1	162.339	0.349	2.472E-01	5.329E-01	130.615	0.758	1.981E-02	1.479E-01
ENSG00000156973	PDE6D	32.528	-0.574	2.480E-01	5.332E-01	45.584	-0.487	2.160E-01	5.213E-01
ENSG00000100379	KCTD17	73.388	-0.510	2.485E-01	5.336E-01	56.266	-0.476	2.111E-01	5.142E-01
ENSG00000198901	PRC1	529.588	-0.258	2.498E-01	5.354E-01	699.665	0.571	1.304E-02	1.146E-01
ENSG00000124222	STX16	457.682	0.291	2.497E-01	5.354E-01	530.721	0.510	1.664E-02	1.322E-01
ENSG00000138621	PPCDC	67.632	0.438	2.503E-01	5.359E-01	37.850	0.931	1.694E-02	1.334E-01
ENSG00000168060	NAALADL1	32.351	-0.600	2.516E-01	5.378E-01	18.501	-0.729	8.099E-02	NA

ENSG00000240771	ARHGEF25	63.931	-0.523	2.525E-01	5.388E-01	61.489	-0.080	8.333E-01	9.345E-01
ENSG00000145041	DCAF1	151.504	-0.425	2.526E-01	5.388E-01	116.706	0.662	5.970E-02	2.691E-01
ENSG00000106853	PTGR1	248.764	0.337	2.531E-01	5.394E-01	134.731	0.961	2.004E-03	3.462E-02
ENSG00000137806	NDUFAF1	50.335	-0.552	2.535E-01	5.400E-01	52.407	0.164	6.542E-01	8.507E-01
ENSG00000076864	RAP1GAP	91.394	0.415	2.536E-01	5.400E-01	75.876	0.685	6.284E-02	2.750E-01
ENSG00000103222	ABCC1	144.427	0.426	2.537E-01	5.402E-01	94.503	0.546	1.036E-01	3.602E-01
ENSG00000157107	FCHO2	53.671	0.477	2.545E-01	5.415E-01	52.231	1.035	5.680E-03	6.885E-02
ENSG00000272277	AL031963.3	31.175	-0.521	2.550E-01	5.421E-01	22.966	0.152	7.175E-01	8.827E-01
ENSG00000007372	PAX6	22.077	0.590	2.561E-01	5.434E-01	32.854	0.652	1.096E-01	3.702E-01
ENSG00000155508	CNOT8	216.157	0.306	2.565E-01	5.440E-01	161.886	0.843	3.706E-03	5.266E-02
ENSG00000173409	ARV1	41.701	-0.538	2.595E-01	5.469E-01	47.618	0.011	9.773E-01	9.909E-01
ENSG00000166886	NAB2	68.169	0.454	2.598E-01	5.474E-01	58.600	0.528	1.567E-01	4.470E-01
ENSG00000186871	ERCC6L	33.431	-0.539	2.629E-01	5.507E-01	32.186	-0.135	7.306E-01	8.886E-01
ENSG00000156795	WDYHV1	55.422	0.440	2.630E-01	5.508E-01	42.054	0.919	1.708E-02	1.340E-01
ENSG00000169955	ZNF747	45.920	-0.502	2.642E-01	5.526E-01	58.564	-0.430	2.566E-01	5.668E-01
ENSG00000089876	DHX32	38.083	-0.531	2.665E-01	5.555E-01	37.383	0.276	4.904E-01	7.553E-01
ENSG00000177034	MTX3	55.099	0.481	2.669E-01	5.557E-01	43.755	0.856	2.972E-02	1.829E-01
ENSG00000235194	PPP1R3E	57.544	-0.501	2.675E-01	5.561E-01	101.228	-0.130	6.707E-01	8.581E-01
ENSG00000214367	HAUS3	76.192	0.445	2.678E-01	5.565E-01	59.460	0.920	1.440E-02	1.209E-01
ENSG00000040199	PHLPP2	43.496	-0.476	2.685E-01	5.573E-01	38.828	0.605	1.158E-01	3.816E-01
ENSG00000168394	TAP1	120.833	0.388	2.691E-01	5.578E-01	188.090	0.633	1.899E-02	1.440E-01
ENSG00000213598	AL049873.1	42.811	0.497	2.692E-01	5.578E-01	44.653	0.520	1.820E-01	4.805E-01
ENSG00000169740	ZNF32	41.882	-0.538	2.700E-01	5.588E-01	23.599	-0.623	1.290E-01	4.049E-01
ENSG00000172345	STARD5	30.512	-0.509	2.716E-01	5.601E-01	33.991	0.869	3.196E-02	1.909E-01
ENSG00000181031	RPH3AL	34.960	-0.509	2.716E-01	5.601E-01	33.714	0.038	9.234E-01	9.719E-01
ENSG00000115641	FHL2	66.098	0.465	2.721E-01	5.607E-01	57.455	0.647	6.095E-02	2.720E-01

ENSG00000221944	TIGD1	44.930	-0.530	2.730E-01	5.617E-01	49.015	-0.349	3.475E-01	6.535E-01
ENSG00000100567	PSMA3	787.142	0.333	2.732E-01	5.617E-01	565.008	0.561	1.131E-02	1.049E-01
ENSG00000166199	ALKBH3	293.216	0.330	2.734E-01	5.619E-01	320.489	0.528	1.384E-02	1.183E-01
ENSG00000236810	ELOA-AS1	31.501	-0.545	2.740E-01	5.623E-01	46.221	0.444	2.445E-01	5.531E-01
ENSG00000141428	C18orf21	90.773	0.435	2.740E-01	5.623E-01	83.550	0.871	1.574E-02	1.284E-01
ENSG00000179454	KLHL28	58.147	0.436	2.740E-01	5.623E-01	46.523	0.628	8.303E-02	3.185E-01
ENSG00000121690	DEPDC7	68.647	0.495	2.744E-01	5.626E-01	55.596	1.168	3.136E-03	4.702E-02
ENSG00000151929	BAG3	208.716	0.354	2.762E-01	5.647E-01	153.427	0.782	6.735E-03	7.592E-02
ENSG00000164306	PRIMPOL	31.822	-0.508	2.768E-01	5.652E-01	43.750	-0.290	4.691E-01	7.443E-01
ENSG00000163608	NEPRO	231.443	0.297	2.768E-01	5.652E-01	249.899	0.638	5.563E-03	6.799E-02
ENSG00000158480	SPATA2	309.570	0.335	2.778E-01	5.661E-01	211.363	0.535	4.479E-02	2.297E-01
ENSG00000157110	RBPMS	595.599	0.312	2.798E-01	5.687E-01	451.702	0.518	1.563E-02	1.278E-01
ENSG00000106635	BCL7B	95.958	0.453	2.805E-01	5.691E-01	93.464	0.745	3.489E-02	2.006E-01
ENSG00000174109	C16orf91	60.044	0.493	2.826E-01	5.710E-01	60.554	0.757	6.407E-02	2.782E-01
ENSG00000130787	HIP1R	29.749	0.514	2.826E-01	5.710E-01	31.484	1.112	5.967E-03	7.083E-02
ENSG00000169131	ZNF354A	26.479	0.527	2.830E-01	5.715E-01	34.743	0.817	3.715E-02	2.072E-01
ENSG00000120963	ZNF706	197.246	0.413	2.852E-01	5.742E-01	187.391	0.760	1.599E-02	1.295E-01
ENSG00000107443	CCNJ	85.144	0.435	2.866E-01	5.756E-01	135.385	0.533	5.726E-02	2.624E-01
ENSG00000152520	PAN3	67.005	0.421	2.878E-01	5.767E-01	92.271	0.841	9.384E-03	9.407E-02
ENSG00000154059	IMPACT	75.197	0.391	2.884E-01	5.771E-01	68.052	0.718	2.963E-02	1.825E-01
ENSG00000120265	PCMT1	1361.603	0.223	2.905E-01	5.791E-01	1002.720	0.521	3.549E-03	5.120E-02
ENSG00000115364	MRPL19	366.529	-0.301	2.909E-01	5.794E-01	328.963	0.647	1.651E-02	1.314E-01
ENSG00000121481	RNF2	81.432	0.414	2.927E-01	5.811E-01	75.642	0.698	3.461E-02	2.001E-01
ENSG00000248909	HMGB1P21	44.277	0.480	2.953E-01	5.842E-01	46.962	0.839	2.653E-02	1.717E-01
ENSG00000154889	MPPE1	44.435	0.479	2.977E-01	5.869E-01	39.450	0.519	1.974E-01	4.986E-01
ENSG00000157796	WDR19	35.139	-0.531	3.041E-01	5.939E-01	42.022	-0.074	8.449E-01	9.404E-01

ENSG00000106993	CDC37L1	191.845	0.287	3.044E-01	5.940E-01	159.008	0.800	6.001E-03	7.099E-02
ENSG00000254911	SCARNA9	276.005	-0.533	3.055E-01	5.953E-01	224.200	-0.267	2.936E-01	6.034E-01
ENSG00000037637	FBXO42	59.527	0.422	3.057E-01	5.954E-01	78.077	0.539	1.035E-01	3.602E-01
ENSG00000114784	EIF1B	329.796	0.311	3.119E-01	6.017E-01	335.115	0.690	4.112E-03	5.648E-02
ENSG00000132950	ZMYM5	51.815	0.411	3.147E-01	6.045E-01	33.440	0.607	1.285E-01	4.041E-01
ENSG00000124209	RAB22A	229.363	0.286	3.154E-01	6.052E-01	245.254	0.581	1.904E-02	1.441E-01
ENSG00000111832	RWDD1	996.171	0.249	3.172E-01	6.066E-01	923.212	0.528	1.356E-02	1.171E-01
ENSG00000042429	MED17	128.801	0.311	3.179E-01	6.070E-01	101.821	0.588	4.969E-02	2.421E-01
ENSG00000155744	FAM126B	194.792	0.273	3.188E-01	6.080E-01	127.009	0.813	5.174E-03	6.535E-02
ENSG00000080822	CLDND1	89.198	0.424	3.191E-01	6.083E-01	59.723	1.224	5.678E-04	1.541E-02
ENSG00000198799	LRIG2	51.454	0.411	3.199E-01	6.092E-01	39.102	0.554	1.758E-01	4.715E-01
ENSG00000166188	ZNF319	34.483	-0.509	3.212E-01	6.111E-01	38.043	-0.292	4.649E-01	7.416E-01
ENSG00000134569	LRP4	42.318	-0.460	3.227E-01	6.127E-01	64.302	0.500	1.706E-01	4.655E-01
ENSG00000186352	ANKRD37	31.621	-0.514	3.268E-01	6.163E-01	18.434	0.246	5.569E-01	NA
ENSG00000010017	RANBP9	144.362	0.420	3.287E-01	6.175E-01	119.572	0.846	1.644E-02	1.313E-01
ENSG00000116954	RRAGC	57.638	0.405	3.295E-01	6.179E-01	48.504	0.806	3.484E-02	2.006E-01
ENSG00000100612	DHRS7	147.104	0.367	3.303E-01	6.187E-01	133.388	0.646	5.753E-02	2.629E-01
ENSG00000203791	EEF1AKMT2	85.590	-0.354	3.312E-01	6.195E-01	52.009	0.531	1.549E-01	4.433E-01
ENSG00000189227	C15orf61	270.987	0.271	3.319E-01	6.203E-01	216.771	0.602	1.679E-02	1.328E-01
ENSG00000134758	RNF138	123.766	0.429	3.325E-01	6.205E-01	77.129	1.174	8.729E-04	2.084E-02
ENSG00000028528	SNX1	546.219	-0.258	3.341E-01	6.223E-01	384.531	0.657	2.540E-02	1.685E-01
ENSG00000152256	PDK1	142.117	-0.311	3.354E-01	6.226E-01	129.924	0.874	3.418E-03	4.972E-02
ENSG00000225470	JPX	250.822	0.264	3.347E-01	6.226E-01	193.777	0.552	3.884E-02	2.127E-01
ENSG00000008394	MGST1	1118.340	0.288	3.359E-01	6.229E-01	586.403	0.740	3.593E-03	5.132E-02
ENSG00000144744	UBA3	81.225	-0.375	3.370E-01	6.238E-01	81.118	0.715	3.873E-02	2.124E-01
ENSG00000153037	SRP19	202.925	0.394	3.388E-01	6.259E-01	122.331	0.553	8.794E-02	3.287E-01

ENSG00000145337	PYURF	280.566	0.298	3.429E-01	6.293E-01	251.050	0.530	2.616E-02	1.713E-01
ENSG00000074935	TUBE1	38.788	0.436	3.449E-01	6.312E-01	46.888	1.019	7.580E-03	8.187E-02
ENSG00000213339	QTRT1	74.834	0.366	3.470E-01	6.329E-01	70.320	0.550	1.494E-01	4.367E-01
ENSG00000101367	MAPRE1	996.242	0.297	3.476E-01	6.334E-01	745.733	0.529	1.150E-02	1.058E-01
ENSG00000143862	ARL8A	355.687	0.292	3.483E-01	6.338E-01	508.614	0.779	4.136E-04	1.240E-02
ENSG00000011198	ABHD5	40.423	0.419	3.490E-01	6.338E-01	35.743	0.639	1.094E-01	3.701E-01
ENSG00000164105	SAP30	83.957	0.333	3.518E-01	6.355E-01	98.263	0.691	2.639E-02	1.714E-01
ENSG00000166908	PIP4K2C	318.629	-0.307	3.519E-01	6.355E-01	213.609	0.549	4.946E-02	2.417E-01
ENSG00000205250	E2F4	355.882	0.343	3.526E-01	6.363E-01	279.432	0.510	3.358E-02	1.964E-01
ENSG00000196437	ZNF569	51.052	0.391	3.544E-01	6.383E-01	35.427	0.558	1.645E-01	4.567E-01
ENSG00000151239	TWF1	64.523	0.388	3.573E-01	6.410E-01	76.978	0.760	4.009E-02	2.167E-01
ENSG00000136940	PDCL	134.139	0.313	3.589E-01	6.421E-01	124.611	0.832	6.908E-03	7.739E-02
ENSG00000013561	RNF14	118.040	0.341	3.598E-01	6.433E-01	103.997	0.663	7.017E-02	2.899E-01
ENSG00000146574	CCZ1B	94.722	-0.373	3.616E-01	6.449E-01	81.948	0.625	8.437E-02	3.212E-01
ENSG00000164916	FOXK1	331.542	0.230	3.674E-01	6.512E-01	186.755	0.945	1.611E-03	2.983E-02
ENSG00000244687	UBE2V1	44.173	0.384	3.723E-01	6.564E-01	40.784	0.944	1.611E-02	1.300E-01
ENSG00000087842	PIR	177.896	0.303	3.731E-01	6.571E-01	179.200	0.569	2.627E-02	1.714E-01
ENSG00000138802	SEC24B	95.853	0.322	3.748E-01	6.586E-01	104.220	0.602	7.288E-02	2.959E-01
ENSG00000153179	RASSF3	218.204	0.341	3.747E-01	6.586E-01	82.668	0.816	4.146E-02	2.204E-01
ENSG00000104671	DCTN6	116.429	0.325	3.814E-01	6.641E-01	105.105	0.864	1.390E-02	1.187E-01
ENSG00000110925	CSRNP2	55.801	0.343	3.820E-01	6.649E-01	39.122	0.787	5.713E-02	2.622E-01
ENSG00000064726	BTBD1	46.925	0.366	3.823E-01	6.649E-01	40.188	0.684	9.433E-02	3.406E-01
ENSG00000092871	RFFL	224.578	0.240	3.844E-01	6.664E-01	152.627	0.619	2.273E-02	1.588E-01
ENSG00000274211	SOCS7	553.787	-0.226	3.870E-01	6.686E-01	441.703	0.538	2.874E-02	1.799E-01
ENSG00000223802	CERS1	80.240	0.343	3.874E-01	6.688E-01	32.207	0.674	8.768E-02	3.282E-01
ENSG00000169598	DFFB	95.456	-0.456	3.883E-01	6.694E-01	57.992	0.678	7.450E-02	3.000E-01

ENSG00000137936	BCAR3	38.395	0.413	3.904E-01	6.714E-01	53.676	1.158	1.468E-03	2.810E-02
ENSG00000273611	ZNHIT3	285.436	0.285	3.906E-01	6.715E-01	225.368	0.810	2.356E-03	3.851E-02
ENSG00000128590	DNAJB9	164.491	0.455	3.920E-01	6.728E-01	91.618	1.088	1.600E-03	2.979E-02
ENSG00000123297	TSFM	312.704	-0.303	3.931E-01	6.740E-01	170.936	0.751	3.055E-02	1.865E-01
ENSG00000123094	RASSF8	27.020	0.444	3.958E-01	6.764E-01	31.865	0.796	5.777E-02	2.634E-01
ENSG00000112592	TBP	120.127	0.312	3.965E-01	6.768E-01	86.559	0.645	5.917E-02	2.674E-01
ENSG00000078487	ZCWPW1	33.164	0.402	3.985E-01	6.783E-01	38.009	0.671	9.288E-02	3.381E-01
ENSG00000179588	ZFPM1	49.737	0.352	3.990E-01	6.790E-01	45.127	0.529	1.607E-01	4.510E-01
ENSG00000038274	MAT2B	161.605	0.258	3.992E-01	6.791E-01	136.733	0.561	5.478E-02	2.560E-01
ENSG00000163932	PRKCD	79.739	0.345	4.012E-01	6.800E-01	47.373	0.962	9.982E-03	9.753E-02
ENSG00000165792	METTL17	265.302	0.286	4.083E-01	6.873E-01	204.236	0.760	7.004E-02	2.898E-01
ENSG00000113716	HMGXB3	57.301	0.331	4.097E-01	6.888E-01	92.810	0.943	6.210E-03	7.246E-02
ENSG00000136997	MYC	280.248	0.428	4.145E-01	6.920E-01	154.411	1.378	1.251E-04	5.375E-03
ENSG00000136122	BORA	73.749	-0.366	4.162E-01	6.923E-01	80.202	0.560	1.202E-01	3.900E-01
ENSG00000115128	SF3B6	437.121	0.247	4.179E-01	6.929E-01	406.885	0.530	3.520E-02	2.016E-01
ENSG00000108010	GLRX3	547.351	0.220	4.191E-01	6.939E-01	392.653	0.538	2.320E-02	1.606E-01
ENSG00000169299	PGM2	82.925	0.292	4.194E-01	6.941E-01	86.961	0.672	4.933E-02	2.415E-01
ENSG00000132603	NIP7	271.602	0.228	4.240E-01	6.968E-01	212.080	0.528	4.239E-02	2.227E-01
ENSG00000164086	DUSP7	75.809	-0.353	4.286E-01	6.999E-01	39.411	1.521	9.545E-05	4.435E-03
ENSG00000134480	CCNH	152.706	0.420	4.297E-01	7.007E-01	102.509	1.270	4.047E-04	1.221E-02
ENSG00000067167	TRAM1	668.689	0.216	4.314E-01	7.023E-01	500.020	0.520	2.077E-02	1.514E-01
ENSG00000146963	LUC7L2	444.772	0.226	4.355E-01	7.060E-01	355.221	0.854	9.351E-04	2.168E-02
ENSG00000135002	RFK	301.579	0.234	4.357E-01	7.063E-01	312.172	0.883	7.172E-03	7.879E-02
ENSG00000105186	ANKRD27	145.779	-0.270	4.372E-01	7.075E-01	110.785	0.816	8.277E-03	8.712E-02
ENSG00000088298	EDEM2	120.508	-0.307	4.430E-01	7.123E-01	118.625	0.582	6.138E-02	2.727E-01
ENSG00000151689	INPP1	154.733	-0.232	4.439E-01	7.128E-01	128.948	0.618	3.952E-02	2.150E-01

ENSG00000153044	CENPH	200.453	0.216	4.456E-01	7.140E-01	135.978	0.661	2.705E-02	1.731E-01
ENSG00000165669	FAM204A	150.830	0.219	4.483E-01	7.165E-01	222.738	0.809	1.204E-03	2.514E-02
ENSG00000159658	EFCAB14	187.454	-0.233	4.506E-01	7.180E-01	172.066	0.845	1.707E-03	3.100E-02
ENSG00000204899	MZT1	255.290	0.240	4.634E-01	7.277E-01	156.720	0.892	4.511E-03	5.989E-02
ENSG00000111652	COPS7A	236.941	-0.372	4.687E-01	7.316E-01	149.770	0.544	1.425E-01	4.268E-01
ENSG00000114850	SSR3	2465.867	-0.156	4.701E-01	7.327E-01	1842.821	0.519	8.468E-03	8.846E-02
ENSG00000130772	MED18	80.237	0.274	4.736E-01	7.354E-01	79.055	0.543	9.835E-02	3.493E-01
ENSG00000174749	FAM241A	50.621	0.280	4.774E-01	7.386E-01	42.306	1.084	3.809E-03	5.360E-02
ENSG00000111711	GOLT1B	90.240	0.266	4.797E-01	7.405E-01	122.972	0.572	5.543E-02	2.579E-01
ENSG00000143384	MCL1	1545.331	0.188	4.811E-01	7.421E-01	1292.565	0.534	7.769E-03	8.304E-02
ENSG00000101391	CDK5RAP1	270.188	0.179	4.833E-01	7.428E-01	272.129	0.580	1.326E-02	1.155E-01
ENSG00000049860	HEXB	453.779	0.205	4.892E-01	7.471E-01	299.229	0.542	3.435E-02	1.994E-01
ENSG00000138050	THUMPD2	66.866	0.308	4.921E-01	7.491E-01	74.064	0.584	1.211E-01	3.907E-01
ENSG00000116752	BCAS2	191.369	0.224	4.925E-01	7.494E-01	156.550	0.564	3.717E-02	2.072E-01
ENSG00000251022	THAP9-AS1	91.970	0.237	4.985E-01	7.545E-01	78.007	1.325	1.042E-04	4.675E-03
ENSG00000135127	BICDL1	62.688	-0.259	5.059E-01	7.600E-01	35.827	1.069	9.278E-03	9.337E-02
ENSG00000163866	SMIM12	185.640	-0.232	5.067E-01	7.605E-01	179.419	0.596	2.172E-02	1.544E-01
ENSG00000196116	TDRD7	59.990	-0.347	5.102E-01	7.628E-01	35.231	0.971	1.858E-02	1.420E-01
ENSG00000131507	NDFIP1	267.524	0.187	5.109E-01	7.631E-01	277.959	0.542	3.677E-02	2.063E-01
ENSG00000006704	GTF2IRD1	206.030	0.197	5.106E-01	7.631E-01	189.062	0.649	1.658E-02	1.319E-01
ENSG00000145979	TBC1D7	134.129	-0.241	5.113E-01	7.631E-01	79.404	0.500	1.379E-01	4.196E-01
ENSG00000173540	GMPPB	84.278	0.236	5.148E-01	7.654E-01	63.179	0.525	1.384E-01	4.202E-01
ENSG00000091483	FH	393.492	0.215	5.190E-01	7.691E-01	240.930	0.514	2.195E-01	5.248E-01
ENSG00000166860	ZBTB39	84.946	-0.235	5.218E-01	7.718E-01	85.158	0.630	4.156E-02	2.205E-01
ENSG00000138750	NUP54	104.710	0.272	5.242E-01	7.737E-01	102.670	0.570	1.043E-01	3.614E-01
ENSG00000026103	FAS	44.994	0.289	5.254E-01	7.746E-01	51.691	1.009	8.968E-03	9.125E-02

ENSG00000163874	ZC3H12A	123.643	0.209	5.271E-01	7.759E-01	98.836	1.045	7.756E-04	1.905E-02
ENSG00000198382	UVRAG	229.519	0.185	5.276E-01	7.761E-01	234.442	0.512	2.955E-02	1.823E-01
ENSG00000139517	LNX2	113.992	0.260	5.291E-01	7.769E-01	122.569	0.698	2.135E-02	1.534E-01
ENSG00000233429	HOTAIRM1	30.345	0.287	5.290E-01	7.769E-01	39.516	0.705	6.510E-02	2.793E-01
ENSG00000164687	FABP5	120.277	0.239	5.302E-01	7.774E-01	92.158	0.519	1.341E-01	4.136E-01
ENSG00000141858	SAMD1	950.222	0.200	5.316E-01	7.784E-01	849.659	0.734	6.282E-04	1.636E-02
ENSG00000141452	RMC1	61.364	-0.297	5.326E-01	7.788E-01	36.875	0.768	5.836E-02	2.651E-01
ENSG00000215421	ZNF407	58.785	-0.259	5.329E-01	7.791E-01	55.193	0.546	1.205E-01	3.905E-01
ENSG00000133818	RRAS2	207.287	0.181	5.348E-01	7.800E-01	245.023	0.530	2.910E-02	1.811E-01
ENSG00000101193	GID8	2605.757	0.149	5.376E-01	7.822E-01	2146.548	0.679	2.783E-03	4.311E-02
ENSG00000112511	PHF1	66.283	0.229	5.381E-01	7.823E-01	50.201	0.842	2.592E-02	1.704E-01
ENSG00000050820	BCAR1	262.488	0.181	5.393E-01	7.827E-01	301.028	0.520	1.960E-02	1.472E-01
ENSG00000130173	ANGPTL8	188.569	0.202	5.455E-01	7.871E-01	105.498	0.889	1.630E-02	1.308E-01
ENSG00000186073	C15orf41	50.772	0.259	5.492E-01	7.885E-01	32.076	0.781	4.575E-02	2.321E-01
ENSG00000118242	MREG	86.163	0.261	5.515E-01	7.892E-01	57.391	0.740	3.359E-02	1.964E-01
ENSG00000240509	RPL34P18	91.192	0.218	5.526E-01	7.894E-01	44.749	0.784	4.035E-02	2.176E-01
ENSG00000124688	MAD2L1BP	152.338	0.313	5.556E-01	7.895E-01	81.627	0.856	2.746E-02	1.743E-01
ENSG00000162972	MAIP1	185.834	0.222	5.559E-01	7.897E-01	113.795	0.792	1.314E-02	1.150E-01
ENSG00000244038	DDOST	524.350	0.136	5.596E-01	7.924E-01	458.276	0.539	2.640E-02	1.714E-01
ENSG00000198074	AKR1B10	166.583	-0.246	5.624E-01	7.936E-01	62.084	1.149	4.294E-03	5.820E-02
ENSG00000115170	ACVR1	54.998	0.268	5.656E-01	7.952E-01	75.465	0.825	1.394E-02	1.188E-01
ENSG00000172954	LCLAT1	162.750	0.169	5.674E-01	7.960E-01	106.670	0.759	1.436E-02	1.209E-01
ENSG00000115163	CENPA	299.678	0.146	5.684E-01	7.967E-01	149.258	0.795	6.612E-03	7.511E-02
ENSG00000168275	COA6	314.885	0.281	5.700E-01	7.980E-01	214.537	0.804	3.789E-03	5.353E-02
ENSG00000231940	RPS7P3	66.577	0.227	5.741E-01	8.004E-01	34.667	0.702	7.721E-02	3.057E-01
ENSG00000237190	CDKN2AIPNL	154.480	0.194	5.749E-01	8.005E-01	114.459	0.543	1.040E-01	3.609E-01

ENSG00000152382	TADA1	145.021	0.166	5.814E-01	8.042E-01	100.600	0.602	4.253E-02	2.229E-01
ENSG00000071282	LMCD1	26.783	-0.277	5.823E-01	8.047E-01	74.965	0.549	9.221E-02	3.372E-01
ENSG00000214087	ARL16	55.170	0.241	5.823E-01	8.047E-01	43.957	1.062	6.102E-03	7.178E-02
ENSG00000255529	POLR2M	120.330	0.201	5.827E-01	8.051E-01	97.110	0.520	1.210E-01	3.906E-01
ENSG00000126249	PDCD2L	164.370	0.197	5.842E-01	8.060E-01	135.946	1.169	7.363E-04	1.843E-02
ENSG00000186481	ANKRD20A5P	16.011	0.281	5.868E-01	8.077E-01	47.151	0.597	1.120E-01	3.741E-01
ENSG00000196636	SDHAF3	45.848	0.254	5.927E-01	8.119E-01	48.586	0.644	7.369E-02	2.983E-01
ENSG00000149564	ESAM	63.357	0.230	5.928E-01	8.120E-01	42.654	0.838	3.141E-02	1.891E-01
ENSG00000189143	CLDN4	253.824	0.137	5.963E-01	8.142E-01	220.036	0.837	9.395E-04	2.172E-02
ENSG00000132432	SEC61G	1178.736	0.243	5.963E-01	8.142E-01	907.129	0.950	6.970E-06	5.063E-04
ENSG00000181722	ZBTB20	77.703	0.191	5.988E-01	8.157E-01	153.617	0.525	6.373E-02	2.773E-01
ENSG00000131876	SNRPA1	141.899	0.184	6.019E-01	8.184E-01	115.292	0.781	8.905E-03	9.112E-02
ENSG00000203883	SOX18	52.542	0.234	6.026E-01	8.184E-01	34.199	0.589	1.575E-01	4.479E-01
ENSG00000233971	RPS20P10	22.986	0.256	6.042E-01	8.192E-01	36.377	0.632	1.134E-01	3.767E-01
ENSG00000166889	PATL1	311.899	-0.161	6.050E-01	8.197E-01	295.469	0.972	2.325E-04	8.299E-03
ENSG00000231312	AC007388.1	110.720	-0.201	6.051E-01	8.197E-01	100.720	0.698	3.236E-02	1.922E-01
ENSG00000179051	RCC2	826.713	0.273	6.055E-01	8.200E-01	264.629	0.680	3.971E-02	2.156E-01
ENSG00000137575	SDCBP	354.052	-0.169	6.074E-01	8.205E-01	338.513	0.693	2.667E-02	1.721E-01
ENSG00000168538	TRAPPC11	68.996	0.198	6.212E-01	8.290E-01	80.975	0.546	8.253E-02	3.178E-01
ENSG00000198855	FICD	77.375	-0.181	6.244E-01	8.305E-01	53.439	0.594	9.975E-02	3.526E-01
ENSG00000120526	NUDCD1	112.645	0.193	6.250E-01	8.309E-01	91.256	0.508	9.202E-02	3.369E-01
ENSG00000153391	INO80C	67.135	0.208	6.285E-01	8.324E-01	66.997	0.926	8.773E-03	9.041E-02
ENSG00000101413	RPRD1B	319.010	0.128	6.297E-01	8.331E-01	225.682	0.553	2.484E-02	1.668E-01
ENSG00000203485	INF2	112.884	0.174	6.394E-01	8.389E-01	86.886	0.716	2.441E-02	1.656E-01
ENSG00000157214	STEAP2	86.526	-0.172	6.401E-01	8.394E-01	73.645	0.535	1.057E-01	3.631E-01
ENSG00000023318	ERP44	355.081	0.151	6.411E-01	8.399E-01	256.559	0.518	5.659E-02	2.605E-01

ENSG00000115233	PSMD14	507.343	0.113	6.431E-01	8.409E-01	506.679	0.651	1.386E-03	2.708E-02
ENSG00000130147	SH3BP4	632.810	0.120	6.439E-01	8.412E-01	427.275	0.563	1.325E-02	1.155E-01
ENSG00000185269	NOTUM	590.526	0.243	6.453E-01	8.414E-01	386.892	0.750	7.295E-02	2.959E-01
ENSG00000134419	RPS15A	6558.310	0.131	6.470E-01	8.421E-01	6136.382	0.521	2.658E-03	4.173E-02
ENSG00000164828	SUN1	281.299	-0.121	6.503E-01	8.441E-01	191.010	0.723	5.828E-03	6.970E-02
ENSG00000150787	PTS	128.380	0.164	6.508E-01	8.445E-01	91.974	1.134	1.678E-03	3.058E-02
ENSG00000137767	SQOR	132.451	0.139	6.552E-01	8.466E-01	105.090	0.601	6.475E-02	2.787E-01
ENSG00000163374	YY1AP1	219.735	0.114	6.559E-01	8.470E-01	261.366	0.603	1.008E-02	9.784E-02
ENSG00000227036	LINC00511	116.301	0.138	6.562E-01	8.471E-01	165.818	0.605	1.924E-02	1.452E-01
ENSG00000129657	SEC14L1	374.058	-0.134	6.591E-01	8.480E-01	307.357	0.514	4.039E-02	2.177E-01
ENSG00000073712	FERMT2	840.832	0.104	6.597E-01	8.484E-01	512.105	0.681	1.418E-03	2.744E-02
ENSG00000182117	NOP10	1008.283	0.111	6.602E-01	8.484E-01	929.129	0.650	2.836E-03	4.365E-02
ENSG00000112242	E2F3	491.708	0.103	6.619E-01	8.492E-01	341.040	0.527	2.471E-02	1.666E-01
ENSG00000110218	PANX1	71.173	0.173	6.659E-01	8.515E-01	83.036	0.794	2.776E-02	1.754E-01
ENSG00000076053	RBM7	66.926	0.181	6.672E-01	8.520E-01	52.637	0.511	1.597E-01	4.505E-01
ENSG00000066855	MTFR1	146.305	-0.163	6.727E-01	8.554E-01	159.341	0.711	8.725E-03	9.006E-02
ENSG00000141068	KSR1	102.360	-0.164	6.755E-01	8.567E-01	140.318	0.561	4.605E-02	2.331E-01
ENSG00000143811	PYCR2	488.316	0.143	6.760E-01	8.571E-01	329.278	0.821	7.994E-03	8.492E-02
ENSG00000119912	IDE	181.752	0.117	6.788E-01	8.581E-01	186.045	0.549	4.004E-02	2.167E-01
ENSG00000092445	TYRO3	170.813	0.125	6.813E-01	8.594E-01	112.397	0.547	7.137E-02	2.925E-01
ENSG00000110429	FBXO3	107.415	0.130	6.816E-01	8.595E-01	93.532	0.521	8.969E-02	3.317E-01
ENSG00000106701	FSD1L	27.480	0.198	6.824E-01	8.600E-01	40.142	0.716	8.004E-02	3.127E-01
ENSG00000092208	GEMIN2	85.469	-0.147	6.845E-01	8.610E-01	60.339	0.542	1.475E-01	4.342E-01
ENSG00000198874	TYW1	108.928	0.134	6.914E-01	8.655E-01	91.794	0.622	4.829E-02	2.389E-01
ENSG00000131941	RHPN2	85.718	0.177	6.937E-01	8.657E-01	56.672	0.732	4.173E-02	2.207E-01
ENSG00000138617	PARP16	48.156	0.172	6.947E-01	8.663E-01	44.099	0.573	1.538E-01	4.422E-01

ENSG00000106484	MEST	165.752	-0.163	6.969E-01	8.677E-01	109.565	0.504	1.799E-01	4.772E-01
ENSG00000131759	RARA	112.119	0.151	7.018E-01	8.699E-01	85.927	0.570	1.328E-01	4.114E-01
ENSG00000144182	LIPT1	18.537	0.195	7.042E-01	8.715E-01	35.098	0.569	1.412E-01	4.250E-01
ENSG00000162676	GFI1	24.716	-0.191	7.094E-01	8.741E-01	44.738	0.652	1.012E-01	3.558E-01
ENSG00000187118	CMC1	136.277	0.137	7.101E-01	8.746E-01	120.450	0.767	1.163E-02	1.068E-01
ENSG00000175104	TRAF6	39.046	0.167	7.170E-01	8.786E-01	47.324	0.581	1.285E-01	4.041E-01
ENSG00000269834	ZNF528-AS1	53.066	0.154	7.228E-01	8.812E-01	42.812	0.816	3.335E-02	1.959E-01
ENSG00000250317	SMIM20	94.409	-0.148	7.247E-01	8.827E-01	87.162	0.604	6.698E-02	2.842E-01
ENSG00000164211	STARD4	57.165	0.138	7.259E-01	8.835E-01	80.021	0.546	1.138E-01	3.778E-01
ENSG00000167904	TMEM68	55.282	0.145	7.259E-01	8.835E-01	42.957	0.524	1.704E-01	4.654E-01
ENSG00000141198	TOM1L1	134.274	0.106	7.271E-01	8.842E-01	128.235	0.549	6.881E-02	2.873E-01
ENSG00000106537	TSPAN13	35.108	0.158	7.280E-01	8.850E-01	30.436	0.571	1.628E-01	4.536E-01
ENSG00000151151	IPMK	142.824	0.106	7.352E-01	8.893E-01	77.811	0.545	1.141E-01	3.782E-01
ENSG00000124641	MED20	144.092	-0.109	7.363E-01	8.903E-01	154.677	0.730	1.226E-02	1.104E-01
ENSG00000132964	CDK8	79.511	0.139	7.363E-01	8.903E-01	80.199	0.565	8.433E-02	3.212E-01
ENSG00000167771	RCOR2	99.003	-0.151	7.370E-01	8.907E-01	63.884	1.029	1.135E-02	1.050E-01
ENSG00000147889	CDKN2A	52.532	-0.145	7.371E-01	8.907E-01	32.075	0.862	3.488E-02	2.006E-01
ENSG00000180488	MIGA1	41.773	0.153	7.371E-01	8.907E-01	71.476	0.693	5.903E-02	2.669E-01
ENSG00000147789	ZNF7	60.027	-0.135	7.406E-01	8.924E-01	58.918	0.693	5.568E-02	2.584E-01
ENSG00000109861	CTSC	263.991	-0.118	7.420E-01	8.930E-01	154.569	0.767	2.143E-02	1.536E-01
ENSG00000120686	UFM1	208.154	0.125	7.425E-01	8.934E-01	233.043	0.701	5.908E-03	7.037E-02
ENSG00000167543	TP53I13	146.623	0.104	7.430E-01	8.934E-01	151.675	0.610	4.383E-02	2.276E-01
ENSG00000130312	MRPL34	718.469	0.105	7.459E-01	8.949E-01	619.742	0.518	2.809E-02	1.769E-01
ENSG00000158079	PTPDC1	58.150	0.131	7.469E-01	8.954E-01	79.846	0.597	8.596E-02	3.243E-01
ENSG00000115514	TXNDC9	162.646	-0.110	7.473E-01	8.955E-01	154.457	0.600	3.821E-02	2.108E-01
ENSG00000272886	DCP1A	161.660	0.105	7.476E-01	8.957E-01	161.511	0.608	3.608E-02	2.038E-01

ENSG00000135047	CTSL	184.078	0.166	7.489E-01	8.961E-01	121.644	0.559	1.215E-01	3.910E-01
ENSG00000188612	SUMO2	193.331	-0.110	7.516E-01	8.970E-01	180.898	0.510	5.157E-02	2.481E-01
ENSG00000102393	GLA	57.543	0.142	7.530E-01	8.970E-01	60.533	0.755	3.760E-02	2.087E-01
ENSG00000105290	APLP1	35.689	0.143	7.533E-01	8.970E-01	37.346	0.634	1.093E-01	3.700E-01
ENSG00000159433	STARD9	69.576	-0.125	7.563E-01	8.984E-01	69.643	0.558	1.094E-01	3.701E-01
ENSG00000066583	ISOC1	253.793	-0.102	7.596E-01	8.995E-01	172.937	1.031	2.931E-03	4.474E-02
ENSG00000170634	ACYP2	48.862	0.122	7.652E-01	9.018E-01	46.545	0.518	1.876E-01	4.870E-01
ENSG00000172071	EIF2AK3	124.129	0.100	7.686E-01	9.032E-01	102.420	0.523	1.503E-01	4.377E-01
ENSG00000096746	HNRNPH3	930.146	-0.136	7.697E-01	9.036E-01	531.762	0.677	1.096E-02	1.029E-01
ENSG00000156374	PCGF6	43.668	0.135	7.732E-01	9.054E-01	31.981	0.839	3.831E-02	2.112E-01
ENSG00000070081	NUCB2	491.570	0.072	7.739E-01	9.055E-01	426.274	0.608	8.329E-03	8.740E-02
ENSG00000101452	DHX35	83.964	-0.115	7.744E-01	9.059E-01	85.923	0.550	9.385E-02	3.399E-01
ENSG00000117650	NEK2	187.159	0.080	7.750E-01	9.063E-01	174.757	0.660	2.498E-02	1.668E-01
ENSG00000120685	PROSER1	200.363	0.075	7.809E-01	9.092E-01	150.811	0.578	4.827E-02	2.389E-01
ENSG00000237550	RPL9P9	136.022	0.146	7.846E-01	9.116E-01	70.977	0.760	4.747E-02	2.374E-01
ENSG00000099246	RAB18	491.554	0.072	7.852E-01	9.119E-01	496.079	0.513	1.066E-02	1.011E-01
ENSG00000090975	PITPNM2	91.383	0.099	7.865E-01	9.128E-01	71.784	0.516	1.342E-01	4.137E-01
ENSG00000136810	TXN	1550.272	0.137	7.875E-01	9.131E-01	822.629	0.611	1.371E-01	4.182E-01
ENSG00000175711	B3GNTL1	41.780	0.119	7.878E-01	9.132E-01	44.620	0.515	1.805E-01	4.780E-01
ENSG00000169902	TPST1	74.358	0.107	7.892E-01	9.136E-01	60.801	0.600	1.050E-01	3.621E-01
ENSG00000172732	MUS81	86.752	-0.106	7.936E-01	9.154E-01	78.728	0.797	2.350E-02	1.621E-01
ENSG00000107566	ERLIN1	625.612	0.089	7.953E-01	9.162E-01	343.846	0.518	9.349E-02	3.392E-01
ENSG00000180228	PRKRA	203.558	0.100	8.003E-01	9.181E-01	227.041	0.543	3.186E-02	1.907E-01
ENSG00000116213	WRAP73	151.726	0.097	8.069E-01	9.198E-01	123.662	0.602	9.519E-02	3.426E-01
ENSG00000156026	MCU	241.053	0.067	8.077E-01	9.202E-01	233.739	0.536	2.654E-02	1.717E-01
ENSG00000158941	CCAR2	61.303	-0.096	8.128E-01	9.226E-01	57.886	0.566	1.089E-01	3.691E-01

ENSG00000086065	CHMP5	389.888	0.075	8.178E-01	9.247E-01	290.443	0.624	3.991E-02	2.163E-01
ENSG00000027001	MIPEP	94.555	-0.085	8.235E-01	9.269E-01	75.002	0.877	1.193E-02	1.087E-01
ENSG00000247844	CCAT1	32.524	-0.111	8.262E-01	9.280E-01	55.714	0.637	7.509E-02	3.015E-01
ENSG00000147119	CHST7	26.779	-0.110	8.274E-01	9.286E-01	30.364	0.775	5.193E-02	2.487E-01
ENSG00000141543	EIF4A3	1009.095	-0.068	8.303E-01	9.302E-01	910.886	0.508	7.322E-03	7.959E-02
ENSG00000050130	JKAMP	141.674	-0.113	8.322E-01	9.310E-01	70.214	0.844	3.383E-02	1.976E-01
ENSG00000131115	ZNF227	73.124	0.104	8.325E-01	9.311E-01	37.723	0.996	1.403E-02	1.194E-01
ENSG00000167977	KCTD5	181.099	0.109	8.343E-01	9.317E-01	84.339	0.847	1.586E-02	1.290E-01
ENSG00000178971	CTC1	43.963	-0.088	8.356E-01	9.327E-01	65.264	0.509	1.638E-01	4.550E-01
ENSG00000125821	DTD1	587.955	0.064	8.392E-01	9.345E-01	460.208	0.671	2.901E-02	1.811E-01
ENSG00000166716	ZNF592	69.614	0.074	8.421E-01	9.357E-01	64.634	0.573	1.057E-01	3.631E-01
ENSG00000178202	KDELC2	48.240	0.088	8.423E-01	9.357E-01	73.308	0.587	8.420E-02	3.209E-01
ENSG00000204267	TAP2	423.381	-0.050	8.441E-01	9.368E-01	308.918	0.548	6.471E-02	2.787E-01
ENSG00000123836	PFKFB2	70.895	0.075	8.441E-01	9.368E-01	62.902	0.577	1.007E-01	3.546E-01
ENSG00000213995	NAXD	88.080	-0.084	8.443E-01	9.369E-01	104.678	0.616	5.265E-02	2.509E-01
ENSG00000104221	BRF2	50.337	-0.089	8.446E-01	9.371E-01	53.504	0.827	2.778E-02	1.754E-01
ENSG00000175110	MRPS22	273.427	0.070	8.461E-01	9.374E-01	170.033	0.872	1.460E-02	1.221E-01
ENSG00000131871	SELENOS	632.083	-0.040	8.479E-01	9.382E-01	460.160	0.569	1.252E-02	1.122E-01
ENSG00000134996	OSTF1	63.160	-0.079	8.497E-01	9.391E-01	54.782	0.528	1.759E-01	4.715E-01
ENSG00000106554	CHCHD3	238.361	0.067	8.634E-01	9.450E-01	145.054	0.718	1.578E-02	1.285E-01
ENSG00000132581	SDF2	168.088	-0.055	8.688E-01	9.470E-01	146.178	0.567	6.098E-02	2.721E-01
ENSG00000130699	TAF4	129.803	0.058	8.690E-01	9.470E-01	122.916	0.672	2.098E-02	1.522E-01
ENSG00000167005	NUDT21	888.799	-0.042	8.705E-01	9.474E-01	623.795	0.578	2.639E-02	1.714E-01
ENSG00000175376	EIF1AD	147.283	0.054	8.734E-01	9.489E-01	86.607	0.623	7.243E-02	2.948E-01
ENSG00000204335	SP5	1812.173	-0.076	8.800E-01	9.522E-01	750.313	0.525	1.816E-01	4.803E-01
ENSG00000133731	IMPA1	123.670	-0.051	8.851E-01	9.538E-01	109.687	0.789	8.707E-03	9.006E-02

ENSG00000108671	PSMD11	2053.869	-0.037	8.872E-01	9.549E-01	1687.249	0.687	2.119E-04	7.749E-03
ENSG00000101544	ADNP2	404.761	0.036	8.949E-01	9.580E-01	407.375	0.586	1.271E-02	1.131E-01
ENSG00000070761	CFAP20	350.302	0.043	9.002E-01	9.606E-01	241.463	0.627	1.332E-01	4.121E-01
ENSG00000198393	ZNF26	53.807	0.051	9.004E-01	9.606E-01	45.942	0.642	8.699E-02	3.267E-01
ENSG00000271601	LIX1L	103.596	-0.055	9.010E-01	9.606E-01	78.341	0.648	7.814E-02	3.075E-01
ENSG00000122882	ECD	234.320	-0.039	9.018E-01	9.609E-01	181.523	0.655	2.096E-02	1.521E-01
ENSG00000134056	MRPS36	238.582	0.041	9.028E-01	9.609E-01	215.932	0.587	5.810E-02	2.643E-01
ENSG00000075131	TIPIN	99.499	0.043	9.029E-01	9.609E-01	58.533	0.519	1.542E-01	4.423E-01
ENSG00000138495	COX17	755.801	0.034	9.058E-01	9.624E-01	534.061	0.701	8.442E-03	8.831E-02
ENSG00000105732	ZNF574	57.745	0.048	9.090E-01	9.631E-01	91.821	0.796	2.163E-02	1.544E-01
ENSG00000118197	DDX59	72.055	0.049	9.103E-01	9.632E-01	73.099	0.677	5.405E-02	2.540E-01
ENSG00000139343	SNRPF	1385.009	-0.033	9.131E-01	9.650E-01	869.751	0.507	5.251E-02	2.508E-01
ENSG00000172932	ANKRD13D	49.695	-0.042	9.176E-01	9.670E-01	69.127	0.708	4.892E-02	2.403E-01
ENSG00000102471	NDFIP2	126.079	0.035	9.182E-01	9.673E-01	107.853	0.656	4.275E-02	2.237E-01
ENSG00000149923	PPP4C	207.209	0.053	9.205E-01	9.674E-01	108.348	0.830	2.647E-02	1.716E-01
ENSG00000076604	TRAF4	375.093	0.024	9.238E-01	9.694E-01	313.746	0.559	1.551E-02	1.273E-01
ENSG00000159399	HK2	251.522	0.030	9.261E-01	9.704E-01	140.170	0.911	1.279E-02	1.132E-01
ENSG00000170222	ADPRM	23.995	-0.041	9.330E-01	9.731E-01	34.633	0.546	1.696E-01	4.642E-01
ENSG00000155959	VBP1	194.026	-0.023	9.334E-01	9.731E-01	183.655	0.513	4.075E-02	2.185E-01
ENSG00000115840	SLC25A12	39.395	0.040	9.327E-01	9.731E-01	38.706	0.506	1.906E-01	4.903E-01
ENSG00000175606	TMEM70	201.133	0.027	9.370E-01	9.744E-01	140.110	0.540	7.553E-02	3.023E-01
ENSG00000088179	PTPN4	65.533	-0.032	9.373E-01	9.746E-01	78.349	0.579	7.295E-02	2.959E-01
ENSG00000158158	CNNM4	68.547	0.030	9.396E-01	9.756E-01	54.094	1.416	1.417E-04	5.756E-03
ENSG00000143486	EIF2D	231.790	0.027	9.431E-01	9.773E-01	150.654	0.516	1.302E-01	4.074E-01
ENSG00000171723	GPHN	75.869	0.027	9.458E-01	9.783E-01	85.877	0.621	5.689E-02	2.614E-01
ENSG00000102580	DNAJC3	484.963	0.014	9.496E-01	9.802E-01	352.466	0.645	8.490E-03	8.855E-02

ENSG00000213341	CHUK	153.260	-0.025	9.504E-01	9.803E-01	110.814	1.056	6.928E-04	1.759E-02
ENSG00000158122	AAED1	127.947	-0.021	9.521E-01	9.809E-01	93.309	0.545	1.092E-01	3.698E-01
ENSG00000253833	AC022868.1	34.371	0.027	9.577E-01	9.832E-01	30.922	0.574	1.567E-01	4.470E-01
ENSG00000247498	GPRC5D-AS1	41.874	-0.022	9.606E-01	9.834E-01	49.868	0.619	1.056E-01	3.631E-01
ENSG00000140577	CRTC3	145.636	0.017	9.600E-01	9.834E-01	176.339	0.565	6.053E-02	2.712E-01
ENSG00000121579	NAA50	934.373	-0.010	9.636E-01	9.848E-01	779.877	0.507	1.481E-02	1.233E-01
ENSG00000123728	RAP2C	115.082	-0.015	9.647E-01	9.852E-01	72.390	0.510	1.355E-01	4.159E-01
ENSG00000179598	PLD6	94.891	-0.016	9.659E-01	9.858E-01	84.727	0.513	1.213E-01	3.907E-01
ENSG00000114648	KLHL18	105.686	-0.014	9.661E-01	9.858E-01	98.167	0.722	3.107E-02	1.880E-01
ENSG00000138785	INTS12	87.601	0.013	9.746E-01	9.893E-01	132.789	0.513	1.012E-01	3.558E-01
ENSG00000117519	CNN3	593.714	0.010	9.753E-01	9.895E-01	366.364	0.715	8.227E-03	8.689E-02
ENSG00000072401	UBE2D1	111.484	-0.012	9.763E-01	9.900E-01	107.278	0.503	1.163E-01	3.823E-01
ENSG00000188215	DCUN1D3	110.329	0.009	9.802E-01	9.915E-01	70.160	0.540	1.324E-01	4.108E-01
ENSG00000139197	PEX5	93.681	-0.008	9.849E-01	9.934E-01	61.710	0.569	1.107E-01	3.722E-01
ENSG00000140939	NOL3	37.455	0.009	9.861E-01	9.938E-01	37.191	0.739	5.953E-02	2.688E-01
ENSG00000127334	DYRK2	95.611	-0.003	9.923E-01	9.971E-01	140.054	0.532	5.347E-02	2.528E-01
ENSG00000118046	STK11	71.332	-0.004	9.924E-01	9.971E-01	77.650	0.617	9.044E-02	3.336E-01
ENSG00000205937	RNPS1	643.478	0.002	9.946E-01	9.980E-01	609.989	0.596	1.449E-02	1.214E-01
ENSG00000168994	PXDC1	147.540	-0.003	9.948E-01	9.980E-01	78.554	0.798	2.485E-02	1.668E-01
ENSG00000077721	UBE2A	289.691	0.000	9.994E-01	9.995E-01	238.164	0.528	2.733E-02	1.740E-01
ENSG00000115009	CCL20	204.771	-1.168	NA	NA	42.286	0.448	2.795E-01	5.905E-01

(Direct) predicted targets

Table S14. STRING clusters analysis of targets down-regulated by miR-512-3p.

Cluster number	Protein name
1	ABCG8
1	ACVR1
1	ACYP2
1	AGFG2
1	AHDC1
1	ANAPC1
1	ANKRD26
1	ANKRD27
1	ANKS1A
1	AREL1
1	ARHGAP1
1	ARHGAP18
1	ARHGAP19
1	ARID3A
1	ARL13B
1	ARVCF
1	ATAD5
1	ATOH8
1	ATXN7L3B
1	BCL7B
1	BIRC5
1	BORA
1	C18orf8
1	C19orf25
1	C21orf33
1	C2orf48
1	C4orf29
1	C6orf120
1	CAPZA2
1	CAPZB
1	CBLL1
1	CBWD1
1	CBX2
1	CCDC138
1	CCDC18
1	CCDC64
1	CCDC77
1	CCDC85C
1	CCNB1
1	CCNF
1	CCNY
1	CCZ1B

1	CDCA3
1	CDK19
1	CENPA
1	CENPF
1	CENPH
1	CENPJ
1	CEP83
1	CHMP5
1	CHTF8
1	CIT
1	CNOT8
1	COLCA2
1	CORO2A
1	CRIP3
1	CRIPAK
1	CSTF1
1	DAB2IP
1	DCAF16
1	DCTN6
1	DCUN1D1
1	DDX11
1	DEPDC1B
1	DHRS7
1	DIAPH1
1	DLGAP5
1	DNAL4
1	DSN1
1	DYNLL1
1	ELK1
1	ELMOD2
1	EME1
1	ERCC6L
1	ERV3-1
1	ESCO2
1	ESPL1
1	FAM213B
1	FAM222A
1	FAM64A
1	FAM83D
1	FANCC
1	FANCG
1	FANCL
1	FANCM
1	FBXL18
1	FBXO21

1	FBXO28
1	FBXO3
1	FBXO9
1	FCHO2
1	FEN1
1	FGFR1OP
1	FKBPL
1	FOXK1
1	FSD1L
1	FZD4
1	GINS1
1	GINS3
1	GPSM2
1	GRTP1
1	GTF2IRD1
1	HAUS3
1	HEATR2
1	HEG1
1	HEXIM2
1	HIP1
1	HPN
1	IFT74
1	IGF2BP3
1	IKBIP
1	INO80C
1	INTS12
1	INTS9
1	ITPRIPL2
1	KDELC2
1	KDM2A
1	KIAA0586
1	KIF13B
1	KIF18B
1	KIF20A
1	KIF7
1	KIFC1
1	KLHL26
1	KLHL28
1	KNSTRN
1	KNTC1
1	LGR4
1	LIN52
1	LIN54
1	LLGL1
1	LMCD1

1	LRRC45
1	MAD2L1BP
1	MAPRE1
1	MARCH8
1	MARCH9
1	MASTL
1	MAU2
1	MCM10
1	MCM3
1	MCPH1
1	MED17
1	MED18
1	MED20
1	MED29
1	MIS18A
1	MOB1A
1	MON1B
1	MRAS
1	MYH10
1	MYRF
1	NAB2
1	NDC80
1	NDEL1
1	NEK2
1	NINL
1	NOTUM
1	NUP37
1	OPN3
1	ORC6
1	OSTF1
1	PBK
1	PDLIM5
1	PHF1
1	PKD2
1	PKN3
1	PLEKHG6
1	PPAPDC2
1	PPP4C
1	PRC1
1	PRIM1
1	PTPDC1
1	RAB21
1	RAB22A
1	RAB31
1	RABL2A

1	RABL2B
1	RAD23B
1	RAD51C
1	RAP1GAP
1	RAP2A
1	RBBP7
1	RCC2
1	REEP6
1	RFFL
1	RFX5
1	RHPN2
1	RIN1
1	RMI2
1	RNF138
1	RNF14
1	RNF38
1	RNF43
1	RNF6
1	RPA3
1	RRAGC
1	RRM2
1	SAMD1
1	SAP30
1	SDCCAG3
1	SEC14L1
1	SGOL1
1	SHKBP1
1	SHROOM1
1	SLC25A24
1	SLC35E2B
1	SLC38A7
1	SMARCA1
1	SNTB1
1	SNX14
1	SPAG16
1	SPG11
1	STIL
1	TADA1
1	TAP2
1	TBX2
1	TESK1
1	TMEM138
1	TMEM45B
1	TMEM50B
1	TOP2B

1	TRAFD1
1	TRIM37
1	TRIM52
1	TRIM66
1	TRPV2
1	TSC22D4
1	TTC21B
1	TUBA1B
1	TUBE1
1	TUBG1
1	UBBP4
1	UBE2T
1	UBE4B
1	UHMK1
1	UHRF1
1	UNC119B
1	UVRAG
1	VPRBP
1	VWA9
1	WDR19
1	WDR27
1	WDR42A
1	WWC2
1	ZBTB33
1	ZBTB39
1	ZBTB9
1	ZCWPW1
1	ZDHHC21
1	ZDHHC23
1	ZNF227
1	ZNF32
1	ZNF354A
1	ZNF362
1	ZNF572
1	ZNF587B
1	ZNF747
2	AFP
2	AGGF1
2	AGO4
2	ALB
2	ALDH18A1
2	AP3B1
2	AP4E1
2	APEX1
2	ARF6

2	ASRGL1
2	BACE1
2	BCAR1
2	BIRC3
2	BPTF
2	BRCA2
2	BRD3
2	CASP9
2	CAT
2	CCAR2
2	CCDC58
2	CCNH
2	CD9
2	CDK12
2	CDK6
2	CDK8
2	CDKN1B
2	CDKN2A
2	CDKN2AIPNL
2	CDR2
2	CHEK2
2	CHKA
2	CHUK
2	CLN3
2	COG2
2	COPS7A
2	CREB1
2	CSRNP2
2	CTTN
2	CUL1
2	CUL3
2	CYP19A1
2	DAD1
2	DDHD1
2	DGCR8
2	DKK1
2	DLG1
2	DMAP1
2	DNA2
2	DNMT3A
2	DUSP6
2	E2F2
2	E2F3
2	E2F4
2	E2F5

2	EEF1B2
2	EIF2AK3
2	EIF4E
2	EPO
2	ESRRA
2	ETAA1
2	EWSR1
2	FADD
2	FAS
2	FERMT2
2	FGFR1
2	FH
2	FHL2
2	FMR1
2	GFI1
2	GGH
2	GID4
2	GLIS2
2	GNB5
2	GNPTG
2	GOLPH3
2	GPHN
2	GPS2
2	H2AFX
2	HCFC1
2	HIP1R
2	HIPK2
2	HK2
2	HMGCR
2	HOMER1
2	HSPA8
2	HTRA2
2	ID1
2	IDE
2	IDH1
2	IGF2BP1
2	IGF2R
2	INF2
2	INSR
2	INTS2
2	IRS1
2	KDELR2
2	KDM4A
2	KDM5B
2	KDM7A

2	KSR1
2	LBR
2	LCLAT1
2	LONP2
2	LOX
2	MACF1
2	MAP2K6
2	MAP3K7
2	MAPK3
2	MAPKAPK5
2	MAT2A
2	MAT2B
2	MCL1
2	MED1
2	METTL3
2	MLXIPL
2	MOCS3
2	MORF4L2
2	MTHFR
2	MYC
2	MYD88
2	NCOA2
2	NDRG1
2	NES
2	NKX3-1
2	NR1H3
2	NR1H4
2	NR2F1
2	NUP210
2	PARVA
2	PAX6
2	PBRM1
2	PCK1
2	PDE3B
2	PDK1
2	PEX5
2	PFN1
2	PIK3C3
2	PIK3CB
2	PIM2
2	PITHD1
2	PLCB3
2	PRKAR1A
2	PRKCD
2	PRKCI

2	PRNP
2	PROM1
2	PSAT1
2	PSEN1
2	PSMA3
2	PSMA4
2	PSMD11
2	PSMD14
2	RANBP9
2	RARA
2	RB1
2	RBL1
2	RBL2
2	RCC1
2	RNF123
2	RNF126
2	RNF2
2	RPTOR
2	RRAS2
2	SDC1
2	SDCBP
2	SDHC
2	SENP1
2	SERPINE1
2	SETD1B
2	SIRT5
2	SKP2
2	SLC25A12
2	SLC45A3
2	SMAD2
2	SMEK2
2	SNX1
2	SORL1
2	SORT1
2	SOX9
2	SP4
2	SREBF1
2	SRSF1
2	STK11
2	STX16
2	SUCLG2
2	SUFU
2	SUMO2
2	SYNE2
2	TAF1

2	TBP
2	TCF7L1
2	TFDP1
2	TFPI
2	TGOLN2
2	TJP2
2	TMEM209
2	TMX1
2	TOPBP1
2	TRADD
2	TRAF6
2	TRIM25
2	TXN
2	TXNDC9
2	UBA3
2	UBE2A
2	UBE2D1
2	UBE2G2
2	UBE2J1
2	UBE2V1
2	UBL4A
2	UFM1
2	USP22
2	UTRN
2	VPS26A
2	VPS37A
2	VPS41
2	WAC
2	WRAP73
2	ZYG11B
3	ABHD17B
3	AIDA
3	AIF1L
3	AK4
3	ALKBH3
3	ALKBH6
3	ALMS1
3	ANKRD37
3	APBB2
3	APEX2
3	APLP1
3	APOL2
3	APPBP2
3	AQR
3	ARL16

3	ASB7
3	ATE1
3	ATF1
3	ATP11A
3	BCAS2
3	BCORL1
3	BRF2
3	BYSL
3	C10orf2
3	C11orf30
3	C11orf73
3	C15orf61
3	C18orf21
3	C3orf17
3	CAMKMT
3	CCDC137
3	CCDC28A
3	CCDC86
3	CCNJ
3	CDC37L1
3	CFAP20
3	CGGBP1
3	CGI-74
3	CLEC16A
3	CLK2
3	CMC1
3	CNN3
3	COIL
3	CREBL2
3	CTC1
3	CTDSPL
3	DCP1A
3	DCUN1D3
3	DDX10
3	DDX23
3	DDX28
3	DDX55
3	DDX59
3	DEPDC7
3	DEXI
3	DHX32
3	DHX35
3	DHX40
3	DNAJA3
3	DOCK4

3	DQX1
3	DTD1
3	DTX4
3	DTYMK
3	DYRK2
3	EFCAB11
3	EFCAB14
3	EIF1B
3	EIF2D
3	EIF3J
3	EIF4A3
3	EMILIN2
3	ERCC4
3	ESF1
3	ESRP2
3	EXOSC2
3	FAM120AOS
3	FAM149B1
3	FAM169A
3	FAM178A
3	FAM8A1
3	FBXO42
3	FBXW9
3	FICD
3	FNTB
3	FRMD8
3	FRYL
3	GAS2
3	GEMIN2
3	GID8
3	GMEB2
3	GTF2H5
3	GTF3C2
3	HELZ
3	HNRNPA3
3	HNRNPH3
3	HNRNPM
3	IMP3
3	ITM2B
3	ITM2C
3	JMJD4
3	JRKL
3	KANK2
3	KATNAL1
3	KIAA1147

3	LEMD2
3	MDN1
3	MEST
3	METTL1
3	METTL10
3	METTL2B
3	METTL7B
3	MKI67
3	MREG
3	MTAP
3	MTG1
3	MUS81
3	MYEF2
3	MZT1
3	NAA50
3	NAV2
3	NCOA5
3	NGDN
3	NHLRC3
3	NIFK
3	NIP7
3	NME7
3	NOL7
3	NOLC1
3	NOP10
3	NPR2
3	NPTXR
3	NR2C2AP
3	NUDCD1
3	NUDT16
3	NUDT21
3	NUP54
3	OCEL1
3	OMA1
3	PAN2
3	PAN3
3	PARP16
3	PASK
3	PATL1
3	PAXIP1
3	PCGF6
3	PDCD2L
3	PDE6D
3	PHF8
3	PLD6

3	PNRC2
3	POC1A
3	POLR1A
3	POLR2M
3	POLR3A
3	POLR3H
3	POLR3K
3	POP4
3	PPA2
3	PPAN
3	PPIH
3	PPIL2
3	PRIMPOL
3	PRKRA
3	PRMT6
3	PRODH2
3	PROSER1
3	PRUNE
3	PSMD10
3	PTCD2
3	PUS7L
3	RANBP6
3	RANGRF
3	RBM10
3	RBM27
3	RBM41
3	RBM7
3	RBMXL1
3	RCL1
3	RCOR2
3	RCOR3
3	REPIN1
3	RFWD3
3	RIPPLY3
3	RMDN1
3	RMND5A
3	RNPS1
3	RPAP1
3	RPF1
3	RPL27
3	RPL34
3	RPP14
3	RPP25L
3	RPRD1B
3	RPS15A

3	RQCD1
3	RRP1B
3	RRP7A
3	RRS1
3	RTCA
3	RUFY1
3	RUFY2
3	S100A13
3	SENP2
3	SETD6
3	SF3B6
3	SLC23A2
3	SLC25A16
3	SLC25A42
3	SLC25A46
3	SLC2A4RG
3	SLC35E3
3	SMG6
3	SMIM12
3	SMU1
3	SNRPA1
3	SNRPB2
3	SNRPF
3	SP5
3	SPICE1
3	SPTSSA
3	SRBD1
3	SRRD
3	SSB
3	SUN1
3	SUPT16H
3	TAF1B
3	TAF4
3	TAP1
3	TARDBP
3	TDP1
3	TDRD7
3	TENC1
3	TIGD1
3	TIPIN
3	TM9SF4
3	TMEM14A
3	TMEM39A
3	TMEM68
3	TRIM26

3	TRIM4
3	TRMT10C
3	TSR1
3	TTLL12
3	UBTD2
3	UPF3A
3	URI1
3	UTP14C
3	VWA8
3	XPNPEP3
3	YAE1D1
3	ZBED5
3	ZBTB25
3	ZBTB37
3	ZC3H11A
3	ZCCHC9
3	ZFP36L1
3	ZFPM1
3	ZGRF1
3	ZKSCAN2
3	ZMYM1
3	ZMYM4
3	ZNF143
3	ZNF395
3	ZNF445
3	ZNF514
3	ZNF569
3	ZNF623
3	ZNF696
3	ZNF7
3	ZNF711
3	ZNF76
3	ZNF764
3	ZNF84
3	ZNHIT3
3	ZNRD1
3	ZSCAN21
3	ZSWIM3
4	ABHD5
4	ACVR2B
4	ADD1
4	AGAP1
4	AGPAT1
4	AGT
4	AKAP11

4	ANK2
4	ANTXR2
4	ANXA4
4	AP5S1
4	ARHGEF10L
4	ARHGEF19
4	ARHGEF25
4	ARHGEF40
4	ARPC1B
4	ATG14
4	ATG16L1
4	ATP2B4
4	BCAR3
4	BCAS1
4	BLOC1S3
4	BMPR1A
4	BNIP3L
4	BRAT1
4	BTBD1
4	C14orf142
4	CAPN7
4	CASP10
4	CCDC71
4	CCL20
4	CD276
4	CD46
4	CDS1
4	CERS1
4	CERS6
4	CLDN4
4	CLIC4
4	CNIH4
4	CNNM4
4	COTL1
4	CPN1
4	CTSL
4	CTSV
4	CYB561
4	DAPK1
4	DCBLD2
4	DDR1
4	DFFB
4	DUSP28
4	DUSP7
4	ECD

4	EFNA4
4	ELOVL2
4	ENOX2
4	ESAM
4	F11R
4	FAM160B2
4	FAM204A
4	FAM21A
4	FILIP1L
4	FNIP2
4	FOXC1
4	FOXJ2
4	FST
4	GDPD5
4	GIPC2
4	GNA12
4	GNB2
4	GNG4
4	GOLGA1
4	GPR157
4	GRK5
4	GUCD1
4	GXYLT2
4	HEATR5B
4	HERC1
4	HIVEP2
4	HPS6
4	IL17RB
4	IL22RA1
4	IMPA1
4	INPP1
4	INPP4A
4	INSIG2
4	IPMK
4	IQGAP2
4	IRAK4
4	IRF2
4	ITFG2
4	ITGA2
4	ITGB2
4	JKAMP
4	KCTD10
4	KCTD17
4	KCTD5
4	KLHL18

4	KLHL2
4	KLHL36
4	LAMA5
4	LIFR
4	LMBR1L
4	LPGAT1
4	LTB4R
4	LYZ
4	LZIC
4	LZTS2
4	MAPKAP1
4	MICAL2
4	MPDZ
4	MPZL2
4	MTMR1
4	MX2
4	MYO1D
4	MYO9A
4	N4BP1
4	NAALADL1
4	NCOA4
4	NDFIP1
4	NDFIP2
4	NEDD9
4	NOL3
4	NRBF2
4	NT5DC1
4	NUCB2
4	ONECUT1
4	OTULIN
4	PDXDC1
4	PELI3
4	PGAM5
4	PHKA2
4	PHLPP2
4	PI4K2B
4	PLAC8
4	PLLP
4	POGK
4	PPP1R3E
4	PPP1R9B
4	PRR15
4	PTGFRN
4	PTPRF
4	PVRL1

4	PVRL2
4	RAB18
4	RAB3D
4	RAP2C
4	RAPGEF5
4	RASSF3
4	RASSF8
4	RBPMS
4	RGP1
4	RGS19
4	RPH3AL
4	RUNDC1
4	SAYSD1
4	SERPINB9
4	SERPINE2
4	SGK2
4	SH3BP4
4	SHOC2
4	SLC12A2
4	SLC38A9
4	SLC39A6
4	SLC44A1
4	SMIM15
4	SMOC1
4	SNAP47
4	SOCS7
4	SOS2
4	SOX18
4	SPATA2
4	SPATA2L
4	SPATC1L
4	SPRYD7
4	ST20
4	STEAP3
4	SYNJ2BP
4	SYPL1
4	TAB2
4	TBC1D7
4	TBC1D8
4	TBCE
4	TECPR1
4	TGFBR3
4	TGIF2
4	THEM6
4	TJP3

4	TMEM104
4	TMEM42
4	TMEM98
4	TNFRSF10D
4	TOLLIP
4	TOM1L1
4	TPST1
4	TRAF3IP2
4	TRAF4
4	TRIM32
4	TRIM38
4	TSPAN14
4	TTYH3
4	TWF1
4	UBR7
4	VANGL1
4	VPS54
4	WHAMM
4	WIPI1
4	WNK4
4	ZBTB20
4	ZHX2
4	ZNF101
4	ZNF253
4	ZNF268
4	ZNF37A
4	ZNF480
4	ZNF772
4	ZXDC
5	AAED1
5	ABCC1
5	ABCC6
5	ABCD1
5	ABCD3
5	ACAT2
5	ACOX2
5	ACSL4
5	ACSL5
5	ACSM2B
5	ADH4
5	ADH5
5	ADPRM
5	AGMAT
5	AHSG
5	AKR1B10

5	AKR1D1
5	ALAS1
5	ALG2
5	ANKRD16
5	ANKRD36B
5	API5
5	APOM
5	ARAP1
5	ARV1
5	ATP7B
5	ATRN
5	BAG3
5	BCAP29
5	BHMT
5	BPHL
5	C15orf41
5	C19orf80
5	C2orf47
5	C4orf3
5	CA5A
5	CAAP1
5	CAPN1
5	CBR1
5	CD302
5	CEACAM1
5	CFD
5	CLCN6
5	CLDND1
5	COA1
5	COL1A1
5	CPN2
5	CRYZ
5	CTAGE5
5	CTSB
5	CTSC
5	CYSTM1
5	DDOST
5	DENND5B
5	DERA
5	DHCR24
5	DHRS1
5	DHRS2
5	DNAJB11
5	DNAJC3
5	DOLK

5	DPYSL2
5	EDEM2
5	EDEM3
5	ELMOD3
5	ELOVL6
5	EML4
5	ERGIC1
5	ERGIC2
5	EXT2
5	F5
5	FABP5
5	FAHD2A
5	FAM101B
5	FAM126B
5	FAM73A
5	GBAS
5	GGT1
5	GK
5	GLDC
5	GLOD4
5	GLT8D1
5	GLUL
5	GMPPB
5	GNS
5	GOLGA3
5	GOLGA8B
5	GOLT1B
5	GPX3
5	GSR
5	GSTA1
5	HAGH
5	HECTD4
5	HEPACAM
5	HEXB
5	HP
5	HS6ST1
5	HSD17B2
5	IGFBP1
5	IMPA2
5	IMPACT
5	ISOC1
5	KLF15
5	LACTB2
5	LANCL1
5	LARP4B

5	LGALS3
5	LIPA
5	LMAN2L
5	LNX2
5	LRP4
5	LRRC42
5	LRRC8E
5	MAOB
5	MAPK1IP1L
5	MASP2
5	MAST2
5	MCU
5	MESDC1
5	MESDC2
5	MFI2
5	MFSD3
5	MGAT4A
5	MGST1
5	MGST2
5	MGST3
5	MINPP1
5	MOGS
5	MPV17L
5	MSRB1
5	NAGPA
5	NCEH1
5	NCF2
5	NMRAL1
5	NPAS2
5	OLFML3
5	OSBPL8
5	OSTM1
5	OXER1
5	PAFAH2
5	PALM
5	PANX1
5	PDCL
5	PFKFB2
5	PIP4K2B
5	PIP4K2C
5	PIR
5	PISD
5	PITPNM2
5	PKDCC
5	PLA2G12B

5	PLA2G2A
5	POMGNT1
5	POMGNT2
5	PPP1R12C
5	PREB
5	PTGR1
5	PTPN4
5	PTS
5	PXDC1
5	RDH10
5	RFT1
5	RILPL2
5	RTN4IP1
5	RWDD1
5	SAMD4B
5	SCML1
5	SCP2
5	SDF2
5	SEC16A
5	SEC24B
5	SEMA4F
5	SEPP1
5	SERPINA10
5	SERPINC1
5	SFT2D3
5	SGSH
5	SIMC1
5	SLC22A3
5	SLC25A44
5	SLC27A4
5	SLC29A4
5	SLC30A3
5	SLC30A5
5	SLC35B1
5	SLC39A11
5	SLC39A14
5	SLCO4A1
5	SMIM20
5	SOAT1
5	STARD4
5	STARD5
5	STARD9
5	STEAP2
5	SULT1A1
5	TEF

5	TF
5	TLDC1
5	TMCC1
5	TMEM150A
5	TMEM184B
5	TMEM187
5	TMEM5
5	TMEM86B
5	TOMM40L
5	TP53INP2
5	TRAPPC11
5	TRAPPC6A
5	TSPAN13
5	TSPAN33
5	TULP4
5	TYRO3
5	UGT2B7
5	VASN
5	XYLT2
5	ZDHHC17
5	ZNF200
5	ZNF248
5	ZNF260
5	ZNF319
5	ZNF557
5	ZNF574
5	ZNF592
5	ZNF646
5	ZNF687
6	AASDH
6	ABAT
6	ACAD9
6	ACADM
6	ACN9
6	ACP2
6	ADAT2
6	ADNP2
6	AFMID
6	AHSA2
6	ALG14
6	ALG6
6	ALG8
6	ANKEF1
6	ANKRD13D
6	APOL1

6	APOOL
6	ARL8A
6	ARSB
6	ARSK
6	ASL
6	ATP5L
6	ATPAF1
6	B3GNTL1
6	B4GALT4
6	B4GALT7
6	BOLA3
6	C10orf10
6	C16orf91
6	C19orf10
6	C1GALT1
6	C1orf174
6	C1orf226
6	C4orf32
6	C6orf203
6	CARKD
6	CASD1
6	CBR4
6	CCBL1
6	CCBL2
6	CCDC115
6	CDC42BPA
6	CDK5RAP1
6	CDYL
6	CEMIP
6	CHCHD3
6	CHPF2
6	CHST13
6	CHST14
6	CHST15
6	CHST7
6	CHSY1
6	CLNS1A
6	CLPX
6	CNPY4
6	COA6
6	COLGALT1
6	COQ6
6	COX17
6	COX18
6	CREB3L2

6	CRTC3
6	CYB5A
6	CYB5B
6	DDC
6	DLST
6	DNAJA1
6	DNAJA2
6	DNAJB9
6	DNAJC11
6	DOK6
6	DPY19L4
6	EIF1AD
6	ENPP3
6	ERLIN1
6	ERP44
6	FAM120A
6	FAM184A
6	FKBP14
6	FRMD3
6	GALE
6	GALK2
6	GCAT
6	GLA
6	GLRX
6	GLRX3
6	GMCL1
6	GOLT1A
6	GYG2
6	HADH
6	HEMK1
6	HIBCH
6	HIF1AN
6	HLF
6	HMGXB3
6	HOGA1
6	HPCAL1
6	IDNK
6	IGFBP7
6	ISCA2
6	L2HGDH
6	LIPT1
6	LIX1L
6	LRIG2
6	MANEAL
6	MARC1

6	MARC2
6	METTL17
6	MIPEP
6	MMD
6	MOCS1
6	MOCS2
6	MPPE1
6	MRI1
6	MRPL19
6	MRPL32
6	MRPL34
6	MRPL45
6	MRPL46
6	MRPL49
6	MRPL50
6	MRPS22
6	MRPS36
6	MT-ND1
6	MTFR1
6	MTR
6	MTX3
6	N6AMT1
6	NAA16
6	NAAA
6	NBPF11
6	NCLN
6	NDUFA8
6	NDUFAF1
6	NDUFAF4
6	NEU3
6	NFS1
6	NHLRC2
6	NUBPL
6	NUS1
6	OAF
6	OGDH
6	OXLD1
6	P4HA1
6	PANK3
6	PAQR7
6	PCMT1
6	PDHB
6	PDXK
6	PEPD
6	PFAS

6	PGAP3
6	PGM1
6	PGM2
6	PIGB
6	PIGF
6	PIGK
6	PIGO
6	PIGV
6	PIGW
6	PIGZ
6	PM20D2
6	PPCDC
6	PPP1R12B
6	PPTC7
6	PROSC
6	PYCR2
6	PYURF
6	QTRT1
6	QTRTD1
6	RBM17
6	RFK
6	RHBDL2
6	RMND1
6	RPIA
6	RPUSD2
6	SCAMP5
6	SDHAF1
6	SEC11C
6	SEC61G
6	SEPHS2
6	SHMT1
6	SLC26A2
6	SLC35A2
6	SLC35D1
6	SLC35F5
6	SLC35F6
6	SLC5A9
6	SNX29
6	SORD
6	SOX13
6	SPRTN
6	SQRDL
6	SRP19
6	SRPRB
6	SSR3

6	ST3GAL6
6	ST6GALNAC6
6	SYAP1
6	TCOF1
6	THADA
6	THAP5
6	THUMPD2
6	TIMM9
6	TMEM129
6	TMEM2
6	TMEM230
6	TMEM70
6	TMTC4
6	TNRC18
6	TP53I13
6	TPST2
6	TRAM1
6	TRIT1
6	TRNAU1AP
6	TSFM
6	TTC28
6	TTC31
6	TTC7A
6	TYW1
6	UFL1
6	UGGT2
6	UMPS
6	USP31
6	VBP1
6	VIMP
6	WDR11
6	WDYHV1
6	YY1AP1
6	ZBED3
6	ZC3H12A
6	ZCCHC24
6	ZFYVE19
6	ZMYM5
6	ZNF232
6	ZNF26
6	ZNF407
6	ZNF500
6	ZNF561
6	ZNF706
6	ZNF740

Table S15. GO terms and KEGG pathways of STRING clusters of mRNAs regulated by miR-512-3p.

STRING Cluster	Category	Term	Description	p-value	Genes
1	**GO Term Biological Process**	GO:0051301	cell division	1.79E-11	ERCC6L, SDCCAG3, CDCA3, CCNF, TUBA1B, DSN1, CCNB1, KNTC1, NEK2, FAM83D, CDK19, BORA, RCC2, HAUS3, MASTL, KNSTRN, NDC80, MAU2, CENPF, KIF18B, MIS18A, CCNY, KIFC1, CENPJ, BIRC5, FAM64A, MAPRE1, ANAPC1, NUP37
	**GO Term Biological Process**	GO:0007062	sister chromatid cohesion	3.09E-08	ERCC6L, RCC2, DDX11, CENPA, NDC80, CENPF, DSN1, RAD51C, CENPH, BIRC5, KNTC1, MAPRE1, NUP37, NDEL1
	**KEGG Pathway**	hsa03460	Fanconi anemia pathway	7.22E-08	RMI2, RAD51C, FANCM, EME1, FANCL, UBE2T, RPA3, FANCC, FANCG

	**GO Term Biological Process**	GO:0000070	mitotic sister chromatid segregation	1.47E-07	DSN1, KIF18B, ESPL1, KIFC1, NEK2, KNSTRN, CENPA, NDC80
	**GO Term Biological Process**	GO:0007067	mitotic nuclear division	4.41E-07	ERCC6L, BORA, CDCA3, RCC2, CCNF, HAUS3, MASTL, NDC80, CENPF, MIS18A, PBK, BIRC5, KNTC1, FAM64A, NEK2, MAPRE1, FAM83D, ANAPC1, NUP37
	**GO Term Molecular Function**	GO:0061630	ubiquitin protein ligase activity	1.87E-06	RNF43, RNF14, UHRF1, FANCL, RNF38, RNF6, MARCH8, MED18, MED17, RNF138, UBE2T, MED20, TRIM37, RFFL, CBLL1
	**GO Term Cellular Component**	GO:0000777	condensed chromosome kinetochore	2.76E-06	DCTN6, ERCC6L, CENPH, FBXO28, BIRC5, KNTC1, NEK2, KNSTRN, NDC80, NDEL1, NUP37

	**GO Term Biological Process**	GO:0000086	G2/M transition of mitotic cell cycle	7.17E-06	BORA, HAUS3, MASTL, DYNLL1, TUBG1, CCNB1, CCNY, CENPJ, BIRC5, FGFR1OP, NEK2, MAPRE1, NINL
	**GO Term Cellular Component**	GO:0005813	centrosome	7.54E-06	DCTN6, UVRAG, LRRC45, ANKRD26, ERCC6L, STIL, DDX11, HAUS3, MASTL, DYNLL1, TUBG1, IFT74, CENPF, CCNB1, ESPL1, KIAA0586, CENPJ, MCM3, FGFR1OP, NEK2, CCDC77, MAPRE1, NDEL1
	**GO Term Cellular Component**	GO:0005874	microtubule	1.02E-05	RCC2, HAUS3, TUBE1, SHROOM1, DYNLL1, TUBG1, TUBA1B, KIF18B, KIFC1, CENPJ, KIF13B, BIRC5, NEK2, KIF20A, MAPRE1, NINL, DNAL4, NDEL1
	**GO Term Biological Process**	GO:0007059	chromosome segregation	3.94E-05	UVRAG, CENPF, DSN1, MIS18A, NEK2, ESCO2, KNSTRN, NDC80, NDEL1

	**GO Term Molecular Function**	GO:0004842	ubiquitin-protein transferase activity	4.54E-05	RNF43, AREL1, RNF14, UHRF1, FANCL, KLHL26, RNF38, KLHL28, RNF6, MARCH8, FBXO21, UBE2T, FBXO3, BIRC5, TRIM37, CBLL1, FBXO9
	**GO Term Cellular Component**	GO:0000776	kinetochore	8.94E-05	CENPF, CENPH, FBXO28, NEK2, DYNLL1, KNSTRN, NDC80, NDEL1, NUP37
	**GO Term Cellular Component**	GO:0030496	midbody	1.10E-04	UVRAG, CENPF, SDCCAG3, RAP2A, PRC1, RCC2, DDX11, BIRC5, NEK2, KIF20A, MYH10
	**GO Term Biological Process**	GO:0036297	interstrand cross-link repair	1.43E-04	FANCM, EME1, FANCL, UBE2T, RPA3, FANCC, FANCG
	**GO Term Cellular Component**	GO:0000922	spindle pole	1.51E-04	GPSM2, CENPF, CCNB1, DSN1, PRC1, DDX11, PLEKHG6, KNTC1, NEK2, KNSTRN

	**GO Term Cellular Component**	GO:0005819	spindle	2.32E-04	CENPF, KIFC1, PRC1, HAUS3, MAD2L1BP, BIRC5, KIF20A, MAPRE1, MYH10, NDEL1
	**GO Term Biological Process**	GO:0000132	establishment of mitotic spindle orientation	3.82E-04	GPSM2, CENPA, NDC80, MCPH1, NDEL1
	**GO Term Molecular Function**	GO:0016874	ligase activity	3.87E-04	RNF43, AREL1, RNF14, UHRF1, FANCL, RNF38, MARCH9, RNF6, UBE4B, MARCH8, RNF138, TRIM37, RFFL, CBLL1
	**GO Term Cellular Component**	GO:0000151	ubiquitin ligase complex	4.24E-04	RNF14, MED20, DCUN1D1, UBE4B, FBXO21, CBLL1, MED18, MED17, FBXO9
	**GO Term Biological Process**	GO:0016567	protein ubiquitination	4.71E-04	RNF43, CDCA3, RNF14, KLHL26, CCNF, RNF38, MARCH9, FBXO21, MED18, MED17, DCAF16, RNF138, MED20, BIRC5, CBLL1, FBXO9

	**GO Term Molecular Function**	GO:0005515	protein binding	5.36E-04	ZNF572, ERCC6L, NAB2, CCNF, FBXO28, TESK1, ELK1, EME1, FBXO3, KNTC1, NEK2, OSTF1, ANKS1A, FBXO9, ACVR1, RNF43, ABCG8, KDM2A, BORA, FKBPL, DDX11, TAP2, TRAFD1, ACYP2, ZBTB33, KNSTRN, SAP30, UHMK1, MAU2, CCNY, RRAGC, RFX5, FAM64A, KIF20A, RIN1, CBLL1, LLGL1, CDCA3, HPN, DCUN1D1, RAP1GAP, DSN1, CCNB1, ORC6, PPP4C, SEC14L1, CSTF1, SLC38A7, FAM83D, MYH10, HEXIM2, UVRAG, STIL, ANKRD27, FANCM, FANCL, FZD4, RCC2, CBX2, MAD2L1BP, ZNF32, FANCC, SMARCA1, FANCG, NDC80, ZBTB9, DIAPH1, MOB1A, KIF18B, FGFR1OP, CEP83, CRIPAK, REEP6, CNOT8, INTS9, DNAL4, MON1B, INTS12, LIN54, GPSM2, FEN1, HIP1, RNF14, PHF1, ARHGAP1, MCM10, PKD2, MED18, RAB22A, MED17, TUBA1B, BCL7B, KIF13B, PBK, SPG11, NINL, DLGAP5, NDEL1, IKBIP, TSC22D4, LRRC45, PLEKHG6, HAUS3, TUBG1, KIF7, DYNLL1, RAD23B, MED29, RAP2A, RAD51C, MRAS, ESPL1, MED20, MCM3, C19ORF25, BIRC5, MAPRE1, MCPH1, CHMP5, WDR27, SDCCAG3, UHRF1, PKN3, RNF6, FOXK1, CENPA, RAB21, DCAF16,

					ARVCF, ARL13B, RNF138, FCHO2, ZNF747, ZNF227, IGF2BP3, RBBP7, RFFL, PDLIM5, RRM2, DAB2IP, ARID3A, TBX2, CENPF, MIS18A, CENPH, PRC1, CENPJ, SHKBP1, RPA3, TRIM37, LGR4, SNTB1, NUP37

	**GO Term Biological Process**	GO:0060271	cilium morphogenesis	5.58E-04	SPAG16, IFT74, ARL13B, TMEM138, KIAA0586, TTC21B, WDR19, CEP83, DYNLL1, PTPDC1
	**GO Term Molecular Function**	GO:0005096	GTPase activator activity	6.51E-04	ANKRD27, GRTP1, ELMOD2, PLEKHG6, DAB2IP, ARHGAP1, ARHGAP19, ARHGAP18, RAP1GAP, DEPDC1B, RIN1, AGFG2, LLGL1
	**GO Term Molecular Function**	GO:0003924	GTPase activity	7.81E-04	RAB21, RAP2A, TUBA1B, RAB31, MRAS, RRAGC, RABL2A, RABL2B, TUBE1, TUBG1, RAP1GAP, RAB22A

	**GO Term Cellular Component**	GO:0005654	nucleoplasm	0.00123047	INTS12, LIN54, ERCC6L, FEN1, PHF1, MCM10, ELK1, PTPDC1, MED17, EME1, FBXO3, ANKS1A, LMCD1, TRIM66, LIN52, LRRC45, RMI2, KDM2A, DDX11, HAUS3, ESCO2, ZBTB33, MASTL, RAD23B, MED29, SAP30, VWA9, MAU2, RAD51C, MED20, MCM3, BIRC5, KIF20A, ANAPC1, MCPH1, WDR27, PRIM1, RNF38, RNF6, CENPA, CCNB1, PPP4C, ORC6, CSTF1, RHPN2, RBBP7, RFFL, GINS1, HEXIM2, RRM2, FANCM, FANCL, GINS3, CBX2, WDR19, FANCC, ARID3A, FANCG, CENPF, MIS18A, CENPH, PRC1, UBE2T, RPA3, TADA1, GTF2IRD1, INTS9, NUP37

	**GO Term Biological Process**	GO:0032006	regulation of TOR signaling	0.00128459	RRAGC, RFFL, FAM83D, FBXO9
	**GO Term Cellular Component**	GO:0005871	kinesin complex	0.00136946	KIF18B, KIFC1, KIF13B, KIF20A, KIF7, NDEL1
	**GO Term Cellular Component**	GO:0043240	Fanconi anaemia nuclear complex	0.00165187	FANCM, FANCL, FANCC, FANCG
	**GO Term Biological Process**	GO:0006260	DNA replication	0.00177909	FEN1, RMI2, RRM2, CHTF8, ORC6, GINS3, RPA3, MCM3, MCM10, RBBP7
	**GO Term Biological Process**	GO:0006281	DNA repair	0.0027396	INO80C, UVRAG, FEN1, RAD51C, EME1, FANCL, UHRF1, UBE2T, RPA3, DDX11, FANCC, FANCG
	**GO Term Molecular Function**	GO:0003697	single-stranded DNA binding	0.00314569	RAD51C, RNF138, PRIM1, RPA3, DDX11, MCM10, RAD23B
	**GO Term Cellular Component**	GO:0000775	chromosome, centromeric region	0.00342417	UVRAG, CENPF, MIS18A, BIRC5, CENPA, NDC80
	**GO Term Biological Process**	GO:0007224	smoothened signaling pathway	0.0036904	STIL, ARL13B, KIAA0586, TTC21B, CENPJ, PTPDC1

	**GO Term Biological Process**	GO:0042787	protein ubiquitination involved in ubiquitin-dependent protein catabolic process	0.00440525	RNF43, CCNB1, AREL1, RNF14, UHRF1, KLHL28, UBE4B, RFFL, ANAPC1
	**GO Term Biological Process**	GO:0007264	small GTPase mediated signal transduction	0.00481253	RAB21, RAP2A, RAB31, MRAS, RRAGC, ARL13B, RABL2A, ARHGAP1, RABL2B, ARHGAP18, RAB22A
	**GO Term Molecular Function**	GO:0008017	microtubule binding	0.00531502	KIF18B, KIFC1, PRC1, RCC2, KIF13B, BIRC5, KIF20A, FAM83D, KIF7, NDEL1
	**GO Term Molecular Function**	GO:0019901	protein kinase binding	0.00647089	BORA, RCC2, DAB2IP, CCNB1, CCNY, RNF138, PRC1, CENPJ, KIF13B, FGFR1OP, KIF20A, FAM83D, RFFL, LLGL1
	**GO Term Molecular Function**	GO:0003777	microtubule motor activity	0.00653846	KIF18B, KIFC1, KIF13B, KIF20A, KIF7, DNAL4
	**GO Term Cellular Component**	GO:0072686	mitotic spindle	0.00664357	DIAPH1, ESPL1, PKD2, DYNLL1, KNSTRN

	**GO Term Cellular Component**	GO:0005929	cilium	0.00700748	IFT74, ARL13B, TMEM138, TTC21B, WDR19, KIF7, PKD2, DNAL4
	**GO Term Cellular Component**	GO:0097542	ciliary tip	0.00725484	IFT74, TTC21B, WDR19, KIF7, DYNLL1
	**KEGG Pathway**	hsa03030	DNA replication	0.00807608	FEN1, PRIM1, RPA3, MCM3
	**GO Term Biological Process**	GO:0007018	microtubule-based movement	0.00854452	KIF18B, KIFC1, KIF13B, KIF20A, KIF7, DNAL4
	**GO Term Biological Process**	GO:0000082	G1/S transition of mitotic cell cycle	0.00864605	ACVR1, RRM2, ORC6, PRIM1, RPA3, MCM3, MCM10
	**GO Term Biological Process**	GO:0035721	intraciliary retrograde transport	0.00920343	TTC21B, WDR19, DYNLL1
	**GO Term Cellular Component**	GO:0030424	axon	0.00951969	DAB2IP, SLC38A7, KIF13B, RNF6, SPG11, MYH10, RAP1GAP, LLGL1, UHMK1
	**GO Term Biological Process**	GO:0051310	metaphase plate congression	0.01168684	CENPF, FAM83D, NDC80
	**GO Term Cellular Component**	GO:0005814	centriole	0.01215083	STIL, KIAA0586, CENPJ, CCNF, BIRC5, CEP83, TUBG1

	**GO Term Cellular Component**	GO:0005634	nucleus	0.01385545	ZNF572, NAB2, CCNF, ELK1, PTPDC1, EME1, KNTC1, NEK2, ZBTB39, AREL1, KDM2A, DDX11, UBE4B, ZBTB33, ESCO2, KNSTRN, UHMK1, MAU2, CCNY, RRAGC, RFX5, FAM64A, DCUN1D1, ARHGAP19, DSN1, CCNB1, ORC6, CHTF8, PPP4C, CSTF1, MYH10, HEXIM2, CDK19, RCC2, CBX2, MAD2L1BP, ZNF32, FANCC, SMARCA1, NDC80, ZBTB9, KIF18B, UBE2T, FGFR1OP, CRIPAK, GTF2IRD1, REEP6, CNOT8, SPAG16, FEN1, HIP1, RNF14, PHF1, MCM10, MED17, PBK, LMCD1, DLGAP5, ZNF587B, TSC22D4, RMI2, ZNF362, MASTL, DYNLL1, RAD23B, RAD51C, ESPL1, KIFC1, MCM3, BIRC5, CHMP5, MYRF, UHRF1, PKN3, RNF38, RNF6, FOXK1, CENPA, IFT74, ARVCF, RNF138, ATOH8, ZNF227, IGF2BP3, RBBP7, GINS1, RRM2, ATAD5, GINS3, ARID3A, TBX2, CENPF, MIS18A, CENPH, PRC1, RPA3, ZNF354A, NUP37

	**GO Term Cellular Component**	GO:0016235	aggresome	0.01391293	ORC6, CCZ1B, TRIM37, TRIM66
	**GO Term Cellular Component**	GO:0005815	microtubule organizing center	0.0151727	DIAPH1, PPP4C, KIFC1, PHF1, FGFR1OP, NINL, DLGAP5, MCPH1
	**GO Term Molecular Function**	GO:0003688	DNA replication origin binding	0.01632257	ORC6, DDX11, MCM10
	**GO Term Biological Process**	GO:0007052	mitotic spindle organization	0.01686299	GPSM2, STIL, CCNB1, NDC80
	**GO Term Biological Process**	GO:0051382	kinetochore assembly	0.01741763	CENPF, CENPH, CENPA
	**GO Term Cellular Component**	GO:0031514	motile cilium	0.01761233	SPAG16, IFT74, ARL13B, WDR19, PKD2
	**GO Term Biological Process**	GO:0006270	DNA replication initiation	0.01848136	ORC6, PRIM1, MCM3, MCM10
	**GO Term Biological Process**	GO:0051726	regulation of cell cycle	0.01926393	LIN54, CENPF, CDK19, CCNB1, CCNF, MASTL, LIN52
	**GO Term Biological Process**	GO:0042384	cilium assembly	0.02003498	SPAG16, IFT74, ARL13B, TMEM138, KIAA0586, WDR19, CEP83

	**GO Term Biological Process**	GO:0007017	microtubule-based process	0.02018297	TUBA1B, TUBE1, DYNLL1, DNAL4
	**GO Term Molecular Function**	GO:0005525	GTP binding	0.02119777	RAB21, RAP2A, TUBA1B, RAB31, MRAS, RRAGC, ARL13B, RABL2A, RABL2B, TUBE1, TUBG1, RAB22A
	**GO Term Biological Process**	GO:0000722	telomere maintenance via recombination	0.021968	FEN1, RAD51C, PRIM1, RPA3
	**GO Term Cellular Component**	GO:0016592	mediator complex	0.02389909	MED20, MED29, MED18, MED17
	**GO Term Biological Process**	GO:0006271	DNA strand elongation involved in DNA replication	0.03165054	GINS1, GINS3, PRIM1
	**GO Term Cellular Component**	GO:0035098	ESC/E(Z) complex	0.03170424	PHF1, TRIM37, RBBP7
	**GO Term Biological Process**	GO:0034080	CENP-A containing nucleosome assembly	0.03214305	MIS18A, CENPH, RBBP7, CENPA
	**GO Term Cellular Component**	GO:0031513	nonmotile primary cilium	0.04006704	WDR19, TUBG1, PKD2
	**GO Term Cellular Component**	GO:0035371	microtubule plus-end	0.04006704	KIF18B, MAPRE1, KNSTRN

	**KEGG Pathway**	hsa03440	Homologous recombination	0.04129865	RAD51C, EME1, RPA3
	**GO Term Cellular Component**	GO:0072372	primary cilium	0.04315604	IFT74, ARL13B, TTC21B, WDR19, DYNLL1
	**GO Term Biological Process**	GO:0000910	cytokinesis	0.04437272	ESPL1, PRC1, BIRC5, KIF20A
	**GO Term Biological Process**	GO:0071539	protein localization to centrosome	0.04445261	STIL, CEP83, MCPH1
	**GO Term Biological Process**	GO:0007020	microtubule nucleation	0.04445261	CENPJ, TUBG1, NDEL1
	**GO Term Cellular Component**	GO:0032154	cleavage furrow	0.04448127	RAB21, PLEKHG6, MASTL, MYH10
	**KEGG Pathway**	hsa04110	Cell cycle	0.04478949	CCNB1, ORC6, ESPL1, MCM3, ANAPC1
	**GO Term Biological Process**	GO:0007368	determination of left/right symmetry	0.04705919	ACVR1, IFT74, STIL, PKD2
	**GO Term Biological Process**	GO:0001947	heart looping	0.0498241	STIL, ARL13B, PKD2, TBX2
	**GO Term Cellular Component**	GO:0005881	cytoplasmic microtubule	0.04994405	TUBA1B, BIRC5, TUBG1, MAPRE1

**2**	**GO Term Cellular Component**	GO:0005654	nucleoplasm	6.69E-15	RB1, KDM5B, CDKN1B, CCNH, FMR1, FHL2, UBE2D1, IDE, BRCA2, SYNE2, CCAR2, RPTOR, STK11, MYC, CHEK2, SUFU, SUMO2, RCC1, SOX9, TOPBP1, SKP2, NCOA2, SREBF1, MED1, PBRM1, TBP, CHUK, METTL3, H2AFX, PRKCD, DNMT3A, VPS37A, PAX6, DUSP6, SENP1, HCFC1, RBL2, PSMA3, PLCB3, RBL1, PSMA4, CREB1, TFDP1, MORF4L2, RARA, WAC, MAPKAPK5, DNA2, UTRN, KDM7A, BIRC3, PSMD11, PSMD14, CUL3, SRSF1, CUL1, GPS2, COPS7A, TXN, RNF2, TGOLN2, E2F2, E2F3, E2F4, DMAP1, E2F5, BPTF, MCL1, MAPK3, MAP2K6, ESRRA, SMAD2, KDM4A, HSPA8, TCF7L1, CDKN2A, SETD1B, INTS2, NR2F1, NR1H4, NR1H3, HIPK2, CDK8, MLXIPL, GOLPH3, CDK6, CDKN2AIPNL, SP4, APEX1, ID1, CDK12, FERMT2, TJP2, TAF1

	**GO Term Molecular Function**	GO:0005515	protein binding	6.41E-14	RB1, EPO, IRS1, CCNH, FMR1, GFI1, SERPINE1, UBE2D1, LCLAT1, CCAR2, EEF1B2, STK11, MYC, CHEK2, RCC1, SOX9, PIM2, SKP2, MAP3K7, PDK1, MED1, PRKCI, PRKCD, COG2, DKK1, HCFC1, RNF126, LOX, EWSR1, PRKAR1A, MORF4L2, MAPKAPK5, UBE2V1, DNA2, PFN1, UTRN, MACF1, PSMD11, TXNDC9, PSMD14, CUL3, CUL1, HTRA2, VPS26A, COPS7A, PSEN1, TXN, NDRG1, GLIS2, TGOLN2, SDCBP, SNX1, FADD, DMAP1, PROM1, CDR2, MCL1, BPTF, MAP2K6, ESRRA, SMAD2, HSPA8, PRNP, HOMER1, SETD1B, INSR, INTS2, NR2F1, EIF2AK3, PARVA, UBE2A, IGF2R, CDK8, DLG1, CDK6, AGO4, SP4, ID1, ALB, UBA3, SDC1, CDK12, GNB5, ALDH18A1, MYD88, FERMT2, FGFR1, KDM5B, CSRNP2, CDKN1B, MOCS3, TRADD, PDE3B, FHL2, IDE, PIK3CB, BRCA2, GPHN, HK2, SYNE2, CASP9, RPTOR, SUFU, SUMO2, TOPBP1, LBR, NKX3-1, BRD3, NCOA2, SREBF1, PBRM1, TBP, CHUK, KSR1, H2AFX, DNMT3A, RRAS2, VPS37A, PAX6, SENP1, RBL2, BACE1, WRAP73, PSMA3, RBL1, PSMA4, CREB1, TFDP1, CTTN, TRAF6, PEX5, RARA, WAC, PIK3C3, BIRC3, ARF6, FH, STX16, SRSF1, GPS2, MAT2B, HMGCR, AFP,

					RNF2, CLN3, MAT2A, IGF2BP1, E2F2, E2F3, E2F4, E2F5, EIF4E, MAPK3, UBL4A, KDM4A, CDKN2A, SORT1, USP22, HIP1R, NR1H4, RANBP9, NR1H3, UBE2G2, SORL1, HIPK2, GOLPH3, APEX1, VPS41, AGGF1, FAS, CD9, BCAR1, TJP2, TAF1

	**GO Term Cellular Component**	GO:0005634	nucleus	2.79E-10	RB1, IRS1, CCNH, FMR1, GFI1, CCAR2, EEF1B2, STK11, MYC, RCC1, SOX9, SKP2, MAP3K7, MED1, PRKCI, METTL3, PRKCD, ASRGL1, HCFC1, RNF126, LOX, EWSR1, MORF4L2, SUCLG2, MAPKAPK5, UBE2V1, DNA2, PFN1, PSMD11, TXNDC9, PSMD14, CUL3, HTRA2, PSEN1, TXN, NDRG1, GLIS2, SDCBP, DMAP1, MCL1, BPTF, ESRRA, SMAD2, HSPA8, PRNP, SETD1B, NR2F1, GGH, PARVA, CDK8, MLXIPL, DLG1, CDK6, AGO4, SP4, ID1, ALB, UBA3, CDK12, GNB5, MYD88, FERMT2, FGFR1, KDM5B, CSRNP2, CDKN1B, TRADD, FHL2, IDE, PIK3CB, BRCA2, SYNE2, CASP9, SUFU, SUMO2, TOPBP1, NKX3-1, BRD3, NCOA2, SREBF1, TBP, H2AFX, DNMT3A, SIRT5, PAX6, SENP1, RBL2, PSMA3, RBL1, PSMA4, CREB1, TFDP1, TRAF6, RARA, WAC, KDM7A, BIRC3, SRSF1, MAT2B, RNF2, CLN3, IGF2BP1, E2F3, E2F4, E2F5, MAPK3, UBL4A, KDM4A, TCF7L1, CDKN2A, NR1H4, RANBP9, NR1H3, HIPK2, UFM1, PITHD1, APEX1, FAS, TAF1

	**GO Term Biological Process**	GO:0000122	negative regulation of transcription from RNA polymerase II promoter	3.72E-10	RB1, EPO, CUL3, GFI1, FHL2, UBE2D1, GPS2, PSEN1, TXN, RNF2, GLIS2, MYC, SUFU, DMAP1, BPTF, ESRRA, SMAD2, SREBF1, NCOA2, MED1, DNMT3A, NR2F1, NR1H4, NR1H3, PAX6, DKK1, HIPK2, HCFC1, MLXIPL, DLG1, RBL1, TRAF6, ID1, RARA, FGFR1
	**GO Term Biological Process**	GO:0045893	positive regulation of transcription, DNA-templated	7.67E-10	RB1, EPO, FHL2, PSEN1, BRCA2, GLIS2, CHEK2, MYC, E2F3, SOX9, PIM2, BPTF, NKX3-1, MAPK3, SMAD2, MED1, TBP, CDKN2A, USP22, INSR, NR1H3, PAX6, HIPK2, MLXIPL, CREB1, RARA, WAC, UBE2V1, KDM7A

	**GO Term Cellular Component**	GO:0005829	cytosol	9.29E-10	CDKN1B, MOCS3, IRS1, TRADD, PDE3B, UBE2D1, PIK3CB, IDE, HK2, EEF1B2, RPTOR, CASP9, STK11, MYC, SUFU, SKP2, MAP3K7, SREBF1, PRKCI, KSR1, CHUK, PRKCD, COG2, SIRT5, ASRGL1, DUSP6, PSMA3, PLCB3, RNF126, PSMA4, PRKAR1A, TRAF6, PEX5, CAT, PIK3C3, MAPKAPK5, UBE2V1, PFN1, BIRC3, FH, PSMD11, PSMD14, STX16, CUL3, CUL1, HTRA2, VPS26A, MAT2B, TXN, NDRG1, TGOLN2, SDCBP, SNX1, MAT2A, IGF2BP1, FADD, PCK1, EIF4E, MCL1, MAPK3, MAP2K6, UBL4A, SMAD2, HSPA8, CHKA, SORT1, CDKN2A, IDH1, GGH, MTHFR, RANBP9, PARVA, UBE2A, UBE2G2, DDHD1, MLXIPL, DLG1, GOLPH3, CDK6, PSAT1, AGO4, VPS41, UBA3, FAS, GNB5, MYD88, BCAR1, FERMT2, TJP2, FGFR1

	**GO Term Molecular Function**	GO:0008134	transcription factor binding	7.58E-09	RB1, SMAD2, NCOA2, MED1, TBP, CDKN2A, FHL2, PAX6, MLXIPL, RBL1, TFDP1, MYC, SUFU, ID1, RARA, E2F2, E2F4, E2F5, BPTF, TAF1, NKX3-1
	**GO Term Biological Process**	GO:0045944	positive regulation of transcription from RNA polymerase II promoter	1.68E-08	RB1, CSRNP2, CCNH, SERPINE1, GLIS2, MYC, SUMO2, FADD, SOX9, E2F4, E2F5, NKX3-1, MAPK3, ESRRA, SMAD2, SREBF1, NCOA2, MED1, CHUK, CDKN2A, NR2F1, NR1H4, NR1H3, PAX6, HIPK2, SENP1, HCFC1, CDK8, MLXIPL, CREB1, RBL1, TFDP1, TRAF6, MORF4L2, RARA, PFN1, TAF1

	**GO Term Biological Process**	GO:0043066	negative regulation of apoptotic process	1.07E-07	PRNP, MED1, PRKCI, CDKN1B, FHL2, NR1H4, PSEN1, CLN3, GOLPH3, DAD1, AGO4, MYC, TRAF6, ID1, CAT, ALB, RARA, FAS, PIM2, SOX9, MYD88, MCL1, BIRC3
	**GO Term Cellular Component**	GO:0043234	protein complex	1.42E-07	SREBF1, PRKCI, CDKN1B, KSR1, CDKN2A, UBE2D1, PSEN1, BRCA2, CDK8, SNX1, PLCB3, PRKAR1A, MYC, TRAF6, PEX5, ALB, SDC1, SOX9, UBE2V1, UTRN, MYD88, BIRC3, MAPK3
	**GO Term Biological Process**	GO:0050852	T cell receptor signaling pathway	1.55E-07	PSMD11, PSMD14, CHUK, CUL1, UBE2D1, PIK3CB, PSEN1, STK11, PSMA3, PSMA4, TRAF6, UBE2V1, MAP3K7, BCAR1

	**KEGG Pathway**	hsa04110	Cell cycle	3.29E-07	RB1, SMAD2, CDKN1B, CDKN2A, CCNH, CUL1, RBL2, RBL1, TFDP1, CDK6, CHEK2, MYC, E2F2, E2F3, E2F4, SKP2, E2F5

	**GO Term Cellular Component**	GO:0005737	cytoplasm	1.06E-06	IRS1, FMR1, UBE2D1, CCAR2, EEF1B2, STK11, RCC1, PIM2, SKP2, MAP3K7, PRKCI, PRKCD, ASRGL1, HCFC1, RNF123, RNF126, EWSR1, PRKAR1A, MAPKAPK5, UBE2V1, PFN1, UTRN, MACF1, TXNDC9, COPS7A, TXN, NDRG1, UBE2J1, GLIS2, SDCBP, SNX1, FADD, DMAP1, PCK1, CDR2, MCL1, BPTF, MAP2K6, SMAD2, PRNP, SETD1B, IDH1, EIF2AK3, PARVA, UBE2A, IGF2R, MLXIPL, DLG1, CDK6, AGO4, SP4, SDC1, ALDH18A1, NES, MYD88, FERMT2, KDM5B, CDKN1B, TRADD, IDE, BRCA2, GPHN, SYNE2, RPTOR, SUFU, TOPBP1, NCOA2, SREBF1, TBP, CHUK, DNMT3A, VPS37A, PAX6, DUSP6, SENP1, RBL2, PSMA3, PSMA4, CTTN, TRAF6, PEX5, RARA, BIRC3, FH, STX16, SRSF1, AFP, CLN3, IGF2BP1, E2F3, E2F5, EIF4E, UBL4A, KDM4A, CDKN2A, HIP1R, RANBP9, DDHD1, HIPK2, ETAA1, UFM1, PSAT1, APEX1, AGGF1, FAS, BCAR1, TJP2

	**KEGG Pathway**	hsa05200	Pathways in cancer	2.25E-06	RB1, FH, CDKN1B, PIK3CB, BRCA2, CASP9, MYC, SUFU, E2F2, E2F3, FADD, SKP2, NKX3-1, MAPK3, SMAD2, TCF7L1, CHUK, CDKN2A, PLCB3, CDK6, TRAF6, RARA, FAS, GNB5, BIRC3, FGFR1
	**GO Term Biological Process**	GO:0002223	stimulatory C-type lectin receptor signaling pathway	4.25E-06	PSMA3, PSMA4, PSMD11, PSMD14, CHUK, TRAF6, PRKCD, CUL1, UBE2D1, UBE2V1, MAP3K7
	**GO Term Biological Process**	GO:0038095	Fc-epsilon receptor signaling pathway	5.30E-06	PSMA3, PSMA4, PSMD11, PSMD14, CHUK, TRAF6, CUL1, UBE2D1, UBE2V1, PIK3CB, MAP3K7, MAPK3
	**GO Term Biological Process**	GO:0051726	regulation of cell cycle	5.30E-06	RB1, RBL2, CDK8, HSPA8, MED1, RBL1, UBA3, E2F2, CDK12, E2F5, SKP2, HIPK2

	**KEGG Pathway**	hsa05222	Small cell lung cancer	6.03E-06	RB1, CASP9, CDKN1B, CDK6, CHUK, MYC, TRAF6, E2F2, E2F3, PIK3CB, SKP2, BIRC3
	**GO Term Biological Process**	GO:0032147	activation of protein kinase activity	6.24E-06	RPTOR, STK11, DLG1, EPO, TRAF6, INSR, PRKCD, NKX3-1
	**GO Term Biological Process**	GO:0000082	G1/S transition of mitotic cell cycle	6.99E-06	RB1, CDKN1B, CDK6, CDKN2A, CCNH, CUL3, CUL1, RCC1, PIM2, SKP2, EIF4E
	**GO Term Molecular Function**	GO:0019899	enzyme binding	1.00E-05	HSPA8, TBP, SORT1, USP22, H2AFX, PRKCD, EIF2AK3, RANBP9, MAT2B, CCAR2, IGF2R, BACE1, GOLPH3, CREB1, PEX5, CAT, RARA, EIF4E
	**KEGG Pathway**	hsa05212	Pancreatic cancer	1.08E-05	RB1, SMAD2, CASP9, CDK6, CHUK, CDKN2A, E2F2, E2F3, PIK3CB, BRCA2, MAPK3

	**GO Term Biological Process**	GO:0000209	protein polyubiquitination	1.27E-05	PSMD11, PSMD14, CDKN2A, CUL3, CUL1, UBE2D1, UBE2A, RNF126, PSMA3, PSMA4, TRAF6, UBE2V1, SKP2, TAF1
	**GO Term Biological Process**	GO:0016032	viral process	1.78E-05	RB1, HSPA8, KDM4A, TBP, NUP210, GFI1, H2AFX, CUL1, DLG1, PSMA3, CREB1, RBL1, PSMA4, RCC1, EIF4E, TAF1, MAPK3
	**KEGG Pathway**	hsa05215	Prostate cancer	1.79E-05	RB1, CASP9, TCF7L1, CDKN1B, CREB1, CHUK, E2F2, E2F3, PIK3CB, FGFR1, NKX3-1, MAPK3
	**GO Term Molecular Function**	GO:0042802	identical protein binding	1.81E-05	RB1, TRADD, FMR1, FHL2, SDCBP, SNX1, MAT2A, CHEK2, TOPBP1, FADD, SKP2, PRNP, DNMT3A, EIF2AK3, UBE2G2, IGF2R, HCFC1, CREB1, EWSR1, TRAF6, VPS41, ALB, UBA3, FAS, ALDH18A1, MYD88, FGFR1

	**GO Term Biological Process**	GO:0006367	transcription initiation from RNA polymerase II promoter	1.91E-05	ESRRA, CDK8, MED1, TBP, CCNH, NR2F1, NR1H4, RARA, E2F2, NR1H3, E2F3, TAF1
	**KEGG Pathway**	hsa05161	Hepatitis B	4.04E-05	RB1, CDKN1B, CHUK, PIK3CB, CASP9, CREB1, CDK6, MYC, FAS, E2F2, E2F3, FADD, MYD88, MAPK3
	**GO Term Biological Process**	GO:0006974	cellular response to DNA damage stimulus	4.11E-05	FMR1, H2AFX, PSEN1, CCAR2, CASP9, STK11, PITHD1, CHEK2, MYC, TRAF6, WAC, TOPBP1, TAF1, MAPK3
	**GO Term Biological Process**	GO:0006919	activation of cysteine-type endopeptidase activity involved in apoptotic process	4.75E-05	CDKN1B, CDKN2A, TRADD, HIP1R, EIF2AK3, FAS, FADD, SENP1, NKX3-1
	**GO Term Cellular Component**	GO:0005667	transcription factor complex	5.38E-05	SMAD2, RBL2, MLXIPL, TCF7L1, TFDP1, RBL1, APEX1, E2F2, E2F3, E2F4, E2F5, TAF1

	**GO Term Molecular Function**	GO:0004672	protein kinase activity	7.14E-05	PRKCI, KSR1, CHUK, PRKCD, EIF2AK3, HIPK2, CDK8, STK11, CHEK2, MAPKAPK5, PIK3C3, PIM2, SOX9, CDK12, MAP3K7, MAP2K6, PDK1
	**GO Term Biological Process**	GO:0007050	cell cycle arrest	8.45E-05	RPTOR, RB1, PRNP, STK11, CDKN1B, CDK6, CDKN2A, MYC, CUL3, CUL1, MAP2K6
	**GO Term Biological Process**	GO:0008284	positive regulation of cell proliferation	9.65E-05	CDKN1B, EPO, IRS1, CUL3, INSR, UBE2A, HIPK2, MLXIPL, SDCBP, DLG1, MYC, RARA, E2F3, SOX9, FGFR1, TAF1, NKX3-1
	**GO Term Molecular Function**	GO:0003713	transcription coactivator activity	1.06E-04	RB1, NCOA2, MED1, USP22, FHL2, NR2F1, NR1H4, NR1H3, HCFC1, TFDP1, APEX1, SP4, RARA, TAF1
	**GO Term Biological Process**	GO:0038061	NIK/NF-kappaB signaling	1.18E-04	PSMA3, PSMA4, PSMD11, PSMD14, CHUK, CUL1, UBA3, BIRC3

	**GO Term Cellular Component**	GO:0005925	focal adhesion	1.25E-04	HSPA8, STX16, FHL2, RRAS2, PARVA, SYNE2, IGF2R, SENP1, SDCBP, CTTN, CAT, CD9, SDC1, PFN1, FERMT2, BCAR1, MAPK3, ARF6
	**GO Term Biological Process**	GO:0000165	MAPK cascade	1.50E-04	PSMD11, PSMD14, KSR1, IRS1, CUL3, DUSP6, PSMA3, PSMA4, MYC, MAPKAPK5, MAP2K6, FGFR1, MAPK3
	**GO Term Cellular Component**	GO:0000790	nuclear chromatin	1.50E-04	SMAD2, CREB1, TBP, H2AFX, RARA, RCC1, NR1H3, PAX6, E2F4, UBE2A, CCAR2, TAF1
	**GO Term Biological Process**	GO:0032355	response to estradiol	1.59E-04	ESRRA, CASP9, CDKN1B, MYC, DNMT3A, CAT, RARA, TFPI
	**KEGG Pathway**	hsa04068	FoxO signaling pathway	1.62E-04	SMAD2, CDKN1B, HOMER1, CHUK, IRS1, INSR, PIK3CB, RBL2, STK11, CAT, SKP2, PCK1, MAPK3

	**GO Term Molecular Function**	GO:0031625	ubiquitin protein ligase binding	1.67E-04	RB1, SMAD2, HSPA8, KDM4A, CUL3, CUL1, PAX6, UBE2A, UBE2G2, UBE2J1, PRKAR1A, CHEK2, TRAF6, SUMO2, UBE2V1
	**GO Term Biological Process**	GO:0006468	protein phosphorylation	1.73E-04	SMAD2, PRKCI, CHUK, CCNH, PRKCD, EIF2AK3, HIPK2, CDK8, STK11, CREB1, CDK6, CHEK2, RARA, PIK3C3, PIM2, PDK1, FGFR1, TAF1, MAPK3
	**GO Term Biological Process**	GO:0007568	aging	1.85E-04	CASP9, SREBF1, CREB1, EPO, APEX1, DNMT3A, CAT, HTRA2, HMGCR, PCK1
	**KEGG Pathway**	hsa05220	Chronic myeloid leukemia	1.95E-04	RB1, CDKN1B, CDK6, CHUK, CDKN2A, MYC, E2F2, E2F3, PIK3CB, MAPK3
	**GO Term Biological Process**	GO:0045471	response to ethanol	2.12E-04	EEF1B2, PSMD14, MYC, CAT, RARA, GGH, HMGCR, DMAP1

	**GO Term Biological Process**	GO:0042493	response to drug	2.16E-04	SREBF1, CDKN1B, CHUK, DNMT3A, GGH, MTHFR, DUSP6, CREB1, LOX, DAD1, MYC, APEX1, CAT, MAP2K6
	**KEGG Pathway**	hsa05169	Epstein-Barr virus infection	2.26E-04	RB1, CDKN1B, PSMD11, PSMD14, CHUK, MYC, TRAF6, TRADD, PIK3CB, SKP2, MAP3K7, MAP2K6
	**KEGG Pathway**	hsa04151	PI3K-Akt signaling pathway	2.27E-04	CDKN1B, CHUK, EPO, IRS1, INSR, PIK3CB, CASP9, RBL2, RPTOR, STK11, CREB1, CDK6, MYC, GNB5, PCK1, EIF4E, MCL1, FGFR1, MAPK3
	**GO Term Biological Process**	GO:0000187	activation of MAPK activity	2.32E-04	TRAF6, INSR, MAPKAPK5, PIK3CB, MAP3K7, DUSP6, MAP2K6, MAPK3

	**GO Term Biological Process**	GO:0045892	negative regulation of transcription, DNA-templated	2.46E-04	RB1, SMAD2, KDM5B, HSPA8, KDM4A, CDKN1B, CDKN2A, GFI1, FHL2, CCAR2, GLIS2, MLXIPL, TRAF6, ID1, UBA3, RARA, SOX9, DMAP1, NKX3-1
	**GO Term Biological Process**	GO:0006511	ubiquitin-dependent protein catabolic process	2.88E-04	PSMA3, PSMA4, PSMD11, PSMD14, CUL3, USP22, CUL1, UBE2D1, UBE2A, UBE2G2, SKP2
	**GO Term Biological Process**	GO:2000059	negative regulation of protein ubiquitination involved in ubiquitin-dependent protein catabolic process	3.01E-04	CDKN2A, SUFU, PSEN1, TAF1

	**GO Term Biological Process**	GO:0006351	transcription, DNA-templated	3.85E-04	RB1, KDM5B, CCNH, GFI1, FHL2, TXN, CCAR2, RNF2, CHEK2, E2F3, E2F4, DMAP1, E2F5, MAP3K7, BPTF, NKX3-1, MAPK3, MAP2K6, BRD3, ESRRA, SMAD2, NCOA2, KDM4A, HSPA8, PBRM1, TCF7L1, CDKN2A, SETD1B, USP22, NR2F1, NR1H4, NR1H3, PAX6, HIPK2, RBL2, MLXIPL, RBL1, TFDP1, EWSR1, APEX1, SP4, MORF4L2, ID1, RARA, WAC, KDM7A, FGFR1
	**KEGG Pathway**	hsa05142	Chagas disease (American trypanosomiasis)	4.58E-04	SMAD2, PLCB3, CHUK, TRAF6, SERPINE1, FAS, FADD, PIK3CB, MYD88, MAPK3

	**GO Term Molecular Function**	GO:0001077	transcriptional activator activity, RNA polymerase II core promoter proximal region sequence-specific binding	5.39E-04	ESRRA, SMAD2, SREBF1, CREB1, MYC, NR1H4, NR1H3, PAX6, E2F4, SOX9, GLIS2
	**KEGG Pathway**	hsa05218	Melanoma	5.61E-04	RB1, CDK6, CDKN2A, E2F2, E2F3, PIK3CB, FGFR1, MAPK3
	**GO Term Biological Process**	GO:0010942	positive regulation of cell death	5.79E-04	CDKN1B, DNMT3A, HTRA2, DUSP6, NKX3-1
	**GO Term Biological Process**	GO:2001022	positive regulation of response to DNA damage stimulus	6.29E-04	MYC, FMR1, PRKCD, NKX3-1
	**GO Term Biological Process**	GO:0034162	toll-like receptor 9 signaling pathway	6.29E-04	TRAF6, NR1H4, PIK3C3, MYD88
	**KEGG Pathway**	hsa05223	Non-small cell lung cancer	6.38E-04	RB1, CASP9, CDK6, CDKN2A, E2F2, E2F3, PIK3CB, MAPK3
	**GO Term Molecular Function**	GO:0061630	ubiquitin protein ligase activity	6.53E-04	MED1, RNF126, CUL3, TRAF6, CUL1, UBE2A, UBE2V1, UBE2G2, SKP2, RNF2, UBE2J1
	**GO Term Biological Process**	GO:0018105	peptidyl-serine phosphorylation	6.84E-04	PRKCI, EPO, CHEK2, PRKCD, EIF2AK3, MAPKAPK5, HIPK2, TAF1, MAPK3

	**GO Term Biological Process**	GO:0070423	nucleotide-binding oligomerization domain containing signaling pathway	6.91E-04	CHUK, TRAF6, UBE2V1, MAP3K7, MAP2K6
	**GO Term Biological Process**	GO:0007049	cell cycle	7.07E-04	RBL2, RBL1, USP22, PRKCD, GPS2, E2F2, E2F3, CCAR2, TAF1, MAPK3, ARF6, HCFC1
	**GO Term Molecular Function**	GO:0043422	protein kinase B binding	7.92E-04	TRAF6, PDE3B, SRSF1, RARA
	**GO Term Biological Process**	GO:0050680	negative regulation of epithelial cell proliferation	7.97E-04	RB1, DLG1, CDK6, PAX6, SOX9, NKX3-1
	**GO Term Cellular Component**	GO:0005794	Golgi apparatus	8.83E-04	MACF1, STX16, CUL3, PDE3B, PSEN1, TGOLN2, CLN3, SNX1, CHEK2, TOPBP1, MAPK3, PRNP, SORT1, AP3B1, SORL1, BACE1, GNPTG, DLG1, GOLPH3, CTTN, ID1, CAT, ALB, KDELR2, ARF6

	**GO Term Molecular Function**	GO:0016301	kinase activity	9.77E-04	STK11, CDKN1B, CHKA, CDKN2A, PRKAR1A, CCNH, PIK3C3, PIK3CB, SKP2, TAF1, PDK1, MAPK3
	**KEGG Pathway**	hsa04910	Insulin signaling pathway	0.00100436	RPTOR, SREBF1, PRKCI, IRS1, PRKAR1A, INSR, PDE3B, PIK3CB, PCK1, EIF4E, HK2, MAPK3
	**GO Term Molecular Function**	GO:0098641	cadherin binding involved in cell-cell adhesion	0.00105497	MACF1, HSPA8, TXNDC9, IDH1, PARVA, NDRG1, HCFC1, SDCBP, DLG1, SNX1, PLCB3, CTTN, PFN1, TJP2

	**GO Term Molecular Function**	GO:0005524	ATP binding	0.00110779	MOCS3, UBE2D1, IDE, PIK3CB, GPHN, HK2, UBE2J1, STK11, MAT2A, CHEK2, PIM2, MAP3K7, MAPK3, MAP2K6, PDK1, HSPA8, PRKCI, KSR1, CHKA, CHUK, INSR, PRKCD, EIF2AK3, UBE2A, UBE2G2, HIPK2, CDK8, CDK6, UBA3, SUCLG2, MAPKAPK5, PIK3C3, ALDH18A1, DNA2, CDK12, FGFR1, TAF1
	**GO Term Biological Process**	GO:0070555	response to interleukin-1	0.00111821	PRKCI, EPO, TRAF6, MTHFR, MYD88
	**GO Term Biological Process**	GO:0097284	hepatocyte apoptotic process	0.00112234	RB1, DNMT3A, FAS, ARF6
	**KEGG Pathway**	hsa04350	TGF-beta signaling pathway	0.00120688	SMAD2, TFDP1, RBL1, MYC, ID1, CUL1, E2F4, E2F5, MAPK3
	**GO Term Cellular Component**	GO:0045121	membrane raft	0.00122209	BACE1, CLN3, SDCBP, PRNP, DLG1, TRADD, FAS, FADD, PSEN1, BIRC3

	**KEGG Pathway**	hsa04668	TNF signaling pathway	0.0012382	CREB1, CHUK, TRADD, FAS, FADD, PIK3CB, MAP3K7, MAP2K6, BIRC3, MAPK3
	**GO Term Molecular Function**	GO:0003700	transcription factor activity, sequence-specific DNA binding	0.00131143	RB1, KDM5B, CSRNP2, MYC, E2F2, E2F3, SOX9, E2F4, E2F5, NKX3-1, ESRRA, SMAD2, SREBF1, TCF7L1, TBP, NR2F1, NR1H4, NR1H3, PAX6, HCFC1, MLXIPL, CREB1, TFDP1, SP4, ID1, RARA
	**GO Term Cellular Component**	GO:0010008	endosome membrane	0.00134005	BACE1, SNX1, SORT1, TRAF6, INSR, VPS41, VPS37A, VPS26A, MAP3K7, MYD88
	**GO Term Biological Process**	GO:0033189	response to vitamin A	0.00143977	EPO, DNMT3A, CAT, RARA
	**GO Term Biological Process**	GO:0001657	ureteric bud development	0.00148901	SMAD2, CAT, RARA, SDC1, FGFR1

	**GO Term Biological Process**	GO:0043488	regulation of mRNA stability	0.00151024	HSPA8, PSMA3, PSMA4, PSMD11, PSMD14, APEX1, FMR1, PRKCD
	**GO Term Biological Process**	GO:0043065	positive regulation of apoptotic process	0.00154647	CDKN2A, TRADD, HIP1R, HTRA2, PSEN1, CCAR2, DUSP6, IGF2R, CREB1, TRAF6, FAS, FADD, MAP2K6
	**GO Term Molecular Function**	GO:0061631	ubiquitin conjugating enzyme activity	0.00155023	UBE2D1, UBE2A, UBE2G2, TAF1, UBE2J1
	**KEGG Pathway**	hsa05162	Measles	0.00157718	HSPA8, CDKN1B, CDK6, CHUK, TRAF6, EIF2AK3, FAS, PIK3CB, MAP3K7, MYD88
	**GO Term Biological Process**	GO:0050821	protein stabilization	0.00160797	CREB1, CDKN2A, CHEK2, PRKCD, HIP1R, PIM2, PFN1, TAF1, HCFC1

	**GO Term Cellular Component**	GO:0005783	endoplasmic reticulum	0.001761	SREBF1, PRNP, KSR1, PRKCD, PDE3B, EIF2AK3, HTRA2, RRAS2, HMGCR, PSEN1, UBE2G2, TFPI, CYP19A1, SORL1, EEF1B2, CLN3, DLG1, TMX1, APEX1, CAT, ALB, KDELR2, PROM1
	**GO Term Cellular Component**	GO:0005913	cell-cell adherens junction	0.0019542	MACF1, HSPA8, TXNDC9, IDH1, PARVA, NDRG1, HCFC1, SDCBP, DLG1, SNX1, PLCB3, CTTN, PFN1, TJP2
	**GO Term Biological Process**	GO:0042593	glucose homeostasis	0.00197913	MLXIPL, STK11, IRS1, INSR, NR1H4, PAX6, PCK1
	**GO Term Biological Process**	GO:0010629	negative regulation of gene expression	0.00203707	RB1, SMAD2, RBL2, KDM4A, CREB1, RBL1, TAF1, NKX3-1
	**KEGG Pathway**	hsa05206	MicroRNAs in cancer	0.00205779	RPTOR, CDKN1B, SLC45A3, CDK6, IRS1, CDKN2A, MYC, DNMT3A, IGF2BP1, E2F2, E2F3, MCL1

	**KEGG Pathway**	hsa04066	HIF-1 signaling pathway	0.00206539	CDKN1B, EPO, INSR, SERPINE1, PIK3CB, EIF4E, HK2, PDK1, MAPK3
	**GO Term Molecular Function**	GO:0046982	protein heterodimerization activity	0.00217227	SMAD2, CHUK, CUL3, FMR1, H2AFX, METTL3, MLXIPL, SDCBP, SNX1, UBA3, RARA, SUCLG2, SOX9, MCL1, TAF1
	**GO Term Molecular Function**	GO:0046983	protein dimerization activity	0.00221102	SREBF1, NCOA2, NUP210, MYC, E2F2, E2F3, E2F4, E2F5
	**GO Term Biological Process**	GO:0010628	positive regulation of gene expression	0.00221678	KDM4A, KDM5B, MED1, CDK6, MYC, ID1, EIF2AK3, PAX6, PIK3CB, NKX3-1, HCFC1
	**KEGG Pathway**	hsa05145	Toxoplasmosis	0.0022463	CASP9, HSPA8, CHUK, TRAF6, MAP3K7, MYD88, MAP2K6, BIRC3, MAPK3
	**GO Term Biological Process**	GO:0071222	cellular response to lipopolysaccharide	0.00227266	GFI1, TRAF6, SERPINE1, NR1H4, RARA, NR1H3, TFPI
	**GO Term Biological Process**	GO:0007623	circadian rhythm	0.00235698	SREBF1, CREB1, METTL3, ID1, SERPINE1, FAS

	**GO Term Biological Process**	GO:0031145	anaphase-promoting complex-dependent catabolic process	0.00243116	PSMA3, PSMA4, PSMD11, PSMD14, CUL3, UBE2D1, SKP2
	**GO Term Biological Process**	GO:0045787	positive regulation of cell cycle	0.0024724	CDKN1B, MYC, RARA, FGFR1, HCFC1
	**GO Term Biological Process**	GO:0046777	protein autophosphorylation	0.00251856	STK11, CHEK2, INSR, EIF2AK3, MAPKAPK5, PIM2, CDK12, FGFR1, TAF1
	**GO Term Biological Process**	GO:0006338	chromatin remodeling	0.00259785	RB1, PBRM1, MYC, MORF4L2, SOX9, DMAP1, BPTF
	**KEGG Pathway**	hsa05168	Herpes simplex infection	0.00261402	TBP, CHUK, TRAF6, SRSF1, CUL1, EIF2AK3, FAS, FADD, SKP2, MAP3K7, MYD88, HCFC1
	**GO Term Biological Process**	GO:0051092	positive regulation of NF-kappaB transcription factor activity	0.00269611	PRKCI, CHUK, TRAF6, TRADD, CAT, UBE2V1, MAP3K7, MYD88
	**GO Term Biological Process**	GO:0043123	positive regulation of I-kappaB kinase/NF-kappaB signaling	0.00276712	CHUK, TRAF6, TRADD, PIM2, FADD, UBE2V1, MAP3K7, MYD88, BIRC3
	**GO Term Biological Process**	GO:0043433	negative regulation of sequence-specific DNA binding transcription factor activity	0.00278616	RB1, PRNP, SUFU, ID1, RNF2, GLIS2

	**KEGG Pathway**	hsa04930	Type II diabetes mellitus	0.00298516	IRS1, INSR, PRKCD, PIK3CB, HK2, MAPK3
	**GO Term Biological Process**	GO:0006915	apoptotic process	0.003029	RB1, CSRNP2, CDKN2A, EPO, TRADD, PRKCD, HIP1R, CUL1, CASP9, DAD1, FAS, PIM2, FADD, MAP3K7, MYD88, MAP2K6, BIRC3, MAPK3
	**GO Term Biological Process**	GO:0030522	intracellular receptor signaling pathway	0.00310046	ESRRA, NCOA2, NR2F1, NR1H4, NR1H3
	**GO Term Biological Process**	GO:0043550	regulation of lipid kinase activity	0.00311241	RB1, RBL2, RBL1
	**KEGG Pathway**	hsa04620	Toll-like receptor signaling pathway	0.00344038	CHUK, TRAF6, FADD, PIK3CB, MAP3K7, MYD88, MAP2K6, MAPK3
	**GO Term Biological Process**	GO:0000278	mitotic cell cycle	0.00345188	TFDP1, CUL3, DNMT3A, E2F4, RNF2
	**KEGG Pathway**	hsa05221	Acute myeloid leukemia	0.00388989	TCF7L1, CHUK, MYC, RARA, PIM2, PIK3CB, MAPK3
	**KEGG Pathway**	hsa04210	Apoptosis	0.00429955	CASP9, CHUK, TRADD, FAS, FADD, PIK3CB, BIRC3

	**GO Term Biological Process**	GO:0060770	negative regulation of epithelial cell proliferation involved in prostate gland development	0.00461335	STK11, CDKN1B, NKX3-1
	**GO Term Molecular Function**	GO:0003682	chromatin binding	0.00463125	BRD3, SMAD2, SREBF1, NCOA2, MED1, PBRM1, FMR1, DNMT3A, PAX6, RNF2, HCFC1, WAC, RCC1, SOX9
	**GO Term Cellular Component**	GO:0005856	cytoskeleton	0.00464619	MACF1, TRADD, HIP1R, HTRA2, PARVA, GPHN, SDCBP, CTTN, PFN1, UTRN, FERMT2, MAP2K6, MAPK3
	**KEGG Pathway**	hsa04152	AMPK signaling pathway	0.00484507	RPTOR, SREBF1, STK11, CREB1, IRS1, INSR, HMGCR, PIK3CB, PCK1, MAP3K7
	**GO Term Molecular Function**	GO:0016922	ligand-dependent nuclear receptor binding	0.00486972	NCOA2, MED1, NR1H4, UBA3
	**GO Term Molecular Function**	GO:0000978	RNA polymerase II core promoter proximal region sequence-specific DNA binding	0.00491181	ESRRA, SMAD2, SREBF1, NCOA2, MED1, MLXIPL, CREB1, TBP, MYC, NR1H3, PAX6, E2F4

	**GO Term Biological Process**	GO:1901796	regulation of signal transduction by p53 class mediator	0.0049419	STK11, TBP, CHEK2, MAPKAPK5, TOPBP1, DNA2, HIPK2, TAF1
	**GO Term Biological Process**	GO:0008285	negative regulation of cell proliferation	0.00520422	RB1, SMAD2, PBRM1, CDKN1B, CDKN2A, CUL1, NDRG1, STK11, CDK6, RARA, CD9, PIM2, NKX3-1
	**GO Term Biological Process**	GO:0033209	tumor necrosis factor-mediated signaling pathway	0.00562965	PSMA3, PSMA4, PSMD11, PSMD14, TRADD, FAS, BIRC3
	**KEGG Pathway**	hsa05214	Glioma	0.00572027	RB1, CDK6, CDKN2A, E2F2, E2F3, PIK3CB, MAPK3
	**GO Term Biological Process**	GO:0007265	Ras protein signal transduction	0.00581351	RB1, SDCBP, KSR1, CDKN2A, RRAS2, MAPKAPK5
	**GO Term Cellular Component**	GO:0031264	death-inducing signaling complex	0.00588087	TRADD, FAS, FADD
	**GO Term Biological Process**	GO:1900182	positive regulation of protein localization to nucleus	0.0060222	STK11, CDKN2A, EIF2AK3, GLIS2
	**GO Term Biological Process**	GO:0032435	negative regulation of proteasomal ubiquitin-dependent protein catabolic process	0.0060222	SDCBP, WAC, CCAR2, SENP1
	**KEGG Pathway**	hsa05219	Bladder cancer	0.00625155	RB1, CDKN2A, MYC, E2F2, E2F3, MAPK3

	**GO Term Molecular Function**	GO:0003707	steroid hormone receptor activity	0.00665594	ESRRA, NR2F1, NR1H4, RARA, NR1H3
	**GO Term Biological Process**	GO:0098609	cell-cell adhesion	0.00666275	HSPA8, SDCBP, MACF1, SNX1, PLCB3, TXNDC9, CTTN, IDH1, PFN1, NDRG1, TJP2, HCFC1
	**GO Term Biological Process**	GO:0071347	cellular response to interleukin-1	0.00669213	MYC, SOX9, PCK1, TFPI, NKX3-1
	**GO Term Cellular Component**	GO:0016020	membrane	0.00687469	PSMD11, STX16, CUL3, FMR1, PDE3B, HTRA2, PSEN1, CYP19A1, LCLAT1, HK2, CLN3, SDCBP, STK11, SNX1, LBR, MCL1, UBL4A, MED1, HSPA8, KSR1, NUP210, INSR, PRKCD, INTS2, COG2, EIF2AK3, RRAS2, AP3B1, SDHC, SORL1, IGF2R, HCFC1, PLCB3, DAD1, PRKAR1A, AGO4, VPS41, PEX5, CAT, CD9, FAS, SDC1, PIK3C3, PFN1, UTRN, ARF6

	**KEGG Pathway**	hsa05166	HTLV-I infection	0.0068758	RB1, SMAD2, TBP, CHUK, CDKN2A, RRAS2, GPS2, PIK3CB, DLG1, CREB1, CHEK2, MYC, E2F2, E2F3
	**KEGG Pathway**	hsa05203	Viral carcinogenesis	0.00692377	RB1, CDKN1B, TBP, CDKN2A, TRADD, PIK3CB, RBL2, DLG1, CREB1, RBL1, CDK6, SKP2, MAPK3
	**GO Term Molecular Function**	GO:0004871	signal transducer activity	0.0072138	PLCB3, CHKA, IRS1, SUFU, TRADD, FAS, GNB5, DKK1, BCAR1
	**GO Term Biological Process**	GO:0032869	cellular response to insulin stimulus	0.00750318	PRKCI, IRS1, MYC, INSR, PDE3B, PCK1
	**GO Term Cellular Component**	GO:0031985	Golgi cisterna	0.00775238	GOLPH3, STX16, SORL1
	**GO Term Biological Process**	GO:0009636	response to toxic substance	0.00797383	CHUK, CHKA, DNMT3A, FAS, SDC1, MAPK3
	**GO Term Molecular Function**	GO:0030295	protein kinase activator activity	0.00821258	RPTOR, STK11, EPO, NKX3-1
	**GO Term Molecular Function**	GO:0050661	NADP binding	0.00821258	IDH1, CAT, MTHFR, HMGCR

	**GO Term Biological Process**	GO:0006975	DNA damage induced protein phosphorylation	0.00840934	CHEK2, MAP2K6, MAPK3
	**GO Term Biological Process**	GO:0071300	cellular response to retinoic acid	0.00854393	MYC, RARA, HTRA2, SOX9, PCK1
	**GO Term Biological Process**	GO:0043401	steroid hormone mediated signaling pathway	0.00854393	ESRRA, NR2F1, NR1H4, RARA, NR1H3
	**KEGG Pathway**	hsa04931	Insulin resistance	0.00869408	SREBF1, MLXIPL, CREB1, IRS1, INSR, PRKCD, NR1H3, PIK3CB, PCK1
	**GO Term Cellular Component**	GO:0009986	cell surface	0.0092191	PRNP, SORT1, EPO, IDE, PSEN1, TFPI, IGF2R, BACE1, RARA, FAS, SDC1, PROM1, FERMT2
	**GO Term Molecular Function**	GO:0042803	protein homodimerization activity	0.00951849	SMAD2, CHUK, CHKA, CUL3, IDH1, FMR1, HIP1R, EIF2AK3, HMGCR, IDE, MLXIPL, SDCBP, GNPTG, SNX1, CHEK2, ID1, CAT, MCL1, FGFR1
	**GO Term Biological Process**	GO:0035666	TRIF-dependent toll-like receptor signaling pathway	0.00987968	CHUK, UBE2D1, FADD, BIRC3

	**GO Term Biological Process**	GO:0048661	positive regulation of smooth muscle cell proliferation	0.0099478	MYC, TRAF6, HMGCR, SKP2, MYD88
	**GO Term Cellular Component**	GO:0005768	endosome	0.00998355	BACE1, TGOLN2, PRKCI, GOLPH3, CDKN1B, VPS26A, SORL1, IGF2R, ARF6
	**GO Term Biological Process**	GO:0051437	positive regulation of ubiquitin-protein ligase activity involved in regulation of mitotic cell cycle transition	0.01006179	PSMA3, PSMA4, PSMD11, PSMD14, CUL1, UBE2D1
	**GO Term Molecular Function**	GO:0048027	mRNA 5'-UTR binding	0.01017059	FMR1, IGF2BP1, RARA
	**GO Term Molecular Function**	GO:0016303	1-phosphatidylinositol-3-kinase activity	0.0102827	IRS1, PIK3C3, PIK3CB, FGFR1
	**GO Term Molecular Function**	GO:0004674	protein serine/threonine kinase activity	0.01087825	PRKCI, PRKCD, EIF2AK3, HIPK2, CDK8, STK11, CHEK2, MAPKAPK5, PIM2, MAP3K7, MAP2K6, TAF1, MAPK3
	**GO Term Biological Process**	GO:0043967	histone H4 acetylation	0.01102248	MORF4L2, USP22, DMAP1, BRCA2
	**KEGG Pathway**	hsa00020	Citrate cycle (TCA cycle)	0.01104425	FH, IDH1, SUCLG2, SDHC, PCK1

	**GO Term Biological Process**	GO:0042981	regulation of apoptotic process	0.01105491	CASP9, FAS, FADD, SOX9, SKP2, NDRG1, BCAR1, MCL1, BIRC3
	**GO Term Biological Process**	GO:0032868	response to insulin	0.01149332	SORT1, IRS1, CAT, GGH, PCK1
	**GO Term Molecular Function**	GO:0019901	protein kinase binding	0.01188889	SREBF1, CDKN2A, PRKCD, PAX6, CASP9, RPTOR, DLG1, CHEK2, SUFU, TRAF6, UTRN, BCAR1, MAP2K6
	**GO Term Biological Process**	GO:1902042	negative regulation of extrinsic apoptotic signaling pathway via death domain receptors	0.01223849	TRADD, SERPINE1, FAS, FADD
	**GO Term Biological Process**	GO:0097192	extrinsic apoptotic signaling pathway in absence of ligand	0.01223849	CASP9, FAS, FADD, MCL1
	**GO Term Biological Process**	GO:0045931	positive regulation of mitotic cell cycle	0.01223849	USP22, BRCA2, EIF4E, NKX3-1
	**GO Term Biological Process**	GO:0007249	I-kappaB kinase/NF-kappaB signaling	0.01232074	CHUK, TRAF6, TRADD, MAP3K7, BIRC3
	**GO Term Molecular Function**	GO:0043560	insulin receptor substrate binding	0.0125674	INSR, PRKCD, PIK3CB

	**GO Term Cellular Component**	GO:0048471	perinuclear region of cytoplasm	0.01303172	SORT1, STX16, FMR1, PRKCD, HIP1R, EIF2AK3, NDRG1, IGF2R, DLG1, INF2, APEX1, TRAF6, IGF2BP1, RARA, AGGF1, EIF4E, BPTF
	**GO Term Biological Process**	GO:0045821	positive regulation of glycolytic process	0.01319855	MLXIPL, MYC, INSR
	**GO Term Biological Process**	GO:0045723	positive regulation of fatty acid biosynthetic process	0.01319855	MLXIPL, SLC45A3, NR1H3
	**GO Term Biological Process**	GO:0006099	tricarboxylic acid cycle	0.01352843	FH, IDH1, SUCLG2, SDHC
	**GO Term Molecular Function**	GO:0001047	core promoter binding	0.01379464	RB1, MED1, E2F2, E2F3, NKX3-1
	**GO Term Molecular Function**	GO:0043565	sequence-specific DNA binding	0.0139103	ESRRA, TCF7L1, CSRNP2, NR2F1, NR1H4, NR1H3, PAX6, CREB1, MYC, RARA, BPTF, TAF1, NKX3-1
	**GO Term Biological Process**	GO:0051291	protein heterooligomerization	0.01408772	MAT2A, TRADD, FADD, IDE, BIRC3

	**GO Term Biological Process**	GO:0007179	transforming growth factor beta receptor signaling pathway	0.01456664	SMAD2, CREB1, ID1, MAP3K7, FERMT2, HIPK2
	**GO Term Cellular Component**	GO:0005802	trans-Golgi network	0.01463195	BACE1, TGOLN2, CLN3, GOLPH3, STX16, SORL1, IGF2R
	**GO Term Cellular Component**	GO:0001741	XY body	0.01471797	H2AFX, DNMT3A, UBE2A
	**GO Term Cellular Component**	GO:0035631	CD40 receptor complex	0.01471797	CHUK, TRAF6, HTRA2
	**GO Term Molecular Function**	GO:0004879	RNA polymerase II transcription factor activity, ligand-activated sequence-specific DNA binding	0.01525628	ESRRA, NR2F1, NR1H4, NR1H3
	**GO Term Biological Process**	GO:0035729	cellular response to hepatocyte growth factor stimulus	0.015942	MED1, CREB1, BCAR1
	**GO Term Biological Process**	GO:0030837	negative regulation of actin filament polymerization	0.015942	PRKCD, HIP1R, PFN1
	**GO Term Biological Process**	GO:0071260	cellular response to mechanical stimulus	0.01600772	FAS, FADD, SOX9, MYD88, MAPK3
	**KEGG Pathway**	hsa04150	mTOR signaling pathway	0.0162279	RPTOR, STK11, IRS1, PIK3CB, EIF4E, MAPK3

	**GO Term Molecular Function**	GO:0032403	protein complex binding	0.01670111	RPTOR, SREBF1, CDKN1B, MYC, APEX1, TRADD, COG2, MTHFR, FADD
	**KEGG Pathway**	hsa04120	Ubiquitin mediated proteolysis	0.01749836	CUL3, TRAF6, CUL1, UBA3, UBE2D1, UBE2A, UBE2G2, SKP2, UBE2J1, BIRC3
	**GO Term Biological Process**	GO:0007165	signal transduction	0.01750524	CHKA, EPO, IRS1, TRADD, PRKCD, PDE3B, NR2F1, NR1H4, PIK3CB, TXN, SORL1, IGF2R, CREB1, CDKN2AIPNL, SUFU, RARA, FAS, MAPKAPK5, FADD, SOX9, GNB5, MYD88, MAP2K6
	**KEGG Pathway**	hsa05230	Central carbon metabolism in cancer	0.01759879	MYC, PIK3CB, HK2, FGFR1, PDK1, MAPK3
	**GO Term Biological Process**	GO:0036092	phosphatidylinositol-3-phosphate biosynthetic process	0.01784717	IRS1, PIK3C3, PIK3CB, FGFR1
	**GO Term Biological Process**	GO:0060070	canonical Wnt signaling pathway	0.01808455	STK11, TCF7L1, MYC, SDC1, PSEN1

	**KEGG Pathway**	hsa04380	Osteoclast differentiation	0.01855482	CREB1, CHUK, TRAF6, FHL2, PIK3CB, MAP3K7, MAP2K6, MAPK3
	**GO Term Cellular Component**	GO:0009898	cytoplasmic side of plasma membrane	0.01890374	DLG1, CHUK, TRAF6, HTRA2
	**GO Term Biological Process**	GO:0048009	insulin-like growth factor receptor signaling pathway	0.01890593	IRS1, EIF2AK3, IGF2R
	**GO Term Biological Process**	GO:0097296	activation of cysteine-type endopeptidase activity involved in apoptotic signaling pathway	0.01890593	TRADD, FAS, FADD
	**GO Term Biological Process**	GO:0051262	protein tetramerization	0.01943767	FH, PEX5, CAT, HMGCR
	**GO Term Molecular Function**	GO:0008022	protein C-terminus binding	0.02007318	SDCBP, DLG1, KSR1, ID1, PEX5, SDC1, TOPBP1, BRCA2

	**GO Term Cellular Component**	GO:0005739	mitochondrion	0.02051707	FH, HTRA2, IDE, PSEN1, MAT2B, TXN, SYNE2, CASP9, CLN3, STK11, MYC, MCL1, MAPK3, PDK1, PRNP, CDKN2A, CCDC58, IDH1, HIP1R, SIRT5, SDHC, HCFC1, GOLPH3, TFDP1, APEX1, TRAF6, PEX5, CAT, SUCLG2, ALDH18A1, SLC25A12
	**GO Term Biological Process**	GO:1901215	negative regulation of neuron death	0.02110401	CREB1, EPO, HTRA2, SORL1
	**GO Term Biological Process**	GO:0071363	cellular response to growth factor stimulus	0.02110401	INSR, CAT, HTRA2, PFN1
	**GO Term Biological Process**	GO:0071364	cellular response to epidermal growth factor stimulus	0.02110401	MED1, MYC, ID1, SOX9
	**GO Term Biological Process**	GO:0008625	extrinsic apoptotic signaling pathway via death domain receptors	0.02110401	SORT1, TRADD, FAS, FADD
	**GO Term Biological Process**	GO:0030838	positive regulation of actin filament polymerization	0.02110401	DLG1, CTTN, PFN1, ARF6

	**GO Term Biological Process**	GO:0006977	DNA damage response, signal transduction by p53 class mediator resulting in cell cycle arrest	0.02150113	RBL2, CDKN1B, TFDP1, CHEK2, E2F4
	**KEGG Pathway**	hsa05152	Tuberculosis	0.02174839	CASP9, CREB1, KSR1, TRAF6, TRADD, PIK3C3, FADD, MYD88, MAPK3
	**GO Term Biological Process**	GO:0044257	cellular protein catabolic process	0.0220816	CHEK2, HTRA2, UBE2G2
	**GO Term Biological Process**	GO:0006978	DNA damage response, signal transduction by p53 class mediator resulting in transcription of p21 class mediator	0.0220816	CHEK2, BRCA2, HIPK2
	**GO Term Biological Process**	GO:0007219	Notch signaling pathway	0.02211821	CDKN1B, CDK6, MYC, NR1H4, SOX9, PSEN1
	**GO Term Biological Process**	GO:0070534	protein K63-linked ubiquitination	0.02284629	RNF126, CDKN2A, TRAF6, UBE2V1
	**GO Term Biological Process**	GO:0043161	proteasome-mediated ubiquitin-dependent protein catabolic process	0.02290821	PSMA3, RNF126, PSMA4, PSMD11, PSMD14, CUL3, CUL1, UBE2D1, UBE2A
	**GO Term Biological Process**	GO:0006357	regulation of transcription from RNA polymerase II promoter	0.02319085	RB1, BRD3, SREBF1, TCF7L1, EPO, FHL2, RBL2, MLXIPL, CREB1, RBL1, TFDP1, PRKAR1A, SP4

	**GO Term Cellular Component**	GO:0030140	trans-Golgi network transport vesicle	0.02353955	TGOLN2, SORT1, IGF2R
	**GO Term Molecular Function**	GO:0004842	ubiquitin-protein transferase activity	0.023824	ZYG11B, CUL3, TRAF6, CUL1, UBE2D1, PAX6, UBE2A, UBE2G2, SKP2, RNF2, BIRC3
	**GO Term Molecular Function**	GO:0008353	RNA polymerase II carboxy-terminal domain kinase activity	0.02427189	CDK8, CCNH, CDK12
	**GO Term Molecular Function**	GO:0043621	protein self-association	0.0248574	VPS41, ID1, MYD88, NKX3-1
	**GO Term Biological Process**	GO:0006898	receptor-mediated endocytosis	0.0251096	CLN3, CTTN, ALB, HIP1R, SORL1, IGF2R
	**GO Term Biological Process**	GO:0045879	negative regulation of smoothened signaling pathway	0.02546049	RB1, SUFU, GLIS2
	**GO Term Cellular Component**	GO:0005730	nucleolus	0.025801	ESRRA, HSPA8, KDM4A, MED1, CDKN2A, FMR1, PIK3CB, RBL2, CDK8, CDKN2AIPNL, TMX1, EWSR1, MYC, APEX1, MORF4L2, TRAF6, CDK12, SKP2, E2F5, KDM7A, BCAR1, TAF1

	**GO Term Biological Process**	GO:0007346	regulation of mitotic cell cycle	0.02655861	RB1, SDCBP, MYC, PIM2
	**GO Term Biological Process**	GO:0032526	response to retinoic acid	0.02655861	SREBF1, RARA, DKK1, IGF2R
	**GO Term Cellular Component**	GO:0031527	filopodium membrane	0.02685659	UTRN, SYNE2, ARF6
	**GO Term Molecular Function**	GO:0003714	transcription corepressor activity	0.0269059	KDM5B, SUFU, APEX1, NR1H4, RARA, GPS2, DMAP1, HIPK2
	**GO Term Cellular Component**	GO:0032588	trans-Golgi network membrane	0.02707113	TGOLN2, STX16, AP4E1, COG2, IGF2R
	**KEGG Pathway**	hsa01200	Carbon metabolism	0.0282973	FH, PSAT1, IDH1, CAT, SUCLG2, MTHFR, SDHC, HK2
	**GO Term Biological Process**	GO:0030521	androgen receptor signaling pathway	0.02852843	RB1, MED1, FHL2, NKX3-1
	**GO Term Biological Process**	GO:0071398	cellular response to fatty acid	0.02903433	SREBF1, CREB1, NR1H4

	**KEGG Pathway**	hsa04010	MAPK signaling pathway	0.02926787	HSPA8, CHUK, MYC, TRAF6, RRAS2, FAS, MAPKAPK5, MAP3K7, DUSP6, FGFR1, MAP2K6, MAPK3
	**GO Term Cellular Component**	GO:0005782	peroxisomal matrix	0.02937749	IDH1, PEX5, CAT, IDE
	**KEGG Pathway**	hsa05010	Alzheimer's disease	0.03035267	BACE1, CASP9, PLCB3, EIF2AK3, FAS, SDHC, FADD, IDE, PSEN1, MAPK3
	**KEGG Pathway**	hsa05210	Colorectal cancer	0.03136693	SMAD2, CASP9, TCF7L1, MYC, PIK3CB, MAPK3
	**GO Term Biological Process**	GO:0034644	cellular response to UV	0.03269424	CASP9, MYC, FMR1, TAF1
	**GO Term Biological Process**	GO:0007254	JNK cascade	0.03269424	TRAF6, GPS2, MAP3K7, MYD88
	**GO Term Biological Process**	GO:0001894	tissue homeostasis	0.03279509	RB1, STK11, SOX9
	**GO Term Biological Process**	GO:1902041	regulation of extrinsic apoptotic signaling pathway via death domain receptors	0.03279509	TRADD, FAS, FADD

	**GO Term Biological Process**	GO:0070371	ERK1 and ERK2 cascade	0.03279509	MED1, SOX9, MAPK3
	**GO Term Cellular Component**	GO:0045171	intercellular bridge	0.03342417	ESRRA, PRKCI, SP4, PIK3CB
	**GO Term Molecular Function**	GO:0044212	transcription regulatory region DNA binding	0.0335031	TCF7L1, CREB1, TBP, GFI1, NR1H3, GLIS2, TAF1, NKX3-1
	**GO Term Cellular Component**	GO:0048269	methionine adenosyltransferase complex	0.03422257	MAT2A, MAT2B
	**GO Term Molecular Function**	GO:0051087	chaperone binding	0.03438657	UBL4A, PRNP, CDKN1B, ALB, GNB5
	**GO Term Molecular Function**	GO:0032810	sterol response element binding	0.03478727	SREBF1, NR1H3
	**GO Term Molecular Function**	GO:0008798	beta-aspartyl-peptidase activity	0.03478727	BACE1, ASRGL1
	**GO Term Molecular Function**	GO:0016890	site-specific endodeoxyribonuclease activity, specific for altered base	0.03478727	APEX1, DNA2
	**GO Term Molecular Function**	GO:1990841	promoter-specific chromatin binding	0.03505098	RBL2, RBL1, E2F4
	**GO Term Molecular Function**	GO:0045296	cadherin binding	0.03505098	PSEN1, PROM1, NDRG1

	**GO Term Biological Process**	GO:0071456	cellular response to hypoxia	0.03560084	DNMT3A, PCK1, NDRG1, HIPK2, NKX3-1
	**GO Term Biological Process**	GO:0048015	phosphatidylinositol-mediated signaling	0.03560084	IRS1, AGO4, PIK3C3, PIK3CB, FGFR1
	**GO Term Biological Process**	GO:0030858	positive regulation of epithelial cell differentiation	0.03568395	PAX6, SOX9
	**GO Term Biological Process**	GO:0097049	motor neuron apoptotic process	0.03568395	FAS, FADD
	**GO Term Biological Process**	GO:0090090	negative regulation of canonical Wnt signaling pathway	0.03626885	PSMA3, PSMA4, PSMD11, PSMD14, CUL3, SOX9, DKK1
	**GO Term Biological Process**	GO:0030901	midbrain development	0.03673493	KDM7A, FGFR1, TAF1
	**GO Term Biological Process**	GO:0015721	bile acid and bile salt transport	0.03673493	NCOA2, ALB, NR1H4
	**GO Term Biological Process**	GO:0045861	negative regulation of proteolysis	0.03673493	CLN3, NR1H3, IDE
	**GO Term Molecular Function**	GO:0019902	phosphatase binding	0.0370441	SMAD2, DLG1, CSRNP2, MAPK3
	**GO Term Biological Process**	GO:0051436	negative regulation of ubiquitin-protein ligase activity involved in mitotic cell cycle	0.03724546	PSMA3, PSMA4, PSMD11, PSMD14, UBE2D1

	**GO Term Biological Process**	GO:0008286	insulin receptor signaling pathway	0.03724546	SREBF1, IRS1, INSR, IDE, BCAR1
	**GO Term Cellular Component**	GO:0031594	neuromuscular junction	0.03774518	DLG1, PRKAR1A, PSEN1, UTRN
	**KEGG Pathway**	hsa04115	p53 signaling pathway	0.03788823	CASP9, CDK6, CDKN2A, CHEK2, SERPINE1, FAS
	**KEGG Pathway**	hsa04621	NOD-like receptor signaling pathway	0.0383861	CHUK, TRAF6, MAP3K7, BIRC3, MAPK3
	**GO Term Molecular Function**	GO:0030332	cyclin binding	0.03898526	CDK6, CUL3, CDK12
	**GO Term Biological Process**	GO:0008630	intrinsic apoptotic signaling pathway in response to DNA damage	0.03950291	CASP9, CHEK2, HTRA2, MCL1
	**GO Term Biological Process**	GO:0016569	covalent chromatin modification	0.03960883	BRD3, RB1, RBL2, PBRM1, RBL1, BPTF
	**GO Term Biological Process**	GO:0042127	regulation of cell proliferation	0.03968142	ESRRA, RNF126, CDKN1B, FAS, E2F4, SOX9, NDRG1
	**GO Term Biological Process**	GO:0007420	brain development	0.03968142	MED1, ID1, CD9, FAS, BRCA2, NES, BPTF
	**GO Term Cellular Component**	GO:0005901	caveola	0.04000754	CLN3, IRS1, INSR, MAPK3

	**GO Term Cellular Component**	GO:0016607	nuclear speck	0.0407748	APEX1, SETD1B, METTL3, SRSF1, WAC, AGGF1, CDK12, GLIS2
	**GO Term Biological Process**	GO:0045930	negative regulation of mitotic cell cycle	0.04084626	DLG1, CDKN1B, NKX3-1
	**KEGG Pathway**	hsa04015	Rap1 signaling pathway	0.04094735	PRKCI, PLCB3, INSR, ID1, PIK3CB, PFN1, BCAR1, FGFR1, MAP2K6, MAPK3
	**GO Term Biological Process**	GO:1900034	regulation of cellular response to heat	0.04243812	RPTOR, HSPA8, NUP210, CCAR2, MAPK3
	**KEGG Pathway**	hsa04932	Non-alcoholic fatty liver disease (NAFLD)	0.04318813	SREBF1, MLXIPL, IRS1, INSR, EIF2AK3, FAS, NR1H3, SDHC, PIK3CB
	**GO Term Biological Process**	GO:0016477	cell migration	0.04329173	PRKCI, GOLPH3, CUL3, SDC1, PIK3CB, BCAR1, FGFR1
	**GO Term Biological Process**	GO:0070936	protein K48-linked ubiquitination	0.04440963	RNF126, UBE2D1, UBE2A, UBE2G2
	**GO Term Biological Process**	GO:0014068	positive regulation of phosphatidylinositol 3-kinase signaling	0.04440963	CAT, SOX9, FGFR1, NKX3-1
	**GO Term Biological Process**	GO:0008637	apoptotic mitochondrial changes	0.0451217	CDKN2A, HK2, MCL1

	**GO Term Biological Process**	GO:0008015	blood circulation	0.0451217	EPO, MTHFR, E2F4
	**GO Term Biological Process**	GO:0016055	Wnt signaling pathway	0.04580966	MACF1, TCF7L1, CUL3, CUL1, VPS26A, FERMT2, CCAR2
	**GO Term Cellular Component**	GO:0005770	late endosome	0.04595512	BACE1, HSPA8, CLN3, PIK3C3, IGF2R, MAPK3
	**GO Term Biological Process**	GO:0010468	regulation of gene expression	0.04611564	CLN3, CDK6, SORT1, MYC, DNMT3A
	**GO Term Biological Process**	GO:0010467	gene expression	0.04697147	GOLPH3, METTL3, IGF2BP1, FAS
	**GO Term Molecular Function**	GO:0005164	tumor necrosis factor receptor binding	0.04732389	TRAF6, TRADD, FADD
	**GO Term Cellular Component**	GO:0005769	early endosome	0.04770735	CLN3, SORT1, VPS41, VPS26A, SORL1, IGF2R, MAPK3, ARF6
	**KEGG Pathway**	hsa04960	Aldosterone-regulated sodium reabsorption	0.04810584	IRS1, INSR, PIK3CB, MAPK3
	**GO Term Biological Process**	GO:0002088	lens development in camera-type eye	0.04955405	MED1, DLG1, PAX6
	**GO Term Biological Process**	GO:0090398	cellular senescence	0.04955405	CDKN2A, H2AFX, PRKCD

	**GO Term Biological Process**	GO:0002053	positive regulation of mesenchymal cell proliferation	0.04955405	MYC, SOX9, FGFR1
	**GO Term Biological Process**	GO:0000083	regulation of transcription involved in G1/S transition of mitotic cell cycle	0.04955405	RB1, GFI1, E2F4
	**GO Term Biological Process**	GO:0010212	response to ionizing radiation	0.0496047	STK11, H2AFX, DNMT3A, TOPBP1

**3**	**GO Term Molecular Function**	GO:0044822	poly(A) RNA binding	1.17E-11	RBM27, DQX1, RTCA, EIF4A3, PPAN, MKI67, RBM7, SUPT16H, AQR, NCOA5, IMP3, DDX55, NGDN, DDX10, PATL1, RPP25L, DHX40, ZC3H11A, CCDC86, RPL27, SNRPA1, HNRNPH3, EIF1B, DDX28, SF3B6, NIP7, ZCCHC9, DDX23, NOL7, NOLC1, RPF1, ZFP36L1, RRP7A, REPIN1, RPS15A, DHX32, DHX35, RRS1, CFAP20, RBM10, TRMT10C, PTCD2, SSB, MYEF2, NIFK, CCDC137, HELZ, RRP1B, BYSL, NUDT21, HNRNPM, PAN3, PRKRA, TSR1, ESF1, RNPS1, TARDBP

	**GO Term Cellular Component**	GO:0005730	nucleolus	1.90E-08	MUS81, DDX28, RPL34, GEMIN2, NIP7, DDX23, ZCCHC9, NOL7, PPAN, NOLC1, MKI67, RPF1, PHF8, SMG6, COIL, ZNRD1, RRS1, SNRPB2, NUDT16, RPP14, EXOSC2, MDN1, BCAS2, UTP14C, NIFK, METTL1, CCDC137, IMP3, NGDN, APEX2, RRP1B, GTF2H5, BYSL, DHX40, RCL1, HNRNPM, POLR1A, TSR1, CCDC86, ESF1, POLR3K
	**GO Term Biological Process**	GO:0006364	rRNA processing	4.39E-07	IMP3, RPL34, POP4, EIF4A3, NOLC1, RRP1B, GTF2H5, BYSL, RRP7A, RCL1, RPS15A, TSR1, CCDC86, RPL27, RPP14, EXOSC2, MDN1, UTP14C

	**GO Term Molecular Function**	GO:0004004	ATP-dependent RNA helicase activity	2.31E-06	DHX40, DDX28, DDX59, DHX32, DDX55, DHX35, DQX1, DDX23, EIF4A3, DDX10
	**GO Term Biological Process**	GO:0000398	mRNA splicing, via spliceosome	2.97E-06	AQR, DQX1, SF3B6, DDX23, EIF4A3, NUDT21, HNRNPM, DHX32, DHX35, RBM41, PPIH, SNRPA1, SNRPB2, RNPS1, SNRPF, HNRNPH3, BCAS2
	**GO Term Molecular Function**	GO:0003723	RNA binding	5.27E-06	RPL34, POP4, RTCA, NIP7, SRBD1, RPF1, RPS15A, EIF2D, RPP14, EXOSC2, DDX59, SSB, NIFK, DDX55, DDX10, UPF3A, PUS7L, PATL1, RCL1, NUDT21, HNRNPM, RBMXL1, RNPS1, SNRPF, HNRNPH3, TARDBP
	**KEGG Pathway**	hsa03040	Spliceosome	6.45E-06	HNRNPM, AQR, RBMXL1, SF3B6, DDX23, EIF4A3, PPIH, SNRPA1, SNRPB2, SNRPF, BCAS2

	**GO Term Cellular Component**	GO:0030687	preribosome, large subunit precursor	7.43E-06	NIP7, PPAN, RRS1, CCDC86, RRP1B, MDN1, RPF1
	**GO Term Cellular Component**	GO:0005681	spliceosomal complex	1.24E-05	HNRNPM, AQR, DHX32, GEMIN2, DQX1, PPIH, SNRPA1, SNRPB2, HNRNPH3, SNRPF, BCAS2

	**GO Term Cellular Component**	GO:0005654	nucleoplasm	1.25E-05	MUS81, ATF1, GMEB2, DYRK2, ZBTB25, POP4, GEMIN2, RTCA, EIF4A3, PHF8, ALKBH3, COIL, ZMYM1, ZNRD1, ZMYM4, NUDT16, RBM7, BCAS2, PRMT6, TIPIN, SUPT16H, AQR, METTL1, IMP3, NAV2, SENP2, POLR1A, ZC3H11A, PPIH, SNRPA1, SNRPF, HNRNPH3, BCORL1, ZFPM1, CGGBP1, PSMD10, GTF3C2, PAXIP1, SF3B6, DDX23, NOLC1, RRP7A, RPS15A, TAF1B, SNRPB2, RPRD1B, RPP14, CFAP20, EXOSC2, ZBED5, RBM10, NR2C2AP, ZNF143, TRMT10C, PPIL2, MYEF2, BRF2, NIFK, UPF3A, GTF2H5, CLK2, BYSL, RCL1, NUDT21, HNRNPM, POLR3A, ERCC4, PRKRA, RFWD3, GID8, TSR1, RANGRF, ESF1, RNPS1, POLR3H, TARDBP, POLR3K, SLC2A4RG, NOP10

	**KEGG Pathway**	hsa03013	RNA transport	2.85E-05	POP4, GEMIN2, EIF3J, EIF4A3, UPF3A, RNPS1, NUP54, RPP14, SENP2, EIF1B, RPP25L

	**GO Term Cellular Component**	GO:0005634	nucleus	3.57E-05	ATF1, ANKRD37, CCNJ, GEMIN2, RTCA, PPAN, MKI67, ALKBH6, ALKBH3, ZNF84, ZNF569, EIF2D, SLC25A42, NUDT16, ZNF445, TIPIN, SUPT16H, METTL1, IMP3, ZBTB37, PASK, RPP25L, CAMKMT, RUFY1, MTAP, RBMXL1, KATNAL1, RUFY2, ZSCAN21, CCDC86, RPL27, ZNF711, ZFPM1, CGGBP1, PSMD10, URI1, ZNF395, PAXIP1, CREBL2, RPAP1, ZFP36L1, S100A13, RRS1, ZKSCAN2, RPRD1B, RBM10, MDN1, CTC1, UPF3A, HELZ, CLK2, SMU1, PAN2, APPBP2, TDP1, SP5, RNPS1, RCOR3, DCP1A, RCOR2, SLC2A4RG, NOP10, MUS81, GMEB2, DYRK2, SETD6, JRKL, PHF8, SMG6, COIL, NUDCD1, ZNF764, RBM7, BCAS2, PRMT6, DDX59, NCOA5, CTDSPL, RIPPLY3, POLR1A, ZNF514, HNRNPH3, DDX28, DTYMK, NIP7, DDX23, NOL7, ZNF7, PRIMPOL, REPIN1, RMND5A, TAF1B, APBB2, ZNF623, RPP14, DTD1, EXOSC2, ATE1, PCGF6, SSB, MYEF2, PPIL2, APEX2, ZNF76, RRP1B, PNRC2, BYSL, NUDT21, ERCC4, RFWD3, DNAJA3, ALMS1, TSR1, RANGRF, TARDBP, ZNF696

	**KEGG Pathway**	hsa03008	Ribosome biogenesis in eukaryotes	4.01E-05	RCL1, RRP7A, IMP3, POP4, NOP10, MDN1, RPP25L, UTP14C
	**GO Term Cellular Component**	GO:0071013	catalytic step 2 spliceosome	5.51E-05	HNRNPM, AQR, DHX35, SF3B6, DDX23, EIF4A3, SNRPA1, SNRPB2, SNRPF, BCAS2
	**GO Term Molecular Function**	GO:0003899	DNA-directed RNA polymerase activity	7.80E-05	POLR2M, ZNRD1, POLR3A, POLR1A, POLR3H, RPAP1, POLR3K
	**KEGG Pathway**	hsa00230	Purine metabolism	9.33E-05	ZNRD1, POLR3A, NME7, POLR1A, NPR2, PDE6D, POLR3H, AK4, NUDT16, POLR3K
	**GO Term Molecular Function**	GO:0000166	nucleotide binding	4.90E-04	RBM27, MYEF2, SSB, NIFK, SF3B6, UPF3A, RRP7A, HNRNPM, RBMXL1, RBM41, SNRPB2, RNPS1, HNRNPH3, RBM7, TARDBP, RBM10
	**GO Term Biological Process**	GO:0008380	RNA splicing	5.34E-04	RBMXL1, GEMIN2, DDX23, EIF4A3, SNRPA1, RNPS1, HNRNPH3, SNRPF, TARDBP, RBM10, BCAS2

	**GO Term Biological Process**	GO:0006397	mRNA processing	5.34E-04	PAN2, RBM27, NUDT21, PTCD2, RBMXL1, PAN3, GEMIN2, TARDBP, RBM10, BCAS2, ZFP36L1
	**GO Term Cellular Component**	GO:0030529	intracellular ribonucleoprotein complex	6.00E-04	HNRNPM, RBMXL1, SSB, DYRK2, EIF4A3, SNRPA1, SNRPB2, RPL27, HNRNPH3, ZFP36L1
	**KEGG Pathway**	hsa03020	RNA polymerase	6.78E-04	ZNRD1, POLR3A, POLR1A, POLR3H, POLR3K
	**GO Term Molecular Function**	GO:0003676	nucleic acid binding	8.62E-04	DDX23, ZCCHC9, EIF4A3, ZNF7, REPIN1, ZMYM1, ZNRD1, DHX35, ZNF84, ZNF569, ZNF623, RBM7, ZNF764, ZNF445, RBM10, ZNF143, DDX59, MYEF2, NIFK, DDX55, ZBTB37, ZNF76, PAN2, DHX40, ZSCAN21, ZNF514, RNPS1, HNRNPH3, TARDBP, POLR3K, ZNF696

	**GO Term Biological Process**	GO:0010501	RNA secondary structure unwinding	8.98E-04	DDX28, DDX59, DDX55, DDX23, EIF4A3, DDX10
	**GO Term Biological Process**	GO:0006139	nucleobase-containing compound metabolic process	0.00113234	SLC23A2, MTAP, ZNRD1, SRBD1, POLR3H, AK4
	**GO Term Molecular Function**	GO:0004386	helicase activity	0.0011929	DHX40, DHX32, DDX55, DDX23, EIF4A3, DDX10, HELZ, NAV2
	**KEGG Pathway**	hsa00240	Pyrimidine metabolism	0.00133878	DTYMK, ZNRD1, POLR3A, NME7, POLR1A, POLR3H, POLR3K
	**GO Term Biological Process**	GO:0000184	nuclear-transcribed mRNA catabolic process, nonsense-mediated decay	0.00152671	RPS15A, RPL34, EIF4A3, UPF3A, RNPS1, RPL27, DCP1A, SMG6, PNRC2
	**GO Term Cellular Component**	GO:0019013	viral nucleocapsid	0.00185693	HNRNPM, SNRPA1, SNRPB2, HNRNPH3, UTP14C
	**KEGG Pathway**	hsa03018	RNA degradation	0.00253956	PAN2, PAN3, NUDT16, DCP1A, EXOSC2, PATL1
	**GO Term Biological Process**	GO:0006383	transcription from RNA polymerase III promoter	0.00327865	GTF3C2, ZNF143, POLR3A, POLR3H, POLR3K

	**GO Term Cellular Component**	GO:0035145	exon-exon junction complex	0.00789149	EIF4A3, UPF3A, RNPS1, SMG6
	**GO Term Biological Process**	GO:0006264	mitochondrial DNA replication	0.01241257	DNAJA3, C10ORF2, PRIMPOL
	**GO Term Biological Process**	GO:0000290	deadenylation-dependent decapping of nuclear-transcribed mRNA	0.01241257	PAN3, DCP1A, PATL1
	**GO Term Cellular Component**	GO:0000932	cytoplasmic mRNA processing body	0.01408671	PAN2, PAN3, DCP1A, PATL1, ZFP36L1, PNRC2

	**GO Term Biological Process**	GO:0006351	transcription, DNA-templated	0.01625527	KANK2, GTF3C2, URI1, ZNF395, PAXIP1, ZBTB25, ZCCHC9, CREBL2, ZNF7, PHF8, ZNRD1, TAF1B, ZNF84, ZKSCAN2, ZNF569, ZNF623, ZNF764, ZNF445, PRMT6, PCGF6, MYEF2, NCOA5, ZBTB37, ZNF76, PNRC2, RIPPLY3, POLR3A, POLR1A, ZSCAN21, SP5, ZNF514, ESF1, ZNF711, RNPS1, POLR3H, RCOR3, BCORL1, ZFPM1, RCOR2, ZNF696, FRYL, SLC2A4RG, CGGBP1
	**GO Term Biological Process**	GO:0006362	transcription elongation from RNA polymerase I promoter	0.01666432	ZNRD1, TAF1B, POLR1A, GTF2H5

	**GO Term Molecular Function**	GO:0003677	DNA binding	0.01754684	MUS81, GTF3C2, ZNF395, GMEB2, ZBTB25, JRKL, MKI67, RPAP1, ZNF7, ZFP36L1, REPIN1, ZMYM1, ZMYM4, ZNF84, ZKSCAN2, ZNF569, ZNF623, TRIM26, ZNF764, ZNF445, DTD1, ZBED5, ZNF143, PCGF6, TIPIN, MYEF2, ZBTB37, ZNF76, APEX2, POLR3A, ERCC4, POLR1A, ZNF514, ZNF711, POLR3H, RCOR3, RCOR2, ZNF696, SLC2A4RG
	**GO Term Biological Process**	GO:0006363	termination of RNA polymerase I transcription	0.01832562	ZNRD1, TAF1B, POLR1A, GTF2H5
	**GO Term Molecular Function**	GO:0003729	mRNA binding	0.01939812	NUDT21, SSB, EIF4A3, TDRD7, NUDT16, DCP1A, ZFP36L1
	**KEGG Pathway**	hsa03015	mRNA surveillance pathway	0.01983604	NUDT21, EIF4A3, UPF3A, RNPS1, SMG6

	**GO Term Biological Process**	GO:0006359	regulation of transcription from RNA polymerase III promoter	0.02190012	ZNF143, BRF2, ZNF76
	**GO Term Biological Process**	GO:0006361	transcription initiation from RNA polymerase I promoter	0.02191451	ZNRD1, TAF1B, POLR1A, GTF2H5
	**GO Term Biological Process**	GO:0042254	ribosome biogenesis	0.02585911	RCL1, MTG1, RPL34, TSR1
	**GO Term Biological Process**	GO:0006396	RNA processing	0.02726236	DHX40, RCL1, SSB, DHX35, RTCA, HNRNPH3
	**GO Term Biological Process**	GO:0006369	termination of RNA polymerase II transcription	0.03078621	NUDT21, ZC3H11A, EIF4A3, RNPS1, SNRPF
	**GO Term Molecular Function**	GO:0004521	endoribonuclease activity	0.03640158	RCL1, PLD6, SMG6
	**GO Term Biological Process**	GO:0014070	response to organic cyclic compound	0.03726759	ATF1, FNTB, EIF4A3, MKI67
	**GO Term Cellular Component**	GO:0031251	PAN complex	0.0393125	PAN2, PAN3
	**GO Term Molecular Function**	GO:0003963	RNA-3'-phosphate cyclase activity	0.04030276	RCL1, RTCA
	**GO Term Biological Process**	GO:0010923	negative regulation of phosphatase activity	0.04795323	URI1, NIFK, ZCCHC9, RRP1B

	**GO Term Cellular Component**	GO:0005666	DNA-directed RNA polymerase III complex	0.04873867	POLR3A, POLR3H, POLR3K
**4**	**GO Term Biological Process**	GO:0007165	signal transduction	8.40E-05	CDS1, BCAR3, INPP1, NEDD9, IQGAP2, RASSF3, IMPA1, HIVEP2, NDFIP2, NDFIP1, DAPK1, CCL20, FST, ANXA4, RASSF8, ANK2, IRAK4, ACVR2B, MYO9A, TNFRSF10D, INPP4A, TOM1L1, CLDN4, TRAF4, TOLLIP, SMOC1, GNB2, PGAM5

	**GO Term Cellular Component**	GO:0005622	intracellular	1.17E-04	BCAR3, CLIC4, TRAF3IP2, POGK, AGAP1, TWF1, CAPN7, GOLGA1, COTL1, ZNF268, SOCS7, ZNF101, DFFB, RPH3AL, ZNF480, CCL20, TBC1D8, SERPINB9, ANK2, IRAK4, MYO9A, RAP2C, TOM1L1, TRAF4, RAB18, RUNDC1, TRIM38, RAPGEF5, TRIM32, SGK2, SOS2, ZNF772
	**GO Term Cellular Component**	GO:0005923	bicellular tight junction	6.06E-04	RAP2C, CLDN4, TRAF4, WNK4, ESAM, F11R, MPDZ, TJP3
	**GO Term Cellular Component**	GO:0031463	Cul3-RING ubiquitin ligase complex	7.07E-04	KLHL18, KLHL36, KCTD10, KLHL2, KCTD17, KCTD5

	**GO Term Cellular Component**	GO:0005886	plasma membrane	0.00126123	LMBR1L, CLIC4, SLC44A1, RAB3D, ITGB2, ANTXR2, F11R, PTPRF, LTB4R, PPP1R9B, RASSF3, SLC39A6, FAM21A, PTGFRN, ZHX2, DAPK1, ANXA4, ITGA2, LIFR, ANK2, IRAK4, PHKA2, IL17RB, RAP2C, CLDN4, TRAF4, CD46, MTMR1, SNAP47, TTYH3, ARHGEF25, MAPKAP1, EFNA4, ADD1, GRK5, GNG4, GNA12, FNIP2, SOCS7, GPR157, SLC12A2, RPH3AL, ATP2B4, ACVR2B, TNFRSF10D, IL22RA1, TSPAN14, GNB2, RAB18, ESAM, TAB2, TJP3, BMPR1A
	**KEGG Pathway**	hsa04070	Phosphatidylinositol signaling system	0.00185515	CDS1, INPP4A, PI4K2B, MTMR1, IMPA1, INPP1, IPMK

	**GO Term Cellular Component**	GO:0009897	external side of plasma membrane	0.00261591	TGFBR3, ENOX2, ITGA2, CTSV, ANTXR2, BMPR1A, CD276
	**GO Term Cellular Component**	GO:0000421	autophagosome membrane	0.00298956	ATG16L1, WIPI1, ATG14, TECPR1
	**KEGG Pathway**	hsa00562	Inositol phosphate metabolism	0.00299647	INPP4A, PI4K2B, MTMR1, IMPA1, INPP1, IPMK
	**GO Term Cellular Component**	GO:0009986	cell surface	0.00412544	CLIC4, ANXA4, ITGA2, ITGB2, IQGAP2, SYNJ2BP, IL17RB, DCBLD2, TGFBR3, TSPAN14, SLC39A6, PTGFRN, CD46
	**GO Term Biological Process**	GO:0001654	eye development	0.0051309	FOXC1, SMOC1, BLOC1S3, RAB18
	**GO Term Biological Process**	GO:0001974	blood vessel remodeling	0.00583102	FOXC1, NOL3, ACVR2B, AGT
	**GO Term Biological Process**	GO:0043123	positive regulation of I-kappaB kinase/NF-kappaB signaling	0.00586131	NDFIP2, NDFIP1, TRAF3IP2, CASP10, TAB2, TRIM38, IRAK4, TRIM32

	**GO Term Biological Process**	GO:0042981	regulation of apoptotic process	0.00600241	BNIP3L, STEAP3, RASSF3, TRAF4, CASP10, DAPK1, NOL3, SGK2, TNFRSF10D
	**KEGG Pathway**	hsa04060	Cytokine-cytokine receptor interaction	0.00658788	IL22RA1, CCL20, LIFR, ACVR2B, IL17RB, BMPR1A, TNFRSF10D
	**GO Term Biological Process**	GO:0043547	positive regulation of GTPase activity	0.00789689	BCAR3, RGS19, ARHGEF25, CCL20, ARHGEF19, AGAP1, ARHGEF10L, F11R, MYO9A, RGP1, HERC1, PGAM5, RAPGEF5, ARHGEF40, SOS2
	**GO Term Biological Process**	GO:0006661	phosphatidylinositol biosynthetic process	0.0079989	CDS1, INPP4A, PI4K2B, MTMR1, IMPA1
	**GO Term Molecular Function**	GO:0048185	activin binding	0.00852788	TGFBR3, FST, ACVR2B
	**KEGG Pathway**	hsa04514	Cell adhesion molecules (CAMs)	0.0087646	CLDN4, ITGB2, ESAM, F11R, PTPRF, CD276
	**GO Term Molecular Function**	GO:0032403	protein complex binding	0.01005956	ITGA2, GNB2, ITGB2, TWF1, PGAM5, CTSV, KCTD17, KCTD5, PTPRF

	**GO Term Molecular Function**	GO:0004871	signal transducer activity	0.01447995	NDFIP2, NDFIP1, GNG4, FST, TOLLIP, GNB2, GNA12, TRIM38
	**GO Term Molecular Function**	GO:0005089	Rho guanyl-nucleotide exchange factor activity	0.01635439	ARHGEF25, ARHGEF19, ARHGEF10L, ARHGEF40, SOS2
	**GO Term Biological Process**	GO:0019221	cytokine-mediated signaling pathway	0.01889625	IL22RA1, LIFR, IRAK4, SOCS7, IL17RB
	**GO Term Biological Process**	GO:0030308	negative regulation of cell growth	0.01895537	SERPINE2, GNG4, SH3BP4, AGT, DCBLD2, PPP1R9B
	**GO Term Molecular Function**	GO:0008157	protein phosphatase 1 binding	0.02045439	AKAP11, SHOC2, PPP1R9B
	**GO Term Biological Process**	GO:0035023	regulation of Rho protein signal transduction	0.02095275	ARHGEF25, ARHGEF19, ARHGEF10L, ARHGEF40, SOS2
	**GO Term Cellular Component**	GO:0034045	pre-autophagosomal structure membrane	0.02213296	ATG16L1, WIPI1, ATG14
	**GO Term Cellular Component**	GO:0015629	actin cytoskeleton	0.02563809	CLIC4, DAPK1, ARPC1B, KLHL2, TWF1, IQGAP2, ZNF268, PPP1R9B

	**GO Term Biological Process**	GO:0043647	inositol phosphate metabolic process	0.02663284	INPP4A, IMPA1, INPP1, IPMK
	**GO Term Cellular Component**	GO:0010008	endosome membrane	0.03235503	TOM1L1, STEAP3, NDFIP1, WIPI1, TAB2, IRAK4, ANTXR2
	**GO Term Cellular Component**	GO:0005834	heterotrimeric G-protein complex	0.03807341	RGS19, GNG4, GNA12
	**GO Term Molecular Function**	GO:0030742	GTP-dependent protein binding	0.04011866	RAB3D, HPS6, RAPGEF5
	**GO Term Biological Process**	GO:0006821	chloride transport	0.04094232	CLIC4, TTYH3, WNK4
	**GO Term Biological Process**	GO:0007264	small GTPase mediated signal transduction	0.04460741	RAP2C, BCAR3, RGS19, RAB3D, RAB18, AGAP1, RAPGEF5, SOS2
	**GO Term Biological Process**	GO:0035556	intracellular signal transduction	0.04475663	TGFBR3, AKAP11, TRAF3IP2, DAPK1, TOLLIP, WNK4, RAPGEF5, SOCS7, MYO9A, SGK2
	**GO Term Biological Process**	GO:0030501	positive regulation of bone mineralization	0.04478003	ACVR2B, BMPR1A, CD276
	**GO Term Biological Process**	GO:0043086	negative regulation of catalytic activity	0.04703281	ANXA4, CD46, FNIP2, PPP1R9B

	**GO Term Biological Process**	GO:0006954	inflammatory response	0.04818316	TPST1, CCL20, TOLLIP, ITGB2, F11R, LYZ, LTB4R, TNFRSF10D
	**GO Term Biological Process**	GO:0007186	G-protein coupled receptor signaling pathway	0.04896973	RGS19, GNG4, CCL20, GNB2, GNA12, AGT, LTB4R, GPR157
**5**	**GO Term Cellular Component**	GO:0005789	endoplasmic reticulum membrane	2.70E-11	LMAN2L, MGST3, XYLT2, MGST1, MGST2, PANX1, ARV1, RDH10, HSD17B2, MFSD3, BCAP29, PIP4K2B, SDF2, LRRC8E, OSBPL8, SEC24B, SEC16A, PLA2G2A, ABCC6, ALG2, ACSL5, ACSL4, ELOVL6, MOGS, DHCR24, DOLK, POMGNT2, DDOST, EXT2, NCEH1, SOAT1, RFT1, TYRO3, PREB, TMCC1, ERGIC2, UGT2B7, ERGIC1, SLC27A4

	**GO Term Cellular Component**	GO:0016021	integral component of membrane	6.38E-09	ABCD3, SFT2D3, GOLT1B, DENND5B, SLC35B1, MPV17L, XYLT2, NAGPA, HS6ST1, ACSM2B, TMEM187, PANX1, ARV1, ATP7B, OXER1, BCAP29, GGT1, SLC25A44, ABCD1, ABCC1, SLC30A3, SLC30A5, ABCC6, SLC39A11, ALG2, ACSL5, ACSL4, MOGS, ELOVL6, POMGNT2, POMGNT1, SLC39A14, ZDHHC17, DDOST, ATRN, CEACAM1, SOAT1, SLCO4A1, PITPNM2, TMEM5, RFT1, TYRO3, SLC29A4, PREB, CD302, ADPRM, SMIM20, ERGIC2, STEAP2, ERGIC1, UGT2B7, SLC27A4, SLC22A3, CLDND1, LMAN2L, MAOB, HEPACAM, MGST3, GLT8D1, MGST1, MGST2, LRP4, SULT1A1, RDH10, HSD17B2, LRRC8E, OSBPL8, TMEM86B, COA1, TMEM184B, SEMA4F, DHCR24, DOLK, VASN, COL1A1, CLCN6, EXT2, TSPAN13, NCEH1, OSTM1, MGAT4A, C4ORF3, CYSTM1, TMCC1, MCU

	**GO Term Cellular Component**	GO:0016020	membrane	1.60E-07	ABCD3, SFT2D3, GOLT1B, DENND5B, HEXB, XYLT2, PANX1, GOLGA3, LGALS3, ATP7B, SCP2, DPYSL2, PALM, BCAP29, CAPN1, PIP4K2C, SDF2, ABCD1, CTSC, ABCC1, SLC30A3, SLC30A5, SLC39A11, ALG2, ACSL5, ACSL4, MOGS, LARP4B, DDOST, F5, EML4, DNAJC3, CEACAM1, RILPL2, SOAT1, DNAJB11, PREB, CD302, ERGIC2, ERGIC1, UGT2B7, SLC27A4, SLC22A3, LMAN2L, MGST3, GLT8D1, EDEM3, OSBPL8, SEC24B, TMEM86B, API5, EDEM2, SEMA4F, DHCR24, GBAS, EXT2, TSPAN13, NCEH1, MGAT4A

	**KEGG Pathway**	hsa05204:Chemical carcinogenesis		7.76E-07	SULT1A1, CBR1, ADH4, MGST3, GSTA1, MGST1, MGST2, ADH5, UGT2B7
	**KEGG Pathway**	hsa00982:Drug metabolism - cytochrome P450		1.39E-06	ADH4, MAOB, MGST3, GSTA1, MGST1, MGST2, ADH5, UGT2B7
	**KEGG Pathway**	hsa01100:Metabolic pathways		1.42E-06	ALAS1, MAOB, GLDC, HEXB, AKR1D1, XYLT2, GNS, ADH5, ACAT2, PTS, ACSM2B, SGSH, GMPPB, ADH4, SCP2, IMPA2, RDH10, HSD17B2, GGT1, GLUL, CBR1, PLA2G12B, GK, PLA2G2A, ALG2, ACSL5, ACSL4, MOGS, DHCR24, DOLK, DDOST, PAFAH2, AGMAT, EXT2, MINPP1, BHMT, AKR1B10, ACOX2, MGAT4A, UGT2B7

	**GO Term Biological Process**	GO:0098869	cellular oxidant detoxification	3.06E-06	AAED1, APOM, GPX3, MGST3, GSR, GSTA1, MGST1, HP, MGST2
	**KEGG Pathway**	hsa00980:Metabolism of xenobiotics by cytochrome P450		5.97E-06	CBR1, ADH4, MGST3, GSTA1, MGST1, MGST2, ADH5, UGT2B7
	**GO Term Cellular Component**	GO:0005783	endoplasmic reticulum	1.74E-05	GOLT1B, MGST3, MGST1, MGST2, PANX1, BCAP29, EDEM3, MESDC2, OSBPL8, PLA2G2A, EDEM2, ACSL5, SEMA4F, ELOVL6, MOGS, DHCR24, POMGNT2, DHRS1, SLC39A14, DDOST, DNAJC3, EXT2, MINPP1, TRAPPC6A, SOAT1, DNAJB11, SLC27A4

	**GO Term Cellular Component**	GO:0070062	extracellular exosome	2.70E-05	HEXB, SERPINA10, HP, LIPA, SGSH, GMPPB, LGALS3, SCP2, DPYSL2, CAPN1, PIP4K2C, GGT1, GLUL, CTSC, CTSB, CBR1, ABCC1, GPX3, AHSG, LANCL1, ACSL4, MOGS, PTGR1, DNAJC3, ATRN, MINPP1, CEACAM1, RILPL2, MASP2, HAGH, UGT2B7, CFD, MAOB, SERPINC1, MGST3, AKR1D1, BPHL, GNS, ADH5, ACAT2, CPN2, CRYZ, APOM, ISOC1, GK, PLA2G2A, GSR, DHRS2, VASN, AGMAT, DERA, EXT2, TF, BHMT, AKR1B10, FABP5, MGAT4A, GLOD4, GSTA1, CYSTM1
	**GO Term Biological Process**	GO:0006805	xenobiotic metabolic process	3.59E-05	SULT1A1, NCEH1, MGST3, MGST1, MGST2, GGT1, BPHL, ACSM2B

	**GO Term Molecular Function**	GO:0008375	acetylglucosaminyltransferase activity	4.69E-05	EXT2, HEXB, XYLT2, POMGNT2, POMGNT1
	**GO Term Biological Process**	GO:0008152	metabolic process	6.03E-05	NCEH1, SCP2, PITPNM2, GSTA1, STARD9, ISOC1, ACSL4, FAHD2A, UGT2B7, ACSM2B, SGSH
	**GO Term Biological Process**	GO:0055114	oxidation-reduction process	8.33E-05	AAED1, CBR1, MAOB, GLDC, NCF2, GPX3, GSR, AKR1D1, MGST1, DHCR24, PTGR1, DHRS1, DHRS2, CRYZ, AKR1B10, RDH10, HSD17B2, PIR, RTN4IP1, STEAP2, MSRB1
	**KEGG Pathway**	hsa00480:Glutathione metabolism		1.12E-04	GPX3, MGST3, GSR, GSTA1, MGST1, MGST2, GGT1
	**GO Term Molecular Function**	GO:0004602	glutathione peroxidase activity	1.15E-04	GPX3, MGST3, GSTA1, MGST1, MGST2

	**GO Term Cellular Component**	GO:0005887	integral component of plasma membrane	4.50E-04	SLC22A3, NAGPA, HS6ST1, TSPAN33, APOM, ATP7B, PALM, MFSD3, BCAP29, LRRC8E, ABCC1, TMEM150A, SLC30A3, SLC30A5, LANCL1, SEMA4F, GBAS, SLC39A14, ATRN, CEACAM1, TSPAN13, SLCO4A1, TMEM5, TYRO3, STEAP2
	**KEGG Pathway**	hsa03320:PPAR signaling pathway		5.80E-04	ACOX2, GK, SCP2, FABP5, ACSL5, ACSL4, SLC27A4
	**GO Term Cellular Component**	GO:0000139	Golgi membrane	7.25E-04	SEC24B, GOLT1B, LMAN2L, SEC16A, XYLT2, DHCR24, POMGNT1, HS6ST1, ZDHHC17, F5, GOLGA3, EXT2, TRAPPC6A, ATP7B, MGAT4A, TMEM5, PREB, ERGIC1, CTSC
	**GO Term Molecular Function**	GO:0005215	transporter activity	7.67E-04	GOLGA3, SLC22A3, SEC24B, ABCC1, TMEM184B, FABP5, SLCO4A1, ABCC6, STEAP2, ABCD1

	**GO Term Molecular Function**	GO:0015485	cholesterol binding	0.00207146	OSBPL8, STARD4, STARD5, SCP2, SOAT1
	**KEGG Pathway**	hsa04146:Peroxisome		0.00224239	ABCD3, ACOX2, SCP2, MPV17L, ACSL5, ACSL4, ABCD1
	**GO Term Cellular Component**	GO:0043231	intracellular membrane-bounded organelle	0.00224418	ABCD3, MGST3, SLC35B1, MPV17L, MGST2, BPHL, ZDHHC17, DDOST, OSTM1, SCP2, ACOX2, RDH10, PITPNM2, PALM, STEAP2, ERGIC2, UGT2B7, CTSB
	**GO Term Biological Process**	GO:0071577	zinc II ion transmembrane transport	0.00225676	SLC30A3, SLC30A5, SLC39A11, SLC39A14
	**GO Term Biological Process**	GO:1901687	glutathione derivative biosynthetic process	0.00225676	MGST3, GSTA1, MGST1, MGST2
	**GO Term Biological Process**	GO:0042760	very long-chain fatty acid catabolic process	0.00274258	ABCD3, ABCD1, SLC27A4

	**GO Term Cellular Component**	GO:0005739	mitochondrion	0.00284884	ALAS1, MAOB, GLDC, MPV17L, MGST1, BPHL, ADH5, ACAT2, PTS, ACSM2B, GMPPB, ATP7B, SCP2, CA5A, DPYSL2, CAPN1, GLUL, ABCD1, CTSB, STARD4, COA1, STARD5, GK, GSR, ACSL5, GBAS, DHRS2, VASN, AGMAT, ACOX2, LACTB2, GLOD4, MCU
	**GO Term Biological Process**	GO:0048208	COPII vesicle coating	0.00289325	TRAPPC6A, SEC24B, SEC16A, PREB, CTSC, F5
	**GO Term Biological Process**	GO:0006629	lipid metabolic process	0.00315851	FABP5, MGST3, ACSL4, LIPA, UGT2B7, ACAT2, PAFAH2, SLC27A4
	**KEGG Pathway**	hsa00590:Arachidonic acid metabolism		0.00381021	CBR1, PLA2G12B, GPX3, PLA2G2A, GGT1
	**KEGG Pathway**	hsa00071:Fatty acid degradation		0.00470031	ADH4, ACSL5, ACSL4, ADH5, ACAT2

	**GO Term Molecular Function**	GO:0004364	glutathione transferase activity	0.00550645	MGST3, GSTA1, MGST1, MGST2
	**GO Term Molecular Function**	GO:0016491	oxidoreductase activity	0.00607712	MAOB, GSR, HSD17B2, RTN4IP1, PTGR1, DHRS1, DHRS2, ADH5, CRYZ
	**GO Term Biological Process**	GO:0001523	retinoid metabolic process	0.00631386	ADH4, APOM, AKR1B10, RDH10, ADH5
	**GO Term Molecular Function**	GO:0016757	transferase activity, transferring glycosyl groups	0.00646279	EXT2, ALG2, GLT8D1, POMGNT2, DDOST
	**GO Term Cellular Component**	GO:0005778	peroxisomal membrane	0.00816829	ABCD3, MPV17L, MGST1, ACSL4, ABCD1
	**GO Term Cellular Component**	GO:0005788	endoplasmic reticulum lumen	0.00857925	EDEM3, DNAJC3, COL1A1, MINPP1, EDEM2, DNAJB11, CTSC, F5
	**GO Term Biological Process**	GO:0016042	lipid catabolic process	0.00859035	NCEH1, PLA2G12B, PLA2G2A, LIPA, PAFAH2
	**KEGG Pathway**	hsa04141:Protein processing in endoplasmic reticulum		0.00901497	EDEM3, DNAJC3, SEC24B, EDEM2, DNAJB11, MOGS, PREB, CAPN1, DDOST

	**KEGG Pathway**	hsa00510:N-Glycan biosynthesis		0.01042918	MGAT4A, ALG2, MOGS, DOLK, DDOST
	**GO Term Molecular Function**	GO:0009055	electron carrier activity	0.0111149	MAOB, ACOX2, NCF2, GLDC, GSR, ADH5
	**GO Term Biological Process**	GO:0006888	ER to Golgi vesicle-mediated transport	0.0114774	TRAPPC6A, SEC24B, LMAN2L, BCAP29, PREB, CTSC, ERGIC1, F5
	**GO Term Biological Process**	GO:0010243	response to organonitrogen compound	0.01167361	MGST3, MGST1, MGST2
	**GO Term Molecular Function**	GO:0004467	long-chain fatty acid-CoA ligase activity	0.01367883	ACSL5, ACSL4, SLC27A4
	**GO Term Biological Process**	GO:0006691	leukotriene metabolic process	0.01411049	ABCC1, PTGR1, GGT1
	**GO Term Cellular Component**	GO:0005777	peroxisome	0.01436315	ABCD3, ACOX2, SCP2, MPV17L, ISOC1, ABCD1
	**GO Term Molecular Function**	GO:0042626	ATPase activity, coupled to transmembrane movement of substances	0.01441151	ABCC1, ABCD3, ABCC6, ABCD1
	**GO Term Biological Process**	GO:0033540	fatty acid beta-oxidation using acyl-CoA oxidase	0.01674622	ACOX2, SCP2, ABCD1

	**KEGG Pathway**	hsa00565:Ether lipid metabolism		0.01748894	TMEM86B, PLA2G12B, PLA2G2A, PAFAH2
	**GO Term Cellular Component**	GO:0005741	mitochondrial outer membrane	0.01825511	MAOB, GK, MGST1, ACSL5, ACSL4, RTN4IP1, BPHL
	**KEGG Pathway**	hsa00830:Retinol metabolism		0.01916691	ADH4, RDH10, ADH5, UGT2B7
	**GO Term Biological Process**	GO:0006882	cellular zinc ion homeostasis	0.01957346	ATP7B, SLC30A5, SLC39A14
	**KEGG Pathway**	hsa04142:Lysosome		0.02083955	HEXB, NAGPA, LIPA, GNS, CTSC, SGSH, CTSB
	**GO Term Cellular Component**	GO:1903561	extracellular vesicle	0.02203176	DNAJC3, CBR1, OLFML3, F5
	**GO Term Biological Process**	GO:0006750	glutathione biosynthetic process	0.02258506	MGST2, HAGH, GGT1
	**KEGG Pathway**	hsa02010:ABC transporters		0.0227817	ABCC1, ABCD3, ABCC6, ABCD1
	**GO Term Biological Process**	GO:0006749	glutathione metabolic process	0.02417616	GSR, GSTA1, MGST1, GGT1
	**GO Term Molecular Function**	GO:0004252	serine-type endopeptidase activity	0.02543915	CFD, HP, MASP2, CTSC, CTSB, F5

	**GO Term Biological Process**	GO:0006635	fatty acid beta-oxidation	0.02593013	ABCD3, ACOX2, ABCD1, ACAT2
	**KEGG Pathway**	hsa00260:Glycine, serine and threonine metabolism		0.02674213	BHMT, ALAS1, MAOB, GLDC
	**GO Term Biological Process**	GO:0035269	protein O-linked mannosylation	0.02913368	TMEM5, SDF2, POMGNT2
	**GO Term Biological Process**	GO:0007031	peroxisome organization	0.02913368	ABCD3, SCP2, ABCD1
	**GO Term Biological Process**	GO:0006491	N-glycan processing	0.02913368	EDEM3, MGAT4A, EDEM2
	**GO Term Cellular Component**	GO:0005764	lysosome	0.03028525	TMEM150A, AKR1B10, CAPN1, LIPA, GNS, CTSC, SGSH, CTSB
	**GO Term Biological Process**	GO:0055085	transmembrane transport	0.03054357	SLC22A3, ABCC1, ABCD3, SLC35B1, ABCC6, MFSD3, ABCD1, SLC25A44
	**KEGG Pathway**	hsa00531:Glycosaminoglycan degradation		0.03260796	HEXB, GNS, SGSH
	**GO Term Biological Process**	GO:0008207	C21-steroid hormone metabolic process	0.03348328	AKR1D1, DHRS2
	**GO Term Biological Process**	GO:0015910	peroxisomal long-chain fatty acid import	0.03348328	ABCD3, ABCD1

	**GO Term Cellular Component**	GO:0005615	extracellular space	0.03367298	CFD, IGFBP1, AHSG, SERPINC1, GPX3, HEXB, PLA2G2A, SERPINA10, HP, SEMA4F, VASN, F5, COL1A1, ATRN, LGALS3, TF, SDF2, GGT1, CTSC, CTSB
	**GO Term Molecular Function**	GO:0005385	zinc ion transmembrane transporter activity	0.03527053	SLC30A5, SLC39A11, SLC39A14
	**GO Term Biological Process**	GO:0001676	long-chain fatty acid metabolic process	0.03633849	ACSL5, ACSL4, SLC27A4
	**KEGG Pathway**	hsa00120:Primary bile acid biosynthesis		0.03680793	ACOX2, SCP2, AKR1D1
	**GO Term Molecular Function**	GO:0016209	antioxidant activity	0.03899721	AAED1, APOM, HP
	**GO Term Biological Process**	GO:0098656	anion transmembrane transport	0.04017098	ABCC1, ABCC6, LRRC8E
	**GO Term Biological Process**	GO:0006699	bile acid biosynthetic process	0.04017098	ACOX2, SCP2, AKR1D1
	**GO Term Biological Process**	GO:0006486	protein glycosylation	0.04109547	EXT2, SDF2, NAGPA, POMGNT1, DDOST

	**KEGG Pathway**	hsa00534:Glycosaminoglycan biosynthesis - heparan sulfate / heparin		0.04120341	EXT2, XYLT2, HS6ST1
	**KEGG Pathway**	hsa01212:Fatty acid metabolism		0.04600085	ACSL5, ACSL4, ELOVL6, ACAT2
	**GO Term Molecular Function**	GO:0004022	alcohol dehydrogenase (NAD) activity	0.04901067	ADH4, ADH5
	**GO Term Molecular Function**	GO:0016309	1-phosphatidylinositol-5-phosphate 4-kinase activity	0.04901067	PIP4K2B, PIP4K2C

**6**	**KEGG Pathway**	hsa01100	Metabolic pathways	7.96E-17	MOCS2, PANK3, PIGO, ABAT, MTR, PIGW, PFAS, PIGV, PPCDC, RFK, PGM2, ACADM, ENPP3, HADH, SEPHS2, HIBCH, PGM1, ALG8, ALG6, CHPF2, ALG14, SORD, HOGA1, PIGB, PIGK, IDNK, UMPS, B4GALT7, PIGF, MOCS1, B4GALT4, ST6GALNAC6, LIPT1, DDC, COX17, MRI1, DLST, PDHB, ATP5L, NFS1, CHSY1, AFMID, ST3GAL6, ASL, ARSB, NDUFA8, C1GALT1, TRIT1, COQ6, RPIA, GALE, P4HA1, OGDH

	**GO Term Cellular Component**	GO:0005739	mitochondrion	1.98E-14	MIPEP, SLC35F6, SRP19, ISCA2, RMND1, ABAT, MRPL34, MRPL32, PROSC, CHCHD3, RFK, CDK5RAP1, ACADM, HADH, COA6, HIBCH, METTL17, HEMK1, TSFM, PPTC7, MRPS22, ACAD9, C10ORF10, MRPL49, HOGA1, MRPL45, MRPL46, GCAT, CLPX, PYURF, OXLD1, RPUSD2, SDHAF1, MTFR1, L2HGDH, ATPAF1, LIPT1, MRPS36, TIMM9, COX17, DLST, GLRX, PDHB, TMEM70, ATP5L, NFS1, MARC2, C6ORF203, MARC1, TNRC18, ARSB, NDUFA8, APOOL, NUBPL, DNAJC11, TRIT1, COQ6, DNAJA1, P4HA1, NDUFAF4, OGDH

	**GO Term Cellular Component**	GO:0005743	mitochondrial inner membrane	3.36E-09	MRPS36, TIMM9, MRPL19, SQRDL, MRPL34, MRPL32, TMEM70, ATP5L, CHCHD3, MARC2, MARC1, HADH, RHBDL2, CYB5B, NDUFA8, MRPS22, ACAD9, MRPL49, DNAJC11, MRPL45, MRPL46, CLPX, GCAT, COQ6, MRPL50, NDUFAF4, L2HGDH, NDUFAF1
	**GO Term Biological Process**	GO:0006506	GPI anchor biosynthetic process	3.48E-09	PIGB, PYURF, PIGO, PGAP3, PIGZ, MPPE1, PIGW, PIGF, PIGV
	**KEGG Pathway**	hsa00532	Glycosaminoglycan biosynthesis - chondroitin sulfate / dermatan sulfate	1.68E-07	CHST7, CHSY1, CHPF2, CHST15, CHST13, CHST14, B4GALT7
	**KEGG Pathway**	hsa00563	Glycosylphosphatidylinositol(GPI)-anchor biosynthesis	2.53E-06	PIGB, PIGO, PIGK, PIGZ, PIGW, PIGF, PIGV
	**GO Term Biological Process**	GO:0070125	mitochondrial translational elongation	1.19E-05	TSFM, MRPL50, MRPS36, MRPS22, MRPL19, MRPL49, MRPL34, MRPL45, MRPL46, MRPL32

	**GO Term Biological Process**	GO:0030206	chondroitin sulfate biosynthetic process	1.28E-05	CHST7, SLC35D1, CHSY1, CHPF2, CHST15, CHST13
	**GO Term Molecular Function**	GO:0016740	transferase activity	3.81E-05	ALG8, CHST15, CDK5RAP1, DLST, GLRX, CASD1, UMPS, PFAS, TRIT1
	**GO Term Biological Process**	GO:0070126	mitochondrial translational termination	1.02E-04	MRPL50, MRPS36, MRPS22, MRPL19, MRPL49, MRPL34, MRPL45, MRPL46, MRPL32
	**GO Term Cellular Component**	GO:0005759	mitochondrial matrix	1.07E-04	MIPEP, ISCA2, LIPT1, TSFM, NUBPL, DLST, ABAT, PDHB, TRIT1, CLPX, NFS1, SDHAF1, OGDH, ACADM, HADH, HIBCH, CBR4
	**GO Term Molecular Function**	GO:0030170	pyridoxal phosphate binding	1.27E-04	PROSC, DDC, NFS1, MARC2, MARC1, ABAT, GCAT
	**GO Term Molecular Function**	GO:0051536	iron-sulfur cluster binding	1.70E-04	NFS1, GLRX3, TYW1, CDK5RAP1, ABAT

	**GO Term Cellular Component**	GO:0005789	endoplasmic reticulum membrane	2.20E-04	TRAM1, SLC35D1, PIGO, FKBP14, PIGZ, PIGW, PIGV, UFL1, NCLN, SEC61G, TMEM129, CREB3L2, DNAJB9, SEC11C, NUS1, CYB5A, ALG8, ALG6, SSR3, ALG14, ERLIN1, PYURF, PIGB, ERP44, PIGK, SRPRB, PIGF
	**GO Term Molecular Function**	GO:0003824	catalytic activity	4.87E-04	NFS1, AASDH, MARC2, MARC1, TYW1, CDK5RAP1, CDYL, MOCS1, GLA, GCAT, PPCDC

	**GO Term Cellular Component**	GO:0016021	integral component of membrane	5.18E-04	SLC35F6, TRAM1, PIGO, PGAP3, GOLT1A, SLC35F5, PIGZ, PIGW, PIGV, SCAMP5, PCMT1, SEC61G, CREB3L2, ACP2, ENPP3, ALG8, CHST7, PAQR7, ALG6, CHPF2, MMD, SSR3, ALG14, HOGA1, MANEAL, CDYL, CASD1, TPST2, FRMD3, PIGB, TMEM2, SLC5A9, L2HGDH, MPPE1, PPP1R12B, B4GALT7, PIGF, B4GALT4, ST6GALNAC6, SLC26A2, TP53I13, SLC35D1, AASDH, COX18, THADA, C4ORF32, TMTC4, NCLN, CHSY1, C16ORF91, MARC2, TMEM129, ARSK, MARC1, CHST15, TYW1, ST3GAL6, CHST13, LRIG2, CHST14, APOL1, RHBDL2, SEC11C, CYB5B, SLC35A2, NUS1, CYB5A, C1GALT1, ERLIN1, TMEM230, WDR11, RPIA, DPY19L4, SRPRB

	**GO Term Biological Process**	GO:0097502	mannosylation	5.29E-04	PIGB, ALG8, PIGZ, PIGV
	**GO Term Biological Process**	GO:0046487	glyoxylate metabolic process	7.69E-04	LIPT1, OGDH, DLST, HOGA1, PDHB
	**GO Term Molecular Function**	GO:0000030	mannosyltransferase activity	9.85E-04	PIGB, DPY19L4, PIGZ, PIGV
	**GO Term Molecular Function**	GO:0016757	transferase activity, transferring glycosyl groups	0.00100242	PIGB, PIGZ, GYG2, B4GALT7, B3GNTL1, B4GALT4
	**KEGG Pathway**	hsa01130	Biosynthesis of antibiotics	0.00107115	RPIA, OGDH, PGM2, DLST, ASL, IDNK, ACADM, PDHB, HADH, PFAS, PGM1
	**GO Term Cellular Component**	GO:0005762	mitochondrial large ribosomal subunit	0.00151651	MRPL50, MRPL19, MRPL49, MRPL34, MRPL46, MRPL32
	**GO Term Biological Process**	GO:0016254	preassembly of GPI anchor in ER membrane	0.0018838	PIGB, PIGW, PIGF, PIGV
	**GO Term Biological Process**	GO:0006687	glycosphingolipid metabolic process	0.00309808	NEU3, ARSK, GLA, ARSB, ST6GALNAC6
	**GO Term Molecular Function**	GO:0051539	4 iron, 4 sulfur cluster binding	0.00397377	ISCA2, NUBPL, TYW1, CDK5RAP1, MOCS1

	**GO Term Biological Process**	GO:0019388	galactose catabolic process	0.00410899	GALE, PGM2, PGM1
	**GO Term Biological Process**	GO:0032324	molybdopterin cofactor biosynthetic process	0.00410899	MOCS2, NFS1, MOCS1
	**GO Term Biological Process**	GO:0006777	Mo-molybdopterin cofactor biosynthetic process	0.00410899	MOCS2, NFS1, MOCS1
	**GO Term Biological Process**	GO:0055114	oxidation-reduction process	0.00457042	CYB5B, CYB5A, NDUFA8, GLRX3, MRPS36, SORD, GLRX, COQ6, P4HA1, MARC2, MARC1, TYW1, ACADM, L2HGDH, HIF1AN, PIGF, CBR4
	**GO Term Biological Process**	GO:0005975	carbohydrate metabolic process	0.00516097	NEU3, PGM2, IDNK, B4GALT7, GLA, PGM1, GALK2, B4GALT4
	**GO Term Biological Process**	GO:0005978	glycogen biosynthetic process	0.00584322	PGM2, ACADM, GYG2, PGM1
	**KEGG Pathway**	hsa00052	Galactose metabolism	0.00628912	GALE, PGM2, GLA, PGM1
	**KEGG Pathway**	hsa01200	Carbon metabolism	0.00670813	RPIA, OGDH, DLST, IDNK, ACADM, PDHB, HIBCH

	**KEGG Pathway**	hsa03060	Protein export	0.00714202	SRP19, SEC61G, SRPRB, SEC11C
	**GO Term Biological Process**	GO:0006012	galactose metabolic process	0.0075003	SLC35A2, GALE, GALK2
	**KEGG Pathway**	hsa00030	Pentose phosphate pathway	0.00904505	RPIA, PGM2, IDNK, PGM1
	**GO Term Biological Process**	GO:0006400	tRNA modification	0.00938754	QTRT1, CDK5RAP1, TRIT1, ADAT2
	**GO Term Cellular Component**	GO:0000139	Golgi membrane	0.00981268	SLC35A2, CHST7, GOLT1A, CHPF2, PGAP3, C1GALT1, MANEAL, SCAMP5, TPST2, CHSY1, CHST15, ST3GAL6, CHST13, CHST14, B4GALT7, B4GALT4, ST6GALNAC6
	**GO Term Cellular Component**	GO:0005761	mitochondrial ribosome	0.01163487	MRPS22, MRPL49, MRPL34, MRPL32
	**GO Term Biological Process**	GO:0008535	respiratory chain complex IV assembly	0.01178791	COX18, COA6, METTL17
	**KEGG Pathway**	hsa04122	Sulfur relay system	0.0124701	MOCS2, NFS1, MOCS1

	**GO Term Cellular Component**	GO:0005788	endoplasmic reticulum lumen	0.01301724	ERP44, P4HA1, FKBP14, ARSK, DNAJB9, UGGT2, COLGALT1, ARSB
	**KEGG Pathway**	hsa00380	Tryptophan metabolism	0.01786815	DDC, AFMID, OGDH, HADH
	**GO Term Biological Process**	GO:0032981	mitochondrial respiratory chain complex I assembly	0.02088426	NDUFA8, NUBPL, NDUFAF4, ACAD9, NDUFAF1
	**GO Term Molecular Function**	GO:0051082	unfolded protein binding	0.02114161	DNAJA1, CCDC115, DNAJA2, NDUFAF1, UGGT2, CLPX
	**GO Term Biological Process**	GO:0008152	metabolic process	0.02180758	AASDH, AFMID, ARSK, DLST, HOGA1, CDYL, ARSB
	**GO Term Cellular Component**	GO:0005840	ribosome	0.02210142	MRPL50, MRPS22, MRPL19, MRPL49, MRPL34, MRPL45, MRPL32, METTL17
	**GO Term Biological Process**	GO:0006488	dolichol-linked oligosaccharide biosynthetic process	0.02601909	ALG8, ALG6, ALG14
	**KEGG Pathway**	hsa00770	Pantothenate and CoA biosynthesis	0.02749518	PANK3, ENPP3, PPCDC
	**GO Term Biological Process**	GO:0009311	oligosaccharide metabolic process	0.029409	ST3GAL6, GLA, ST6GALNAC6

	**GO Term Molecular Function**	GO:0046872	metal ion binding	0.0302646	ZNF232, MIPEP, TIMM9, ABAT, PFAS, ZNF26, NFS1, CHSY1, ZNF706, ZNF407, RFK, TMEM129, ARSK, CDK5RAP1, TYW1, ADNP2, ENPP3, ZNF500, ZNF740, ARSB, ZBED3, CYB5B, ZNF561, CYB5A, GLRX3, PPTC7, NUBPL, C1GALT1, CDC42BPA, CLPX, QTRT1, DNAJA1, THAP5, ZFYVE19, DNAJA2, SPRTN, OGDH, MOCS1, B4GALT4
	**GO Term Cellular Component**	GO:0043202	lysosomal lumen	0.03205868	NAAA, ACP2, GYG2, GLA, ARSB
	**GO Term Molecular Function**	GO:0051087	chaperone binding	0.03233565	DNAJA1, TIMM9, DNAJA2, OGDH, AHSA2
	**GO Term Biological Process**	GO:0006103	2-oxoglutarate metabolic process	0.03296408	MRPS36, OGDH, L2HGDH

	**GO Term Biological Process**	GO:0070900	mitochondrial tRNA modification	0.03365266	CDK5RAP1, TRIT1
	**GO Term Biological Process**	GO:0051410	detoxification of nitrogen compound	0.03365266	MARC2, MARC1
	**GO Term Biological Process**	GO:0006490	oligosaccharide-lipid intermediate biosynthetic process	0.03365266	ALG8, ALG6
	**GO Term Biological Process**	GO:0006505	GPI anchor metabolic process	0.03365266	PGAP3, PIGW
	**GO Term Biological Process**	GO:0042126	nitrate metabolic process	0.03365266	MARC2, MARC1
	**GO Term Molecular Function**	GO:0008940	nitrate reductase activity	0.03411766	MARC2, MARC1
	**GO Term Molecular Function**	GO:0042281	dolichyl pyrophosphate Man9GlcNAc2 alpha-1,3-glucosyltransferase activity	0.03411766	ALG8, ALG6
	**GO Term Molecular Function**	GO:0004376	glycolipid mannosyltransferase activity	0.03411766	PIGB, PIGV
	**GO Term Cellular Component**	GO:0019008	molybdopterin synthase complex	0.03501875	MOCS2, MOCS1
	**KEGG Pathway**	hsa00280	Valine, leucine and isoleucine degradation	0.0459559	ABAT, ACADM, HADH, HIBCH

	**GO Term Biological Process**	GO:0005980	glycogen catabolic process	0.04870827	PGM2, GYG2, PGM1
	**GO Term Biological Process**	GO:0006479	protein methylation	0.04870827	N6AMT1, HEMK1, PCMT1
